# Supplementary material for: A novel score system of blood tests for differentiating Kawasaki disease from febrile children
Source: PLoS One. 2021 Jan 22;16(1):e0244721. doi: 10.1371/journal.pone.0244721 (PMC7822339; doi:10.1371/journal.pone.0244721)
Supplement: S1 Data — (PDF) [file pone.0244721.s001.pdf]

index	hospital	typicalKD group (0: I	SEX (0: F(	AGE (year	WBC (100	RBC (mill	Hemoglob
1	0	0	0	1	23.6	3.69	9
2	0	0	0	1	11.8	4.55	12.5
3	0	0	0	0	9.8	4.19	10.9
4	0	0	1	3	13	5.02	13.4
5	0	0	0	4	10.5	4.27	12.3
6	0	0	1	4	14.2	4.63	12.5
7	0	0	1	4	13.2	4.31	11.6
8	0	0	0	4	8.1	4.55	11.4
9	0	0	0	4	13.4	4.73	12.4
10	0	0	1	4	7.2	4.33	11.5
11	0	0	1	4	10.4	4.22	10.8
12	0	0	1	4	16.7	4.94	11.6
13	0	0	1	4	19.7	4.67	13.1
14	0	0	0	4	12.4	4.71	13.4
15	0	0	1	4	8.2	4.68	13
16	0	0	1	4	8.6	4.06	11.5
17	0	0	1	4	15.1	4.55	13.1
18	0	0	1	4	6.9	4.7	11.5
19	0	0	1	4	23	4.75	13.4
20	0	0	1	4	8.1	4.56	12.6
21	0	0	1	4	20.3	4.04	11.7
22	0	0	1	4	17.1	4.45	11.9
23	0	0	0	4	3.6	4.4	11.7
24	0	0	1	4	13.8	4.92	13.2
25	0	0	1	4	17.9	4.68	12.6
26	0	0	1	4	8.2	4.3	11.5
27	0	0	1	4	14.3	4.31	11.5
28	0	0	1	4	9.8	4.88	13.2
29	0	0	0	4	5.2	4.16	12
30	0	0	1	4	19	5.38	12.5
31	0	0	0	4	28.8	4.75	14.1
32	0	0	1	4	8.2	4.47	12.4
33	0	0	0	3	7	4.04	10.9
34	0	0	1	4	10	5.08	13.2
35	0	0	1	4	14.7	4.41	11.9
36	0	0	1	3	13.8	4.79	12.7
37	0	0	1	4	4.3	4.7	12.9
38	0	0	0	4	7.9	4.13	10.9
39	0	0	1	4	3.4	4.67	13.1
40	0	0	1	4	9.1	4.42	11.7
41	0	0	0	3	5.2	4.79	12.9
42	0	0	1	3	8.3	4.16	11.4
43	0	0	1	4	5.7	4.4	12.2
44	0	0	0	3	17.2	4.95	12.6
45	0	0	1	4	12.7	3.86	10.9

46	0	0	0	3	10	4.49	12.6
47	0	0	1	3	10.7	4.81	13.5
48	0	0	1	4	15.6	4.25	11.6
49	0	0	0	3	9.5	4.44	12.5
50	0	0	1	4	7.9	4.41	11.5
51	0	0	1	3	12.2	4.53	12.7
52	0	0	1	4	6.2	5	13.4
53	0	0	1	4	18.7	4.43	12.2
54	0	0	1	3	22.3	5.39	14.9
55	0	0	0	3	13.6	4.32	12.1
56	0	0	1	3	10.8	4.6	12.6
57	0	0	1	3	10.4	4.87	13
58	0	0	1	3	11.6	4.68	13
59	0	0	0	4	7.9	4.49	12.7
60	0	0	0	3	8.8	4.05	11.4
61	0	0	1	4	4.6	4.43	11.8
62	0	0	0	3	19.8	4.67	12.5
63	0	0	0	4	9.5	2.28	5.9
64	0	0	1	3	8	4.23	11.9
65	0	0	0	3	2.4	5.08	12.8
66	0	0	0	2	7	4.03	11.3
67	0	0	1	3	8.5	5.17	14.3
68	0	0	0	3	19.4	4.25	12
69	0	0	0	2	16.1	4.36	11.6
70	0	0	1	3	14.2	4.75	13.2
71	0	0	0	2	13.4	4.76	11.8
72	0	0	0	2	21.3	3.93	10.5
73	0	0	0	2	12.5	4.25	11.5
74	0	0	1	2	20.4	5.02	13
75	0	0	0	3	9.9	4.35	11.7
76	0	0	0	3	12.3	4.88	12.7
77	0	0	0	4	10	4.3	12
78	0	0	0	3	5.7	4.63	12.7
79	0	0	1	4	10	3.9	10.7
80	0	0	1	2	15.7	4.82	12.9
81	0	0	0	2	6.6	4.7	12.4
82	0	0	1	3	29.2	4.53	11.7
83	0	0	0	3	8.3	4.16	12.3
84	0	0	1	2	7.4	4.66	12.8
85	0	0	1	2	18.3	5.3	14.2
86	0	0	1	2	5.4	4.49	11.5
87	0	0	1	2	12.4	5.08	12.1
88	0	0	0	3	12.6	4.32	11.5
89	0	0	0	4	9.8	4.28	11.7
90	0	0	0	3	12.1	4.33	12.3
91	0	0	1	2	9.2	4.42	12

92	0	0	0	3	14.5	4.38	11.8
93	0	0	0	4	9.8	4.73	13.8
94	0	0	0	2	18.7	4.9	12.3
95	0	0	1	4	20	4.36	11.7
96	0	0	0	4	15.5	4.01	10.6
97	0	0	0	2	7.5	4.43	12.2
98	0	0	1	2	20.7	4.49	11.9
99	0	0	1	2	7.4	5.4	13.6
100	0	0	0	2	10.6	4.33	8.9
101	0	0	1	2	2.9	4.64	11.7
102	0	0	1	2	7	4.56	12.1
103	0	0	1	2	6.2	4.29	11.8
104	0	0	1	2	2.5	3.05	7.6
105	0	0	1	2	13.4	4.49	12.8
106	0	0	1	4	7.1	4.88	13
107	0	0	1	2	6.4	4.89	13.2
108	0	0	1	2	18.6	4.92	13.1
109	0	0	1	3	7.9	4.71	12.1
110	0	0	1	3	5.3	4.9	12.9
111	0	0	0	2	20.4	4.23	11.5
112	0	0	0	3	6.2	4.09	12.5
113	0	0	0	3	16.8	4.57	12.7
114	0	0	1	2	11.6	4.76	12.2
115	0	0	1	2	22.6	4.68	12
116	0	0	0	2	2.3	4.49	12.4
117	0	0	1	3	10.5	4.8	13.2
118	0	0	0	2	8.2	4.39	12
119	0	0	1	3	13.8	4.9	13.4
120	0	0	1	3	6.4	5.12	14.3
121	0	0	0	3	9.6	4.5	12.1
122	0	0	1	4	9.5	4.31	12.3
123	0	0	1	3	4.9	4.7	12.5
124	0	0	0	2	11.9	4.44	12.1
125	0	0	1	1	5.2	4.81	12.4
126	0	0	0	2	2.2	4.53	12.8
127	0	0	0	3	22.1	4.69	12.3
128	0	0	0	2	8.2	4.43	11.7
129	0	0	0	2	20.3	4.65	12.5
130	0	0	1	2	18.2	4.33	12
131	0	0	1	4	2.6	3.13	9.5
132	0	0	1	3	21.4	4.77	13
133	0	0	0	1	3.5	4.54	12
134	0	0	0	1	20.3	4.37	11.7
135	0	0	1	4	18.4	4.31	12.1
136	0	0	0	1	6.1	5.09	13.6
137	0	0	0	4	9.4	4.68	13.5

138	0	0	0	1	8.7	4.79	12.6
139	0	0	1	1	4	4.73	12.9
140	0	0	1	1	8	4.63	12.9
141	0	0	0	1	8.1	4.65	13
142	0	0	0	3	4.5	5.12	13.5
143	0	0	1	1	5.3	4.62	13.5
144	0	0	1	4	8.9	4.88	13.2
145	0	0	0	1	2.6	4.71	12.8
146	0	0	0	2	22.4	5.11	12.2
147	0	0	1	2	5.8	4.62	12.3
148	0	0	1	2	8.1	5.01	13.1
149	0	0	0	1	6.6	4.56	12.1
150	0	0	1	2	7.2	5.16	12.8
151	0	0	1	4	9.9	4.77	13.2
152	0	0	1	1	4.5	4.39	11.3
153	0	0	0	1	15.5	4.09	11.8
154	0	0	0	2	10.8	3.36	9.1
155	0	0	1	1	11.3	4.5	11.9
156	0	0	1	1	5.9	4.77	12
157	0	0	0	1	5.1	3.92	11.2
158	0	0	0	2	10.1	4.35	12.7
159	0	0	1	3	4.9	4.87	11.8
160	0	0	0	3	7.6	5.16	14.1
161	0	0	1	1	8.6	4.78	12.4
162	0	0	0	1	14.1	4.2	11.8
163	0	0	0	2	7.8	4.43	12
164	0	0	0	1	16.8	4.99	13.5
165	0	0	1	1	5.7	4.64	12.5
166	0	0	0	1	17.1	4.48	11
167	0	0	1	1	18.5	4.54	11.8
168	0	0	1	4	21.2	4.39	12.2
169	0	0	0	1	18.6	4.13	11.3
170	0	0	1	1	17.9	4.45	11.8
171	0	0	1	2	7.7	4.92	13.3
172	0	0	1	2	6.5	4.76	12.6
173	0	0	0	1	8.5	5.04	13.2
174	0	0	0	1	6.1	4.26	11.9
175	0	0	1	4	9.6	4.03	11.6
176	0	0	0	2	13	4.56	12.8
177	0	0	0	1	3.6	4.6	11.8
178	0	0	0	1	8.3	4.96	13.5
179	0	0	1	4	20.3	4.04	12
180	0	0	0	4	11.5	4.47	11.9
181	0	0	1	1	7.7	4.43	12.3
182	0	0	0	1	9.4	3.67	9.8
183	0	0	1	2	6.4	4.03	11.4

184	0	0	1	4	14.5	5.17	14.3
185	0	0	0	4	22.4	4.09	10.7
186	0	0	1	1	8.4	5.29	13.3
187	0	0	0	4	6.5	4.28	11.6
188	0	0	0	1	12	4.94	12.9
189	0	0	1	1	14.1	4.84	13.3
190	0	0	1	2	23.3	4.71	13
191	0	0	0	2	14.2	4.2	10.9
192	0	0	1	1	16.6	4.69	13.3
193	0	0	1	3	16.1	3.95	10.3
194	0	0	0	2	15.6	4.68	12.3
195	0	0	1	1	15.3	5.02	12.9
196	0	0	1	4	9.8	4.16	11
197	0	0	1	3	16.3	5.04	12.9
198	0	0	1	4	6.5	4.44	11.8
199	0	0	1	1	8.6	5.15	13.1
200	0	0	0	1	7	4	11.5
201	0	0	0	1	4.1	4.68	13
202	0	0	1	1	5.9	4.44	12.4
203	0	0	1	1	10	4.67	12.1
204	0	0	1	1	9.9	4.63	11.9
205	0	0	1	1	10.3	4.74	10.4
206	0	0	1	1	10.3	4.89	13.5
207	0	0	1	1	9.3	4.43	12.3
208	0	0	1	1	8.2	4.55	12
209	0	0	1	1	8.4	3.85	10.7
210	0	0	1	1	7.9	4.84	12.6
211	0	0	0	1	9.3	4.27	11.7
212	0	0	1	1	17.2	4.67	10
213	0	0	1	2	9.3	4.76	12.4
214	0	0	1	1	9.9	4.19	11.6
215	0	0	1	4	19.8	4.54	12
216	0	0	0	3	9.7	4.59	12.6
217	0	0	0	4	7.3	4.56	11.5
218	0	0	0	1	24.2	3.9	11.1
219	0	0	0	2	6.5	3.83	11.2
220	0	0	1	1	7.4	5.07	13.9
221	0	0	1	2	19.7	4.31	11
222	0	0	1	2	15.5	4.7	12.7
223	0	0	0	2	6.3	4.81	13.1
224	0	0	1	4	5.6	4.48	12
225	0	0	1	0	6.3	4.46	12.3
226	0	0	1	0	17.3	3.78	11.6
227	0	0	1	1	4.2	4.91	13.3
228	0	0	0	1	4.2	4.8	13.8
229	0	0	1	4	14.5	4.97	13.3

230	0	0	1	0	5	4.62	12.2
231	0	0	1	0	3.7	4.4	12
232	0	0	0	0	9.4	4.55	15.3
233	0	0	1	4	4.5	4.68	12.1
234	0	0	0	1	4.8	4.69	11.3
235	0	0	0	4	4.9	4.33	12.1
236	0	0	0	2	14.1	3.99	11.2
237	0	0	0	4	8.3	4.5	12.1
238	0	0	1	4	8.8	4.62	12.9
239	0	0	0	0	16.9	4.16	12
240	0	0	0	1	21.2	4.47	11.1
241	0	0	0	3	2.5	5.34	12.9
242	0	0	1	1	6.9	4.24	11.6
243	0	0	1	2	7.3	4.69	12.1
244	0	0	0	1	3.7	4.47	11.8
245	0	0	0	1	8.6	4.91	12.6
246	0	0	0	1	3.6	4.53	12.1
247	0	0	0	1	9.3	3.37	10.2
248	0	0	1	1	11	4.83	13.2
249	0	0	0	4	15.2	3.77	10.7
250	0	0	1	1	10	3.45	10.1
251	0	0	0	2	18.1	4.57	12.5
252	0	0	1	2	20.1	5.14	12.9
253	0	0	1	4	18.1	5.3	13.2
254	0	0	0	0	9.9	4.44	11.5
255	0	0	0	4	18.5	4.85	13.6
256	0	0	0	1	6.5	4.36	12.6
257	0	0	0	4	8.2	4.52	12.2
258	0	0	0	0	21.1	4.08	12
259	0	0	1	4	6.6	4.95	13.4
260	0	0	0	2	6	4.42	12.4
261	0	0	1	3	10.2	4.22	11.7
262	0	0	0	1	8.8	5.02	11.7
263	0	0	1	1	12.1	4.66	12.2
264	0	0	1	1	16.9	5.27	13.6
265	0	0	1	1	13.2	4.81	13.2
266	0	0	1	4	8.6	4.26	11.1
267	0	0	1	2	23.5	5.73	15.5
268	0	0	1	0	13.7	4.94	11.2
269	0	0	1	4	13	4.44	12.1
270	0	0	0	0	14.7	4.47	11.6
271	0	0	1	4	14.8	5.12	13.6
272	0	0	0	1	12.2	5.3	12.7
273	0	0	1	2	7.5	4.38	10.5
274	0	0	1	4	6.5	4.84	13.4
275	0	0	1	4	4	4.31	12.2

276	0	0	1	1	26.1	5.32	13
277	0	0	1	4	10.2	4.56	13.5
278	0	0	1	4	8.2	5.09	14.2
279	0	0	1	4	5.4	4.93	13.2
280	0	0	1	0	11.5	4.72	12.6
281	0	0	1	0	23.2	4.46	11.9
282	0	0	1	4	7.5	4.79	12.9
283	0	0	0	4	6.4	4.28	11.3
284	0	0	0	4	7.8	4.27	11.7
285	0	0	1	4	13.1	4.8	12.7
286	0	0	1	4	12.5	4.75	13.5
287	0	0	0	1	5.5	4.71	13.1
288	0	0	1	0	23.8	5.56	15.1
289	0	0	1	4	5.4	4.65	11.5
290	0	0	0	4	8.3	4.03	11.9
291	0	0	1	4	5.5	4.29	12.2
292	0	0	0	4	9.1	4.77	12.9
293	0	0	0	2	3.9	4.84	12.7
294	0	0	1	0	8.3	4.55	10.9
295	0	0	0	4	20.4	5.02	13.7
296	0	0	0	1	19	4.83	12.7
297	0	0	1	3	6.3	4.83	12.8
298	0	0	1	2	4.4	4.28	11.1
299	0	0	1	0	7.7	4.92	16.6
300	0	0	0	1	8.8	4.83	11.8
301	0	0	1	4	21.5	4.6	12.9
302	0	0	1	4	4.6	4.15	10.6
303	0	0	1	0	4.6	4.23	11.6
304	0	0	1	2	8.1	4.71	12
305	0	0	0	4	10.7	4.6	12
306	0	0	1	4	16.4	4.47	12.2
307	0	0	0	0	22.3	4.41	14.7
308	0	0	1	0	13.7	3.26	10
309	0	0	0	0	16.7	4.04	11
310	0	0	1	4	4.7	4.88	13.4
311	0	0	1	3	8.5	4.78	12.6
312	0	0	0	4	7	4.65	13.1
313	0	0	1	4	7.7	4.63	11.7
314	0	0	1	4	14.9	4.17	11.3
315	0	0	0	4	36	4.6	13
316	0	0	1	3	16.6	4.9	13.5
317	0	0	0	1	16.5	4.79	12.8
318	0	0	0	1	6.4	4.54	11.4
319	0	0	1	4	17.4	4.47	12.2
320	0	0	1	0	16	3.41	10.4
321	0	0	1	3	13.1	4.67	13

322	0	0	1	2	14.3	3.99	11.3
323	0	0	1	4	13.8	4.96	13.1
324	0	0	0	0	13.5	3.19	9.1
325	0	0	0	1	22.5	4.13	11.7
326	0	0	0	4	6.7	4.18	11.7
327	0	0	0	4	6.3	4.35	12.4
328	0	0	1	0	5.5	4.82	12.7
329	0	0	0	4	4.4	5.02	13.3
330	0	0	1	4	17.2	4.38	11.7
331	0	0	1	4	8.5	4.49	12.7
332	0	0	0	1	7.7	4.58	11.8
333	0	0	0	1	20.9	4.02	10.3
334	0	0	1	1	5.3	4.33	11.2
335	0	0	0	4	13.2	3.69	10.4
336	0	0	0	1	4.2	3.89	10.6
337	0	0	0	0	10	4.27	14.9
338	0	0	1	1	13.6	4.71	11.9
339	0	0	1	3	11.2	4.51	12
340	0	0	0	4	7.8	4.62	13
341	0	0	0	4	25.1	4.5	13.1
342	0	0	1	1	14.5	4.91	13.2
343	0	0	1	4	10.6	4.63	11.9
344	0	0	0	2	8.3	4.49	12
345	0	0	1	4	9.5	4.62	13.6
346	0	0	0	3	10.1	4.45	11.3
347	0	0	1	1	8.3	4.06	11.4
348	0	0	0	1	6.9	4.63	12.7
349	0	0	1	0	1.8	2.84	9.1
350	0	0	1	4	7.5	4.93	13.1
351	0	0	0	4	4.9	4.48	12.7
352	0	0	1	4	2.5	4.33	12.3
353	0	0	1	4	22.9	5.1	13.5
354	0	0	1	4	8.3	4.11	11.4
355	0	0	1	1	8.9	4.73	11.6
356	0	0	1	1	24.7	4.52	11.3
357	0	0	1	1	8.9	4.93	11.6
358	0	0	0	4	5.3	4.44	12.1
359	0	0	1	1	18	4.02	10.1
360	0	0	1	1	3.7	4.54	11
361	0	0	0	4	5.6	4.45	12.3
362	0	0	0	1	33.4	4.17	11.5
364	0	0	0	4	29.8	4.23	11.9
365	0	0	1	1	11.5	3.75	9.5
366	0	0	0	1	5.6	4.57	12.2
367	0	0	1	0	10.4	4.34	15.7
368	0	0	0	1	20.1	4.51	11.4

369	0	0	1	4	5.7	4.61	12.7
370	0	0	1	3	8.5	4.63	12.4
371	0	0	0	2	15.5	4	11.2
372	0	0	1	2	8.7	5.38	13.7
373	0	0	1	0	10.4	3.24	10.3
374	0	0	0	4	5.3	4.48	12.5
375	0	0	1	3	9	4.08	11.1
376	0	0	0	0	15.2	4.06	10.8
377	0	0	1	3	6	4.12	11
378	0	0	0	0	13.6	4.31	14.8
379	0	0	0	3	15.5	4.29	11.6
380	0	0	0	4	16	4.21	10.9
381	0	0	0	4	9.7	4	11.3
382	0	0	1	4	21.8	4.51	12.2
383	0	0	1	1	7.2	3.6	11.3
384	0	0	0	3	10.9	4.68	12.5
385	0	0	0	1	15.6	3.5	10.5
386	0	0	1	1	13.2	4	11
387	0	0	0	4	19.3	4.63	13.3
388	0	0	1	4	6.2	4.73	12.8
389	0	0	1	0	6.1	3.98	10.8
390	0	0	0	1	14.7	4.65	12.1
391	0	0	0	3	10.5	5.07	13.1
392	0	0	1	4	10.4	4.93	14.2
393	0	0	1	1	11.1	4.73	12.5
394	0	0	1	3	9	4.32	12.5
395	0	0	0	3	14.9	4.38	12.4
396	0	0	1	1	15.1	5.07	12.5
397	0	0	0	0	4.9	3.84	10.8
398	0	0	0	3	8.7	4.24	11.5
399	0	0	0	1	15.1	4.83	13.6
400	0	0	0	3	2.9	4.8	13.5
401	0	0	1	3	6.3	4.09	11.7
402	0	0	1	4	16.6	4.98	14.1
403	0	0	1	2	12	5.02	13.1
404	0	0	1	1	25.4	4.92	11.9
405	0	0	0	4	6.3	4.27	12.2
406	0	0	1	1	20.6	4.55	12.3
407	0	0	1	1	16.3	4.31	11.5
408	0	0	0	2	8.6	4.35	12.4
409	0	0	1	0	9.2	4.06	13.2
410	0	0	1	4	11.9	4.03	11.7
411	0	0	0	2	16.9	4.2	11.3
412	0	0	0	2	19.7	4.92	12.7
413	0	0	1	3	13.1	4.57	9.9
414	0	0	0	1	5.2	4.1	10.6

415	0	0	1	3	5.5	4.11	11.3
416	0	0	1	2	27	5.4	15.3
417	0	0	0	0	11.1	4.82	12.3
418	0	0	0	2	9.9	4.71	12.3
419	0	0	0	3	9.8	3.88	12.1
420	0	0	0	0	6.3	3.1	9.6
421	0	0	1	0	16.6	4.94	11.8
422	0	0	1	2	2.9	4	10.7
423	0	0	0	3	11.7	4.43	12.3
424	0	0	1	0	11.5	3.35	10
425	0	0	1	2	18.2	4.28	12.1
426	0	0	1	4	20.1	5.24	14.1
427	0	0	0	1	8.3	4.08	10.9
428	0	0	0	2	5.3	4.71	12.1
429	0	0	0	4	16.5	4.46	12.3
430	0	0	0	4	6	4.68	13
431	0	0	1	3	12.2	4.87	13.4
432	0	0	1	2	11.4	4.91	13.3
433	0	0	1	4	11.9	4.47	11.2
434	0	0	1	3	9.3	4.8	12.3
435	0	0	0	0	15.8	3.48	9.3
436	0	0	1	4	11.9	4.48	11.7
437	0	0	0	3	8.5	4.83	13.3
438	0	0	0	1	8.1	4.58	12.3
439	0	0	1	4	5.8	5.45	13.4
440	0	0	1	3	10.1	4.52	12
441	0	0	1	3	7.2	4.4	12.2
442	0	0	1	3	7.2	4.12	11.2
443	0	0	1	4	10.6	4.93	13.1
444	0	0	1	2	10.2	4.73	13.2
445	0	0	1	2	12	5.16	13.1
446	0	0	1	0	12.5	3.84	11
447	0	0	1	4	11.1	4.57	12.7
448	0	0	0	3	9.4	4.52	12.2
449	0	0	0	4	10.5	4.14	11.3
450	0	0	1	0	13.6	3.53	10.3
451	0	0	0	1	22.6	4.72	13.1
452	0	0	1	1	14.3	5.16	13.3
453	0	0	1	3	9.9	4.45	12
454	0	0	0	3	4.8	4.4	11.9
455	0	0	0	0	13.3	4.25	12.1
456	0	0	0	3	11	3.98	10.6
457	0	0	1	3	13.3	4.32	12.1
458	0	0	0	4	2.6	4.17	11.6
459	0	0	0	0	13.2	3.21	9.9
460	0	0	1	3	5.2	4.6	12.7

461	0	0	1	3	7.8	4.24	11.3
462	0	0	0	1	11.6	4.31	11.4
463	0	0	1	3	18.8	4.55	13.1
464	0	0	0	4	18.2	3.95	10.7
465	0	0	1	3	11.7	4.43	12.8
466	0	0	0	1	26.7	3.98	9.9
467	0	0	1	3	16.7	4.29	12.3
468	0	0	1	4	24.3	4.59	13
469	0	0	0	4	18	4.67	12.4
470	0	0	1	3	7.8	4.16	11.4
471	0	0	0	0	15.5	3.64	10.1
472	0	0	1	0	7.1	3.54	10.1
473	0	0	0	1	20	4.87	12.6
474	0	0	0	4	27.4	4.82	11.9
475	0	0	0	3	8.9	4.05	11
476	0	0	0	3	14.3	4.29	11.7
477	0	0	0	2	14.3	4.58	11.6
478	0	0	0	4	7.5	4.57	12.6
479	0	0	1	2	20.5	4.26	11.6
480	0	0	1	3	8.4	4.19	11.5
481	0	0	0	4	3	4.26	12.3
482	0	0	1	3	15.9	4.37	11.6
483	0	0	0	0	8.1	3.51	10.3
484	0	0	0	3	2.1	4.71	12.6
485	0	0	0	0	7.1	3.23	11
486	0	0	0	3	18	3.85	10.3
487	0	0	0	2	10.7	4.75	13.5
488	0	0	0	3	13.2	4.94	13.7
489	0	0	1	1	7.1	4.91	12.7
490	0	0	0	4	15.6	4.34	11.6
491	0	0	1	3	11.1	4.46	13.4
492	0	0	1	0	19.2	3.82	12.5
493	0	0	1	4	5.5	4.38	12.7
494	0	0	0	4	17	4.81	13
495	0	0	1	2	13.2	4.79	13.7
496	0	0	1	1	17.1	4.89	12.8
497	0	0	0	3	23.4	5.18	13.7
498	0	0	0	4	15	4.56	12.4
499	0	0	0	1	11.3	4.19	11.2
500	0	0	1	4	6	4.19	12.2
501	0	0	0	3	9.1	4.22	11.7
502	0	0	1	4	10.7	4.95	12.5
503	0	0	0	3	7.5	4.7	13.2
504	0	0	0	2	5.7	4.57	12.6
505	0	0	0	0	3.8	3.45	10
506	0	0	1	4	7	4.79	12.6

507	0	0	0	3	3.5	4.59	13
508	0	0	0	3	6.2	4.73	12.1
509	0	0	0	4	4.7	4.72	12.8
510	0	0	0	1	25.9	4.83	13.4
511	0	0	0	3	4.2	5.41	12.7
512	0	0	1	2	10.7	4.22	10.8
513	0	0	0	3	6.1	4.54	13.1
514	0	0	1	3	8.2	4.45	12.7
515	0	0	0	3	9.4	5.09	14.6
516	0	0	0	1	24.7	4.07	11.2
517	0	0	1	3	3.5	4.25	12.1
518	0	0	1	0	15.8	3.62	11.5
519	0	0	0	3	13.4	4.48	12.6
520	0	0	1	1	9.1	4.1	11.5
521	0	0	1	4	9.7	4.69	12.7
522	0	0	1	3	4.2	4.35	11.7
523	0	0	1	1	22	5.12	12.8
524	0	0	0	3	14.3	3.95	10.5
525	0	0	1	4	29.2	4.17	10.5
526	0	0	0	3	6.4	4.72	13
527	0	0	0	3	8.6	5.01	13.9
528	0	0	0	4	13.9	4.5	11.7
529	0	0	0	3	26.4	4.35	12.3
530	0	0	0	4	1	4.69	13.5
531	0	0	1	3	20	4.88	13.2
532	0	0	0	1	10.9	3.92	10.6
533	0	0	0	2	22.3	4.9	12.9
534	0	0	1	3	7.2	4.19	10.9
535	0	0	0	2	13.7	4.52	12
536	0	0	0	4	12.5	3.55	10.7
537	0	0	1	1	15.7	4.54	9.9
538	0	0	1	0	9.9	3.39	10.2
539	0	0	1	4	15.8	4.6	12.8
540	0	0	1	4	13.9	4.83	13.1
541	0	0	1	1	14.2	4.16	12
542	0	0	0	4	6.1	4.75	13.9
543	0	0	1	3	18.9	4.93	12.2
544	0	0	1	4	8.3	4.69	12.9
545	0	0	0	4	11.4	4.31	13.2
546	0	0	1	4	10.1	4.39	12.2
547	0	0	1	3	5.8	4.54	12.3
548	0	0	0	1	17.9	3.87	10.4
549	0	0	1	3	5.3	4.08	10.9
550	0	0	1	3	16.6	4.54	12.6
551	0	0	0	2	14	4.5	11.5
552	0	0	1	3	12.2	4.14	11.8

553	0	0	1	0	11.2	3.87	12.9
554	0	0	0	1	14.2	5.05	14
555	0	0	1	4	22.6	5.02	13.2
556	0	0	0	2	4.2	4.25	10.8
557	0	0	1	2	6.9	4.22	11.4
558	0	0	1	4	9.1	4.81	13.7
559	0	0	0	4	9.5	4.51	11.9
560	0	0	0	2	13.7	4.06	10.9
561	0	0	1	2	14.2	4.85	11.8
562	0	0	1	2	6.4	4.68	12.2
563	0	0	1	3	14.4	4.52	13.1
564	0	0	0	2	11.8	4.82	12.5
565	0	0	1	3	4	4.32	11.9
566	0	0	0	4	16.3	5.28	13.8
567	0	0	0	3	3.6	4.4	12.5
568	0	0	1	2	19.1	4.83	13.2
569	0	0	0	3	10.4	4.73	12.3
570	0	0	0	3	11.6	4.52	11.4
571	0	0	1	4	9.4	4.48	12.4
572	0	0	1	0	2.7	4.58	12.7
573	0	0	0	2	5.4	4.82	13
574	0	0	1	3	6.7	4.79	13
575	0	0	1	0	4.6	4.59	12.5
576	0	0	0	2	3.5	4.37	11.7
577	0	0	1	2	5.4	4.5	12.5
578	0	0	1	4	13.4	4.81	12.5
579	0	0	1	0	16.5	4.72	12.6
580	0	0	0	0	10.8	5.46	18.4
581	0	0	1	3	9.4	4.86	13.1
582	0	0	1	3	8.3	4.8	13.2
583	0	0	0	3	18.3	4.82	13
584	0	0	1	3	23.7	4.54	12.9
585	0	0	1	1	5.3	4.87	13.4
586	0	0	1	3	12.2	5.02	14.2
587	0	0	0	3	4.8	4.49	12.5
588	0	0	1	3	7.8	5.02	14
589	0	0	1	3	8.5	4.26	12.4
590	0	0	0	1	17.6	4.3	11.5
591	0	0	1	0	18	4.79	12.3
592	0	0	1	2	9.3	4.22	11.7
593	0	0	0	1	9.1	4.36	11.3
594	0	0	1	2	11	5.26	12.1
595	0	0	0	1	6.5	4.26	11.4
596	0	0	1	3	8	4.61	12.3
597	0	0	0	3	9.8	4.23	12
598	0	0	1	2	18.8	4.28	11.9

599	0	0	0	3	5.1	3.8	11.2
600	0	0	1	2	6.1	4.65	13.1
601	0	0	0	2	16.7	4.75	12.7
602	0	0	1	2	10.3	4.01	11.2
603	0	0	0	2	5.4	4.07	10.9
604	0	0	0	4	10.4	3.83	10.3
605	0	0	0	3	27.5	4.37	11.7
606	0	0	1	3	4.6	4.77	13.3
607	0	0	0	0	5.2	3.6	10.4
608	0	0	0	0	10.9	3.79	10.2
609	0	0	0	2	11.1	4.06	11.4
610	0	0	0	1	4.5	4.94	13
611	0	0	0	2	5.7	4.76	12.5
612	0	0	0	1	15.4	4.37	11.1
613	0	0	0	1	13.9	4.83	12.4
614	0	0	0	3	6.3	4.52	12.4
615	0	0	1	2	12.6	4.33	12.3
616	0	0	0	2	11.2	4.41	11.7
617	0	0	0	3	6.4	4.63	12.9
618	0	0	1	2	7.6	4.74	13
619	0	0	0	2	11.6	4.74	12
620	0	0	1	3	6.8	4.32	12
621	0	0	0	2	6.4	4.72	12.8
622	0	0	0	1	25.4	4.13	11.9
623	0	0	1	3	10	4.42	11.1
624	0	0	1	2	6	4.75	14.1
625	0	0	1	3	8.5	3.76	10.7
626	0	0	0	0	5.4	4.09	11
627	0	0	1	2	4.9	4.75	12.5
628	0	0	1	0	14.5	4.53	11.3
629	0	0	1	2	23.1	4.86	12.6
630	0	0	1	2	3.7	5.02	13.3
631	0	0	1	2	7.6	3.98	11.3
632	0	0	1	2	7.8	4.33	12.3
633	0	0	1	4	8.1	4.23	11.8
634	0	0	1	2	10.3	4.36	11.5
635	0	0	0	4	11.6	4.31	12.3
636	0	0	0	1	14.4	4.76	12.7
637	0	0	0	2	10.6	4.45	11.9
638	0	0	1	0	10.7	3.55	10.7
639	0	0	1	2	4.8	4.46	12.1
640	0	0	0	4	7.3	4.69	13.3
641	0	0	1	2	10	4.58	11.1
642	0	0	0	1	14.6	4.25	11.4
643	0	0	1	3	15.4	4.54	11.7
644	0	0	0	2	9.4	4.72	13.5

645	0	0	1	2	6.3	5.24	13.6
646	0	0	1	3	7.1	4.11	10.7
647	0	0	0	2	12.1	4.55	12.5
648	0	0	0	3	15.9	4.78	13.4
649	0	0	1	0	9.2	4.48	13.7
650	0	0	1	3	13.1	5.1	13.6
651	0	0	0	1	10.2	4.61	11
652	0	0	1	2	9.6	4.92	13
653	0	0	0	2	5.8	4.41	11.9
654	0	0	1	2	4.8	4.6	12.2
655	0	0	0	2	7.4	4.11	10.9
656	0	0	1	3	14.3	4.33	12
657	0	0	1	1	7.9	4.86	12.4
658	0	0	1	2	3.2	4.87	12.4
659	0	0	1	1	10.2	4.47	10.3
660	0	0	1	2	4.1	4.33	12
661	0	0	1	3	17.8	4.97	12.8
662	0	0	1	4	16.8	4.62	12.4
663	0	0	0	1	16.8	4.67	10.8
664	0	0	1	1	39.1	5.21	11.5
665	0	0	1	2	10.3	4.64	12.6
666	0	0	0	1	12.9	4.19	10.3
667	0	0	1	2	11.6	4.54	12.2
668	0	0	0	4	10.9	4.14	11.8
669	0	0	1	2	8.4	4.29	11.2
670	0	0	1	0	6.2	3.98	11.2
671	0	0	1	4	10.3	5.1	12.6
672	0	0	0	2	5.6	4.24	11.3
673	0	0	1	4	8.8	4.61	12.4
674	0	0	1	1	10.6	4.53	11.6
675	0	0	0	4	4.3	4.97	12.7
676	0	0	0	2	6	4.57	11.7
677	0	0	1	1	11.2	4.84	13
678	0	0	0	2	5.7	4.48	11.6
679	0	0	0	2	6.2	4.92	13.1
680	0	0	0	2	11.8	4.11	12.1
681	0	0	0	2	14.3	4.88	13.3
682	0	0	1	3	9.8	4.53	12.5
683	0	0	1	2	5	4.71	12.6
684	0	0	1	1	14.8	4.85	12.4
685	0	0	1	2	14.8	4.65	12.6
686	0	0	1	2	12.3	4.72	11.9
687	0	0	1	1	14.5	4.31	11.4
688	0	0	1	2	6	4.9	13.8
689	0	0	1	1	15.7	4.07	11.9
690	0	0	0	2	11.8	4.69	12.5

691	0	0	0	2	5.6	4.35	12.1
692	0	0	1	2	4.5	3.64	10.3
693	0	0	1	2	9.3	4.62	10.4
694	0	0	0	2	12.2	4.49	12.5
695	0	0	0	2	6.9	3.82	10.8
696	0	0	1	3	4.5	4.54	12.6
697	0	0	0	2	6.7	4.26	11.4
698	0	0	0	2	6.2	4.86	13.2
699	0	0	0	1	17.2	4.41	9.9
700	0	0	1	0	8	4.26	11
701	0	0	0	0	4.6	4.63	14.2
702	0	0	1	0	4.5	4.11	12
703	0	0	0	0	5.3	3.95	11.2
704	0	0	0	1	7.1	4.51	12.4
705	0	0	1	1	16.4	5.11	12.4
706	0	0	1	0	10.6	4.66	16.2
707	0	0	1	1	6.8	3.82	10.9
708	0	0	1	1	9.7	4.08	11
709	0	0	1	1	16.9	4.15	13.8
710	0	0	0	3	8.9	4.55	12.8
711	0	0	1	4	13.9	4.01	11.6
712	0	0	0	2	15.9	4.64	12.8
713	0	0	0	1	19.4	4.05	10.9
714	0	0	0	1	3.5	4.83	13.2
715	0	0	1	1	3.4	4.39	11.8
716	0	0	0	0	16.9	4.73	12.1
717	0	0	1	0	14.9	4.19	14.6
718	0	0	1	0	10.3	3.97	9.7
719	0	0	0	1	8.1	4.79	12.2
720	0	0	1	0	5.3	5.13	13.3
721	0	0	0	2	15.5	4.86	14
722	0	0	1	1	6	4.04	11.2
723	0	0	1	1	5.8	5.11	13.2
724	0	0	0	0	26.6	3.35	11.8
725	0	0	0	1	16.6	4.81	12.7
726	0	0	1	0	6.7	4.71	12.6
727	0	0	1	0	12	4.19	10.3
728	0	0	1	1	7.8	4.78	12.6
729	0	0	0	1	6.4	4.55	12.2
730	0	0	0	1	7	4.96	12.5
731	0	0	1	1	3.9	4.1	8.1
732	0	0	1	1	8.1	4.16	10.8
733	0	0	1	1	7.2	4.63	12.3
734	0	0	1	0	6	5.33	12.1
735	0	0	0	1	11.4	4.36	11.8
736	0	0	0	1	6	4.69	12.4

737	0	0	1	1	12.9	4.15	10.8
738	0	0	0	1	10.2	4.46	11.8
739	0	0	0	1	8.3	4.73	12.6
740	0	0	1	2	7	5.04	14
741	0	0	1	1	15	4.08	11.5
742	0	0	1	0	17.4	3.16	10.6
743	0	0	1	0	17.4	4.09	11.2
744	0	0	0	0	6.1	4.2	11.6
745	0	0	1	2	9	4.07	11
746	0	0	1	1	9.2	4.45	11.8
747	0	0	0	0	7.9	3.68	11.4
748	0	0	0	1	8.2	4.39	10.9
749	0	0	0	0	12.5	2.58	8.7
750	0	0	0	0	13.4	4.75	16.3
751	0	0	0	2	4.1	4.24	11.8
752	0	0	0	1	12.8	3.5	8.2
753	0	0	1	0	13.3	4.3	11.6
754	0	0	0	0	10.2	3.53	9.4
755	0	0	1	4	5.3	4.96	12.4
756	0	0	1	2	10.3	5.32	14.4
757	0	0	1	1	10.6	4.69	12.1
758	0	0	1	1	8.1	4.56	11.3
759	0	0	1	4	3.2	4.16	11.9
760	0	0	1	1	1.8	4.76	11.5
761	0	0	0	1	7	4.44	11.8
762	0	0	1	1	6.4	4.75	13.2
763	0	0	1	1	9.1	4.39	11.7
764	0	0	1	4	8	5.25	12.8
765	0	0	1	1	8.1	4.39	11.4
766	0	0	0	1	11.9	4.45	10.7
767	0	0	1	1	7.8	4.39	11.8
768	0	0	1	1	10.3	4.6	11.8
769	0	0	1	1	10.3	4.72	12.3
770	0	0	0	1	5.3	4.11	11.2
771	0	0	1	4	12.6	4.17	11.9
772	0	0	0	1	11.8	4.17	11.1
773	0	0	1	4	16.6	5.05	14.5
774	0	0	0	2	16.3	4.56	12.4
775	0	0	0	4	5.8	4.33	11.5
776	0	0	0	1	12.3	4.42	11.8
777	0	0	0	2	4.1	4.42	12.1
778	0	0	1	1	10	4.78	11.7
779	0	0	0	2	26.7	4.48	10.6
780	0	0	1	3	16.2	4.48	12
781	0	0	1	2	18.6	4.6	12.7
782	0	0	1	3	8.3	4.85	12.7

783	0	0	1	2	7.8	4.52	11.5
784	0	0	1	2	6.2	4.33	11.7
785	0	0	0	3	7.3	4.49	13
786	0	0	1	3	17.6	4.92	13.4
787	0	0	1	2	20.7	4.31	10.9
788	0	0	0	3	9.1	4.45	12.4
789	0	0	0	1	11.8	3.82	10.9
790	0	0	0	2	16	4.39	11.9
791	0	0	1	2	10.4	4.65	12.5
792	0	0	0	2	8.4	4.29	11.4
793	0	0	0	1	8.6	4.24	11.5
794	0	0	0	1	5.7	3.51	10.2
795	0	0	0	2	12.8	4.69	11.9
796	0	0	1	1	8.6	4.52	12.4
797	0	0	0	0	13.3	3.61	11.6
798	0	0	1	0	4.7	3.41	9.7
799	0	0	0	1	7.8	5.09	13.4
800	0	0	1	0	4.2	4.34	11.1
801	0	0	0	3	9.1	4.12	11.7
802	0	0	1	1	8	4.63	12.3
803	0	0	0	0	22.7	3.61	11
804	0	0	1	4	8.9	5.08	13.4
805	0	0	1	1	6.9	4.57	11.5
806	0	0	1	1	11.3	4.2	10.7
807	0	0	0	1	13.2	4.66	12.3
808	0	0	1	1	8.6	4.36	9.6
809	0	0	0	1	19.7	4.28	11.5
810	0	0	1	0	8	3.38	10.1
811	0	0	1	0	15.9	4.56	15
812	0	0	1	1	10.2	4.41	11.9
813	0	0	1	1	12.9	4.64	11.3
814	0	0	1	1	12.3	3.89	9.5
815	0	0	1	0	12.5	4.27	11.3
816	0	0	1	2	9.1	5.23	12.9
817	0	0	1	0	12.1	5.01	18.7
818	0	0	0	0	16.8	4.38	11.6
819	0	0	0	1	8.2	5.24	12.1
820	0	0	1	0	25.2	3.9	11
821	0	0	1	4	8.5	4.53	12.1
822	0	0	1	1	4.5	4.64	11.1
823	0	0	1	0	8.7	4.06	13.9
824	0	0	0	1	2.4	3.83	10.2
825	0	0	0	2	3.2	3.76	11
826	0	0	0	1	10.6	4.23	11.2
827	0	0	1	0	12.5	3.16	9.5
828	0	0	0	1	4.6	4.45	11.8

829	0	0	0	1	10.7	4.32	11.4
830	0	0	1	1	18.3	4.62	13
831	0	0	0	1	19.1	5.07	13.2
832	0	0	1	0	27.5	4.24	11.1
833	0	0	1	2	5.6	4.87	13.3
834	0	0	0	1	2.7	4.46	12.1
835	0	0	0	0	9.4	3.28	10.2
836	0	0	0	1	12.2	4.21	10.8
837	0	0	1	1	5.9	4.09	12.9
838	0	0	0	0	12.5	4.22	11.3
839	0	0	0	1	20.2	4.74	12.3
840	0	0	1	0	7.6	3.31	10.3
841	0	0	0	2	4.6	4.27	12.1
842	0	0	1	0	6.5	3.71	11.6
843	0	0	1	1	5.9	4.7	12.1
844	0	0	1	0	16.6	4.89	16.4
845	0	0	0	2	12.9	4.61	12.3
846	0	0	0	1	8.9	4.6	11.5
847	0	0	0	1	4.5	4.46	11.7
848	0	0	0	1	8.4	4.2	11.2
849	0	0	0	4	2.1	4.72	12.7
850	0	0	0	1	15.8	4.43	11.3
851	0	0	0	1	3.8	4.54	12.2
852	0	0	1	1	16.5	4.08	11
853	0	0	0	1	11.7	4.24	10.6
854	0	0	1	1	10.5	4.7	12
855	0	0	1	1	12.8	5.08	11.6
856	0	0	1	1	18.7	4.44	11.6
857	0	0	0	1	9.9	4.47	12.3
858	0	0	1	4	8.8	4.53	12.2
859	0	0	0	1	13.3	4.28	11.7
860	0	0	0	1	11.6	4.71	11.9
861	0	0	1	1	20.1	3.57	10.3
862	0	0	1	1	6.9	4.26	10.9
863	0	0	1	1	4.3	3.29	8.5
864	0	0	0	2	14.4	4.81	13.2
865	0	0	0	0	12.4	3.19	10.1
866	0	0	0	1	9.6	4.07	11.9
867	0	0	0	1	18.3	3.41	9.9
868	0	0	1	1	6.7	4.14	10.8
869	0	0	1	2	9.2	5.03	12.9
870	0	0	0	1	3.1	4.18	11
871	0	0	1	1	6.2	4.3	10.9
872	0	0	1	1	16.5	4.33	11.7
873	0	0	1	1	14.7	4.03	9.8
874	0	0	0	4	12.6	4.68	12.7

875	0	0	1	1	5.7	3.65	8.7
876	0	0	1	2	7.1	4.45	11
877	0	0	1	2	5.3	4.62	12.4
878	0	0	0	1	6.8	3.95	10.6
879	0	0	1	1	11.3	3.86	10.6
880	0	0	0	1	7.7	4.32	11.9
881	0	0	1	1	18.6	3.73	9.9
882	0	0	0	1	12.2	4.69	12.2
883	0	0	1	1	3.9	3.3	9.2
884	0	0	0	4	7.8	4.88	12.8
885	0	0	1	1	8.7	4.44	9.7
886	0	0	0	1	5.9	4.16	11
887	0	0	1	1	16	4.67	11.6
888	0	0	0	1	5	3.2	9.8
889	0	0	1	0	18.9	4.43	11.4
890	0	0	1	1	9.2	4.68	13
891	0	0	1	1	5.3	5.33	13.4
892	0	0	1	0	9.2	4.27	10.6
893	0	0	0	0	6.2	3.58	10.9
894	0	0	1	3	4.2	4.98	12.2
895	0	0	0	0	9.1	4.35	11.6
896	0	0	0	3	14.4	4.89	13.4
897	0	0	1	1	8.9	4.88	12.7
898	0	0	1	0	9.5	3.38	11
899	0	0	1	0	6.2	4.24	10.8
900	0	0	0	3	7.2	4.74	13
901	0	0	0	0	7.1	4.64	13.1
902	0	0	1	0	13.1	4.63	16.5
903	0	0	0	0	14.1	3.94	10.8
904	0	0	0	0	4.9	4.27	10.5
905	0	0	1	1	9	4.55	12.5
906	0	0	1	0	10.8	3.63	10.3
907	0	0	1	1	3.9	4.86	12.1
908	0	0	1	0	4.2	3.97	11.5
909	0	0	1	1	9.8	4.77	12.6
910	0	0	0	0	8.5	3.78	12.1
911	0	0	1	1	7.6	4.88	13.2
912	0	0	0	1	9.1	4.78	13.6
913	0	0	1	0	12.8	4.43	11
914	0	0	1	0	9.8	5.47	20.6
915	0	0	1	1	6.5	5.12	13
916	0	0	1	0	4.3	3.75	11.3
917	0	0	1	1	12.8	4.74	12.4
918	0	0	0	0	13.8	5.13	18.1
919	0	0	0	0	9.1	4.23	12
920	0	0	0	1	11.1	5.19	14.6

921	0	0	1	0	7.9	4.7	11.1
922	0	0	1	0	21.1	3.74	8.1
923	0	0	1	0	3.8	3.93	10
924	0	0	0	0	10.2	3.99	13
925	0	0	0	1	6.4	4.33	11.7
926	0	0	0	1	6.1	4.34	11.6
927	0	0	0	0	5.6	3.21	9.4
928	0	0	0	0	10.7	3.71	9.2
929	0	0	1	0	8.8	3.95	11.1
930	0	0	1	0	11.9	2.92	9.7
931	0	0	0	1	10.9	4.72	12.4
932	0	0	1	0	10.4	3.24	10
933	0	0	0	1	9.4	4.7	12.8
934	0	0	0	1	8.3	4.5	12.1
935	0	0	1	0	6.2	3.94	11.3
936	0	0	0	0	9.3	3.76	12.7
937	0	0	1	1	9.4	4.27	11.2
938	0	0	0	1	6.8	4.74	12.7
939	0	0	1	1	5.3	4.44	11.9
940	0	0	1	1	13.2	4.61	12.2
941	0	0	0	1	4.5	4.54	12.3
942	0	0	1	1	6.5	4.83	11.6
943	0	0	1	1	10.6	5.12	13.1
944	0	0	0	1	13.4	4.56	12.4
945	0	0	1	1	7.8	5.19	12.3
946	0	0	0	1	7.1	4.54	11.7
947	0	0	0	1	4.2	3.97	10.6
948	0	0	1	1	22	4.17	10.8
949	0	0	0	1	6.6	4.97	12.8
950	0	0	0	1	4.5	4.34	11.8
951	0	0	1	1	9.5	3.72	9.6
952	0	0	1	1	12.1	4.49	12.1
953	0	0	1	1	10.4	4.15	10.4
954	0	0	1	1	12.2	3.9	10.5
955	0	0	0	0	7.3	4.7	12
956	0	0	0	0	4.9	4.46	11.7
957	0	0	0	1	21.3	3.92	10
958	0	0	0	1	13.7	4.51	12.1
959	0	0	1	1	5.9	4.04	11.1
960	0	0	0	1	7.5	4.51	12.4
961	0	0	1	3	7.8	4.58	11.9
962	0	0	1	0	3.8	3.46	10.9
963	0	0	1	0	9.3	3.69	10.2
964	0	0	0	2	7.4	4.53	12
965	0	0	1	0	8.3	3.48	10.6
966	0	0	1	0	5.4	4.38	11.4

967	0	0	0	0	17.5	3.84	9.8
968	0	0	0	2	5.8	4.55	12.5
969	0	0	0	0	18.9	4.58	12.4
970	0	0	1	0	21.7	3.74	9.1
971	0	0	0	0	7.2	3.7	10.6
972	0	0	0	0	11.6	4.82	11.9
973	0	0	1	0	16.5	4.63	12.1
974	0	0	0	0	4.3	3.32	9.9
975	0	0	0	4	12.6	5.13	13.4
976	0	0	0	4	6.9	4.46	12.4
977	0	0	0	4	26.4	4.02	10.8
978	0	0	1	4	22.4	4.82	12.1
979	0	0	1	4	11	4.73	13.6
980	0	0	1	4	7.3	4.69	13
981	0	0	1	4	7.9	4.15	12
982	0	0	1	4	23.2	4.83	12.9
983	0	0	0	4	13.7	4.1	11
984	0	0	1	4	11.2	4.35	11.7
985	0	0	1	4	15.5	4.49	13.2
986	0	0	1	4	14.6	4.4	11.9
987	0	0	1	4	13.4	5.22	15
988	0	0	1	4	20.3	4.74	12.1
989	0	0	0	4	15.9	5.19	12.8
990	0	0	0	4	3.9	4.52	11.3
991	0	0	1	4	10.5	4.33	11.7
992	0	0	1	4	19.3	4.13	12
993	0	0	0	4	9.2	4.55	12.6
994	0	0	1	3	21.4	4.64	12.3
995	0	0	0	4	9.3	4.41	12.1
996	0	0	1	4	6.3	3.65	10.4
997	0	0	0	3	10.4	4.45	13.2
998	0	0	0	4	13.4	4.59	12.6
999	0	0	0	4	17.8	4.98	12.9
1000	0	0	1	4	13.8	4.53	12.6
1001	0	0	0	4	14.8	4.89	13.9
1002	0	0	1	3	16.1	4.68	12.1
1003	0	0	0	4	16.9	4.47	12.7
1004	0	0	0	4	15	4.85	12.8
1005	0	0	0	3	11.7	3.8	11.1
1006	0	0	0	3	4.4	5.17	14.1
1007	0	0	1	4	12.4	4.73	12.9
1008	0	0	0	4	14.8	4.26	11.2
1009	0	0	1	4	10.4	4.61	12.2
1010	0	0	0	4	4.3	4.11	11.6
1011	0	0	1	3	6.3	4.21	11.7
1012	0	0	0	4	12.3	4.76	13.4

1013	0	0	1	4	13.4	4.76	12.8
1014	0	0	0	3	12.5	4.49	12.5
1015	0	0	0	4	16.1	4.21	11.3
1016	0	0	1	4	16	4.52	12.7
1017	0	0	0	4	11	5.01	14.6
1018	0	0	1	3	12.3	4.3	12.1
1019	0	0	1	3	10.6	4.27	11.9
1020	0	0	1	3	20.3	4.74	12.8
1021	0	0	0	4	21.4	4.45	12.1
1022	0	0	1	3	10.2	4.67	12.8
1023	0	0	0	4	16.9	4.13	12.1
1024	0	0	1	4	17	4.53	12.1
1025	0	0	1	3	6	4.43	12.5
1026	0	0	1	4	12.5	4.36	11
1027	0	0	1	4	10.2	4.85	12.9
1028	0	0	1	4	11.2	4.35	12.1
1029	0	0	1	4	13.4	4.36	11.7
1030	0	0	1	3	6.5	4.49	13.6
1031	0	0	0	4	13.2	4.94	13.4
1032	0	0	0	4	13.8	4.4	11.2
1033	0	0	1	4	7.2	4.72	13
1034	0	0	1	3	9.5	5	12.9
1035	0	0	0	4	8.24	4.62	12.6
1036	0	0	0	4	16.3	4.67	13.1
1037	0	0	0	4	6.9	4.49	12.8
1038	0	0	1	3	7.5	4.72	13
1039	0	0	0	4	27.1	4.88	13.5
1040	0	0	1	3	22.8	4.69	13.2
1041	0	0	1	4	4.7	4.21	12.5
1042	0	0	1	4	14.5	3.86	11.1
1043	0	0	0	3	10.2	4.2	11.7
1044	0	0	1	4	9.2	4.04	11.3
1045	0	0	1	4	19.1	4.54	12.7
1046	0	0	1	4	4.8	4.76	12.9
1047	0	0	0	3	8.5	4.74	13
1048	0	0	1	3	8.2	3.83	10.3
1049	0	0	1	3	11.4	4.73	12.7
1050	0	0	1	3	13.3	4.53	12.1
1051	0	0	1	3	14.4	4.72	12.9
1052	0	0	0	4	12.9	4.81	12.3
1053	0	0	1	3	3.6	4.15	11.5
1054	0	0	1	4	14.3	4.26	11.7
1055	0	0	1	4	22.6	4.02	11.1
1056	0	0	1	3	21.3	4.03	11.2
1057	0	0	0	3	14.5	4.93	13.3
1058	0	0	1	4	21.4	4.31	11.4

1059	0	0	0	3	7.9	4.01	11.2
1060	0	0	0	4	20.6	4.75	12.9
1061	0	0	0	3	19.3	4.53	12.2
1062	0	0	0	3	13.5	4.63	13.2
1063	0	0	0	4	8.4	4.43	12.2
1064	0	0	0	3	12	4.39	11.5
1065	0	0	1	3	8.3	5.02	13.3
1066	0	0	1	3	7.1	4.17	11.2
1067	0	0	0	3	13.8	4.96	13
1068	0	0	1	4	19.5	3.94	11.4
1069	0	0	0	4	11.9	4.47	12.4
1070	0	0	0	3	14.1	4.31	11.2
1071	0	0	0	3	3.4	4.42	12.6
1072	0	0	1	4	7.6	5.25	14.1
1073	0	0	1	4	20	4.65	12.8
1074	0	0	0	3	2.9	4.24	11.3
1075	0	0	0	2	16.3	4.8	13
1076	0	0	0	2	6	4.57	12.6
1077	0	0	1	2	20.4	4.48	12.4
1078	0	0	0	3	21.6	4.56	12.9
1079	0	0	0	3	30.7	5.11	13.7
1080	0	0	0	3	6.2	4.34	12.4
1081	0	0	1	4	24.9	4.77	12.7
1082	0	0	0	3	5.8	4.74	13.5
1083	0	0	0	3	22.4	4.58	12.5
1084	0	0	1	4	7.7	5.47	14
1085	0	0	0	3	15.4	4.63	12.2
1086	0	0	1	3	20.3	4.97	13.6
1087	0	0	0	4	6.6	4.34	12
1088	0	0	0	3	8.9	4.59	13.5
1089	0	0	1	4	19.1	4.48	12.5
1090	0	0	0	3	17.8	4.42	12
1091	0	0	1	3	6.5	5.07	13.1
1092	0	0	0	3	13.7	4.54	12.3
1093	0	0	1	4	27.7	4.48	11.3
1094	0	0	0	3	12	4.55	12.3
1095	0	0	0	2	9.5	3.97	10.8
1096	0	0	0	4	11.1	4.1	8.6
1097	0	0	1	4	9.5	4.68	12.7
1098	0	0	0	3	19.5	4.54	11.8
1099	0	0	0	4	8.2	4.48	12.9
1100	0	0	0	3	8.8	4.88	13.2
1101	0	0	1	4	11.3	3.94	11
1102	0	0	1	3	28	4.8	13.6
1103	0	0	0	3	12.3	4.7	11.6
1104	0	0	1	3	9.9	4.49	11.6

1105	0	0	0	3	9.8	4.57	12.8
1106	0	0	0	3	24.9	4.92	13.5
1107	0	0	0	4	12.7	4.26	11.4
1108	0	0	1	4	8.5	5.11	10.5
1109	0	0	0	4	4.3	4.15	11.3
1110	0	0	1	3	13.5	4.36	12.2
1111	0	0	1	2	6.6	4.19	12
1112	0	0	1	4	14.5	4.96	13
1113	0	0	0	3	10.5	4.64	12.3
1114	0	0	1	3	7.9	5.19	13.8
1115	0	0	0	4	2	3.4	10.5
1116	0	0	0	3	27	5.03	13.3
1117	0	0	0	4	4.1	3.78	10.7
1118	0	0	0	4	13.6	4.75	13.3
1119	0	0	0	3	15.9	4.23	12
1120	0	0	0	3	14.6	4.44	11.8
1121	0	0	1	4	27.9	5.15	14.5
1122	0	0	0	4	7.8	5.22	12.7
1123	0	0	1	3	13	4.71	12.2
1124	0	0	0	2	9.3	4.73	13.1
1125	0	0	0	3	20.8	4.56	12.5
1126	0	0	1	2	4.4	4.32	12.3
1127	0	0	0	2	6.9	4.04	12.2
1128	0	0	0	3	13.7	4.58	11.7
1129	0	0	1	3	6.5	4.71	12.3
1130	0	0	1	4	9.3	4.15	12
1131	0	0	1	4	10	4.16	11.3
1132	0	0	1	3	4.8	4.16	11.9
1133	0	0	1	3	29.6	4.33	11.9
1134	0	0	1	2	18.4	4.64	11
1135	0	0	1	4	8.7	3.81	10.3
1136	0	0	1	3	5.4	4.83	13.1
1137	0	0	1	2	11.4	4.14	11.5
1138	0	0	1	3	11.8	4.91	13.1
1139	0	0	0	2	29.7	4.18	12
1140	0	0	0	4	15	4.09	11.1
1141	0	0	0	4	4.9	4.6	13
1142	0	0	1	4	10.5	5.13	14
1143	0	0	1	3	12.9	5.02	13.7
1144	0	0	0	4	16.1	3.97	11
1145	0	0	1	3	7.3	4.96	12.4
1146	0	0	0	2	7.7	4.76	12.7
1147	0	0	0	3	4.2	4.84	13
1148	0	0	1	2	6.1	4.58	12.6
1149	0	0	0	2	12.5	4.32	11.3
1150	0	0	1	2	17.1	5.05	13.5

1151	0	0	1	3	15.2	4.3	11.9
1152	0	0	1	3	9.2	4.13	11.2
1153	0	0	1	2	10.7	4.21	12.3
1154	0	0	0	3	4.5	5.01	13.5
1155	0	0	0	4	11.9	4.52	12.6
1156	0	0	1	4	8.8	4.5	11.5
1157	0	0	0	4	15.4	4.83	13.7
1158	0	0	0	3	7.6	4.18	11.4
1159	0	0	1	2	15	4.31	11.8
1160	0	0	1	4	9.9	4.85	12.3
1161	0	0	1	2	6.5	4.49	12.8
1162	0	0	1	3	18.2	4.35	12.3
1163	0	0	0	3	6.1	4.41	12.4
1164	0	0	1	2	9.1	4.82	12.7
1165	0	0	1	2	12.6	3.9	11.4
1166	0	0	1	2	9.8	5.74	14.4
1167	0	0	1	3	7.7	4.63	13.1
1168	0	0	1	2	8.8	4.08	11.5
1169	0	0	0	2	19	4.57	12.7
1170	0	0	1	2	7.6	4.68	12.6
1171	0	0	0	3	12.8	4.31	12
1172	0	0	0	3	17.1	4.54	12.8
1173	0	0	0	3	7.3	5.85	16.8
1174	0	0	1	4	13.7	4.77	13
1175	0	0	1	4	6.5	4.87	13.4
1176	0	0	1	3	9.2	3.95	10.8
1177	0	0	0	3	13.7	4.03	10.5
1178	0	0	0	2	6.7	4.09	10.4
1179	0	0	1	4	22.9	5.16	13.7
1180	0	0	0	3	7.7	4.26	12.2
1181	0	0	1	3	11	4.41	11.2
1182	0	0	0	3	20.3	4.41	12.4
1183	0	0	0	2	20.7	4.72	12.6
1184	0	0	1	2	5.9	3.48	8.9
1185	0	0	0	2	14.1	4.63	12
1186	0	0	1	2	19.7	4.97	13.5
1187	0	0	1	3	21.7	4.53	12.7
1188	0	0	1	2	3.2	4.98	13.6
1189	0	0	1	4	13.6	4.88	13.7
1190	0	0	1	2	18.5	4.25	11.9
1191	0	0	1	3	5.4	4.18	11.2
1192	0	0	0	4	14.5	4.69	12.5
1193	0	0	1	3	5.1	4.93	13.9
1194	0	0	1	2	7.7	4.67	12.2
1195	0	0	1	2	4.5	4.89	13.3
1196	0	0	0	4	7	3.84	11.1

1197	0	0	0	4	6.3	4.26	12
1198	0	0	0	2	14	4.3	11.7
1199	0	0	1	3	22.5	4.16	12.1
1200	0	0	1	2	7	4.47	12.4
1201	0	0	1	2	13.1	4.67	12.4
1202	0	0	1	2	13	4.71	12.5
1203	0	0	1	4	16.5	4.55	13.1
1204	0	0	0	2	4.4	4.44	11.7
1205	0	0	1	2	6	4.82	12.4
1206	0	0	1	4	11.3	4.57	13.8
1207	0	0	1	3	12.7	5.06	13.6
1208	0	0	1	3	7.7	4.61	12.6
1209	0	0	0	3	22.3	4.55	12.8
1210	0	0	0	3	32.3	2.61	6.7
1211	0	0	1	4	3.7	4.79	12.8
1212	0	0	0	3	21.1	4.71	13.3
1213	0	0	1	4	19.5	4.47	11.4
1214	0	0	1	3	12.6	4.23	11.6
1215	0	0	1	2	5.5	4.75	13.7
1216	0	0	1	1	15.3	3.93	11.7
1217	0	0	1	2	26.1	4.04	10.8
1218	0	0	0	4	8.1	4.13	11.2
1219	0	0	0	3	22.4	4.5	12.5
1220	0	0	1	4	17.3	3.89	10.7
1221	0	0	1	2	7.8	4.56	12.1
1222	0	0	0	3	20.2	4.16	11.8
1223	0	0	1	2	6.2	4.67	12
1224	0	0	1	2	5.6	5.12	13.9
1225	0	0	0	2	9.2	4.26	11.6
1226	0	0	1	2	5.1	4.4	12.3
1227	0	0	0	4	27.8	4.87	12.1
1228	0	0	0	2	14.3	3.6	9.3
1229	0	0	1	2	6.4	4.65	12.5
1230	0	0	0	4	6.9	4.2	11.5
1231	0	0	1	3	5.9	5.05	13.2
1232	0	0	1	2	7.1	5.14	12
1233	0	0	0	2	20.8	3.91	10.8
1234	0	0	0	1	1.9	4.8	13
1235	0	0	1	1	11.6	4.12	11.4
1236	0	0	0	1	17.2	3.76	10.3
1237	0	0	1	2	10.2	4.44	12.2
1238	0	0	1	1	14.2	4.81	13.3
1239	0	0	0	3	25.5	4.66	12.7
1240	0	0	1	4	4.2	4.99	12.8
1241	0	0	1	3	5.5	5.09	12.8
1242	0	0	1	1	7	4.29	11.2

1243	0	0	1	3	15.9	4.62	12.4
1244	0	0	0	2	14.6	4.5	12
1245	0	0	0	2	8	4.28	12.5
1246	0	0	1	4	29.5	4.95	12.2
1247	0	0	0	2	3.3	5.13	13.5
1248	0	0	1	4	5.7	4.31	12.7
1249	0	0	0	3	11.3	4.48	12.2
1250	0	0	0	1	21.7	4.78	12.4
1251	0	0	0	4	17.7	4.51	12.7
1252	0	0	0	3	13.7	4.48	12.1
1253	0	0	1	2	13.5	4.21	11.7
1254	0	0	0	2	26.9	4.23	11.7
1255	0	0	1	3	7.4	4.62	12.7
1256	0	0	0	1	18.5	5.2	14.1
1257	0	0	0	3	16.7	4.39	12
1258	0	0	0	4	9.9	4.63	14.1
1259	0	0	1	4	12.9	4.55	11.9
1260	0	0	1	1	19.4	4.53	12.6
1261	0	0	1	2	17.4	4.06	11.2
1262	0	0	1	2	6.3	4.64	12.9
1263	0	0	1	1	13.1	4	10.5
1264	0	0	1	2	15.3	4.73	12.9
1265	0	0	1	4	26.2	4.47	12.4
1266	0	0	0	1	24.1	4.56	10.4
1267	0	0	0	3	9.1	3.74	10.9
1268	0	0	0	4	14.9	4.53	12.8
1269	0	0	1	2	6.6	4.44	12.7
1270	0	0	1	4	11.2	5.14	13.5
1271	0	0	1	1	15.9	4.49	12.1
1272	0	0	0	4	13.2	4.77	13.6
1273	0	0	1	1	21.8	4.81	12.4
1274	0	0	1	2	5.9	4.61	13
1275	0	0	1	1	8.7	4.84	12.4
1276	0	0	0	2	16.1	4.17	12
1277	0	0	0	2	19.6	4.18	10.2
1278	0	0	0	2	8.6	4.4	12
1279	0	0	1	4	3.8	4.2	10.9
1280	0	0	0	4	9.3	4.04	10.6
1281	0	0	1	2	14.1	4.69	12.7
1282	0	0	0	2	12.4	4.39	13
1283	0	0	0	2	8.8	4.81	12.9
1284	0	0	1	3	15.1	4.36	12.1
1285	0	0	0	4	20	4.2	11.9
1286	0	0	0	2	10.1	4.26	11.7
1287	0	0	1	4	8	4.75	13.7
1288	0	0	1	2	21.4	5.11	13.7

1289	0	0	1	3	13.1	4.4	13.2
1290	0	0	1	2	19.9	4.06	11.3
1291	0	0	1	2	10.4	4.19	12.1
1292	0	0	0	4	21.4	4.13	11.2
1293	0	0	1	3	8.1	4.68	13
1294	0	0	0	2	9.3	4.42	12.6
1295	0	0	1	1	7.8	4.85	12.5
1296	0	0	1	1	13	4.57	11.8
1297	0	0	1	4	15.5	5.09	13.7
1298	0	0	0	2	24.8	4.19	11.5
1299	0	0	0	2	11.3	3.87	9.6
1300	0	0	0	2	18	4.26	11.2
1301	0	0	1	3	9.3	4.86	13.2
1302	0	0	0	3	9.5	4.11	11.7
1303	0	0	1	4	6.8	4.34	12.6
1304	0	0	0	2	5.3	4.73	13.4
1305	0	0	1	1	6.6	4.74	11
1306	0	0	1	2	18.1	4.22	12.2
1307	0	0	0	2	11.1	4.96	13.5
1308	0	0	1	3	15.4	4.72	12.2
1309	0	0	1	1	15.3	5.07	13.1
1310	0	0	1	4	4.2	3.81	11.4
1311	0	0	0	1	8.3	4.76	12
1312	0	0	1	3	7.6	4.83	12.8
1313	0	0	1	2	9	4.94	13.5
1314	0	0	0	2	8.7	4.29	12.2
1315	0	0	0	4	12.7	4.16	11.6
1316	0	0	1	1	13.1	5.31	13.6
1317	0	0	1	2	10.2	5.11	12.7
1318	0	0	0	2	8.5	5.05	12.3
1319	0	0	1	2	19	4.95	12.2
1320	0	0	1	3	9.5	5.21	14
1321	0	0	1	2	15.3	4.38	11.7
1322	0	0	1	2	6.4	4.45	11.8
1323	0	0	1	4	13.8	5.12	12.5
1324	0	0	1	4	17.2	5.05	13.7
1325	0	0	0	4	13.2	5.03	13
1326	0	0	1	2	9.7	4.53	12.5
1327	0	0	0	2	6.2	3.92	10.4
1328	0	0	0	2	12.3	3.77	10.4
1329	0	0	1	2	8.5	4.52	11.6
1330	0	0	0	3	8.6	4.35	12.1
1331	0	0	0	3	17.6	4.7	12.2
1332	0	0	0	1	5.7	4.07	11.2
1333	0	0	0	4	15.4	4.36	11.8
1334	0	0	0	1	13.3	4.33	11.6

1335	0	0	1	1	23.4	5.12	13.1
1336	0	0	0	2	12.8	4.73	12.3
1337	0	0	1	1	14.8	5.43	15.3
1338	0	0	1	1	18.7	4.1	11.1
1339	0	0	1	2	10.9	4.58	12.1
1340	0	0	1	1	9.5	4.54	11.9
1341	0	0	1	2	4.3	4.85	13.1
1342	0	0	1	3	14	4.54	12.2
1343	0	0	0	4	16.7	4.38	11
1344	0	0	1	1	14.1	4.52	12.8
1345	0	0	1	1	11.7	3.88	10.5
1346	0	0	1	3	6	5.13	15.1
1347	0	0	0	1	11.3	4.43	10.9
1348	0	0	0	3	11.2	4.72	12.5
1349	0	0	1	1	10.8	5.02	13.8
1350	0	0	1	3	13.1	4.22	11.4
1351	0	0	1	3	4.9	4.02	10.9
1352	0	0	1	1	9.5	4.17	11.4
1353	0	0	1	1	13.3	4.45	11.9
1354	0	0	1	1	10	2.83	8.2
1355	0	0	1	3	12	4.36	11.6
1356	0	0	1	3	27.6	4.78	12.3
1357	0	0	0	1	18.9	3.77	9.9
1358	0	0	0	1	12.5	4.14	11.3
1359	0	0	0	3	12.1	4.5	12.2
1360	0	0	1	2	23.4	4.1	10.6
1361	0	0	0	4	17.4	4.85	12.6
1362	0	0	1	4	21	4.89	12.7
1363	0	0	0	1	13.3	4.14	11.2
1364	0	0	1	1	17.8	4.31	11.5
1365	0	0	1	2	10.6	4.77	11.9
1366	0	0	1	2	5	4.61	11.6
1367	0	0	1	3	8.3	4.3	12.4
1368	0	0	0	2	17.9	4.56	12.7
1369	0	0	1	3	18.4	3.63	10.5
1370	0	0	0	1	8	4.39	11.2
1371	0	0	1	1	7	4.97	13.8
1372	0	0	1	1	16.1	4.3	11.2
1373	0	0	0	1	8.9	4.73	12.3
1374	0	0	1	2	12	4.51	11.7
1375	0	0	0	4	13.2	5.11	14.2
1376	0	0	1	1	19.3	4.43	12.2
1377	0	0	1	2	16.4	4.89	13
1378	0	0	1	1	6.6	4.83	12.7
1379	0	0	0	3	7	4.52	12.7
1380	0	0	1	2	17.8	4.69	12.1

1381	0	0	1	1	13.5	4.01	10
1382	0	0	0	4	31.6	3.6	10.3
1383	0	0	1	4	14.3	4.28	10.9
1384	0	0	1	1	16	4.23	10.9
1385	0	0	0	4	32.2	4.55	13.2
1386	0	0	1	1	13.1	5.11	13.1
1387	0	0	1	4	23.5	3.97	11
1388	0	0	1	3	8.1	4.61	12.5
1389	0	0	0	3	14.9	4.14	11.9
1390	0	0	1	4	5.2	3.86	10.7
1391	0	0	0	2	9.8	4.82	12.9
1392	0	0	1	2	7.2	4.68	13.3
1393	0	0	0	2	3.6	4.28	12.1
1394	0	0	1	3	13.1	4.94	13.6
1395	0	0	1	2	12	4.79	12
1396	0	0	0	3	12.6	4.24	10.2
1397	0	0	1	1	7.3	4.12	11.6
1398	0	0	1	4	11.4	4.56	11.8
1399	0	0	1	4	10.8	4.71	12.1
1400	0	0	1	1	7.5	5.04	12.6
1401	0	0	1	4	14.2	5.07	13.9
1402	0	0	0	2	8.5	4.84	12.5
1403	0	0	1	1	12.9	5.08	12.8
1404	0	0	0	1	6.5	4.65	12.5
1405	0	0	0	2	5.2	4.46	12.5
1406	0	0	1	4	7.8	4.15	10.4
1407	0	0	1	4	14.3	4.25	11.8
1408	0	0	1	1	13.4	4.76	13
1409	0	0	1	2	7.2	4.3	11.2
1410	0	0	1	1	5.5	4.79	12.7
1411	0	0	1	3	9.2	4.79	13.4
1412	0	0	0	2	7.3	4.45	9.9
1413	0	0	1	3	6.3	4.35	11.8
1414	0	0	1	4	25.6	5.2	13.5
1415	0	0	1	1	13.8	4.7	12.7
1416	0	0	0	2	13.7	3.28	9.2
1417	0	0	1	3	10.9	5.13	13.4
1418	0	0	1	1	6.7	4.59	12.5
1419	0	0	0	4	19.6	3.88	10.9
1420	0	0	0	1	13.7	4.38	11.6
1421	0	0	1	2	6	4.94	13.4
1422	0	0	1	2	33.2	4.4	10.3
1423	0	0	1	1	5.4	3.89	11
1424	0	0	0	1	3.9	4.58	12.7
1425	0	0	1	3	11.8	4.97	11.6
1426	0	0	0	3	21.3	4.69	13.3

1427	0	0	0	1	6.7	4.63	13
1428	0	0	1	2	6.5	4.49	12.1
1429	0	0	0	1	13.3	3.84	11.4
1430	0	0	1	1	12.2	4.68	11.4
1431	0	0	1	4	13	4.41	12.4
1432	0	0	1	1	11.7	4.14	11.8
1433	0	0	1	3	11.3	4.23	11.3
1434	0	0	1	2	3.2	4.43	11.8
1435	0	0	0	3	15.4	4.44	12
1436	0	0	0	1	9.2	4.51	10.7
1437	0	0	0	4	3.3	3.43	11.2
1438	0	0	1	1	4.4	4.99	13
1439	0	0	1	2	8.3	4.63	12.2
1440	0	0	1	1	13.3	4.81	13.6
1441	0	0	0	4	13.1	4.82	13.4
1442	0	0	1	1	14.1	3.88	10.5
1443	0	0	1	1	16.4	4.5	11.9
1444	0	0	0	1	11.1	4.38	11.4
1445	0	0	1	1	9.7	5.3	12.5
1446	0	0	1	1	12.8	3.07	8.6
1447	0	0	1	2	5.6	4.08	10.9
1448	0	0	0	1	8.8	4.12	10.9
1449	0	0	1	1	17.9	4.73	12.6
1450	0	0	0	1	3.4	4.6	12.3
1451	0	0	0	2	19.6	4.43	11.9
1452	0	0	0	1	9.5	3.33	9.9
1453	0	0	1	3	18.2	4.57	12.7
1454	0	0	0	1	12.6	4.6	12.8
1455	0	0	1	1	30.8	3.91	10
1456	0	0	0	3	19.6	4.74	12.9
1457	0	0	1	1	11.6	3.56	9.8
1458	0	0	0	2	13.4	4.84	13.4
1459	0	0	1	1	6.2	5.13	12
1460	0	0	1	1	25.9	4.53	12.4
1461	0	0	0	1	11.1	4.1	10.9
1462	0	0	1	1	17.6	3.81	12.8
1464	0	0	0	1	17.8	3.7	12.5
1465	0	0	0	1	21.2	4.11	11.2
1466	0	0	0	4	10.2	5.1	13.9
1467	0	0	0	2	4.8	3.72	10.2
1468	0	0	1	1	19.9	4.58	11.7
1469	0	0	1	1	11.7	4.39	13.8
1470	0	0	0	1	24.7	3.46	9.4
1471	0	0	1	2	5.6	4.54	12
1472	0	0	1	1	7	3.5	11
1473	0	0	1	1	9.8	4.64	12.4

1474	0	0	0	0	6.9	4.38	11.9
1475	0	0	0	2	5.5	4.47	12.1
1476	0	0	1	1	7.6	4.4	12.3
1477	0	0	1	2	6.4	4.31	11.7
1478	0	0	0	4	8.8	5.15	14
1479	0	0	1	3	17.4	4.68	12.3
1480	0	0	1	1	4.8	4.74	12.5
1481	0	0	1	2	19.9	4.55	12
1482	0	0	0	2	2.7	5	12.2
1483	0	0	1	2	2.6	4.53	11.9
1484	0	0	1	1	16.7	3.32	9.9
1485	0	0	1	1	15.3	3.94	10.6
1486	0	0	1	0	18.6	3.97	11
1487	0	0	1	4	13.2	4.5	12.4
1488	0	0	0	1	11.4	4.07	11.3
1489	0	0	0	2	5.6	4.7	12.8
1490	0	0	0	1	25.2	4	10.6
1491	0	0	0	1	7.7	4.7	12
1492	0	0	1	1	5.2	4.54	11.8
1493	0	0	0	1	7.6	4.7	12.5
1494	0	0	0	1	7.6	4.51	11.7
1495	0	0	1	2	9.4	4.8	10
1496	0	0	1	1	9.8	4.25	11
1497	0	0	0	1	9.5	4.95	13
1498	0	0	0	1	17	4.56	12.4
1499	0	0	1	0	7.2	3.02	10.4
1500	0	0	1	1	21.2	4.52	12.6
1501	0	0	1	1	23.4	4.57	11.2
1502	0	0	1	3	5.2	4.73	11.8
1503	0	0	1	1	12.2	4.04	11.2
1504	0	0	0	0	4.9	3.26	11.5
1505	0	0	1	1	9.8	3.1	9.1
1506	0	0	1	3	6.5	4.62	12.3
1507	0	0	1	3	18.3	4.35	12
1508	0	0	1	1	8.8	4.59	11
1509	0	0	0	1	5	4.84	12.9
1510	0	0	1	1	6.8	4.71	13.2
1511	0	0	0	4	8.4	4.93	13.8
1512	0	0	1	1	18.3	4.3	12.5
1513	0	0	0	2	7.4	4.26	11.8
1514	0	0	0	1	4.8	3.95	10
1515	0	0	0	0	11.2	4.13	13.6
1516	0	0	0	1	6	4.74	13
1517	0	0	0	1	8.1	3.75	9.9
1518	0	0	1	1	14.6	4.47	12
1519	0	0	1	4	17.7	4.91	13.6

1520	0	0	1	1	21.4	4.03	11.2
1521	0	0	0	1	9.5	4.95	12.1
1522	0	0	1	0	3	3.18	9.5
1523	0	0	0	1	7.3	4.24	11.5
1524	0	0	1	0	10.3	4.43	15
1525	0	0	0	0	15.6	3.83	11.4
1526	0	0	1	1	26.5	3.62	9.9
1527	0	0	1	4	19.1	4.43	11.8
1529	0	0	0	1	5.7	4.5	11.8
1530	0	0	0	1	10.6	3.99	10.8
1531	0	0	1	3	15.1	5.15	11.5
1532	0	0	0	1	6.1	4.36	12
1533	0	0	1	0	3.1	3.7	10.4
1534	0	0	1	3	23	4.33	11.8
1535	0	0	1	1	16.5	4.33	11.9
1536	0	0	1	0	6.2	3.55	10.8
1537	0	0	0	3	8.4	4.95	13.8
1538	0	0	0	3	8.4	4.29	12.1
1539	0	0	1	0	10.4	3.77	12.8
1540	0	0	0	1	2.5	4.66	12.1
1541	0	0	0	1	11.2	4.44	12.1
1542	0	0	0	1	5.7	4.8	12.2
1543	0	0	1	4	5.5	4.99	14.5
1544	0	0	1	0	13.1	3.27	9.8
1545	0	0	1	3	11.1	4.64	12.7
1546	0	0	0	2	15.9	4.38	11.5
1547	0	0	1	4	9.5	3.95	11
1548	0	0	0	4	10.6	4.05	12
1549	0	0	1	1	11.8	4.3	11.6
1550	0	0	1	1	24.6	4.35	11.4
1551	0	0	1	0	12.5	3.23	9.5
1552	0	0	0	3	0.4	3.05	7.8
1553	0	0	1	1	6.5	5.2	13.1
1554	0	0	0	1	7.2	4.42	12.2
1555	0	0	1	0	7.9	3.54	10.5
1556	0	0	1	1	4.6	4.67	12.4
1557	0	0	0	1	14.4	4.18	11.4
1558	0	0	1	4	11.6	4.57	13.1
1559	0	0	1	1	7.2	4.84	12.9
1560	0	0	0	2	7.9	4.25	12.1
1561	0	0	0	1	11.8	4.14	11.6
1562	0	0	0	1	13.9	4.82	12.7
1563	0	0	0	1	12.2	4.78	13
1564	0	0	0	1	6.9	5.52	13.4
1565	0	0	1	3	11.1	3.94	10.4
1566	0	0	1	1	15.7	4.64	12.4

1567	0	0	0	2	19.6	4.61	12.9
1568	0	0	1	2	12.8	4.85	12.5
1569	0	0	1	1	7.6	4.62	12.6
1570	0	0	0	1	12.5	4.58	12.1
1571	0	0	1	1	15.3	3.71	9.7
1572	0	0	1	2	2.3	4.61	12
1573	0	0	1	0	9.2	4.82	11.8
1574	0	0	1	0	12.2	4.02	13
1575	0	0	0	4	13.8	4.01	11.5
1576	0	0	1	2	11.8	3.86	10.7
1577	0	0	1	0	6.4	3.29	9.3
1578	0	0	1	1	7.4	3.95	10.9
1579	0	0	0	0	7.8	3.72	11
1580	0	0	1	0	9.2	2.92	9.9
1581	0	0	1	2	11	4.45	12.1
1582	0	0	1	1	6.5	4.34	11.9
1583	0	0	1	1	16	4.49	11.1
1584	0	0	1	1	8	3.98	11.2
1585	0	0	0	2	27.8	4.74	13
1586	0	0	0	2	3.7	4.34	12.2
1587	0	0	0	1	19.9	3.87	11.1
1588	0	0	0	1	2.1	4.72	12
1589	0	0	1	1	4.2	4.87	12.5
1590	0	0	1	1	7.6	4.51	10.8
1591	0	0	1	1	19.2	4.14	11.4
1592	0	0	1	2	6.9	4.87	13.1
1593	0	0	0	1	10.2	5.04	13.5
1594	0	0	1	0	9.3	3.39	9.9
1595	0	0	0	1	6.9	4.6	12.8
1596	0	0	0	4	7.9	4.69	12.5
1597	0	0	1	1	7.7	4.75	11.8
1598	0	0	0	0	11.5	4.2	9.3
1599	0	0	0	1	21.4	4.14	11
1600	0	0	1	3	24.6	5.01	13.2
1601	0	0	0	3	10.7	3.66	9.5
1602	0	0	1	0	9.5	4.58	10.4
1603	0	0	1	0	16.9	3.65	10.4
1604	0	0	1	1	7.3	4.82	11.8
1605	0	0	0	0	9.6	4.29	13.8
1606	0	0	0	2	11.3	4.44	11.6
1607	0	0	1	1	6.4	4.86	12.1
1608	0	0	1	0	9.7	2.76	8.4
1609	0	0	1	0	13.7	4.12	10.9
1610	0	0	1	0	9.6	3	8.9
1611	0	0	1	0	9.1	3.74	10.5
1612	0	0	0	4	10.3	5.14	11.8

1613	0	0	1	1	8.9	3.96	10.5
1614	0	0	1	2	15.2	4.99	13.2
1615	0	0	1	3	20.2	4.17	11.8
1616	0	0	1	1	22.5	4.85	12.1
1617	0	0	1	1	5.2	4.37	11.8
1618	0	0	1	1	16	4.82	12.6
1619	0	0	1	4	18.1	4.92	12.2
1620	0	0	1	1	3.9	4.61	11.2
1621	0	0	1	2	3.2	4.7	12.6
1622	0	0	0	1	8	4.87	12.2
1623	0	0	0	2	10.7	4.38	11.4
1624	0	0	1	2	13.7	4.43	11.4
1625	0	0	0	0	18.4	3.19	10.4
1626	0	0	1	0	10.4	2.94	8.6
1627	0	0	1	1	13.7	4.6	11.9
1628	0	0	0	1	9.4	4.12	11.4
1629	0	0	0	1	3	4.93	12.9
1630	0	0	0	2	8	4.67	12.5
1631	0	0	1	0	8.3	3.87	13
1632	0	0	1	0	16.4	3.69	10.7
1633	0	0	1	0	10.8	4.25	11.7
1634	0	0	1	1	5.8	4.76	13.2
1635	0	0	0	1	9.2	5.38	14.3
1636	0	0	1	1	6.6	4.95	12.7
1637	0	0	1	4	18.7	4.4	11.5
1638	0	0	0	0	15.8	3.5	10.1
1639	0	0	1	2	15.1	4.64	12.1
1640	0	0	0	2	12.4	4.21	11.3
1641	0	0	1	4	13.4	4.32	11.4
1642	0	0	0	1	15.1	4.17	11.1
1643	0	0	1	3	9.1	4.82	13.4
1644	0	0	1	1	23.7	3.36	8.8
1645	0	0	1	2	6.6	4.57	12.6
1646	0	0	0	3	5.6	4.27	10.9
1647	0	0	0	1	10	3.46	10.3
1648	0	0	1	1	17.6	4.22	11.3
1649	0	0	0	0	6	3.08	9.5
1650	0	0	0	1	3.9	4.2	11.6
1651	0	0	1	1	10.5	5.07	13.8
1652	0	0	0	1	11.3	4.69	12.4
1653	0	0	1	2	5.6	4.46	11.5
1654	0	0	1	4	10.2	3.88	10.7
1655	0	0	0	1	9.7	4.05	10.2
1656	0	0	1	1	13.9	4.27	11.7
1657	0	0	0	2	3.6	4.73	12.9
1658	0	0	1	1	14	4.25	12.1

1659	0	0	1	1	18.8	4.56	11.1
1660	0	0	0	1	6.8	3.83	10.3
1661	0	0	1	1	17.5	3.79	9.6
1662	0	0	1	2	5.9	5.02	12.2
1663	0	0	1	1	4.2	4.76	12.5
1664	0	0	1	1	4	4.2	11.6
1665	0	0	0	0	5.7	3.24	10.1
1666	0	0	1	1	5.6	4.13	11.3
1667	0	0	1	2	7.1	4.9	13.3
1668	0	0	0	4	13.2	4.03	11.6
1669	0	0	0	1	12.4	3.9	10.2
1670	0	0	1	1	12	4.49	12.2
1671	0	0	1	0	11.9	4.03	10.7
1672	0	0	1	1	20.1	4.46	10.9
1673	0	0	0	2	5.5	4.14	11.8
1674	0	0	1	4	22.4	4.38	12.5
1675	0	0	0	4	7.8	5.05	13.6
1676	0	0	1	0	10.6	4.22	13.5
1677	0	0	0	1	8.2	4.5	12.3
1678	0	0	1	1	11.8	3.92	11.1
1679	0	0	0	1	5.1	4.84	12.6
1680	0	0	0	3	0.7	3.17	9.7
1681	0	0	1	4	4.3	4.51	12
1682	0	0	0	4	13.6	4.65	12.1
1683	0	0	0	3	18.3	4.64	12.6
1684	0	0	0	1	14.1	4.84	13
1685	0	0	1	0	16.5	3.36	9.9
1686	0	0	0	4	13	4.34	11.6
1687	0	0	0	1	17.1	4.3	12.4
1688	0	0	1	0	15.2	4.52	12.5
1689	0	0	0	1	4.9	4.79	12.6
1690	0	0	0	0	11.4	3.68	11.7
1691	0	0	0	1	5.1	4.52	12.3
1692	0	0	1	0	7.9	4.97	17.2
1693	0	0	1	3	8.4	4.51	12.1
1694	0	0	1	1	11	4.21	11.2
1695	0	0	0	2	8.7	4.2	11.4
1696	0	0	0	1	9.4	3.94	10.6
1697	0	0	0	1	9	4.84	12.3
1698	0	0	0	1	22	4.76	12.8
1699	0	0	1	1	7.4	4.71	12.1
1700	0	0	0	1	19.9	3.61	10
1701	0	0	1	1	17.5	3.61	9.5
1702	0	0	1	1	11.1	4.34	11.6
1703	0	0	1	3	9.3	5.24	13.8
1704	0	0	0	4	4.9	4.33	12

1705	0	0	1	1	11	5.09	13.2
1706	0	0	1	1	3.6	3.72	9.9
1707	0	0	1	1	5.9	5.01	12.2
1708	0	0	1	1	9.1	4.43	11.8
1709	0	0	1	1	13.5	4.58	11.1
1710	0	0	0	1	27.2	4.23	11.8
1711	0	0	1	2	2.9	4.2	11
1712	0	0	1	4	20.1	3.97	11.6
1713	0	0	0	3	11.3	4.62	11.8
1714	0	0	1	0	13.1	3.22	9.3
1715	0	0	1	4	22.2	4.45	12.6
1716	0	0	1	3	8.2	3.94	10.1
1717	0	0	1	1	18	4.63	12
1718	0	0	1	1	7.5	5.08	12.4
1719	0	0	1	1	12.2	4.53	12.9
1720	0	0	0	1	18.2	4.7	12.3
1721	0	0	1	1	8.5	5.26	13.5
1722	0	0	1	1	17.1	4.44	10.9
1723	0	0	1	2	8.2	3.8	10.4
1724	0	0	0	1	10	5.17	13
1725	0	0	0	2	8.8	4.59	12.6
1726	0	0	1	0	7.9	3.17	9.1
1727	0	0	1	0	28	3.99	11.3
1728	0	0	0	1	11	4.56	12.2
1729	0	0	1	0	12.6	4	11.2
1730	0	0	1	4	9.8	4.26	11.8
1731	0	0	1	0	15.7	4.03	10.7
1732	0	0	0	2	9.4	4.31	11.2
1733	0	0	1	1	2.8	4.96	13.6
1734	0	0	0	4	15.1	4.72	12
1735	0	0	0	2	5.5	4.52	12.4
1736	0	0	0	3	15.1	4.46	12.6
1737	0	0	0	2	5	4.74	12.7
1738	0	0	1	1	10.7	4.3	11.7
1739	0	0	1	0	16.1	3.55	10.1
1740	0	0	0	2	12	4.09	11.3
1741	0	0	1	2	6	4.15	10.8
1742	0	0	0	0	26.5	4.49	12.5
1743	0	0	1	1	8.6	4.37	11.9
1744	0	0	1	2	9.2	4.62	12.1
1745	0	0	0	1	24.9	4.21	11
1746	0	0	0	4	21.2	4.47	11.6
1747	0	0	1	3	10.2	4.53	12.3
1748	0	0	1	1	8.9	4.73	12.8
1749	0	0	0	0	9.5	4.21	11.8
1750	0	0	1	1	8.2	4.03	11.6

1751	0	0	1	0	18.8	2.68	7.6
1752	0	0	1	1	12.7	3.69	10.5
1753	0	0	0	1	9.5	4.88	13.7
1754	0	0	1	0	7	4.3	11.7
1755	0	0	0	0	9	4.11	11.3
1756	0	0	1	0	19.7	3.91	12.1
1757	0	0	0	1	17.2	5.2	14.3
1758	0	0	1	0	19.4	2.85	9.1
1759	0	0	0	3	6.2	4.41	12
1760	0	0	1	4	15.8	5.22	13.3
1761	0	0	1	1	13.2	4.37	11.2
1762	0	0	1	3	13.1	4.63	12.8
1763	0	0	0	1	2.7	4.28	11.9
1764	0	0	0	3	9.2	4.37	11.4
1765	0	0	1	2	13.3	4.98	14
1766	0	0	0	0	5.2	3.65	9
1767	0	0	1	1	8.8	4.74	12.3
1768	0	0	0	2	12.6	4.42	12.5
1769	0	0	1	0	9.8	4.46	12.4
1770	0	0	0	0	8.8	4.79	12.1
1771	0	0	1	4	10.7	4.37	12.2
1772	0	0	0	0	37.8	4.84	12
1773	0	0	0	1	15.6	4.69	10.3
1774	0	0	0	3	8.4	4.47	11.8
1775	0	0	1	3	11.7	4.85	13.1
1776	0	0	0	2	12.4	3.81	10.5
1777	0	0	1	3	12.4	4.81	13.5
1778	0	0	0	0	11.3	3.24	9.6
1779	0	0	1	0	10.2	4.32	11.3
1780	0	0	0	0	7.3	3.65	10.8
1781	0	0	1	0	9.3	2.95	8.4
1782	0	0	1	4	6.5	4.43	11.7
1783	0	0	1	4	14.5	4.18	11.7
1784	0	0	1	2	6.2	5.04	11.3
1785	0	0	1	4	8.2	4.7	11.6
1786	0	0	0	1	16.9	4.39	12.4
1787	0	0	1	0	13	3.73	12
1788	0	0	1	4	12.1	5.1	13.5
1789	0	0	0	1	8.7	4.89	12.8
1790	0	0	0	0	20.5	3.56	10.2
1791	0	0	0	4	13	4.06	11.8
1792	0	0	1	0	20.6	5.07	12.4
1793	0	0	0	1	8.7	4.34	12
1794	0	0	0	1	12.1	4.45	12.3
1795	0	0	0	4	16.2	3.44	9.9
1796	0	0	0	1	5.2	4	11.3

1797	0	0	0	2	13.4	4.64	13
1798	0	0	1	0	13.1	3.74	9.7
1799	0	0	0	0	11.1	3.69	11.2
1800	0	0	0	1	6.2	4.11	11.6
1801	0	0	1	4	25.9	4.89	12.2
1802	0	0	0	0	7.2	4.31	12
1803	0	0	0	0	15.2	4.35	11.5
1804	0	0	0	3	5.5	4.35	12.2
1805	0	0	0	1	14	5.42	13.5
1806	0	0	1	3	19.4	4.56	11.9
1807	0	0	1	1	14.2	4.37	11.7
1808	0	0	0	0	18.9	5.39	19.6
1809	0	0	1	0	9.3	3.37	10.5
1810	0	0	1	4	16.3	4.73	12
1811	0	0	0	0	10.9	3.88	12.1
1812	0	0	1	2	18.4	5.2	13.7
1813	0	0	1	2	10.6	4.81	13.9
1814	0	0	0	3	12.2	4.86	13.1
1815	0	0	0	2	5.7	4.49	11.5
1816	0	0	1	1	4.8	4.51	12.3
1817	0	0	0	1	6.1	4.5	11.7
1818	0	0	1	1	3.2	4.62	11.6
1819	0	0	1	1	4.7	4.97	12.9
1820	0	0	1	1	5.7	4.48	13.1
1821	0	0	1	0	5.5	5.17	12.4
1822	0	0	1	1	14.2	4.81	12.5
1823	0	0	1	2	7.8	4.73	12.7
1824	0	0	0	3	7.5	4.07	11
1825	0	0	0	0	8.8	5.15	18.7
1826	0	0	1	3	14.4	4.65	12.4
1827	0	0	1	4	3.4	4.07	11.6
1828	0	0	1	1	13.7	4.55	11.8
1829	0	0	1	0	15.9	4.23	11.8
1830	0	0	1	2	4.3	4.52	12.5
1831	0	0	0	0	17.1	3.75	10
1832	0	0	1	3	7.4	4.28	11.5
1833	0	0	0	0	11.8	3.38	10.4
1834	0	0	0	0	8.5	3.46	10.3
1835	0	0	0	3	8.5	4.49	11.6
1836	0	0	1	2	7.3	4.74	13.2
1837	0	0	1	0	13.5	4.19	14.9
1838	0	0	0	0	12.5	5.14	17.9
1839	0	0	1	0	9.4	3.99	14.2
1840	0	0	0	4	8.4	4.48	12.2
1841	0	0	1	3	11.2	4.18	10.8
1842	0	0	1	0	12.2	3.77	10.8

1843	0	0	0	0	11.7	4.81	11.9
1844	0	0	0	1	10.3	4.47	11.9
1845	0	0	0	1	15.7	4.78	12.8
1846	0	0	1	2	9.8	5.59	14.3
1847	0	0	1	1	13.8	4.41	13.5
1849	0	0	1	0	26.3	4.25	10.1
1850	0	0	0	0	17.5	4.46	15.9
1851	0	0	0	0	8.7	4.52	11.3
1852	0	0	0	1	25.5	4.37	10.8
1853	0	0	1	0	16.8	4.01	10.8
1854	0	0	0	0	6.3	4.19	11
1855	0	0	1	3	15.5	4.55	11.2
1856	0	0	0	2	12.3	4.34	12.2
1857	0	0	0	2	19.7	3.93	10.4
1858	0	0	1	2	6.8	4.58	12.1
1859	0	0	0	0	16.2	4.5	12.1
1860	0	0	1	1	6	6.05	16
1861	0	0	1	0	12.8	3.31	9.1
1862	0	0	0	0	5.1	4.03	11.6
1863	0	0	0	4	13	4.25	11.3
1864	0	0	1	4	8.1	4.65	12.1
1865	0	0	1	1	4.5	5.16	13.6
1866	0	0	1	0	9	4.48	11.6
1867	0	0	1	0	9.6	4.33	11.5
1868	0	0	0	0	18.6	4.54	11.8
1869	0	0	0	1	7	5.03	13.3
1870	0	0	1	1	9.6	4.73	12.8
1871	0	0	1	2	6.7	4.87	13.2
1872	0	0	0	0	7.7	4.53	13.5
1873	0	0	0	2	17.2	4.44	12
1874	0	0	0	1	7.3	4.67	12.7
1875	0	0	1	2	21.9	4.01	10.6
1876	0	0	1	0	18.4	4.33	11
1877	0	0	0	2	7.3	4.62	13
1878	0	0	1	1	16.4	5.03	13.5
1879	0	0	1	2	13.1	5.4	14.9
1880	0	0	1	1	4	4.64	12.5
1881	0	0	0	1	17.9	4.09	11.6
1882	0	0	0	0	6.4	4.65	15.6
1883	0	0	1	2	6.3	4.43	11.2
1884	0	0	0	3	21.6	5.08	14
1885	0	0	1	0	11	4.03	11.8
1886	0	0	0	1	5.7	4.89	12.3
1887	0	0	1	0	11.1	4.43	10.4
1888	0	0	0	0	14.3	4.2	12.2
1889	0	0	0	4	8.2	4.5	13

1890	0	0	0	0	5.7	3.97	11.3
1891	0	0	0	3	9	4.44	12.2
1892	0	0	1	1	4.1	4.94	12.9
1893	0	0	0	0	4.2	4.61	13
1894	0	0	0	2	3.9	4.52	13
1895	0	0	1	0	14	3.5	10.1
1896	0	0	0	1	12.6	4.44	12
1897	0	0	1	1	5.9	4.94	13.4
1898	0	0	0	2	8.1	4.36	11.9
1899	0	0	1	1	8.3	5.31	12.9
1900	0	0	0	4	13.4	4.4	11.4
1901	0	0	1	4	8.2	4.87	13.7
1902	0	0	1	0	8.8	3.24	10.7
1903	0	0	0	4	15.4	4.73	13.4
1904	0	0	0	3	5.3	4.23	12.6
1905	0	0	0	0	16.7	5.12	11.8
1906	0	0	0	2	3	4.29	11.7
1907	0	0	0	4	6.8	4.64	12.6
1908	0	0	1	1	18.9	4.69	12.5
1909	0	0	1	0	10.5	4.33	10.9
1910	0	0	1	2	7.4	5.01	13.3
1911	0	0	0	2	6.2	4.21	12
1912	0	0	1	0	8.1	3.77	10.8
1913	0	0	1	0	11.5	4.09	13.4
1914	0	0	1	0	42.8	3.56	9.6
1915	0	0	1	0	7.5	4.26	11.1
1916	0	0	0	0	6.7	4.33	12
1917	0	0	0	2	6.1	4.52	12.2
1918	0	0	0	0	17.4	3.58	9.6
1919	0	0	0	1	6.3	4.2	11
1920	0	0	1	1	13.7	4.18	12
1921	0	0	0	0	10.4	5.04	13.2
1922	0	0	1	0	12.7	3.35	9.5
1923	0	0	1	3	21.1	4.83	12.9
1924	0	0	0	4	5.8	4.5	12
1925	0	0	0	0	11.6	3.43	10.8
1926	0	0	1	4	9.7	4.75	10.2
1927	0	0	1	1	16	4.76	12.6
1928	0	0	0	2	13.7	4.75	12.1
1929	0	0	0	3	4.4	4.05	10.5
1930	0	0	1	3	15.7	4.22	9.6
1931	0	0	1	3	20.2	4.36	10.9
1932	0	0	1	0	9.3	3.03	9.9
1933	0	0	1	1	20.9	4.05	11
1934	0	0	0	1	8.8	4.38	11.2
1935	0	0	1	1	6.9	4.19	9

1936	0	0	1	0	8.1	3.42	10
1937	0	0	0	4	7.2	4.54	12.5
1938	0	0	0	1	23.2	4.35	12.1
1939	0	0	0	4	8.9	4.8	13.3
1940	0	0	0	2	4.6	4.66	13.2
1941	0	0	1	1	8.1	4.29	11.6
1942	0	0	0	0	6.6	4.16	11.4
1943	0	0	0	4	6.5	4.02	11.3
1944	0	0	0	1	11.2	4.47	11.2
1945	0	0	0	4	4.4	4.65	12.8
1946	0	0	0	2	32.9	4.62	13
1947	0	0	1	2	12.9	5.1	13.2
1948	0	0	0	1	7.6	4.31	12.6
1949	0	0	1	0	2.4	4.08	11.9
1950	0	0	1	2	11.4	4.4	11.8
1951	0	0	1	1	22.9	5.15	13.1
1952	0	0	1	0	18.6	3.87	10.8
1953	0	0	1	0	8.8	3.1	8.8
1954	0	0	1	0	4.2	4.77	13.2
1955	0	0	0	0	14.5	3.56	10.4
1956	0	0	1	4	12.9	4.19	11.9
1957	0	0	0	2	9.9	4.5	12.8
1958	0	0	1	0	9.8	3.46	11.3
1959	0	0	1	0	16.9	4.27	11
1960	0	0	0	1	22.9	4.13	10.5
1961	0	0	1	4	17.5	4.9	12.7
1962	0	0	1	4	10.3	4.74	12.3
1963	0	0	0	1	6.5	4.64	11.7
1964	0	0	0	1	11.5	5	11.8
1965	0	0	0	3	13.8	4.09	11
1966	0	0	0	0	19.3	4.84	12.3
1967	0	0	0	0	21.3	3.81	11.4
1968	0	0	0	2	12.8	4.43	11.6
1969	0	0	0	0	23.1	4.28	11.8
1970	0	0	0	0	8	4.47	12.4
1971	0	0	1	2	5.4	4.46	11.4
1972	0	0	0	3	21.9	4.94	13.5
1973	0	0	1	0	6.8	3.35	10.4
1974	0	0	1	1	18.1	4.55	12.4
1975	0	0	0	2	21.2	4.92	12.3
1976	0	0	0	1	9.9	5.24	13.9
1977	0	0	1	1	6.6	4.38	12.2
1978	0	0	0	1	7.4	3.94	10.9
1979	0	0	0	0	9.5	4.39	12.1
1980	0	0	1	0	6.3	4.08	10.3
1981	0	0	0	4	10.6	4.32	12

1982	0	0	1	1	15.7	4.46	11.4
1983	0	0	0	1	15.1	4.19	11.1
1984	0	0	0	0	10.2	3.01	8.6
1985	0	0	1	1	24.3	4.82	12.6
1986	0	0	0	2	9.4	4.75	10.2
1987	0	0	0	4	5.8	4.24	12.8
1988	0	0	0	2	28.8	4.2	11.3
1989	0	0	1	1	22.9	4.79	12.8
1990	0	0	1	1	12.9	4.52	12.4
1991	0	0	0	2	7.9	4.16	11.9
1992	0	0	1	0	13.4	3.49	9.8
1993	0	0	1	0	19.4	4.35	11.3
1994	0	0	1	0	4.7	4.75	11.9
1995	0	0	0	1	9.9	4.91	13.1
1996	0	0	1	0	13.4	4.29	11.3
1997	0	0	1	4	11.8	4.55	11.5
1998	0	0	1	0	6.8	3.26	10
1999	0	0	1	0	14.5	3.25	9.9
2000	0	0	1	0	13.5	3.35	9.5
2001	0	0	0	0	7.2	3.11	9.6
2002	0	0	0	0	7.9	3.91	11.4
2003	0	0	0	1	15.5	4.8	12.4
2004	0	0	0	1	9.4	4.53	12.1
2005	0	0	1	3	17.7	4.69	12.4
2006	0	0	1	3	14.7	4.41	12.2
2007	0	0	0	4	10.2	4.53	12.6
2008	0	0	1	0	5.8	3.06	9.5
2009	0	0	1	0	5.5	3.75	12.9
2010	0	0	0	0	7.3	3.21	9.7
2011	0	0	1	1	6.2	4.09	11.1
2012	0	0	0	1	43.7	3.94	9.9
2013	0	0	0	0	5.2	4.41	11.7
2014	0	0	0	1	3.3	4.68	13.4
2015	0	0	1	0	7	4.34	11.7
2016	0	0	1	4	4.9	3.46	10.1
2017	0	0	1	1	10.1	4.83	12.6
2018	0	0	1	1	8.3	4.4	11.5
2019	0	0	1	0	3.5	3.53	12.7
2020	0	0	0	1	4.1	4.56	12.5
2021	0	0	1	0	7.2	4.38	11.8
2022	0	0	1	1	17.3	4.79	12.2
2023	0	0	1	4	14.3	4.15	11
2024	0	0	1	3	10.1	4.17	11.4
2025	0	0	1	0	13.3	4.22	11.3
2026	0	0	0	0	18.5	4.73	16.4
2027	0	0	1	2	9.1	4.37	11.6

2028	0	0	1	1	9	4.53	11.5
2029	0	0	0	0	22.6	4.19	10.7
2030	0	0	1	0	9.5	3.67	13.7
2031	0	0	1	0	10.3	4.25	12.6
2032	0	0	1	0	11.4	4.58	13.7
2033	0	0	0	2	10	4.15	10.2
2034	0	0	1	0	4	3.25	9.7
2035	0	0	0	0	17.2	3.67	11.6
2036	0	0	1	3	13.2	4.8	12.6
2037	0	0	1	0	8.1	4.31	10.8
2038	0	0	0	3	10	3.12	8.6
2039	0	0	0	0	6	4.61	12
2040	0	0	1	0	17.8	3.96	10.9
2041	0	0	1	0	4.3	4.18	11.7
2042	0	0	0	4	2.8	4.15	11.5
2043	0	0	0	1	7.1	4.23	10.9
2044	0	0	0	0	10	3.79	11.1
2045	0	0	1	1	10.9	4.82	12.9
2046	0	0	1	0	7.6	3.53	10.5
2047	0	0	0	0	3	3.83	10.3
2048	0	0	0	1	7.8	4.64	12
2049	0	0	1	0	9.8	4.59	11.1
2050	0	0	0	2	6.9	4.74	13.3
2051	0	0	1	1	17.4	4.36	11.7
2052	0	0	0	4	17.5	3.33	10.6
2053	0	0	1	0	9.6	5.09	11.6
2054	0	0	1	3	12.1	5.14	13.1
2055	0	0	0	1	15	4.81	12.6
2056	0	0	1	1	17	4.41	11.5
2057	0	0	0	4	9.5	4.02	11.4
2058	0	0	1	4	6.2	4.54	12.9
2059	0	0	1	0	13.8	4.12	11.1
2060	0	0	1	0	17.3	3.41	10.1
2062	0	0	0	3	9.6	4.76	13
2063	0	0	0	4	29.3	4.86	13.1
2064	0	0	1	0	18.8	4.19	11.2
2065	0	0	1	4	3.7	4.34	11.8
2066	0	0	0	4	22.3	4.51	12.3
2067	0	0	1	0	19.7	4.33	12.1
2068	0	0	1	4	6.9	4.81	12
2069	0	0	0	0	17	3.58	10.6
2070	0	0	1	0	19.7	3.9	11.2
2071	0	0	1	0	6.2	4.47	12
2072	0	0	1	0	15.1	2.61	8.7
2073	0	0	0	0	9.7	4	10.7
2074	0	0	1	1	28.9	4.3	11.7

2075	0	0	1	0	14.9	4.08	10.9
2076	0	0	0	1	12.6	4.49	11.1
2077	0	0	0	1	21.2	3.97	10.5
2078	0	0	0	1	14.3	4.66	12.4
2079	0	0	1	0	12.4	4.1	9.1
2080	0	0	1	0	10.2	4.12	14
2081	0	0	0	4	7.9	4.37	11.6
2082	0	0	1	3	16.9	5.02	13.5
2083	0	0	0	1	15.1	3.01	9.7
2084	0	0	1	2	5.4	5.23	13.9
2085	0	0	0	0	6.7	4.52	12.2
2086	0	0	0	2	22.4	4.85	11.8
2087	0	0	1	1	11.2	4.88	12.8
2088	0	0	1	4	17.9	4.66	12.1
2089	0	0	0	2	17.5	4.78	12.3
2090	0	0	1	4	15.1	5.07	12.4
2091	0	0	1	0	13.5	3.33	9.3
2092	0	0	1	4	8.2	4.73	12.9
2093	0	0	1	1	13	3.99	10.5
2094	0	0	0	1	15.9	3.86	11
2095	0	0	1	1	19.2	4.2	10.4
2096	0	0	1	4	12.5	5.51	13.7
2097	0	0	1	0	8.8	4.61	12.7
2098	0	0	0	0	16.7	4.46	14.8
2099	0	0	1	1	13.9	3.61	9.4
2100	0	0	1	3	26.9	5.02	12.6
2101	0	0	1	0	17.9	3.92	11.2
2102	0	0	1	0	16	3.51	10.2
2103	0	0	0	0	6.7	3.98	11
2104	0	0	0	0	12.8	4.92	16.4
2105	0	0	1	2	8.2	5.27	14.4
2106	0	0	1	4	9.4	4.44	12
2107	0	0	1	4	9.8	4.7	12.5
2108	0	0	1	4	6.9	4.77	12
2109	0	0	1	1	33.7	4.5	11.3
2110	0	0	1	2	10.6	4.71	11.9
2111	0	0	1	4	23.2	5.06	13.8
2112	0	0	1	4	15	4.93	13.4
2113	0	0	0	1	9.9	3.22	9.8
2114	0	0	0	3	23.7	4.38	12.1
2115	0	0	1	0	8.1	4.79	16.1
2116	0	0	1	3	7.7	4.32	11.7
2117	0	0	0	4	32.5	4.62	12.5
2118	0	0	1	0	6.1	4.86	12.9
2119	0	0	1	0	11.7	3.37	10.6
2120	0	0	1	0	7.3	3.81	10.4

2121	0	0	1	2	11	4.76	12.7
2122	0	0	1	0	11.7	4.34	11.6
2123	0	0	1	3	12.3	4.89	13.3
2124	0	0	0	4	9.3	3.97	10.9
2125	0	0	0	3	28.1	4.3	11.8
2126	0	0	1	3	8.7	4.58	12.6
2127	0	0	0	2	12	3.81	10.2
2128	0	0	1	4	17.1	4.68	12.5
2129	0	0	0	1	4.1	4.06	10
2130	0	0	0	0	13.7	4.05	12.8
2131	0	0	0	4	10.2	4.43	12.1
2132	0	0	1	1	15.3	4.69	12.1
2133	0	0	1	0	9.4	3.3	10.2
2134	0	0	0	1	11.5	5.01	11.3
2135	0	0	1	4	9.3	4.37	12.2
2136	0	0	1	1	13.7	4.69	12.8
2137	0	0	1	3	15	4.67	12.3
2138	0	0	0	2	7.1	3.64	10.1
2139	0	0	0	4	7.6	4.36	12.1
2140	0	0	1	1	6.4	5.24	13.3
2141	0	0	1	4	7.6	5.34	12
2142	0	0	0	3	4.8	4.15	10.6
2143	0	0	0	1	26.8	4.65	12.3
2144	0	0	1	1	5.3	5.23	14.1
2145	0	0	1	2	13.5	4.67	12.4
2146	0	0	1	2	2.1	4.58	12.8
2147	0	0	1	1	28.8	4.08	10.3
2148	0	0	1	2	4.9	3.98	9.7
2149	0	0	1	1	29.8	4.87	12.3
2150	0	0	1	2	5.7	4.68	12.7
2151	0	0	0	1	7	4.14	10.7
2152	0	0	1	4	8	4.91	12.4
2153	0	0	0	4	6.1	3.72	11
2154	0	0	1	3	14.6	4.8	13.6
2155	0	0	0	1	6.2	4.45	12.5
2156	0	0	0	2	19.7	4.36	11.7
2157	0	0	1	1	5.8	4.02	10.8
2158	0	0	1	1	4	3.75	10.3
2159	0	0	1	1	5.5	3.69	10.3
2160	0	0	1	1	19	4.26	10.8
2161	0	0	1	1	11.8	5.26	13.5
2162	0	0	1	1	17.8	4.85	12.2
2163	0	0	0	4	7.5	4.53	13.4
2164	0	0	0	2	5.8	4.96	13.3
2165	0	0	0	1	9.9	5.39	13.5
2166	0	0	0	3	13.3	4.09	11.8

2167	0	0	0	1	14.7	4.37	11.9
2168	0	0	1	0	10.9	3.48	11.9
2169	0	0	0	4	10.7	4.3	11.2
2170	0	0	0	4	18.6	5.14	13.6
2171	0	0	1	4	10.2	4.84	13.3
2172	0	0	1	4	6.5	4.02	11.4
2173	0	0	0	4	13	4.53	12
2174	0	0	1	0	15.3	3.11	9.4
2175	0	0	0	1	20.3	4.34	11.4
2177	0	0	1	2	17.3	4.38	11.3
2178	0	0	1	1	13.6	3.49	9.7
2179	0	0	1	4	10.4	4.38	12
2180	0	0	0	4	2.6	3.68	10.5
2181	0	0	1	1	1.9	3.91	10.4
2182	0	0	1	2	15.9	4.41	12
2183	0	0	0	4	6.8	4.17	11.2
2184	0	0	1	1	17.9	4.61	11.2
2185	0	0	1	1	22.3	4.44	12.1
2186	0	0	1	2	7.9	4.24	11.4
2187	0	0	1	4	8.1	4.01	10.9
2188	0	0	1	3	19	4.36	11.3
2189	0	0	1	0	10.7	4.31	11.6
2190	0	0	1	1	13.8	4.92	12.6
2191	0	0	1	4	15	4.11	11.1
2192	0	0	1	2	13.3	4.26	11.6
2193	0	0	0	1	6.1	4.08	10.8
2194	0	0	1	0	17.5	3.2	11.3
2195	0	0	0	1	18.1	5.08	13.3
2196	0	0	0	4	7.6	4.44	12.1
2197	0	0	0	1	10.7	4.47	11.9
2198	0	0	0	0	7.2	3.08	9.6
2199	0	0	0	3	10.1	3.89	10.7
2200	0	0	1	4	14.7	4.22	11.5
2201	0	0	0	4	6.6	4.37	12.1
2202	0	0	0	1	31.1	4.41	12.4
2203	0	0	1	4	15.3	4.27	11.1
2204	0	0	1	1	24	3.8	10.2
2205	0	0	1	1	24.2	4.72	11.7
2206	0	0	1	3	11.1	5.27	13.9
2207	0	0	1	4	13.8	4.82	12.6
2208	0	0	1	3	16.9	4.49	12.6
2209	0	0	1	0	11.4	3.67	9.7
2210	0	0	1	3	4	4.21	12.1
2211	0	0	0	4	10	3.95	10.5
2212	0	0	0	1	12.6	4.21	10.2
2213	0	0	1	2	18.3	4.8	11.7

2214	0	0	1	4	18.5	4.67	12
2215	0	0	0	1	7.5	4.4	12
2216	0	0	0	1	9.8	4.67	9.6
2217	0	0	0	1	16	4.1	9.6
2218	0	0	1	4	5.9	4.56	12.4
2220	0	0	1	4	10.5	4.45	12.4
2221	0	0	0	0	14	4.74	12
2222	0	0	1	2	16.8	5.48	12.7
2223	0	0	1	0	12.5	5.42	11.3
2224	0	0	1	1	14.9	4.25	11
2225	0	0	0	0	12.6	4.24	10.6
2226	0	0	0	1	9.5	4.74	12.4
2227	0	0	1	2	25	4.61	12
2228	0	0	0	4	14	4.68	12.4
2229	0	0	1	4	8	4.71	11.9
2230	0	0	1	0	5.2	3.09	9.5
2231	0	0	1	0	9.3	3.6	9.8
2232	0	0	1	0	8	3.57	12
2233	0	0	1	1	12.8	4.56	12.1
2234	0	0	1	1	10.4	4.49	12.1
2235	0	0	1	0	10.1	2.86	9.1
2236	0	0	0	0	11.2	3.32	10.2
2237	0	0	0	1	15.2	3.72	10.2
2238	0	0	1	3	5.9	4.83	11
2239	0	0	1	2	28.1	4.31	11.3
2240	0	0	1	0	3.6	3.87	10.4
2241	0	0	0	1	11.9	3.84	10.6
2242	0	0	1	1	8	4.88	11.8
2243	0	0	1	4	8.4	4.82	12.8
2244	0	0	0	0	11.8	3.65	11.9
2245	0	0	1	1	19.3	5.15	14.1
2246	0	0	1	0	8.6	3.69	10.4
2247	0	0	1	4	15.6	4.96	12.9
2248	0	0	0	4	16.7	4.34	11.7
2249	0	0	0	4	6.5	4.35	11.5
2250	0	0	0	0	15.3	5.07	18
2251	0	0	0	4	15.8	4.39	12.8
2252	0	0	1	0	14.1	3.59	10.1
2253	0	0	0	1	7	4.47	12.2
2254	0	0	0	0	5.2	3.47	11.2
2255	0	0	1	0	15.5	4.19	11.8
2256	0	0	0	2	9.4	4.37	11.5
2257	0	0	1	2	11	4.57	11.6
2258	0	0	1	1	6.6	4.71	11.9
2259	0	0	1	2	7.6	4.67	12.7
2260	0	0	1	4	17.4	4.5	12.1

2261	0	0	0	1	13.2	3.56	10
2262	0	0	1	0	8.5	3.54	10.2
2263	0	0	1	3	10.8	4.49	12.4
2264	0	0	0	0	23.3	4.56	12.2
2265	0	0	1	0	9.6	5.32	14
2266	0	0	1	1	4.2	4.93	12.7
2267	0	0	0	4	9.6	4.8	12.9
2268	0	0	0	0	13.7	4.15	11.8
2269	0	0	0	0	18.4	3.72	11.2
2270	0	0	1	0	4.8	4.41	12
2271	0	0	1	1	10.5	4.02	10.7
2272	0	0	0	3	6.3	4.53	12.3
2273	0	0	1	0	11.7	4.47	14.2
2274	0	0	1	0	13.8	4.74	10.3
2275	0	0	0	2	5.9	4.86	13.5
2276	0	0	0	3	15.2	4.35	11.6
2277	0	0	1	1	9.9	4.33	11.8
2278	0	0	0	1	10	4.37	11.7
2279	0	0	1	2	10.5	4.43	11.2
2280	0	0	0	1	17.2	4.84	9.8
2281	0	0	1	0	12.4	5.15	17.5
2282	0	0	1	1	10.3	4.41	8.8
2283	0	0	0	1	24.9	4.51	12.3
2284	0	0	0	2	19.2	4.39	11.8
2285	0	0	0	1	7.5	4.3	11.5
2286	0	0	1	1	3.7	4.8	12
2287	0	0	1	1	9.9	4.3	11.8
2288	0	0	1	2	16.3	4.6	12.2
2289	0	0	1	3	3.9	4.23	11.5
2290	0	0	0	0	9.8	3.93	14.2
2291	0	0	1	3	11.9	4.55	12
2292	0	0	0	1	20.4	4.43	11.3
2293	0	0	0	2	17.4	4.25	11.9
2294	0	0	1	0	25.8	4.33	11.1
2295	0	0	1	3	15.6	4.7	12.1
2296	0	0	0	4	8.5	4.23	11.7
2297	0	0	1	1	12.8	4.34	11.3
2298	0	0	1	4	8.4	4.63	12.1
2299	0	0	1	1	12.2	4.57	12.4
2300	0	0	0	2	26.4	3.3	9.4
2301	0	0	0	4	8.2	3.9	10.6
2302	0	0	1	1	12.7	4.78	12
2303	0	0	1	3	16.8	4.89	13.1
2304	0	0	0	0	3.3	5.07	16.5
2305	0	0	1	0	15.4	3.33	10.2
2306	0	0	1	3	4.6	4.36	12.2

2307	0	0	1	0	32.3	3.76	9.9
2308	0	0	0	1	17.4	4.19	10.6
2309	0	0	1	0	7	3.9	10.5
2310	0	0	1	0	12.9	4.11	14
2311	0	0	1	1	7.3	5.05	12.3
2312	0	0	0	4	9.2	4.5	12.6
2313	0	0	0	0	4.9	3.38	11
2314	0	0	1	4	22	4.34	11.4
2315	0	0	1	0	11.7	3.71	10.5
2316	0	0	0	2	24.1	4.5	11.5
2317	0	0	1	1	21.2	4.12	10.2
2318	0	0	0	0	18.5	3.75	10.5
2319	0	0	0	1	11.5	4.86	12.6
2320	0	0	1	0	5.9	5	16.9
2321	0	0	0	1	5.4	4.03	11.2
2322	0	0	0	4	8.1	4.21	11.7
2323	0	0	0	0	15.2	4.22	11.7
2324	0	0	0	4	6	4.3	11.3
2325	0	0	1	4	19.8	4.22	10.8
2326	0	0	1	1	12.4	4.16	11.5
2327	0	0	1	0	7	3.76	11
2328	0	0	1	3	18.4	4.43	12
2329	0	0	1	4	12.3	5.15	13.6
2330	0	0	0	4	7.1	4.49	11.4
2331	0	0	1	3	20.5	4.49	11.9
2332	0	0	0	0	16.1	3.57	11.6
2333	0	0	0	0	8.1	4.87	13.1
2334	0	0	1	4	7.1	4.57	12.2
2335	0	0	1	4	4.8	4.47	12
2336	0	0	1	0	18.3	3.49	10.9
2337	0	0	0	0	12.2	4.81	12.6
2338	0	0	0	0	8.1	3.69	10
2339	0	0	0	2	8.8	4.91	12.8
2340	0	0	1	4	8.4	4.27	12.1
2341	0	0	0	1	12.5	4.37	10.5
2342	0	0	1	3	8.6	4.54	11.3
2343	0	0	0	4	19.1	4.39	13
2344	0	0	1	2	4.1	4.38	11.7
2345	0	0	1	1	11.3	4.71	12.2
2346	0	0	0	4	6.7	5.22	11.9
2347	0	0	1	1	9.1	4.14	11.3
2348	0	0	0	1	19.9	3.94	10.5
2349	0	0	0	2	12.6	4.67	12.1
2350	0	0	1	1	7.2	2.92	9.1
2351	0	0	0	4	17.2	4.31	11.8
2352	0	0	0	4	14.1	4.74	12.7

2353	0	0	1	1	9.7	3.38	10.9
2354	0	0	1	3	30.4	4.51	11.7
2355	0	0	0	3	11.2	4.48	11.2
2356	0	0	1	2	15.6	4.24	11.4
2357	0	0	1	3	3.8	4.35	11.3
2358	0	0	0	1	6	4.26	11.1
2359	0	0	0	0	9.1	3.77	12.2
2360	0	0	0	2	13	4.28	11.8
2361	0	0	1	3	17.8	4.61	12.2
2362	0	0	1	0	22	3.88	10.5
2363	0	0	1	2	3.4	4.54	12.2
2364	0	0	1	0	8.8	3.81	11.5
2365	0	0	0	2	9.2	4.74	13
2366	0	0	0	1	9.6	3.55	10.9
2367	0	0	0	1	6.7	4.36	11.2
2368	0	0	0	1	21.2	3.9	10.6
2369	0	0	0	1	7.8	4.16	11.1
2370	0	0	1	0	12.3	3.04	9.5
2371	0	0	1	3	9.2	4.39	11.7
2372	0	0	1	1	17.8	3.62	9.7
2373	0	0	0	4	27.1	4.23	12.1
2374	0	0	0	3	6	4.93	12
2375	0	0	0	4	3.5	4.64	14.2
2376	0	0	0	0	14.6	3.95	12.6
2377	0	0	1	1	3.6	4.65	11.6
2378	0	0	1	1	8.2	4.3	10.3
2379	0	0	1	1	4.2	4.09	10.7
2380	0	0	1	1	15	4.16	10.2
2381	0	0	1	1	5.9	4.64	11.6
2382	0	0	1	3	13.1	3.95	6.9
2383	0	0	1	1	4.3	4.91	12.1
2384	0	0	0	1	8.3	4.39	12
2385	0	0	0	1	12	4.33	12.4
2386	0	0	1	4	13.4	4.7	13
2387	0	0	1	0	8.6	3.33	9.8
2388	0	0	1	3	16.5	4.58	12.7
2389	0	0	1	4	10.8	4.86	12.6
2390	0	0	1	0	9.2	3.35	10.6
2391	0	0	0	0	7.8	3.34	10.6
2392	0	0	1	1	6.3	4.25	11.8
2393	0	0	1	4	19.3	4.83	13
2394	0	0	1	1	9.5	4.34	11.6
2395	0	0	0	4	10.6	4.15	11.8
2396	0	0	0	4	23	4.38	12.6
2397	0	0	1	0	21.4	4.55	11.4
2398	0	0	1	3	6	4	10.8

2399	0	0	0	0	11.2	2.9	8.6
2400	0	0	1	3	8.2	4.55	11.9
2401	0	0	1	4	5	4.59	12.6
2402	0	0	1	1	21.7	5.16	11.8
2403	0	0	0	0	16.3	3.9	12.9
2404	0	0	0	2	11.5	5.11	13.8
2405	0	0	1	3	7.6	4.41	12.3
2406	0	0	0	1	10.6	3.94	11
2407	0	0	0	2	7.9	4	10.9
2408	0	0	0	4	14.4	4.89	13.3
2409	0	0	1	1	11	4.49	12.4
2410	0	0	0	4	9.7	4.57	12.4
2411	0	0	0	1	4	4.75	12.2
2412	0	0	1	4	12.8	4.39	11.8
2413	0	0	1	0	28.5	4.52	11.9
2414	0	0	1	0	10.1	4.13	10.8
2415	0	0	1	0	24	4.4	13.7
2416	0	0	0	0	17.6	4.02	10.7
2417	0	0	1	2	12.1	4.49	12.1
2418	0	0	1	1	9.5	4.24	12
2419	0	0	1	3	13.3	4.1	11.6
2420	0	0	1	2	12.3	4.46	12.1
2421	0	0	1	0	11.9	4.09	12.7
2422	0	0	1	0	7.1	4.33	11.1
2423	0	0	0	1	4.8	4.48	11.7
2424	0	0	1	1	16.3	3.91	9.4
2425	0	0	1	2	6.2	5.23	13.8
2426	0	0	1	3	22.8	4.97	12.6
2427	0	0	0	1	3.4	3.52	8.8
2428	0	0	1	2	8.9	4.42	12
2429	0	0	0	3	7.1	4.16	11.7
2430	0	0	1	4	7.7	4.52	12.7
2431	0	0	0	1	10.9	4.63	12
2432	0	0	1	0	5.2	2.97	9.3
2433	0	0	0	0	11.5	3.81	12
2434	0	0	1	4	7.3	4.62	13
2435	0	0	1	3	20.6	4.3	11.7
2436	0	0	0	4	11.5	4.56	13
2437	0	0	0	0	4.6	3.03	10.5
2438	0	0	1	1	4.9	4.56	10.7
2439	0	0	1	0	19.9	4.25	9.9
2440	0	0	1	3	16.2	3.97	12.7
2441	0	0	1	2	17.9	4.88	12.4
2442	0	0	1	0	8.3	2.42	8.1
2443	0	0	1	0	7.3	3.28	9.4
2444	0	0	1	1	8.8	5.35	12.6

2445	0	0	1	4	6.1	3.95	11.5
2446	0	0	1	1	13.2	4.21	11.5
2447	0	0	1	4	4.7	4.94	13.1
2448	0	0	1	3	11.6	4.53	12.5
2449	0	0	1	4	5.7	4.4	11.7
2450	0	0	1	0	18.3	3.56	10.6
2451	0	0	1	2	7.9	4.81	13.3
2452	0	0	1	2	9.9	4.75	13
2453	0	0	1	0	13.8	3.61	11
2454	0	0	1	4	6.2	4.09	11.7
2455	0	0	1	0	9.2	4.01	11
2456	0	0	1	0	18.4	4.56	12.7
2457	0	0	1	1	10.2	4.88	11.9
2458	0	0	0	2	12.7	4.49	12.5
2459	0	0	0	4	10.7	3.86	10.8
2460	0	0	1	0	4.7	3.66	12.5
2461	0	0	1	0	11	4.54	12.2
2462	0	0	0	1	22.5	4.69	12.2
2463	0	0	1	3	5.6	4.54	11.7
2464	0	0	0	3	6.5	4.75	12.8
2465	0	0	1	1	8.6	4.99	12.9
2466	0	0	1	4	10.4	4.65	12.2
2467	0	0	0	4	11	4.17	11.2
2468	0	0	0	3	12.2	3.81	11.8
2469	0	0	0	3	30.4	4.67	13.6
2470	0	0	0	0	8.7	4.47	12.4
2471	0	0	1	4	14.3	4.46	12.3
2472	0	0	1	1	16.1	4.4	11.8
2473	0	0	0	4	10.8	3.98	10.6
2474	0	0	1	3	11.3	4.48	12
2475	0	0	0	3	10.8	4.05	11.6
2476	0	0	0	3	17.4	5.13	13.3
2477	0	0	0	4	14.7	4.32	11.6
2478	0	0	0	0	9	4.38	14.6
2479	0	0	1	4	9.6	5.35	13.7
2480	0	0	1	3	6.9	4.79	12.4
2481	0	0	0	3	26.9	4.83	12.2
2482	0	0	0	3	6.6	4.6	13
2483	0	0	0	0	19.1	4.63	15.5
2484	0	0	1	3	2.9	4.45	12.1
2485	0	0	1	0	16.4	4.16	13.4
2486	0	0	0	2	5.8	4.36	12.1
2487	0	0	1	1	7.6	4.57	12.1
2488	0	0	1	4	11.3	4.28	11.2
2489	0	0	0	1	10.7	4.67	12.3
2490	0	0	0	1	7.3	4.22	11.2

2491	0	0	0	1	2	4.13	11.4
2492	0	0	0	3	9.6	3.82	10.4
2493	0	0	0	0	13.6	3.89	13.5
2494	0	0	0	1	13.4	4.12	11.4
2495	0	0	1	0	19.3	4.26	10.4
2496	0	0	0	4	10.9	4.12	11.7
2497	0	0	0	3	19.3	4.84	13.5
2498	0	0	0	4	4.2	4.08	11.1
2499	0	0	1	1	3.9	4.47	13.2
2500	0	0	0	0	16.2	3.06	9.6
2501	0	0	0	0	17.7	5.02	13.5
2502	0	0	1	3	7.6	5	14.7
2503	0	0	1	4	14.8	4.62	13
2504	0	0	1	1	7.2	4.25	11.5
2505	0	0	1	4	8.2	4.29	11.8
2506	0	0	1	1	8.8	4.21	10.1
2507	0	0	1	0	16.2	4.49	10.9
2508	0	0	0	3	4.1	4.82	13.6
2509	0	0	0	0	14.5	4.47	15.6
2510	0	0	0	1	4.7	4.38	12.1
2511	0	0	0	3	17.1	4.77	13.3
2512	0	0	1	4	14.6	4.72	12.6
2513	0	0	0	4	3.3	4.73	13
2514	0	0	1	1	8.7	4.17	10.6
2515	0	0	0	3	20.2	4.52	11.9
2516	0	0	0	3	11.3	4.41	11.5
2517	0	0	0	3	10.4	4.53	12.6
2518	0	0	1	1	21.5	4.95	12.5
2519	0	0	0	0	11.6	3.19	10.2
2520	0	0	1	4	13.5	4.13	11.4
2521	0	0	1	2	11.4	5.07	13.2
2522	0	0	1	4	15.1	5.15	13.4
2523	0	0	0	0	11.6	4.39	15.4
2524	0	0	1	1	15.3	5.37	13.8
2525	0	0	1	0	15	2.89	9.4
2526	0	0	0	4	12.2	4.74	11.4
2527	0	0	1	1	4.2	4.69	12
2528	0	0	1	1	13.4	4.62	11.9
2529	0	0	1	0	11.8	4.09	10.3
2530	0	0	1	1	13.4	4.42	13
2531	0	0	0	4	35.6	4.06	10.7
2532	0	0	0	0	10.7	3.68	11.9
2533	0	0	1	0	3.9	4.37	11.4
2534	0	0	1	4	8.1	4.63	12.1
2535	0	0	1	4	10	4.76	12.6
2536	0	0	1	4	19.4	4.91	13.3

2537	0	0	0	0	9.3	2.92	10.3
2538	0	0	1	0	13.1	4.71	12.5
2539	0	0	0	0	10.5	4.1	14
2540	0	0	0	0	15	4.5	11.4
2541	0	0	1	1	9	4.76	10.5
2542	0	0	1	0	11	3.85	13
2543	0	0	1	4	5.4	4.22	13.1
2544	0	0	0	0	4.5	3.91	11.2
2545	0	0	0	0	8.1	4.32	11.5
2546	0	0	0	4	4.9	4.08	11.6
2547	0	0	1	0	21.7	4.09	11.5
2548	0	0	1	1	7.5	4.23	11.3
2549	0	0	0	3	16.6	3.74	9.6
2550	0	0	1	0	6.1	4.54	11.7
2551	0	0	1	3	11.3	4.07	10.9
2552	0	0	0	1	7.5	4.07	11.3
2553	0	0	0	1	20.1	3.69	8.6
2554	0	0	1	2	11	4.73	12.2
2555	0	0	0	3	7	4.66	11.7
2556	0	0	0	3	11.2	4.52	12.6
2557	0	0	1	4	8.8	4.2	11.9
2558	0	0	0	0	9.7	2.97	10.3
2559	0	0	1	0	21	3.12	10.2
2560	0	0	0	2	9.1	4.91	14.5
2562	0	0	0	2	17	4.29	11.6
2563	0	0	0	3	6.8	5.06	13.1
2564	0	0	1	0	5.6	3.48	10.9
2565	0	0	1	2	6.5	4.89	13.2
2566	0	0	0	0	15.7	4.58	12.4
2567	0	0	1	0	15.2	3.82	10.2
2568	0	0	0	1	9.2	4.91	13.1
2569	0	0	0	4	19.5	4.56	12.4
2570	0	0	1	0	8	4.18	14.3
2571	0	0	0	0	10.6	4.81	16.4
2572	0	0	0	1	3.5	4.63	12.2
2573	0	0	0	3	13.8	4.43	12.8
2574	0	0	0	0	9.1	4.1	11.1
2575	0	0	1	3	5.5	4.76	13.5
2576	0	0	1	2	8.3	4.91	13
2577	0	0	0	4	8.8	4.21	11.3
2578	0	0	0	3	9.5	4.6	12.8
2579	0	0	0	3	5.5	4.43	12.3
2580	0	0	0	0	13.9	3.89	9.8
2581	0	0	0	0	26.7	4.29	11.7
2582	0	0	0	2	25.7	4.77	13.1
2583	0	0	0	4	9.3	4.18	11.7

2584	0	0	1	1	11.2	5.15	13.7
2585	0	0	1	3	7	4.62	12.5
2586	0	0	1	0	10.8	4.77	16.2
2587	0	0	1	0	6.2	3.73	9.7
2588	0	0	1	3	16.2	4.55	12.1
2589	0	0	1	0	18.2	2.98	10.3
2590	0	0	1	3	6.2	4.58	12
2591	0	0	1	3	13	4.44	11.9
2592	0	0	1	0	24.1	4.89	12.4
2593	0	0	0	1	5.2	4.87	12.4
2594	0	0	0	3	7.2	4.74	13
2595	0	0	1	4	18.8	4.45	12.3
2596	0	0	1	4	9.2	4.7	13.3
2597	0	0	1	1	21.9	4.13	10.6
2598	0	0	1	1	8.7	5.09	13.1
2599	0	0	1	0	17.2	3.89	11.2
2600	0	0	0	0	16.9	4.24	11.4
2601	0	0	0	1	13.7	4.26	11.4
2602	0	0	1	0	23.6	4.56	12.2
2603	0	0	0	1	10.5	4.39	11.3
2604	0	0	0	1	10.6	4.61	13.5
2605	0	0	0	3	11.5	4.21	12.4
2606	0	0	1	1	6.5	4.41	11.5
2607	0	0	0	4	9	4.31	12.1
2608	0	0	1	1	11.8	4.26	10.4
2609	0	0	0	0	10.1	3.04	9.5
2610	0	0	0	4	7.5	4.5	11.1
2611	0	0	0	2	21.1	4.43	12.5
2612	0	0	0	3	7.6	4.87	13.2
2613	0	0	1	3	7.3	4.16	11.5
2614	0	0	1	3	7.3	4.87	13.3
2615	0	0	0	3	9.8	4.38	12.6
2616	0	0	1	4	15.2	4.41	11.8
2617	0	0	1	2	16.2	4.75	12.4
2618	0	0	0	2	16.2	4.62	12.2
2619	0	0	1	0	8.2	4	12.7
2620	0	0	1	3	15	5.11	13.5
2621	0	0	1	1	16.3	4.43	11.1
2622	0	0	1	3	5.2	4.57	12.2
2623	0	0	1	3	7.5	4.51	12.3
2624	0	0	1	3	6	4.42	11.6
2625	0	0	1	4	9.4	4.56	12.5
2626	0	0	0	2	8	4.33	11.7
2627	0	0	0	3	24.9	4.24	12.2
2628	0	0	0	2	8.2	4.8	12.5
2629	0	0	1	4	16.3	4.65	13.1

2630	0	0	0	0	6.3	3.59	9.8
2631	0	0	1	4	6.4	4.41	11.5
2632	0	0	1	4	16.6	4.43	12.1
2633	0	0	0	4	17.1	4.53	13.5
2634	0	0	1	2	16.8	4.88	12.3
2635	0	0	1	4	7.8	4.63	12.8
2636	0	0	1	3	8.6	4.26	12.2
2637	0	0	1	0	20.5	3.72	10.9
2638	0	0	1	3	12	4.34	12.2
2639	0	0	1	0	2.4	3.19	9.7
2640	0	0	0	0	6.3	4.22	13.6
2641	0	0	0	0	15.5	4.15	11.7
2642	0	0	1	0	9.4	3.15	10.3
2643	0	0	1	3	16.7	4.76	12.1
2644	0	0	0	0	20.8	4.1	12.1
2645	0	0	0	3	22.1	4.92	13
2646	0	0	1	2	10.8	4.8	12.9
2647	0	0	1	3	9.2	4.89	12.6
2648	0	0	0	1	5.8	4.4	12
2649	0	0	1	1	12.1	4.64	12.6
2650	0	0	1	2	8	4.08	11.1
2651	0	0	1	1	13.8	4.02	11
2652	0	0	0	3	7	4.67	12.6
2653	0	0	0	1	5.6	4.39	12.9
2654	0	0	1	4	7	4.21	11
2655	0	0	1	0	9.6	4.52	13
2656	0	0	0	1	11.3	4.67	12.1
2657	0	0	1	3	15.5	4.72	12.7
2658	0	0	0	0	3.6	4.56	11.4
2659	0	0	1	1	5.9	4.14	11.6
2660	0	0	1	0	8.7	5.53	12.8
2661	0	0	0	1	7.3	4.59	11.5
2662	0	0	0	3	5.4	4.59	11.5
2663	0	0	1	4	8.2	5.68	15.8
2664	0	0	0	3	15.2	4.29	11.8
2665	0	0	1	0	7.3	3.84	10.6
2666	0	0	0	1	20.6	3.91	11.2
2667	0	0	0	1	7.5	4.73	12.4
2668	0	0	0	4	6	4.25	12.3
2669	0	0	1	1	27.8	3.51	10.6
2670	0	0	0	4	16.3	4.27	12.5
2671	0	0	1	4	11.7	4.14	11.1
2672	0	0	0	2	11.9	4.55	12.1
2673	0	0	1	1	23.9	5.11	12.8
2674	0	0	1	4	8.4	4.66	13.5
2675	0	0	0	2	7	4.26	10.9

2676	0	0	1	4	11.2	4.96	13.5
2677	0	0	1	3	14	4.17	12.3
2678	0	0	1	0	7.8	3.46	10.8
2679	0	0	0	0	13.2	4.99	11.9
2680	0	0	1	2	17.9	4.69	11.5
2681	0	0	0	3	19	4.36	11.5
2682	0	0	0	1	12.2	4.47	10.5
2683	0	0	0	3	8.5	4.39	11.9
2684	0	0	0	1	8.7	4.44	12.4
2685	0	0	1	3	10	4.81	12.7
2686	0	0	0	1	7.3	4.1	10.7
2687	0	0	1	2	1.6	3.15	8.9
2688	0	0	0	1	7.9	4.14	10.3
2689	0	0	1	4	10.4	5.07	13.2
2690	0	0	0	1	21.8	5.07	13.4
2691	0	0	0	0	12.6	3.6	10.6
2692	0	0	0	1	11.6	3.66	11.1
2693	0	0	1	3	5.3	4.74	12
2694	0	0	0	1	4.8	3.67	10.9
2695	0	0	1	1	4.5	4.54	12.4
2696	0	0	0	3	10.3	4.27	12.3
2697	0	0	0	1	4.1	3.57	10.8
2698	0	0	1	1	6.1	4.48	11.4
2699	0	0	0	3	8	4.37	11.9
2700	0	0	1	0	6.6	3.68	9.9
2701	0	0	0	4	20.6	4.13	11.2
2702	0	0	0	3	14	4.55	12.1
2703	0	0	1	3	17.9	4.37	11.9
2704	0	0	1	3	5.5	4.68	12
2705	0	0	0	2	12.6	4.48	12.1
2706	0	0	1	0	6.7	3.8	10.9
2707	0	0	1	3	8.6	4.41	11.9
2708	0	0	1	1	12.4	4.06	10.5
2709	0	0	1	1	3.6	4.54	12.1
2710	0	0	1	1	5.8	4.33	11.4
2711	0	0	1	1	10.6	4.28	11.4
2712	0	0	1	2	7.9	5	13.3
2713	0	0	1	3	17.9	5.01	13.2
2714	0	0	0	1	20.3	4.04	10.9
2715	0	0	1	4	8.1	3.98	11.2
2716	0	0	0	1	11.1	4.43	11.5
2717	0	0	0	3	3.4	4.17	11.8
2718	0	0	1	4	6.5	4.38	11.3
2719	0	0	1	0	13.9	3.84	10.8
2720	0	0	1	3	8.1	4.59	11.7
2721	0	0	0	1	14.5	4.58	12.5

2722	0	0	0	3	32.2	4.21	11.4
2723	0	0	1	4	18.5	3.78	10.9
2724	0	0	1	4	13	5.13	14.9
2725	0	0	1	1	17.2	3.59	9.1
2726	0	0	1	1	15.2	5.28	12.2
2727	0	0	0	1	14.5	3.41	10.3
2728	0	0	0	3	4.5	4.6	12.7
2729	0	0	0	3	9.2	3.95	11.5
2730	0	0	0	1	19.3	4.88	12.3
2731	0	0	0	4	4.1	4.18	12.7
2732	0	0	1	3	8.3	4.37	12.7
2733	0	0	1	4	19.5	4.84	13
2734	0	0	1	2	15.2	4.96	13.9
2735	0	0	0	1	24.4	5.27	13.6
2736	0	0	0	1	14.4	3.79	10.2
2737	0	0	0	4	18.6	4.33	12.2
2738	0	0	1	1	11.2	4.4	11
2739	0	0	0	3	13.3	5.15	14.4
2740	0	0	0	1	15.6	4.16	11.5
2741	0	0	1	1	22.3	4.25	11
2742	0	0	1	1	5.9	3.98	11
2743	0	0	1	4	6.1	4.74	13.1
2744	0	0	0	3	5.5	4.4	12.1
2745	0	0	0	2	14.6	4.48	11.5
2746	0	0	0	4	8	4.92	13.3
2747	0	0	1	3	11.6	4.82	10.4
2748	0	0	0	3	10.1	4.74	12.6
2749	0	0	0	3	30.1	4.35	11.6
2750	0	0	0	2	17.6	4.91	13.3
2751	0	0	1	2	17.4	4.75	12.1
2752	0	0	1	1	22.5	3.8	10.8
2753	0	0	1	1	17	4.76	12.9
2754	0	0	0	3	22	4.47	12.5
2755	0	0	0	4	15.7	4.85	14.1
2756	0	0	1	3	18.3	4.71	13.2
2757	0	0	1	4	5.2	5.11	13.9
2758	0	0	1	1	19	4.31	11.5
2759	0	0	0	1	12.6	4.43	11.9
2760	0	0	0	3	25.8	4.4	12.4
2761	0	0	1	1	13.5	5.01	12.4
2762	0	0	1	4	9.7	4.21	11.1
2763	0	0	0	4	30.5	0.96	2.5
2764	0	0	0	3	5.6	4.4	11.7
2765	0	0	0	1	3.7	4.47	12.2
2766	0	0	1	4	15.8	4.43	12.4
2767	0	0	1	1	11.3	4.57	11.7

2768	0	0	0	4	10.8	4.4	11.8
2769	0	0	1	1	12.1	5.4	13.8
2770	0	0	0	4	8.1	3.99	11.2
2771	0	0	1	1	12.7	4.2	12
2772	0	0	0	4	15.5	4.37	11.8
2773	0	0	0	3	19.7	4.29	12.2
2774	0	0	1	4	12.1	4.29	11.2
2775	0	0	1	3	19.1	4.55	11.7
2776	0	0	1	1	10.3	3.34	10
2777	0	0	1	1	6.2	3.92	9.7
2778	0	0	1	1	12.8	4.14	10.5
2779	0	0	0	3	11.2	4.94	13.4
2780	0	0	0	4	10.2	4.32	11.3
2781	0	0	0	3	11.2	4.2	12.1
2782	0	0	0	3	8.2	5.53	14.7
2783	0	0	1	0	9.1	4.74	12.9
2784	0	0	0	0	9.4	4.24	14.5
2785	0	0	1	0	9.2	4.74	12.1
2786	0	0	0	0	11.6	4.62	16.3
2787	0	0	1	0	11.3	4.02	12
2788	0	0	1	4	5.5	4.44	11.8
2789	0	0	1	1	9	3.1	9
2790	0	0	1	1	19.3	4.13	11.1
2791	0	0	0	3	5.8	4.67	12.9
2792	0	0	0	3	14.8	4.41	12.2
2793	0	0	1	1	3.9	4.37	10.9
2794	0	0	1	3	11.6	4.25	11
2795	0	0	1	1	16.6	4.28	11.3
2796	0	0	1	1	8	3.59	9.4
2797	0	0	1	3	8.4	4.33	11.6
2798	0	0	0	2	11.8	5.3	12.7
2799	0	0	1	4	15.3	4.92	13.6
2800	0	0	1	2	11.3	4.43	11.8
2801	0	0	0	4	6.3	4.93	14.2
2802	0	0	1	2	16	4.36	11.3
2803	0	0	1	1	4.7	4.65	12.2
2804	0	0	1	0	24.6	6.08	21.7
2805	0	0	1	1	13.3	3.73	9.4
2806	0	0	1	2	14.7	4.79	12.7
2807	0	0	1	3	11.2	4.78	13.1
2808	0	0	0	2	12.2	4.32	11.4
2809	0	0	0	3	5.4	4.3	11.9
2810	0	0	1	1	6.2	4.1	10.9
2811	0	0	0	4	6.4	4.11	11.5
2812	0	0	1	4	8.6	3.88	10.2
2813	0	0	0	0	18.7	3.67	10.8

2814	0	0	0	3	8.8	4.67	12.7
2815	0	0	0	1	9.2	3.96	11.2
2816	0	0	0	4	5.9	4.27	11.5
2817	0	0	1	1	4.1	4.62	12.3
2818	0	0	0	4	13.9	4.47	12.3
2819	0	0	1	2	13.4	4.76	12.5
2820	0	0	0	3	3.5	4.35	11.7
2821	0	0	1	3	3.1	4.93	10.6
2822	0	0	0	0	10.3	4.67	15.1
2823	0	0	1	0	8.2	3.1	9.6
2824	0	0	0	0	7.9	3.25	10
2825	0	0	1	0	9.8	3.54	10
2826	0	0	0	4	15	4.25	11.8
2827	0	0	1	2	11.2	4.1	12
2828	0	0	1	4	14.3	5.1	14.2
2829	0	0	0	1	12.1	3.65	9.6
2830	0	0	0	3	28.4	5.02	14.4
2831	0	0	0	1	10.1	4.16	11.3
2832	0	0	1	3	6.9	4.94	12.7
2833	0	0	0	1	10.2	4.38	10.8
2834	0	0	0	3	10.1	4.43	12.4
2835	0	0	1	3	14.1	4.9	13.5
2836	0	0	0	3	25.7	4.97	13.4
2837	0	0	0	1	18.6	4.69	12.7
2838	0	0	1	4	8.7	4.8	12.5
2839	0	0	1	3	7.6	4.48	12.9
2840	0	0	1	1	12.6	3.82	10.8
2841	0	0	1	2	15.4	4.63	12.2
2842	0	0	1	3	7	4.68	12.6
2843	0	0	0	3	14.4	4.26	12.1
2844	0	0	1	3	11.8	4.97	12.7
2845	0	0	1	0	14.9	3.62	11.6
2846	0	0	1	4	6.1	4.61	12.4
2847	0	0	0	2	12.4	4.09	10.9
2848	0	0	1	1	5.6	4.21	10.5
2849	0	0	0	0	9.5	3.92	11.8
2850	0	0	1	0	10.6	4.66	11
2851	0	0	0	1	6	4.73	12.8
2852	0	0	1	3	11.5	5.08	12.8
2853	0	0	1	0	7	4.64	14.8
2854	0	0	0	1	5	4.63	12.4
2855	0	0	0	4	14.4	4.41	12.1
2856	0	0	0	3	8.7	4.66	12.5
2857	0	0	0	1	18.9	4.62	11.9
2858	0	0	0	0	13.8	4.4	14.7
2859	0	0	0	3	16.8	5.27	11.1

2860	0	0	1	1	26.1	4	11.8
2861	0	0	0	2	4.4	3.92	11.8
2862	0	0	0	1	15.2	3.55	9.5
2863	0	0	0	4	12.6	4.36	12
2864	0	0	1	3	12.2	4.53	12.9
2865	0	0	1	2	5.9	4.77	12.4
2866	0	0	0	4	15.2	4.16	11.7
2867	0	0	1	4	6.1	4.87	13.1
2868	0	0	1	3	9.4	4.73	12.5
2869	0	0	1	2	8.1	4.43	11.6
2870	0	0	0	1	13.4	3.99	10.9
2871	0	0	1	4	13.8	4.22	12.2
2872	0	0	0	4	7.2	4.2	12.1
2873	0	0	1	3	5.6	3.96	10.6
2874	0	0	1	0	12.7	3.73	10.1
2875	0	0	1	3	7.3	4.25	11.8
2876	0	0	0	1	24	3.98	11.2
2877	0	0	1	1	25.1	4.93	12.5
2878	0	0	1	2	5.3	4.55	12.9
2879	0	0	0	0	7.2	3.84	10.7
2880	0	0	1	3	6.3	4.43	11.2
2881	0	0	0	4	4.8	4.97	13.1
2882	0	0	1	3	4.6	4.28	12.1
2883	0	0	1	0	18.4	3.15	10.2
2884	0	0	0	1	20.3	5.02	13.4
2885	0	0	1	1	5	4.2	11.2
2886	0	0	1	0	20.2	3.75	10.8
2887	0	0	0	1	21.8	4.55	12.6
2888	0	0	0	1	13.6	4.21	10.7
2889	0	0	0	1	6.5	4.66	12.9
2890	0	0	0	0	8.8	3.55	10.1
2891	0	0	0	4	6.6	4.76	12.9
2892	0	0	1	3	5.3	5.41	12.8
2893	0	0	0	3	7.8	4.3	11.4
2894	0	0	0	3	3.7	4.58	12.1
2895	0	0	1	0	16.2	4.64	11.6
2896	0	0	0	2	17.8	4.86	12.9
2897	0	0	1	1	16.3	5.05	13
2898	0	0	1	1	4.2	4.28	11.1
2899	0	0	0	0	6.3	3.67	10.1
2900	0	0	1	0	10.5	3.43	11.7
2901	0	0	1	4	3.5	4.59	12
2902	0	0	0	3	7.6	5.05	13.1
2903	0	0	0	3	16.2	4.53	12.1
2904	0	0	1	1	16.7	4.29	11.7
2905	0	0	0	0	14.1	3.79	10.3

2906	0	0	1	3	7.2	4.42	12.2
2907	0	0	1	1	19.6	3.86	10.6
2908	0	0	1	1	11.1	4.5	11.8
2909	0	0	0	1	13.4	4.98	12.9
2910	0	0	0	4	13.9	4.7	13
2911	0	0	0	3	9.5	4.13	11.4
2912	0	0	0	4	9.8	4.53	12.5
2913	0	0	0	3	9.4	4.39	12.3
2914	0	0	0	1	7.2	4.12	11.2
2915	0	0	1	3	7.3	4.64	12.7
2916	0	0	0	4	6.1	4.98	13.1
2917	0	0	0	3	8.1	4.63	13.1
2918	0	0	1	2	9.9	4.91	11.6
2919	0	0	0	2	19.2	4.89	13.5
2920	0	0	1	2	17.1	4.21	11
2921	0	0	0	1	4.8	4.9	13
2922	0	0	0	3	6	4.21	11.9
2923	0	0	1	1	20.4	4.06	11.5
2924	0	0	1	3	15.1	4.65	12.6
2925	0	0	0	3	9.1	4.1	11.4
2926	0	0	0	2	5.2	4.32	12.3
2927	0	0	1	1	13.2	4.62	10.6
2928	0	0	1	3	7.5	4.45	11.9
2929	0	0	1	3	13.3	4.38	12.6
2930	0	0	0	0	18.2	3.53	10.7
2931	0	0	1	0	24.4	4.46	11.6
2932	0	0	1	4	6	4.1	10.6
2933	0	0	0	3	8.2	4.38	11.6
2934	0	0	1	0	4	4.51	12.2
2935	0	0	0	4	4.5	4.51	12
2936	0	0	0	2	5.4	3.98	10.6
2937	0	0	0	1	6	4.42	11.8
2938	0	0	0	4	10.4	4.43	12.7
2939	0	0	1	3	12.3	4.73	12.9
2940	0	0	1	3	16.4	4.87	13.6
2941	0	0	0	4	19.6	5.18	13.9
2942	0	0	0	2	11.1	4.7	13
2943	0	0	1	2	14.9	4.25	11.6
2944	0	0	1	3	17.1	4.4	11.6
2945	0	0	0	3	7.1	4.56	12
2946	0	0	1	2	30	4.57	12.5
2947	0	0	0	2	5.5	4.48	11.9
2948	0	0	1	3	6.5	4.5	11.7
2949	0	0	1	3	10.5	4.54	11.3
2950	0	0	0	2	5.3	4.74	13.2
2951	0	0	0	4	12.6	5.22	14.2

2952	0	0	1	4	21.2	4.64	12.4
2953	0	0	1	4	14.8	4.24	11.9
2954	0	0	1	0	7.5	4.04	10.6
2955	0	0	0	2	7.6	4.66	12.8
2956	0	0	1	3	26.5	4.21	11.9
2957	0	0	1	4	14.9	4.3	11.6
2958	0	0	1	2	6.9	4.21	11.8
2959	0	0	0	2	14	3.69	9.6
2960	0	0	1	0	11.1	4.26	10.5
2961	0	0	0	2	12.8	4.61	12.5
2962	0	0	1	0	12.3	4.9	17.6
2963	0	0	0	1	12.7	4.42	11.8
2964	0	0	0	1	28.1	4.47	11.7
2965	0	0	0	3	8.6	4.41	12.7
2966	0	0	1	4	17.3	4.86	13.4
2967	0	0	1	2	6	4.83	13.1
2968	0	0	1	2	5.6	4.32	11.4
2969	0	0	0	4	17.1	4.57	12.1
2970	0	0	1	3	13.9	5.11	14.1
2971	0	0	0	4	15.5	4.69	11.9
2972	0	0	0	2	18.8	4.47	12
2973	0	0	0	2	9.8	5.08	13.3
2974	0	0	1	2	13.3	4.22	11.5
2975	0	0	1	2	5	4.64	11.7
2976	0	0	0	3	13.3	4.66	11.8
2977	0	0	1	2	13.9	4.65	12.3
2978	0	0	0	1	24.1	4.94	12.6
2979	0	0	1	4	8.7	4.15	11.2
2980	0	0	0	0	23.6	2.54	8.2
2981	0	0	1	3	11	3.94	11.5
2982	0	0	1	1	8.3	4.35	11.5
2983	0	0	0	2	9.4	4.49	12.5
2984	0	0	0	3	10.4	4.32	11.6
2985	0	0	1	2	4.1	4.13	11.1
2986	0	0	1	3	10.1	4.73	12.4
2987	0	0	1	4	13.9	4.22	10.4
2988	0	0	1	2	13.9	5.52	14.7
2989	0	0	0	2	6.6	4.68	12.6
2990	0	0	1	4	9.2	4.72	12.8
2991	0	0	0	3	4.6	4.46	12.4
2992	0	0	0	3	7.4	4.34	11.7
2993	0	0	1	4	11.8	4.46	12.3
2994	0	0	1	2	9.4	4.57	12.5
2995	0	0	1	1	13	4.46	11.2
2996	0	0	1	3	5.1	4.49	13
2997	0	0	1	0	11.3	4.71	11.8

2998	0	0	1	1	8.8	4.55	12.3
2999	0	0	0	2	9.2	4.62	12
3000	0	0	0	1	6.6	4.6	11.6
3001	0	0	1	2	11.2	4.77	13
3002	0	0	1	3	10.7	4.56	10.6
3003	0	0	1	3	14.5	4.68	12.5
3004	0	0	1	1	6.6	2.99	9.5
3005	0	0	0	1	23.8	3.92	10.4
3006	0	0	1	3	4.5	4.93	13.4
3007	0	0	1	4	16.3	4.67	11.9
3008	0	0	1	2	8.7	4.68	12.8
3009	0	0	1	4	4.9	4.82	13.8
3010	0	0	0	3	13.5	4.76	13
3011	0	0	1	2	5.8	4.43	12
3012	0	0	0	4	13.6	3.91	10.9
3013	0	0	0	4	12.6	4.36	12.4
3014	0	0	0	4	14.1	4.36	12
3015	0	0	1	3	9	4.42	11.7
3016	0	0	1	3	5.7	4.5	11.2
3017	0	0	0	4	7.1	4.08	11
3018	0	0	0	3	18.3	5	13.1
3019	0	0	0	4	7.2	4.44	12.3
3020	0	0	1	1	9.8	4.47	12.4
3021	0	0	1	3	16.1	4.51	12.2
3022	0	0	1	0	10.4	3.72	11.5
3023	0	0	1	4	16.5	4.5	12
3024	0	0	0	2	9.4	5.41	11
3025	0	0	1	3	11.7	4.92	12.8
3026	0	0	0	4	13.8	4.62	12.7
3027	0	0	1	3	7.3	4.17	11.2
3028	0	0	1	1	11.8	4.62	12.3
3029	0	0	0	1	19.1	4.29	10.1
3030	0	0	1	3	8.9	4.54	12.4
3031	0	0	0	1	12.8	4.47	12
3032	0	0	0	3	6.8	4.47	12.2
3033	0	0	0	0	6.1	3.06	9.1
3034	0	0	1	3	9	4.83	11.7
3035	0	0	0	2	15.2	4.69	12.2
3036	0	0	0	2	8.4	4.94	13
3037	0	0	1	0	20.3	3.69	10.2
3038	0	0	1	0	8	3.43	11.5
3039	0	0	0	1	13	5	13
3040	0	0	1	4	15.1	3.95	11
3041	0	0	0	4	9	5.2	13.8
3042	0	0	0	3	5.9	4.32	11.3
3043	0	0	0	2	5.9	4.38	12

3044	0	0	1	4	8.1	4.36	12.1
3045	0	0	1	3	5.4	4.26	11.6
3046	0	0	0	0	10.9	4.66	13.2
3047	0	0	1	0	8	4.19	16.5
3048	0	0	0	3	3.8	4.26	11.6
3049	0	0	1	3	2.5	4.64	12.2
3050	0	0	1	2	7.5	4.45	11.4
3051	0	0	1	3	4.4	3.28	10.3
3052	0	0	0	3	5.4	4.32	12
3053	0	0	1	1	10.9	4.24	11.5
3054	0	0	1	3	5.2	4.51	12.8
3055	0	0	1	3	7.3	4.35	11.6
3056	0	0	0	2	19.3	4.86	13
3057	0	0	0	1	9.1	4	11
3058	0	0	0	4	15.6	4.59	12.4
3059	0	0	1	3	11	4.62	12.5
3060	0	0	0	2	6.6	4.12	11.9
3061	0	0	1	3	16.8	4.35	10.5
3062	0	0	0	3	17.2	4.36	12.6
3063	0	0	1	3	9.2	3.74	10.6
3064	0	0	0	2	11.6	4.5	11.8
3065	0	0	0	2	11.7	4.24	11.8
3066	0	0	0	3	6.5	4.74	13.1
3067	0	0	0	2	11.3	4.64	11.9
3068	0	0	1	3	5.2	4.09	10.8
3069	0	0	0	4	4.7	4.79	13.2
3070	0	0	0	4	11.9	5.24	14.6
3071	0	0	0	2	6.9	4.78	12.9
3072	0	0	0	4	3.7	4.28	12.4
3073	0	0	0	4	6.6	4.88	10.1
3074	0	0	0	2	5.6	4.22	11.8
3075	0	0	0	3	16	4.26	11.5
3076	0	0	1	3	7.9	4.73	13.3
3077	0	0	0	1	4.6	4.72	12.4
3078	0	0	0	2	10	4.66	13.5
3079	0	0	0	3	5.9	4.44	12.4
3080	0	0	0	2	11.5	5.19	13.6
3081	0	0	0	3	6.6	4.23	11.1
3082	0	0	1	3	14.8	4.99	13.7
3083	0	0	1	3	10.8	5.04	13.7
3084	0	0	0	1	8.1	3.78	10.9
3085	0	0	0	1	8.4	4.03	11.6
3086	0	0	1	2	5.9	4.46	12.1
3087	0	0	1	4	13.5	4.17	11.5
3088	0	0	1	2	12.8	4.45	12.3
3089	0	0	0	4	22.5	4.77	11.6

3090	0	0	1	3	11	4.8	12.2
3091	0	0	1	3	5.1	4.76	13.2
3092	0	0	0	2	8	4.45	12
3093	0	0	1	2	31.4	4.62	12.8
3094	0	0	0	4	12.1	4.41	11.5
3095	0	0	1	1	27.8	5	13.5
3096	0	0	0	2	5.6	4.55	12.6
3097	0	0	0	0	14	4.21	14.3
3098	0	0	1	0	11.6	4.7	12.3
3099	0	0	1	0	11.9	3.64	10.1
3100	0	0	0	2	4.4	2.64	7.9
3101	0	0	1	4	17.9	3.44	9
3102	0	0	0	1	4.3	3.38	10
3103	0	0	0	1	26.5	3.91	10
3104	0	0	0	2	6.1	5.1	12
3105	0	0	1	3	7.6	4.31	10.8
3106	0	0	0	2	6.4	4.79	12.9
3107	0	0	1	2	6.6	5.2	13.9
3108	0	0	0	2	15.3	4.45	11.6
3109	0	0	1	3	5.8	4.77	13.1
3110	0	0	0	2	4.9	3.81	10.7
3111	0	0	1	4	14.4	4.42	12.4
3112	0	0	1	3	8.2	4.69	12.5
3113	0	0	1	2	3.4	4.74	13.4
3114	0	0	0	2	5.3	4.58	12.9
3115	0	0	0	3	12.9	3.87	10.2
3116	0	0	1	0	16	4.32	14.1
3117	0	0	1	0	7.9	3.99	11.7
3118	0	0	1	3	6	4.64	12.8
3119	0	0	0	3	7	4.31	11.6
3120	0	0	1	1	13.4	4.31	10.9
3121	0	0	1	0	13.7	4.71	15.7
3122	0	0	1	4	12.6	4.41	12.1
3123	0	0	1	1	8.2	4.81	12.4
3124	0	0	0	3	5.1	4.64	12.7
3125	0	0	1	2	9.1	4.32	11.8
3126	0	0	1	1	24.1	4.32	11.2
3127	0	0	0	1	13.5	3.93	10.6
3128	0	0	0	3	7.6	4.71	13
3129	0	0	0	3	8.1	4.39	12.4
3130	0	0	1	0	7.9	4.5	9.8
3131	0	0	0	3	7.9	4.23	11.7
3132	0	0	0	0	8.8	3.45	10.7
3133	0	0	0	0	23.5	3.99	11.6
3134	0	0	0	2	14.3	4.26	11.4
3135	0	0	0	3	4.8	4.76	13.1

3136	0	0	1	3	8.4	4.73	12.7
3137	0	0	0	0	4.9	3.41	10.7
3138	0	0	1	3	6.9	4.33	11.4
3139	0	0	0	3	13.1	4.33	11.5
3140	0	0	1	3	9.7	4.63	12.2
3141	0	0	1	2	10.2	4.7	13.6
3142	0	0	0	3	10.6	4.67	12.4
3143	0	0	0	3	5.7	4.28	11.2
3144	0	0	1	4	5.3	4.82	12.2
3145	0	0	1	0	17.4	3.13	9.8
3146	0	0	0	3	7.7	4.31	12.5
3147	0	0	1	1	17	4.26	11.5
3148	0	0	0	2	9.1	5.22	14.2
3149	0	0	0	1	3.7	4.79	10.4
3150	0	0	1	2	28.1	4.73	12.9
3151	0	0	1	0	17.3	4.06	13.7
3152	0	0	1	1	12.4	5.16	12.8
3153	0	0	1	4	3.8	5.15	13.7
3154	0	0	1	4	18	5.29	14.3
3155	0	0	1	3	5.7	5.17	13.9
3156	0	0	1	4	16.3	4.86	12
3157	0	0	1	3	3.9	4.97	12
3158	0	0	0	4	9.4	4.79	13.1
3159	0	0	1	1	9.5	3.96	9.7
3160	0	0	1	3	10.7	4.44	12.3
3161	0	0	1	3	4.6	5.26	12
3162	0	0	0	4	12.9	4.39	12
3163	0	0	0	0	3.8	3.61	12.5
3164	0	0	0	2	7.6	4.94	13
3165	0	0	0	3	8.8	4.66	12.7
3166	0	0	1	1	15.2	4.66	12.5
3167	0	0	1	2	8.5	4.94	12.6
3168	0	0	0	2	14.1	4.42	12.1
3169	0	0	0	2	12	4.73	11.8
3170	0	0	0	2	5.2	4.11	12
3171	0	0	0	3	13.2	4.28	11.8
3172	0	0	1	2	12.6	4.62	12.7
3173	0	0	0	4	7.5	4.55	12.6
3174	0	0	1	2	3.6	4.32	11.7
3175	0	0	0	3	8.2	4.06	11.8
3176	0	0	1	3	15.7	4.8	12
3177	0	0	1	2	17.4	5.01	12.9
3178	0	0	1	4	6.8	4.9	12.8
3179	0	0	1	2	9.5	4.7	13.3
3180	0	0	1	4	9.2	4.85	13.1
3181	0	0	1	4	11.4	4.67	13.7

3182	0	0	1	2	21.8	4.59	12.7
3183	0	0	1	4	17.7	4.96	12.1
3184	0	0	1	3	6.4	4.56	12.1
3185	0	0	1	4	3.7	4.77	13
3186	0	0	1	3	8.9	4.51	12.2
3187	0	0	1	1	13.6	4.91	12.1
3188	0	0	1	3	8	4.8	13
3189	0	0	1	4	18.5	4.05	11.3
3190	0	0	1	3	10	5.15	13.9
3191	0	0	1	4	9.2	5.25	13.1
3192	0	0	0	2	8.9	4.4	11.6
3193	0	0	1	3	8.7	4.47	12.1
3194	0	0	1	3	5.1	3.87	11.9
3195	0	0	1	1	10.1	4.24	11.2
3196	0	0	0	3	11.8	5.08	13.5
3197	0	0	1	2	4.6	4.94	13.3
3198	0	0	0	3	7	4.34	12.6
3199	0	0	1	3	7.8	4.14	11.4
3200	0	0	1	3	7	4.71	12.5
3201	0	0	1	3	5.6	4.96	13.2
3202	0	0	1	2	9.5	5.16	13.9
3203	0	0	1	2	15.1	4.6	11.4
3204	0	0	1	1	9.5	3.95	11.2
3205	0	0	0	4	33.9	1.85	4.9
3206	0	0	0	4	19.8	3.9	10.7
3207	0	0	1	1	11.7	4.32	10.9
3208	0	0	0	1	11.2	4.49	12.3
3209	0	0	0	1	11.7	4	10.2
3210	0	0	0	1	25.9	2.83	8.2
3211	0	0	0	2	10.9	4.39	12.1
3212	0	0	1	2	8	4.79	13.4
3213	0	0	0	1	13.7	4.01	10.6
3214	0	0	0	3	2.9	4.52	12
3215	0	0	0	3	5.5	3.81	10.8
3216	0	0	1	1	7.5	3.13	9.8
3217	0	0	1	0	10.6	4.35	14.5
3218	0	0	0	4	24.8	4.01	11
3219	0	0	1	2	14	4.95	12.5
3220	0	0	0	2	12.9	4.82	12.8
3221	0	0	1	3	10.4	5.15	13.5
3222	0	0	0	3	7.3	3.7	9.1
3223	0	0	0	2	4.1	4.95	10.2
3224	0	0	1	1	3.9	2.83	8.4
3225	0	0	1	3	12.9	4.84	12.5
3226	0	0	1	3	23.7	4.64	12.1
3227	0	0	0	1	18.5	4.38	11.1

3228	0	0	1	1	13	4.61	11.6
3229	0	0	1	2	16.1	5.49	14.3
3230	0	0	0	0	17.3	3.75	9.7
3231	0	0	1	2	2.8	4.59	11.6
3232	0	0	1	1	12.9	3.51	10.3
3233	0	0	0	2	13.1	5.1	13.8
3234	0	0	0	1	9.5	4.24	11.7
3235	0	0	0	3	10.9	4.54	12.4
3236	0	0	0	1	12.6	3.36	9.3
3237	0	0	0	1	5.3	4.69	12.7
3238	0	0	0	2	8.8	4.81	11.9
3239	0	0	1	1	25.3	3.57	10.4
3240	0	0	1	2	12.1	4.59	13.1
3241	0	0	0	4	2.2	4.03	10.3
3242	0	0	0	3	7.7	4.51	12.7
3243	0	0	1	3	0.8	3.6	10.6
3244	0	0	1	1	11	4.14	11.3
3245	0	0	1	1	12.5	4.76	12.8
3246	0	0	1	2	12.1	4.34	11.4
3247	0	0	0	4	9.8	4.16	10.8
3248	0	0	0	3	11.3	4.85	14
3249	0	0	1	1	12.3	4.09	10.5
3250	0	0	0	3	5.3	4.76	12.7
3251	0	0	0	2	8.5	4.77	12.8
3252	0	0	1	1	6.4	4.45	12.6
3253	0	0	0	3	15.3	4.72	13.2
3254	0	0	1	3	8	4.28	11.8
3255	0	0	1	4	10.8	4.66	11.3
3256	0	0	0	3	14.9	4.47	12.3
3257	0	0	1	1	18.3	4.63	11.1
3258	0	0	0	4	7.8	4.22	11.8
3259	0	0	0	4	6.3	4.5	12.6
3260	0	0	0	2	9.2	4.72	11.9
3261	0	0	0	3	15.2	4.67	12.9
3262	0	0	0	1	7.5	4.37	12.1
3263	0	0	0	2	4.5	4.29	11.8
3264	0	0	1	2	6.2	4.31	11.4
3265	0	0	1	2	9	4.8	13.1
3266	0	0	1	2	3.4	4.29	11.4
3267	0	0	0	3	14.5	4.58	12.4
3268	0	0	1	0	21.1	3.64	10
3269	0	0	1	3	10.9	4.5	11.9
3270	0	0	1	3	4.8	4.7	12.5
3271	0	0	0	2	3.9	4.3	12.1
3272	0	0	1	3	16.3	4.34	11.9
3273	0	0	0	1	10.5	4.52	11.9

3274	0	0	0	4	23.8	3.45	9.1
3275	0	0	1	0	18.6	4.48	11.9
3276	0	0	0	2	8.4	4.47	12.4
3277	0	0	0	1	33.7	4.68	13
3278	0	0	1	0	13.7	3.58	11.1
3279	0	0	0	3	22.1	4.34	11.7
3280	0	0	1	0	9.2	3.65	11.8
3281	0	0	1	1	28.8	3.94	10.2
3282	0	0	0	1	7.6	4.11	11.5
3283	0	0	1	0	5.8	3.36	10.1
3284	0	0	0	2	13.1	4.49	12.4
3285	0	0	1	2	11.9	4.53	12.4
3286	0	0	0	1	27	4.69	12.5
3287	0	0	1	2	12.9	4.46	11.7
3288	0	0	1	4	8	4.26	12.1
3289	0	0	0	0	10.4	2.79	8.6
3290	0	0	1	4	7.7	5.09	13.8
3291	0	0	0	1	9.8	4.08	10.3
3292	0	0	1	4	13.7	4.77	13.1
3293	0	0	0	2	8.3	4.77	12.9
3294	0	0	0	2	3.3	4.02	11.2
3295	0	0	0	4	4.2	4.94	12.9
3296	0	0	1	1	16.9	3.91	11.2
3297	0	0	1	1	17.7	4.44	10.7
3298	0	0	1	0	11.6	3.84	10
3299	0	0	1	1	12.5	4.48	11.6
3300	0	0	1	2	29.1	4.89	13.4
3301	0	0	0	2	3.4	4.32	11.5
3302	0	0	0	2	20.1	4.74	11.8
3303	0	0	0	3	7.2	4.59	12.5
3304	0	0	1	3	7.6	4.14	11.8
3305	0	0	0	1	15.7	4.57	12.5
3306	0	0	1	4	8.3	4.55	12.6
3307	0	0	1	1	20.7	4.44	11.6
3308	0	0	0	2	9.9	4.42	11.8
3309	0	0	0	3	6.6	3.77	10.2
3310	0	0	0	1	28.7	4.65	11.6
3311	0	0	1	3	12.2	4.55	12.2
3312	0	0	0	2	6.7	4.15	11
3313	0	0	0	0	10	3.91	9.9
3314	0	0	0	2	10.4	4.67	12.1
3315	0	0	0	2	10.8	4.91	12
3316	0	0	1	3	14	4.94	12.9
3317	0	0	1	2	10.4	4.7	11.2
3318	0	0	0	1	14.8	4.38	11.6
3319	0	0	1	1	7.9	4.65	12.2

3320	0	0	0	1	16.6	4.24	11.2
3321	0	0	0	0	8.5	2.87	8.7
3322	0	0	1	2	6.3	4.46	12
3323	0	0	0	2	14.8	4.02	11.2
3324	0	0	1	1	5.5	4.46	11.6
3325	0	0	1	4	30	5.43	15.2
3326	0	0	0	1	18.8	4.16	10.3
3327	0	0	0	1	5.2	5.29	12.4
3328	0	0	1	0	21.4	3.93	13.6
3329	0	0	0	1	10.2	3.73	10.9
3330	0	0	1	2	5.3	3.44	8.5
3331	0	0	0	1	12.1	4.13	11.2
3332	0	0	1	0	16.5	3.44	11
3333	0	0	0	0	10.5	3.52	9.6
3334	0	0	1	1	4	4.29	11.3
3335	0	0	0	2	10.6	3.94	11.1
3336	0	0	0	0	23.2	3.39	9.4
3337	0	0	0	1	17.4	4.54	11.3
3338	0	0	0	2	13.7	4.82	13
3339	0	0	1	2	13.7	3.94	11
3340	0	0	0	3	7.3	4.77	13.2
3341	0	0	0	1	9.4	4.27	11.9
3342	0	0	1	0	4.1	3.35	9.8
3343	0	0	1	0	10.3	3.15	9.7
3344	0	0	0	2	4.2	4.84	12.7
3345	0	0	0	3	10.3	4.6	12.7
3346	0	0	1	0	10.9	3.42	10.4
3347	0	0	0	3	8.7	4.41	11.8
3348	0	0	0	2	16.4	4.24	11.5
3349	0	0	0	3	13	4.44	12
3350	0	0	1	3	6.4	3.91	11.1
3351	0	0	1	4	8.4	4.82	12.4
3352	0	0	1	0	26	4.08	9.9
3353	0	0	1	1	18.1	3.93	9.9
3354	0	0	0	1	3.5	4.64	11.6
3355	0	0	0	2	18.6	3.99	11.2
3356	0	0	0	2	16.3	4.81	12.6
3357	0	0	1	0	11.8	3.03	9.7
3358	0	0	0	2	10.8	4.87	11.3
3359	0	0	1	0	7.7	3.43	11.3
3360	0	0	0	4	5	4.23	11.6
3361	0	0	0	3	6.2	4.87	13
3362	0	0	0	0	10.2	2.9	9.3
3363	0	0	0	2	8.2	4.31	11
3364	0	0	1	3	6.2	4.64	11.8
3365	0	0	1	0	5	3.77	8.1

3366	0	0	0	1	18.5	4.31	11.8
3367	0	0	0	3	10.1	4.17	11.4
3368	0	0	0	1	4.9	4.87	12.8
3369	0	0	0	3	19.5	4.48	12.4
3370	0	0	1	2	19.2	4.61	13.5
3371	0	0	0	2	17.7	4.78	12.5
3372	0	0	1	0	15.1	3.39	9.6
3373	0	0	0	2	23.6	4.19	11.2
3374	0	0	0	1	29.1	4.19	10.9
3375	0	0	0	2	7.9	5.21	13.1
3376	0	0	1	2	17.4	4.4	12.3
3377	0	0	1	2	5	4.58	12.6
3378	0	0	0	0	9.5	4.07	11.3
3379	0	0	1	4	10.2	4.16	11.6
3380	0	0	1	3	14.8	4.35	11.6
3381	0	0	1	2	7.8	4.6	12.4
3382	0	0	0	2	3.1	3.73	10.7
3383	0	0	0	4	7.6	4.24	11.4
3384	0	0	1	0	3.8	4.09	10.7
3385	0	0	1	2	5.6	4.59	12.2
3386	0	0	1	3	8.2	4.74	13.3
3387	0	0	0	1	28.3	4.11	10.4
3388	0	0	1	3	9.5	4.46	12.2
3389	0	0	1	0	15.6	3.53	10.3
3390	0	0	1	0	13.1	4.5	11.7
3391	0	0	1	2	6	4.72	12.3
3392	0	0	0	4	10.1	4.02	10
3393	0	0	1	1	4.8	4.49	11.5
3394	0	0	1	4	7.5	3.94	11.6
3395	0	0	0	0	11.8	4.07	12.6
3396	0	0	1	2	14.3	5.09	12.9
3397	0	0	0	1	20.5	3.96	10.9
3398	0	0	0	1	15.9	4.89	12.9
3399	0	0	1	1	12.3	4.77	12.1
3400	0	0	0	4	2.8	4.57	12.4
3401	0	0	1	0	7.9	4.58	15.4
3402	0	0	0	0	11.4	4.09	10.7
3403	0	0	0	0	11.7	4.07	10.6
3404	0	0	0	2	13.4	4.45	12.5
3405	0	0	0	3	11.9	4.58	13.2
3406	0	0	1	2	12.1	5.59	13
3407	0	0	0	0	31.1	4.48	14
3408	0	0	0	1	17.1	4.13	9.6
3409	0	0	1	0	17	4.38	11.3
3410	0	0	0	3	2.2	4.5	12.4
3411	0	0	1	4	6.9	4.96	13

3412	0	0	0	3	5.8	4.55	12.7
3413	0	0	0	2	8.6	4.84	10.9
3414	0	0	0	4	8.5	4.99	13.9
3415	0	0	0	4	14.2	4.57	12.9
3416	0	0	0	3	3	4.3	11.2
3417	0	0	1	0	14.3	3.54	11.2
3418	0	0	0	0	15.8	3.71	11.3
3419	0	0	0	3	3.6	4.1	11.5
3420	0	0	0	2	8.3	4.24	11.9
3422	0	0	0	4	4.9	4.73	13.6
3423	0	0	0	2	8.8	4.64	11.8
3424	0	0	1	1	12.3	4.47	11.4
3425	0	0	1	3	5.7	4.64	12
3426	0	0	1	0	17	3.12	9.3
3427	0	0	0	2	16.2	4.43	12.1
3428	0	0	0	2	6.7	4.56	12.3
3429	0	0	1	4	20.7	5.55	14.6
3430	0	0	1	2	15.8	4.18	10.8
3431	0	0	0	2	9.3	3.99	11.2
3432	0	0	0	0	10.2	3.87	10.9
3433	0	0	1	4	5.2	4.24	12.1
3434	0	0	1	0	10.1	3.82	13.7
3435	0	0	0	3	13.7	4.54	12.5
3436	0	0	1	1	5	4.12	11.9
3437	0	0	0	0	13.2	3.55	9.6
3438	0	0	0	3	17	4.18	11.7
3439	0	0	1	1	12.6	4.76	11.9
3440	0	0	1	2	5.9	4.34	12.2
3441	0	0	1	1	8.4	4.64	11.5
3442	0	0	1	2	8.6	5.67	13.3
3443	0	0	1	3	8.4	4.56	11.6
3444	0	0	0	2	10.3	4.32	12.6
3445	0	0	1	4	7.6	4.52	11.6
3446	0	0	0	2	21.2	4.67	10.6
3447	0	0	0	0	13.8	3.51	11
3448	0	0	0	2	6	4.6	12.4
3449	0	0	1	0	8.5	3.91	10.7
3450	0	0	1	0	7.1	3.81	12.1
3451	0	0	0	3	4	4.73	13
3452	0	0	1	0	11.6	3.73	13.3
3453	0	0	1	1	9.1	4.57	11
3454	0	0	0	2	17.6	4.77	12.7
3455	0	0	1	0	11.3	3.24	9.5
3456	0	0	1	2	5.2	4.86	13
3457	0	0	1	0	11.7	3.79	10.6
3458	0	0	1	2	10.6	4.63	12.7

3459	0	0	1	0	9.9	3.79	11
3460	0	0	1	1	14.7	5.28	12.2
3461	0	0	0	2	21.9	3.77	10.1
3462	0	0	0	2	13.7	4.54	11.6
3463	0	0	1	0	18	3.25	9.5
3464	0	0	0	0	14	3.68	9.8
3465	0	0	0	3	3.1	4.31	11.3
3466	0	0	0	0	8.3	4.37	11.6
3467	0	0	0	0	35.8	4.2	11.1
3468	0	0	1	1	7.5	4.67	12
3469	0	0	0	2	8.4	4.77	12.8
3470	0	0	0	1	11.4	4.96	13.2
3471	0	0	1	0	13.9	3.7	11.9
3472	0	0	0	3	25.1	4.95	13.3
3473	0	0	1	1	10.1	4.15	10.8
3474	0	0	1	4	22.9	4.71	11.8
3475	0	0	1	0	9.6	3.94	11.1
3476	0	0	1	2	16.5	4.87	12.7
3477	0	0	0	0	12.5	4.81	17.7
3478	0	0	0	1	16.9	4.84	12.2
3479	0	0	1	4	8.8	4.35	12.1
3480	0	0	0	0	12.5	4.07	11.3
3481	0	0	0	2	16	4.59	12.8
3482	0	0	1	3	9	4.54	12.2
3483	0	0	1	0	20.6	4.01	11
3484	0	0	1	1	4.3	4.53	11.7
3485	0	0	0	4	10.8	4.1	11
3486	0	0	1	3	5	4.41	11.7
3487	0	0	0	1	16	3.7	9.6
3488	0	0	0	3	4.1	4.06	11.2
3489	0	0	0	0	23.5	4.07	10.7
3490	0	0	0	4	2.6	4.78	12.1
3491	0	0	0	2	9.3	5.18	14.2
3492	0	0	1	4	13.1	4.47	12.4
3493	0	0	0	4	7.4	4.21	10.6
3494	0	0	1	3	8.8	4.43	11.8
3495	0	0	1	0	8.2	3.37	9.9
3496	0	0	1	1	13.4	5.26	13.3
3497	0	0	1	4	5.1	4.7	13.1
3498	0	0	1	4	16.9	4.57	11.7
3499	0	0	1	1	16.4	4.77	11.7
3500	0	0	1	0	11	4.39	10.8
3501	0	0	1	0	7.2	3.52	11.2
3502	0	0	1	0	9.6	4.34	11.3
3503	0	0	0	0	11.1	3.6	10.3
3504	0	0	1	1	4.5	4.32	11.1

3505	0	0	0	2	13.9	3.61	9.7
3506	0	0	1	0	12.4	3.35	10.9
3507	0	0	0	0	10.5	4.63	14.3
3508	0	0	1	0	19.9	4.68	11.9
3509	0	0	0	4	3	4.61	11.3
3510	0	0	0	1	22.3	4.31	11.7
3511	0	0	1	2	32.7	5.1	13.6
3512	0	0	1	2	7.6	5.24	13.5
3513	0	0	1	0	8.3	3.73	10.5
3514	0	0	1	2	4.4	4.56	12.3
3515	0	0	1	0	15.3	3.84	12.8
3516	0	0	0	0	10.2	3.57	10.4
3517	0	0	0	0	26.1	4.58	12.4
3518	0	0	1	2	25.9	4.69	11.9
3519	0	0	1	1	5.7	4.87	12.5
3520	0	0	1	3	9	4.35	12
3521	0	0	1	3	6	4.19	11.8
3522	0	0	1	1	8.2	4.44	11.9
3523	0	0	0	0	18.4	4.52	11.3
3524	0	0	1	2	5.1	4.95	12.7
3525	0	0	1	0	10.9	4	12.9
3526	0	0	1	2	10.6	4.83	13.4
3527	0	0	0	1	4.2	3.68	9
3528	0	0	1	3	2.8	3.19	9.5
3529	0	0	0	1	18.6	4.43	12
3530	0	0	0	0	10.1	4.13	12.1
3531	0	0	1	1	8	4.45	11.9
3532	0	0	1	0	9.2	3.3	9.7
3533	0	0	0	1	17.1	3.7	9.5
3534	0	0	1	2	8.1	4.61	12.7
3535	0	0	1	2	12.8	4.53	11.8
3536	0	0	1	0	14.1	3.37	10.3
3537	0	0	0	0	10.9	3.76	12.1
3538	0	0	1	1	8.3	4.99	13.1
3539	0	0	1	4	21.9	4.16	11
3540	0	0	1	4	13.1	4.02	11.3
3541	0	0	1	1	27.5	4.27	11.3
3542	0	0	0	4	25.5	5.34	12.6
3543	0	0	1	1	4.8	4.64	11.9
3544	0	0	0	0	8	3.77	11.5
3545	0	0	1	3	4.8	4.81	12.3
3546	0	0	0	0	12	2.34	7.5
3547	0	0	0	4	8.6	4.46	11.5
3548	0	0	1	1	7.3	4.11	10.7
3549	0	0	1	1	7.5	4.11	10
3550	0	0	1	0	21.5	3.29	9.8

3551	0	0	1	2	19	4.4	11.2
3552	0	0	1	0	23.1	3.55	11.1
3553	0	0	1	2	9.8	4.29	12.3
3554	0	0	0	3	7	4.85	12.5
3555	0	0	1	0	6.5	3.76	11.3
3556	0	0	0	3	4.4	5.19	12.4
3557	0	0	1	2	22.5	4.41	11.7
3558	0	0	0	0	17.8	4.46	11.5
3559	0	0	0	3	15.4	4.81	12.7
3560	0	0	0	1	25.9	4.14	11.2
3561	0	0	1	0	16.5	3.56	10.5
3562	0	0	1	3	13.5	4.78	13.8
3563	0	0	1	2	3.8	4.89	13.3
3564	0	0	1	1	9.4	4.96	12.2
3565	0	0	1	3	6.7	4.56	12.7
3566	0	0	0	4	23.6	4.16	11.6
3567	0	0	0	0	12.4	4.55	11.9
3568	0	0	1	2	8.8	4.67	12.5
3569	0	0	0	1	17.9	4.26	11.2
3570	0	0	1	0	4.5	3.7	10.3
3571	0	0	1	0	10.4	3.45	9.8
3572	0	0	1	3	14.8	5.03	12.9
3573	0	0	1	4	5.3	4.31	11.5
3574	0	0	0	3	7.8	5.26	11.1
3575	0	0	1	1	4	3.98	9.2
3576	0	0	1	2	10.8	4.53	12.6
3577	0	0	1	1	7.7	4.7	13
3578	0	0	1	4	11.8	4.24	12.1
3579	0	0	0	4	4.9	4.27	12.2
3580	0	0	0	2	7.3	4.2	11.4
3581	0	0	0	1	4.3	3.94	10.5
3582	0	0	0	1	3.7	4.04	10.7
3583	0	0	0	0	5.4	3.72	10.9
3584	0	0	0	2	9.2	3.84	9.4
3585	0	0	0	3	10.4	4.47	12.3
3586	0	0	0	2	11.6	5.03	14.6
3587	0	0	1	3	3.6	4.48	12.3
3588	0	0	1	2	13.3	5.3	13.6
3589	0	0	0	4	16.4	4.48	12.4
3590	0	0	1	1	15.5	4.5	11.4
3591	0	0	0	2	4.9	5.32	12.4
3592	0	0	0	2	10.3	4.27	11.7
3593	0	0	0	2	9.1	4.77	11.9
3594	0	0	1	1	13.5	4.61	10
3595	0	0	1	4	6	4.32	11.8
3596	0	0	1	2	6	4.65	12

3597	0	0	0	4	6.8	4.73	11.4
3598	0	0	0	1	6.9	4.41	11.8
3599	0	0	0	1	13.9	4.24	11.4
3600	0	0	0	3	20.9	4.7	13.5
3601	0	0	0	4	15.8	4.73	10.7
3602	0	0	0	3	11.6	4.42	12.1
3603	0	0	1	1	20.9	4.33	11.7
3604	0	0	1	4	11.2	4.69	12.6
3605	0	0	0	1	8.1	4.1	11.2
3606	0	0	0	4	7.7	4.75	13.6
3607	0	0	1	0	12.4	4.29	16.3
3608	0	0	1	1	19.4	4.49	11.4
3609	0	0	0	1	27.6	3.39	8.7
3610	0	0	0	2	15.6	4.33	10.5
3611	0	0	0	3	17.2	4.47	12.2
3612	0	0	1	1	6.4	3.75	10.5
3613	0	0	1	1	15	3.59	10
3614	0	0	0	4	4.5	4.56	12.8
3615	0	0	1	1	31.6	4.11	10.3
3616	0	0	1	1	7.3	4.36	11.7
3617	0	0	1	0	11.6	3.35	10.6
3618	0	0	1	3	40.5	3.97	10.7
3619	0	0	0	1	33.4	4.33	11.2
3620	0	0	1	1	13.5	3.73	11.2
3621	0	0	1	4	8.2	4.85	13.5
3622	0	0	0	1	6.8	3.85	11.6
3623	0	0	1	3	8.4	4.54	12.2
3624	0	0	0	0	12.9	3.77	11.2
3625	0	0	1	2	11.9	4.86	12.9
3626	0	0	1	0	13.3	3.19	10.3
3627	0	0	0	3	8.2	4.17	12.1
3628	0	0	1	4	8.9	4.18	9.7
3629	0	0	0	1	20.2	4.62	12.5
3630	0	0	1	2	7.6	4.37	11.3
3631	0	0	1	3	4	4.21	11.8
3632	0	0	0	1	6.7	4.43	12
3633	0	0	0	0	7	3.98	14
3634	0	0	0	0	11.1	4.35	14.9
3635	0	0	0	4	34.7	4.37	11.9
3636	0	0	0	1	28.8	3.92	10.6
3637	0	0	0	2	41.4	4.39	12.3
3638	0	0	1	1	13.4	4.06	12
3639	0	0	1	2	7.2	4.56	11.4
3640	0	0	1	1	8.3	4.02	9.7
3641	0	0	1	2	6.2	4.76	12.6
3642	0	0	1	2	15.2	4.72	12.4

3643	0	0	1	0	5.6	3.83	10.6
3644	0	0	0	1	4.3	3.81	10.3
3645	0	0	0	2	6.7	4.12	10.2
3646	0	0	0	0	16.8	3.95	11.1
3647	0	0	0	0	12.8	4.16	11.6
3648	0	0	0	1	20.2	4.17	11.4
3649	0	0	1	0	9.6	3.34	11.5
3650	0	0	1	0	10.6	4	10.3
3651	0	0	0	1	4.4	3.92	9.7
3652	0	0	1	0	13	3.27	10.2
3653	0	0	1	0	7.2	2.96	9.8
3654	0	0	1	0	9.9	3.93	10.6
3655	0	0	1	0	25.7	3.49	11.6
3656	0	0	0	1	10.2	4.27	11.4
3657	0	0	0	3	26.8	4.3	11
3658	0	0	0	1	16	4.08	11.1
3659	0	0	0	1	10.4	4.59	13
3660	0	0	1	3	4.9	4.16	10.8
3661	0	0	1	1	9.2	4.95	12.3
3662	0	0	1	4	15.5	4.68	12.5
3663	0	0	1	2	6	4.35	11.3
3664	0	0	1	3	7	4.12	11.3
3665	0	0	0	4	25.9	4.11	11.6
3666	0	0	0	0	18	3.53	10.1
3667	0	0	0	1	5.7	4.73	12.3
3668	0	0	1	4	5.8	5.08	14.3
3669	0	0	1	0	10.9	4.36	11.3
3670	0	0	1	3	13.3	4.72	11.6
3671	0	0	0	4	18.9	4.12	11.2
3672	0	0	1	0	11.3	2.94	8.4
3673	0	0	1	0	14.3	4.68	11.2
3674	0	0	0	0	12.9	3.27	9.2
3675	0	0	0	2	15.1	4.23	11.4
3676	0	0	1	2	6.7	4.72	13.6
3677	0	0	1	2	10	4.16	11
3678	0	0	0	1	11.5	4.35	11.3
3679	0	0	1	0	11	4.77	16.8
3680	0	0	1	1	15.2	4.61	11.9
3681	0	0	0	0	4.5	3.79	10.7
3682	0	0	0	1	7.7	4.52	12.4
3683	0	0	1	1	11.4	4.56	12.5
3684	0	0	1	3	10.8	4.99	12.9
3685	0	0	1	4	13.5	4.91	13.6
3686	0	0	0	0	9.3	4.22	12.4
3687	0	0	0	2	5.5	4.67	13.2
3688	0	0	0	1	12.4	4.27	11.9

3689	0	0	1	3	8.5	4.2	11.5
3690	0	0	0	1	21.1	4.21	9.9
3691	0	0	0	0	13.5	3.64	11.7
3692	0	0	0	0	7.2	3.15	9.8
3693	0	0	1	4	6.7	4.74	13
3694	0	0	1	0	31.3	3.74	10.4
3695	0	0	1	2	6.2	5.11	14.2
3696	0	0	1	1	19.9	4.27	11.8
3697	0	0	1	1	6.5	4.16	10.2
3698	0	0	1	2	5.7	4.48	12.1
3699	0	0	0	2	10.7	4.89	13.7
3700	0	0	1	2	12.3	4.42	11.8
3701	0	0	0	4	9	4.32	13
3702	0	0	1	1	8.5	4.2	11
3703	0	0	1	1	4.7	4.59	12.8
3704	0	0	0	0	5.7	3.28	9.6
3705	0	0	0	0	7.7	4.18	11.4
3706	0	0	1	0	15.1	3.23	9.3
3707	0	0	1	0	9.5	4.47	13.2
3708	0	0	1	4	12.6	4.92	13.3
3709	0	0	0	2	5.6	4.58	12.1
3710	0	0	1	4	17.6	3.64	9.9
3711	0	0	1	0	39.2	4.04	12.5
3712	0	0	0	0	6.1	4.13	11
3713	0	0	1	0	20.6	4.48	11.2
3714	0	0	1	0	12.3	3.7	10.3
3715	0	0	1	0	13.1	4.41	11.8
3716	0	0	0	2	8.9	4.36	11.5
3717	0	0	0	1	9.4	5.21	13.6
3718	0	0	0	1	20.4	4.49	12.3
3719	0	0	0	1	14.2	4.59	11.8
3720	0	0	1	4	4.9	4.62	12.5
3721	0	0	1	3	4.2	4.85	12.6
3722	0	0	1	4	17.6	4.7	12.6
3723	0	0	0	4	11.6	4.7	12.9
3724	0	0	1	4	12.4	4.3	11.5
3725	0	0	0	4	12	4.15	12.2
3726	0	0	1	1	18.6	4.62	11.4
3727	0	0	1	1	4.5	4.3	11.5
3728	0	0	1	3	11	4.41	12
3729	0	0	1	4	15.4	4.31	10.8
3730	0	0	0	1	13.5	4.87	11.3
3731	0	0	1	1	5.6	3.58	10.2
3732	0	0	0	1	10.2	4.55	10.8
3733	0	0	1	0	21.1	3.1	9.5
3734	0	0	1	0	11.8	4.14	13.6

3735	0	0	1	2	10	5.13	13.7
3736	0	0	1	3	5.8	4.86	13.6
3737	0	0	1	3	11.7	4.52	12.3
3738	0	0	1	1	3.7	4.12	10.6
3739	0	0	1	1	6	4.37	11.3
3740	0	0	0	1	3.7	3.91	9.1
3741	0	0	1	0	5	4.51	12.6
3742	0	0	0	0	10	3.3	10.5
3743	0	0	1	0	14.6	3.18	9.5
3744	0	0	1	1	6.1	4.74	12.7
3745	0	0	1	0	7.4	4.03	11.6
3746	0	0	0	1	20.5	3.87	10.5
3747	0	0	0	3	8.8	3.97	11.3
3748	0	0	1	1	3.9	3.97	10.1
3749	0	0	0	1	5.4	4.7	13.5
3750	0	0	0	1	5.1	4.73	13
3751	0	0	1	0	7.9	3.04	7.4
3752	0	0	1	0	3.9	2.96	8.1
3753	0	0	0	3	8.2	5.6	12.3
3754	0	0	0	2	15.6	4.37	11.7
3755	0	0	1	2	27.2	3.96	11.4
3756	0	0	1	0	14.4	4.34	11.3
3757	0	0	1	0	10.3	4.05	10.1
3758	0	0	0	4	10.4	4.62	12.9
3759	0	0	0	1	7.9	4.34	11.7
3760	0	0	1	0	15.9	3.29	10.9
3761	0	0	1	0	13.3	4.61	14.7
3762	0	0	1	2	4.8	5.35	13.6
3763	0	0	1	1	16.4	4.54	10.8
3764	0	0	0	0	11	5.08	17
3765	0	0	1	4	12.5	4.12	11.4
3766	0	0	0	2	9.8	4.37	11.4
3767	0	0	0	1	12.1	4.63	10.5
3768	0	0	1	1	6.3	4.06	11.4
3769	0	0	0	0	10.5	4.12	11.4
3770	0	0	1	3	5.3	4.75	13
3771	0	0	1	0	9.8	4.33	11.8
3772	0	0	0	0	5.4	3.43	11.1
3773	0	0	0	0	21.9	4.4	11.4
3774	0	0	1	0	14.1	4.12	11.7
3775	0	0	1	0	22.5	4.04	11.4
3776	0	0	0	1	8.6	4.61	10.3
3777	0	0	1	0	18.5	4.31	11.3
3778	0	0	1	4	23.1	4.49	12.4
3779	0	0	0	0	4	3.4	10.7
3780	0	0	1	1	2.7	3.11	9.7

3781	0	0	1	3	10	5.26	13.1
3782	0	0	0	1	8.4	4.67	12.4
3783	0	0	1	0	13.4	4.19	13.9
3784	0	0	1	0	16.3	4.09	11.4
3785	0	0	1	0	10.7	3.66	10.5
3786	0	0	1	1	6.4	4.88	12.2
3787	0	0	1	0	14.1	3.26	10.7
3788	0	0	1	0	9.3	3.45	9.6
3789	0	0	1	2	4.1	4.82	13.2
3790	0	0	0	0	17.6	3.09	8.6
3791	0	0	1	2	5.5	4.15	10.8
3792	0	0	0	1	7.1	4.1	11.1
3793	0	0	1	1	5.9	4.39	11.7
3794	0	0	1	0	13.6	3.41	9.4
3795	0	0	0	0	11.4	3.7	10.6
3796	0	0	1	0	19.9	4.2	12.1
3797	0	0	0	4	9.2	4.95	13.7
3798	0	0	0	2	11	4.94	12.8
3799	0	0	0	1	4.1	3.92	10.3
3800	0	0	0	1	9.4	4.31	11.3
3801	0	0	0	1	7.7	3.27	9.8
3802	0	0	0	2	11.7	4.62	12.8
3803	0	0	0	1	16.3	3.83	10.5
3804	0	0	0	2	7.7	4.09	10.9
3805	0	0	1	1	7.1	4.12	11
3806	0	0	0	3	3.8	3.5	9.9
3807	0	0	1	3	7.5	4.56	11.9
3808	0	0	1	2	11.3	4.81	10.4
3809	0	0	1	1	8.6	4.14	10.1
3810	0	0	0	2	7.1	4.31	11.8
3811	0	0	1	1	20.7	4.24	11
3812	0	0	0	0	14.3	4.38	12.2
3813	0	0	1	2	9.3	4.77	13.3
3814	0	0	1	0	28.6	4.63	11.7
3815	0	0	1	2	7.4	4.83	12.8
3816	0	0	0	2	7.6	5.44	13.9
3817	0	0	1	4	11.5	3.95	10.5
3818	0	0	1	2	24.4	4.3	11.6
3819	0	0	0	4	5.2	4.48	11.9
3820	0	0	1	3	13.8	4.61	12.5
3821	0	0	0	1	13.7	3.91	10.5
3822	0	0	0	4	7.9	4.21	12.1
3823	0	0	1	1	6	4.96	13
3824	0	0	1	3	5.3	4.9	12.9
3825	0	0	1	0	15.1	4.85	13
3826	0	0	0	2	8.9	4.57	11.7

3827	0	0	0	1	7.3	4.66	13
3828	0	0	0	3	6	4.62	12.7
3829	0	0	0	1	7.7	4.52	11.9
3830	0	0	0	2	6.6	4.19	11.5
3831	0	0	0	0	13.4	3.63	10.7
3832	0	0	1	1	19.2	4.67	10.7
3833	0	0	1	2	10.5	4.33	11.9
3834	0	0	1	2	7.6	4.92	12.8
3835	0	0	0	1	5.4	4.84	13.6
3836	0	0	0	1	6.6	4.13	11.2
3837	0	0	1	0	29.1	4.93	11.7
3838	0	0	0	2	9.7	4.27	11.9
3839	0	0	0	2	7.2	4.33	11.3
3840	0	0	1	1	5.9	5.19	12.7
3841	0	0	1	3	8.1	4.34	11.2
3842	0	0	0	1	10.4	4.62	12.3
3843	0	0	1	1	17.3	4.5	11.6
3844	0	0	0	3	5.9	4.65	12.9
3845	0	0	1	1	7.7	4.23	11.5
3846	0	0	1	1	8.8	4.53	12.3
3847	0	0	0	4	14	4.37	11.4
3848	0	0	1	1	15.3	3.89	11.2
3849	0	0	1	1	13.2	4.3	10.6
3850	0	0	1	1	7.5	4.48	11.3
3851	0	0	0	1	6	4.14	11
3852	0	0	1	1	11	3.5	9.9
3853	0	0	1	1	7.3	4.44	11.2
3854	0	0	1	2	14.3	3.97	11.3
3855	0	0	0	2	10.7	4.23	11.1
3856	0	0	1	4	28.3	4.72	12.7
3857	0	0	1	0	10.2	4.18	11.6
3858	0	0	0	3	12.8	4.66	13.5
3859	0	0	1	1	14.6	3.93	10.3
3860	0	0	1	1	4.6	3.99	10.9
3861	0	0	0	2	7.2	4.42	11.9
3862	0	0	0	4	14.2	4.43	11.4
3863	0	0	1	4	10.1	4.59	11.6
3864	0	0	1	0	10	4.59	15.5
3865	0	0	1	2	7.7	4.07	11.3
3866	0	0	1	2	10.2	4.6	12.6
3867	0	0	1	3	25.4	4.8	11.8
3868	0	0	0	1	9.3	4.43	11.3
3869	0	0	1	1	16.8	3.99	10.5
3870	0	0	0	1	6.2	4.54	12.3
3871	0	0	0	1	10.8	2.84	9.5
3872	0	0	0	1	9.1	3.68	9.8

3873	0	0	0	1	4.8	4.35	12
3874	0	0	0	1	10.4	3.64	9.9
3875	0	0	1	1	6.8	4.38	11.9
3876	0	0	1	2	9.7	4.6	12.1
3877	0	0	1	1	10.3	4.43	12.1
3878	0	0	1	1	5.5	2.54	7.8
3879	0	0	0	1	2.8	4.62	12.3
3880	0	0	0	1	19.2	4.16	10.4
3881	0	0	0	4	7.3	4.12	11.2
3882	0	0	1	3	16.3	4.71	12.5
3883	0	0	1	2	8.6	4.78	13.4
3884	0	0	0	1	16.1	4.46	11.9
3885	0	0	1	1	10	4.63	12.4
3886	0	0	0	1	11.2	4.86	12.6
3887	0	0	1	0	2.8	3.77	10.6
3888	0	0	1	2	7.9	4.35	11.6
3889	0	0	1	0	6.3	4	11.5
3890	0	0	0	3	4.9	5.14	13.1
3891	0	0	1	0	6.3	3.79	11.4
3892	0	0	0	2	9.1	4.71	13.9
3893	0	0	1	1	9.3	4.57	12.9
3894	0	0	1	3	13.5	4.19	11.4
3895	0	0	1	1	12.6	4.74	12.4
3896	0	0	1	1	5	4.71	11.2
3897	0	0	0	1	11.1	4.55	11.4
3898	0	0	1	0	12.8	3.82	10.1
3899	0	0	1	1	19	4.73	12.2
3900	0	0	1	4	4.9	4.56	12
3901	0	0	1	0	10.6	3.57	9.9
3902	0	0	1	2	10.2	4.89	13.5
3903	0	0	1	1	7.1	4.89	12.3
3904	0	0	0	3	5.1	5.09	14.5
3905	0	0	1	4	8.2	5.19	13.8
3906	0	0	0	4	11.3	5.64	13
3907	0	0	1	1	17.9	4.42	11.2
3908	0	0	0	1	7.8	4.74	12.5
3909	0	0	0	0	4.8	2.88	9
3910	0	0	1	0	9.4	3.69	11.3
3911	0	0	1	1	7.6	4.32	12.1
3912	0	0	1	2	13.3	4.92	13.5
3913	0	0	0	1	5.1	4.01	10.5
3914	0	0	1	1	11.2	4.57	11.4
3915	0	0	1	0	7.8	4.96	11.7
3916	0	0	0	0	11.9	3.7	12.1
3917	0	0	1	0	16.3	4.26	11.1
3918	0	0	0	1	7.7	4.33	11.3

3919	0	0	1	0	8.5	3.54	11
3920	0	0	1	1	8.3	4.4	12.2
3921	0	0	0	0	7	3.89	10.6
3922	0	0	1	0	4.1	2.87	8
3923	0	0	0	4	8.1	5.22	14
3924	0	0	0	4	11.9	4.53	12.2
3925	0	0	1	1	6	3.97	10.6
3926	0	0	1	3	11.3	4.5	12.6
3927	0	0	0	1	5.5	5.36	12.9
3928	0	0	1	1	11	4.59	12.5
3929	0	0	1	1	13.8	4.98	12.5
3930	0	0	0	1	13.5	4.36	11.5
3931	0	0	1	0	20.7	3.91	11.1
3932	0	0	0	0	16.4	3.77	10.7
3933	0	0	1	2	10	4.23	11.8
3934	0	0	0	1	5	4.32	11.7
3935	0	0	1	0	7.8	4.89	12.3
3936	0	0	1	1	9.8	4.47	11.7
3937	0	0	0	2	9.7	3.78	10.7
3938	0	0	0	2	5.3	4.17	11.4
3939	0	0	1	1	5.3	4.28	11.2
3940	0	0	0	4	10.9	4.27	11.9
3941	0	0	1	1	6.9	5.4	12.7
3942	0	0	1	4	7.8	4.46	11.8
3943	0	0	1	0	20.5	2.57	8
3944	0	0	0	3	13	4.47	12.2
3945	0	0	0	0	7	4.29	11.8
3946	0	0	0	2	10.5	4.68	11.6
3947	0	0	1	1	15.1	4.82	12
3948	0	0	0	0	13.1	4.71	12.9
3949	0	0	0	1	14.2	4.29	11.4
3950	0	0	0	0	7.4	4.09	10.9
3951	0	0	1	3	7.8	4.07	11.2
3952	0	0	0	0	11.8	4.15	12.3
3953	0	0	1	0	5.8	3.21	9.9
3954	0	0	1	2	7.5	4.64	13.1
3955	0	0	1	0	7.5	3.27	10.3
3956	0	0	1	2	5.6	4.6	12.5
3957	0	0	1	1	13.3	3.9	10
3958	0	0	0	0	17.5	4.12	11.5
3959	0	0	0	1	4.9	4.31	10.3
3960	0	0	1	3	10.8	4.93	12.8
3961	0	0	1	3	2.9	4.29	11.6
3962	0	0	1	1	9.7	4.7	11
3963	0	0	0	1	4.6	4.55	11.8
3964	0	0	0	1	8.5	4.52	12

3965	0	0	0	1	3.4	4.13	10.8
3966	0	0	1	1	11.3	4.2	11.3
3967	0	0	1	1	6.4	4.72	12.6
3968	0	0	0	0	11.5	4.11	11.8
3969	0	0	0	0	18.3	3.43	10.2
3970	0	0	1	3	3.4	4.92	12.9
3971	0	0	0	1	11.5	4.38	11.9
3972	0	0	0	1	7.3	4.12	10.8
3973	0	0	0	2	5.4	5.18	12.6
3974	0	0	0	1	13.7	4.64	12.3
3975	0	0	1	0	14.1	3.39	11
3976	0	0	0	1	5.2	4.4	11.7
3977	0	0	0	1	7.2	4.82	13.1
3978	0	0	0	2	9.1	5.22	11.7
3979	0	0	1	3	9.2	4.43	11.4
3980	0	0	0	1	6.8	4.67	12.4
3981	0	0	0	0	10.5	4.16	14
3982	0	0	1	3	6.1	3.88	10
3983	0	0	0	1	4.2	4.44	11.7
3984	0	0	1	2	13.4	3.86	10.8
3985	0	0	1	1	6.7	5.04	13.7
3986	0	0	0	1	4.6	4.68	11.5
3987	0	0	0	0	17.3	3.95	12.5
3988	0	0	0	0	19.2	3.58	11.2
3989	0	0	1	1	11.7	4.74	13.1
3990	0	0	1	2	4.9	4.64	12.5
3991	0	0	0	0	16.8	4.38	12.4
3992	0	0	1	1	9.2	4.77	12.8
3993	0	0	1	0	12.8	3.94	11.3
3994	0	0	0	0	16.7	4.07	10.9
3995	0	0	1	1	17.3	3.96	10.2
3996	0	0	0	2	9.8	5.11	12.8
3997	0	0	0	3	11.5	4.52	12
3998	0	0	1	0	9.2	3.48	9.7
3999	0	0	0	1	8.7	4.4	11.5
4000	0	0	1	1	11.9	4.35	11.7
4001	0	0	0	1	15.4	4.79	12.2
4002	0	0	1	1	7.7	4.26	11.3
4003	0	0	0	1	10.8	4.92	12.3
4004	0	0	0	1	5.1	4.55	11.9
4005	0	0	0	2	13.9	4.44	12
4006	0	0	1	0	5.1	4.17	11.4
4007	0	0	0	1	7.8	4.4	11.8
4008	0	0	0	0	20.5	3.67	10.5
4009	0	0	0	1	17.5	3.7	9.1
4010	0	0	1	0	14.5	3.65	12.6

4011	0	0	0	4	13.8	4.03	11.6
4012	0	0	0	2	17.9	4.5	11.6
4013	0	0	1	1	11.6	4.39	10.4
4014	0	0	1	1	10.8	4.75	11.2
4015	0	0	0	3	7.8	4.85	13.2
4016	0	0	0	0	13.4	3.06	9.3
4017	0	0	0	2	8.2	4.33	12.5
4018	0	0	1	4	3.5	4.05	10.8
4019	0	0	0	2	5.4	4.67	12.7
4020	0	0	1	1	4.2	4.02	11
4021	0	0	0	1	7.4	2.98	9.1
4022	0	0	0	1	6.6	4.42	12.2
4023	0	0	1	1	6.2	4.49	12
4024	0	0	1	1	9.6	4.61	11.9
4025	0	0	0	4	5.3	4.37	11.9
4026	0	0	0	4	6.1	4.13	11
4027	0	0	0	1	5.6	4.49	12.1
4028	0	0	0	2	5.2	4.43	11.4
4029	0	0	1	2	2.8	4.6	12.3
4030	0	0	1	1	14.8	2.65	8.8
4031	0	0	0	1	13	3.75	10.6
4032	0	0	1	1	5.7	4.47	11.2
4033	0	0	1	1	7.1	4.34	11.2
4034	0	0	0	1	8.5	4.46	12.4
4035	0	0	1	0	8	3.55	11.5
4036	0	0	0	1	6.9	3.92	11.3
4037	0	0	0	1	8	4.12	11.6
4038	0	0	1	3	7.4	4.36	12.2
4039	0	0	1	2	7.5	4.32	12.5
4040	0	0	1	1	7.3	4.62	12.3
4041	0	0	1	2	15.5	4.62	12.6
4042	0	0	1	4	8.1	4.56	12
4043	0	0	1	1	6.7	4.29	11.5
4044	0	0	1	1	14.8	3.04	9.6
4045	0	0	1	1	8.3	3.33	9.3
4046	0	0	0	1	8.7	4.18	11.2
4047	0	0	1	3	17.7	4.56	12.6
4048	0	0	1	2	3.6	4.72	12.9
4049	0	0	0	1	23.3	3.59	10
4050	0	0	1	3	7.4	4.49	11.7
4051	0	0	1	0	22.8	4.72	15.9
4052	0	0	1	1	3.7	2.91	8.6
4053	0	0	1	2	19.7	4.91	13.2
4054	0	0	1	2	15.6	4.86	11.9
4055	0	0	0	1	15.1	3.14	9.7
4056	0	0	0	1	18.4	3.69	9.3

4057	0	0	1	1	15.2	4.05	10.9
4058	0	0	0	1	19.2	4.12	10.4
4059	0	0	1	1	9.1	3.46	9.6
4060	0	0	0	4	5.2	4.19	11.7
4061	0	0	0	1	4.5	4.78	12.9
4062	0	0	1	3	4.8	4.8	12.9
4063	0	0	1	1	4.8	4.31	11.1
4064	0	0	0	0	12.7	4.41	15.3
4065	0	0	0	1	16	4.19	12.3
4066	0	0	0	2	13.6	4.77	13.4
4067	0	0	1	1	14.1	4.37	11.6
4068	0	0	0	2	11.4	4.33	11.8
4069	0	0	0	4	3.6	3.95	11.1
4070	0	0	0	0	12.1	4.93	16.8
4071	0	0	0	0	7	3.26	10.4
4072	0	0	1	1	8.2	4.61	12.2
4073	0	0	0	4	21.4	4.47	11.6
4074	0	0	1	3	12.9	4.71	11.8
4075	0	0	0	2	7.7	4.34	11.4
4076	0	0	0	2	5.8	4.35	12.3
4077	0	0	0	3	21.5	4.26	10.9
4078	0	0	1	3	12.5	4.59	12.5
4079	0	0	0	4	10.8	4.14	11.6
4080	0	0	0	4	14.4	4.29	11.5
4081	0	0	0	1	6.6	4.03	10.5
4082	0	0	1	4	11.9	4.69	12
4083	0	0	1	2	14.6	5.05	13.4
4084	0	0	1	3	9.4	5.04	13.2
4085	0	0	1	1	12.6	3.52	9.2
4086	0	0	1	1	10	4.13	10.7
4087	0	0	0	4	8.6	4.36	12.6
4088	0	0	1	2	10.1	4.62	12.4
4089	0	0	1	1	7	4.38	11.5
4090	0	0	1	1	15.5	4.23	10.9
4091	0	0	1	1	9.5	5.29	11
4092	0	0	0	1	17.4	4.44	11.9
4093	0	0	0	2	4.8	5.17	14
4094	0	0	1	2	11.7	5.1	13.1
4095	0	0	0	0	16.8	4.93	14.8
4096	0	0	1	1	4.7	3.78	11
4097	0	0	1	2	5.5	4.54	12.1
4098	0	0	1	1	11.9	4.52	12.5
4099	0	0	1	4	17.4	4.63	12.3
4100	0	0	0	1	6.6	5.37	14.1
4101	0	0	0	1	22.7	4.41	10.8
4102	0	0	0	3	5.5	4.5	11.4

4103	0	0	1	1	9.8	4.49	12.2
4104	0	0	1	4	6.2	4.75	12.4
4105	0	0	0	1	11.4	3.89	8.3
4106	0	0	0	4	10.3	4.25	11.1
4107	0	0	0	0	14.2	5.47	19.8
4108	0	0	0	3	6.2	4.89	12.6
4109	0	0	1	3	22.4	4.29	11.8
4110	0	0	1	1	6.5	4.09	11.8
4111	0	0	1	1	7.9	5.13	11.9
4112	0	0	1	2	8.1	4.57	12.5
4113	0	0	1	1	4.9	4.56	11.5
4114	0	0	1	1	11.2	4.7	12.6
4115	0	0	0	1	16.4	4.39	10.5
4116	0	0	0	2	11.1	4.91	12.9
4117	0	0	1	1	7.3	4.2	10.2
4118	0	0	1	3	4.9	4.97	13.8
4119	0	0	1	0	6.3	3.56	11.5
4120	0	0	0	1	4.9	4.65	12
4121	0	0	1	1	15.4	3.95	11.1
4122	0	0	0	0	16.4	3.83	10.5
4123	0	0	1	1	10.5	4.12	9.7
4124	0	0	1	2	5.6	4.21	11.8
4125	0	0	0	1	16.3	4.11	9.8
4126	0	0	0	1	6.5	4.41	11.1
4127	0	0	1	4	4.2	4.24	11.5
4128	0	0	1	1	6.2	4.64	11.5
4129	0	0	1	1	9.2	3.84	9.5
4130	0	0	1	3	7.2	4.3	11.8
4131	0	0	0	1	21.2	4.18	10.5
4132	0	0	0	1	9.6	3	9.4
4133	0	0	1	1	12.1	5.09	12.8
4134	0	0	1	3	10.9	4.64	12.2
4135	0	0	1	0	10.5	3.57	11.3
4136	0	0	1	1	3.7	4.83	12.4
4137	0	0	1	1	6.1	4.49	10.2
4138	0	0	0	1	7	4.03	11
4139	0	0	0	1	5.9	4.15	11.4
4140	0	0	1	1	4	4.5	11.4
4141	0	0	1	2	7.1	4.7	11.5
4142	0	0	0	3	4.8	4.97	12.4
4143	0	0	1	1	7.7	3.98	10.4
4144	0	0	1	2	14.9	5.06	13.2
4145	0	0	0	2	7.2	4.34	11.3
4146	0	0	1	2	7.5	4.43	12.1
4147	0	0	1	3	7.8	4.55	12.2
4148	0	0	0	0	11.9	3.57	12.1

4149	0	0	0	1	9.9	4.35	12.2
4150	0	0	0	3	8.1	4.64	12.4
4151	0	0	0	1	10.5	3.99	10.1
4152	0	0	1	1	5.2	4.36	10.9
4153	0	0	1	1	12.6	4.8	12.6
4154	0	0	1	2	7.9	4.98	11.7
4155	0	0	1	3	11.8	4.66	11.1
4156	0	0	0	2	11.8	4.33	11.1
4157	0	0	1	1	3.6	4.49	11.7
4158	0	0	1	0	10.8	3.89	10.8
4159	0	0	0	0	10.6	4.1	10.5
4160	0	0	0	0	6.5	4.53	15.9
4161	0	0	1	1	6.8	4.43	11.8
4162	0	0	0	0	12.9	4.18	13.4
4163	0	0	1	0	15.6	3.92	12.7
4164	0	0	1	1	15.7	4.34	11.4
4165	0	0	1	1	11	4.93	13.2
4166	0	0	1	1	23	4.55	11.7
4167	0	0	1	2	15	5.23	12.9
4168	0	0	1	1	18.5	4.23	11.9
4169	0	0	1	0	8.9	3.09	9.5
4170	0	0	1	0	10.6	4.05	12.4
4171	0	0	1	1	11	4.79	12.5
4172	0	0	0	2	19.6	4.23	11.6
4173	0	0	1	2	5.1	3.73	10.1
4174	0	0	0	2	5.8	4.22	11.6
4175	0	0	1	3	10.9	4.68	12.6
4176	0	0	1	2	3.8	5.25	13.6
4177	0	0	0	0	16.4	3.17	10.4
4178	0	0	0	2	22.1	4.2	11.3
4179	0	0	0	2	7.5	4.1	10.8
4180	0	0	0	1	4.7	4.42	11.9
4181	0	0	1	1	13	3.57	10
4182	0	0	0	2	8.9	3.9	10.7
4183	0	0	0	1	16	4.62	11.8
4184	0	0	1	2	4.1	4.68	12
4185	0	0	0	1	5.4	4.54	11.8
4186	0	0	1	2	9.5	4.06	11.1
4187	0	0	0	1	8	4.32	11.3
4188	0	0	0	1	6.2	3.32	9.1
4189	0	0	0	1	12.6	4.01	10.1
4190	0	0	1	1	5.6	4.2	11.1
4191	0	0	0	1	14.3	4.3	11.7
4192	0	0	1	1	7	4.34	11.8
4193	0	0	1	1	11.3	4.37	11
4194	0	0	0	1	10	4.19	11.1

4195	0	0	1	2	1.1	4.14	10.9
4196	0	0	0	3	13.3	4.85	12.6
4197	0	0	0	2	11.3	4.45	12.7
4198	0	0	0	0	11.9	3.29	10.5
4199	0	0	1	1	6.5	4.38	10.7
4200	0	0	1	1	6.7	4.01	11.1
4201	0	0	1	0	13.3	3.83	10.6
4202	0	0	0	1	6.3	4.49	11
4203	0	0	1	0	17.8	3.3	9.9
4204	0	0	0	4	8.3	5.11	13.5
4205	0	0	1	2	8.4	4.73	13
4206	0	0	0	1	7.7	4.92	11.9
4207	0	0	1	2	8.2	4.09	11.3
4208	0	0	1	1	13.8	4.3	11
4209	0	0	0	1	4.4	4.67	13.4
4210	0	0	0	2	4.8	4.75	13.2
4211	0	0	0	1	6.6	4.21	11.4
4212	0	0	1	1	2.7	4.68	11.5
4213	0	0	0	1	9.4	4.79	11.9
4214	0	0	1	2	22.7	4.58	12.3
4215	0	0	1	1	10.8	4.4	11.9
4216	0	0	1	0	12.2	4	12.6
4217	0	0	0	1	17.8	5.12	13.8
4218	0	0	1	0	6.9	4.14	14.8
4219	0	0	1	1	5.2	4.44	11.5
4220	0	0	1	1	9.6	4.42	12.5
4221	0	0	1	3	9.4	4.64	12.1
4222	0	0	0	2	8.6	4.74	12.9
4223	0	0	1	0	12.4	3.16	9.8
4224	0	0	1	1	15.7	4.6	11.6
4225	0	0	0	1	16.4	4.33	11.8
4226	0	0	0	1	4.7	4.75	12.7
4227	0	0	0	4	10.9	4.43	12.2
4228	0	0	1	2	15.7	4.72	13.3
4229	0	0	0	0	26.7	5.17	13.5
4230	0	0	0	1	10.4	4.94	13.3
4231	0	0	1	0	12.5	4.72	12.1
4232	0	0	1	0	6.3	3.21	9.5
4233	0	0	1	4	12.6	3.75	9.7
4234	0	0	0	2	7.8	4.73	12.1
4235	0	0	0	3	6.1	4.48	12.1
4236	0	0	1	1	13.7	4.7	11.3
4237	0	0	1	1	12.4	2.99	9
4238	0	0	1	1	7.3	5	11.9
4239	0	0	1	2	4	4.76	12
4240	0	0	0	1	25.1	2.92	8.1

4241	0	0	1	0	3.7	4.35	11.1
4242	0	0	1	0	14.4	3.84	10.7
4243	0	0	0	2	21	4.61	10.8
4244	0	0	1	0	5.6	3.53	9.1
4245	0	0	1	1	3.6	4.55	11.8
4246	0	0	0	1	15.2	4.06	10.5
4247	0	0	0	1	6	4.05	11.1
4248	0	0	1	2	16.5	4.6	12.8
4249	0	0	0	2	19.5	4.91	12.7
4250	0	0	0	0	9.1	4.02	11.2
4251	0	0	0	4	10.1	4.89	12
4252	0	0	0	2	13.1	4.83	13.2
4253	0	0	0	2	5.3	4.91	12.8
4254	0	0	1	0	17	3.44	9.7
4255	0	0	0	0	13.5	3.26	9.3
4256	0	0	0	4	10.3	4.32	12
4257	0	0	1	1	12.3	4.87	13
4258	0	0	1	1	9.2	5.04	12.9
4259	0	0	1	0	6.9	3.49	10.4
4260	0	0	1	4	9.4	4.36	11.8
4261	0	0	0	2	5	5.07	12
4262	0	0	0	1	10.2	4.44	11.5
4263	0	0	1	2	8.5	5.04	12.3
4264	0	0	1	1	3.2	4.74	12.4
4265	0	0	0	1	5.5	4.41	12
4266	0	0	1	0	17.6	3.22	10
4267	0	0	1	1	6.1	4.75	12.8
4268	0	0	0	3	13.3	4.02	10.2
4269	0	0	0	2	5.9	3.89	11.1
4270	0	0	0	3	7.1	4.51	12.8
4271	0	0	1	4	21.4	5.12	13.7
4272	0	0	1	0	13.5	3.84	10
4273	0	0	1	4	22.8	1.83	5.8
4274	0	0	1	2	7.3	4.32	11.3
4275	0	0	0	0	7	4.46	16.4
4276	0	0	1	0	9.6	4.51	12.4
4277	0	0	0	2	7.4	4.71	12.4
4278	0	0	1	1	9.9	5.06	13.2
4279	0	0	0	4	17.1	4.71	11.8
4280	0	0	1	1	10.1	4.26	11.4
4281	0	0	0	2	8.6	4.44	13
4282	0	0	0	4	13.7	4.46	11.8
4283	0	0	1	4	8.8	4.51	12.3
4284	0	0	1	1	4.7	4.29	11.5
4285	0	0	1	3	20.2	4.36	11.5
4286	0	0	0	1	9.2	4.83	12.9

4287	0	0	1	1	20.5	4.51	11.7
4288	0	0	1	3	8.8	4.81	13.8
4289	0	0	0	3	4.8	4.42	12.3
4290	0	0	0	3	10.6	4.55	12.5
4291	0	0	0	2	13.8	4.63	12.3
4292	0	0	0	1	7.3	4.66	12.5
4293	0	0	0	4	15.8	4.4	11.6
4294	0	0	1	1	14.6	5.43	13.8
4295	0	0	1	4	2.7	4.36	11.8
4296	0	0	1	1	10.1	4.42	12.1
4297	0	0	1	1	3.1	4.67	10.4
4298	0	0	1	2	13.8	5.16	14.3
4299	0	0	0	1	15.7	4.58	11.1
4300	0	0	1	1	3.2	4.66	12.4
4301	0	0	1	2	14.5	4.57	12.6
4302	0	0	1	1	3	5.02	11.6
4303	0	0	0	3	3.8	4.47	12.5
4304	0	0	1	1	16.8	4.47	11.8
4305	0	0	0	2	4.6	4.92	13.3
4306	0	0	1	2	15.4	4.88	13.6
4307	0	0	1	1	16	4.42	12.4
4308	0	0	1	2	9.1	5.15	13.9
4309	0	0	0	1	18.3	4.17	11.9
4310	0	0	0	4	22	4.03	11.7
4311	0	0	0	1	13.2	4	10.3
4312	0	0	0	3	3.8	4.81	12.4
4313	0	0	0	1	4.9	4.27	11.6
4314	0	0	0	2	9.3	4.22	12.1
4315	0	0	0	3	11.8	4.74	13
4316	0	0	1	1	7.8	4.76	12.4
4317	0	0	1	1	5.9	4.82	12.8
4318	0	0	1	1	4.8	4.12	12
4319	0	0	0	2	7.1	4.57	11.9
4320	0	0	0	3	13.3	5.08	13.7
4321	0	0	1	4	14	4.64	11.4
4322	0	0	0	2	20.4	4.35	13
4323	0	0	1	0	10.2	5.48	12.1
4324	0	0	0	1	2.4	4.62	12.5
4325	0	0	0	3	12.5	4.73	13.1
4326	0	0	0	1	4.8	4.28	11.3
4327	0	0	1	1	5.2	4.74	10
4328	0	0	0	3	5	4.57	12.8
4329	0	0	1	1	12.6	4.06	9.5
4330	0	0	0	1	11.6	4.76	12.4
4331	0	0	1	2	7.6	4.89	13.1
4332	0	0	1	0	15.9	3.63	10.2

4333	0	0	0	2	9.5	5.01	13
4334	0	0	1	4	5.7	3.57	10.5
4335	0	0	1	1	7.3	4.04	10.6
4336	0	0	1	3	9.4	4.7	12.4
4337	0	0	0	1	8.1	4.53	11.4
4338	0	0	1	4	6.3	4.62	12.8
4339	0	0	0	3	7.4	4.75	13.9
4340	0	0	0	2	2.9	4.45	11.9
4341	0	0	1	0	12.5	3.29	9.8
4342	0	0	0	2	4.6	4.74	12.5
4343	0	0	0	1	8.4	3.95	10.8
4344	0	0	1	1	8.2	3.83	10.5
4345	0	0	0	0	8.4	3.33	9.5
4346	0	0	1	1	5.2	4.4	10.4
4347	0	0	1	2	12.5	4.72	11.6
4348	0	0	1	1	9.2	4.54	11.8
4349	0	0	1	4	16.5	4.6	12.5
4350	0	0	0	1	7.3	4.19	13
4351	0	0	1	4	6.8	4.08	11.1
4352	0	0	0	1	9.2	4.86	14.5
4353	0	0	0	2	9.8	5.1	11.8
4354	0	0	1	1	6.5	4.52	11.8
4355	0	0	0	0	10.7	2.89	9.4
4356	0	0	0	0	12.9	3.71	10.5
4357	0	0	0	2	9.6	4.71	12.5
4358	0	0	0	1	8.1	3.99	10.5
4359	0	0	0	4	12.8	4.07	11.2
4360	0	0	0	0	15.7	2.92	9
4361	0	0	0	1	4	4.62	12.3
4362	0	0	1	4	13.7	4.33	12.2
4363	0	0	1	0	8	3.06	8.9
4364	0	0	0	0	13.7	3.19	9.9
4365	0	0	0	2	15.9	4.4	12.2
4366	0	0	1	1	15	4.95	12.6
4367	0	0	1	2	13.6	4.37	11.7
4368	0	0	1	1	4.4	4.49	10.5
4369	0	0	1	0	5.3	4.51	10.8
4370	0	0	1	1	6.3	4.63	12.7
4371	0	0	1	2	9.5	4.79	12.8
4372	0	0	1	1	13.9	4.58	11.6
4373	0	0	0	1	12	4.75	12.6
4374	0	0	1	3	6.5	4.55	12.2
4375	0	0	1	3	8.6	4.72	13.3
4376	0	0	0	2	9.8	4.34	11.7
4377	0	0	1	1	9.2	4.93	12.3
4378	0	0	0	1	12.6	4.58	11.7

4379	0	0	1	4	11	4.55	12.2
4380	0	0	0	1	10.4	4.04	11
4381	0	0	1	1	12.7	4.52	11.9
4382	0	0	1	0	15.6	3.16	9.4
4383	0	0	0	1	4.1	5.44	13.9
4384	0	0	0	1	13.8	4.54	12.8
4385	0	0	1	0	6	3.73	10.4
4386	0	0	0	0	24.3	3.94	10.4
4387	0	0	0	1	15.9	4.96	13.5
4388	0	0	1	0	11.5	4.34	11.2
4389	0	0	0	0	11.8	4.24	9.6
4390	0	0	0	0	3.5	3.86	11.2
4391	0	0	1	3	12.8	4.24	11
4392	0	0	1	1	10.8	4.69	11.9
4393	0	0	0	0	10.9	4.55	11.8
4394	0	0	0	1	12.3	4.59	12.2
4395	0	0	0	0	8.1	3.89	10.1
4396	0	0	0	1	16.6	4.3	11.5
4397	0	0	0	1	3.3	4.56	11.8
4398	0	0	0	3	6.5	4.34	12.1
4399	0	0	1	0	22	4.29	11.2
4400	0	0	0	0	13.2	4.51	13.6
4401	0	0	0	0	6.9	3.86	12.9
4402	0	0	0	2	14.6	4.75	12.8
4403	0	0	1	1	16.9	4.32	11.5
4404	0	0	1	1	13.8	4.93	12.8
4405	0	0	1	0	6.2	3.99	10.4
4406	0	0	1	0	13.8	4.74	15.7
4407	0	0	1	3	8.7	5.2	14.4
4408	0	0	1	1	3.2	4.43	12.2
4409	0	0	0	0	17.1	4.07	11
4410	0	0	1	4	7.3	4.3	12.2
4411	0	0	1	1	6.5	4.77	12.6
4412	0	0	1	1	7.4	4.22	10.8
4413	0	0	1	1	7.4	5.01	13.6
4414	0	0	0	0	12.8	3.69	12.6
4415	0	0	0	3	12.5	4.62	12.9
4416	0	0	1	0	21.9	4.19	11.4
4417	0	0	1	1	4.3	4.45	11.7
4418	0	0	1	0	8.7	4.02	10.6
4419	0	0	0	1	9.8	4.66	12.4
4420	0	0	1	4	7.7	5.17	13.8
4421	0	0	0	0	12	3.65	10.8
4422	0	0	0	1	6.5	4.48	12.6
4423	0	0	1	1	4.3	5.21	13.3
4424	0	0	1	3	4.9	5	13.8

4425	0	0	0	0	20.6	3.95	13.9
4426	0	0	0	2	8.8	4.38	12.3
4427	0	0	1	1	15.1	4.02	10.6
4428	0	0	1	0	10.5	3.68	10.7
4429	0	0	0	3	4.1	4.64	12.9
4430	0	0	1	1	5.2	5.02	13.4
4431	0	0	0	0	5.1	4.16	11
4432	0	0	0	1	11.2	4	10.6
4433	0	0	1	1	13.8	4.13	10.4
4434	0	0	0	1	5.3	4.69	12.2
4435	0	0	1	4	11.2	4.09	11
4436	0	0	0	0	5.1	3.31	10.6
4437	0	0	1	0	11.6	4.7	16.3
4438	0	0	0	1	6.1	4.71	12.4
4439	0	0	0	2	14.2	4.45	12.3
4440	0	0	0	1	6.8	4.68	11.7
4441	0	0	1	1	14.5	4.21	11.8
4442	0	0	1	1	11.2	4.74	12.5
4443	0	0	0	1	13.3	4	10.2
4444	0	0	0	0	6.3	3.24	9.3
4445	0	0	0	0	15.5	3.49	9.8
4446	0	0	1	1	3.2	4.45	11.6
4447	0	0	0	3	6.6	3.74	10.7
4448	0	0	1	1	10.7	4.47	10.7
4449	0	0	1	4	7.2	4.2	11.5
4450	0	0	1	3	5.6	5.07	13.2
4451	0	0	1	2	10.3	4.37	10
4452	0	0	0	1	21.7	4.18	10.9
4453	0	0	0	1	6.2	5.09	13.5
4454	0	0	0	1	6.1	4.91	12.9
4455	0	0	0	0	6.3	3.99	12.8
4456	0	0	0	1	5	4.29	11.5
4457	0	0	1	3	4.8	3.4	9.8
4458	0	0	1	2	5.7	5.15	12.8
4459	0	0	0	3	6.1	4.83	11.6
4460	0	0	1	1	10.4	4.61	12.5
4461	0	0	0	1	6.5	4.61	12.1
4462	0	0	1	1	3.2	4.4	11.6
4463	0	0	0	2	2.3	4.54	12.7
4464	0	0	0	0	8.3	4.07	11
4465	0	0	1	0	14	3.39	8.8
4466	0	0	1	0	10.7	4.15	10.2
4467	0	0	0	3	8.4	3.94	10.4
4468	0	0	0	0	16.8	4.33	12.1
4469	0	0	1	1	8.7	4.28	11.5
4470	0	0	1	0	14.7	3.16	9.3

4471	0	0	1	0	17.7	4.26	11.4
4472	0	0	1	0	10.6	2.99	9.2
4473	0	0	1	0	16.7	3.37	10.3
4474	0	0	0	1	1.6	1.7	4.1
4475	0	0	0	3	8.3	4.52	11.7
4476	0	0	1	4	4.3	4.67	12.4
4477	0	0	0	0	29.7	4.17	10.9
4478	0	0	1	0	6.5	4.4	11.6
4479	0	0	0	2	13.7	4.19	11.3
4480	0	0	1	1	21	4.41	11.5
4481	0	0	1	1	7.3	4.57	12.5
4482	0	0	1	1	38.7	4.17	10.3
4483	0	0	0	1	31	3.98	9.5
4484	0	0	1	1	5	4.69	11.5
4485	0	0	0	3	4.8	4.28	12
4486	0	0	1	1	8.7	4.87	12.7
4487	0	0	0	1	9.6	4.36	12.2
4488	0	0	0	0	7.1	4.49	11.9
4489	0	0	1	1	5.8	4.59	12
4490	0	0	1	0	11.2	4.82	15.5
4491	0	0	0	2	12.3	4.48	12
4492	0	0	0	3	4	4.64	12.3
4493	0	0	1	1	7.4	4.59	12.4
4494	0	0	1	1	12.9	4.65	11.6
4495	0	0	0	0	20.7	3.75	12.5
4496	0	0	0	0	20.6	3.94	11
4497	0	0	1	1	5.5	3.82	10
4498	0	0	1	3	7.7	4.52	12.4
4499	0	0	0	0	6.2	4.43	12.3
4500	0	0	1	1	10.9	4.85	12.8
4501	0	0	1	4	9.1	4.49	11.9
4502	0	0	0	2	6.4	4.58	13.1
4503	0	0	0	4	15.7	4.46	11.9
4504	0	0	0	3	10.3	4.67	12.6
4505	0	0	0	1	9.7	4.57	11.3
4506	0	0	0	1	15.2	4.45	12.5
4507	0	0	1	4	20.9	4.73	12.2
4508	0	0	1	1	5.9	4.83	12.9
4509	0	0	0	1	5	4.31	11.4
4510	0	0	0	2	7.9	4.7	11.1
4511	0	0	0	2	11	4.2	11.4
4512	0	0	0	1	6.5	4.53	12.8
4513	0	0	1	2	8.6	4.74	12.9
4514	0	0	1	0	13	4.73	10.4
4515	0	0	1	4	17.6	4.8	11.9
4516	0	0	1	0	6.7	3.49	10

4517	0	0	1	1	8.3	4.86	12.6
4518	0	0	1	3	6.9	4.15	10.3
4519	0	0	0	0	12.1	4.18	11.6
4520	0	0	1	0	18	4.46	11.9
4521	0	0	1	4	8.1	4.67	13.1
4522	0	0	0	0	17.1	4.81	12.5
4523	0	0	1	2	6.7	4.8	11
4524	0	0	1	0	13.6	3.26	9.9
4525	0	0	0	1	3.9	4.32	10.5
4526	0	0	0	0	4	4.04	10.8
4527	0	0	0	2	15.1	4.87	12.5
4528	0	0	0	1	12.7	4.83	13.6
4529	0	0	1	1	6	5.18	13.8
4530	0	0	0	3	24.3	4.6	12.5
4531	0	0	1	2	9.7	4.41	12
4532	0	0	0	1	18.1	4.35	11.7
4533	0	0	1	4	8.1	4.57	12.6
4534	0	0	0	1	7.2	4.71	12.3
4535	0	0	0	4	6	5.34	14.2
4536	0	0	0	3	9.4	4.03	11.5
4537	0	0	1	3	13.6	4.58	11.4
4538	0	0	0	2	11.1	4.34	12
4539	0	0	1	1	8.2	4.39	11.7
4540	0	0	1	1	14.6	4.75	13.2
4541	0	0	1	1	16.5	4.22	11
4542	0	0	1	2	17.9	4.73	13.2
4543	0	0	0	1	7.7	5.09	12.9
4544	0	0	0	1	17.8	4.51	11.9
4545	0	0	0	1	17.8	4.68	11.7
4546	0	0	1	1	9.2	5.06	13
4547	0	0	0	0	7.2	3.95	11.8
4548	0	0	1	4	5.1	4.37	12.1
4549	0	0	0	2	9.2	4.4	12
4550	0	0	1	1	13.2	4.82	11.9
4551	0	0	0	1	5.4	4.74	12
4552	0	0	1	0	3.2	3.52	10.9
4553	0	0	1	2	8.6	4.21	11.9
4554	0	0	0	0	14.4	3.44	10.5
4555	0	0	0	1	8.9	4.8	13.4
4556	0	0	0	1	5.9	4.8	10.4
4557	0	0	1	1	15.9	4.76	12.2
4558	0	0	0	1	6.5	4.42	12.1
4559	0	0	1	0	14.6	3.15	9.4
4560	0	0	1	0	8.3	3.87	10.6
4561	0	0	1	2	6.8	4.83	12.8
4562	0	0	0	1	12.2	4.29	11.5

4563	0	0	1	1	8.2	4.5	11.5
4564	0	0	0	0	13.5	3.65	10.1
4565	0	0	1	0	11.9	4.96	12.7
4566	0	0	0	1	3	4.68	12.8
4567	0	0	0	1	11.5	4.36	11.3
4568	0	0	0	3	8.7	4.58	13.7
4569	0	0	1	3	10.9	4.82	12.7
4570	0	0	0	1	15.1	4.9	11.2
4571	0	0	1	3	14	4.7	12.6
4572	0	0	0	4	11.9	5.06	13.8
4573	0	0	0	1	15.2	4.6	12.2
4574	0	0	1	1	6.8	4.51	11.7
4575	0	0	1	1	13.8	4.62	11.6
4576	0	0	1	2	11.7	4.97	13.5
4577	0	0	0	1	10.4	4.9	13.6
4578	0	0	1	1	6.2	4.68	12.4
4579	0	0	0	1	3.3	4.83	13.3
4580	0	0	0	4	9.4	4.79	13.1
4581	0	0	1	1	5.4	4.55	12.2
4582	0	0	0	2	7.2	4.55	11.9
4583	0	0	0	2	21.7	4.71	12.8
4584	0	0	1	4	4.3	4.34	12.1
4585	0	0	1	1	11.6	4.19	12
4586	0	0	1	1	5	4.76	11.6
4587	0	0	0	1	8.4	4.19	11.3
4588	0	0	0	1	14	4.9	12.6
4589	0	0	1	1	14.2	4.89	12.3
4590	0	0	0	1	5.4	4.26	12.1
4591	0	0	1	1	9.8	4.53	12.3
4592	0	0	1	1	5.8	4.37	11.9
4593	0	0	0	1	3.8	4.39	11.3
4594	0	0	1	4	10.2	4.77	10
4595	0	0	0	1	7.8	4.12	10.8
4596	0	0	1	1	10.4	4.43	12.3
4597	0	0	1	3	9.5	4.45	11.1
4598	0	0	0	1	20.6	4.67	12.7
4599	0	0	1	0	13.9	3.93	10.9
4600	0	0	1	2	6.4	4.54	12.3
4601	0	0	1	0	10	4.26	15.4
4602	0	0	1	1	6	4.93	13.3
4603	0	0	1	0	8	3.89	13.1
4604	0	0	0	2	5.5	4.44	11.7
4605	0	0	0	1	13.9	4.12	11.1
4606	0	0	0	1	12.2	4.02	11.1
4607	0	0	0	1	5.9	4.68	12.4
4608	0	0	0	1	4.9	4.4	12.2

4609	0	0	0	3	22.1	4.89	13.9
4610	0	0	1	3	7.3	5.24	13.6
4611	0	0	1	2	13.4	4.84	12.8
4612	0	0	0	1	14.6	4.7	12.4
4613	0	0	1	1	7.2	4.02	10.8
4614	0	0	1	1	14.8	4.06	10.3
4615	0	0	0	1	11.5	4.17	11.4
4616	0	0	0	2	10.5	4.93	13.2
4617	0	0	0	3	11.9	4.5	12.7
4618	0	0	1	3	4	4.3	12.3
4619	0	0	1	1	27.1	4.74	12.7
4620	0	0	1	1	6.6	3.87	10.9
4621	0	0	1	4	15.6	5	12.8
4622	0	0	1	1	21.1	4.76	13.1
4623	0	0	0	1	6.5	4.55	12
4624	0	0	1	0	10	5.01	12.9
4625	0	0	0	2	5.4	3.92	10.9
4626	0	0	0	3	10.4	4.5	12.2
4627	0	0	1	0	15.1	3.87	11.3
4628	0	0	1	3	5	5.08	10.1
4629	0	0	1	1	20.9	4.31	11.5
4630	0	0	1	0	8.6	3.82	11.5
4631	0	0	0	3	7.9	4.44	11.6
4632	0	0	0	1	9.5	4.56	11.9
4633	0	0	1	0	10.5	3.37	11
4634	0	0	1	0	11.3	3.34	9.7
4635	0	0	1	1	9.4	3.8	10.9
4636	0	0	0	0	20.9	3.67	10.4
4637	0	0	1	2	8.8	4.46	11.6
4638	0	0	0	1	3.4	4.23	12
4639	0	0	0	0	12	3.85	9.8
4640	0	0	0	0	9.4	4.68	16.4
4641	0	0	0	1	9.2	3.37	11.4
4642	0	0	1	0	6.8	4.24	15.1
4643	0	0	1	2	3.8	4.8	13.5
4644	0	0	0	2	4.8	5.1	11.9
4645	0	0	1	1	15	4.7	12.2
4646	0	0	1	0	8.3	3.95	13.7
4647	0	0	0	2	24.6	5.04	14.3
4648	0	0	1	0	22	4.48	11.8
4649	0	0	0	3	5.2	3.99	10.9
4650	0	0	1	1	4.1	4.53	12.5
4651	0	0	0	1	13.8	3.93	9.3
4652	0	0	1	2	4	4.65	12.2
4653	0	0	0	3	6.4	4.4	12
4654	0	0	0	1	4.6	4.58	12

4655	0	0	1	2	13.9	4.49	12.1
4656	0	0	0	0	5.8	3.05	9
4657	0	0	1	1	13.5	4.68	12.8
4658	0	0	1	0	19.6	3.23	9.6
4659	0	0	1	0	11.1	3.06	8.6
4660	0	0	0	3	4.1	4.22	11.2
4661	0	0	0	0	14.9	3.2	9.7
4662	0	0	1	2	19.2	4.31	12.2
4663	0	0	1	1	5.4	4.77	12.5
4664	0	0	1	2	8.5	4.39	12.2
4665	0	0	1	0	12.3	4.05	10.5
4666	0	0	1	1	5.3	4.72	13
4667	0	0	1	1	8.8	3.99	10.7
4668	0	0	1	0	26.2	3.44	10.5
4669	0	0	0	1	14.3	4.36	10.5
4670	0	0	1	1	6.7	4.27	11.6
4671	0	0	0	1	14.2	4.3	11
4672	0	0	1	0	26.5	4.25	11.1
4673	0	0	0	0	3.7	3.16	9.9
4674	0	0	1	1	5.1	5.25	12.8
4675	0	0	0	1	17.6	4.2	11.6
4676	0	0	0	1	6.5	4.17	11.7
4677	0	0	0	1	6.1	4.44	12.1
4678	0	0	0	1	10.2	4.53	12
4679	0	0	1	1	22.2	5.22	14.4
4680	0	0	0	1	3.9	4.14	11.9
4681	0	0	1	1	13.2	4.71	12
4682	0	0	0	3	5.8	4.56	12.9
4683	0	0	0	0	13.8	3.71	10.1
4684	0	0	0	3	20.7	4.27	11.3
4685	0	0	0	0	5.4	4.4	11.6
4686	0	0	1	0	12.1	4.75	11.9
4687	0	0	0	0	13	4.56	12.1
4688	0	0	1	0	7.9	4.93	12.1
4689	0	0	1	0	5.3	4.68	12.1
4690	0	0	0	0	4.4	4.69	9.8
4691	0	0	1	4	6.3	4.45	12.3
4692	0	0	1	2	11.6	4.64	12.6
4693	0	0	0	2	5.3	4.36	11.6
4694	0	0	1	2	4.6	4.31	11.9
4695	0	0	0	1	4.6	4.25	11.5
4696	0	0	1	1	3.8	4.05	11.1
4697	0	0	1	0	13.4	4.17	10
4698	0	0	1	1	7.9	4.67	12.9
4699	0	0	0	1	11.4	4.42	12.6
4700	0	0	0	1	9.7	4.17	10.7

4701	0	0	0	1	6.5	4.71	11.9
4702	0	0	0	1	10.7	4.72	12.7
4703	0	0	1	2	5.7	4.64	12.8
4704	0	0	1	2	24.9	4.41	11.7
4705	0	0	0	1	5.8	4.4	9.6
4706	0	0	1	3	5.5	4.84	13.2
4707	0	0	1	2	6.1	5.18	11.9
4708	0	0	1	4	6	4.69	12.3
4709	0	0	0	4	7.5	3.9	11
4710	0	0	1	2	4	4.6	12.5
4711	0	0	1	0	11.1	2.96	9.1
4712	0	0	0	1	8	4.37	11.6
4713	0	0	1	0	17.4	3.65	10.4
4714	0	0	1	1	15.5	4.63	11.6
4715	0	0	1	3	12.1	4.51	12.2
4716	0	0	0	0	6.9	4.63	12.6
4717	0	0	1	3	6.6	4.61	11.5
4718	0	0	1	0	13.6	3.95	12.7
4719	0	0	0	3	12.4	4.04	11.2
4720	0	0	1	1	24.7	4.49	11.9
4721	0	0	1	0	11	3.75	10.5
4722	0	0	0	1	7	4.21	10.8
4723	0	0	0	1	8.4	4.59	12.5
4724	0	0	0	3	18.3	4.52	11.5
4725	0	0	1	1	3.4	4.31	11.7
4726	0	0	0	1	4	4.25	11.6
4727	0	0	1	1	10	5.02	12.1
4728	0	0	0	1	1.9	4.28	11.5
4729	0	0	1	2	3.4	4.52	12.1
4730	0	0	1	4	6	3.9	10.1
4731	0	0	0	0	5	3.82	8.7
4732	0	0	0	1	4.7	4.56	12.2
4733	0	0	0	1	8.7	4.3	10.9
4734	0	0	1	3	3.4	4.4	11.7
4735	0	0	0	1	11.1	4.36	12.3
4736	0	0	1	1	2.6	4.35	11.7
4737	0	0	0	0	17.4	4.51	12
4738	0	0	0	1	6.3	4.5	12.3
4739	0	0	1	1	21.9	4.21	10.7
4740	0	0	1	1	14.1	4.31	11.5
4741	0	0	0	1	15.1	4.46	11.9
4742	0	0	1	1	10.2	3.92	10.8
4743	0	0	0	1	11.1	4.99	13.6
4744	0	0	0	2	16.4	5.07	14
4745	0	0	0	0	5.2	3.93	12.7
4746	0	0	0	1	7.6	4.6	12.5

4747	0	0	1	1	2.2	4.63	10.6
4748	0	0	1	3	8.6	4.04	10.7
4749	0	0	1	1	6.3	4.56	11.3
4750	0	0	1	1	5.5	4.49	10.3
4751	0	0	1	0	1.7	3.99	12.4
4752	0	0	0	0	6.1	4.44	12
4753	0	0	1	1	6.8	4.58	12.9
4754	0	0	1	0	8.5	3.15	8.4
4755	0	0	1	0	7.4	3.51	9.7
4756	0	0	0	4	10.5	4.12	11.9
4757	0	0	0	1	10.7	4.39	11.9
4758	0	0	0	1	12.6	4.37	11.6
4759	0	0	0	1	5.7	4.4	11.9
4760	0	0	0	1	13.2	4.5	12.4
4761	0	0	0	1	6	4.41	11
4762	0	0	0	0	26.9	4.08	11.8
4763	0	0	1	1	5.9	4.76	10.6
4764	0	0	0	2	17.4	4.15	10.4
4765	0	0	0	1	4.4	4.54	11.6
4766	0	0	0	3	3.7	4.39	12.5
4767	0	0	1	0	3.4	3.57	11.1
4768	0	0	0	2	4.7	4.77	12.4
4769	0	0	0	3	5.6	4.53	12.6
4770	0	0	1	2	7.2	4.28	11.4
4771	0	0	1	2	5.1	4.55	10.9
4772	0	0	0	2	11.8	4.44	12.3
4773	0	0	1	3	5.4	4.85	12.9
4774	0	0	1	2	4.6	4.57	12.7
4775	0	0	1	3	4.6	4.5	12.6
4776	0	0	1	2	7.7	4.93	12.6
4777	0	0	0	2	16.6	4.08	10.5
4778	0	0	1	3	12.7	5.39	13.1
4779	0	0	1	2	12	4.7	13.2
4780	0	0	1	4	19.1	5.12	13.4
4781	0	0	0	2	14.2	4.81	11.8
4782	0	0	0	2	9.9	3.95	8.5
4783	0	0	1	4	12.9	4.91	12.5
4784	0	0	1	2	18.9	4.66	13.2
4785	0	0	1	1	11.9	4.75	12.4
4786	0	0	1	0	12.6	3.47	9.2
4787	0	0	1	3	13.9	4.33	10.9
4788	0	0	1	3	5.1	4.61	11.9
4789	0	0	1	3	4.7	4.19	11.7
4790	0	0	1	4	2.7	4.96	13.2
4791	0	0	1	2	11.3	4.3	11.7
4792	0	0	1	1	5.3	4.21	8.7

4793	0	0	0	1	9.8	3.66	10.3
4794	0	0	1	1	5.2	4.18	11.8
4795	0	0	1	1	11.4	4.26	11.3
4796	0	0	1	2	12.8	4.75	11.5
4797	0	0	1	3	7.4	3.95	11.4
4798	0	0	1	4	16	5.32	14
4799	0	0	0	4	5.1	4.6	13.6
4800	0	0	0	4	14.7	4.43	11.9
4801	0	0	0	4	15.7	4.63	12.4
4802	0	0	1	4	11	4.47	12.4
4803	0	0	1	4	18.6	4.47	12.2
4804	0	0	1	4	2.6	4.58	12.5
4805	0	0	1	4	13.3	4.63	12.9
4806	0	0	1	4	10.8	4.77	13.1
4807	0	0	1	4	4.5	5	13.3
4808	0	0	0	4	10.1	4.77	13
4809	0	0	0	4	5.9	4.7	12.9
4810	0	0	0	4	4.7	5.2	13.9
4811	0	0	1	4	5.8	4.41	12.1
4812	0	0	0	4	6.8	4.74	13.1
4813	0	0	0	4	29.5	5.02	12.9
4814	0	0	0	4	13.2	4.64	13
4815	0	0	1	4	14.2	5.08	13
4816	0	0	0	4	10.7	4.59	12.2
4817	0	0	1	4	18.2	3.94	10.6
4818	0	0	0	4	22	5.59	15.9
4819	0	0	0	4	6	4.48	11.9
4820	0	0	1	4	15.1	4.19	11.7
4821	0	0	1	4	9.4	4.24	11.9
4822	0	0	1	4	13.5	4.82	13.6
4823	0	0	1	4	5.9	4.6	12.4
4824	0	0	1	4	8.8	4.38	12.2
4825	0	0	0	4	12.5	4.26	11.5
4826	0	0	1	4	10.5	4.31	12.2
4827	0	0	0	4	6.8	4.95	12.6
4828	0	0	1	3	7.5	4.56	12.1
4829	0	0	0	4	23	4.87	13.1
4830	0	0	0	3	7.1	4.78	13.1
4831	0	0	0	4	16.4	4.32	11.8
4832	0	0	1	4	22	4.78	13
4833	0	0	1	4	6.8	4.4	11.6
4834	0	0	0	4	14.1	4.71	13.3
4835	0	0	1	3	13.9	4.81	13.3
4836	0	0	1	4	11.1	4.61	12.6
4837	0	0	1	3	8.5	4.57	12
4838	0	0	0	4	13.2	4.96	13.1

4839	0	0	1	4	33.1	4.86	14.1
4840	0	0	0	4	11	4.71	12.7
4841	0	0	0	4	9.5	4.09	11.3
4842	0	0	0	4	19.4	4.56	12.9
4843	0	0	0	4	12.9	4.6	13
4844	0	0	0	3	12.2	4.15	11.3
4845	0	0	0	3	18.3	4.73	13.5
4846	0	0	1	4	16.2	4.47	13.1
4847	0	0	1	4	18.4	4.1	12.1
4848	0	0	0	4	8.5	4.73	13.5
4849	0	0	1	4	7.9	4.72	12.5
4850	0	0	1	3	20.3	4.84	13.3
4851	0	0	0	4	7	4.61	12.3
4852	0	0	0	4	5.1	5.32	14
4853	0	0	0	4	11.9	4.33	11.9
4854	0	0	0	3	8.2	4.58	13
4855	0	0	1	4	13.3	4.54	12.4
4856	0	0	0	3	12.4	4.62	12.5
4857	0	0	0	3	2.6	4.47	12.6
4858	0	0	1	3	11.8	4.05	12
4859	0	0	0	4	10.1	4.94	13.7
4860	0	0	1	4	15.9	4.89	13
4861	0	0	0	3	5.2	4.32	11.6
4862	0	0	0	4	19.4	5.11	14.6
4863	0	0	1	4	8.9	4.13	11.7
4864	0	0	1	4	5.4	4.79	12.2
4865	0	0	1	4	19.5	4.94	14.1
4866	0	0	1	3	9.1	4.75	12.9
4867	0	0	1	4	7.9	4.94	12.3
4868	0	0	0	4	14.7	5.12	14.5
4869	0	0	0	4	18.5	4.26	11.8
4871	0	0	1	4	14.1	4.57	11.4
4872	0	0	1	3	8.8	4.3	12
4873	0	0	0	4	11.5	4.76	13.3
4874	0	0	1	3	21.6	4.27	11.5
4875	0	0	1	3	22.1	4.94	12
4876	0	0	0	3	21.1	4.55	11.9
4877	0	0	0	3	25.6	4.38	11.6
4878	0	0	0	3	28.4	3.86	10.4
4879	0	0	1	4	45.5	4.54	12.1
4880	0	0	0	4	18	4.13	12.1
4881	0	0	1	4	9	4.55	12.4
4882	0	0	1	4	7.2	4.91	11.4
4883	0	0	0	4	11.8	4.39	11.7
4884	0	0	0	4	12.6	4.23	12
4885	0	0	1	3	8.7	4.57	11.9

4886	0	0	1	4	7.7	5.22	13
4887	0	0	0	3	21.5	5.09	13.6
4888	0	0	1	3	12.6	4.6	12.9
4889	0	0	1	4	9.7	5.1	14.8
4890	0	0	0	3	11.2	4.79	12.9
4891	0	0	1	3	21	5.07	12.9
4892	0	0	1	3	12.9	4.81	13.1
4893	0	0	1	3	20.5	4.44	12.8
4894	0	0	0	3	13.9	4.22	11.5
4895	0	0	0	4	23.3	4.58	12.3
4896	0	0	0	3	12	4.76	12.1
4897	0	0	1	3	18.2	4.34	11.6
4898	0	0	1	3	8.3	4.54	12.4
4899	0	0	0	2	7.3	4.18	11.1
4900	0	0	0	4	5.7	4.36	12
4901	0	0	1	3	16.5	4.34	13.1
4902	0	0	0	2	9.7	4.25	12.4
4903	0	0	1	3	9.1	4.74	12.6
4904	0	0	1	4	7.8	4.55	12
4905	0	0	1	2	5.4	4.47	12.7
4906	0	0	1	3	5.4	3.98	11
4907	0	0	1	3	6.4	4.46	12.5
4908	0	0	1	3	12.3	4.21	12.1
4909	0	0	0	4	13.2	4.68	12.1
4910	0	0	1	3	8.5	4.87	13.1
4911	0	0	0	2	9.6	4.55	12.7
4912	0	0	1	3	6.2	4.96	12.9
4913	0	0	1	3	7.7	4.34	12.1
4914	0	0	1	4	18.9	4.76	13.1
4915	0	0	0	2	9.4	4.29	11.5
4916	0	0	1	2	5.2	4.63	13.3
4917	0	0	1	3	11.7	4.96	13.3
4918	0	0	1	4	23.6	4.53	12.5
4919	0	0	1	2	7.7	4.37	12.4
4920	0	0	1	2	13.6	4.04	10.3
4921	0	0	0	2	15	4.43	11.8
4922	0	0	1	3	7.7	4.6	12.3
4923	0	0	1	3	7.6	4.77	12.9
4924	0	0	1	3	25.5	4.45	12.3
4925	0	0	1	3	19.1	4.84	13.4
4926	0	0	1	3	7.8	4.5	11.7
4927	0	0	1	4	12.3	4.81	13.1
4928	0	0	0	3	4.6	4.22	9.4
4929	0	0	1	3	7.6	4.67	12.1
4930	0	0	0	4	19.4	4.84	13
4931	0	0	1	4	20.4	4.69	12.2

4932	0	0	1	3	26.7	4.18	13.2
4933	0	0	1	2	8.5	4.53	12.2
4934	0	0	0	3	8.2	5.15	13.5
4935	0	0	1	3	17.9	4.19	11.6
4936	0	0	0	2	15.2	4.27	11.9
4937	0	0	0	4	8.3	4.55	12.8
4938	0	0	1	3	8.4	4.54	12.5
4939	0	0	0	2	10.8	4.46	12.1
4940	0	0	0	4	15.7	4.67	12.7
4941	0	0	1	3	16.8	4.81	13
4942	0	0	0	2	13.2	4.59	13
4943	0	0	0	4	8.7	5.09	13.9
4944	0	0	1	2	9.8	5.33	12.5
4945	0	0	0	2	12.7	4.09	11.2
4946	0	0	0	2	26.1	4.31	12.3
4947	0	0	0	1	3.6	3.5	10.1
4948	0	0	0	1	3.7	4.12	11.3
4949	0	0	1	2	14.8	4.76	11.7
4950	0	0	0	0	8.9	3.13	9.3
4951	0	0	1	2	21.6	4.79	12.8
4952	0	0	1	3	3.3	4.2	11.2
4953	0	0	1	4	12.9	4.06	11
4954	0	0	0	2	7.4	4.45	12.3
4955	0	0	1	2	15.2	4.32	11.3
4956	0	0	1	3	12.5	4.62	12.2
4957	0	0	0	2	10.6	4.97	11.7
4958	0	0	0	2	7.5	6.1	14.4
4959	0	0	0	3	14.7	4.89	12.5
4960	0	0	1	4	6	4.06	11.9
4961	0	0	0	3	6.1	4.37	12.2
4962	0	0	1	2	17.1	4.46	12.8
4963	0	0	0	4	16.5	3.65	10.4
4964	0	0	1	4	15	4.47	12.6
4965	0	0	1	2	6.9	4.5	11.8
4966	0	0	0	2	11.6	4.84	12.7
4967	0	0	1	3	5.8	4.51	12.5
4968	0	0	0	4	11.2	4.65	12.9
4969	0	0	1	3	7.8	4.5	12.1
4970	0	0	1	2	4.9	4.24	11.8
4971	0	0	0	2	14	4.06	10.7
4972	0	0	0	4	8.3	4.48	12.5
4973	0	0	0	2	10.3	4.57	12.3
4974	0	0	1	3	13.9	4.82	12.2
4975	0	0	1	2	11	4.22	11.6
4976	0	0	1	3	5.5	4.56	12.4
4977	0	0	0	2	9.7	4.91	13

4978	0	0	0	2	17.7	4.51	12.9
4979	0	0	0	4	7.3	4.77	12.7
4980	0	0	1	4	8.4	4.27	11.3
4981	0	0	1	2	13.5	5.17	13.2
4982	0	0	1	4	13.6	4.69	12.2
4983	0	0	1	2	13.2	5.16	13.2
4984	0	0	1	2	13.7	5	12.5
4985	0	0	1	4	11.1	4.75	13.3
4986	0	0	1	2	8.6	5.03	13.4
4987	0	0	0	2	4.3	4.34	12.1
4988	0	0	1	2	11.7	4.74	13.5
4989	0	0	1	3	8.6	4.25	11.9
4990	0	0	0	2	19.4	4.56	12.6
4991	0	0	1	3	9.4	5.01	12.9
4992	0	0	0	4	17	4.26	12.1
4993	0	0	0	3	9.8	4.27	11.6
4994	0	0	1	3	12.6	4.25	11.7
4995	0	0	1	4	17.2	4.58	11.9
4996	0	0	0	3	19	4.64	11.9
4997	0	0	1	2	14.7	5.11	14.1
4998	0	0	0	4	17.4	4.65	13.6
4999	0	0	0	2	10.7	4.71	13.3
5000	0	0	1	3	24.8	5.59	14.2
5001	0	0	1	1	9.8	4.52	12
5002	0	0	1	1	16.6	4.69	12.1
5003	0	0	1	2	10.8	3.99	10.7
5004	0	0	1	3	22.6	5.16	13.6
5005	0	0	1	1	19.2	5.38	14.6
5006	0	0	0	4	14.6	4.47	12.6
5007	0	0	1	3	8.7	4.65	11.4
5008	0	0	0	4	21.4	4.9	12.2
5009	0	0	1	1	7.1	4.5	12.5
5010	0	0	0	4	15.8	4.34	11.9
5011	0	0	1	4	8.1	4.71	12.4
5012	0	0	0	3	9	4.8	13.2
5013	0	0	1	2	8.7	5.26	13.4
5014	0	0	1	4	8.6	4.86	13
5015	0	0	1	2	14.1	4.37	11.3
5016	0	0	1	2	8.1	4.46	11.8
5017	0	0	1	3	9.1	4.5	12.4
5018	0	0	0	4	12.9	4.02	11.7
5019	0	0	0	4	15.4	4.36	11.4
5020	0	0	0	2	17.8	4.3	12.2
5021	0	0	1	2	33	3.88	10.4
5022	0	0	1	1	16.8	4.67	12.5
5023	0	0	0	3	20.5	4.19	11.9

5024	0	0	1	1	7.4	5.06	13.8
5025	0	0	1	2	10	4.03	11
5026	0	0	0	3	12.7	4.44	12.1
5027	0	0	1	1	4.7	4.95	12.5
5028	0	0	1	3	9.6	4.22	11.8
5029	0	0	0	2	8	4.61	11.9
5030	0	0	0	2	14.1	4.77	12.7
5031	0	0	1	1	15	4.5	12
5032	0	0	1	2	4.9	4.19	11.8
5033	0	0	0	2	7.6	5.11	14.8
5034	0	0	0	4	17.8	4.41	11.7
5035	0	0	0	4	4.8	4.48	12
5036	0	0	1	3	9.6	4.97	14.1
5037	0	0	1	1	7.8	4.72	12.8
5038	0	0	1	2	24.4	4.6	11.9
5039	0	0	0	3	10.2	4.75	12.6
5040	0	0	0	3	8.5	5.1	14.6
5041	0	0	0	1	12.6	5.19	14
5042	0	0	0	2	34	5.05	13.4
5043	0	0	0	2	10.3	4.78	13.3
5044	0	0	1	2	13.3	4.18	11.1
5045	0	0	1	2	12.8	4.56	12.2
5046	0	0	1	2	13.7	3.56	10.7
5047	0	0	1	4	12.5	4.32	11.8
5048	0	0	1	1	19.6	4.64	12.3
5049	0	0	1	4	9	4.75	13
5050	0	0	1	0	8.2	3.36	10.6
5051	0	0	1	4	9.6	4.39	12.6
5052	0	0	1	1	4.4	4.63	11.5
5053	0	0	1	1	10.6	4.14	11.7
5054	0	0	1	4	5.1	4.36	11.7
5055	0	0	0	3	4.5	4.94	13.2
5056	0	0	0	1	6.4	4.46	12.3
5057	0	0	0	3	22.5	5.09	12.2
5058	0	0	0	1	4.2	3.72	11.1
5059	0	0	1	3	6.4	4.89	12.3
5060	0	0	1	3	8.1	4.64	12.9
5061	0	0	1	4	16.3	4.74	13.5
5062	0	0	1	2	9.9	4.37	11.5
5063	0	0	0	2	15	3.77	9.7
5064	0	0	0	4	17.3	4.09	11.6
5065	0	0	1	3	8.3	4.55	11.9
5066	0	0	0	2	14.1	4.55	11.6
5067	0	0	0	4	18.2	5.36	11.9
5068	0	0	1	2	3.4	5.12	13.3
5069	0	0	1	4	13.8	4.23	11.6

5070	0	0	1	1	10.5	4.52	12.1
5071	0	0	0	4	8.6	3.97	11.2
5072	0	0	1	4	10.6	4.67	12.6
5073	0	0	0	1	6.3	4.44	11.9
5074	0	0	0	2	5.3	4.99	13.3
5075	0	0	0	2	7.4	4.72	13.2
5076	0	0	1	1	12.8	4.24	11.7
5077	0	0	0	4	17.1	4.62	11.5
5078	0	0	0	4	30.6	4.52	12.7
5079	0	0	0	4	18.5	4.63	12.3
5080	0	0	1	1	10.2	4.2	10.7
5081	0	0	1	2	5.5	4.69	12.8
5082	0	0	0	2	10.2	4.86	13.4
5083	0	0	1	4	9.8	4.85	13
5084	0	0	0	3	4.4	4.59	11.8
5085	0	0	0	2	10.4	4.41	12.2
5086	0	0	1	1	22.9	4.16	11.6
5087	0	0	1	3	9.5	4.4	11.8
5088	0	0	1	1	4.4	4.16	12.4
5089	0	0	1	2	8.6	4.81	13.4
5090	0	0	1	2	11.4	4.49	11.2
5091	0	0	0	4	12.6	4.91	13.6
5092	0	0	0	3	17.6	4.44	12.3
5093	0	0	1	3	20.1	4.66	12
5094	0	0	1	1	12.8	4.83	12
5096	0	0	1	1	4.3	4.66	13.3
5097	0	0	1	3	6	4.24	11.7
5098	0	0	1	1	5.5	4.35	11.9
5099	0	0	0	3	7.2	4.67	12.6
5100	0	0	1	2	6	4.88	11.9
5101	0	0	0	4	7.6	3.92	11.4
5102	0	0	1	1	8.3	4.21	11.3
5103	0	0	0	1	10	4.33	12
5104	0	0	1	1	4.1	4.63	12.2
5105	0	0	1	4	8.5	5.07	11.8
5106	0	0	0	2	15.8	4.98	12
5107	0	0	1	1	16.8	3.76	10.3
5108	0	0	1	3	13.1	4.88	11.5
5109	0	0	1	1	9	4.61	12
5110	0	0	1	2	9.6	4.61	12.3
5111	0	0	0	1	6	4.52	12.5
5112	0	0	0	4	9.2	4.7	12.5
5113	0	0	0	1	8.1	4.28	11.7
5114	0	0	1	4	9.8	4.64	13.3
5115	0	0	0	4	14.6	4.95	13.8
5116	0	0	1	4	11.4	3.98	10.9

5117	0	0	0	1	5.6	4.28	10.8
5118	0	0	0	1	16.8	4.04	10.5
5119	0	0	0	1	15.8	4.68	11.6
5120	0	0	1	3	5.6	3.97	10.5
5121	0	0	0	3	24.9	4.19	12.5
5122	0	0	1	3	25	4.58	11.9
5123	0	0	1	1	19.4	4.43	11.3
5124	0	0	0	4	7.5	4.53	13.1
5125	0	0	1	1	18.9	4.52	11.9
5126	0	0	0	2	16	4.67	12.7
5127	0	0	1	1	11.7	5.08	12.9
5128	0	0	0	4	10.7	4.16	11
5129	0	0	1	1	5	3.97	11.1
5130	0	0	0	4	8.4	5.42	14.9
5131	0	0	1	2	5.8	3.98	11.2
5132	0	0	0	2	11	4.9	11.9
5133	0	0	0	1	5.9	4.23	12.2
5134	0	0	1	1	4.1	4.76	12.4
5135	0	0	0	1	12.5	4.73	13.4
5136	0	0	1	4	5.3	4.5	12.3
5137	0	0	0	2	14.1	4.99	14.1
5138	0	0	1	4	6.8	3.64	10.2
5139	0	0	1	1	6.3	4.21	11.4
5140	0	0	0	4	6.6	4.58	12.7
5141	0	0	1	4	9.3	4.62	12.6
5142	0	0	1	1	28.9	3.99	10.7
5143	0	0	1	1	17.6	5.46	13.5
5144	0	0	1	2	5.9	5.09	13.5
5145	0	0	1	2	13.3	4.82	12.5
5146	0	0	1	2	7.2	4.46	12.4
5147	0	0	0	2	20.6	4.89	13.1
5148	0	0	0	1	3.4	4.45	12
5149	0	0	1	1	6.7	4.79	13.2
5150	0	0	0	1	8.6	4.99	12.6
5151	0	0	1	1	7.4	3.76	11.2
5152	0	0	1	3	7.8	5.15	13.5
5153	0	0	1	1	17.6	4.71	12.9
5154	0	0	1	2	24.8	3.79	10
5155	0	0	0	4	5.7	4.33	12
5156	0	0	0	3	19.7	4.8	10.8
5157	0	0	1	1	18.9	4.51	11.6
5158	0	0	0	2	10	4.7	12.5
5159	0	0	1	2	6	5.02	12.9
5160	0	0	0	1	9.8	3.28	8.4
5161	0	0	1	1	8.9	4.17	10
5162	0	0	1	1	8.3	4.18	11.2

5163	0	0	0	4	18.6	4.92	13.4
5164	0	0	0	4	28.4	3.77	11.4
5165	0	0	0	1	8.2	4.75	12
5166	0	0	1	2	6.9	4.79	13
5167	0	0	0	2	11.2	4.49	12.5
5168	0	0	1	1	20.3	4.07	10.3
5169	0	0	1	1	17.6	3.4	11.2
5170	0	0	0	2	11.4	4.55	12.3
5171	0	0	1	2	18.9	4.61	12.9
5172	0	0	1	2	15.2	4.6	12
5173	0	0	0	1	5.4	4.81	12.9
5174	0	0	1	1	12.8	4.66	12.7
5175	0	0	1	3	19.2	4.46	12.4
5176	0	0	0	3	11	4.31	12.3
5177	0	0	1	1	3	4.7	13.1
5178	0	0	0	1	11.9	4.77	12.3
5179	0	0	1	1	4.2	4.36	11.2
5180	0	0	0	2	14.9	5.16	13.6
5181	0	0	1	4	15.4	4.4	12
5182	0	0	1	1	21.7	4.63	12.2
5183	0	0	1	2	9.7	4.22	10.8
5184	0	0	1	4	14.8	4.66	12.9
5185	0	0	0	4	20.2	4.22	10.9
5186	0	0	0	4	17.1	4.41	12.4
5187	0	0	1	4	21.2	4.5	12.5
5188	0	0	0	1	7.2	4.51	11.7
5189	0	0	0	1	13.1	4.42	12.5
5190	0	0	1	0	22	3.82	11.3
5191	0	0	1	1	12.6	5.02	12.6
5192	0	0	0	1	20.7	4.35	11.5
5193	0	0	1	4	7.8	4.29	12
5194	0	0	1	4	19.5	4.28	11.8
5195	0	0	1	1	17.7	4.9	12
5196	0	0	1	4	11.6	4.42	12
5197	0	0	1	3	25.2	4.45	12.4
5198	0	0	1	1	4.8	4.26	11.2
5199	0	0	1	2	4.6	4.78	11.9
5200	0	0	0	3	6.3	4.75	13.2
5201	0	0	0	1	20.3	4.65	11.6
5202	0	0	0	0	20.6	4.17	11.2
5203	0	0	1	1	8.2	4.03	10.6
5204	0	0	1	3	9	4.79	13.2
5205	0	0	0	1	5	4.67	12.4
5206	0	0	1	0	22.8	5.4	13.5
5207	0	0	0	1	6.8	5.06	12.9
5208	0	0	0	4	11.2	5.1	15.3

5209	0	0	0	3	20.6	3.91	10.7
5210	0	0	1	1	10.3	5.06	12.2
5211	0	0	0	1	9.7	4.5	12.4
5212	0	0	1	4	8	4.49	12.2
5213	0	0	1	0	23	4.4	12.1
5214	0	0	0	4	16.2	4.5	11.6
5215	0	0	1	3	13.7	4.58	11.1
5216	0	0	1	4	17	4.96	13.6
5217	0	0	0	0	12.9	4.86	11.7
5218	0	0	1	1	4.2	4.63	11.7
5219	0	0	1	1	4.9	5.13	11.8
5220	0	0	0	1	9.2	5.17	14
5221	0	0	1	3	13.7	4.72	12.2
5222	0	0	0	2	12.5	4.82	12.6
5223	0	0	0	0	10.3	3.11	10.5
5224	0	0	1	2	21.5	5.17	11
5225	0	0	1	1	10.4	4.89	13.8
5226	0	0	1	3	9.1	5.06	13.5
5227	0	0	0	1	8	4.37	12.4
5228	0	0	1	4	8.4	4.8	12.8
5229	0	0	1	1	4.9	4.48	11.9
5230	0	0	1	2	12.1	4.96	12.3
5231	0	0	1	4	8.7	4.25	11.2
5232	0	0	0	4	6.6	4.2	11.4
5233	0	0	0	4	5.7	4.9	13.7
5234	0	0	1	1	2	3.29	9.3
5235	0	0	0	4	10.5	4.32	12.7
5236	0	0	1	1	12.6	4.32	11.6
5237	0	0	1	3	6	4.2	11.4
5238	0	0	1	4	7.3	4.81	11.9
5239	0	0	1	2	4.4	4.28	11.7
5240	0	0	1	3	32.8	4.44	11.9
5241	0	0	0	4	17.5	4.49	12.4
5242	0	0	0	1	8.6	4.6	12.5
5243	0	0	0	4	12	4.4	11.8
5244	0	0	0	3	19.9	4.08	11
5245	0	0	1	4	11.1	4.45	11.6
5246	0	0	0	1	3.8	4.72	13.1
5247	0	0	1	0	4.7	4.53	12
5248	0	0	0	1	7.3	4.56	12.6
5249	0	0	1	2	11.1	4.38	12.2
5250	0	0	1	0	6.5	4.3	11
5251	0	0	0	4	6.9	4.72	12.8
5252	0	0	1	1	3	4.62	12.3
5253	0	0	1	2	11.4	4.18	11.3
5254	0	0	1	0	21	4.4	11.2

5255	0	0	0	0	14.1	3.63	10.7
5256	0	0	1	0	18	4.41	11.6
5257	0	0	1	1	9.2	4.27	11.2
5258	0	0	1	2	9.6	4.7	12.7
5259	0	0	1	2	7.4	4.73	11.9
5260	0	0	1	2	24.6	5.17	11.3
5261	0	0	0	4	13.8	5.26	11.2
5262	0	0	1	3	16.1	5.48	16.7
5263	0	0	1	1	7.5	4.97	12.6
5264	0	0	0	1	9.7	4.16	11.5
5265	0	0	0	3	4.3	4.12	12
5266	0	0	1	4	4.3	4.19	11.2
5267	0	0	1	1	5.8	4.82	12.8
5268	0	0	1	0	26.8	3.83	10.7
5269	0	0	0	4	15.4	4.93	13.2
5270	0	0	1	2	10.9	4.73	11.7
5271	0	0	0	1	12.4	5.17	13.7
5272	0	0	1	4	5.8	4.99	13.8
5273	0	0	0	4	14.8	5.08	13.6
5274	0	0	1	2	11.1	4.41	12
5275	0	0	0	0	5.6	3.83	11.3
5276	0	0	1	0	25.5	5.13	13.3
5277	0	0	1	0	7.9	4.66	11.4
5278	0	0	1	4	15.3	5.18	13.5
5279	0	0	1	2	11	5.37	14.1
5280	0	0	0	4	5.2	4.78	13.2
5281	0	0	1	1	30.8	4.46	12.7
5282	0	0	1	4	7.5	5.01	12.5
5283	0	0	1	1	6.2	4.92	12.8
5284	0	0	0	0	16.2	4.24	11.9
5285	0	0	1	2	9.3	5.26	12.4
5286	0	0	1	4	12.7	4.47	12.5
5287	0	0	0	4	9.4	4.01	11.5
5288	0	0	0	4	15.7	4.29	11.2
5289	0	0	1	2	11.9	4.07	11.7
5290	0	0	1	2	18.8	4.24	11
5291	0	0	1	4	5	4.81	13.2
5292	0	0	1	2	9.6	4.62	11.9
5293	0	0	1	4	18.9	4.37	11.8
5294	0	0	0	4	14.6	4.58	12.8
5295	0	0	1	4	11.9	4.49	13
5296	0	0	0	2	14.1	4.37	12.3
5297	0	0	1	3	7.3	4.83	13.5
5298	0	0	0	2	5.5	4.31	12.2
5299	0	0	0	4	22.6	4.27	11.2
5300	0	0	0	2	18.4	4.39	11.3

5301	0	0	0	2	7.4	3.99	10.1
5302	0	0	0	2	9.8	4.36	12
5303	0	0	0	1	14.4	4.67	12.2
5304	0	0	1	1	18.4	4.65	11.7
5305	0	0	0	1	7.4	4.44	12.7
5306	0	0	1	1	7.3	4.66	12.5
5307	0	0	0	0	8.7	4.21	9.6
5308	0	0	1	4	13.3	4.57	12.1
5309	0	0	1	2	18.6	4.91	12.6
5310	0	0	1	4	11.1	4.66	13.7
5311	0	0	1	2	18.6	4.46	12
5312	0	0	1	4	3.7	4.73	11.3
5313	0	0	0	2	18	4.8	12.7
5314	0	0	0	2	13.2	4.3	12.3
5315	0	0	1	1	8.3	4.32	12.8
5316	0	0	1	4	18	4.88	13.2
5317	0	0	1	1	19.5	4.44	10.4
5318	0	0	1	1	26.7	4.49	10.5
5319	0	0	0	1	16.3	4.38	12.4
5320	0	0	1	1	8.4	4.58	11
5321	0	0	0	4	11.7	4.41	11.7
5322	0	0	1	2	11.5	4.84	12.5
5323	0	0	0	1	18.5	4.91	12.4
5324	0	0	0	4	13.6	4.59	11.8
5325	0	0	0	1	8.4	5.02	14
5326	0	0	0	4	6.6	4.41	11.6
5327	0	0	0	1	5	4.02	10.8
5328	0	0	1	2	18.8	5.19	12.6
5329	0	0	0	1	18.8	4.34	11.9
5330	0	0	0	4	7.4	3.75	10.7
5331	0	0	1	4	16.6	4.91	11.3
5332	0	0	1	0	17.1	4.6	13.8
5333	0	0	1	1	6.2	4.56	11.6
5334	0	0	1	2	15.3	4.85	12.3
5335	0	0	0	3	10.3	4.39	11.1
5336	0	0	0	2	16.3	4.25	11.5
5337	0	0	1	0	14.2	2.66	8
5338	0	0	1	4	4.4	4.28	10.9
5339	0	0	1	1	5.2	4	11.1
5340	0	0	1	0	13.1	3.73	11.1
5341	0	0	1	1	9.8	4.64	11.5
5342	0	0	1	3	17.1	4.91	13.7
5343	0	0	1	0	21.2	4.43	12.8
5344	0	0	1	1	9.4	4.27	10.2
5345	0	0	0	1	8.8	4.32	12.4
5346	0	0	1	0	15.8	4.4	10.7

5347	0	0	1	1	7	5.02	13.7
5348	0	0	0	3	17.8	4.4	12.4
5349	0	0	1	4	7.8	4.41	11.4
5350	0	0	0	4	7.3	4.62	12.8
5351	0	0	0	3	38.4	4.32	11.8
5352	0	0	1	3	7.9	4.82	13.3
5353	0	0	0	0	25.5	4.28	10.5
5354	0	0	1	1	6.2	4.36	10.6
5355	0	0	1	4	7.8	4.21	11.4
5356	0	0	0	4	8.8	4.6	11.9
5357	0	0	0	1	6.7	4.46	12.4
5358	0	0	0	1	13.6	4.1	11.1
5359	0	0	1	4	10.1	4.13	11.8
5360	0	0	1	2	21.5	4.73	11.6
5361	0	0	1	3	7.3	4.86	12.8
5362	0	0	1	1	8.9	5.21	13.2
5363	0	0	0	2	9.2	5	13.3
5364	0	0	1	4	6.2	4.33	11.9
5365	0	0	1	4	18.2	4.7	12.7
5366	0	0	0	2	20.2	4.94	12.8
5367	0	0	1	2	8.8	4.54	12
5368	0	0	1	4	5.2	4.04	10.9
5369	0	0	1	4	24.5	4.21	11.6
5370	0	0	0	2	2.9	3.88	11.4
5371	0	0	1	1	7.9	4.51	11.6
5372	0	0	1	4	20.4	4.3	11.9
5373	0	0	1	4	14.5	4.46	11.5
5374	0	0	0	4	19.2	4.17	11
5375	0	0	1	2	8.5	4.26	12.5
5376	0	0	1	3	5	4.77	13
5377	0	0	0	1	10.2	4.48	11
5378	0	0	0	4	36.1	4	10.5
5379	0	0	0	4	17.4	4.91	11.9
5380	0	0	1	4	8.9	4.26	11.5
5381	0	0	0	4	17	4.52	12.5
5382	0	0	0	1	9.2	4.5	11.2
5383	0	0	0	4	12.6	4.21	12.1
5384	0	0	1	4	10.3	4.57	12.3
5385	0	0	1	4	5.4	4.81	13
5386	0	0	0	2	13.3	4.8	11.8
5387	0	0	0	3	8.6	4.74	12.4
5388	0	0	0	4	11.7	4.67	13
5389	0	0	1	4	16.5	4.65	12.4
5390	0	0	1	2	8.1	4.1	10.7
5391	0	0	1	4	6.7	4.48	11.9
5392	0	0	0	3	16.8	3.7	10

5393	0	0	1	1	7.6	4.69	12.4
5394	0	0	1	4	4.1	4.88	13.1
5395	0	0	1	2	10.5	4.59	12.2
5396	0	0	0	4	9.5	4.82	14.4
5397	0	0	1	4	7.6	4.75	12.9
5398	0	0	1	4	5.3	4.42	12.8
5399	0	0	0	3	21.1	4.66	12.6
5400	0	0	0	4	4.3	4.43	11.7
5401	0	0	0	2	8.2	4.18	11.2
5402	0	0	1	3	13.1	4.83	12
5403	0	0	1	1	17.6	4.24	10.6
5404	0	0	0	2	25.6	3.92	10.8
5405	0	0	0	2	6.4	4.95	12.2
5406	0	0	0	2	14.6	4.38	11.4
5407	0	0	1	2	9.9	4.82	13
5408	0	0	0	4	5.3	4.21	12.4
5409	0	0	0	3	5.3	4.67	13.2
5410	0	0	0	3	30.2	4.7	13.7
5411	0	0	1	4	7.1	4.28	12.1
5412	0	0	1	4	32	4.95	13.5
5413	0	0	0	4	7.9	4.37	12.6
5414	0	0	1	2	6.4	4.22	11.7
5415	0	0	1	2	18.3	4.87	13.6
5416	0	0	0	4	15.2	3.95	10.5
5417	0	0	0	4	8.7	4.41	12.2
5418	0	0	1	4	31.5	4.1	10.4
5419	0	0	1	1	17.9	4.09	10.7
5420	0	0	1	3	18.4	4.63	12.7
5421	0	0	0	2	9.1	4.59	11.7
5422	0	0	0	2	16.9	4.69	12.5
5423	0	0	1	4	17.2	4.52	12.2
5424	0	0	0	3	9.3	4.57	12.1
5426	0	0	0	4	7	4.26	11.7
5427	0	0	0	4	8.1	4.35	12.5
5428	0	0	0	4	5.9	3.97	11.2
5429	0	0	0	2	10.5	5.15	13.5
5430	0	0	1	3	18	4.14	11.1
5431	0	0	1	3	4	4.7	12.8
5432	0	0	0	4	8.5	4.59	12.7
5433	0	0	0	2	31.1	3.87	10.2
5434	0	0	0	0	13.5	3.1	9.1
5435	0	0	0	4	12.4	4.66	12.3
5436	0	0	0	1	7.5	4.41	11.9
5437	0	0	1	1	10.4	4.54	11.9
5438	0	0	1	0	5.7	3.95	10.2
5439	0	0	1	3	9.8	4.34	12

5440	0	0	0	2	14.1	4.86	13.3
5441	0	0	0	1	24.5	3.52	10
5442	0	0	1	2	13.3	4.64	13
5443	0	0	1	4	7.8	5.13	12.1
5444	0	0	1	4	6.6	4.38	12.3
5445	0	0	1	1	10.6	4.72	12.1
5446	0	0	1	2	10.2	4.63	11.2
5447	0	0	0	4	8.9	4.84	13.8
5448	0	0	1	4	15.5	4.34	12
5449	0	0	1	3	8.2	4.7	12.6
5450	0	0	0	1	18.4	5.4	12.4
5451	0	0	1	4	16.2	5.09	13.2
5452	0	0	1	4	13.5	3.65	9.9
5453	0	0	0	1	10.7	4.42	12.4
5454	0	0	1	2	10.8	4.11	10.4
5455	0	0	1	3	3.9	4.58	13.1
5456	0	0	1	2	13.2	4.12	11.4
5457	0	0	0	4	4.9	4.56	11.7
5458	0	0	0	4	10.5	4.64	13.1
5459	0	0	1	3	8.6	4.87	12.8
5460	0	0	1	4	15.9	4.91	12.3
5461	0	0	0	4	4.5	4.47	12.4
5462	0	0	1	4	2.1	4.74	13.6
5463	0	0	1	4	17.5	4.22	11.2
5464	0	0	0	3	7.7	4.09	10.9
5465	0	0	1	1	3.3	5.05	13.5
5466	0	0	0	4	17.9	5.33	14.7
5467	0	0	1	3	12.6	4.71	12.5
5468	0	0	0	4	12.3	4.46	12
5469	0	0	1	4	4.7	4.47	12
5470	0	0	0	3	23.7	4.92	12.8
5471	0	0	0	2	18.1	4.13	10.8
5472	0	0	1	4	6	4.19	12
5473	0	0	0	1	6.9	4.67	13.2
5474	0	0	0	0	12.6	3.39	10.7
5475	0	0	0	4	6.3	4.29	12
5476	0	0	1	4	5.1	4.73	12.9
5477	0	0	1	1	8.8	4.45	12.4
5478	0	0	0	4	7.1	4.89	12.5
5479	0	0	0	0	16.1	4.04	10.3
5480	0	0	1	2	13.2	4.66	11.2
5481	0	0	0	4	6.5	4.74	12.6
5482	0	0	1	2	15.4	4.59	11.8
5483	0	0	0	4	12	4.63	12.8
5484	0	0	1	1	6	4.32	10.6
5485	0	0	0	4	9.1	4.78	13.2

5486	0	0	1	1	30.7	4	10.2
5487	0	0	1	1	11.1	3.95	11.2
5488	0	0	1	3	14	5.12	13.5
5489	0	0	0	3	10.6	4.71	12.5
5490	0	0	1	2	19.7	4.37	11.2
5491	0	0	0	2	8	4.5	12.5
5492	0	0	1	4	4.4	4.79	13.1
5493	0	0	0	2	10.9	4.56	11.9
5494	0	0	1	3	6.9	4.4	11.9
5495	0	0	0	4	13.5	4.79	13.4
5496	0	0	1	0	16.4	3.65	10.3
5497	0	0	1	4	11.1	4.34	10.4
5498	0	0	1	2	7.7	4.56	12.4
5499	0	0	0	0	9.5	4.29	11.7
5500	0	0	0	4	10.2	4.3	12.1
5501	0	0	1	3	9.8	5.09	12.7
5502	0	0	0	1	15.7	5.03	13.4
5503	0	0	0	0	12.1	4.89	12.6
5504	0	0	0	1	9	5.03	11.7
5505	0	0	0	0	6.3	4.95	12.3
5506	0	0	1	2	3.1	4.79	12.4
5507	0	0	1	3	12	3.94	10.9
5508	0	0	1	1	17.8	4.81	11.7
5509	0	0	0	3	7.1	4.74	12.8
5510	0	0	1	3	11.6	4.15	10.8
5511	0	0	1	3	4.6	4.6	12.2
5512	0	0	0	1	18.3	4.84	11.7
5513	0	0	1	2	3.9	5.3	13.3
5514	0	0	0	4	4.7	4.18	11.5
5515	0	0	1	1	20.2	4.6	11.5
5516	0	0	0	2	13.4	4.67	12
5517	0	0	1	2	7.7	4.3	11.9
5518	0	0	1	1	13.3	4.93	13.1
5519	0	0	0	0	15.5	4.57	12.9
5520	0	0	0	2	5.3	4.21	11.4
5521	0	0	1	0	13.5	4.49	10.9
5522	0	0	1	3	15.4	4.29	11.2
5523	0	0	0	0	12.2	4.47	9.5
5524	0	0	0	3	16.1	4.2	11.4
5525	0	0	1	4	6.2	4.7	13.3
5526	0	0	1	2	2.8	5.82	14
5527	0	0	1	2	12.4	5.2	13.3
5528	0	0	0	2	7.8	4.47	12.4
5529	0	0	0	1	5.5	4.46	12.3
5530	0	0	1	4	9.8	4.09	11.4
5531	0	0	1	3	13.5	4.17	11.5

5532	0	0	1	3	17.8	5.07	11.9
5533	0	0	1	3	17.6	4.53	13.2
5534	0	0	1	0	8.3	2.64	7.5
5535	0	0	1	4	12.6	4.65	12.1
5536	0	0	0	3	18.6	4.42	12.7
5537	0	0	1	2	4.4	4.25	11.9
5538	0	0	0	3	4	4.43	11.9
5539	0	0	0	4	6.9	4.74	13
5540	0	0	0	4	6.2	4.88	13.5
5541	0	0	1	0	7.7	4.52	12.2
5542	0	0	1	2	10.6	4.31	11.5
5543	0	0	1	0	11.9	4.06	11.1
5544	0	0	1	3	6.5	4.94	12.9
5545	0	0	0	0	9.8	4.92	12.6
5546	0	0	1	1	5.2	4.39	12.4
5547	0	0	1	4	8.1	5.13	11.6
5548	0	0	0	3	10.5	4.79	12.7
5549	0	0	0	3	7	5.2	13.9
5550	0	0	0	3	11.8	5.25	12.7
5551	0	0	1	3	10.9	4.38	11.9
5552	0	0	1	2	6.4	5.32	13.7
5553	0	0	1	1	15.7	4.76	11.8
5554	0	0	1	4	16.2	4.32	12.5
5555	0	0	1	4	9.2	4.33	11.6
5556	0	0	1	0	10.3	4.04	11.1
5557	0	0	0	0	13.9	4.08	10.6
5558	0	0	0	3	9.3	4.49	13.2
5559	0	0	0	4	4.8	4.37	11.6
5560	0	0	1	2	6.5	4.77	13.1
5561	0	0	1	3	8.3	5.43	14.8
5562	0	0	0	3	8	4.62	12.5
5563	0	0	0	4	7.7	4.47	12.3
5564	0	0	1	1	21.4	4.89	11.9
5565	0	0	1	4	21	6.18	18.5
5566	0	0	1	4	10.7	4.34	11.5
5567	0	0	0	3	5.3	4.85	13.4
5568	0	0	1	4	16.6	4.81	12.8
5569	0	0	1	2	15.3	4.91	12.8
5570	0	0	0	2	11.3	4.07	11.1
5571	0	0	0	1	19.4	4.1	10.7
5572	0	0	1	2	14	5.1	12.9
5573	0	0	0	3	6.7	4.49	12.3
5574	0	0	0	2	13.5	4.36	11.7
5575	0	0	1	4	6.3	4.56	11.9
5576	0	0	0	3	11.2	4.19	12
5577	0	0	1	3	8.6	4.12	10.4

5578	0	0	1	4	11.9	4.99	12.9
5579	0	0	0	3	5.8	4.24	12.1
5580	0	0	0	1	13.7	4.4	11.6
5581	0	0	1	2	13.2	4.49	12.5
5582	0	0	1	3	14.3	4.78	13.5
5583	0	0	1	3	12.5	4.16	11
5584	0	0	0	3	20	4.16	11.6
5585	0	0	1	2	2.8	4.65	11.6
5586	0	0	1	4	5.6	4.27	11.6
5587	0	0	0	3	12.2	3.76	10.8
5588	0	0	1	2	6.8	4.54	11.3
5589	0	0	1	4	5.3	3.95	11.7
5590	0	0	1	2	22	4.03	11.1
5591	0	0	1	4	4.8	4.62	11.3
5592	0	0	1	0	7.3	2.92	8.8
5593	0	0	0	4	6.8	4.18	11.6
5594	0	0	1	1	15.1	4.79	12.8
5595	0	0	1	4	18.9	4.92	13.4
5596	0	0	0	3	16.4	4.18	11.3
5597	0	0	0	3	7.9	4.35	10.9
5598	0	0	1	2	8.9	4.48	12.5
5599	0	0	1	4	13.4	4.47	11.9
5600	0	0	0	3	10.5	4.02	11.8
5601	0	0	0	4	7.7	4.23	12.5
5602	0	0	0	0	25.6	4.72	12.5
5603	0	0	1	3	6	4.59	12.6
5604	0	0	0	4	5.2	4.61	12.3
5605	0	0	1	3	26.8	4.37	11.6
5606	0	0	0	3	10.2	4.51	12.4
5607	0	0	1	4	6.4	4.85	14.3
5608	0	0	1	2	7.3	4.11	11.2
5609	0	0	0	3	13.1	4.55	12.4
5610	0	0	1	3	13.7	5.39	13.6
5611	0	0	1	0	9.8	3.63	9.8
5612	0	0	1	3	7.7	4.46	12.2
5613	0	0	0	3	11.3	3.33	9.7
5614	0	0	0	4	14.7	4.4	12.8
5615	0	0	0	1	12.9	3.89	10.9
5616	0	0	1	4	15	5.17	14.1
5617	0	0	1	3	15.9	5.13	13.8
5618	0	0	1	4	10.6	4.91	13.4
5619	0	0	0	3	10.6	4.93	11.9
5620	0	0	1	4	11.1	4.38	11.9
5621	0	0	1	4	15.3	4.37	11.9
5622	0	0	0	4	12.4	4.29	12
5623	0	0	0	4	12	4.81	13.2

5624	0	0	1	4	8.1	4.08	10.4
5625	0	0	1	3	8.1	4.8	13.1
5626	0	0	0	3	7.3	5.03	13.2
5627	0	0	1	3	12.6	4.7	12.9
5628	0	0	0	4	7.3	4.39	12.5
5629	0	0	0	3	6.8	4.47	12.7
5630	0	0	0	4	35.1	4.5	12.2
5631	0	0	1	4	27.2	4.73	13.2
5632	0	0	1	1	20.6	4.89	12.5
5633	0	0	1	4	13.2	4.43	11.9
5634	0	0	1	3	8.7	3.86	11.2
5635	0	0	0	4	18.5	4.37	12.4
5636	0	0	1	3	13.8	4.21	11.8
5637	0	0	1	4	12.8	4.26	11.1
5638	0	0	0	1	18	4.66	12.8
5639	0	0	0	1	14.3	5.09	12.7
5640	0	0	0	2	7.8	4.2	11
5641	0	0	1	2	9.7	4.86	12.2
5642	0	0	0	4	7.8	4.75	13.5
5643	0	0	0	0	18.9	4.15	11.6
5644	0	0	0	3	5.1	4.79	13.8
5645	0	0	1	4	5.7	4.34	11.9
5646	0	0	1	4	5.3	4.63	12.7
5647	0	0	0	4	5.7	4.22	12.5
5648	0	0	1	4	11.4	4.63	12.6
5649	0	0	1	4	6	4.56	12.5
5650	0	0	0	3	5.7	4.34	12.2
5651	0	0	1	3	9.6	4.63	12.7
5652	0	0	1	2	9.8	4.77	12.6
5653	0	0	1	2	6	4.42	11.7
5654	0	0	1	3	24.1	4.55	12.5
5655	0	0	1	3	15.2	4.82	13.1
5656	0	0	0	3	8.4	4.43	12.5
5657	0	0	1	1	8.2	3.78	11.4
5658	0	0	0	4	9.3	4.7	12.9
5659	0	0	1	3	7.2	4.97	12.6
5660	0	0	1	4	6	4.56	12.7
5661	0	0	1	2	12.1	4.13	10.6
5662	0	0	0	3	8.4	4.04	11.3
5663	0	0	1	4	10.3	4.69	12.1
5664	0	0	0	0	6.7	4.25	11
5665	0	0	0	1	16.5	4.44	11.2
5666	0	0	0	4	13.5	4.83	12.6
5667	0	0	1	3	8.4	4.42	13.3
5668	0	0	1	2	7.1	4.18	10.5
5669	0	0	0	3	28.5	4.73	11.8

5670	0	0	0	3	5.3	4.57	12
5671	0	0	0	1	26.7	3.93	10.4
5672	0	0	0	3	9.4	4.14	10.2
5673	0	0	1	1	3.8	4.41	11.1
5674	0	0	0	2	34.4	4.5	11.8
5675	0	0	1	3	7.3	4.32	12.5
5676	0	0	1	3	12.2	4.91	12.1
5677	0	0	1	1	21.7	4.62	11.7
5678	0	0	1	4	9.8	4.52	12.3
5679	0	0	1	3	7.1	4.57	12.6
5680	0	0	0	3	6.9	4.32	12
5681	0	0	1	1	17.1	4.56	12.8
5682	0	0	0	4	7.2	3.94	11.6
5683	0	0	0	1	3.2	3.95	10.4
5684	0	0	0	3	8.3	4.57	12.7
5685	0	0	0	2	19.4	4.19	11.7
5686	0	0	0	4	9.7	4.46	12.4
5687	0	0	0	3	6.9	4.75	12.3
5688	0	0	1	1	22.1	5.35	14.2
5689	0	0	1	3	8.7	4.14	11.6
5690	0	0	1	4	28.1	4.75	12.3
5691	0	0	1	3	4.7	4.71	12.8
5692	0	0	0	2	14.5	4.65	12.2
5693	0	0	0	2	10	4.43	12.3
5694	0	0	0	3	12.6	4.32	11.5
5695	0	0	0	3	6.1	4.4	10.7
5696	0	0	0	4	6.6	4.46	12.5
5697	0	0	0	4	4.8	5	13.8
5698	0	0	1	3	5.6	4.28	11.7
5699	0	0	0	3	5.6	3.9	10.9
5700	0	0	0	3	6.3	4.69	12.8
5701	0	0	1	3	18.4	4.48	12.9
5702	0	0	0	4	3.8	4.79	12.9
5703	0	0	0	3	22.5	4.65	12.7
5704	0	0	0	4	16.4	4.19	11.9
5705	0	0	1	4	7.1	4.64	12.7
5706	0	0	1	3	3.6	4.64	12.4
5707	0	0	0	2	15.1	4.83	12.7
5708	0	0	1	1	22.2	4.77	12.3
5709	0	0	1	3	5.9	4.26	11.6
5710	0	0	0	4	18	5.1	13.2
5711	0	0	0	3	4.1	4.33	12
5712	0	0	0	3	3.9	4.15	11.1
5714	0	0	1	2	22.4	5.48	12.1
5715	0	0	0	2	9.5	4.71	12.4
5716	0	0	1	2	4.6	4.74	12.5

5717	0	0	1	3	24	5.41	12.6
5718	0	0	1	3	21.1	4.45	12.3
5719	0	0	0	3	16.7	4.32	12.3
5720	0	0	0	3	14.1	4.19	11.2
5721	0	0	0	3	13.1	4.34	12.4
5722	0	0	0	3	15.2	4.24	12.2
5723	0	0	1	2	16.5	4.41	11.7
5724	0	0	0	2	3.2	4.32	12.1
5725	0	0	0	1	7.7	4.43	12.3
5726	0	0	1	4	7.1	4.82	13.8
5727	0	0	1	4	7.8	4.31	12.2
5728	0	0	0	3	15.7	4.81	12.4
5729	0	0	1	3	7	4.26	12.1
5730	0	0	1	4	5	5.09	14.4
5731	0	0	1	4	5	5.36	14.5
5732	0	0	1	1	19.6	4.04	11.2
5733	0	0	0	3	8.5	4.36	12
5734	0	0	0	1	13.2	4.74	11.2
5735	0	0	1	4	14.5	5.61	15.3
5736	0	0	1	4	3.8	4.86	12
5737	0	0	1	3	13.1	4.12	11.2
5738	0	0	0	4	3.1	5.03	14
5739	0	0	1	3	13	4.35	11.7
5740	0	0	0	3	13.8	4.3	11.6
5741	0	0	0	3	9.7	4.83	12.3
5742	0	0	0	3	13.1	4.37	11.7
5743	0	0	1	1	15.8	4.85	11.2
5744	0	0	0	4	9.2	4.69	12.7
5745	0	0	0	0	3.1	3.67	12
5746	0	0	0	4	11.6	3.87	11.1
5747	0	0	1	4	20.9	4.23	11.3
5748	0	0	1	2	22.1	4.26	11.9
5749	0	0	0	4	12.8	4.3	12.1
5750	0	0	1	2	7.5	4.52	10.9
5751	0	0	1	4	3.6	4.44	11.2
5752	0	0	1	2	10.1	4.31	11.4
5753	0	0	0	2	16.6	3.63	10.2
5754	0	0	1	1	12.8	4.69	12.3
5755	0	0	0	3	5.6	4.77	12.7
5756	0	0	1	2	7.4	4.7	12.9
5757	0	0	0	4	27.9	4.41	12.1
5758	0	0	0	3	10.5	4.53	12.7
5759	0	0	1	1	8.1	4.56	11.3
5760	0	0	1	2	7.6	4.58	12.6
5761	0	0	1	3	13.4	5.15	14.1
5762	0	0	1	3	5.8	4.13	11.1

5763	0	0	0	1	4.4	4.81	12.7
5764	0	0	1	3	4.3	4.08	10.7
5765	0	0	0	3	6.7	4.49	11.2
5766	0	0	0	3	20.8	4.47	12.2
5767	0	0	0	3	6.9	4.7	12.7
5768	0	0	0	3	9.8	4.19	12.2
5769	0	0	0	1	6.1	4.26	10.7
5770	0	0	1	4	9.4	4.38	12.1
5771	0	0	1	3	8.3	4.55	12.7
5772	0	0	0	4	23.5	4.23	12.1
5773	0	0	1	2	7	4.66	12.7
5774	0	0	0	4	2.7	4.17	11.5
5775	0	0	0	2	9.2	4.69	12.8
5776	0	0	1	4	6.4	4.25	12.1
5777	0	0	0	2	6.9	4.38	12.1
5778	0	0	0	4	8.2	4.95	12.6
5779	0	0	1	3	3.2	4.2	11.2
5780	0	0	1	3	35.9	4.07	10.9
5781	0	0	1	2	14.1	4.66	11
5782	0	0	0	2	16.5	4.86	13.3
5783	0	0	0	2	13.1	4.58	12.9
5784	0	0	0	4	13.6	4.78	13.8
5785	0	0	1	3	18.8	4.29	11.4
5786	0	0	1	4	10.1	4.83	12.9
5787	0	0	0	3	5.1	4.05	11.4
5788	0	0	0	4	7.5	5.81	16.6
5789	0	0	0	2	7	4.73	12.7
5790	0	0	1	3	15.8	4.4	13
5791	0	0	1	3	9.5	4.38	12.2
5792	0	0	0	4	7.7	4.09	11.2
5793	0	0	1	2	18.1	4.59	12
5794	0	0	1	4	5.6	4.08	11.5
5795	0	0	1	4	6.9	4.49	11.6
5796	0	0	1	3	10.3	5.05	12.9
5797	0	0	0	2	6.2	4.64	11.6
5798	0	0	1	3	15.9	4.34	12.1
5799	0	0	0	4	13	4.39	11.9
5800	0	0	1	4	4.1	4.24	11.7
5801	0	0	1	3	5.8	4.65	12.5
5802	0	0	1	4	8	4.54	12.7
5803	0	0	1	4	6.3	4.14	10.7
5804	0	0	0	3	3.8	4.57	11.9
5805	0	0	0	2	25.3	3.63	10.1
5806	0	0	1	4	3.6	4.53	11.2
5807	0	0	1	3	28.9	4.68	12.6
5808	0	0	1	2	2.2	4.12	10.4

5809	0	0	1	4	3.3	5.21	13.6
5810	0	0	1	3	10	4.58	10.4
5811	0	0	0	2	6.7	4.24	11.3
5812	0	0	0	2	15	4.33	11.9
5813	0	0	1	2	7.6	4.46	11.4
5814	0	0	0	4	5.2	4.45	11.9
5815	0	0	0	1	11.5	4.32	11
5816	0	0	1	2	7.1	4.38	10.5
5817	0	0	0	3	6.3	4.14	11.3
5818	0	0	1	2	18.6	4.18	11.2
5819	0	0	1	2	3.7	3.92	11.2
5820	0	0	1	4	4.7	3.94	10.9
5821	0	0	0	3	3.8	4.38	11.5
5822	0	0	1	3	16.1	4.37	12.4
5823	0	0	0	2	6.5	4.62	12.6
5824	0	0	0	3	5.2	4.74	12.3
5825	0	0	0	2	8.9	4.44	11.9
5826	0	0	1	2	13	4.26	11.3
5827	0	0	0	2	7.5	4.48	12.4
5828	0	0	0	3	7.5	4.57	12
5829	0	0	0	2	9.8	4.47	11.2
5830	0	0	0	2	18.6	4.35	11.8
5831	0	0	1	4	4.5	4.98	14.5
5832	0	0	1	3	11.3	4.38	11.7
5833	0	0	1	3	9.3	4.17	11
5834	0	0	1	2	4.3	4.51	11.8
5835	0	0	1	4	7.8	4.52	12.2
5836	0	0	0	4	20.8	4.31	12.5
5837	0	0	0	2	8.6	4.35	11.8
5838	0	0	1	2	9.1	4.07	12
5839	0	0	0	2	17	4.01	11.1
5840	0	0	1	3	9.8	4.56	12
5841	0	0	0	3	19.6	4.64	12.3
5842	0	0	1	2	13.4	4.94	12.8
5843	0	0	0	1	17.5	4.15	10.3
5844	0	0	0	3	17.2	4.95	13.8
5845	0	0	0	3	21.1	5.23	14.4
5846	0	0	1	2	3.7	4.28	12.5
5847	0	0	1	2	4.6	4.77	12.5
5848	0	0	1	2	2.8	4.28	11.5
5849	0	0	0	4	6.7	4.65	12.3
5850	0	0	1	4	5.2	4.47	11.9
5851	0	0	1	2	7.6	4.61	12.3
5852	0	0	1	2	0.19	3.84	9.3
5853	0	0	0	3	9.3	4.38	12.4
5854	0	0	0	2	4.7	4.07	11

5855	0	0	1	3	11.9	4.99	13.2
5856	0	0	1	2	11.4	4.79	13.2
5857	0	0	0	1	4.3	3.71	10.9
5858	0	0	1	1	8	4.61	12.1
5859	0	0	1	2	8.4	4.18	10.7
5860	0	0	1	2	9.7	4.97	13.8
5861	0	0	1	1	6.8	3.92	10.6
5862	0	0	1	1	18.1	4.38	11.7
5863	0	0	1	2	4.1	5.29	13
5864	0	0	0	3	9.1	4.26	12.2
5865	0	0	1	3	10.1	4.47	12.4
5866	0	0	1	1	14.4	2.79	8.2
5867	0	0	1	2	10.1	4.63	12.6
5868	0	0	1	2	7.4	4.59	11.5
5869	0	0	1	2	7.8	3.66	9.9
5870	0	0	0	2	15.8	4.28	12.7
5871	0	0	1	3	7.9	4.51	11.1
5872	0	0	1	1	10.1	4.04	9.3
5873	0	0	0	2	4.4	4.76	12.3
5874	0	0	0	2	19.2	3.92	10.5
5875	0	0	1	2	12	4.44	11.3
5876	0	0	1	2	9.6	4.84	12.9
5877	0	0	1	3	4.2	4.36	12.4
5878	0	0	0	3	9.2	5.24	13.7
5879	0	0	1	2	16.5	4.4	11.7
5880	0	0	0	2	3	4.48	12.3
5881	0	0	1	2	8.3	4.5	12
5882	0	0	1	2	8.6	4.6	11.9
5883	0	0	0	2	14.1	4.73	12.8
5884	0	0	1	3	15.2	4.62	12.6
5885	0	0	0	2	3.5	4.96	13.3
5886	0	0	1	3	15.8	4.06	10.4
5887	0	0	0	2	5.4	4.17	11.7
5888	0	0	1	2	12.1	4.8	12.4
5889	0	0	1	4	13.3	4.04	11.3
5890	0	0	0	4	13	4.82	12.9
5891	0	0	1	2	13.5	4.87	12
5892	0	0	0	0	18.2	3.62	11.1
5893	0	0	1	3	13.2	4.54	11.6
5894	0	0	0	3	6.4	4.88	12.9
5895	0	0	0	3	9.8	3.94	10.5
5896	0	0	1	4	7.6	4.88	12.3
5897	0	0	1	2	10.5	4.68	13.3
5898	0	0	0	2	8	4.19	11
5899	0	0	1	1	8.7	4.79	12.6
5900	0	0	0	2	15.9	5.03	13.9

5901	0	0	0	1	8.3	4.79	12.6
5902	0	0	1	4	18.5	4.13	11.8
5903	0	0	1	2	12.3	5.35	10.8
5904	0	0	1	3	17.7	4.19	11.9
5905	0	0	1	2	14.6	4.35	11.4
5906	0	0	0	3	10.4	4.73	12.3
5907	0	0	1	4	3.4	3.97	11.3
5908	0	0	1	2	7.4	4.31	11.6
5909	0	0	0	4	21.8	4.23	11.9
5910	0	0	1	2	7.7	4.59	11.8
5911	0	0	0	1	10.5	4.69	13
5912	0	0	1	1	9.2	4.54	11.3
5913	0	0	0	3	8.1	4.58	12.5
5914	0	0	0	2	8.5	4.4	12
5915	0	0	1	4	4.8	4.5	12.1
5916	0	0	0	4	7.7	5.09	13.5
5917	0	0	1	2	8.8	4.6	12.2
5918	0	0	1	1	10.8	4.57	12
5919	0	0	1	2	20.8	4.57	11.4
5920	0	0	0	2	14.6	4.78	12.7
5921	0	0	1	3	8.5	4.87	13
5922	0	0	1	3	11.5	4.58	12.5
5923	0	0	0	4	15.9	3.88	10.8
5924	0	0	1	4	5.4	4.6	12.4
5925	0	0	1	2	3.7	2.94	8.5
5926	0	0	0	2	8	4.64	12.2
5927	0	0	1	2	17.1	4.2	11.5
5928	0	0	1	3	29	5.12	13.2
5929	0	0	0	2	16.9	4.79	13
5930	0	0	1	3	7.7	5.16	13.5
5931	0	0	0	4	11.3	4.03	10.9
5932	0	0	0	3	5.8	4.25	12.4
5933	0	0	0	3	4.9	4.26	12.1
5934	0	0	1	3	4.5	4.48	12.3
5935	0	0	1	4	9.8	4.72	12.7
5936	0	0	1	4	8.7	5.05	13.5
5937	0	0	0	2	4.5	4.47	12.3
5938	0	0	0	2	14.8	4.44	11.1
5939	0	0	0	3	5.2	4.41	11.9
5940	0	0	0	4	6.1	4.53	12.3
5941	0	0	0	1	10.9	4.02	11.2
5942	0	0	0	3	13.2	4.86	13
5943	0	0	1	2	9.6	4.57	11.8
5944	0	0	1	1	6.4	4.08	10.8
5945	0	0	1	0	12	4.37	11.6
5946	0	0	0	1	6.2	4.46	11.7

5947	0	0	0	2	9.9	4.66	12.7
5948	0	0	1	2	14.1	4.68	11.9
5949	0	0	0	1	6.4	4.64	12.5
5950	0	0	1	2	8.4	4.87	13.3
5951	0	0	1	3	5.2	4.94	13.2
5952	0	0	0	1	8.2	4.77	12.9
5953	0	0	0	2	5.7	4.72	10.9
5954	0	0	0	1	21.3	4.6	12.8
5955	0	0	0	2	6	4.42	12.4
5956	0	0	1	1	4.3	4.75	12.7
5957	0	0	1	2	6.5	4.35	12.3
5958	0	0	1	2	7.7	4.75	12.4
5959	0	0	1	2	13.3	4.43	11.9
5960	0	0	1	1	15.7	4.64	12.9
5961	0	0	1	3	8	4.61	12.3
5962	0	0	0	4	4.1	4.93	13.6
5963	0	0	1	0	16.9	4.38	11.2
5964	0	0	0	1	8.7	4.89	13
5965	0	0	1	1	6.4	4.75	12.1
5966	0	0	0	4	7.1	4.58	12.2
5967	0	0	0	4	23.9	4.57	11.5
5968	0	0	0	1	14	4.54	11.7
5969	0	0	0	3	6	4.48	12.1
5970	0	0	0	2	9.9	4.43	12.3
5971	0	0	0	1	2.5	4.41	11.5
5972	0	0	0	1	12.6	4.46	11.2
5973	0	0	1	0	11.2	3.77	10.7
5974	0	0	0	1	6.3	3.88	10.1
5975	0	0	1	1	17.2	4.89	12.1
5976	0	0	1	1	16.5	4.55	12
5977	0	0	0	2	5.4	4.52	12.5
5978	0	0	0	2	19.7	3.96	10.6
5979	0	0	0	2	6	3.97	9.7
5980	0	0	1	2	14.2	4.18	11.2
5981	0	0	1	0	11.6	3.69	10.7
5982	0	0	0	1	27.1	4.69	11.2
5983	0	0	0	2	13.6	4.7	12.6
5984	0	0	1	0	27	3.78	10.5
5985	0	0	0	2	9	4.24	11
5986	0	0	0	1	27.4	4.73	13.5
5987	0	0	1	1	7.1	4.49	12.6
5988	0	0	0	4	13.7	4.63	12.2
5989	0	0	1	0	13	4	11
5990	0	0	1	2	11.3	4.91	13.5
5991	0	0	1	1	26.6	4.07	11.6
5992	0	0	1	1	14.6	4.4	11.7

5993	0	0	1	4	19.3	4.76	12.8
5994	0	0	1	0	18	2.98	9.2
5995	0	0	0	2	14.7	4.96	13.3
5996	0	0	0	2	5.5	4.5	12.9
5997	0	0	1	0	12.2	4	10
5998	0	0	0	1	6.8	4.68	12.9
5999	0	0	1	0	15.3	4.32	11.7
6000	0	0	0	1	8.7	4.7	11.5
6001	0	0	0	2	8.4	4.76	12.5
6002	0	0	1	1	14.9	4.53	12
6003	0	0	1	1	5	4.66	12.2
6004	0	0	0	1	7.4	4.4	12.4
6005	0	0	1	3	20	4.21	11.2
6006	0	0	0	0	12.2	3.83	11.3
6007	0	0	1	2	7.5	4.76	11.8
6008	0	0	1	1	8	4.4	12.4
6009	0	0	1	1	5.3	4.54	12
6010	0	0	1	0	10.3	3.8	11.7
6011	0	0	1	2	14.2	4.45	12.1
6012	0	0	1	2	12.7	4.31	11.6
6013	0	0	1	1	4.9	4.14	10.7
6014	0	0	1	2	28.9	4.32	12
6015	0	0	1	2	18	4.72	13.2
6016	0	0	0	1	8	4.47	11.9
6017	0	0	1	2	13.8	4.21	11.3
6018	0	0	1	2	7.3	5.31	13.1
6019	0	0	0	1	6	4.59	11.7
6020	0	0	1	1	20.2	4.27	11.4
6021	0	0	1	1	25.5	4.12	11.2
6022	0	0	0	3	2.6	4.21	11.5
6023	0	0	0	2	7	4.41	11.4
6024	0	0	0	1	26.9	4.28	11.4
6025	0	0	1	3	8.4	5.18	13.5
6026	0	0	0	0	6.3	3.36	10.7
6027	0	0	0	1	12.2	4.34	11.6
6028	0	0	0	0	28.6	3.46	9.9
6029	0	0	0	1	13.9	3.78	9.6
6030	0	0	0	4	19.3	4.44	13.1
6031	0	0	0	0	23.1	3.85	10.6
6032	0	0	1	1	10.8	4.39	12
6033	0	0	1	4	10.3	4.55	11.9
6034	0	0	1	1	27.7	4.75	11.3
6035	0	0	0	0	8.4	3.7	10.2
6036	0	0	0	4	19.9	4.1	11.9
6037	0	0	0	2	7.1	4.72	13.2
6038	0	0	0	1	4.7	3.81	10.4

6039	0	0	0	2	9.3	4.63	12
6040	0	0	1	3	13.3	4.55	12.5
6041	0	0	1	3	4.1	4.65	11.9
6042	0	0	1	1	3.2	4.4	10.7
6043	0	0	1	1	9.3	4.98	13.5
6044	0	0	0	2	5.1	4.28	11.9
6045	0	0	0	1	9.8	4.41	11.6
6046	0	0	0	1	19.4	4.67	10.8
6047	0	0	1	2	5	4.45	12
6048	0	0	1	1	32	3.47	9.1
6049	0	0	0	0	13.8	3.71	11.5
6050	0	0	1	2	8.1	4.75	12.5
6051	0	0	1	2	5.6	4.27	12.1
6052	0	0	1	3	11.2	5.06	13
6053	0	0	0	1	4.7	4.04	11.1
6054	0	0	1	0	22.6	4.22	11.5
6055	0	0	1	0	27	4.59	12.1
6056	0	0	1	1	6	5.16	13.2
6057	0	0	0	3	0.8	3.18	8.2
6058	0	0	0	4	10.9	4.7	13.4
6059	0	0	1	1	16.9	4.58	11.8
6060	0	0	1	4	9.4	5.24	14.1
6061	0	0	0	0	9.6	3.68	11.5
6062	0	0	0	4	11.8	4.46	12.7
6063	0	0	1	1	10.1	4.35	11.2
6064	0	0	1	1	17.5	5.04	12.7
6065	0	0	1	0	13.3	4.26	10.9
6066	0	0	1	2	10.9	4.34	11.9
6067	0	0	0	1	9.3	4.3	11.9
6068	0	0	0	4	15	4.38	11.8
6069	0	0	1	4	8.1	4.52	12.4
6070	0	0	1	0	21.3	3.71	10.3
6071	0	0	0	1	13.4	3.94	10.3
6072	0	0	1	1	17	4.47	11
6073	0	0	1	1	15	4.28	11.7
6074	0	0	1	1	8.2	4.32	10.6
6075	0	0	0	3	13.6	4.74	13.2
6076	0	0	1	2	7	4.44	11.8
6077	0	0	0	1	9	4	13.4
6078	0	0	1	1	8	4.04	10.1
6079	0	0	1	1	12.5	3.76	10.4
6080	0	0	1	1	16.7	4.43	10.7
6081	0	0	0	1	7	4.81	12
6082	0	0	0	4	7.8	4.46	12.3
6083	0	0	1	4	6.9	4.78	12.8
6084	0	0	1	1	9.2	4.63	12

6085	0	0	1	1	3.9	3.74	10.2
6086	0	0	1	1	6	4.63	11.9
6087	0	0	0	2	3.6	4.29	11.8
6088	0	0	0	1	4.4	4.07	10.9
6089	0	0	1	1	14.8	3.51	10
6090	0	0	0	1	13.7	4.06	10.2
6091	0	0	1	4	6.8	4.42	12.1
6092	0	0	0	1	11.4	4.6	12.2
6093	0	0	0	1	6.2	4.31	12.4
6094	0	0	1	2	9.1	4.58	11.7
6095	0	0	1	1	13.5	4.33	11.6
6096	0	0	0	1	5.1	4.61	11.9
6097	0	0	0	1	15.9	4.26	10.8
6098	0	0	1	1	5.4	2.77	8.8
6099	0	0	1	1	3.5	4.18	11.1
6100	0	0	0	1	41.5	4.74	12.3
6101	0	0	0	1	5.9	4.43	12.3
6102	0	0	1	3	8.2	5.39	13.3
6103	0	0	1	2	5.4	4.53	10.9
6104	0	0	0	4	17.3	4.26	11
6105	0	0	1	1	6.6	4.17	10.3
6106	0	0	0	1	11.7	4.08	11
6107	0	0	1	1	21.1	4.83	12
6108	0	0	1	0	10.6	4.12	11.4
6109	0	0	0	1	17.3	4.45	11.8
6110	0	0	1	1	5	4.86	12.8
6111	0	0	1	1	12.2	4.3	11.1
6112	0	0	1	1	4.2	3.56	10.6
6113	0	0	1	1	9.2	4.39	12
6114	0	0	0	1	7.5	4.51	12.6
6115	0	0	0	1	19.7	5.21	12.7
6116	0	0	0	1	2.7	4.36	11.6
6117	0	0	1	1	6	4.25	11.1
6118	0	0	0	2	11.4	4.75	12.8
6119	0	0	1	3	5.7	4.85	13.3
6120	0	0	0	2	7.9	3.99	12.3
6121	0	0	1	2	20.3	4.77	12.3
6122	0	0	0	0	12.2	4.35	15.6
6123	0	0	1	1	5	4.66	11.5
6124	0	0	1	1	4.7	4.33	9.7
6125	0	0	1	1	5.9	4.51	12.3
6126	0	0	1	3	18.2	4.6	12.2
6127	0	0	0	4	8.7	4.37	11.5
6128	0	0	0	2	3	4.54	12.8
6129	0	0	0	2	5.3	5.11	11.9
6130	0	0	1	0	19.8	5	10.6

6131	0	0	0	2	6.6	4.74	10.4
6132	0	0	1	1	10	4.02	10.1
6133	0	0	1	4	6.1	5.07	14.2
6134	0	0	0	3	31.7	3.63	9.9
6135	0	0	1	1	8.3	4.72	12.2
6136	0	0	1	1	18.2	4.43	11.7
6137	0	0	1	3	7.3	4.07	11.7
6138	0	0	0	1	6.2	4.2	11.4
6139	0	0	1	1	6	3.91	10.7
6140	0	0	0	4	5.4	4.72	12
6141	0	0	1	1	8.5	4.63	12
6142	0	0	0	1	21.4	5.13	13
6143	0	0	0	2	9.8	4.82	12
6144	0	0	1	4	12.6	4.15	11.4
6145	0	0	0	1	2.9	4.18	10.3
6146	0	0	0	4	12.7	4.67	13.1
6147	0	0	1	1	17.5	3.78	9.8
6148	0	0	1	2	11.9	4.53	11.1
6149	0	0	1	1	5.3	4.8	11.7
6150	0	0	1	1	6.3	4.34	11.9
6151	0	0	0	2	9.9	4.39	12.3
6152	0	0	0	2	7.7	4.47	12.1
6153	0	0	1	1	9.3	5.09	12.8
6154	0	0	1	2	7.6	4.51	12.5
6155	0	0	0	2	5.6	4.91	13.2
6156	0	0	0	0	12.1	4.98	12.5
6157	0	0	1	1	3.2	4.47	11.7
6158	0	0	1	1	13.6	4.83	10.1
6159	0	0	1	0	7.8	4.55	12.3
6160	0	0	1	1	3.5	4.33	11.6
6161	0	0	0	2	4.2	4.09	11.5
6162	0	0	1	0	6.9	4.6	12.1
6163	0	0	0	4	22.6	4.64	12.8
6164	0	0	1	1	5.4	5.46	12.8
6165	0	0	1	2	22.2	4.51	11.4
6166	0	0	1	1	7.8	4.29	11.2
6167	0	0	1	2	6.3	5.09	12.6
6168	0	0	0	2	8.4	5.14	13.6
6169	0	0	1	1	10.5	4.06	10.6
6170	0	0	0	0	7.6	2.25	7.1
6171	0	0	1	2	4.6	4.16	11.2
6172	0	0	0	1	8	3.45	11.2
6173	0	0	0	4	8.9	4.37	12.2
6174	0	0	0	4	6	4.1	11.6
6175	0	0	1	2	8	3.94	11.4
6176	0	0	0	2	11	4.81	12.1

6177	0	0	1	2	4	4.43	11.5
6178	0	0	1	0	6.7	3.78	10.3
6179	0	0	1	1	17.9	4.04	11.7
6180	0	0	1	2	8.1	4.58	12.5
6181	0	0	1	1	12.6	4.75	11.9
6182	0	0	0	1	6	4.69	11.5
6183	0	0	1	1	12.4	5.05	13.3
6184	0	0	1	1	9.6	4.78	13.2
6185	0	0	1	1	16.7	4.69	11.9
6186	0	0	0	2	7.5	4.59	12.6
6187	0	0	1	2	17.1	4.17	10.7
6188	0	0	1	1	7.9	4.23	10
6189	0	0	1	3	7.1	5.46	11.9
6190	0	0	1	2	9.8	4.7	12.1
6191	0	0	0	1	12.8	4.05	10.7
6192	0	0	0	1	14.8	3.92	10.1
6193	0	0	0	3	5.4	5.14	11.6
6194	0	0	0	2	6.6	5.13	12.9
6195	0	0	1	2	15.7	4.78	11.9
6196	0	0	0	4	11.3	4.38	11.9
6197	0	0	0	1	13.3	3.8	10.4
6198	0	0	0	1	6	4.32	12
6199	0	0	0	1	9.1	4.68	12.5
6200	0	0	0	2	8	4.59	11.9
6201	0	0	0	2	5.3	4.54	12
6202	0	0	1	2	8.9	4.65	12
6203	0	0	0	4	5.2	4.06	10.4
6204	0	0	1	0	6.1	4.22	11.3
6205	0	0	0	4	10.1	4.45	11.9
6206	0	0	0	1	12.9	4.04	10.8
6207	0	0	0	1	11.2	4.6	12.4
6208	0	0	1	4	9.3	4.42	12.5
6209	0	0	1	1	12.9	3.99	11
6210	0	0	1	1	9.6	4.86	12.6
6211	0	0	0	1	10.8	4.67	13
6212	0	0	0	0	3.4	4.38	11.6
6213	0	0	0	1	4.5	4.51	12.4
6214	0	0	1	0	10.3	4.05	10.5
6215	0	0	0	0	11.2	4.56	12.3
6216	0	0	1	0	7.2	3.15	9.5
6217	0	0	0	1	19.7	4.32	11.2
6218	0	0	0	1	12.5	3.94	10.2
6219	0	0	1	0	13.7	3.89	11.5
6220	0	0	1	1	5.7	4.54	11.6
6221	0	0	1	1	3.3	4.74	11.3
6222	0	0	0	3	3.9	4.45	12.8

6223	0	0	1	0	4.2	4.29	10
6224	0	0	1	2	10.8	4.36	11.7
6225	0	0	0	1	17.5	4.83	13.1
6226	0	0	1	0	10.4	4.86	13.2
6227	0	0	0	2	11	4.7	12.6
6228	0	0	0	3	6.8	4.08	11.8
6229	0	0	1	0	9.9	4.47	11.6
6230	0	0	1	0	10.3	4.03	9.8
6231	0	0	1	4	17.5	5.05	13.6
6232	0	0	0	1	7.9	4.07	11.8
6233	0	0	1	1	8.4	4.04	10.7
6234	0	0	1	0	7	3.59	10.1
6235	0	0	0	1	14.9	5.18	13.8
6236	0	0	0	4	5.7	4.84	13.4
6237	0	0	0	1	8.8	4.14	11
6238	0	0	1	1	5	4.78	13
6239	0	0	0	1	8	4.48	12
6240	0	0	1	2	4.7	4.81	13.1
6241	0	0	0	3	9.5	4.01	11.3
6242	0	0	1	1	19.1	5.04	13.3
6243	0	0	1	1	5.2	4.45	12.4
6244	0	0	1	1	5.2	4.33	12.9
6245	0	0	1	1	3.2	3.09	9.4
6246	0	0	0	1	14.1	4.62	12.7
6247	0	0	1	1	9	4.31	11.9
6248	0	0	1	3	11.6	4.07	11.5
6249	0	0	0	1	16.5	4.58	13.1
6250	0	0	1	3	5.1	4.69	12.5
6251	0	0	1	3	5.5	4.53	11.9
6252	0	0	1	1	3.5	4.98	12.7
6253	0	0	1	1	16	4.8	11.9
6254	0	0	0	1	5.9	4.52	12.2
6255	0	0	0	1	16.1	4.69	12.9
6256	0	0	0	1	6.2	4.52	12.4
6257	0	0	0	0	6.9	4.18	11.2
6258	0	0	1	1	5.8	4.53	12
6259	0	0	0	1	5.7	4.59	12.4
6260	0	0	0	1	10.6	4.64	12.4
6261	0	0	0	1	10.9	4.76	12.9
6262	0	0	0	3	18.1	5.01	13.5
6263	0	0	1	3	12.4	4.38	11.7
6264	0	0	1	1	7.2	4.29	11
6265	0	0	1	3	10	4.48	12.1
6266	0	0	1	1	12.6	4.8	13
6267	0	0	0	1	11.6	4.36	11.8
6268	0	0	1	1	6.5	4.32	11.4

6269	0	0	1	1	8	4.96	11.6
6270	0	0	1	4	9.2	4.37	12.1
6271	0	0	0	2	5	4.58	12.3
6272	0	0	0	1	2.8	2.6	8.4
6273	0	0	1	2	17	4.57	11.2
6274	0	0	1	1	2.7	4.41	11.7
6275	0	0	0	1	6.9	4.17	10.9
6276	0	0	1	1	13.1	4.66	13.4
6277	0	0	0	1	3.4	4.52	12
6278	0	0	1	1	13.5	4.75	12.6
6279	0	0	1	1	13.1	3.94	10.7
6280	0	0	1	1	8	4.96	12.5
6281	0	0	0	1	6.5	3.95	10.8
6282	0	0	0	3	9.6	4.81	13.1
6283	0	0	0	0	5.1	3.64	10.1
6284	0	0	0	1	9.9	4.43	12.7
6285	0	0	0	1	6.6	4.75	12.8
6286	0	0	1	0	11.7	4.51	11.7
6287	0	0	0	3	29.3	4.63	11.7
6288	0	0	1	1	10.8	4.65	11.1
6289	0	0	1	2	12.1	5	13.5
6290	0	0	0	1	9.8	5.39	14.8
6291	0	0	1	1	5.4	4.69	12.4
6292	0	0	1	1	4.7	4.46	11.5
6293	0	0	1	1	5.8	4.95	13
6294	0	0	0	2	8.5	4.24	11.9
6295	0	0	0	1	7	4.77	12.8
6296	0	0	1	0	16.2	4.62	11.9
6297	0	0	1	1	2.9	4.46	11.5
6298	0	0	0	0	5.6	3.33	10.4
6299	0	0	0	2	7	4.75	11.6
6300	0	0	0	1	5.2	4.48	11.6
6301	0	0	1	1	4	5.11	13
6302	0	0	1	1	12.4	4.33	11.3
6303	0	0	1	0	8.4	4.01	10.4
6304	0	0	1	1	9.6	4.29	11.8
6305	0	0	0	1	23.9	4.42	11.1
6306	0	0	1	0	2.7	4.12	11.1
6307	0	0	1	1	10.3	4.32	12.4
6308	0	0	0	3	4.6	4.21	11.9
6309	0	0	1	2	14	4.6	13.2
6310	0	0	0	0	15	4.28	15.5
6311	0	0	1	1	6	4.4	10.4
6312	0	0	1	2	6.7	4.97	13.3
6313	0	0	1	2	7.9	4.86	12.9
6314	0	0	1	1	9.8	5	11.2

6315	0		0	1	4	6.3	4.8	13.6
6316	0		0	1	1	22.1	4.36	11.4
6317	0		0	1	4	15.8	4.25	11.5
6318	0		0	1	2	7.8	5.05	13.3
6319	0		0	1	1	24.5	3.91	10.2
6320	0		0	0	2	10.1	4.29	11.1
6321	0		0	0	3	14.8	4.2	11.2
6322	0		0	0	4	7.3	4.34	11.8
6323	0		0	1	0	16.8	4.27	10.2
6324	0	1	1	0	1	11.5	4.49	12.1
6325	0	1	1	0	1	18.9	3.92	10.4
6326	0	1	1	1	3	14.7	4.43	12.1
6327	0	0	1	1	1	12.3	3.87	10.1
6328	0	0	1	1	1	20.1	3.45	9
6329	0	1	1	0	1	7.5	4.32	11.5
6330	0	1	1	1	3	18.8	4.17	11
6331	0	0	1	1	2	13.7	4.8	13.5
6332	0	1	1	1	7	14.5	4.9	14.1
6333	0	0	1	0	0	10.9	3.39	8.8
6334	0	1	1	0	1	12.4	4.29	11.5
6335	0	1	1	1	3	14.6	4.6	12.3
6336	0	1	1	0	2	8.2	4.35	12.5
6337	0	0	1	1	2	9.4	3.74	10.5
6338	0	1	1	0	7	13.4	4.33	12.4
6339	0	1	1	1	1	14.9	4.59	11.6
6340	0	1	1	1	1	23	3.88	10.3
6341	0	1	1	1	6	16.1	4.27	12
6342	0	1	1	0	8	3.4	4.11	11.5
6343	0	1	1	1	2	11	4.6	12.2
6344	0	1	1	0	2	10.8	4.26	12.1
6345	0	1	1	0	1	11.2	3.55	9.7
6346	0	1	1	0	2	13.7	4.53	11.5
6347	0	1	1	1	5	5	3.41	8.7
6348	0	1	1	0	1	18.6	4.64	11.3
6349	0	1	1	1	2	26.6	4.58	12.5
6350	0	1	1	1	1	4.4	4.51	12.3
6351	0	0	1	1	3	10.2	4.22	11.6
6352	0	1	1	0	1	15.5	4.58	12.7
6353	0	1	1	1	2	19.9	4.65	12.8
6354	0	0	1	1	1	14.9	3.46	8.3
6355	0	0	1	1	3	15.2	3.55	10.5
6356	0	1	1	0	1	6.5	4.34	11.5
6357	0	1	1	1	1	12.1	3.9	9.5
6358	0	1	1	1	1	16.5	5.03	9.9
6359	0	1	1	1	1	8.2	4.27	11.4
6360	0	1	1	1	1	19.6	4.47	12.4

6361	0	1	1	1	2	12.5	4.74	12.2
6362	0	1	1	1	2	17.7	4.62	12.3
6363	0	1	1	0	4	15.8	3.98	10.6
6364	0	1	1	1	1	11.6	3.8	11
6365	0	1	1	0	1	11.2	4.79	12.2
6366	0	0	1	1	5	11.7	4.4	11.3
6367	0	1	1	0	1	12.9	5.07	13.4
6368	0	1	1	1	3	10.3	4.84	11.1
6369	0	1	1	1	2	9	3.85	9.9
6370	0	1	1	1	4	13.2	4.51	11.5
6371	0	1	1	1	1	14	4.42	12.5
6372	0	0	1	0	1	14.8	4.34	11.5
6373	0	1	1	1	3	11.3	3.9	10.2
6374	0	1	1	1	1	14.1	3.98	10.3
6375	0	1	1	1	1	16.6	4.28	11.7
6376	0	1	1	1	1	12.8	4.69	12
6377	0	1	1	1	1	11.8	4.43	11.7
6378	0	1	1	1	4	11.2	4.08	11
6379	0	1	1	0	1	4.8	3.52	9.4
6380	0	0	1	1	0	17	4.07	10.2
6381	0	1	1	0	0	16.4	4.25	11.2
6382	0	1	1	1	3	12.7	4.3	11.7
6383	0	1	1	1	0	9.2	3.07	8.1
6384	0	1	1	0	2	8.2	3.69	9.6
6385	0	0	1	1	3	22.7	4.56	10.8
6386	0	1	1	1	2	12.9	3.55	9.5
6387	0	1	1	0	0	30.5	3.99	10.5
6388	0	1	1	1	3	19.8	3.77	10.5
6389	0	1	1	1	1	13.7	4.08	10.7
6390	0	1	1	0	6	13.7	3.81	10.3
6391	0	1	1	0	1	12.6	4.48	8.4
6392	0	1	1	0	0	27.4	4.36	10.9
6393	0	0	1	1	0	9.4	3.18	8.2
6394	0	1	1	1	2	14	3.62	9.7
6395	0	1	1	1	1	21	4.63	11.3
6396	0	0	1	1	3	12.5	4.58	11
6397	0	0	1	1	4	4.7	4.13	10.8
6398	0	1	1	0	2	16.1	5.22	14.2
6399	0	1	1	0	1	14.4	4.33	10.8
6400	0	0	1	0	3	16.3	4.04	10.8
6401	0	1	1	1	1	13.8	4.04	10.3
6402	0	1	1	1	1	10.2	4.11	11.7
6403	0	1	1	1	0	14.4	3.78	10.7
6404	0	1	1	1	2	17.2	4.3	11.4
6405	0	1	1	1	2	12.9	4.51	12.7
6406	0	1	1	1	1	11.1	2.94	8.2

6407	0	0	1	1	1	31.1	4.01	10.2
6408	0	1	1	0	1	13.7	4.44	11
6409	0	1	1	0	3	16.2	4.58	9.1
6410	0	0	1	0	5	4.4	3.69	10.2
6411	0	1	1	1	2	13.5	3.92	11.3
6412	0	1	1	1	0	15.1	3.34	8.9
6413	0	0	1	0	2	9.7	3.66	9.6
6414	0	1	1	0	3	9.4	4.19	12.4
6415	0	1	1	1	1	11.8	3.87	10.3
6417	0	1	1	0	1	10.5	3.61	10
6418	0	1	1	0	1	13.9	4.1	9.9
6419	0	1	1	1	2	15.5	4.16	11.3
6420	0	1	1	0	2	22.9	4.11	10.4
6421	0	1	1	1	2	16.1	4.19	11.5
6422	0	0	1	1	1	21.6	4.15	10.1
6424	0	1	1	0	3	4.9	5.61	10.4
6425	0	1	1	1	0	13	3.49	8.8
6426	0	1	1	0	2	5.2	4.57	12.3
6427	0	1	1	0	0	18.3	4.01	10.4
6428	0	1	1	0	4	19.5	3.69	10.3
6429	0	1	1	1	1	17.4	3.9	10.3
6430	0	1	1	1	1	14.6	3.96	9.5
6431	0	0	1	0	1	8.5	3.47	9.9
6432	0	1	1	0	7	12.9	4.2	11.1
6433	0	1	1	1	0	7.9	4.4	10.8
6434	0	1	1	1	2	16.8	4.26	9.9
6435	0	1	1	1	3	10.5	4.46	11.5
6436	0	0	1	1	3	12.3	4.03	11.3
6437	0	1	1	0	1	14.7	4.62	11.8
6438	0	0	1	0	2	10.2	4.07	10.7
6439	0	1	1	0	1	13.5	4.1	10.8
6440	0	1	1	0	3	15.2	4.52	12.1
6441	0	1	1	0	1	7.2	4.82	12
6442	0	1	1	0	2	13.6	4.41	11.1
6443	0	1	1	0	3	29.4	4.49	12.1
6444	0	1	1	1	3	7.1	4.37	12
6445	0	1	1	1	1	12.7	4.53	8.4
6446	0	0	1	0	0	17.5	2.75	7.3
6447	0	1	1	0	1	22.9	4.07	11.1
6448	0	1	1	0	0	9.9	3.96	11
6449	0	1	1	1	0	11.4	3.99	10.4
6450	0	1	1	1	1	19.7	4.11	11.2
6451	0	1	1	1	1	10.2	4.52	12
6452	0	1	1	0	1	12.5	4.21	10.8
6453	0	1	1	1	0	21.1	3.8	9.4
6454	0	1	1	1	0	9.1	4.89	12.5

6455	0	1	1	0	4	7.9	3.9	10.9
6456	0	1	1	0	4	12.4	4.38	11.7
6457	0	1	1	1	0	9.4	4.25	10.9
6458	0	1	1	0	2	8.6	4.42	12
6459	0	0	1	0	0	13.7	3.86	10.1
6460	0	1	1	0	2	19	4.2	1.4
6461	0	1	1	1	1	18.8	3.74	10.1
6462	0	1	1	1	2	9.5	3.86	10.6
6463	0	1	1	0	1	15.4	4.23	10.9
6464	0	1	1	1	2	6	4.97	12.7
6465	0	1	1	0	6	14.9	4.76	13.4
6466	0	0	1	0	1	10.3	4.35	11.1
6467	0	1	1	0	1	12.2	4.28	11.4
6468	0	0	1	0	2	9.7	4.58	10.9
6469	0	1	1	1	1	8.5	5.26	11.7
6470	0	1	1	0	1	15.2	4.69	11.8
6471	0	1	1	0	1	3.7	4.07	11.4
6472	0	1	1	1	1	15.3	4.57	11.6
6473	0	1	1	0	1	11.2	5.03	12.6
6474	0	0	1	0	1	11.8	3.78	9.7
6475	0	1	1	0	1	8.8	5.47	10.5
6476	0	1	1	1	1	16.2	4.42	12.1
6477	0	1	1	1	1	13.4	4.7	11.1
6478	0	1	1	1	1	6.9	4.28	11.7
6479	0	1	1	1	3	11.1	4.08	11
6480	0	1	1	0	0	13.1	5.42	9.9
6481	0	1	1	1	3	22.6	4.36	11.4
6482	0	1	1	0	3	15.2	4.38	12.1
6483	0	1	1	0	1	13.4	4.21	10.2
6484	0	1	1	0	3	13.6	4.53	11.8
6485	0	1	1	1	0	8.5	3.59	9.4
6486	0	1	1	0	0	16.6	3.39	8.9
6487	0	1	1	1	0	7.4	3.24	9.3
6488	0	1	1	0	1	20.4	5.42	10
6489	0	1	1	1	1	13.4	3.89	10.9
6490	0	1	1	0	2	18.7	4.79	13
6491	0	1	1	0	2	11.2	3.56	9.9
6492	0	1	1	1	1	17.3	4.29	11.5
6493	0	1	1	1	1	16.5	4.01	10.1
6494	0	1	1	0	1	11.6	4.09	10.5
6495	0	1	1	1	3	13.5	4.44	11.6
6496	0	1	1	1	1	16.6	4.33	11.5
6497	0	1	1	0	3	17.3	4.51	12
6498	0	0	1	1	0	13.9	4.17	10.5
6499	0	1	1	1	1	32.9	4.5	11.6
6500	0	1	1	0	5	15	4.78	12.7

6501	0	1	1	0	1	7.3	4.87	12
6502	0	1	1	0	0	11.5	3.96	10.7
6503	0	1	1	0	3	14.4	4.06	10.4
6504	0	0	1	1	1	21.1	4.38	11.6
6505	0	1	1	0	2	21.2	4.15	11.1
6506	0	1	1	0	1	13	3.75	9.8
6507	0	1	1	1	2	15.2	4.1	10.9
6508	0	1	1	1	4	6.2	4.24	11.9
6509	0	1	1	0	3	22.3	3.58	9.1
6510	0	1	1	1	1	25.2	3.69	9.8
6511	0	1	1	1	2	21.7	4.67	11.9
6512	0	1	1	1	5	10.2	4.15	11.4
6513	0	1	1	0	2	18.4	4.13	11.1
6514	0	0	1	0	0	19.4	3.91	11.5
6515	0	1	1	0	1	19	3.56	9.3
6516	0	0	1	0	1	15.4	3.5	9.5
6517	0	0	1	0	1	12.5	3.59	9.8
6518	0	1	1	1	3	14.1	3.99	10.9
6519	0	1	1	1	0	11.4	3.66	10.1
6520	0	1	1	1	3	12.1	4.32	11.1
6521	0	1	1	1	2	11.3	4.51	12.4
6522	0	1	1	0	0	6.6	3.81	10.5
6523	0	1	1	1	1	8.7	4.59	11.9
6524	0	1	1	1	2	7.5	4.88	11.7
6525	0	1	1	1	4	5.8	4.23	12.1
6526	0	1	1	1	3	16.4	4.47	12.3
6527	0	1	1	1	1	12.2	4.33	11.4
6528	0	1	1	1	2	9.8	4.22	11.9
6529	0	1	1	1	1	14.2	4.71	12.2
6531	0	1	1	1	1	12.9	4.27	11.1
6532	0	1	1	1	5	19.5	3.99	11.1
6533	0	1	1	1	1	9.4	4.87	12.5
6534	0	1	1	1	4	9.4	4.04	11.7
6535	0	1	1	1	0	17.4	4.19	11.3
6536	0	1	1	1	0	18.9	3.82	9.8
6537	0	1	1	1	1	14.3	3.93	9.5
6538	0	1	1	1	0	10.1	3.88	10.8
6539	0	1	1	1	2	16.6	4.09	11.8
6540	0	1	1	1	1	15.5	4.6	11.2
6541	0	1	1	1	1	18	4.68	11.2
6542	0	1	1	0	1	14.3	3.75	10.1
6543	0	1	1	0	0	13	4.44	12.7
6544	0	1	1	1	1	11	4.52	12
6545	0	1	1	0	1	11.4	4.2	11.5
6546	0	1	1	1	4	8.1	4.41	11.6
6547	0	1	1	0	2	14.9	4.26	11.4

6548	0	0	1	1	0	17.4	4.26	10.8
6549	0	1	1	1	1	18.2	5.05	13.5
6550	0	0	1	0	1	13.6	4.24	11.3
6551	0	1	1	1	0	19.8	4.29	10.4
6552	0	1	1	1	2	6.7	4.46	11.8
6553	0	1	1	1	2	9.4	3.48	9.2
6554	0	1	1	0	3	11.1	4.11	11.3
6555	0	1	1	1	1	13.1	4.13	11.3
6556	0	1	1	0	4	22	5.23	9.9
6557	0	1	1	1	2	9.8	4.25	11.8
6558	0	1	1	0	2	21.5	4.3	12.3
6559	0	1	1	0	3	11	3.99	10.9
6560	0	1	1	0	1	18.2	4.01	11.4
6561	0	1	1	1	4	7.9	4.36	10.9
6562	0	1	1	0	2	14	3.54	10
6563	0	1	1	1	1	15.7	3.91	10.3
6564	0	1	1	1	1	14.6	3.68	7.9
6565	0	1	1	0	2	15.5	4.1	11.5
6566	0	1	1	0	9	21.1	4.26	12.1
6567	0	1	1	1	1	9.2	4.68	11.8
6568	0	0	1	1	1	12.1	4.92	9.1
6569	0	1	1	0	1	14.3	4.66	11.9
6570	0	1	1	0	1	12.6	4.79	12.7
6571	0	1	1	1	2	9.6	4.1	11.1
6572	0	1	1	1	2	12.7	5.89	11.6
6573	0	1	1	0	3	21.3	4.08	10.7
6574	0	1	1	1	3	10.5	4.32	12.2
6575	0	1	1	1	2	11.1	4.51	11.3
6576	0	1	1	0	1	19	4.04	10.7
6577	0	1	1	1	2	16.6	4.13	10.8
6578	0	1	1	0	4	8.4	4	11.1
6579	0	1	1	1	0	12.5	5.7	10.9
6580	0	1	1	0	1	10.5	4.61	12.3
6581	0	1	1	1	2	11.4	4.89	11.9
6582	0	0	1	1	2	11.9	3.47	8.6
6583	0	0	1	1	1	15.6	4.4	10.1
6584	0	1	1	1	4	16.1	4.38	11.6
6585	0	0	1	1	1	22.1	4.18	10.7
6586	0	1	1	1	2	10.4	4.5	11.6
6587	0	0	1	1	2	12.8	4.03	11
6588	0	1	1	1	4	23.7	4.36	11.7
6589	0	1	1	1	1	12.1	4.55	11.7
6590	0	0	1	1	5	6.9	4.51	12
6591	0	1	1	1	1	13.1	3.91	10.1
6592	0	0	1	1	2	12.4	4.37	11.9
6593	0	1	1	1	0	12.1	4.68	10.2

6594	0	1	1	1	1	13	3.86	10.2
6595	0	0	1	1	1	9.3	4.73	10.6
6596	0	1	1	0	1	10.6	3.7	10.2
6597	0	1	1	1	1	13	4.22	11.3
6598	0	1	1	1	1	17.5	3.88	10.5
6599	0	1	1	1	1	14.3	4.46	11.6
6600	0	1	1	1	2	14.1	3.78	10.2
6601	0	1	1	1	1	11.2	4.42	10.4
6602	0	1	1	0	2	8.5	4.46	12.4
6603	0	1	1	1	1	9.7	4.17	10
6604	0	1	1	1	2	15.9	4.06	10.6
6605	0	1	1	1	1	12.9	4.12	10.8
6606	0	1	1	1	1	6.5	4.87	12.8
6607	0	0	1	1	1	12.5	4.06	10.5
6608	0	1	1	0	2	13.3	4.11	11.3
6609	0	1	1	1	1	9.4	3.72	9.6
6610	0	0	1	1	0	18.4	3.73	10.4
6611	0	0	1	1	9	9.4	3.8	9.8
6612	0	1	1	0	3	11.3	4.39	12.2
6613	0	1	1	1	1	5.9	4.71	12.5
6614	0	1	1	1	2	9.4	4.63	11.5
6615	0	1	1	1	2	18.4	3.77	10.2
6616	0	1	1	0	0	22.5	4.53	11.4
6617	0	1	1	1	1	10.2	4.11	10.6
6618	0	1	1	1	1	8	4.56	11.9
6619	0	1	1	1	3	16.1	4.07	11.2
6620	0	0	1	1	1	12.7	4.26	11.6
6621	0	0	1	1	1	21.1	3.66	10.2
6622	0	1	1	0	5	18.1	4.2	11.7
6623	0	1	1	1	6	13.5	4.7	12.7
6624	0	1	1	1	2	17.4	4.34	11.3
6625	0	0	1	1	1	12	4.76	11.7
6626	0	1	1	0	2	35	4.09	11.4
6627	0	1	1	0	1	12.1	3.93	9.9
6628	0	1	1	1	1	7.8	4.51	11.7
6629	0	0	1	0	2	17	3.96	11.1
6630	0	1	1	0	1	11	4.23	10.7
6631	0	1	1	1	1	6.8	4.46	11.7
6632	0	0	1	1	1	9.4	4.44	11.2
6633	0	1	1	0	1	12.1	4.21	10.6
6634	0	1	1	0	1	12.5	4.73	12.2
6635	0	0	1	1	1	15.8	4.46	11.9
6636	0	1	1	1	2	13.8	4.06	10.1
6637	0	1	1	1	1	9.3	3.62	9.8
6638	0	0	1	1	1	9.8	4.82	12.1
6639	0	0	1	1	1	17	4.48	10.1

6640	0	0	1	0	6	12.3	4.45	11.5
6641	0	1	1	0	4	10.5	4.25	11.6
6642	0	1	1	1	3	8.8	5.17	13.4
6643	0	1	1	1	1	11.2	5.08	13.5
6644	0	1	1	1	1	10.4	3.83	10.8
6645	0	1	1	1	1	12	4.97	12.8
6646	0	0	1	0	0	14.7	3.73	11.1
6647	0	1	1	0	2	10.2	4.47	12
6648	0	1	1	1	2	14.4	4.75	12
6649	0	1	1	1	1	10.9	3.97	9.8
6650	0	1	1	0	2	13.5	4.24	11.3
6651	0	0	1	0	1	8.4	4.14	10.3
6652	0	1	1	1	1	7.5	4.24	11.2
6653	0	1	1	1	0	16.9	4.51	11.6
6654	0	1	1	1	2	8.4	4.09	10.8
6655	0	1	1	0	4	12.2	3.76	10.3
6656	0	1	1	1	2	13.2	4.87	12.6
6657	0	0	1	1	1	21.4	4.14	10.9
6658	0	1	1	0	3	17.2	4.65	12.5
6659	0	0	1	0	1	7.5	4.75	11.8
6660	0	0	1	1	2	9.1	3.9	10.4
6661	0	1	1	0	3	13.1	4.44	12.9
6662	0	1	1	0	5	13.2	4.33	11.6
6663	0	1	1	0	3	15.5	3.65	9.4
6664	0	1	1	1	1	10.8	4.41	12.1
6665	0	0	1	1	1	7.6	4.36	11.1
6666	0	0	1	1	3	16.5	4.39	11.5
6667	0	1	1	1	1	16.9	4.08	10.8
6668	0	1	1	0	2	7.6	4.23	11.6
6669	0	1	1	0	1	12.4	5.19	12.9
6670	0	1	1	1	1	8.6	4.07	10.7
6671	0	1	1	1	3	14.6	3.89	10.2
6672	0	1	1	0	2	11.1	4.57	11
6673	0	1	1	0	1	23.3	4.13	11.5
6674	0	1	1	0	4	13.1	5.29	10.2
6675	0	1	1	1	2	11.7	4.59	11.1
6676	0	1	1	0	5	11	4.14	11.3
6677	0	0	1	0	4	14.7	4.31	11.5
6678	0	0	1	0	3	9.8	4.31	11.6
6679	0	0	1	1	3	10.3	4.38	11.8
6680	0	0	1	0	1	10	4.26	11.7
6681	0	0	1	1	2	16.9	4.65	12.9
6682	0	0	1	0	2	13.1	3.8	10.1
6683	0	0	1	1	2	13.2	4.85	128
6684	0	0	1	0	2	16	4.4	10.8
6685	0	0	1	1	2	4.8	3.91	10.7

6686	0	0	1	1	1	9.5	4.07	9.5
6687	0	0	1	0	3	14.6	4.03	10.3
6688	0	0	1	1	5	6	3.7	9.6
6689	0	0	1	0	2	14.8	4.95	13.4
6690	0	0	1	1	3	8	4.99	13.1
6691	0	0	1	1	2	12.4	3.83	10.1
6692	0	0	1	1	1	8.9	4.5	10.4
6693	0	0	1	0	1	11.4	4.6	11.9
6694	0	0	1	0	1	18.1	4.36	11.3
6695	0	0	1	1	2	11.9	4.15	10.6
6696	0	0	1	0	1	12	4.31	11.2
6697	0	0	1	1	1	11.3	4.17	10.8
6698	0	0	1	1	1	8.4	4.84	12.8
6699	0	0	1	0	5	9.8	4.27	11.9
6700	0	0	1	1	1	13.9	5.34	10.3
6701	0	0	1	0	1	11.8	4.07	10.6
6702	0	0	1	1	1	9.6	4.77	12.2
6703	0	0	1	1	2	8.2	4.56	12.6
6704	0	0	1	0	3	18.9	4.1	10.5
6705	0	0	1	1	1	12.7	4.59	9
6706	0	0	1	0	1	15.6	4.03	10.7
6707	0	0	1	1	3	8.6	3.86	10.3
6708	0	0	1	0	3	15.7	4.1	10
6709	0	1	1	1	1	12.5	4.27	11.3
6710	0	0	1	1	1	9.6	4.47	11
6711	0	1	1	0	1	10.5	4.59	12.4
6712	0	0	1	1	1	14.6	4.7	11.9
6713	0	0	1	1	1	14.7	4.37	11.3
6714	0	1	1	1	2	17.2	4.5	12.1
6715	0	1	1	1	1	12	5.03	13.2
6717	0	1	1	1	1	2.6	4.66	12.3
6718	0	1	1	0	3	14.3	4.24	11.8
6719	0	1	1	0	4	10.9	3.89	10.6
6720	0	0	1	1	7	16.3	4.22	12
6721	0	0	1	0	3	12.8	4.41	11.6
6722	0	1	1	0	2	4.2	4.78	11.9
6723	0	1	1	1	1	19.2	4.56	11.4
6724	0	0	1	1	2	8.8	4.66	9.1
6725	0	1	1	1	4	12.8	3.8	10.7
6726	0	1	1	0	2	10.7	4.08	11
6727	0	1	1	0	3	5.9	4.6	13.1
6728	0	1	1	1	1	18.2	3.47	8.9
6729	0	1	1	1	1	14	4.64	11.8
6730	0	1	1	1	5	12	4.38	12.1
6731	0	1	1	0	4	2.6	4.04	11.6
6732	0	1	1	0	1	16.8	4.05	7.7

6733	0	0	1	1	13	4.4	3.62	9.6
6734	0	0	1	0	0	14	4.31	11.5
6735	0	1	1	1	5	7.7	5.12	10.1
6736	0	0	1	1	0	17.1	4.43	12.1
6737	0	0	1	0	5	5.5	4.2	12.1
6738	0	0	1	0	1	19.7	4.39	9.8
6739	0	1	1	1	2	8.5	4.42	1.4
6740	0	0	1	0	1	12.8	4.1	10.3
6741	0	1	1	1	3	15.2	4.49	12.5
6742	0	1	1	1	4	18.2	4.53	12.6
6743	0	1	1	1	1	12.1	4.16	10.9
6744	0	1	1	1	2	10.5	4.91	9.5
6745	0	1	1	1	1	9.5	3.6	7.2
6746	0	1	1	0	2	14.3	4.19	11.2
6747	0	1	1	1	4	19.1	4.93	10
6748	0	1	1	1	3	17	4.42	11.7
6749	0	1	1	1	1	12.9	4.66	12.9
6750	0	1	1	1	1	19.6	4.47	12.4
6751	0	0	1	1	1	14.2	4.75	12
6752	0	1	1	1	4	10.5	3.81	10
6753	0	0	1	0	2	10.6	3.7	10.5
6754	0	0	1	1	1	12.8	4.55	12
6755	0	1	1	0	10	9	4.3	12.5
6756	0	1	1	1	2	15.4	4.14	11.6
6757	0	1	1	1	2	9.7	4.55	12.7
6758	0	1	1	1	1	17.4	4.53	12
6759	0	1	1	0	2	17.3	4.66	11.8
6760	0	1	1	1	0	16.4	4.63	12.2
6761	0	1	1	0	3	11.1	4.55	12.1
6763	0	0	1	0	2	10.2	3.82	10.6
6764	0	1	1	1	2	6.8	4.47	12.3
6765	0	1	1	1	3	5.9	4.68	12.1
6766	0	1	1	0	2	9.4	4.6	12.2
6767	0	1	1	0	1	14.3	4.36	11.3
6768	0	1	1	0	1	4.9	4.35	12.1
6769	0	1	1	0	1	8.2	4.06	11.6
6770	0	0	1	0	1	9.6	4.79	12.5
6771	0	1	1	1	6	14.8	4.62	13.3
6772	0	1	1	1	2	25.3	3.92	10.6
6773	0	0	1	1	1	17.3	4.4	11.6
6774	0	1	1	0	3	13.6	4.13	11.7
6775	0	1	1	0	3	14.8	3.62	10.1
6777	0	1	1	1	1	15.4	4.44	12.4
6778	0	1	1	0	9	10.5	4.46	12.6
6780	0	1	1	0	7	10.1	4.26	11.1
6781	0	0	1	0	1	12.7	4.16	11.2

6785	0	1	1	1	3	3	4.49	11.8
6786	0	1	1	1	1	20.8	4.41	11.6
6788	0	1	1	1	1	15.3	4.69	12.5
6789	0	1	1	1	2	9.1	4.97	12.6
6790	0	0	1	0	1	6.8	4.1	10.9
6791	0	0	1	0	6	11.5	4.8	12.9
6792	0	1	1	0	1	10.8	4.16	11.8
6793	0	1	1	1	1	11.3	4.64	11.6
6794	0	0	1	1	1	14.4	5.44	11
6795	0	1	1	1	1	9.7	4.78	12.6
6796	0	1	1	0	4	10	3.93	11.1
6797	0	1	1	0	1	13.2	4.1	10.6
6798	0	0	1	1	1	11.3	4.39	11.4
6799	0	1	1	0	2	13.7	4.42	10.9
6800	0	1	1	1	4	12.4	4.17	11.3
6801	0	1	1	0	5	20.5	4.06	11.1
6802	0	1	1	1	4	11.6	4.64	11.9
6803	0	0	1	1	2	16.3	4.35	12.3
6804	0	1	1	0	1	13	4.03	10.7
6805	0	1	1	0	5	14.6	4.19	11.7
6806	0	1	1	1	1	12.5	5.09	9.9
6807	0	0	1	1	2	7.4	4.51	10.8
6808	0	1	1	0	2	8.2	3.96	10.3
6809	0	1	1	0	2	10.2	3.52	10
6810	0	1	1	0	2	12.4	4.14	10.6
6811	0	0	1	0	2	9.7	4.8	12.3
6812	0	0	1	0	2	13.2	3.87	10.6
6813	0	0	1	1	1	13.6	4.83	13.1
6814	0	0	1	0	2	9.2	4.97	9.2
6815	0	0	1	1	2	13.2	4.28	11.2
6816	0	0	1	0	0	14.7	4.55	11.1
6817	0	0	1	1	3	8.9	4.25	11.9
6818	0	0	1	1	3	8.5	4.59	8.9
6936	0	1	1	1	1	9.7	4.36	11.9
6955	1		1	1	1	5.7		10.6
6956	1		1	1	6	5.6		8.8
6957	1		1	0	2	8.4		10.2
6958	1		1	1	5	15.56		28.5
6959	1		1	0	1	10.7		11
6960	1		1	0	3	10		485
6961	1		1	0	1	12.2		10.2
6962	1		1	1	1	10.9		10.5
6963	1		1	0	3	8.4		9.2
6964	1		1	1	1	23		11.3
6965	1		1	0	2	13.6		10.5
6966	1		1	1	6	4.4		11.8

6967	1	1	1	1	15.2	10.7
6968	1	1	0	1	9.1	9
6969	1	1	0	1	3.9	9.7
6970	1	1	1	1	21.4	8.9
6971	1	1	1	2	14.12	12
6972	1	1	1	2	4.7	10.3
6973	1	1	1	3	8.06	12.2
6974	1	1	0	1	10.8	9.7
6975	1	1	1	1	6.7	11.9
6976	1	1	0	0	7	11.1
6977	1	1	0	1	13	10
6978	1	1	0	3	9.02	11.8
6979	1	1	0	0	13.6	10.1
6980	1	1	0	2	21.7	10.8
6981	1	1	1	2	11.1	11.6
6982	1	1	1	5	17.9	11
6983	1	1	1	1	15	11.5
6984	1	1	1	1	9.3	12
6985	1	1	1	2	12.5	10.9
6986	1	1	0	1	15.3	10
6987	1	1	0	3	9.5	12
6988	1	1	1	3	8.2	11.7
6989	1	1	1	1	12.2	10
6990	1	1	0	1	13.9	9.1
6991	1	1	1	1	18.2	10.3
6992	1	1	1	8	3.8	11.7
6993	1	1	0	1	19.9	11.2
6994	1	1	1	1	9.9	10.7
6995	1	1	0	2	8.3	10.4
6996	1	1	0	1	9.7	10.5
6997	1	1	1	1	8.9	11.7
6998	1	1	1	1	8.9	9.9
6999	1	1	0	0	15.4	10.2
7000	1	1	1	1	15.1	10.8
7001	1	1	1	7	20.2	10
7002	1	1	1	1	13.2	9.1
7003	1	1	1	15	6.1	12
7004	1	1	1	1	16.9	11.4
7005	1	1	0	1	6.7	8.3
7006	1	1	1	1	6.2	8.3
7007	1	1	0	1	15.49	9.1
7008	1	1	1	1	12.61	11.4
7009	1	1	1	5	11.12	10.4
7010	1	1	1	2	15.57	11.1
7011	1	1	0	2	12.6	9.6
7012	1	1	1	3	11.5	10.2

7013	1	1	0	1	12.6	10.6
7014	1	1	1	1	7.4	7.8
7015	1	1	1	2	13.4	11
7016	1	1	0	2	11.8	9.4
7017	1	1	1	1	10.2	11.1
7018	1	1	0	2	8.4	10.9
7019	1	1	1	3	12.2	12.1
7020	1	1	1	0	7.9	10.5
7021	1	1	1	1	15.2	10.1
7022	1	1	1	0	9.8	10.5
7023	1	1	1	6	35.34	12.2
7024	1	1	0	1	22.65	10.2
7025	1	1	1	4	9.5	12.3
7026	1	1	0	2	7.1	15.1
7027	1	1	1	1	15.6	11.4
7028	1	1	1	1	8.1	12.4
7029	1	1	1	2	8.6	12.3
7030	1	1	1	1	8.5	12.9
7031	1	1	0	1	14.6	11.6
7032	1	1	0	1	12.9	10.3
7033	1	1	1	4	14.4	10.5
7034	1	1	1	2	10.8	9.4
7035	1	1	1	1	15.25	9.6
7036	1	1	1	1	12.8	11.5
7037	1	1	1	4	11.3	11.3
7038	1	1	0	1	18.5	9.7
7039	1	1	0	1	16.5	9.8
7040	1	1	0	3	4.9	9.8
7041	1	1	0	1	9.7	12.5
7042	1	1	1	1	16.7	10.8
7043	1	1	1	0	16.8	9.7
7044	1	1	1	1	13.5	10.3
7045	1	1	0	1	12.7	11.7
7046	1	1	0	3	10.9	11.2
7047	1	1	0	3	5.66	11.1
7048	1	1	1	1	20.5	10.9
7049	1	1	1	1	9.3	11.7
7050	1	1	1	0	8.59	9.9
7051	1	1	0	2	15.1	12.6
7052	1	1	1	1	22.05	10.7
7053	1	1	0	2	10.34	10.2
7054	1	1	1	4	14.48	11.7
7055	1	1	1	1	17.19	11.5
7056	1	1	0	3	9.7	11.8
7057	1	1	1	2	10.5	11.2
7058	2	1	0		18.54	10.8

7059	2	1	0	7.95	10.9
7060	2	1	1	16.1	11
7061	2	1	1	15.71	11.5
7062	2	1	1	15.26	9.5
7063	2	1	0	18.59	10.8
7064	2	1	0	19.7	9.8
7065	2	1	0	14.96	9.7
7066	2	1	1	21.63	9.4
7067	2	1	1	25.19	10.7
7068	2	1	1	20.64	11.9
7069	2	1	1	14.78	11.8
7070	2	1	0	18.01	11.3
7071	2	1	1	18.49	11.1
7072	2	1	1	13.25	12
7073	2	1	0	12.07	10.7
7074	2	1	1	12.21	11.4
7075	2	1	0	7.9	12.7
7076	2	1	1	5.75	11.2
7077	2	1	1	14.45	12.2
7078	2	1	0	16.37	10.8
7079	2	1	1	4.29	12.6
7080	2	1	1	12.49	11
7081	2	1	0	6.95	11.6
7082	2	1	1	11.45	9.3
7083	2	1	0	16.81	12.1
7084	2	1	0	21.45	9.3
7085	2	1	1	21.01	11.1
7086	2	1	0	14.25	9.2
7087	2	1	1	17	11.5
7088	2	1	1	10.72	10.1
7089	2	1	0	13.88	11.8
7090	2	1	1	9.42	11.3
7091	2	1	1	12.09	11.3
7092	2	1	1	13.88	10.9
7093	2	1	1	16.28	10.5
7094	2	1	0	11.84	9.2
7095	2	1	0	22.71	11.4
7096	2	1	1	16.15	10
7097	2	1	0	10.59	9.6
7098	2	1	0	15.57	11.2
7099	2	1	1	11.16	9.7
7100	2	1	0	15.98	10.6
7101	2	1	0	14.24	10.4
7102	2	1	1	14.94	10.2
7103	2	1	0	13.04	11.1
7104	2	1	1	9.79	11.5

7105	2	1	0	7.46	8.8
7106	2	1	0	6.75	10.6
7107	2	1	0	8.53	10.6
7108	2	1	1	26.42	11.1
7109	2	1	0	15.87	11.5
7110	2	1	1	6.62	11.6
7111	2	1	1	14.17	9.2
7112	2	1	1	11.27	11.5
7113	2	1	1	5.97	12.2
7114	2	1	1	16.35	10
7115	2	1	1	3.46	11.9
7116	2	1	1	14.79	10.8
7117	2	1	1	17.31	10
7118	2	1	0	12.57	9.8
7119	2	1	1	10.44	10.4
7120	2	1	1	14.68	10.2
7121	2	1	1	16.61	10.3
7122	2	1	0	15.36	10.8
7123	2	1	1	9.49	10.6
7124	2	1	0	5.03	11.3
7125	2	1	1	14.85	11.5
7126	2	1	1	4.53	9.9
7127	2	1	1	9.49	14
7128	2	1	1	17.08	11.5
7129	2	1	0	13.67	12.1
7130	2	1	0	18.08	10
7131	2	1	0	9.61	10.5
7132	2	1	1	6.71	12.5
7133	2	1	0	7.24	11.1
7134	2	1	1	2.85	12.4
7135	2	1	1	9.72	10.2
7136	2	1	1	10.12	12.1
7137	2	1	0	16.33	9.9
7138	2	1	1	13.77	10.3
7139	2	1	1	12.04	9.9
7140	2	1	1	20.56	9.1
7141	2	1	1	19.23	9.3
7142	2	1	0	14.55	12.1
7143	2	1	1	8.5	11.8
7144	2	1	0	13.33	11.5
7145	2	1	1	16.9	10.9
7146	2	1	0	17.48	14.2
7147	2	1	1	20.97	11
7148	2	1	1	15.98	11
7149	2	1	1	15.66	10.7
7150	2	1	1	12.31	10.5

7151	2	1	1	19.67	11.5
7152	2	1	1	9.68	11.8
7153	2	1	0	10.08	11.2
7154	2	1	1	8.36	11.4
7155	2	1	1	10.01	12.1
7156	2	1	0	17.99	9.7
7157	2	1	1	16.72	10.5
7158	2	1	0	17.85	11.3
7159	2	1	1	12.42	9.5
7160	2	1	0	6.7	11.5
7161	2	1	0	7.85	11.9
7162	2	1	1	19.07	9.9
7163	2	1	1	10.81	11.3
7164	2	1	0	15.35	11.5
7165	2	1	0	8	11.1
7166	2	1	1	8.34	10.5
7167	2	1	1	6.58	10.8
7168	2	1	1	13.46	10
7169	2	1	1	22.23	12.1
7170	2	1	1	19.5	9.2
7171	2	1	0	27.95	10.9
7172	2	1	0	20.72	9.3
7173	2	1	0	11.54	10
7174	2	1	0	9.96	10.9
7175	2	1	0	15.26	10.7
7176	2	1	0	17.48	10.9
7177	2	1	1	20.53	9.8
7178	2	1	1	13.79	9.2
7179	2	1	1	19.1	9.7
7180	2	1	0	19.56	11.1
7181	2	1	0	10.02	9.3
7182	2	1	1	38.74	11.9
7183	2	1	1	11.4	10.7
7184	2	1	1	11.9	11
7185	2	1	0	22.19	10.2
7186	2	1	1	11.67	10.6
7187	2	1	0	13.89	10.5
7188	2	1	1	5.88	9.2
7189	2	1	1	18.8	7.8
7190	2	1	0	14.62	8.1
7191	2	1	0	17.09	10.7
7192	2	1	0	6.59	10.5
7193	2	1	0	20.54	11.9
7194	2	1	0	10.86	11.6
7195	2	1	0	22.75	10.5
7196	2	1	1	25.74	11

7197	2	1	0	19.51	9.9
7198	2	1	0	11.8	10.2
7199	2	1	1	4.18	11.5
7200	2	1	1	24.1	10.3
7201	2	1	1	14.38	11.1
7202	2	1	1	10.26	9.1
7203	2	1	0	6.42	8.3
7204	2	1	1	8.51	8.8
7205	2	1	0	16.36	10.8
7206	2	1	1	7.28	11
7207	2	1	1	26.39	11.4
7208	2	1	1	16.57	9.2
7209	2	1	1	10.69	11.5
7210	2	1	1	13.29	12.3
7211	2	1	1	13.88	11.3
7212	2	1	0	14.13	11.8
7213	2	1	0	11.51	11.8
7214	2	1	1	15	12.2
7215	2	1	1	23.06	9.2
7216	2	1	0	9.42	10.7
7217	2	1	0	16.22	12.7
7218	2	1	1	13.44	11
7219	2	1	0	12.61	9
7220	2	1	0	14	10.4
7221	2	1	1	21.6	10.7
7222	2	1	1	15.89	9.6
7223	2	1	1	21.97	10.8
7224	2	1	0	15.16	10.7
7225	2	1	0	7.25	9.2
7226	2	1	0	21.71	12.3
7227	2	1	1	8.34	12.1

MCV (fL)	MCH (pg/	MCHC (g	Platelets (	Segment ('	Lymphocy	Monocyte	Eosinophil	Basophil (
80.2	24.4	30.4	271	81	9	5	0	0
83.7	27.5	32.8	257	37.6	49.3	10.6	1.9	0.6
78.5	26	33.1	312	54	31	10	0	0
79.7	26.7	33.5	345	45.2	41.2	11.9	1.2	0.5
82.4	28.8	34.9	194	72	22	3	0	0
81.6	27	33.1	269	78.8	13.9	6.6	0.3	0.4
79.8	26.9	33.7	315	82	12	5	0	1
76.9	25.1	32.6	391	49.4	40.4	6.8	3.2	0.2
77.4	26.2	33.9	301	82.1	13.8	3.7	0.3	0.1
76.7	26.6	34.6	205	57.8	36.8	4.7	0.6	0.1
77.5	25.6	33	163	73.1	17.4	8.8	0.6	0.1
70.6	23.5	33.2	326	62	32	5	0	0
81.4	28.1	34.5	269	73	17	6	2	0
82.4	28.5	34.5	366	50.8	34.3	6.9	7.5	0.5
83.3	27.8	33.3	278	84	10	6	0	0
83.5	28.3	33.9	306	55	23	22	0	0
83.5	28.8	34.5	271	76	17	7	0	0
71.5	24.5	34.2	310	58.5	23.5	9.1	8.6	0.3
81.5	28.2	34.6	468	91	2	6	0	0.5
81.6	27.6	33.9	224	71	15	4	1	0
84.4	29	34.3	275	74.9	15.1	9.7	0.1	0.2
80.2	26.7	33.3	199	66.1	25.8	7.7	0	0.4
77.7	26.6	34.2	191	48.7	38	12	0.3	1
77.8	26.8	34.5	236	73.4	19.1	7.1	0.2	0.2
79.3	26.9	34	421	61.7	27.9	9.9	0.3	0.2
79.3	26.7	33.7	149	48.2	43.7	7.9	0	0.2
79.8	26.7	33.4	311	53.3	35.2	11.3	0.1	0.1
79.9	27	33.8	224	70.5	21.8	7.1	0.4	0.2
86.1	28.8	33.5	210	53.4	32	13	0.8	0.8
69.9	23.2	33.2	180	48	45	6	0	0
85.7	29.7	34.6	412	89.5	5	5	0	0
83.2	27.7	33.3	179	65.4	28.1	5.4	1	0.1
81.9	27	32.9	125	66.9	22	10.9	0.1	0.1
81.7	26	31.8	417	68	24.9	6.1	0.6	0.4
78.5	27	34.4	363	62.7	27.7	8.5	0.9	0.2
74.9	26.5	35.4	200	52.4	42	5.4	0.1	0.1
79.6	27.4	34.5	114	69.5	23.6	6.5	0.2	0.2
78	26.4	33.9	240	79.8	15.3	4.8	0	0.1
79.7	28.1	35.2	168	67.2	26	6.2	0.3	0.3
79.4	26.5	33.3	205	72.6	19.8	7.2	0.2	0.2
80.6	26.9	33.4	215	46.6	46.9	5.9	0.2	0.4
79.3	27.4	34.5	217	58	26	9	0	0
82.7	27.7	33.5	278	72	21.6	6.2	0	0.2
75.6	25.5	33.7	173	75.2	16.7	7.8	0.1	0.2
84.7	28.2	33.3	200	72.1	22	5.4	0.4	0.1

80.6	28.1	34.8	218	78.3	12.7	8.7	0.1	0.2
80.5	28.1	34.9	200	55.1	39.5	4	0.9	0.5
80.9	27.3	33.7	209	75.8	17.3	6.6	0.1	0.2
81.5	28.2	34.5	243	70.4	23.8	5.5	0.1	0.2
76.4	26.1	34.1	151	71.7	17.1	9.4	1.5	0.3
82.6	28	34	328	80.9	13.7	4.7	0.5	0.2
78	26.8	34.4	194	74.7	19	5	1.1	0.2
82.4	27.5	33.4	374	84.3	9.3	6.3	0	0.1
78.8	27.6	35.1	251	63	27	10	0	0
81.5	28	34.4	249	76.3	13.2	4	6.4	0.1
80	27.4	34.2	206	52.6	33.2	5.9	8.1	0.2
77.8	26.7	34.3	266	36.7	50.9	10.8	1	0.6
79.5	27.8	34.9	209	66.7	23.5	9.7	0	0.1
83.5	28.3	33.9	225	63.4	30.8	4.7	0.5	0.6
80.5	28.1	35	334	50.3	38.1	9.7	1.1	0.8
77	26.6	34.6	139	51	32	3	0	0
77.9	26.8	34.3	248	72.6	19.5	7.8	0	0.1
77.6	25.9	33.3	192	47	39	14	0	0
80.9	28.1	34.8	341	85.1	12	2.8	0	0.1
73	25.2	34.5	200	42.5	46	10.2	0.4	0.9
82.4	28	34	245	57.6	33.8	7.5	0.1	1
77.9	27.7	35.5	335	51.2	36.5	11.5	0.4	0.4
81.6	28.2	34.6	305	73.8	19.5	5.7	0.8	0.2
81.2	26.6	32.8	241	61	30	8	1	0
81.3	27.8	34.2	250	69.5	23.2	7	0.1	0.2
75.2	24.8	33	169	33.6	54.7	10.4	0.2	1.1
78.1	26.7	34.2	367	78	17	4	1	0
81.4	27.1	33.2	330	78.8	13.8	7	0.2	0.2
74.3	25.9	34.9	659	65.7	28.1	4.9	0.9	0.4
80.2	26.9	33.5	427	55.7	38.6	5.1	0.4	0.2
77.3	26	33.7	270	56.8	36.8	6.1	0.1	0.2
80.9	27.9	34.5	232	69.5	18.2	12	0.1	0.2
81	27.4	33.9	200	47.1	46.7	5	0.5	0.7
79.2	27.4	34.6	297	54.3	33.8	7.3	4.3	0.3
78.6	26.8	34	379	85.3	10.6	4	0	0.1
82.3	26.4	32	224	20.4	69.6	8.1	1.4	0.5
76.2	25.8	33.9	624	75	12	11	0	0
86.1	29.6	34.4	237	49.3	35.7	12.4	1.4	1.2
79	27.5	34.8	187	66.8	27.7	5.4	0	0.1
77.9	26.8	34.4	279	68	24.1	7.1	0.6	0.2
77.7	25.6	33	306	73.1	17.4	8.9	0.2	0.4
70.5	23.8	33.8	205	83.6	9.8	6.1	0.3	0.2
81.3	26.6	32.8	256	55.5	35.4	8.6	0.2	0.3
81.3	27.3	33.6	199	84.3	10.8	4.6	0.1	0.2
82	28.4	34.6	294	88.6	8.2	3.1	0	0.1
79.9	27.1	34	253	64.2	23.8	10.3	1.5	0.2

79.2	26.9	34	234	69.9	18.4	10.4	1	0.3
86	29.2	33.9	208	72	18	9	0	0
77.1	25.1	32.5	281	63	21	10	0	0
80.5	26.8	33.3	460	63.5	27	9.5	0	0
78.3	26.4	33.8	341	72.6	20	6.8	0.5	0.1
78.8	27.5	35	228	47.8	40.7	10.8	0.3	0.4
79.1	26.5	33.5	194	80	15	5	0	0
74.3	25.2	33.9	206	30	64.3	5.2	0	0.5
67	20.6	30.7	231	48.2	46.8	3.6	1.2	0.2
74.8	25.2	33.7	118	51.5	41.9	6.6	0	0
77.4	26.5	34.3	264	67.2	25.4	6.5	0.6	0.3
80.9	27.5	34	237	43.9	43.4	11.6	0.8	0.3
82.6	24.9	30.2	44	40.2	49	10	0.4	0.4
82.6	28.5	34.5	258	71.4	25.8	2.3	0.1	0.4
78.3	26.6	34	351	44.7	44.3	7.6	3.1	0.3
78.1	27	34.6	250	28	63	6	3	0
77.4	26.6	34.4	244	63.1	29.1	7	0.6	0.2
77.5	25.7	33.2	316	65	13	5	0	0
78.4	26.3	33.6	147	34.7	55.9	6.6	2.4	0.4
80.4	27.2	33.8	484	60.6	32.7	6.2	0.4	0.1
88.3	30.6	34.6	220	56.3	35	6.2	2.2	0.3
80.5	27.8	34.5	318	72	10	16	0	0
76.1	25.6	33.7	311	25.3	65.1	7.2	1.3	1.1
76.7	25.6	33.4	286	60	31	9	0	0
82.4	27.6	33.5	218	42.1	46	11.5	0	0.4
81.5	27.5	33.8	306	80.4	11.9	4.9	2.8	0
81.8	27.3	33.4	152	47	39	8	1	0
78.6	27.3	34.8	305	55	38.4	4.7	1.7	0.2
80.7	27.9	34.6	171	24	66	9	1	0
80.9	26.9	33.2	179	57	18	20	0	0
84.2	28.5	33.9	170	69	24	7	0	0
77.2	26.6	34.4	178	40.8	43.8	13.8	0.2	1.4
80	27.3	34.1	163	59.8	28.6	8.3	2.7	0.6
77.1	25.8	33.4	143	77	5	5	0	0
81.7	28.3	34.6	138	33.7	51.6	14.2	0	0.5
80.2	26.2	32.7	296	73.3	21.2	5.1	0.3	0.1
77	26.4	34.3	258	59	29	11	0	0
80.4	26.9	33.4	336	50.7	42.3	6.3	0.4	0.3
82.2	27.7	33.7	196	45.4	43.3	10.1	0.7	0.5
87.9	30.4	34.5	121	65.7	19.5	8.2	6.2	0.4
78	27.3	34.9	288	68.8	19.7	11	0.3	0.2
78.6	26.4	33.6	304	5	67	18	10	0
78.7	26.8	34	244	52	38	8	0	0
84.2	28.1	33.3	238	73	18	9	0	0
78.2	26.7	34.2	259	42	45	12	1	0
83.1	28.8	34.7	291	64.4	24.7	10.3	0.2	0.4

78.1	26.3	33.7	309	53.9	35	10.9	0	0.2
77.8	27.3	35.1	155	56.1	31.6	12.1	0	0.2
81	27.9	34.4	153	73.5	18.2	8.2	0	0.1
82.2	28	34	260	48.5	41.1	10.1	0.1	0.2
80.3	26.4	32.8	277	59.1	30.1	7.3	3.1	0.4
84.8	29.2	34.4	93	31	53	12	0	0
79.7	27	33.9	187	81.6	12.6	5.6	0	0.2
79	27.2	34.4	160	39	46	13	0	2
71.8	23.9	33.2	271	54	28	16.5	0	0.5
78.6	26.6	33.9	196	65	24	10	0	0
76.4	26.1	34.2	164	43.5	44	11.2	0.9	0.4
79.4	26.5	33.4	100	37.8	52.5	8.9	0.6	0.2
74.6	24.8	33.2	373	75	13	10	0	0
79.5	27.7	34.8	260	68.3	24.9	6.4	0.3	0.1
76.8	25.7	33.5	259	51.6	38.9	9.1	0.2	0.2
84.1	28.9	34.3	280	75.4	14.5	9.8	0.1	0.2
83.6	27.1	32.4	275	45	41	13	0	1
78	26.4	33.9	263	43.7	50.5	5.1	0.4	0.3
73.2	25.2	34.4	47	53.2	36.6	9	1	0.2
84.4	28.6	33.8	242	52	22	23	0	0
83.4	29.2	35	279	84.7	9.3	3.8	2.1	0.1
69.2	24.2	35	257	41	47	7	2	0
79.1	27.3	34.6	136	79.4	15.8	4.7	0	0.1
77.4	25.9	33.5	207	31.3	52.9	10.5	4.1	1.2
83.3	28.1	33.7	427	60	32.6	6.9	0.1	0.4
81.7	27.1	33.1	219	58.8	30.6	9.5	0.5	0.6
78.6	27.1	34.4	516	60	30	8.4	1.3	0.3
79.1	26.9	34.1	187	72.8	18.4	7.2	1.2	0.4
77.5	24.6	31.7	554	59.9	21	18.6	0.1	0.4
77.3	26	33.6	419	55.1	36.1	7.3	1.2	0.3
79.3	27.8	35.1	358	74	18	8	0	0
82.3	27.4	33.2	387	57.5	32	9.7	0.4	0.4
76	26.5	34.9	301	71.3	19.2	9	0.2	0.3
81.9	27	33	255	44	51	3	0	1
79.6	26.5	33.2	154	23.9	68.1	7.3	0.2	0.5
75.8	26.2	34.6	281	31.8	59.9	7.8	0	0.5
84.7	27.9	33	252	10	69	15	2	0
81.9	28.8	35.2	193	56	30	8	2	1
81.8	28.1	34.3	179	71.4	16.4	11.8	0.3	0.1
81.1	25.7	31.6	231	83	9	7	0	0
77.8	27.2	35	304	51.4	32.9	15	0.1	0.6
86.9	29.7	34.2	183	80	11	8	0	1
79.4	26.6	33.5	288	53.7	37.3	7.8	0.8	0.4
78.8	27.8	35.2	190	61.5	32.4	6	0	0.1
78.2	26.7	34.1	123	48	31	10	0	0
85.4	28.3	33.1	152	32	58	9	0	1

79.9	27.7	34.6	244	81.7	15.7	2.4	0.1	0.1
76.5	26.2	34.2	161	81.5	14.5	4	0	0
73.2	25.1	34.4	304	44.9	41	13.7	0	0.4
79	27.1	34.3	165	62.2	30.6	6	0.9	0.3
76.7	26.1	34	429	52.9	39.7	4.7	2.3	0.4
79.5	27.5	34.5	233	57	29	14	0	0
80.5	27.6	34.3	351	79	14	7	0	0
79.5	26	32.6	221	53.5	31.2	15	0.1	0.2
80.2	28.4	35.4	205	53.3	36.9	9.2	0.4	0.2
79.5	26.1	32.8	380	64.7	29.1	5.7	0.3	0.2
79.9	26.3	32.9	243	55.2	35.3	9.1	0.1	0.3
73.5	25.7	35	308	58.7	35.8	5	0.3	0.2
78.8	26.4	33.5	211	52.2	37.4	9.2	1	0.2
80.4	25.6	31.9	170	65	24	11	0	0
78.2	26.6	34	216	48.8	39.5	11	0.2	0.5
75.3	25.4	33.8	236	32.8	59.3	5.7	0.3	1.9
87	28.8	33	527	59.7	24.8	13.3	1.9	0.3
80.6	27.8	34.5	187	45.7	43.5	10.3	0	0.5
81.3	27.9	34.3	117	64.8	25.6	9.2	0.2	0.2
76.9	25.9	33.7	241	49.4	40.7	8.9	0.8	0.2
76	25.7	33.8	246	62.6	29.9	6	1.4	0.1
68.4	21.9	32.1	303	39.9	50.9	8.6	0.1	0.5
77.9	27.6	35.4	355	34.3	53.5	7.2	3.2	1.8
77.7	27.8	35.8	278	71.7	15.6	11.9	0.4	0.4
77.4	26.4	34.1	270	35.4	54.6	9	0.5	0.5
80.3	27.8	34.6	287	64.1	26.9	8.9	0.1	0
75.4	26	34.5	240	46.4	39.5	13.2	0.1	0.8
80.1	27.4	34.2	339	53	38	6	3	0
67.5	21.4	31.7	406	42.4	44.1	12.3	1	0.2
75.8	26.1	34.3	191	40	39	13	0	0
79.5	27.7	34.8	260	48.2	40.3	10.2	0.7	0.6
79.1	26.4	33.4	199	84	7	6	0	0
80.6	27.5	34.1	171	81.1	14.1	4.4	0	0.4
77.2	25.2	32.7	152	58	29	12	0	1
85.6	28.5	33.2	539	64.5	26	9	0	0
86.2	29.2	33.9	213	37	41	14	0	1
78.5	27.4	34.9	291	70	11	17	0	0
77.5	25.5	32.9	261	59	33	8	0	0
77.7	27	34.8	277	67.5	22.8	9.5	0	0.2
80.5	27.2	33.9	240	38.8	49.8	8.9	1.9	0.6
81.5	26.8	32.9	148	46.9	44.4	8.3	0.2	0.2
81.4	27.6	33.9	330	46.8	31.2	19.3	2.4	0.3
89.4	30.7	34.3	476	61.9	24.2	12.2	1.5	0.2
77	27.1	35.2	176	35.1	57.6	6.1	0.7	0.5
82.7	28.8	34.8	182	20.8	66.9	10.7	0.2	1.4
76.7	26.8	34.9	288	78.8	14.8	5.7	0.6	0.1

80.1	26.4	33	275	26.3	55	14.9	3	0.8
79.8	27.3	34.2	231	32.7	60	6.5	0.3	0.5
96.3	33.6	34.9	354	24	63	9	4	0
78.4	25.9	33	191	52.5	39.1	6.2	2	0.2
73.8	24.1	32.7	192	54.7	36.5	8.2	0.2	0.4
80.8	27.9	34.6	244	29	65	5	0	0
82	28.1	34.3	382	68.1	24.5	5.9	1.4	0.1
80.7	26.9	33.3	230	50	41	8	1	0
81.2	27.9	34.4	310	59.6	30.5	9	0.2	0.7
86.1	28.8	33.5	465	68.8	25.2	5.7	0.1	0.2
76.3	24.8	32.6	322	39	51	9	1	0
71.9	24.2	33.6	153	41	46	13	0	0
80.7	27.4	33.9	271	49	43	8	0	0
75.7	25.8	34.1	184	50	28	20	0	0
76.3	26.4	34.6	169	31	45	22	1	0
76	25.7	33.8	419	28.7	54.9	13.8	2.3	0.3
80.1	26.7	33.3	153	60.6	33.6	5.5	0	0.3
92	30.3	32.9	396	30.6	56.2	12.9	0.1	0.2
81	27.3	33.8	248	66	27	4	0	1
84.9	28.4	33.4	251	69.5	23.3	6.9	0.2	0.1
86.7	29.3	33.8	390	16	74.8	7.2	1.9	0.1
82.1	27.4	33.3	484	50.8	42.6	5.6	0.3	0.7
78.2	25.1	32.1	233	76	16	8	0	0
73.6	24.9	33.8	258	66	27.6	4	2.3	0.1
76.4	25.9	33.9	159	58.1	35.3	6	0.3	0.3
83.1	28	33.7	454	88.3	6.8	4.6	0.2	0.1
86.5	28.9	33.4	288	42	33	11	0	0
81.4	27	33.2	184	77	18	5	0	0
93.9	29.4	31.3	547	52.5	38.5	2.5	5.5	0
79.2	27.1	34.2	157	70.7	23.2	5.9	0	0.2
81.4	28.1	34.4	259	50.6	33.9	14.3	0.7	0.5
81.8	27.7	33.9	180	78.6	9.2	12	0.1	0.1
70.9	23.3	32.9	243	75.9	19.4	4.6	0	0.1
80.9	26.2	32.4	200	49.8	41.8	8.2	0	0.2
76.9	25.8	33.6	394	52	41	3	0	0
80.5	27.4	34.1	144	58.7	32.8	8.2	0.1	0.2
78.2	26.1	33.3	199	69.5	22.9	6.7	0.7	0.2
77	27.1	35.1	272	41.5	44.5	8	0.5	0
69.6	22.7	32.6	254	59.4	29.5	10.7	0.3	0.1
78.4	27.3	34.8	251	75.5	14.4	9.7	0.2	0.2
79.2	26	32.8	465	50.2	39.2	9.4	0.7	0.5
76.8	26.6	34.6	296	67.4	27.5	4.3	0.6	0.2
74.9	24	32	238	53.6	38.5	5.7	1.6	0.6
74.9	24	32	174	60.3	27.9	11.4	0.3	0.1
76.4	27.7	36.2	211	56.6	35.8	6.7	0.6	0.3
84.2	28.3	33.6	216	51.7	34.4	9.4	4	0.5

74.2	24.4	32.9	460	73	21	6	0	0
83.3	29.6	35.5	225	80.1	13	6.6	0.2	0.1
80	27.9	34.9	221	72.7	17.4	6.5	3.3	0.1
81.1	26.8	33	188	55	39	6	0	0
81.6	26.7	32.7	357	30	64	2	4	0
79.4	26.7	33.6	578	41.1	45.6	12	1	0.3
81.4	26.9	33.1	204	47	44	7	0	0
79.4	26.4	33.2	113	44.7	47.3	7.1	0	0.9
79.4	27.4	34.5	128	76.2	13.8	9.9	0	0.1
76.5	26.5	34.6	188	62	28	4	5	0
78.7	28.4	36.1	212	70	20	7	3	0
79.2	27.8	35.1	185	37.3	49.9	10.3	2	0.5
78.1	27.2	34.8	495	49	45	5	1	0
76.6	24.7	32.3	227	59	26.9	12.6	0.9	0.6
85.6	29.5	34.5	164	66.1	23.2	10.1	0.2	0.4
79	28.4	36	174	54.8	37.8	6.1	0.2	1.1
78.8	27	34.3	263	63	27	7	1	0
77.7	26.2	33.8	135	31.6	59.9	7.7	0.3	0.5
73.6	24	32.5	408	63.7	30.2	5.3	0.4	0.4
78.7	27.3	34.7	264	91.5	5	3.5	0	0
79.9	26.3	32.9	268	55.1	35.2	8.8	0.6	0.3
78.5	26.5	33.8	237	64	27.5	7.4	0.6	0.5
76.9	25.9	33.7	226	24	54	12	0	1
98	33.7	34.4	350	35	44	13	4	1
73.7	24.4	33.1	246	46.1	46.8	6.6	0.2	0.3
79.1	28	35.4	411	71	15	11	0	0
79.5	25.5	32.1	170	43	22	11	0	0
80.4	27.4	34.1	380	20.8	65.8	12.3	0.7	0.4
76.4	25.5	33.3	188	58.7	35.6	5.2	0	0.5
78.9	26.1	33.1	158	69.7	16.5	13.5	0.1	0.2
82.1	27.3	33.2	241	52	39	6	2	0
95.5	33.3	34.9	461	83	14	2	0	0
89.9	30.7	34.1	466	60.2	28.8	9.9	0.7	0.4
80	27.2	34.1	443	59.7	30.1	8.9	1.1	0.2
78.5	27.5	35	154	65	20	15	0	0
74.9	26.4	35.2	162	72.9	16.9	9.9	0.1	0.2
82.6	28.2	34.1	133	69	18	12	0	0
74.3	25.3	34	198	76.2	15.7	7.7	0.1	0.3
81.5	27.1	33.2	274	77.1	13	9.3	0.5	0.1
82.2	28.3	34.4	382	81	13	6	0	0
78.8	27.6	35	294	81.8	8.4	5.5	4.2	0.1
78.9	26.7	33.9	354	60.9	32.6	5.6	0.7	0.2
78.4	25.1	32	171	42	51.7	6.1	0	0.2
80.5	27.3	33.9	399	75	10	13	0	0
88	30.5	34.7	365	43.9	44	10.5	1.3	0.3
78.4	27.8	35.5	190	89	8	3	0	0

80.5	28.3	35.2	220	62	18	15	0	0
78.2	26.4	33.8	226	67.4	21	7.1	4.3	0.2
84.3	28.5	33.8	314	59.4	35	5.1	0.2	0.3
83.5	28.3	33.9	421	81	8	6	0	0
83.5	28	33.5	253	67	20.2	8.6	3.6	0.6
85.1	28.5	33.5	191	48.8	39.6	11.1	0.2	0.3
74.7	26.3	35.3	299	63.4	29.4	5.7	1.1	0.4
78.3	26.5	33.8	144	63.2	29.7	5.9	0.7	0.5
78.8	26.7	33.9	199	75	17	7	0	1
79.5	28.3	35.6	295	81	12.3	4.7	1.8	0.2
77.5	25.8	33.2	197	49.3	41.3	9	0	0.4
75.4	25.6	34	384	60	19	19	0	0
76.9	25.9	33.6	206	42.5	47.1	9.8	0.2	0.4
85.1	28.2	33.1	271	67	23	10	0	0
81.2	27.2	33.5	182	26.7	66	7.3	0	0
100.2	34.9	34.8	474	46	46	6	2	0
74.5	25.3	33.9	323	55.1	34.8	9.9	0.1	0.1
77.6	26.6	34.3	300	55	19	19	1	0
80.1	28.1	35.1	199	68	20	12	0	0
86.7	29.1	33.6	246	85.5	10	4.5	0	0
78	26.9	34.5	368	33.3	55.2	4.3	6.9	0.3
76.7	25.7	33.5	352	74.6	17.6	7.5	0.1	0.2
78.8	26.7	33.9	165	60	29	5	0	0
83.1	29.4	35.4	295	73.5	23.2	3	0.1	0.2
79.3	25.4	32	237	42.5	48.5	8.6	0	0.4
84.5	28.1	33.2	223	39.8	51	8.8	0.2	0.2
81	27.4	33.9	410	59.5	33.1	7	0.1	0.3
96.8	32	33.1	248	11	89	0	0	0
75.7	26.6	35.1	193	56.1	36.2	6.9	0.4	0.4
79.7	28.3	35.6	157	66.7	24.7	8.4	0.2	0
81.8	28.4	34.7	141	56.8	31.6	11.2	0	0.4
78.2	26.5	33.8	306	76.5	18	5	0.5	0
81.5	27.7	34	226	75.5	16.3	6.5	1.2	0.5
72.5	24.5	33.8	307	38.6	44.9	12.6	3.7	0.2
73.2	25	34.1	282	68	24.5	5.5	0	0
70.6	23.5	33.3	391	64.5	32.7	1.9	0.7	0.2
78.6	27.3	34.7	126	75.7	18.2	5.9	0	0.2
75.4	25.1	33.3	480	54.4	36.6	8	0.7	0.3
73.3	24.2	33	220	18	52	24	0	1
81.1	27.6	34.1	166	55.2	34.5	9.7	0.2	0.4
85.1	27.6	32.4	492	83.5	10.5	5	0	0
83	28.1	33.9	335	82	12.5	5	0	0
76.5	25.3	33.1	344	40	47.6	5.6	6.4	0.4
77.2	26.7	34.6	222	51.3	36.7	8.6	3	0.4
104.8	36.2	34.5	245	40	45	14	0	0
75.4	25.3	33.5	389	70.4	22.1	6.8	0.6	0.1

80.5	27.5	34.2	195	76.8	15.4	4.4	3	0.4
75.6	26.8	35.4	231	46.1	46	5.5	2.2	0.2
82.3	28	34	244	75	15	8	0	0
75.5	25.5	33.7	242	56.1	30.9	12.1	0.7	0.2
92	31.8	34.6	427	53	34.1	11.2	1.3	0.4
83.5	27.9	33.4	204	50	43	5	1	1
78.7	27.2	34.6	228	81.7	9.2	9	0	0.1
79.6	26.6	33.4	661	62	32.6	4.3	0.9	0.2
81.6	26.7	32.7	222	83.6	11.6	3.9	0.7	0.2
98.4	34.3	34.9	536	43	46	8	3	0
76.2	27	35.5	323	66.1	26.8	6.5	0.2	0.4
78.9	25.9	32.8	184	66	19	15	0	0
83.5	28.3	33.8	215	62.4	31.3	5.5	0.6	0.2
77.8	27.1	34.8	447	89.5	5.5	4	0.5	0.5
90.8	31.4	34.6	286	35.3	44.9	16.9	2.6	0.3
77.8	26.7	34.3	198	70	15	15	0	0
91.7	30	32.7	176	28	60	11	0	0
78.8	27.5	34.9	404	55.6	35.7	8.5	0	0.2
83.4	28.7	34.5	284	56	34	6	2	0
79.7	27.1	34	167	61	29	10	0	0
81.4	27.1	33.3	248	51	43	6	0	0
76.6	26	34	236	62.8	26.4	9.7	0.8	0.3
79.9	25.8	32.3	392	71.5	22.1	5.4	0.8	0.2
81.5	28.8	35.3	278	57.7	28.2	13.5	0.4	0.2
78.2	26.4	33.8	236	58	27	7	1	0
82.4	28.9	35.1	218	54.6	32.2	11.3	0.9	1
84.2	28.3	33.6	174	76	17	6	1	0
72	24.7	34.2	194	57	32	9	0	0
82.6	28.1	34.1	453	47.2	49.1	2.3	1.2	0.2
81.8	27.1	33.1	153	77.2	16.4	6.1	0.2	0.1
86.3	28.2	32.6	495	62	30	4	0	0
81.7	28.1	34.4	216	39	48	10	2	1
83.4	28.6	34.3	144	80.1	12	7.7	0.2	0
80.5	28.3	35.2	240	85	9	6	0	0
78.1	26.1	33.4	236	28	63	8	0	1
74.2	24.2	32.6	300	63	25	12	0	0
80.6	28.6	35.5	168	49.3	39.9	10	0.3	0.5
78.2	27	34.6	393	54.5	36.5	7	1.5	0.5
81.2	26.7	32.9	303	36	55	3	6	0
83.7	28.5	34.1	216	72.5	21.1	5.4	0.8	0.2
96.1	32.5	33.8	247	65.9	23.7	9.8	0.3	0.3
85.1	29	34.1	229	52	37	10	1	0
79	26.9	34	328	50	38	12	0	0
77.6	25.8	33.2	252	56.5	33.4	9.5	0.4	0.2
66.5	21.7	32.6	220	71	21	6	0	0
79.8	25.9	32.4	174	53.8	28.1	17.7	0	0.4

78.1	27.5	35.2	210	72.5	14.6	11.8	0.9	0.2
84.1	28.3	33.7	352	49	39	7.5	3.5	0
74.7	25.5	34.2	353	52	40	7	1	0
76.9	26.1	34	227	42	43	12	0	1
90.2	31.2	34.6	203	66	12	5	0	0
89.4	31	34.7	524	22.2	66.7	5.2	5.4	0.5
70.9	23.9	33.7	266	53.2	38.4	7.5	0.8	0.1
77.3	26.8	34.6	128	39	48	11	0	1
83.1	27.8	33.4	245	61	29	9	1	0
88.4	29.9	33.8	332	33.8	48.2	6.9	10.8	0.3
80.4	28.3	35.2	312	90	4	6	0	0
78.4	26.9	34.3	515	75.5	19.5	4.5	0.5	0
83.3	26.7	32.1	223	52.6	39.7	6.3	0.2	1.2
76.6	25.7	33.5	137	61	32	6	0	0
84.3	27.6	32.7	326	75.1	13.5	11	0.2	0.2
81.8	27.8	33.9	212	42	45	12	1	0
78	27.5	35.3	208	78.9	12.4	8.6	0	0.1
77.2	27.1	35.1	282	53.7	37.8	6.9	1.3	0.3
75.8	25.1	33	194	60	23	13	1	1
75	25.6	34.2	200	53.7	33.8	12.1	0.2	0.2
82.2	26.7	32.5	548	50	38	9	2	0
77.7	26.1	33.6	189	20	39	4	0	0
79.3	27.5	34.7	316	58.2	33.9	6.9	0.9	0.1
79.3	26.9	33.9	243	51.3	32.9	15.5	0.1	0.2
75.2	24.6	32.7	284	59	25	12	2	0
79.9	26.5	33.2	273	50.9	35.9	6.6	6.3	0.3
82.3	27.7	33.7	227	58	34	6	0	0
78.4	27.2	34.7	129	45.9	39.3	7.7	6.1	1
78.5	26.6	33.9	220	61.1	31.4	7.1	0.1	0.3
78	27.9	35.8	267	73.7	17.6	8.6	0	0.1
72.7	25.4	34.9	391	37	51	8	1	1
80.5	28.6	35.6	346	43.6	48.6	5.3	2.3	0.2
80.7	27.8	34.4	182	74.8	16.6	8.3	0.1	0.2
79.9	27	33.8	178	64.3	26.7	8.7	0.1	0.2
82.9	27.3	32.9	212	81.2	15.4	3.3	0	0.1
86.7	29.2	33.7	596	27	64	6	3	0
79.2	27.8	35	355	53.6	39	6.9	0.3	0.2
78.1	25.8	33	270	28	51	10	6	0
83.6	27	32.3	201	47.2	40.6	10.1	1.8	0.3
78.9	27	34.3	209	67.6	18.8	13	0.2	0.4
79.1	28.5	36	303	73.6	22.8	3.3	0.1	0.2
80.7	26.6	33	196	81.5	13.8	4.6	0	0.1
75.5	25.5	33.8	205	67.4	20.8	10.8	0.5	0.5
84.4	27.8	33	174	44.4	49.1	5.7	0	0.8
89.1	30.8	34.6	476	47	43	8	2	0
78.5	27.6	35.2	151	46.9	41.5	8.3	2.5	0.8

80.4	26.7	33.1	296	63	23	13	1	0
78	26.5	33.9	314	70	17	3	9	0
82.4	28.8	34.9	250	63	25	10	1	0
82.3	27.1	32.9	256	75	10	9	0	0
82.6	28.9	35	305	82.5	10.4	6.9	0.1	0.1
74.4	24.9	33.4	360	66	26.5	6.5	0	0
80.9	28.7	35.4	352	79.2	11.2	7.1	2.3	0.2
80.8	28.3	35	273	84	9	4	1	0
80.9	26.6	32.8	410	74.1	21.1	4.5	0.1	0.2
79.6	27.4	34.4	157	52	30	18	0	0
81.9	27.7	33.9	392	72.8	19	8	0	0.2
86.7	28.5	32.9	348	52.4	40.8	5.1	1.6	0.1
75.8	25.9	34.1	406	74.5	16	7.5	0.5	0.5
74.7	24.7	33.1	468	79.5	12	7.5	0	0
80	27.2	34	244	37	44	19	0	0
80.4	27.3	33.9	262	71	21	5	0	1
74.9	25.3	33.8	246	62	25	10	0	0
85.3	27.6	32.3	146	79.1	15.6	5.2	0	0.1
79.6	27.2	34.2	342	70	18	12	0	0
80.4	27.4	34.1	163	37.7	50.6	9.4	1.8	0.5
88	28.9	32.8	126	76	14.7	9	0	0.3
77.8	26.5	34.1	361	74.6	17.4	7.8	0.1	0.1
89.2	29.3	32.9	349	62.2	30	5.8	1.9	0.1
79.4	26.8	33.7	56	18	66	15	0	0
100.6	34.1	33.8	488	20.2	64.2	5.9	9.4	0.3
82.6	26.8	32.4	350	59.9	33.4	5.1	1.4	0.2
80.4	28.4	35.3	255	45	41	7	4	0
82.6	27.7	33.6	238	81.1	14.4	3.9	0.3	0.3
76.2	25.9	34	305	34	48	6	0	0
81.6	26.7	32.8	133	37	54	9	0	0
85.4	30	35.2	305	79.1	14.1	5.9	0.7	0.2
93.7	32.7	34.9	435	49	31	19	0	1
86.1	29	33.7	128	68	22.6	9.4	0	0
78.8	27	34.3	259	85.1	11.2	2.8	0.8	0.1
80	28.6	35.8	334	32.3	56.2	5.4	5.8	0.3
78.9	26.2	33.2	319	51.4	40.2	3.5	4.5	0.4
79.3	26.4	33.3	558	83.5	9	7	0	0
82.7	27.2	32.9	274	69.9	22.6	7.2	0.1	0.2
81.6	26.7	32.7	281	73.6	21.8	4.5	0	0.1
86.2	29.1	33.8	181	37	56.7	5.7	0.3	0.3
80.8	27.7	34.3	258	61.9	28.7	8.2	0.9	0.3
74.5	25.3	33.9	302	75	16.5	8.2	0.1	0.2
83.8	28.1	33.5	88	45.6	36.6	13.3	3.6	0.9
80.7	27.6	34.1	197	33.7	54.7	10.4	0	1.2
85.8	29	33.8	272	33.6	49.1	14.7	1.8	0.8
76.4	26.3	34.4	141	15	69	8	4	0

81.5	28.3	34.8	184	60	37	1	2	0
75.7	25.6	33.8	223	50.3	42.9	5.3	1	0.5
82.4	27.1	32.9	145	40.8	52.3	6.1	0.4	0.4
79.7	27.7	34.8	212	26.5	58	6	0.5	0
73.6	23.5	31.9	211	19	73	8	0	0
76.3	25.6	33.5	171	57	21	16	0	0
83	28.9	34.7	258	62	30	7.7	0	0.3
77.3	28.5	36.9	135	24	65	9	1	1
79.2	28.7	36.2	182	61	33	6	0	0
83	27.5	33.1	444	74.5	19.5	6	0	0
81.2	28.5	35.1	109	78	15.4	5.7	0.6	0.3
95	31.8	33.4	297	42.4	48.7	4.6	4.2	0.1
82.4	28.1	34.1	369	65.4	25.9	5.6	2.9	0.2
79.3	28	35.4	554	57	33.3	8.7	0.8	0.2
77.2	27.1	35.1	244	61.3	29.2	8.8	0.3	0.4
79.5	26.9	33.8	142	49	38	10	0	1
74.8	25	33.4	300	51.2	40.5	7.8	0.3	0.2
78.5	26.6	33.9	255	54.1	36.2	8.9	0.5	0.3
73.9	25.2	34.1	239	55.1	34	9	0	0.6
80.9	27.5	34	209	62.1	28.8	8.6	0.3	0.2
81	27.7	34.2	199	83.4	13.1	3.4	0	0.1
77.3	26	33.6	269	73.8	19.3	6.3	0.4	0.2
84.4	28.3	33.5	300	67	26	4.5	1.5	0
87.6	28.8	32.8	172	38	49	11	0	0
78.9	27	34.3	388	87.5	8	3.5	0	0.5
78.1	27	34.6	370	72.8	17.8	7.6	1.7	0.1
79.2	26.3	33.2	394	53	39	6	2	0
78.8	26	33	233	61.3	28.9	6.6	2.9	0.3
77.7	26.5	34.2	243	63	28	7	1	0
89	30.1	33.9	287	77.4	14.2	8.1	0.1	0.2
67.2	21.8	32.5	365	46.3	43.6	6.6	3.2	0.3
89.7	30.1	33.6	331	60.3	24.5	13	2.1	0.1
84.1	27.8	33.1	278	87.5	7.9	4.4	0.1	0.1
77.6	27.1	34.9	357	71.9	19.5	7.7	0.7	0.2
82	28.8	35.2	240	45.1	45	6.4	3.1	0.4
82.9	29.3	35.3	209	58.5	31.5	9.7	0	0.3
72.4	24.7	34.2	322	64.1	28.8	6.6	0.2	0.3
82.5	27.5	33.3	275	68	20	12	0	0
86.5	30.6	35.4	242	58.6	32.8	7.5	0.8	0.3
82.7	27.8	33.6	164	52	40.2	7.7	0	0.1
80.6	27.1	33.6	256	75.6	18.9	4.1	1.2	0.2
81.1	26.9	33.1	117	49.8	43	6.8	0.2	0.2
77.5	26.7	34.5	138	79.7	13.1	7	0	0.2
82.4	27.8	33.7	252	76.5	15.8	6.9	0.6	0.2
76.9	25.6	33.2	265	66	24	10	0	0
85.7	28.5	33.2	244	72	20	8	0	0

99.5	33.3	33.5	446	9	73	5	9	0
80	27.7	34.7	332	54	40	5	1	0
76.5	26.3	34.4	235	85.5	8.5	4.5	0	0
75.1	25.4	33.9	221	31	49.8	10.8	7.5	0.9
80.8	27	33.4	208	22	65	12	0	0
81.7	28.5	34.9	203	85.9	10.5	3.3	0.2	0.1
79.6	26.4	33.1	208	62	28	7	0	0
82.5	26.8	32.5	457	54.8	37.7	7	0.1	0.4
74	24.3	32.9	212	55	37	8	0	0
76.9	26.1	33.9	139	60.1	30.3	8.9	0.5	0.2
84.3	29	34.4	275	62.4	29.5	7.2	0.6	0.3
73.9	25.9	35.1	161	62.1	28.2	9.1	0.3	0.3
84.3	27.5	32.7	180	37.7	50.9	10.4	0.5	0.5
77.8	26.1	33.6	302	87.4	9.7	2.7	0.1	0.1
85.9	28.4	33.1	122	19	65	12	0	0
80.5	27.3	33.9	251	41.8	42.7	5.6	9.6	0.3
78	26	33.3	154	71.5	21.5	6.8	0.1	0.1
74.3	25.2	33.9	219	54.5	38.6	6.4	0.1	0.4
79.9	27.7	34.6	199	56.2	28.3	14.7	0.2	0.6
80.3	27.7	34.5	304	24	67	8	1	0
82.4	27	32.7	173	53.9	37.8	7.2	0.9	0.2
79.7	27.1	34	176	69.5	19	11.3	0.1	0.1
76.3	27.2	35.7	137	31.6	54.7	10.8	2	0.9
78.7	26.8	34	178	40	47.4	10.6	1.4	0.6
82.2	27.8	33.8	184	82.3	11.9	5.4	0	0.4
74.6	26	34.8	190	69	20	10	0	0
78.2	26.7	34.1	397	58.5	29.3	10.7	1.3	0.2
99.6	33.7	33.8	382	32.5	47.2	17.6	1.1	1.6
75.9	27	35.5	182	53	35	8	2	0
79.6	27.5	34.6	285	50.5	38.1	11	0	0.4
78.2	27	34.5	326	75.5	19.7	4.4	0.2	0.2
82.6	28.4	34.4	347	85	12.5	2.5	0	0
76.2	27.5	36.1	257	27.3	63.3	8	0.8	0.6
77.3	28.3	36.6	285	82.5	11.2	6.1	0	0.2
81.1	27.8	34.3	119	32.2	59.9	7.3	0.4	0.2
78.1	27.9	35.7	218	53	37.6	5.3	3.6	0.5
85.2	29.1	34.2	250	49	42	5	1	0
78.6	26.7	34	288	60.1	31	8.4	0.2	0.3
73.3	25.7	35	442	61.6	26.4	11.2	0.4	0.4
79.4	27.7	34.9	257	72	19	9	0	0
78.7	25.9	32.9	211	26.8	63.3	7.1	2.6	0.2
73	23	31.5	269	52	37	9	2	0
76.8	26.8	34.9	207	62	35	3	0	0
79.2	26.7	33.7	231	68.7	22	8.9	0.3	0.1
86.3	28.4	32.9	305	78.9	14.7	4.3	2	0.1
81.8	27.8	34	280	55	27	10	4	0

88.4	29.5	33.3	211	66.5	26.2	5.9	1	0.4
82.6	28.2	34.1	86	49.7	41.7	7.9	0	0.7
79.2	26.7	33.8	251	69.4	21.2	5.8	3.4	0.2
82.8	27.9	33.7	156	47	40	8	5	0
79.1	26.8	33.9	130	44	45	10	1	0
80.4	26.9	33.4	153	77	15.2	7.7	0.1	0
80.1	26.8	32.5	487	70.8	24.6	3.8	0.6	0.2
78.2	27.9	35.7	161	53	31	15	0	0
82.5	28.9	35	169	33.6	52.2	10.3	3.7	0.2
79.9	26.9	33.7	448	46.5	41.5	9.3	2.6	0.1
83.7	28.1	33.5	349	50.8	45.6	3.3	0.1	0.2
78.3	26.3	33.6	242	43	51	6	0	0
80.5	26.3	32.6	226	42.9	42.5	11.7	1.1	1.8
77.8	25.4	32.6	240	48	39	11	1	1
75.2	25.7	34.2	259	69.9	22.1	7.6	0.1	0.3
80.1	27.4	34.3	295	52	36	12	0	0
82.4	28.4	34.5	244	60	27.7	11.7	0.2	0.4
78.7	26.5	33.7	232	42.8	49.4	7.4	0	0.4
83.6	27.9	33.3	131	65	27.4	7.2	0.2	0.2
77.2	27.4	35.5	182	69	16	13	0	0
74.9	25.3	33.8	311	79.1	14.3	6.5	0	0.1
81.9	27.8	33.9	216	43	45	9	0	0
81.8	27.1	33.2	279	37.3	52.2	9.4	0.6	0.5
82.1	28.8	35.1	188	63	32.5	3.5	0	0
76.5	25.1	32.8	229	53.1	29.2	12.8	4.4	0.5
82.5	29.7	36	224	25.9	62.9	8.6	2.4	0.2
87.8	28.5	32.4	302	48.8	44.2	5.5	1.4	0.1
81.9	26.9	32.8	526	59.7	25.9	9.6	4.4	0.4
79.2	26.3	33.2	162	69.7	17.5	12.4	0.2	0.2
74.6	24.9	33.4	303	69.2	22.1	7.7	0.8	0.2
77.8	25.9	33.3	203	64	30	6	0	0
80.1	26.5	33.1	125	30	54	15	0	0
82.4	28.4	34.5	190	63.7	21.9	14.3	0	0.1
81.8	28.4	34.7	222	72.6	20.3	6.5	0.3	0.3
80.6	27.9	34.6	243	58	28	12	1	0
78.2	26.4	33.7	313	57.5	27.2	12.1	2.8	0.4
83.5	28.5	34.2	149	75.9	16.6	7.2	0	0.3
77.1	26.7	34.6	296	60	33	6	0	1
78	26.7	34.3	264	61.4	36.9	0.5	0.6	0.6
88.7	30.1	34	264	54.1	34.4	9.5	1.8	0.2
80.9	27.1	33.5	148	66	21	13	0	0
83.6	28.4	33.9	290	89.5	7.7	2.7	0.1	0
72.1	24.2	33.6	407	58	34	8	0	0
78.1	26.8	34.3	195	57	31	10	1	0
78.9	25.8	32.7	337	63.8	25.3	10.1	0.5	0.3
85.6	28.6	33.4	361	59	31	10	0	0

76.1	26	34.1	214	57	24	15	1	0
79.1	26	32.9	180	40	47	10	0	0
78.2	27.5	35.1	238	78.6	15	6.2	0.1	0.1
81.2	28	34.5	220	84.2	7.3	8	0.3	0.2
87.9	30.6	34.8	367	46.9	42	8.3	2.6	0.2
78	26.7	34.2	249	64.9	27.7	5	2.2	0.2
73.3	23.9	32.5	365	34	56	6	0	0
79.1	26.4	33.4	341	53	32	11	3	0
80.3	27	33.6	163	46	47	6	1	0
79.6	26.5	33.3	105	41	46	13	0	0
80	26.5	33.1	238	48	43	9	0	0
83.1	27.7	33.3	285	67.4	18.7	12.2	1.2	0.5
75.1	25.5	34	224	56.7	36.5	6.2	0.3	0.3
77.2	25.5	33	139	39.3	46.7	12.8	0.6	0.6
72	23	32	232	68.8	25	5.7	0.4	0.1
82.2	27.7	33.7	165	62.5	27.8	9.5	0	0.2
75.3	25.8	34.2	546	69	16	12	0	0
77.7	26.8	34.5	293	77.1	15	7.7	0.1	0.1
73	23.1	31.7	488	54	40	5	1	0
67	22.1	33	545	63	22	11.5	0	0
81	27.2	33.5	286	45	35	13	6	1
73.5	24.6	33.4	314	48	43	7	1	0
79.5	26.9	33.8	322	37	51	9	3	0
84.5	28.5	33.7	241	73	14	7	0	1
78.1	26.1	33.4	230	86.9	4.6	7.7	0.4	0.4
83.4	28.1	33.7	233	24.4	59.5	14.6	1	0.5
74.7	24.7	33.1	274	85.9	7.3	6.7	0	0.1
78.3	26.7	34	125	52.2	39.6	8	0	0.2
79.2	26.9	34	258	70.5	21.2	7.6	0.2	0.5
76.8	25.6	33.3	170	65	19	11	0	0
75.9	25.6	33.7	180	22.1	66.7	10	0	1.2
78.6	25.6	32.6	173	31.9	56.8	9.3	1.7	0.3
79.5	26.9	33.8	314	70.4	19.9	9.1	0.3	0.3
77.2	25.9	33.5	243	61.7	23.9	14.2	0	0.2
78.7	26.6	33.9	177	46.4	43.5	9.3	0.5	0.3
84.7	29.4	34.8	364	65	27	6	0	0
82.2	27.3	33.2	143	48	45	7	0	0
79	27.6	34.9	182	23	52	11	0	1
76.9	26.8	34.8	156	12	74	12	0	0
74.2	25.6	34.4	293	43.7	43	10	2.8	0.5
79.4	27.1	34.1	292	61	28	10	1	0
74.2	25.2	34	198	67.1	27.5	5	0.2	0.2
79.6	26.5	33.2	406	60	28	7	4	0
81.4	28.2	34.6	201	44	53	2	1	0
86	29.2	34	342	57.4	33	8.6	0.8	0.2
77.2	26.7	34.5	374	53	35.1	8.1	3.6	0.2

81.8	27.8	34	79	62.2	25.2	11.3	1.1	0.2
84.1	28.3	33.7	87	4	85	11	0	0
71.2	22.5	31.6	251	67	25	8	0	0
79.5	27.8	35	223	60.2	26.8	12.2	0.6	0.2
80.6	28.3	35.1	139	50	31	17	2	0
82.8	27.8	33.5	157	29	60	10.2	0.4	0.4
80.3	26.8	33.3	349	72	17	6	2	0
77	27.2	35.3	375	33.6	55.7	8.3	1.9	0.5
70.1	22.4	32	366	70	21.1	8.6	0.2	0.1
78.9	25.8	32.7	268	64.5	22.6	7.7	5	0.2
90.3	30.7	34	362	42	34	19	4	0
85.4	29.2	34.2	218	52	27	17	3	0
81.3	28.4	34.9	327	3	83	8	2	1
81.6	27.5	33.7	151	8	83	7	0	0
74.4	24.3	32.6	306	36	53	10	1	0
98.3	34.8	35.4	287	39.3	43.2	11.3	5.6	0.6
81.9	28.5	34.8	238	58	27.4	14	0.3	0.3
83.3	27	32.4	206	46	35	19	0	0
96.4	33.3	34.5	257	52	31	16	1	0
81.8	28.1	34.4	207	78.6	15.3	5.6	0.4	0.1
83.3	28.9	34.7	279	77.9	16.1	5.4	0.4	0.2
83.4	27.6	33.1	247	67.2	27.5	4.8	0.1	0.4
79.5	26.9	33.9	368	69.5	21.5	8.6	0.2	0.2
77.2	27.3	35.4	185	45	36	17	2	0
78.6	26.9	34.2	161	70.2	20.4	8.8	0.3	0.3
76.3	25.6	33.5	278	59.6	31.6	8	0.6	0.2
95.7	34.8	36.4	156	30	55	8	6	1
74.1	24.4	33	555	48	29	22	0	0
73.5	25.5	34.7	161	64	25	8	0	0
72.7	25.9	35.7	201	23	58	15	1	0
83.5	28.8	34.5	214	82.5	12.4	5	0	0.1
83.2	27.7	33.3	196	49.9	40	9.5	0.3	0.3
80	25.8	32.3	143	50.7	38.4	10.2	0.2	0.5
100.6	35.2	35	465	84	7.5	5	0	0
77.5	26.4	34	300	38	48	14	0	0
76.9	26.8	34.8	272	53.8	34.7	11.1	0.1	0.3
71.6	24.6	34.3	399	57.3	34.2	7.8	0.5	0.2
78.5	26.4	33.6	275	27.9	60.3	8.8	2.6	0.4
76.9	26.8	34.9	173	60.5	33.9	5.1	0.3	0.2
77.8	25.2	32.4	110	61	26	7	0	1
65.4	19.8	30.2	166	33	51	13	0	0
78.4	26	33.1	208	20.2	70	6.4	3.2	0.2
80.6	26.6	33	192	27.1	67.7	4.8	0.1	0.3
71.9	22.7	31.6	481	24.3	57.1	15	3.3	0.3
81.7	27.1	33.1	248	65.5	19.7	12	2.5	0.3
77.4	26.4	34.2	151	53.7	40.3	5.4	0.3	0.3

76.6	26	34	169	59.2	34.7	5.9	0	0.2
81.4	26.5	32.5	187	47.2	45.9	6.5	0.2	0.2
81.6	26.6	32.6	211	57.9	35.4	6.5	0.1	0.1
80.2	27.8	34.7	261	50	38.4	10.6	0.3	0.7
77.9	28.2	36.2	274	65.1	28.7	5.5	0.5	0.2
95.9	33.5	35	467	52	35	11	2	0
79.5	27.4	34.5	425	74	15.4	9.7	0.7	0.2
80.7	27.6	34.2	267	46.1	41	11.6	1	0.3
81.6	27	33.1	194	57.1	33.8	8.5	0.3	0.3
78.9	26.5	33.6	235	66	23	11	0	0
91	31	34	446	52.7	33.6	10.4	2.7	0.6
77.7	24.8	32	205	57.7	34.7	6.8	0.6	0.2
100.8	33.7	33.5	334	45.2	48.9	5.2	0.2	0.5
100.4	34.3	34.2	467	47.7	40.8	9	2.2	0.3
85.4	27.8	32.6	158	14	79	4	1	0
75.1	23.4	31.2	462	47	25	17	0	0
78.8	27	34.2	162	68	24	7	1	0
79.9	26.6	33.3	428	53	38.3	7.5	1.1	0.1
72.2	25	34.6	154	76.2	13.3	10.1	0.2	0.2
81.4	27.1	33.3	198	45.3	43.6	9.6	0.8	0.7
77.4	25.8	33.3	168	43.3	46.4	9	1.1	0.2
76.3	24.8	32.5	239	22	68	10	0	0
83.2	28.6	34.4	136	54	27	16	0	1
72.7	24.2	33.2	164	35	50	11	1	0
82.7	26.6	32.2	197	48.5	38.9	9.7	2.3	0.6
82.3	27.8	33.8	185	38.9	44.3	12.6	4	0.2
80.6	26.7	33.1	270	30	52	16	2	0
73	24.4	33.4	225	56.2	32	10.4	1	0.4
76.8	26	33.8	188	13	86	1	0	0
71.5	24	33.6	502	49	47	3	1	0
80	26.9	33.6	236	28	58	9	1	1
75.7	25.7	33.9	283	46	36	11	0	0
78.4	26.1	33.2	415	42.4	44	11.5	1.7	0.4
81.8	27.3	33.3	284	21.7	61.7	13	3.2	0.4
84.2	28.5	33.9	307	62.6	27.1	8.1	2	0.2
78.9	26.6	33.7	322	37.1	53.3	9.2	0.3	0.1
80.8	28.7	35.5	236	77.6	16	6	0.2	0.2
79.6	27.2	34.2	233	19	75	4	2	0
81.8	26.6	32.5	173	48	40.1	11.4	0	0.5
79.9	26.7	33.4	375	41.4	41.5	11.6	5.3	0.2
84.6	27.4	32.4	141	25	68	3	0	0
73.2	24.5	33.4	434	68	22	10	0	0
71.2	23.7	33.2	173	66.5	25	6	0	0
80.8	26.8	33.1	357	79.4	14	6	0.4	0.2
81.3	27.6	34	216	70	20	10	0	0
80	26.2	32.7	160	77	16	7	0	0

75.7	25.4	33.6	243	20	73	4	2	0
79.4	27	34	169	13	76	11	0	0
87.5	29	33.1	239	63.2	29.8	6.5	0.1	0.4
79.1	27.2	34.4	286	64.2	27.2	6.7	1.7	0.2
77.7	25.3	32.5	355	65	23	9	1	0
81.3	27.9	34.3	316	63	29.2	7.1	0.3	0.4
89	28.5	32.1	375	52	27	19	2	0
78.6	27.1	34.5	307	69	22	8	1	0
79.4	26.9	33.9	305	53.5	31.7	13.9	0.6	0.3
79.7	26.6	33.3	208	20	75	5	0	0
81.1	27.1	33.4	198	45	54	1	0	0
86.9	29.1	33.4	475	29	43	24	3	1
76.1	25.4	33.3	225	73	23.9	2.7	0.2	0.2
79	27.4	34.7	205	52.3	40.5	7	0	0.2
93.6	32.1	34.3	602	44	40	11	4	0
82.1	28.4	34.6	486	39.9	41.2	13.2	5.3	0.4
79	26.3	33.3	129	47	37	15	0	1
77	25.6	33.2	328	7	51	35	0	0
80.3	28.4	35.3	152	72.5	18	9.1	0.3	0.1
78.4	26.6	33.9	149	63.5	26.7	9.2	0.4	0.2
90.3	30.5	33.7	349	41	44	15	0	0
79.3	26.4	33.3	311	66	29.7	3	0.4	0.9
78.1	25.2	32.2	131	58.9	31.5	9.5	0	0.1
74.8	25.5	34.1	403	50.9	36.1	10.9	1.9	0.2
77.3	26.4	34.2	257	27	62	7	2	0
68.1	22	32.3	321	2	55	34	4	0
80.1	26.9	33.5	303	70.7	19.7	6.2	3.2	0.2
88.2	29.9	33.9	405	26.5	62.7	7	3.4	0.4
91.7	32.9	35.9	206	33.5	55.5	11	0	0
79.8	27	33.8	268	55.4	32.7	11.3	0.1	0.5
73.7	24.4	33	198	25	68	5	1	0
73.8	24.4	33.1	442	41.9	47.9	8.9	1.1	0.2
80.6	26.5	32.8	490	56.8	32.5	8.7	1.8	0.2
73.4	24.7	33.6	283	31.3	51.5	11.5	5.1	0.6
104.4	37.3	35.8	316	42	49	7	1	1
79.2	26.5	33.4	375	53.4	37.3	8.6	0.6	0.1
71.6	23.1	32.3	236	42	45	13	0	0
80.5	28.2	35	388	75.5	15	9.5	0	0
79.7	26.7	33.5	216	66	21	11	0	0
74.1	23.9	32.3	136	45.8	42.4	10.9	0.2	0.7
96.1	34.2	35.6	338	27	57	16	0	0
78.1	26.6	34.1	112	40.9	41.7	14.9	1.7	0.8
88	29.3	33.2	83	44	44	5	0	0
78.7	26.5	33.6	521	21	68.8	5.8	3.7	0.7
87.3	30.1	34.4	454	26	61	11	1	0
79.3	26.5	33.4	160	63	25	6	0	0

78.2	26.4	33.7	255	69.9	19.3	10.5	0.1	0.2
85.1	28.1	33.1	264	68.7	28	3.1	0.1	0.1
78.5	26	33.2	526	39	43	11	3	0
78.3	26.2	33.4	369	43.5	43.5	10.5	2	0
80.1	27.3	34.1	208	65.1	26.3	8.2	0.2	0.2
81.4	27.1	33.3	119	32	55	12	0	0
87.2	31.1	35.7	463	57.1	32.8	7.4	2.4	0.3
75.8	25.7	33.9	317	67.3	27.8	3.9	0.7	0.3
91.2	31.5	34.6	200	10	81	8	0	1
79.4	26.8	33.7	407	43.3	43.5	11.7	1	0.5
77.6	25.9	33.4	305	64	31.5	4	0	0
92.4	31.1	33.7	349	21.2	66.2	8.7	3.2	0.7
82.9	28.3	34.2	238	35.1	57	7	0	0.9
90.8	31.3	34.4	361	27.2	60.4	8.4	3.4	0.6
73.8	25.7	34.9	239	34.7	50.7	13	1.4	0.2
98	33.5	34.2	472	54	29	15	0	1
78.3	26.7	34.1	269	61.3	29.6	6.7	2.2	0.2
76.1	25	32.9	319	37	49	8	2	1
76	26.2	34.5	145	28.5	62.4	7.6	1.1	0.4
80.5	26.7	33.1	263	52.8	31.7	9.8	5.5	0.2
80.5	26.9	33.4	108	43.2	48.1	6.3	2.4	0
76.7	25.5	33.2	295	54.6	40.6	3.8	0.9	0.1
77.5	26.9	34.7	118	34.7	48.4	14.5	1.1	1.3
78.7	27	34.3	284	61.8	32.2	5.3	0.5	0.2
75.9	25	32.9	240	33	62	5	0	0
78.7	25.5	32.4	263	58.6	34.6	5.8	0.4	0.6
70.1	22.8	32.6	331	59.7	34.8	4.8	0.5	0.2
77.9	26.1	33.5	226	28	71	1	0	0
80.8	27.5	34.1	224	37.1	51.7	8.2	2.7	0.3
74.8	26.9	36	279	55	30	7	0	0
79.2	27.3	34.5	297	49.1	43	6.5	1.2	0.2
76.6	25.3	33	358	31.2	58.8	9.2	0.6	0.2
82.6	28.9	34.9	520	63	26	11	0	0
75.6	25.6	33.9	201	50	37	12	1	0
77.8	25.8	33.2	119	8	83	8	0	0
81.5	27.4	33.7	220	52	40.6	5.1	2.1	0.2
98.7	31.7	32.1	677	40	51	8	0	0
84.8	29.2	34.5	362	54.8	35.1	8.9	0.9	0.3
84.2	29	34.5	285	59	31.7	8.9	0.2	0.2
79.7	26.1	32.7	187	66	25	9	0	0
77.7	25.6	33	263	63.6	22.3	10.2	3.8	0.1
79.2	26.3	33.2	199	40	44	15	0	1
80	25.3	31.7	306	38.1	51.5	2.2	0	0
77.4	27	34.9	384	42.6	48	5.8	3.4	0.2
74.7	24.3	32.6	305	49.7	40.8	8.9	0.5	0.1
78.8	27.1	34.4	345	77.3	15	7.4	0.1	0.2

73.2	23.8	32.6	709	20	73	7	0	0
75.1	24.7	32.9	241	76	18	5	0	0
79.2	26.8	33.9	162	19	54	24	0	0
80.8	26.8	33.2	295	48.9	39.5	9.7	1.8	0.1
80.6	27.5	34.1	371	44.2	43	11.8	0.8	0.2
82.6	27.5	33.3	272	48.6	39.3	10.8	1	0.3
79.9	26.5	33.2	329	58.2	33.7	5.4	2.5	0.2
76.5	26	34	315	64.3	32	3	0.4	0.3
83.6	27.9	33.3	239	39	36	23	2	0
78.7	26.2	33.3	417	61.2	25.6	11.9	0.9	0.4
67.3	21.8	32.4	312	67.4	27.3	4.9	0.3	0.1
80	26.4	33	276	35	47	16	1	1
75.6	24.8	32.9	357	48.8	45.8	3.8	1.3	0.3
93.4	30.6	32.8	306	28.6	54.1	13.9	2.6	0.8
75.6	25.7	34	523	71	15	9	0	0
79.7	27.8	34.9	300	49.6	36.7	11.5	2	0.2
74.3	25.1	33.8	113	27	39	21	4	1
72.8	24.8	34.1	342	74.4	22.5	2.8	0.1	0.2
91.1	30.4	33.4	402	15.6	68.6	12.5	2.6	0.7
74.1	24.5	33.1	128	68	29	2	0	0
77.9	26.7	34.2	350	38.1	51.5	9.8	0.4	0.2
81	27.4	33.8	204	75.1	17.1	4.8	2.9	0.1
77.5	26	33.6	153	26.7	61.7	9.2	1.7	0.7
95.6	32.5	34.1	370	21.6	69.8	6.3	2.1	0.2
73.8	25.5	34.5	230	72.8	10.5	14.2	2.3	0.2
81	27.4	33.9	300	31	56	9	3	1
82.8	28.2	34.1	307	48	38	10	1	1
102.2	35.6	34.9	184	39	45	14	1	1
83	27.4	33	226	22	66	10	2	0
74	24.6	33.2	141	30.7	54.1	14.2	0.4	0.6
80.4	27.5	34.2	260	67.8	21.2	10.7	0.2	0.1
86.8	28.4	32.7	261	59.5	28.8	10	1.5	0.2
77.2	24.9	32.3	106	67.3	25.4	6.7	0.3	0.3
82.1	29	35.3	258	56.1	29.1	11.9	1.7	1.2
80.3	26.4	32.9	204	57.6	36.3	5.6	0.1	0.4
91.8	32	34.9	417	47.5	40.2	10.1	1.7	0.5
79.7	27	33.9	329	49.1	38.1	10.2	1.4	1.2
86.2	28.5	33	394	21	69	7	1	0
74.5	24.8	33.3	497	17	40	36	3	0
113.3	37.7	33.2	206	24.4	62.1	10.6	2.2	0.7
76.6	25.4	33.2	163	31.1	60.2	7.7	0.8	0.2
88.5	30.1	34	304	50	45.4	3.7	0.7	0.2
75.9	26.2	34.4	248	62.6	31	5	1.2	0.2
103.3	35.3	34.2	339	25	55	12	5	1
84.9	28.4	33.4	265	8	87	5	0	0
83.6	28.1	33.6	231	83.2	14.6	1.4	0.5	0.3

72.3	23.6	32.6	421	44	42	14	0	0
68.2	21.7	31.8	509	65.5	25	8.5	0.5	0.5
76.1	25.4	33.4	249	30.2	66	2.4	1.1	0.3
96.5	32.6	33.8	271	24.4	61	9	4.8	0.8
79.4	27	34	196	36.6	51.4	10.1	1.3	0.6
79.5	26.7	33.6	192	43.7	40.3	14.4	1.3	0.3
84.4	29.3	34.7	389	48.6	37.6	12.4	0.7	0.7
78.2	24.8	31.7	336	66	21	12	0	1
83	28.1	33.8	433	56.9	36.8	5.4	0.7	0.2
96.2	33.2	34.5	703	34.9	53.7	7.2	3.8	0.4
81.1	26.3	32.4	194	31	49	12	1	1
90.7	30.9	34	357	64.3	27.4	6.8	1.3	0.2
81.9	27.2	33.2	213	15	73	9	0	0
82	26.9	32.8	323	21	69	6	2	0
82.7	28.7	34.7	297	11	77	7	2	1
102.1	33.8	33.1	481	61.1	31.2	6.7	0.8	0.2
79.6	26.2	32.9	149	59	20	12	0	0
80.2	26.8	33.4	195	28.8	63.1	7.1	0.3	0.7
81.3	26.8	33	91	36.2	54.2	9	0.2	0.4
80.7	26.5	32.8	314	72	20	5	0	0
81.3	27.1	33.3	214	24.4	65.7	8.8	0.7	0.4
72.9	24	33	288	37	52	7	4	0
77.5	25.6	33	262	48.4	42.8	7.8	0.6	0.4
81.6	27.2	33.3	337	57	26	17	0	0
74.2	23.7	31.9	134	31.8	61.6	5.6	0.1	0.9
77.8	25.8	33.1	182	57.2	27.1	7	8.1	0.6
78.1	26.7	34.2	328	32.7	55.3	8.7	3.1	0.2
79.4	25.9	32.6	335	29	57	9	2.5	0.5
77.7	25.8	33.2	220	59	21	15	0	0
79.7	27.2	34.1	122	22.9	67.3	8.3	0.4	1.1
79.3	25.8	32.5	261	13	55	22	0	0
82.2	26.9	32.8	197	54.5	37.4	7.5	0.4	0.2
76.4	25.1	32.8	179	62.9	26.6	10.4	0	0.1
79.7	26.9	33.8	281	46	36	16	1	0
78.5	25.5	32.5	339	53	34.9	10.2	1.4	0.5
78.7	26.2	33.3	155	63.4	25.2	11.2	0	0.2
78.3	25.5	32.6	515	73.5	16	10.5	0	0
80.9	26.8	33.2	230	65	25	10	0	0
80.9	27.5	33.9	256	43	45	7	4	0
83.1	27.5	33.1	240	20	69	7	1	1
75.8	26	34.3	366	44	48	7	0	0
92.2	31.5	34.2	252	45.1	35.9	12.4	6.1	0.5
83.7	27.6	33	255	25.6	62.5	6.3	5.2	0.4
77.3	26.5	34.3	216	71	21	7	0	0
90.8	30.5	33.5	350	12	73	6	8	1
77.6	26	33.5	244	22	69	8	0	0

77.9	25.5	32.8	425	53.2	41.9	4.4	0.3	0.2
82.4	27.5	33.3	120	53	28	16	0	0
80.3	27.1	33.7	311	43	44	12	0	0
73.5	24.3	33.1	217	59.5	25	11	1.5	0
84.6	28.6	33.9	401	9	88	1	2	0
75.7	24.7	32.6	232	42.7	48.8	7.1	1.1	0.3
78.4	26.1	33.3	541	30	60	4	6	0
86.7	29.8	34.4	287	47	31	20	1	1
77.8	26.1	33.6	203	85.8	8	6	0	0.2
82.5	27.8	33.7	305	47.7	45.2	4.3	1.9	0.9
80.3	26.9	33.4	297	77	18	5	0	0
74.5	25.1	33.7	318	85	9.5	5.3	0.1	0.1
83.3	28.8	34.5	487	58.2	31	7.3	2.1	1.4
79.7	27.7	34.8	172	52.9	38.7	8.2	0.1	0.1
85.1	28.9	34	337	66	16	17	1	0
79.9	26.7	33.4	340	81	10	8	0	0
81.7	26.8	32.8	397	61.2	21.6	14.6	1.8	0.8
79.3	26.9	33.9	273	43.8	50	5.8	0.2	0.2
80.8	29.4	36.4	276	76	15.5	7.2	1	0.3
79.3	27	34.1	216	62.8	30.5	6.5	0.1	0.1
83.3	28.7	34.5	181	94.6	4.3	1	0	0.1
76.2	25.5	33.5	226	68.5	22	8.5	0.5	0
74.8	24.7	33	416	82.1	12	3.5	2.1	0.3
73.7	25	33.9	152	54	20	25	0	0
81.8	27	33.1	485	31	56	9	1	0
84.3	29.1	34.5	324	83.7	10.6	5.4	0.1	0.2
84	27.7	33	307	72.3	23.1	3.6	0.8	0.2
77.6	26.5	34.2	358	80.8	14.2	4.6	0.3	0.1
82.1	27.4	33.4	165	75.7	17.9	6.3	0	0.1
83.8	28.5	34	156	64	19	14	0	0
86.3	29.7	34.4	246	62.2	26.9	10.2	0.4	0.3
82.4	27.5	33.3	257	86.3	6.8	6.7	0	0.2
76.3	25.9	33.9	370	78.9	13.1	7.2	0.5	0.3
80.4	27.8	34.6	154	81	16	3	0	0
78.9	28.4	36	238	86.4	8.2	4.2	1	0.2
75.6	25.9	34.2	286	77.4	13.8	8.3	0.3	0.2
84.3	28.4	33.7	268	80.6	12.4	6.5	0.3	0.2
77.1	26.4	34.2	399	66.1	25.5	7.3	0.9	0.2
84.7	29.2	34.5	254	62.6	29.4	7.9	0	0.1
79.5	27.3	34.3	275	57.9	33.2	7.5	0.7	0.7
78	27.3	35	224	76	16.5	7.4	0	0.1
79.1	26.3	33.2	263	77.5	14	8.2	0.1	0.2
77.2	26.5	34.3	257	87.1	8.4	3.8	0.5	0.2
82.7	28.2	34.1	180	28.3	53.1	17.7	0.7	0.2
81.5	27.8	34.1	182	32.8	49.1	17.5	0.3	0.3
80.5	28.2	35	233	65.3	24.8	9.2	0.1	0.6

77.9	26.9	34.5	314	72	15	13	0	0
79.5	27.8	35	85	68	12	19	0	0
79.6	26.8	33.7	279	77.8	15.9	6.2	0	0.1
81	28.1	34.7	232	83.7	10.9	5.2	0	0.2
82.8	29.1	35.2	280	44	50	6	0	0
84.7	28.1	33.2	243	77.6	15.1	7	0.2	0.1
81	27.9	34.4	192	70.4	20.5	8.8	0.1	0.2
78.7	27	34.3	340	80.1	12.1	7.6	0.1	0.1
81.3	27.2	33.4	456	72.4	18.8	8.5	0.1	0.2
77.3	27.4	35.5	231	56.6	35	7.9	0.3	0.2
84.5	29.3	34.7	392	76.1	18.3	5.4	0.1	0.1
77	26.7	34.7	439	69	21.1	8.4	1.4	0.1
81	28.2	34.8	277	67.1	22.7	10	0	0.2
73.2	25.2	34.5	197	74	6	6	0	0
79.6	26.6	33.4	270	66.9	23.5	8.5	0.9	0.2
81.4	27.8	34.2	393	65.8	21.8	12	0.2	0.2
77.8	26.8	34.5	358	70.2	26	2.8	0.7	0.3
86.2	30.3	35.1	90	50.9	42.4	6.3	0.2	0.2
78.7	27.1	34.4	397	80	16	3	1	0
75.7	25.5	33.6	299	79.8	14	6	0.1	0.1
81.4	27.5	33.9	256	54.1	34.8	9.6	1.2	0.3
73.4	25.8	35.1	383	49.3	42.4	5.5	2.4	0.4
79.9	27.3	34.1	378	62.2	24.6	9.2	3.6	0.4
81.8	28.1	34.3	304	83.3	12.1	3.2	1.3	0.1
82.9	28.5	34.4	230	64.9	27.3	7.7	0	0.1
79.9	27.5	34.5	165	56	29	14	0	1
82.4	27.7	33.6	229	83	10	7	0	0
81.7	28.1	34.5	387	71.5	19.5	8	1	0
87.4	29.7	34	138	54.7	32.8	11.7	0.2	0.6
84.2	28.8	34.2	297	48	40	10	1	1
77.9	27.9	35.8	346	45.4	47.6	6.3	0.3	0.4
81.4	28	34.3	249	78.1	12.7	8.9	0	0.3
81.3	28	34.4	226	70	20.8	8.9	0.1	0.2
82.6	27.1	32.8	202	58.9	29.2	11.1	0.2	0.6
78.5	27.4	34.9	351	75	20	5	0	0
79.6	26.9	33.8	163	63.6	27.8	8.1	0.4	0.1
78	26.8	34.4	257	59.7	36.2	3.4	0.3	0.4
80.8	26.7	33.1	432	69	21	10	0	0
76.3	27.3	35.8	342	68.4	20.1	3.2	8.2	0.1
78.8	25.6	32.5	361	59.4	34.2	5.1	0.9	0.4
82.2	27.7	33.7	184	69	18	6	2	0
79.3	27.5	34.6	199	79.4	13.1	7.3	0.1	0.1
80.3	27.6	34.4	296	69	14.5	7	0	0
79.7	27.8	34.9	215	78	11	3	0	0
79.9	27	33.8	303	75.2	15.4	8.5	0.7	0.2
80.7	26.5	32.8	273	85	6	8	0	1

83.8	27.9	33.3	112	68.9	22.4	8.6	0	0.1
81.3	27.2	33.4	404	84	9	7	0	0
81.5	26.9	33.1	227	76.8	16	7	0	0.2
82.5	28.5	34.6	250	86.4	8.7	4.7	0	0.2
79.9	27.5	34.5	159	65.9	18.9	12.2	2.3	0.7
79.5	26.2	33	220	77.6	13.5	8.4	0.3	0.2
77.9	26.5	34	318	31.7	56.5	9.5	1.9	0.4
82	26.9	32.7	419	37.1	54.9	5.9	1.7	0.4
77.8	26.2	33.7	275	86.7	10.9	2.2	0	0.2
83	28.9	34.9	254	88.4	6.2	4.9	0.4	0.1
83	27.7	33.4	241	79.6	14	5.9	0.3	0.2
78.7	26	33	229	82.5	11.6	5	0.8	0.1
83.5	28.5	34.1	161	71.2	20.8	6.8	0.6	0.6
79.4	26.9	33.8	161	62.3	25.1	12.2	0.1	0.3
81.9	27.5	33.6	212	68.2	17.3	14	0.3	0.2
77.8	26.7	34.2	130	36.1	54.7	8.8	0	0.4
77.9	27.1	34.8	241	78.4	12.2	8.7	0.5	0.2
78.1	27.6	35.3	180	60.3	21.3	16.1	2	0.3
84.2	27.7	32.9	234	56.4	33	9.3	0.8	0.5
82.2	28.3	34.4	208	85.6	9.2	4.9	0.1	0.2
80	26.8	33.5	356	59	27	7	3	0
84.3	28.6	33.9	144	85.3	8.9	5.3	0.3	0.2
79	26.6	33.7	287	82	11	7	0	0
82.3	28.5	34.6	165	79.4	9.7	10.2	0.5	0.2
81.4	27.3	33.5	247	73	19	8	0	0
77.1	25.6	33.2	244	65	26	7.6	0.4	1
76	26.3	34.7	350	86.6	9.3	4	0	0.1
75.7	27.4	36.2	344	74	13	13	0	0
82.3	27.6	33.6	207	72	24	4	0	0
87.8	29.4	33.5	157	51	36	11	2	0
81	27.9	34.4	311	79.7	12.9	5	2.2	0.2
81	27.1	33.5	470	59.6	32.2	7.8	0.2	0.2
77.7	25.8	33.2	230	49.7	35.7	8.9	5.1	0.6
82.6	27.1	32.8	158	75.6	15	8.8	0.1	0.5
74.3	25.2	33.9	306	70.5	14	11	0	0
81.3	27	33.2	259	47	36	14	2	0
80.9	27.2	33.6	264	57.5	28.7	11.3	2.1	0.4
68.3	21	30.7	300	48.8	34.1	13.8	2.9	0.4
79.9	27.1	34	143	82	12	6	0	0
77.1	26	33.7	378	72.3	18.4	9.1	0	0.2
83.9	28.8	34.3	177	70	20	10	0	0
82.2	27	32.9	206	61.2	31.1	7.5	0	0.2
84.3	27.9	33.1	230	72	20.7	6.5	0.4	0.4
82.1	28.3	34.5	439	64.5	27	8	0	0
77.4	24.7	31.9	126	25	60	11	1	3
76.8	25.8	33.6	305	59.3	30.6	4.5	5.4	0.2

82.9	28	33.8	300	47.8	43.2	4.5	3.8	0.7
81.7	27.4	33.6	240	83	9	7	0	0
79.6	26.8	33.6	300	75.4	14.2	9.8	0.5	0.1
65.4	20.5	31.4	269	54.1	40.7	4.5	0.2	0.5
80.7	27.2	33.7	182	63.7	27	6.3	2.8	0.2
82.1	28	34.1	270	77.1	16.8	5.6	0.1	0.4
85.2	28.6	33.6	177	69.8	21.5	8.5	0	0.2
75.8	26.2	34.6	260	61.2	29.4	7.2	1.7	0.5
78.7	26.5	33.7	205	36.6	55.3	7.5	0.2	0.4
80.2	26.6	33.2	146	77.7	14.7	7.5	0	0.1
91.2	30.9	33.9	65	14	71	15	0	0
80.1	26.4	33	217	71	19	10	0	0
84.4	28.3	33.5	234	69.7	25.2	4.7	0.2	0.2
80.2	28	34.9	183	76.3	16.9	6.6	0.1	0.1
80.1	28.4	35.4	195	84.1	8.2	7.4	0.1	0.2
80.9	26.6	32.9	180	74.5	18.4	6.6	0.2	0.3
79.4	28.2	35.5	476	84	6	9	0	0
73	24.3	33.3	210	40.1	53.7	3.9	1.3	1
77.3	25.9	33.5	236	79.9	12.4	7.4	0.1	0.2
80.3	27.7	34.5	305	56.8	38	4.2	0.7	0.3
74.8	27.4	36.7	289	60	37	3	0	0
80.3	28.5	35.4	173	66.4	29.1	4.3	0	0.2
87.9	30.2	34.4	222	28.5	50.5	14.5	6.1	0.4
77.1	25.5	33.1	258	71.9	19.6	8	0.2	0.3
74.3	26.1	35.1	284	46.1	46.7	6.5	0.2	0.5
82.2	28.9	35.2	204	79.2	9.8	10.8	0	0.2
80.5	27.2	33.7	194	75.1	17.8	7	0	0.1
82.5	28.6	34.7	173	72.7	19.4	6.9	0.6	0.4
77.6	27.5	35.4	270	86.5	9	4.5	0	0
73.9	23.7	32.1	268	69	24	6	1	0
80.1	27	33.8	309	50	33	15	2	0
79.7	27.1	34	264	58	32.7	7.2	1.5	0.6
80	27.8	34.7	176	47.4	31.6	20.4	0.4	0.2
76.4	26.7	34.9	299	52.2	34.8	5.7	6.8	0.5
82.1	28.7	35	253	87.3	7.8	4.8	0	0.1
81.2	27.1	33.4	193	60	19	7	0	0
83	28.3	34	198	65.7	27.9	6.2	0	0.2
78.8	27.3	34.7	413	61.1	33.1	4.3	1.3	0.2
77.3	27.3	35.3	227	56.1	35.4	8.2	0.2	0.1
80.6	27.7	34.4	276	70	24	6	0	0
75.2	25	33.2	192	62.8	32.4	4	0.5	0.3
77.7	26.7	34.3	226	61.6	24.8	12.8	0.7	0.1
79.8	26.9	33.7	156	35.8	55.5	7.2	0.5	1
81.7	27.5	33.7	249	60.7	28.5	8.6	2	0.2
81.7	26.2	32	480	82	10	7	0	0
75.8	26.7	35.2	274	86	10	4	0	0

81.6	27.7	33.9	184	74.2	15.6	9.9	0.2	0.1
80.4	27.1	33.7	179	73.8	16.9	9	0.2	0.1
85.7	29.2	34.1	310	27.1	64.6	4.4	3.5	0.4
80.8	26.9	33.3	156	67.1	23.4	7.1	2	0.4
81.9	27.9	34.1	350	62.7	27.7	6.4	3	0.2
76	25.6	33.6	294	67	15	15	0	0
84.5	28.4	33.6	308	85.5	9.4	4	0.9	0.2
82.1	27.3	33.2	202	62	34	4	0	0
82.1	27.4	33.3	519	66.7	20.7	12.2	0.1	0.3
75.1	25.4	33.8	161	68	22	10	0	0
83.3	28.5	34.2	211	68.5	21.6	8.6	1.1	0.2
83	28.3	34.1	289	60.6	23.6	15.2	0.1	0.5
82.8	28.1	34	186	52.3	38.4	8.6	0.2	0.5
80.1	26.3	32.9	168	39	55	6	0	0
81.8	29.2	35.7	304	68.2	21.3	9	1.3	0.2
73.9	25.1	34	263	53	37	9	0	0
78.8	28.3	35.9	167	73	7	11	0	0
78.7	28.2	35.8	232	47	31	18	0	0
81.6	27.8	34	276	80.5	11.9	6.6	0.8	0.2
78	26.9	34.5	199	56	31	12.1	0.5	0.4
81.2	27.8	34.3	165	52	40	8	0	0
83.9	28.2	33.6	305	59.1	29.9	10.6	0.2	0.2
78.6	28.7	36.5	357	61	16	10	0	0
80.5	27.3	33.9	353	62.8	29.4	7.1	0.5	0.2
77	27.5	35.7	221	62	29.2	8.3	0.3	0.2
79.7	27.3	34.3	122	63.5	23.2	12.8	0.2	0.3
79.7	26.1	32.7	256	80.2	12.2	7.5	0	0.1
77.5	25.4	32.8	243	46.9	37.9	14.8	0	0.4
78.5	26.6	33.8	268	71	23	5.5	0.5	0
84.7	28.6	33.8	344	59.6	28.4	11.9	0	0.1
74.8	25.4	33.9	297	57.2	33.5	7.2	1	1.1
83.9	28.1	33.5	313	85	8	6	0	0
79	26.7	33.8	354	60	29	10	0	0
86.5	25.6	29.6	125	51.1	40	5.7	3	0.2
75.8	25.9	34.2	226	54	40.7	4.7	0.3	0.3
78.7	27.2	34.5	467	55.6	34.1	7.8	2.2	0.3
83	28	33.8	309	81.2	13.4	5.2	0.1	0.1
79.7	27.3	34.3	146	58.9	29.8	10.7	0.3	0.3
79.9	28.1	35.1	274	74.2	16.8	8.2	0.7	0.1
79.1	28	35.4	232	43	28	26	1	0
80.1	26.8	33.4	205	58.2	30.1	10.9	0.6	0.2
79.5	26.7	33.5	227	78	15	6	1	0
81.5	28.2	34.6	156	64	23	11	2	0
77.3	26.1	33.8	153	59.2	26.7	14	0	0.1
79.1	27.2	34.4	183	28.1	61.4	9.6	0.2	0.7
84.4	28.9	34.3	196	56.7	35.5	6.2	1.3	0.3

82.2	28.2	34.3	267	64.3	26.9	7.8	0.8	0.2
82.8	27.2	32.9	363	57.3	32.5	9.4	0.4	0.4
87	29.1	33.4	280	77	12	9	0	0
81.2	27.7	34.2	173	51.4	29.4	12.5	6	0.7
78.4	26.6	33.9	202	59.2	31.8	8.6	0.2	0.2
79	26.5	33.6	214	72.6	17.2	9.8	0.2	0.2
79.8	28.8	36.1	324	80.8	15.9	3	0.2	0.1
78.8	26.4	33.4	116	40	39	18	0	0
78.4	25.7	32.8	198	19.6	65.2	14.5	0.2	0.5
85.8	30.2	35.2	261	85	10	5	0	0
77.7	26.9	34.6	284	71.8	22.3	5.7	0	0.2
77.9	27.3	35.1	167	74.8	13.5	11.5	0.1	0.1
83.5	28.1	33.7	322	79	16	4	0	0
77.8	25.7	33	20	5	23	0	0	0
79.5	26.7	33.6	174	51.6	34.7	11.8	0.3	1.6
83.2	28.2	33.9	358	66.4	23.1	9.7	0.6	0.2
76.1	25.5	33.5	274	69.8	21.3	8.1	0.5	0.3
84.2	27.4	32.6	326	81.3	12.1	5.1	1.2	0.3
80.8	28.8	35.7	346	63	29	8	0	0
86.3	29.8	34.5	256	71.1	24.4	3.9	0.4	0.2
78	26.7	34.3	516	77	15	8	0	0
80.1	27.1	33.8	153	79	11	8	0	0
82.4	27.8	33.7	347	78	11	11	0	0
79.4	27.5	34.6	238	54	31	14	0	0
74.3	26.5	35.7	168	23	68.2	7.4	0.8	0.6
85.1	28.4	33.3	369	81	10	6	0	1
74.9	25.7	34.3	223	74.9	17.3	7.6	0	0.2
78.1	27.1	34.8	195	58	33	8.6	0.4	0
81	27.2	33.6	249	49.4	40.7	9.3	0.2	0.4
83.9	28	33.3	141	50	33	16	1	0
74.7	24.8	33.2	435	82	15	3	0	0
75.3	25.8	34.3	223	57	30	10	0	0
78.9	26.9	34.1	205	28	50	18	0	0
81	27.4	33.8	176	58	29	13	0	0
76	26.1	34.4	167	29.4	63.2	6.6	0.3	0.5
71.8	23.3	32.5	148	49	43	8	0	0
84.1	27.6	32.8	360	67.5	22.5	6	0.5	0
79	27.1	34.3	105	39	49	9	1	2
79.1	27.7	35	205	78.4	13.8	7.5	0	0.3
83	27.4	33	233	42	29	29	0	0
79.3	27.5	34.7	287	69.8	19.8	6.8	3.4	0.2
80	27.7	34.5	238	78.7	12.1	9	0	0.2
80.5	27.3	33.9	578	70	22.5	6	1.5	0
75.8	25.7	33.9	141	32.1	53.9	12.9	0.9	0.2
76.6	25.1	32.8	143	32	47	18	0	0
77.4	26.1	33.7	247	62.2	31.3	6.3	0.1	0.1

81	26.8	33.2	149	75.9	10.3	13.7	0	0.1
79.3	26.7	33.6	258	58.4	34	6.3	1.2	0.1
84.8	29.2	34.4	219	79.2	15.4	4.6	0.5	0.3
71.7	24.6	34.4	237	85	5.5	5.5	0	0
76.2	26.3	34.5	210	19.9	70.9	8.3	0	0.9
84.5	29.5	34.9	197	43.6	35.1	15.6	5.3	0.4
78.8	27.2	34.6	271	44	42	12	1	1
75.7	25.9	34.3	271	70	20	8	2	0
84	28.2	33.5	390	70.6	20.9	8.3	0	0.2
79.2	27	34.1	196	81.1	8.7	8.6	1.3	0.3
80.8	27.8	34.4	2.9	27	56	8	0	0
83.9	27.7	33	293	84.5	7.5	6.5	0	0
80.5	27.5	34.1	124	52	32	16	0	0
78.8	27.1	34.4	226	49	49	0	2	0
80	27.3	34.2	205	71.2	22	6.3	0.4	0.1
89	30.5	34.2	222	79.1	15.5	5.3	0	0.1
76.7	26.2	34.1	293	83.5	8.4	6	1.9	0.2
81	27.8	34.3	381	61	20	18	0	0
79.6	27.6	34.7	288	76	9	11	0	0
80.8	27.8	34.4	174	45.3	40.5	12.7	0.5	1
80.3	26.3	32.7	386	47.9	44.8	7	0.1	0.2
80.3	27.3	33.9	229	81.5	12.1	6.2	0.1	0.1
81.7	27.7	34	277	83	7.5	7.5	0	0
73.5	22.8	31	508	70	21	6	2	0
84.5	29.1	34.5	308	70	23	6	0	0
81.7	28.3	34.6	306	77.3	16.5	5.8	0.1	0.3
84	28.6	34	223	52	36.4	10.6	0.5	0.5
78.2	26.3	33.6	323	81.6	13.5	4.7	0.1	0.1
80.8	26.9	33.3	263	64.4	26.3	8.7	0.3	0.3
82.8	28.5	34.4	206	76.6	16.9	5.8	0.5	0.2
76.9	25.8	33.5	245	45	48	7	0	0
81.1	28.2	34.8	183	56.2	33.6	9.6	0.3	0.3
78.5	25.6	32.6	304	59	32	8	1	0
84.4	28.8	34.1	289	58.1	36.2	3.4	2.1	0.2
73.7	24.4	33.1	555	73	13	13	0	0
79.5	27.3	34.3	135	67	19	13.1	0.7	0.2
74	26	35	291	54.6	32.9	7.8	4.4	0.3
78.5	26.2	33.4	378	50.1	39.7	7.3	2.4	0.5
80.2	27.1	33.8	282	75.2	14.9	9.2	0.3	0.4
85	29.6	34.9	334	35	58	5	2	0
79	26.8	33.9	140	55.1	33.3	11	0.3	0.3
80.5	27.8	34.5	343	63.6	26.5	9.8	0	0.1
83.1	28.3	34.1	179	77	12	10	0.5	0
82.4	27.5	33.3	231	49.9	42.3	7.7	0	0.1
80.6	28.8	35.8	227	70	12	9	0	0
81.4	26.8	32.9	248	59.1	29.5	9.7	1.3	0.4

87	30	34.5	366	70.9	23.4	5.4	0.2	0.1
82.3	27.8	33.8	223	71	24	5	0	0
84.7	28.9	34.1	249	71.3	19.8	8.7	0.1	0.1
83.1	27.1	32.7	437	72	18	8	0	0
78.6	27.8	35.3	192	70.4	20.6	8.8	0.1	0.1
85.3	28.5	33.4	264	58	27	15	0	0
76.9	25.8	33.5	211	30.2	63.6	5.8	0	0.4
73.7	25.8	35	227	55	31.6	12.1	1.1	0.2
77.8	26.9	34.6	272	73.1	14.6	11.2	0.9	0.2
81.4	27.4	33.7	288	79.5	9	9	0.5	0
77.8	24.8	31.9	219	59	24	14	1	0
78.4	26.3	33.5	371	68	18	8	0	0
78.4	27.2	34.6	211	61	22.6	15	1.1	0.3
86.1	28.5	33.1	185	55.4	33.6	10.4	0.2	0.4
84.3	29	34.4	174	75.3	16.5	7.5	0.6	0.1
81.6	28.3	34.7	231	57.4	31.5	10.7	0.2	0.2
69	23.2	33.6	381	52.1	32.7	14	0.9	0.3
82.2	28.9	35.2	313	62	24.5	6.5	0	0
78.4	27.2	34.7	250	54.1	37.3	7.3	0.9	0.4
74.4	25.8	34.8	484	58	23	17	0	0
75.9	25.8	34	234	70.6	22.2	6.9	0.1	0.2
84.8	29.9	35.3	193	60.4	26.4	12	1	0.2
75.8	25.2	33.2	235	61.6	28	8.9	1.1	0.4
76.2	26.5	34.8	161	37	52	11	0	0
80.8	27.3	33.8	217	50.9	38.5	10.1	0.4	0.1
83.7	28.4	34	241	53.4	39.3	6.5	0.1	0.7
84.1	27.9	33.1	336	68.6	24.7	4.4	2.1	0.2
73.3	25.6	35	233	45.5	48.1	5.3	0.6	0.5
72.8	24.9	34.1	230	57	35	8	0	0
73.9	24.4	33	250	36.1	53.7	9	0.1	1.1
73.7	24.6	33.4	429	57	32	10	1	0
77	26.9	34.9	349	51.5	35.9	7.9	3.7	1
79.2	26.7	33.7	167	77.1	17.6	3.1	1.9	0.3
78.7	26.5	33.7	235	51.2	39.7	7.3	0.6	1.2
70.7	24.4	34.5	167	68.8	20.4	9.8	0.8	0.2
82	27.1	33.1	357	65	20	14	0	0
75.3	25.8	34.3	260	67	25	8	0	0
81.7	27.6	33.8	290	48.2	42.7	8.4	0.5	0.2
79.6	26.5	33.3	111	64.9	28	6.6	0.3	0.2
86.5	27.6	31.9	321	62	31	6	0	0
79.4	25.7	32.3	277	50.3	35.9	12.7	0.5	0.6
80.9	27.8	34.4	170	76.6	15.4	6.5	1.4	0.1
77.9	26	33.3	426	65	27	8	0	0
80.8	27.5	34	291	49.9	39.2	10.6	0	0.3
81.9	27.1	33.1	289	76.6	18	5.2	0.1	0.1
77.8	26.8	34.4	307	72.6	23.1	4.1	0	0.2

74.6	25.6	34.3	347	67	19	12.5	0	1
76.5	26	34	203	65	23	12	0	0
77.7	28.2	36.3	449	68.1	23.9	7.6	0.1	0.3
76.8	27.1	35.2	551	65.7	24.8	9.2	0.2	0.1
75.1	26.4	35.2	243	64	22.4	12.3	1	0.3
78.2	26.2	33.5	352	30.8	60.4	5	3.4	0.4
77.3	27	34.9	176	51.6	38.6	9.4	0.2	0.2
78.6	26.9	34.2	373	76.5	19.3	3.6	0.4	0.2
76.7	25.1	32.7	273	77.4	13	9.4	0.1	0.1
85.2	28.3	33.2	272	43.3	44.5	12	0.1	0.1
79.1	27.1	34.2	250	72.4	16.9	10.1	0.4	0.2
82.7	29.4	35.6	164	56	39.2	4.6	0	0.2
73.1	24.6	33.6	389	49.3	44.8	5.4	0.2	0.3
78	26.5	34	95	76.9	13.1	9.9	0	0.1
77.3	27.5	35.6	276	48.9	45.8	4.6	0.6	0.1
79.9	27	33.8	237	77	18	5	0	0
82.6	27.1	32.8	275	34.6	55.2	9.2	0.6	0.4
78.7	27.3	34.8	291	30.4	56.4	12.6	0.4	0.2
80	26.7	33.4	387	58.7	28.5	12.2	0.3	0.3
84.1	29	34.5	13	45.6	45.1	7.5	1.6	0.2
79.4	26.6	33.5	393	62	31	7	0	0
76.6	25.7	33.6	628	60	30	8	2	0
79	26.3	33.2	381	67.5	21.3	8.5	2.5	0.2
80.2	27.3	34	450	53.4	36.6	8.4	1.4	0.2
76.9	27.1	35.3	236	57.9	25.6	7.4	8.9	0.2
80.5	25.9	32.1	283	64	17	17	0	0
78.6	26	33.1	226	79.7	14.3	5.6	0.2	0.2
79.1	26	32.8	349	78	13	9	0	0
81.2	27.1	33.3	305	65.6	28.3	4.1	1.6	0.4
77.7	26.7	34.3	223	50.7	40.7	7.1	0.8	0.7
76.9	24.9	32.4	212	46.7	43.7	7.9	1.3	0.4
74	25.2	34	228	41.2	51.8	6.6	0.2	0.2
83.5	28.8	34.5	234	76	16	8	0	0
84.9	27.9	32.8	400	79.8	16.4	3.5	0.1	0.2
81.3	28.9	35.6	247	76.8	13.7	9.3	0.1	0.1
78.8	25.5	32.4	102	15.9	74.3	8.7	0.7	0.4
81.1	27.8	34.2	387	17.3	71.9	8.8	1.4	0.6
77.9	26	33.4	388	38.7	52.6	6	1	1.7
77	26	33.8	311	32.2	53.6	12.7	1.2	0.3
75.2	25.9	34.5	288	75.9	17.2	6.5	0.2	0.2
81.6	27.8	34.1	159	87.2	9.5	3.2	0	0.1
80.4	27.5	34.3	386	72	15.2	12.1	0.4	0.3
76.5	26.6	34.8	300	59.6	30.7	9.4	0.1	0.2
80.3	26.3	32.7	232	48.8	45.7	5.3	0	0.2
81.2	28.1	34.6	144	50.7	43.1	6	0.1	0.1
77.6	25.8	33.2	231	68	23	9	0	0

75.6	24.9	33	490	53	34	10	3	0
81.4	28.6	35.2	270	74	13	8	1	0
79	25.5	32.2	202	71	20	9	0	0
79.4	25.8	32.4	298	49.2	42.9	6.1	1.6	0.2
84.4	29	34.4	231	80	15	4.5	0	0
76.1	25.6	33.7	226	60.3	31.4	7.3	0.8	0.2
82.4	27.7	33.6	318	78	10.5	10.5	0	0
75.7	27.1	35.8	232	54	35	10	1	0
83.1	28.7	34.6	274	76	17	7	0	0
78.2	28	35.8	196	3	52	24	0	0
80.5	26.8	33.2	242	83	13	2	2	0
81.2	28.4	35	227	49.5	41.8	7.3	1.1	0.3
85	28.3	33.2	129	29.9	61.8	6.9	0	1.4
77.5	27.5	35.5	260	56	31	12	1	0
71	25.1	35.3	220	57.9	26.8	14.2	0.7	0.4
72.4	24.1	33.2	174	46.9	38.2	14.5	0.1	0.3
83.3	28.2	33.8	335	21.9	64.8	10.3	2.6	0.4
76.3	25.9	33.9	204	63	29.4	4.6	2.8	0.2
74.1	25.7	34.7	210	55	31	11	1	1
76	25	32.9	143	55.5	35.1	8.9	0	0.5
78.5	27.4	34.9	308	74	14	9	3	0
77.7	25.8	33.2	253	42.5	50.1	6	0.6	0.8
74.4	25.2	33.9	271	79	14	6	0	0
75.5	26.9	35.6	307	40	34	26	0	0
82.7	28	33.9	280	58	20	16	0	0
75.9	25.1	33	376	56.1	34.2	9.1	0.3	0.3
81.4	27.8	34.1	270	58	31	9	1	1
76.3	27.3	35.8	326	58.7	33.7	6.8	0.1	0.7
77	26	33.8	211	41	35	15	0	0
77	26.5	34.4	131	38.1	51.3	10	0.2	0.4
78.7	28	35.5	270	80.8	14	4	1.2	0
72.4	22.2	30.7	242	15	56	25	0	0
81.1	27.1	33.4	183	55.8	36.6	6.2	1.1	0.3
74.8	26	34.7	316	68	25	5	0	1
80.2	27	33.7	322	60	30	9	1	0
82.3	28	34.1	158	78	6	12	0	0
76.8	26.1	34	291	64	27	8	1	0
80.2	27.2	34	134	16	82	1	0	0
83.2	28.1	33.7	308	81.2	14.1	4.3	0.2	0.2
75.1	26.5	35.3	397	58	29	12	1	0
80.6	27.1	33.7	156	77.6	17.1	4.8	0.2	0.3
77.5	23.4	30.2	383	73.5	16.5	8.5	0.5	0
82.8	28.3	34.2	224	43	43	13	1	0
83	27.7	33.4	233	56.5	35.3	7.4	0.3	0.5
69	23.3	33.8	306	60.8	32.2	4.7	2	0.3
82.5	28.4	34.4	467	80	13	5	1	1

81.6	28.1	34.4	171	41.8	49	8.5	0.3	0.4
78	26.9	34.6	154	40.3	45.2	13.7	0.5	0.3
88.3	29.7	33.6	571	56.5	33	9.9	0.3	0.3
76.3	24.4	31.9	487	44.2	36.8	15.8	3	0.2
81	28.1	34.7	357	75.7	20.9	2.5	0.7	0.2
80.2	28.5	35.5	444	40.7	47	9.7	2.3	0.3
80.6	26.7	33.1	260	50	24	23	2	0
79	26.6	33.7	142	39.9	54.5	5	0	0.6
80.6	27	33.5	206	76.3	16.4	6.9	0	0.4
71.8	23.7	33	150	33.5	61.9	3.6	0.2	0.8
93.9	32.7	34.8	142	42	56	2	0	0
76.4	26.1	34.1	253	48.6	42.7	8	0.5	0.2
78	26.3	33.8	249	57.1	30.1	12.3	0.1	0.4
79.4	28.3	35.6	276	63	30	5.8	1	0.2
82	27.8	33.9	249	66	27	6	0	0
80.7	27.1	33.5	325	49	41	8.8	0.8	0.4
80.7	26.4	32.8	169	66.5	21.4	11.4	0.3	0.4
77.9	26	33.4	244	36	49	11	1	1
70	23.6	33.7	185	47.6	44.5	7.7	0.1	0.1
85	28	33	435	44.9	46.2	7.2	1.6	0.1
80.4	26.7	33.2	67	60.9	28.2	10.7	0	0.2
78.9	26.5	33.5	508	39.1	54.1	5	1.7	0.1
76.7	26.6	34.7	199	68.9	21.1	9.8	0	0.2
80	26.7	33.4	176	39.3	50.3	9.8	0.3	0.3
81.7	26.9	32.9	286	65	28	7	0	0
86.2	29.7	34.5	392	66.4	26.1	6.7	0.7	0.1
85.6	27.8	32.5	186	64	22	10	0	0
80.9	27.8	34.4	389	46.2	50.6	2.5	0.3	0.4
75.2	25.6	34	390	53	25	19.5	1	0
77.6	27.2	35.1	283	76.5	15.1	8.1	0	0.3
82.3	27.5	33.4	336	33.3	44.2	18.2	4.2	0.1
80.6	27.7	34.4	291	70.5	24.9	4.4	0.1	0.1
71.9	23.4	32.5	254	51.2	32.5	15.2	0.8	0.3
79.7	27.4	34.3	317	65	26	8	0	0
78.5	26.6	33.9	295	37.3	52.7	4.4	5.2	0.4
95.3	33.6	35.3	474	34	29	17	0	0
93.8	33.8	36	784	36	49.3	10.3	4.1	0.3
80	27.3	34	309	59.6	32.7	7.2	0.4	0.1
76.9	27.3	35.5	203	84.1	11.2	4.6	0	0.1
80.4	27.4	34.1	215	49.3	34.9	13.9	1.5	0.4
77.5	25.5	33	328	59.4	29.2	10.9	0.3	0.2
79.3	31.4	39.7	334	42	47	7	4	0
80.1	27.2	33.9	415	59.8	30.6	8.2	1.3	0.1
79.3	26.4	33.3	133	17	64	16	0	0
92.3	31.4	34.1	453	13.2	76.7	6.1	3.6	0.4
78.4	26.7	34.1	191	52.5	40.5	6.7	0.1	0.2

80.1	27.2	33.9	385	39	50	6	2	0
80.5	27.1	33.6	178	46.9	43.6	8.7	0.4	0.4
78.9	28	35.4	223	58.4	28	13	0.3	0.3
79.8	27.1	34	231	43	43	14	0	0
77.1	27.2	35.3	273	41	49	9	0	1
78.6	26.3	33.4	296	68	19	12	0	0
75.9	26.4	34.7	216	36.7	51	10.5	1.2	0.6
76.9	26.4	34.3	309	75.5	16.2	8.1	0	0.2
73	24.4	33.4	167	28.1	58.4	13.1	0	0.4
80.8	26.3	32.5	127	29.2	62	8	0.4	0.4
89.5	29.8	33.3	356	64.6	23.3	11.5	0.4	0.2
78.9	26.9	34.1	342	59	30.9	9.6	0.4	0.1
83.4	27.7	33.2	324	59	30	7	3	1
82	27.6	33.6	299	67.8	21.1	10.4	0.3	0.4
82.8	27.8	33.5	260	50.5	40.2	8.8	0.3	0.2
81.1	27.2	33.6	141	40.8	51.3	6.6	0.2	1.1
76.5	26.5	34.6	414	58.5	32	6.5	3	0
74.3	25.5	34.4	312	33.9	53.4	10.6	1.6	0.5
78.9	26	33	283	40.2	43.2	13.1	3.1	0.4
79.1	26.6	33.6	206	68.9	24.2	6.4	0.1	0.4
74.5	25.9	34.8	412	25.6	64.9	6.5	2.7	0.3
65.4	20.8	31.8	299	39.5	49.4	4.6	6.3	0.2
76.5	25.9	33.8	373	37.1	44.4	16.8	1.4	0.3
75.8	26.3	34.7	263	54.5	36.6	8.6	0.2	0.1
82.2	27.2	33.1	348	45	43	7	3	2
103	34.4	33.4	225	21	73	2	0	0
82.5	27.9	33.8	364	72.7	21.7	5.2	0.3	0.1
76.1	24.5	32.2	385	54	34	10	2	0
73.4	24.9	34	199	66	24	10	0	0
77.7	27.7	35.7	325	47.8	38.9	12.5	0.7	0.1
99.1	35.3	35.6	218	72.9	13.3	10.5	3.3	0
85.5	29.4	34.3	441	53.5	36.2	8.3	1.8	0.2
81	26.6	32.9	218	52	38.7	8.5	0.6	0.2
81.4	27.6	33.9	385	79.2	14	6.2	0.4	0.2
69.9	24	34.3	279	67	22	10	0	0
77.7	26.7	34.3	193	31.1	63.9	4	0.2	0.8
79.2	28	35.4	191	33	60.8	5.7	0.1	0.4
82.6	28	33.9	290	57.5	32.8	8.4	0.1	1.2
81.2	29.1	35.8	418	49	40	7	4	0
82.9	27.7	33.4	245	46	41	8	0	0
77.7	25.3	32.6	317	30.5	55.9	12.4	0.6	0.6
95.6	32.9	34.4	520	30.7	56.3	8.9	3.8	0.3
79.1	27.4	34.7	246	61.5	31	7.2	0	0.3
78.7	26.4	33.6	203	65.3	25.9	8.6	0	0.2
77.6	26.8	34.6	359	26.4	65.9	7.5	0.1	0.1
79	27.7	35.1	367	71.5	24.8	3.3	0.2	0.2

82.6	27.8	33.6	779	50	32	14	2	0
72.9	24.4	33.5	440	56.8	36.3	6.4	0.3	0.2
89.6	29.9	33.3	326	52.6	33.4	11	3	0
83.5	27.1	32.5	115	32	65	3	0	0
96.4	33.9	35.1	301	50	35	9	4	0
87.7	29.8	33.9	490	45.1	48.1	6.3	0.4	0.1
83.4	27.3	32.8	528	55	27	14	0	0
78.6	26.6	33.9	278	66	26	8	0	0
75.8	26.2	34.6	288	38.4	48.1	12.9	0.2	0.4
81.7	27.1	33.1	316	69.6	16.5	12.8	0.9	0.2
65.8	22.3	33.9	242	69.7	17.6	12.6	0	0.1
81	27.5	34	275	65	23	11	0	0
85.7	28.1	32.8	216	24.9	60.7	12.5	1.6	0.3
80.4	27.3	33.9	258	64.5	21	12.5	0	1
81.1	27.5	33.9	238	52.6	36.1	9.7	1.5	0.1
87.6	30.4	34.7	566	49.7	36.9	9.8	2.8	0.8
79.4	27.9	35.1	530	60.4	30	7.4	1.8	0.4
83.4	28.2	33.8	269	87.5	10.1	1.8	0.5	0.1
99.5	34	34.1	399	24.2	62.1	9.6	2.7	1.4
77	26	33.7	149	37.1	41.1	12.3	8.7	0.8
82.7	27.3	33	332	57.6	32.6	9.4	0	0.4
75.8	25.4	33.5	204	5	76	18	0	0
86.2	29.1	33.7	231	26.1	66.6	6	0.9	0.4
89.9	30	33.3	302	55.2	38.2	5.2	1.3	0.1
83	27.4	33	271	47.4	46.5	4.4	1.4	0.3
81.3	26.3	32.3	197	79	13	8	0	0
83	27.8	33.5	222	77.9	18.9	3	0	0.2
87.2	29.6	34	224	59.3	32	7.8	0.6	0.3
79.1	27	34.1	230	56.2	32.9	10.5	0.2	0.2
76.8	26.2	34.1	403	53	32	15	0	0
86.1	29.4	34.2	434	16.2	74	6.1	3.4	0.3
76.4	25.6	33.5	150	54	34	6	2	2
76.2	25.2	33.1	510	8	80	9	0	0
79	27.6	35	252	35.5	52.4	11.5	0	0.6
89.5	29.7	33.1	497	55.2	33.5	10.4	0.6	0.3
79.7	26.6	33.3	186	12.9	76.7	6.7	3	0.7
79.2	27.3	34.4	490	63.8	26.8	9	0.3	0.1
81.6	28.7	35.1	182	68	24	5	0	0
79.3	26.7	33.6	234	69	22	8	0	0
84	28.5	33.9	195	66.3	26.6	6.7	0.1	0.3
82.9	28	33.8	385	68.9	26	4.7	0.2	0.2
77.8	26.3	33.9	328	61.3	28.1	10.3	0.1	0.2
79.1	27.2	34.4	308	48.4	32	19.2	0.2	0.2
74.5	24.3	32.6	234	57.9	35.7	5.1	0.3	1
79.2	26.4	33.3	210	59	23	15	0	1
79.7	26.7	33.5	325	61.9	24.8	12.6	0.4	0.3

82	28	34.1	284	70.3	21.7	7.1	0.7	0.2
74.6	25.8	34.5	267	54.4	42.6	2.5	0.3	0.2
79.2	27.3	34.4	214	34	52	12	1	0
79.5	26.4	33.2	202	55.4	33.6	9	1.8	0.2
76.3	26.1	34.3	321	66.6	23.7	9.5	0.1	0.1
74.8	26	34.8	111	52.8	41.6	5.2	0.4	0
74.7	24.5	32.8	339	49	43.7	6.9	0	0.4
93.3	32.3	34.7	444	37.7	45.2	16	0.7	0.4
84.5	28.7	33.9	246	70.3	24.7	4.9	0	0.1
81.1	27.7	34.2	425	63	24	12	0	0
83.9	28.3	33.7	362	26.1	61	12.4	0.3	0.2
85.8	27.6	32.2	299	50.9	39.5	9	0.1	0.5
87.1	29.6	34	228	29.6	58.1	9.9	1.9	0.5
97.9	33.9	34.6	369	28.3	62.1	8.3	1	0.3
78.9	27.2	34.5	174	31	60	4	2	1
80.9	27.4	33.9	211	69.1	14.8	15.9	0	0.2
77.1	24.7	32.1	444	22	72	5	0	0
81.7	28.1	34.5	349	32.8	55	10.2	1.6	0.4
80.4	27.4	34.1	342	67	29	4	0	0
81.3	28.1	34.6	181	53.9	35.4	9.9	0.3	0.5
87.3	28.7	32.8	380	16.8	73.8	6.6	2	0.8
74.4	25.4	34.2	141	47.1	43.5	8.4	0.5	0.5
75.8	25.7	33.9	151	38.4	44.6	16.5	0	0.5
74.1	23.9	32.3	315	31	53.7	13.7	1.2	0.4
78.7	27.5	35	518	59.8	31.5	7.6	0.9	0.2
80.7	26.9	33.3	237	76	14	4	0	0
77.6	26.8	34.5	371	41.2	50	7.4	1.2	0.2
88.5	29.2	33	379	32.5	51.7	11	4.2	0.6
80.2	27.8	34.7	375	29.1	61.6	8	0.9	0.4
77.6	26.7	34.3	170	77	19	4	0	0
73.3	24.8	33.9	180	50	31	17	2	0
69.3	22.1	32	532	48.9	42.1	7.1	1.6	0.3
77.5	26.6	34.3	468	57.9	31.2	10	0.8	0.1
75.6	26.3	34.8	429	65	26	6	1	1
82.2	26	31.6	340	76	12	11	0	0
71	22.7	32	268	70.8	24.4	4.5	0	0.3
86.3	28.5	33	685	63	25	11	0	0
72.4	24.5	33.8	189	28.6	58.4	12.5	0	0.5
93.5	32.2	34.4	323	22	65.7	6.5	5.6	0.2
79.7	26.1	32.8	333	80	12	7	0	0
74.3	24.9	33.5	110	63.9	26.4	9.1	0	0.6
88.8	30.4	34.3	457	37	43	17	2	0
75.7	26.5	34.9	308	65.7	26.6	6.9	0.7	0.1
89.7	29.7	33.1	473	45.5	39.6	13.4	1.4	0.1
86.9	28.1	32.3	350	58.8	24.5	16.2	0.1	0.4
71.2	23	32.2	203	52	40	7	1	0

80.3	26.5	33	315	68.4	24	7.2	0.2	0.2
79.4	26.5	33.3	212	58.5	32.7	8.1	0.5	0.2
86.8	28.3	32.6	407	71	22	6	0	0
74.8	24.9	33.3	448	75	14.5	10.5	0	0
80.8	27	33.4	250	24.5	61.3	12.3	1.1	0.8
75.5	26.1	34.6	407	68.2	20.4	10.8	0.3	0.3
76	24.8	32.6	195	81.7	9.7	8.3	0.1	0.2
74.2	24.3	32.7	192	41.9	45.1	12.4	0.3	0.3
80.9	26.8	33.2	221	32	50	11	0	0
75.4	25.1	33.2	485	24.1	61	9.8	3.8	1.3
77.6	26	33.5	293	66.9	29	3.3	0.6	0.2
78.3	25.7	32.9	309	75	17.2	5.9	1.8	0.1
98.1	32.6	33.2	502	74	22	3	0	0
85	29.3	34.4	294	50	41	8	1	0
76.7	25.9	33.7	274	46.4	45.7	7.4	0.1	0.4
82.3	27.7	33.6	255	57.2	29.1	11.6	1.9	0.2
79.1	26.2	33.1	173	35.1	52.2	11.4	0.3	1
77.7	26.8	34.4	291	74.2	16.4	8.9	0.1	0.4
95.6	33.6	35.1	212	72	9	16	1	0
82.1	29	35.3	569	41.8	49.8	7.6	0.6	0.2
80.9	27.5	34	288	67.2	27.6	4.3	0.8	0.1
80	27.7	34.6	277	46	30	23	0	0
75.5	26.6	35.2	243	41.1	47.9	10.6	0	0.4
74.1	25.7	34.6	227	68.3	24.1	7.4	0	0.2
76.8	26.1	34	237	73	19	7	0	0
88	28.9	32.8	782	23	49	26	2	0
78	26.1	33.4	300	52.3	32.4	14.7	0.3	0.3
78.1	26.8	34.3	301	57.8	30.9	10.5	0.6	0.2
77.5	26.4	34	312	82.6	12.4	4.9	0	0.1
76.5	26.6	34.8	234	54	37.7	5	3.2	0.1
83.2	27.8	33.4	162	76.8	15.7	7.3	0	0.2
79.5	26.2	33	456	57.6	27.6	13	1.7	0.1
79.6	27.6	34.6	204	60.9	27.3	11.4	0.2	0.2
77.3	25.5	33	286	35	36	27	1	0
87.6	29.8	34	506	48.6	46.8	3.7	0.6	0.3
79.6	26.8	33.6	445	58.3	30.6	10.7	0.2	0.2
90.3	30.8	34.2	309	27.6	59.1	9.2	3.9	0.2
78.8	27.6	35	264	49.8	37.2	10.6	2.1	0.3
80.3	27.2	33.9	203	51.2	38.4	9.9	0.1	0.4
78.7	26.4	33.6	159	4	88	7	0	0
76.2	25.8	33.8	191	66.5	27.4	5.9	0	0.2
81.4	27.6	33.9	221	66.4	23.3	9.7	0	0.6
79	25.2	31.9	305	53.1	37.1	9.5	0.1	0.2
80.6	27.4	34	316	57.4	30.2	9.9	2.1	0.4
82	27.3	33.2	127	51	41	7	1	0
84.7	28.5	33.6	188	46.7	41.8	10.6	0.5	0.4

73.9	24.3	32.9	404	41	46	7	5	0
78.3	26.9	34.3	240	53.8	37.8	7.3	1	0.1
78.1	25.3	32.4	245	69	15	16	0	0
73.7	24.3	33	188	53.9	38.2	7.5	0.2	0.2
77.5	26.3	33.9	142	29	66	5	0	0
80.7	27.6	34.2	116	45.7	41.3	12.6	0.2	0.2
91	31.2	34.2	285	46.7	43.3	7.9	1.6	0.5
80.6	27.4	33.9	136	71.5	23.6	4.3	0.2	0.4
78.2	27.1	34.7	188	57	35	8	0	0
85.9	28.8	33.5	234	84.2	9.9	5.5	0.2	0.2
79.5	26.2	32.9	282	39.7	49.1	8.8	2.2	0.2
78.4	27.2	34.7	219	69	18	9	1	0
78.7	26.6	33.8	492	38.7	49.5	9.9	1.8	0.1
74.2	24.4	32.9	286	62.5	23.1	14	0.1	0.3
83.1	28.5	34.3	258	43	43	11	2	1
86.3	28.5	33.1	359	89	5	5	1	0
78.4	26.9	34.3	252	75.3	13.9	9.4	0.9	0.5
91	32	35.2	446	29.3	57.4	7.5	5.5	0.3
80.4	27.3	34	125	16	75	8	0	1
83.7	28.3	33.8	188	48	43	8	1	0
77.3	26	33.7	251	29	61	8.4	1.2	0.4
91.5	30.6	33.4	193	8	44	48	0	0
80.3	26.6	33.1	232	68	22	10	0	0
77.8	26	33.4	221	70	17	6	0	0
79.1	27.2	34.3	374	79.4	14.7	5.3	0.4	0.2
80.6	26.9	33.3	339	65.2	27.3	6.7	0.4	0.4
89.3	29.5	33	369	63	26.7	8.8	1.4	0.1
81.3	26.7	32.9	248	81.4	13.9	3.9	0.6	0.2
83.3	28.8	34.6	271	58.5	30.1	10.5	0.6	0.3
78.3	27.7	35.3	126	38.4	56.8	4	0.7	0.1
79.3	26.3	33.2	113	46.4	42	9.2	0.6	1.8
91	31.8	34.9	408	25.6	63	8.2	2.9	0.3
78.5	27.2	34.6	328	56.3	33.5	9.6	0.4	0.2
100.6	34.6	34.4	284	14	60	24	1	0
77.8	26.8	34.5	132	73.5	21.7	4.5	0.1	0.2
76.2	26.6	34.9	368	36.3	51.4	5.3	6.5	0.5
78.8	27.1	34.4	256	60	27	12	0	1
80.7	26.9	33.3	531	31	58	5	6	0
76	25.4	33.4	346	10	62	24	3	0
79.4	26.9	33.9	373	59	30	10	0	0.5
77.3	25.7	33.2	213	58.6	29.4	11.2	0.5	0.3
79.5	27.7	34.8	316	70.2	20.7	8.3	0.6	0.2
77.6	26.3	33.9	467	73.6	13	13.2	0.1	0.1
79.3	26.7	33.7	314	55.3	36.5	7.9	0	0.3
76	26.3	34.7	249	67.3	27	5.3	0.2	0.2
80.6	27.7	34.4	223	58	25	14	1	0

76.4	25.9	33.9	274	20.9	72.2	6.3	0.3	0.3
77.4	26.6	34.4	331	1	87	7	5	0
71.9	24.4	33.9	363	26	68	6	0	0
77.2	26.6	34.5	207	71	24	4	1	0
75.8	24.2	32	442	46	43	10	0	0
80.6	27.9	34.6	368	65.5	28	5	0.5	1
78.6	26.2	33.3	106	72	15	7	0	0
84.4	29.2	34.6	326	83	7	6	1	0
75.3	25.5	33.9	528	59.2	32	6.6	1.8	0.4
83.2	28.9	34.7	229	47	44	7	2	0
82.5	28.3	34.3	268	72	19	9	0	0
75.9	25.6	33.8	158	65.6	24.9	9.3	0.2	0
76	25.9	34.1	365	61.8	24.2	12.4	1.4	0.2
74.6	24.4	32.7	255	33	56	11	0	0
81.9	28.5	34.8	342	10	88	2	0	0
77.4	26.2	33.8	336	66	14	16	0	0
74.3	25.7	34.5	242	17.4	75.5	6.5	0.4	0.2
75.5	24.5	32.5	318	35	60	3	0	0
79.5	27.4	34.4	198	38	36	17	0	0
73.9	25.1	34	191	14.9	79.3	4.7	0	1.1
82.1	27.5	33.4	334	78.6	15.3	5.8	0.1	0.2
86.1	28.7	33.3	313	46.1	43.1	9.1	0.9	0.8
84	28.3	33.7	376	76	19	5	0	0
77.6	26.8	34.5	622	1	90	5	3	0
82	28	34.1	343	60	25	10	1	0
81.9	27.7	33.8	225	78.5	12.2	8.3	0.8	0.2
78.9	26.6	33.6	120	53.5	39.6	6.1	0.6	0.2
79.1	26	32.8	229	52.1	38.5	9.1	0	0.3
79.8	27.4	34.3	153	52.1	35.9	10.9	0.7	0.4
76.5	25.4	33.2	329	79.7	14.6	4.6	0.9	0.2
81	27.4	33.9	178	61.5	20.6	17.5	0.2	0.2
80.5	28.3	35.1	225	69	25	5	0	1
82.1	26.8	32.6	260	60	28.4	11.2	0	0.4
78.6	27.2	34.6	193	31.8	57	10.3	0.4	0.5
82.5	28.5	34.5	254	48	45	7	0	0
80.2	27.6	34.5	202	41	47.7	9.2	1.8	0.3
76.4	26	34.1	145	57.6	29.7	12.3	0.2	0.2
80.6	27.8	34.5	681	66.3	24.5	8.8	0.3	0.1
78	27.2	34.9	208	67.5	23.2	9	0.2	0.1
77.5	26.2	33.8	127	58.7	31.5	7.3	2.1	0.4
79.6	26.1	32.8	308	28.5	62	7	0	0
76.3	26	34	415	78	10	12	0	0
80.4	27.2	33.8	253	63.4	26.3	9.2	0.9	0.2
79.1	27.1	34.2	341	42.9	41.7	13.8	1.2	0.4
81.2	28	34.5	337	58.3	31.5	8.9	1.1	0.2
85.1	28.8	33.8	215	55.5	33.5	10.8	0	0.2

84.7	28.4	33.5	98	52	37	7	4	0
82.7	28.5	34.4	183	49	27	18	4	0
83.2	28.1	33.7	379	37	53.3	7.9	0	1.8
78.6	27.2	34.6	306	37.9	50.2	10.5	1	0.4
77.9	27.5	35.3	445	19.3	72.1	4.4	3.9	0.3
90.5	30.9	34.2	509	40.7	48.9	8.6	1.6	0.2
78.8	27.5	34.9	362	84.8	12	3	0.1	0.1
90.2	31.9	35.4	395	54.9	37.4	4.7	2.8	0.2
80.5	27.2	33.8	204	43.4	48.3	7.8	0.2	0.3
75.1	25.5	33.9	266	83.6	7.1	9	0	0.3
76	25.6	33.7	349	67	19	12.3	1.5	0.2
81.2	27.6	34	324	66.9	21.1	10.8	0.8	0.4
80.8	27.8	34.4	156	44.2	48.7	6.3	0.4	0.4
76	26.1	34.3	346	73.2	18.3	7.7	0.4	0.4
78.7	28.1	35.7	337	52.2	40.4	6	1.3	0.1
73.4	24.7	33.6	503	19	72	5	3	1
75.7	25.9	34.3	208	57	29	13	1	0
82.8	28.3	34.2	248	52.2	37.3	9.9	0.4	0.2
78	27.8	35.6	250	50	42	7	1	0
76.6	25.3	33	434	22	67	4	4	0
80.1	27.9	34.9	239	84.5	10.6	3.9	0.7	0.3
75	24.8	33.1	521	55.6	35.5	7.7	0.9	0.3
66.7	22	32.9	173	62.5	29.9	6.6	0.8	0.2
78.3	26.4	33.7	361	61.5	28.2	10	0.1	0.2
80.6	27	33.5	209	75.3	15.4	8.7	0.1	0.5
84	27.6	32.8	384	60.5	32.2	6.9	0.2	0.2
81.1	28.1	34.6	243	61	33.2	5.2	0.4	0.2
87.3	29.6	33.9	426	23	59	14	3	1
73.4	26.2	35.6	190	63.8	28.2	7.4	0.3	0.3
85.2	29.6	34.7	471	50	28	20	2	0
86.8	28.5	32.8	604	34.9	53.5	10.9	0.5	0.2
79.7	26.4	33.1	239	47	30	23	0	0
81.8	28	34.2	169	79.5	13.8	6.5	0.1	0.1
72.6	22.4	30.9	246	58.9	33.4	6.8	0.6	0.3
72.6	24.7	34	288	63.9	26.9	8.3	0.7	0.2
83.4	28.2	33.9	438	25	70	3	1	1
90.6	32.2	35.5	375	32.3	56.7	5.5	5.2	0.3
76.5	26.5	34.6	191	71	21	8	0	0
75.5	26.2	34.7	258	63	25	11	1	0
83.1	28.7	34.5	412	61.2	29.2	8	1.3	0.3
83	29.1	35	254	70.4	21.6	7.8	0	0.2
74.6	24.5	32.8	235	56	26	14	4	0
83.2	27.6	33.2	272	49.8	39.3	9.5	0.9	0.5
81.1	27.6	34.1	291	71.9	17.8	9.1	0.9	0.3
85.8	28.8	33.6	414	75.7	17.9	6	0	0.4
82.8	28.3	34.1	215	54.6	32.8	12	0.2	0.4

76.5	28	36.6	371	61.7	31.2	6.3	0.7	0.1
78.6	25.9	33	380	46.9	38.3	12.6	2	0.2
85.6	30.4	35.4	417	34.6	55.8	9.1	0.4	0.1
85.2	28.2	33.1	269	56.2	31.1	11.4	0.5	0.8
74.4	24.9	33.5	260	76.5	15	5	1.5	0
81	27.8	34.4	224	61.1	28.1	10.4	0.3	0.1
79.3	26.4	33.3	369	16	70	9	3	0
83.9	28	33.4	169	31.1	59.7	7.6	1.1	0.5
71.2	24.9	35	374	83.8	5.6	10.1	0.3	0.2
76.8	26.1	34	354	77.3	14.6	7.7	0.2	0.2
80.8	26.8	33.1	419	55.2	35.6	7.7	1.1	0.4
104.5	36.4	34.8	161	86	9	5	0	0
92	31.2	33.9	523	44.8	42.2	9.2	3.4	0.4
77.4	25.4	32.8	375	48	40	7	5	0
91.8	31.2	34	461	20.6	62.8	8.1	8.1	0.4
78.8	26.3	33.4	281	46	43	10	0	0
79.4	28.9	36.4	252	82.2	13.2	4.5	0	0.1
80.9	27	33.3	265	42.9	51.2	4.9	0.6	0.4
78.8	25.6	32.5	198	30.3	61.1	7.4	0.5	0.7
79.8	27.3	34.2	267	45.4	45.1	6.8	2.1	0.6
77.6	26	33.5	227	35.7	55.5	7.8	0.7	0.3
75.3	25.1	33.3	120	47	47.2	5.2	0	0.6
76.5	26	33.9	146	50.6	41.1	6.4	1.5	0.4
82.6	29.2	35.4	176	54.4	37.3	7.6	0.5	0.2
72.9	24	32.9	219	50.4	38.7	10.3	0.2	0.4
77.3	26	33.6	248	52	36	12	0	0
78.4	26.8	34.2	221	35.4	51.8	10.3	2	0.5
78.6	27	34.4	242	51	36	7	2	2
107.8	36.3	33.7	203	63	24	12	1	0
76.8	26.7	34.7	265	74.4	17.1	8	0.3	0.2
81.6	28.5	34.9	144	61.4	26	12	0.3	0.3
76.7	25.9	33.8	511	58.4	33.2	7.1	0.9	0.4
80.4	27.9	34.7	215	76.5	18.2	4.7	0.3	0.3
80.5	27.7	34.3	242	54.7	31.1	11.9	1.4	0.9
79.2	26.7	33.7	368	60	32	7	1	0
76.9	26.9	35	202	56.4	35.5	7.8	0	0.3
88.8	30.8	34.7	376	19.2	71.5	6.2	2.7	0.4
88.7	29.8	33.6	536	31.3	57.5	7.3	3.8	0.1
77.1	25.8	33.5	293	70.8	19.7	9.3	0	0.2
82.1	27.8	33.9	166	59	24.5	16.1	0.1	0.3
103.1	35.6	34.5	389	37	44	18	1	0
97.9	34.8	35.6	235	48	36.5	11.1	3.9	0.5
102.5	35.6	34.7	233	37.5	52.8	7	2.5	0.2
81	27.2	33.6	265	82.9	11.4	4.9	0.7	0.1
76.1	25.8	34	172	70.7	21.4	7.3	0.3	0.3
84.1	28.6	34.1	419	20	62	17	1	0

74	24.7	33.4	405	60.7	28	10.1	0.9	0.3
79.4	26.6	33.5	213	49.1	43.5	6.8	0	0.6
79.5	26.8	33.7	208	61.7	27.7	10.3	0.1	0.2
74.8	25.6	34.2	434	31.2	53	5.1	10.5	0.2
85.7	30.6	35.7	427	72	20	6	0	0
69.6	23.8	34.1	412	74	18	7.5	0	0
98.4	35.7	36.2	245	61	18	19	2	0
73	25	34.2	538	36.5	51.1	11	0.5	0.9
74.6	24.7	33.1	512	71.5	16.5	8	2.5	0.5
82.8	26.9	32.5	374	39	54	4.7	2	0.3
76.1	26.3	34.5	278	11	83	6	0	0
71.6	24.6	34.4	268	57	32	11	0	0
81.8	28.1	34.4	198	72	9	4	0	0
80.7	26.5	32.8	214	49.6	38.2	11.2	0.3	0.7
77.9	26.4	33.9	265	30.6	57.4	11.2	0.4	0.4
79.1	26.9	34	366	57.9	31	10.2	0.5	0.4
76	26.4	34.8	186	48.9	39	11.6	0.2	0.3
83.1	27.5	33.1	278	54	33	13	0	0
82.4	28.8	34.9	264	49.4	35.8	13.8	0.4	0.6
80	26.6	33.2	361	67.5	25.6	6.2	0.5	0.2
75.1	26	34.7	201	54.5	35.7	8.6	1	0.2
76.6	26.4	34.4	100	29	54	14	0	0
77.2	25.9	33.5	297	29.5	54.3	14.6	0.9	0.7
81.3	26.6	32.7	284	44.1	47.3	7.6	0.4	0.6
75.3	26	34.5	535	80.2	13.3	5.6	0.6	0.3
77.7	26.4	34	317	37.2	48.9	12.9	0.6	0.4
76.1	27.1	35.6	273	58.2	32.4	8	1.1	0.3
79.7	27.1	34	193	70.3	18.6	10.4	0.4	0.3
86.3	29.8	34.5	360	12	62	18	4	0
80.4	27	33.6	327	87	4	1	0	0
79.4	27.2	34.2	252	81	16.2	2.6	0.1	0.1
79.8	26.4	33.1	332	71.9	18	9.6	0.3	0.2
76.2	25.4	33.3	353	74.5	15.3	9.7	0.3	0.2
80.3	28.1	35	250	36.2	49.2	12	2.3	0.3
78.5	26.8	34.2	378	54.5	36.7	7.5	0.8	0.5
81.7	27.6	33.8	240	63.9	25	10.2	0.6	0.3
79.7	26.9	33.8	248	53.6	33.2	12.6	0.3	0.3
82.9	28.4	34.2	307	51	41.1	7	0.7	0.2
97.6	33.5	34.4	281	54	20	22	0	0
74.3	25.3	34	230	49.6	40.8	9.2	0.2	0.2
82.5	27.6	33.4	370	78	15	5	2	0
83.6	29.3	35	213	22.8	66.5	6.7	3.7	0.3
77.7	25.2	32.4	211	28.3	59.2	10	0.7	1.8
71.3	23.5	32.9	221	53	40	5	2	0
86.2	29	33.7	275	51.8	40.2	7.1	0.7	0.2
80	28.9	36.1	183	57.6	35.3	6.5	0.1	0.5

83.9	28.5	33.9	168	21.4	62.2	15.1	0.2	1.1
81.3	27.5	33.8	203	41.1	47.9	9.2	1.2	0.6
78.7	26.1	33.2	195	65.6	27.8	6.1	0	0.5
82.9	28.2	34	148	46.7	40.3	10.1	1.7	1.2
85	28.8	33.9	197	44.1	44.3	9.8	1.5	0.3
86.3	28.9	33.4	367	65.5	30.9	3.2	0.1	0.3
83.8	27	32.3	226	51	42	7	0	0
79.4	27.1	34.2	154	45.5	43.3	10.5	0	0.7
81.2	27.3	33.6	207	56.7	31.5	11.4	0.2	0.2
73.3	24.3	33.2	305	34.2	51.7	12	1.5	0.6
77.7	25.9	33.3	200	73	19	7	0	0
79.5	28.1	35.4	264	48	31	15	2	0
96.9	33	34.1	413	25.9	65.3	4.6	3.5	0.7
82	28.3	34.5	206	85.1	7	7.7	0.1	0.1
91	29.8	32.7	258	44	48.2	7.6	0	0.2
74.2	23	31.1	331	48.8	40.1	9.8	0.9	0.4
82.3	27.3	33.1	127	48.8	41.7	8.9	0.3	0.3
79.5	27.2	34.1	257	58.5	27.4	11.2	2.6	0.3
75.5	26.7	35.3	405	48.9	41.7	8.9	0.3	0.2
76	25.2	33.1	481	42.8	49.3	6.5	1.2	0.2
75.4	26.5	35.2	300	63	16	5	0	0
84.1	28.5	33.9	180	58.4	28.9	12.2	0.3	0.2
84.9	28.6	33.8	390	52	26	22	0	0
95.1	32.8	34.4	339	26.8	61	7.7	4.2	0.3
80.1	27	33.7	370	63.5	19	16	0.5	0
76.5	26.1	34	272	64.8	32.4	2.4	0.1	0.3
79.4	27.7	34.9	406	46.2	45	7.4	0.8	0.6
79.4	27	34	226	52.5	38.9	7.9	0.5	0.2
82.7	26.8	32.4	555	57.4	35.2	6.5	0.6	0.3
78.3	26.2	33.4	239	59.5	29.6	9.4	1	0.5
82.5	28.7	34.8	177	53.2	40.1	6.4	0.1	0.2
79.6	26.2	32.9	230	30.9	60.3	7.5	0.4	0.9
83	28.4	34.2	400	51.7	37.5	7.4	2.9	0.5
78.1	26.7	34.2	302	79	13	8	0	0
80.4	26.7	33.1	214	85.3	8.6	5.5	0.3	0.3
93	31.5	33.9	643	46	40.9	9.3	3.3	0.5
65.9	21.5	32.6	215	52.5	37.9	5.5	4	0.1
77.9	26.5	34	232	37.1	58.6	3.7	0.1	0.5
77.1	25.5	33.1	316	70.5	22.2	7.2	0	0.1
77.5	25.9	33.4	224	70	22	7	1	0
67.5	22.7	33.7	485	62.2	27.3	10.1	0.2	0.2
74.3	25	33.6	354	62	27	11	0	0
92.4	32.7	35.4	272	55.7	34.2	5.9	3.9	0.3
78.5	27.2	34.6	346	56.7	34.2	8.3	0.6	0.2
76	25.6	33.6	169	71.6	20.9	7.4	0	0.1
70.4	21.5	30.5	140	65	17	13	0	0

84.2	29.2	34.7	343	43	41.2	14.7	0.7	0.4
78.2	27.5	35.2	188	74.6	20.6	4.1	0.6	0.1
83.7	27.8	33.2	473	58	34	8	0	0
82.5	27.7	33.6	216	76.8	13.5	9.7	0	0
80.7	28.3	35.1	187	40.6	46.7	12	0	0.7
79.3	27	34.1	172	62	19	11	0	0
78.8	27.4	34.8	277	32.2	62.3	4.4	0.8	0.3
81.8	28.1	34.3	219	48.9	41.8	8.8	0.3	0.2
77.4	25.1	32.4	353	40.3	48.1	5.3	5.1	1.2
82.4	27.5	33.4	148	47	48.5	2.7	1.6	0.2
81.4	28.1	34.6	478	73	22	5	0	0
74.9	25.9	34.6	221	75.7	17.3	5	1.8	0.2
83.5	29.2	35	214	58.5	34.8	6	0.3	0.4
76.7	29.2	38	161	31.8	55.6	10.5	0.8	1.3
77.7	26.8	34.5	314	52.9	35.8	10.2	0.7	0.4
73.4	25.4	34.7	414	88	9	3	0	0
82.7	27.9	33.8	455	62	29	8	1	0
81.6	28.4	34.8	229	30.3	60.2	7.7	0.9	0.9
82.2	27.7	33.7	201	56.6	31.9	9.3	1.7	0.5
91.9	29.2	31.8	618	51	26	21	0	2
82.6	28.4	34.4	298	62	29	5	2	1
86.2	28.4	33	317	75	21	4	0	0
93.6	32.7	34.9	434	38	33	23	0	0
78.2	25.8	32.9	478	52.9	36.8	8.1	2.1	0.1
77.5	25.4	32.8	246	52	43	3	1	0
76.3	25.9	34	476	42	48	8	1	1
75.7	25.9	34.3	266	73.9	20	5.5	0.4	0.2
76.9	25.2	32.8	288	53.5	40.1	5.4	0.5	0.5
71.4	23.6	33.1	314	53.6	36.9	8.9	0.3	0.3
80	26.9	33.6	377	56	34	8	2	0
73.8	25.4	34.5	415	63.5	26.9	8.5	0.9	0.2
89.5	29.9	33.4	426	54	42	3	0	1
79	26.2	33.1	254	55.5	36.6	7	0.5	0.4
78.5	27.6	35.1	394	44.5	39	14.5	0	2
80.3	27.7	34.5	327	39.3	53.9	4.4	2	0.4
77.1	25.6	33.1	206	61.7	32.3	5.8	0	0.2
78.7	27.3	34.7	270	51.5	32.5	11	0	0.5
88.1	31	35.3	340	53.3	44.1	1.8	0.7	0.1
80.4	27.3	33.9	244	71.5	18.9	5.3	4.2	0.1
77	25	32.5	343	44	42	6	5	1
76.5	26.5	34.7	305	43	45	10	1	1
82.9	27.9	33.6	625	53.2	40.5	5.4	0.6	0.3
80.7	27.7	34.3	40	69	26	3	0	0
79.5	27.6	34.7	360	71.4	25.1	3.2	0.1	0.2
73.3	25.2	34.4	375	33.1	58.1	6.8	1.7	0.3
81.7	27.8	34	234	70.1	20.2	8.7	0.8	0.2

74.7	25.6	34.2	290	48.6	41.8	9.3	0.2	0.1
79.7	26.5	33.2	419	68.5	24.2	6.7	0.5	0.1
84.4	28.6	33.9	352	37.8	52.3	7.3	2.4	0.2
77	26.1	34	292	73.5	12.5	12.5	0	0
65.7	21.5	32.7	285	62.7	25	11.8	0.3	0.2
87.3	30.2	34.6	176	60.7	34.9	4.2	0	0.2
81.2	26.9	33.1	400	62	27.5	7.5	3	0
74.7	26.7	35.8	367	56	33	11	0	0
81.4	27.4	33.7	324	54	37	8	1	0
85.6	28.6	33.4	278	29.7	62.5	7	0.4	0.4
89.1	28.1	31.5	406	16.7	78.4	3.9	0.7	0.3
77.9	26	33.3	585	60	28.4	10.5	0.7	0.4
76.6	25.1	32.7	200	45.9	34.8	16.5	1.9	0.9
79.6	26.7	33.5	229	38.6	51.4	8.8	0.3	0.9
77.2	26.3	34.1	299	54.2	37	8.1	0.5	0.2
73.8	25.3	34.2	233	81.8	10.4	7.6	0	0.2
87.1	30.7	35.2	268	45.2	42.7	7.8	4	0.3
89.2	30.5	34.1	483	48.1	39.8	10.5	1.3	0.3
83.6	28.4	33.9	346	45	37.8	12.1	4.6	0.5
92	30.9	33.6	432	51.7	40.1	7.4	0.7	0.1
82.1	29.2	35.5	387	63.9	27.1	6.5	2.4	0.1
76.3	25.8	33.9	249	69	21	9	0	1
80.1	26.7	33.3	298	45.9	40.3	11.6	2	0.2
78	26.4	33.9	227	36	51	10	3	0
77.6	27.7	35.7	280	76.2	11.8	11.6	0.3	0.1
80.6	27.8	34.5	229	68.3	22.8	8.4	0.3	0.2
93.8	31	33.1	305	63.9	25.2	9.9	0.5	0.5
104	34.4	33.1	407	47.6	44.9	7.1	0	0.4
87.2	30.2	34.6	310	54.2	28.7	16.9	0.1	0.1
80.7	27.1	33.6	151	11	84	4	0	0
78.7	25.1	31.9	611	66	21	5	1	0
78	26.5	34	383	54.5	35.6	9.3	0.2	0.4
82.1	28.6	34.9	210	37.8	55.5	5.5	0.3	0.9
79.3	27	34	234	42	50.8	6.2	0.7	0.3
90.8	29.2	32.2	279	55	32	13	0	0
76	26.1	34.3	274	49.3	39.4	10.6	0.4	0.3
76.4	26.1	34.2	197	55.2	34.3	9.7	0.4	0.4
104	36	34.6	241	68.8	25.8	4	1.1	0.3
80.5	27.4	34.1	201	38.6	52.1	8.6	0.2	0.5
77.6	26.9	34.7	321	67	21	10	0	0
75.6	25.5	33.7	270	72.4	16.3	10.9	0.3	0.1
76.1	26.5	34.8	270	73.1	21.8	4.3	0.6	0.2
81.5	27.3	33.5	199	75	18	6	0	1
77.7	26.8	34.5	540	41.9	47.9	9	0.8	0.4
100	34.7	34.7	439	53	36	8	3	0
77.1	26.5	34.4	326	54.3	35.1	8.8	1	0.8

73.1	25.4	34.7	296	39	37	13	0	0
74.5	25.5	34.3	495	38	46	13	2	1
107.6	37.3	34.7	372	35	44	19	0	0
84	29.6	35.3	339	57.9	31.1	9.4	1.3	0.3
86.9	29.9	34.4	212	26	58	8	8	0
75.7	24.6	32.5	331	35.5	56.4	6.1	1.7	0.3
86.2	29.8	34.6	351	34.5	49.1	14.1	2	0.3
91.3	31.6	34.6	377	45	49.9	3.7	1.2	0.2
76.7	26.3	34.2	211	73	17	8	0	0
75.2	25.1	33.3	246	68	22	8	0	0
80.4	27.6	34.3	26	4	56	2	0	0
79.2	26	32.9	184	35.3	55.5	8.7	0	0.5
82.6	27.5	33.3	287	49.6	42.4	6.8	1	0.2
84	28	33.3	362	38	56	3	1	0
82.2	27.7	33.7	90	28	59.9	10.6	1.1	0.4
75.7	25.8	34.1	232	66.1	24.6	8.5	0.4	0.4
86	29.3	34	417	42.1	49.7	7.1	0.6	0.5
77.2	26.8	34.7	238	72	17	9	0	0
87	29.7	34.2	380	54.1	28	15.3	2.1	0.5
83.8	26.9	32.1	146	58.6	28.3	11.5	1.3	0.3
78	25.9	33.1	186	39	34	14	0	0
74.1	24.2	32.6	663	50.7	36.1	12.4	0.4	0.4
82.1	28.1	34.2	193	85	10	5	0	0
80.5	26.8	33.3	319	84	6	4	0	0
93.4	31.8	34.1	370	48	38	9	0	0
69.2	22.8	33	301	54.6	37.2	7.6	0.5	0.1
77	25.5	33.1	230	57	28	14	0	1
77.5	26.2	33.8	267	48	40.4	10.4	0.7	0.5
75.7	26.1	34.4	831	26	67	5	1	1
84.1	28.4	33.7	241	54.9	34.4	9.8	0.1	0.8
81.9	28.4	34.7	179	32	61	7	0	0
79.4	26.9	33.9	192	43	35	19	3	0
88	29.6	33.7	517	40	41	15	4	0
77.5	27.3	35.2	214	67.4	24.5	7.7	0.2	0.2
78.2	27	34.5	457	75.5	17.5	5	0.5	0
79.7	26.7	33.5	450	62.7	23.9	9.5	3.5	0.4
78.1	27.2	34.8	46	24.1	61.1	12.4	1.9	0.5
80	27.3	34.1	344	72.5	16.5	8.5	1	0.5
82.4	27.9	33.9	574	45.1	49.3	4.1	1.1	0.4
75.3	24.9	33.1	189	67.4	23.1	9.2	0	0.3
88.3	29.6	33.5	563	58.2	30.5	10.6	0.6	0.1
81.5	28.7	35.2	484	49.4	42.6	6.9	0.9	0.2
80.3	26.8	33.4	173	57.8	32.3	9.6	0	0.3
99.6	33.3	33.5	202	52.3	37.4	9.7	0.3	0.3
79.5	26.8	33.6	249	60.6	28.8	10.3	0.1	0.2
81.4	27.2	33.4	307	55	34	9	0	0

75.7	26.7	35.3	248	58	29	11	0	0
73.9	24.7	33.4	424	46.7	45.4	4.6	3.1	0.2
78.8	26.4	33.5	338	64	18	18	0	0
77.7	26.6	34.3	265	52.3	37	9.7	0.8	0.2
69.5	22.2	31.9	302	50	33	15	2	0
98.5	34	34.5	415	39	45	10	5	0
81.7	26.5	32.5	290	69.2	18.1	4.3	8.1	0.3
79.5	26.9	33.8	186	48.2	42.4	9	0.1	0.3
95.7	32.2	33.7	641	14.2	74.8	6.3	4.1	0.6
79.3	26.6	33.5	228	70	25	5	0	0
80.1	27	33.7	210	45.1	38.6	12.7	2.7	0.9
74.4	24.3	32.7	336	56.5	32.5	9	0	0
78.1	26.2	33.6	197	65.2	23.7	10.5	0.3	0.3
76	26	34.2	455	61.9	32.3	4.3	1.2	0.3
77	25.7	33.4	479	77.4	14.6	7.6	0.3	0.1
72.6	24.5	33.7	318	89.1	7.6	3.1	0.1	0.1
83.5	27.9	33.5	350	29	59	12	0	0
76.5	27.3	35.6	298	62.6	29.1	8	0.1	0.2
79.2	26.3	33.2	363	52	36	11	1	0
83.4	28.5	34.2	377	76.5	14.9	8.2	0.1	0.3
74.5	24.8	33.2	390	49	43	3	0	0
72.8	24.9	34.2	345	42.8	48.5	6.5	1.6	0.6
79.2	27.5	34.8	415	35.2	47.6	14	2.6	0.6
96.6	33.2	34.3	547	41	36	21	2	0
76.7	26	33.9	280	69.4	19.3	10.6	0.3	0.4
72.7	25.1	34.5	319	86.5	6	7.5	0	0
85.5	28.6	33.4	292	63.1	24.3	12.2	0.1	0.3
88.3	29.1	32.9	314	47	42	9	1	1
82.7	27.6	33.4	231	52.5	28.2	17.4	1.3	0.6
96.1	33.3	34.7	741	33	54	9	4	0
76.7	27.3	35.6	225	64.7	22	12	0.9	0.4
80	27	33.8	180	43	45	11	0	0
77.9	26.6	34.2	307	76.7	10.4	11.1	1.5	0.3
77.4	25.2	32.5	156	87	11	1.9	0	0.1
75.8	25.1	33.1	534	69	21	5	0	0
78.3	25.3	32.2	256	50	38	12	0	0
81	27.3	33.7	313	93	3.5	3	0	0
78.3	27.2	34.7	275	51.7	38.7	5.1	4.4	0.1
91	30.4	33.4	403	27.9	64.5	4.6	2.6	0.4
82.6	27.6	33.4	290	82.5	11.5	6	0	0
95.4	33.6	35.2	202	67	19	11	0	1
78.5	27.1	34.5	194	48.5	43.4	8	0	0.1
77.1	27.1	35.1	307	81.3	10	7.1	0	0
80.7	26.5	32.9	219	30.4	58.9	8.9	1.5	0.3
90.8	31.5	34.6	483	47.2	33.9	17.2	1.6	0.1
84.3	27.3	32.4	292	40.3	44.6	13.9	0.6	0.6

77.7	26.7	34.3	186	86.6	8.9	4.3	0	0.2
79.5	26.7	33.6	397	47.1	41.1	11.1	0.3	0.4
79.1	27.2	34.4	221	64.5	24.6	9.6	1.1	0.2
79.8	27.5	34.4	191	42	43	14	0	0
83.3	27.4	33	358	80	16	3.5	0	0
80.1	27.5	34.3	288	58	32	9	1	0
81.1	26.8	33	466	50.9	43.9	2.9	2	0.3
79.7	26.7	33.5	226	85	9	6	0	0
77.8	24.6	31.6	93	47	42	10.3	0	0.7
94.6	31.6	33.4	493	56.1	34.9	7.5	1.2	0.3
80.8	27.3	33.8	229	58.4	35.2	5.9	0.2	0.3
76.5	25.8	33.7	248	54.6	33.6	10.4	1.2	0.2
91.2	30.9	33.9	528	36.1	49.4	14.1	0.2	0.2
70.7	22.6	31.9	219	19	64	10	2	2
78	27.9	35.8	401	46	48	6	0	0
81	27.3	33.7	302	62.9	29.6	7.3	0.1	0.1
76.7	26.3	34.4	233	74.6	15.8	8.9	0.4	0.3
82.4	27.7	33.7	169	60.7	28.7	10.3	0	0.3
82.3	27.8	33.7	204	60.9	31.9	6.8	0.3	0.1
75.2	25.4	33.8	236	41.1	50.6	7	1.1	0.2
68.2	22.5	33	215	63.6	24.9	7.3	3.8	0.4
75.9	25.5	33.7	170	51.2	36.3	12.1	0.2	0.2
77.8	26.5	34	186	34	55	8	1	0
78.8	27	34.2	252	32	61	2	0	1
77.7	26.6	34.2	281	62	31	7	0	0
83.2	27.9	33.6	174	5	62	19	0	1
73.3	25.2	34.4	464	79	13	7	1	0
75.9	24.4	32.1	219	14	40	38	0	1
76.8	25.3	32.9	386	58	35	5	1	0
80.3	27.1	33.8	164	28.7	61.2	9.3	0.3	0.5
77.1	25.8	33.5	276	76.9	15.6	4.9	1.9	0.7
75.2	25.3	33.6	314	63	19	10	1	0
87.1	29.6	34	188	54.5	31.2	13.4	0.7	0.2
84.6	28.3	33.5	393	77.9	11.4	7.7	2.9	0.1
82.7	28.1	34	385	27	59	12.4	1.1	0.5
79.4	26.8	33.8	137	20	64	5	0	0
79.6	26.9	33.8	393	46.2	33.8	14.2	4.9	0.9
78.4	27.5	35	330	56.7	31	8.4	3.2	0.7
79.1	27.9	35.3	230	40.1	46.2	11.5	2	0.2
75.4	25.4	33.6	257	63.5	23.7	12	0.3	0.5
76.8	25.7	33.4	450	44	41	13	2	0
73.8	25.2	34.1	432	56	36.7	6.4	0.7	0.2
83	29.6	35.6	250	78.7	14	5.6	1.6	0.1
80.2	26.8	33.4	166	45.9	38.9	14.2	0	1
74.8	25	33.5	226	25.2	67.6	5.2	0.7	1.3
86.3	28.9	33.4	252	57	36	6	0	1

82.2	27.2	33.1	214	52.8	41.8	3.3	1.6	0.5
97.1	34.2	35.2	281	17.1	67	11.3	3.9	0.7
79.3	26	32.8	134	79	13.9	6.6	0.2	0.3
77.8	26.5	34	346	38	56	4	2	0
81.2	27.5	33.8	266	60.5	19.8	9.2	9.9	0.6
83.8	28.4	33.8	193	49.1	39.1	9.8	1.4	0.6
79.2	26.5	33.4	304	48.8	38.5	12.2	0.2	0.3
87.8	30.2	34.4	543	40	42	16	1	0
78.1	26.3	33.6	372	51	39	9	0	0
78.8	25.8	32.8	284	70	23.7	6.1	0	0.2
81.4	27.8	34.2	424	54.9	38	6.1	0.8	0.2
77.2	27.4	35.5	294	49	41	9	0	0
82.1	28.5	34.8	22	2	96	1	0	0
79.5	26.6	33.4	203	2	84	8	1	0
81.4	27.2	33.4	283	54.8	34.2	10.6	0	0.4
80.3	26.9	33.4	247	38	42	15	0	0
73.1	24.3	33.2	328	57.4	31.5	10.8	0.1	0.2
79.5	27.3	34.3	413	45.6	43.6	9	1.6	0.2
80.4	26.9	33.4	182	44	33	7	1	0
77.3	27.2	35.2	195	44.7	44.8	8.7	1.4	0.4
77.1	25.9	33.6	400	73.1	16	9	1.7	0.2
78.2	26.9	34.4	312	38.5	48	12.3	1.1	0.1
76	25.6	33.7	318	59	29	8	4	0
78.3	27	34.5	272	68	16.6	14.4	0.7	0.3
80	27.2	34	323	60.8	27.9	10.5	0.5	0.3
78.7	26.5	33.6	143	29	59	9	0	0
102.5	35.3	34.5	700	57	30	10	2	1
79.3	26.2	33	263	32.5	58.7	6.9	1.1	0.8
80.6	27.3	33.8	206	65	30	3	1	0
79.4	26.6	33.5	273	67	9	19	0	0
90.9	31.2	34.3	243	24.3	63.7	8.7	2.9	0.4
80.7	27.5	34.1	288	60.6	28.7	9.3	1.2	0.2
80.8	27.3	33.7	257	75	15	9	0	0
84.7	27.7	32.7	174	68.6	21.4	9.5	0	0.5
81.9	28.1	34.3	463	59.5	34.5	6	0	0
78.7	26	33	339	72.7	21.2	5.8	0.1	0.2
80.3	26.8	33.4	556	73.2	14.4	11.8	0.5	0.1
72	24.8	34.4	395	66	23	10	1	0
74.8	26.4	35.3	199	39.9	49.6	9.8	0.3	0.4
76.1	26.1	34.3	297	69.7	19.5	9.8	0.8	0.2
80.6	28.1	34.8	296	70.9	21.6	6.1	1.2	0.2
81.7	26.4	32.3	201	41	55	3	1	0
81.9	28.7	35.1	186	66.5	26.1	6.3	0.8	0.3
79.7	26.6	33.3	316	72.4	21.7	5.3	0	0.6
75.3	24.2	32.2	309	66.3	25.3	7.6	0.6	0.2
74.6	24.4	32.7	259	80	16	2	0	0

75.8	25.7	33.9	189	17	76	6	0	1
81.8	27.3	33.3	197	63.6	29.6	6.3	0	0.5
67.2	20.6	30.6	244	54	37.6	7.9	0.3	0.2
71	23.4	33	374	40	45	13	2	0
78.1	27.2	34.8	181	74.4	19	6.4	0	0.2
78	27.9	35.7	290	62.1	27.4	8.9	1.1	0.5
74.9	25.3	33.8	431	41	48	8	3	0
71.4	23.2	32.5	250	59.9	32.8	6.7	0.4	0.2
67.7	20.8	30.8	527	48	34	16	1	0
75.5	25.9	34.3	201	42	43.8	13	0.9	0.3
78.1	25	32	679	61.4	26.8	10.8	0.8	0.2
78.9	26.2	33.2	213	44.2	46.7	8.7	0.1	0.3
80	26	32.5	237	74	10	14	0	0
80.1	26.5	33.1	330	75.2	17.4	6.6	0.4	0.4
73	25.3	34.6	372	60	13	26	0	0
88	30.7	34.9	349	43.6	47.4	7.3	1.5	0.2
82.2	27.2	33.1	448	43.7	48.3	6.5	1.2	0.3
99.7	33.6	33.7	338	43.4	46.7	7.4	1.9	0.6
80.3	26.5	33.1	285	70.3	16.9	11.8	0.5	0.5
78.4	26.9	34.4	237	52.1	43.8	2.9	1	0.2
98.6	31.8	32.3	379	54	28	15	1	0
91.9	30.7	33.4	276	33.5	53.8	7.9	4.5	0.3
82.8	27.4	33.1	360	58.5	30.3	7.9	3	0.3
70.2	22.8	32.4	377	60.8	27.7	10.5	1	0
77.3	26.2	33.9	395	74	15.5	9	0.5	0
79.3	26.9	33.9	247	41.4	42	13.5	2.8	0.3
82	27.6	33.7	195	28	55	17	0	0
75.8	24.2	31.9	197	36.3	56.8	6.4	0	0.5
78.2	26.6	34	289	23	67	7	3	0
96.7	32.6	33.7	451	40.9	47.7	10.1	0.8	0.5
79.4	27.4	34.5	318	53.8	38.4	7	0.5	0.3
80.8	28.2	34.9	387	49	42.5	7.5	0.8	0.2
75.2	26	34.6	305	83	11	6	0	0
82.3	27	32.8	269	70.3	24.4	4.8	0.1	0.4
78.9	26.4	33.5	152	38	51	9	0	0
98.2	35.5	36.1	387	36	42	14	7	1
88.6	29.2	32.9	291	89	9	2	0	0
84.7	28.1	33.2	444	43	46	9	2	0
78.3	27.3	34.9	263	23	50	14	12	0
92.8	32.3	34.8	287	37	46	9	6	0
82.1	28.2	34.3	468	40.6	48.3	8.4	2.2	0.5
80.3	26.3	32.8	358	54.1	35.7	4.8	5.2	0.2
75.3	25.4	33.7	195	26	62	5	1	0
76.2	25.3	33.1	236	56	35	1	1	2
82.7	27.2	32.9	187	38	54	8	0	0
80.7	26.9	33.3	255	81	11	8	0	0

84.8	28.1	33.1	358	53.6	36.2	4.8	5	0.4
83.9	28.8	34.3	231	34.8	56.3	7.9	0.9	0.1
78.8	27.6	35	226	62.3	25.4	9.8	2.2	0.3
78.3	26.8	34.2	419	74	21	5	0	0
73.9	26.3	35.6	427	31	54	10	5	0
76.3	25.8	33.8	126	48.5	44	7.1	0.2	0.2
81.9	26.9	32.8	249	76	17.8	6.1	0	0.1
84.6	28.4	33.6	315	64	25	10.1	0.7	0.2
88.4	30.1	34	609	54	39	5	2	0
81.2	27.2	33.5	210	30.2	57.4	10.4	1.2	0.8
79.1	26.6	33.6	200	55	28	16	0	0
81.5	27.2	33.3	207	57.4	36.9	5.2	0.3	0.2
94.4	31.8	33.6	394	59.7	30.2	9.2	0.6	0.3
69.4	21.7	31.3	483	42.6	49.2	5.7	2.3	0.2
81.9	27.8	33.9	184	55	33	10	2	0
78.6	26.7	33.9	247	80.7	14	4.8	0.2	0.3
75.8	27.3	36	371	55.1	30.8	12.9	1	0.2
81	26.8	33.1	324	86.8	8.5	4.2	0.1	0.4
78.6	25.3	32.2	383	62	28	10	0	0
67.4	20.2	30.1	479	36.6	53.3	8.2	1.4	0.5
98.4	34	34.5	390	43	42	11	4	0
65.3	20	30.6	484	37.4	54	4.3	4.2	0.1
81.2	27.3	33.6	390	56	29	15	0	0
80.6	26.9	33.3	433	60.9	32.8	5.7	0.4	0.2
83	26.7	32.2	343	25	60	12	2	1
74.8	25	33.4	151	54	35.3	9.6	0.8	0.3
81.4	27.4	33.7	284	60	26	14	0	0
78.5	26.5	33.8	506	33	33	29	0	0
79.7	27.2	34.1	180	66.4	23.9	9.1	0.3	0.3
107.4	36.1	33.6	221	35	42	15	6	0
78.5	26.4	33.6	269	69	15	13	2	1
76.1	25.5	33.5	290	70	23	7	0	0
79.8	28	35.1	151	63.2	23.7	12.6	0.2	0.3
75.8	25.6	33.8	493	57	30	11	1	0
76.2	25.7	33.8	282	86.2	8.7	3.9	1.1	0.1
80.1	27.7	34.5	187	69	24	6.9	0	0.1
76.3	26	34.1	289	51	29	17	1	0
79.5	26.1	32.9	255	65	22.8	5.9	6.1	0.2
79	27.1	34.3	353	48.9	40.4	9	1.4	0.3
86.4	28.5	33	324	78	10	9	0	0
82.1	27.2	33.1	298	51.4	38.9	5.2	4.3	0.2
75.9	25.1	33.1	228	66	17	16	0	0
77.1	26.8	34.7	234	65	28	5	1	1
95.5	32.5	34.1	227	53	23	23	0	1
92.2	30.6	33.2	485	47.4	41.7	8.8	1.7	0.4
79.1	28	35.4	142	45.9	39.7	14.2	0	0.2

83.2	26.3	31.6	501	63	22	14	1	0
78	25.3	32.4	654	50.8	40.5	5.6	2.9	0.2
80.3	26.9	33.5	405	39.8	57	2.4	0.7	0.1
101	34.1	33.7	406	35.9	53.6	8.4	1.8	0.3
74.7	24.4	32.6	210	65	25	9	1	0
85.3	28	32.8	220	73	16.5	10.3	0	0.2
95	32.5	34.3	266	47.5	38.6	10.2	3.5	0.2
80.4	26.3	32.7	251	88.5	8	3.5	0	0
85.2	28.3	33.2	517	32	60	6	2	0
76.7	25.6	33.3	211	80.7	8.6	10.3	0.3	0.1
74	24.8	33.4	347	75	20	3	0	0
85.6	28	32.7	411	47.3	41.4	8.7	2.1	0.5
78	25.9	33.2	239	63.5	25	10.5	0.7	0.3
96	33.8	35.2	472	33.1	52.6	8.4	5.1	0.8
83.1	27.8	33.4	303	12.2	77.2	7.6	2.6	0.4
83.4	27.8	33.3	252	85.5	8.9	5.1	0.4	0.1
78.7	27.7	35.2	421	59.3	32.5	6.8	1.2	0.2
79.5	26.3	33	172	80.1	11.1	8.6	0.2	0
75.4	25.6	34	329	64	22	12	0	1
82	27.6	33.7	328	55.6	36.6	6.3	1.3	0.2
88.6	29.3	33	321	23.5	61.2	11.9	2.8	0.6
77.9	27.1	34.8	271	70.6	17.3	11.5	0.2	0.4
79	26.4	33.4	295	80.8	14	4.8	0.2	0.2
75.7	25.4	33.5	225	60	30	6	1	0
79.1	26.5	33.5	342	71.2	21.3	7.3	0.1	0.1
96.9	32.5	33.5	842	51.4	33.3	13.8	1.2	0.3
80.5	26.9	33.4	396	30.3	61.8	4.7	2.8	0.4
80.7	26.7	33.1	266	40	46	10	3	0
81	26.8	33.1	265	39.4	49.3	10.1	0.6	0.6
94.3	31.2	33.1	252	44.1	45.4	7.2	3.1	0.2
77.3	26.2	33.9	526	44.3	37.4	14.8	3.3	0.2
85.4	27.1	31.7	212	50	34	11	2	0
79.2	26.1	32.9	249	41.8	42.1	13.5	1.8	0.8
80.6	28.3	35.2	158	46.3	43.1	9.7	0.1	0.8
72.8	24	33	320	41.7	47.6	8.2	2.3	0.2
74.4	24.9	33.4	202	49	43	7	0	1
84.1	29.6	35.2	331	76.3	15.3	7.3	0.8	0.3
80.1	26.7	33.3	188	46	36	14	3	0
74.1	25.9	35	426	24	62	12	0	1
69.9	22.8	32.6	151	49.3	44.5	5.6	0.3	0.3
81.4	27.3	33.5	257	34.3	56.8	7.1	1.3	0.5
80.2	26.6	33.2	272	59.8	28.5	10.8	0.8	0.1
78.8	25.9	32.9	316	50.1	34.7	13.7	0.9	0.6
90.1	31.2	34.6	451	22	66	12	0	0
80.7	27.4	33.9	285	77.4	18.7	3.8	0	0.1
79.3	26.8	33.8	281	56	26	18	0	0

94.1	32.2	34.3	344	31	60	6	2	1
77.8	25.9	33.3	335	69.5	13.5	14	0	0
77	25	32.5	199	55	30	14	1	0
76.7	26.9	35.1	190	67.1	23.8	8.8	0.2	0.1
77.2	26	33.6	210	68	21	11	0	0
76.3	26.1	34.2	194	26.9	66.8	4.5	1.5	0.3
97.3	32.4	33.2	437	15	57	23	1	1
83.2	27.6	33.1	288	59	29	11	0	0
77.2	26.5	34.3	249	69	25	5	1	0
86.1	27.1	31.4	311	60	30	10	0	0
78	26.9	34.5	160	49	41.9	8.8	0	0.3
88.2	30.2	34.2	406	29.2	53.9	11.2	4.7	1
79.7	27.4	34.4	209	3	78	9	1	1
87.6	30.7	35	373	36.9	50.7	10.9	1.3	0.2
78.9	25.7	32.6	208	38	51	9.3	0.2	1.5
79	27.2	34.4	125	31.5	52	9	0	0
80	26.7	33.3	298	42.8	46.2	10.8	0.1	0.1
96.7	31.3	32.3	480	49	26	18	1	0
79	26.7	33.7	250	58.8	30.8	8.5	1.6	0.3
79.3	26.8	33.8	407	46.4	44.4	7.4	1.6	0.2
84.4	28.6	33.9	319	79.5	15.5	4.5	0	0.5
71.8	24.3	33.9	199	79	10	7	0	0
87.5	30.6	35	135	26.4	62	10.4	0.6	0.6
92.4	31.9	34.5	308	11.9	78.7	4.5	3.8	1.1
75.5	24.9	33	206	42	36	17	0	1
72.8	24	32.9	367	37.3	51.2	9.4	2	0.1
80.9	26.2	32.3	213	21	45	23	6	0
75	24.5	32.7	333	66.9	20.5	8.7	3.6	0.3
76.5	25	32.7	225	44.2	47.1	5.5	2.9	0.3
66.3	17.5	26.3	283	55	32	8	2	1
73.7	24.6	33.4	157	48.6	37	13.7	0.2	0.5
82	27.3	33.3	188	36.4	54.1	8.1	1.2	0.2
81.5	28.6	35.1	533	65.1	33	1.7	0.1	0.1
80.2	27.7	34.5	212	52.5	35.8	10.5	0.8	0.4
87.4	29.4	33.7	432	54	31	15	0	0
81	27.7	34.2	278	82.3	15.3	2.2	0.1	0.1
74.5	25.9	34.8	277	51	35	9	5	0
88.7	31.6	35.7	442	33	51	10	2	1
93.1	31.7	34.1	460	57.7	38.1	2.8	1.1	0.3
84.2	27.8	33	223	51.2	41.1	7	0.2	0.5
77.6	26.9	34.7	195	82	10	4	0	0
77.4	26.7	34.5	516	32.6	53.2	13	0.7	0.5
82.7	28.4	34.4	133	82.4	11.9	5.2	0.4	0.1
82.4	28.8	34.9	266	90	6	4	0	0
76.5	25.1	32.8	442	41	52.4	5	1.4	0.2
82.3	27	32.8	169	50	40	10	0	0

86.9	29.7	34.1	338	65.5	25.6	8	0.6	0.3
77.6	26.2	33.7	159	53.5	32.3	11.2	2.8	0.2
78	27.5	35.2	182	45.9	44.8	8.7	0.4	0.2
70.2	22.9	32.6	590	55	37	7	1	0
94.4	33.1	35.1	592	39	40	9	7	4
78.7	27	34.3	238	71.4	17.2	10.9	0.2	0.3
78.5	27.9	35.5	333	68	19.8	10.8	1.1	0.3
83.8	27.9	33.3	499	43.3	47	8.5	0.8	0.4
81.8	27.3	33.3	228	68	22	9	0	0
77.3	27.2	35.2	282	70.3	22.5	6.1	0.8	0.3
79.7	27.6	34.6	273	42.3	49.2	7.3	0.7	0.5
78.8	27.1	34.4	157	64.1	26.6	8.4	0.7	0.2
74.7	25.7	34.4	223	47.2	42.2	10.1	0	0.5
78.4	26.9	34.3	292	85.6	7.7	6.3	0.2	0.2
78.8	26.3	33.4	338	63	30.5	5	1.5	0
77.2	26.2	33.9	444	60.7	30.7	6.6	1.8	0.2
88.6	31.1	35.1	619	44	23	21	1	0
81.6	26.6	32.6	507	41.3	48.1	7.8	2.6	0.2
80.8	26.9	33.3	374	54	39	7	0	0
81.4	28.3	34.8	193	73.3	20.4	6	0.1	0.2
84.6	28.3	33.4	272	67.1	21	11.5	0.2	0.2
82.1	27.1	33.1	252	76	14	7	0	0
90.7	31.1	34.2	340	35	36	20	1	0
78.5	25.6	32.6	208	36	45	14	0	1
77.7	26.1	33.6	215	40.2	50.9	8.1	0.4	0.4
74.2	24	32.4	333	37.7	52.1	8.3	1.7	0.2
74.2	26.4	35.6	177	44.8	44.2	6.8	3.7	0.5
76.3	25.4	33.2	315	91	7	2	0	0
72.7	25	34.4	88	59	18	16	0	0
82.1	27.1	33.1	237	52.4	32.2	14.9	0.3	0.2
84.1	28.1	33.4	170	76	12	12	0	0
81.6	28.1	34.4	221	67.2	19.9	11.9	0.6	0.4
78	25.9	33.2	217	56.2	31.2	11.9	0.1	0.6
92.3	31.3	33.9	353	12.5	67.6	15.1	4.4	0.4
89.2	31.5	35.3	563	54.9	24.2	14.9	5.4	0.6
82.3	28.1	34.2	223	72.4	17.2	6.3	3.6	0.5
78.4	27.2	34.7	323	88	5	2	0	0
80.7	28.5	35.3	248	73.2	16.2	10.3	0.1	0.2
100.3	34.7	34.5	285	65.3	24.8	5.4	4.3	0.2
72.1	23.5	32.5	211	39	53.8	6.2	0.4	0.6
70.8	23.3	32.9	261	71.4	20.9	6	1.5	0.2
78.3	32	40.8	252	74	14	4	6	0
76.4	25.4	33.2	257	50.8	37.8	10.8	0.4	0.2
99.2	33.5	33.8	389	75	16	5	3	0
85.4	28.7	33.6	334	20	60	16	3	1
71	23.6	33.2	230	29.4	61.5	5.9	2.6	0.6

88.6	29.1	32.9	151	73.9	21.1	4.8	0	0.2
78.4	27.3	34.8	403	49.4	37.7	8.8	3.9	0.2
80.4	26.5	33	182	63.5	29.3	6.6	0.4	0.2
79.7	27.6	34.6	335	71.1	24.2	3.7	0.8	0.2
79.8	26.6	33.3	221	48.6	36.2	11.3	3.2	0.7
87.1	29.8	34.2	384	68	25	7	0	0
79.8	27.7	34.6	271	38	50	11	0	1
79.8	27.4	34.3	230	56.6	35.9	4.6	2.7	0.2
89.2	30.5	34.2	458	45.9	40.7	10.2	2.9	0.3
85.1	28.6	33.6	262	39.9	46.9	10.3	2.6	0.3
80.8	27.4	34	444	6	84	7	1	1
81.1	27.9	34.3	314	38.4	51.5	8.3	1.4	0.4
74	24.4	33	262	60	32.7	6.6	0.4	0.3
80	27.8	34.8	212	62.9	25	10.2	1.5	0.4
83.9	28	33.3	144	70	21.6	7.5	0.7	0.2
100.3	34.2	34.1	238	25	60	12	0	0
77.8	26.9	34.6	425	49	36.9	7.9	5.9	0.3
76.8	26	33.9	458	74.5	18.5	5	0	0
75.8	25.8	34	250	58.1	29.7	11.5	0.5	0.2
80.6	26.9	33.4	236	47.9	43.9	6.6	1.1	0.5
76.4	25.9	33.9	315	58	35	4	0	1
78.3	26.2	33.5	219	56	28.9	12.3	2.3	0.5
80.6	26.9	33.3	198	52	31	17	0	0
87.1	31	35.5	239	68.8	22.2	8.8	0.1	0.1
85	29.1	34.3	455	91	7	2	0	0
82.8	27.7	33.5	319	45.9	46.7	6.8	0.3	0.3
80.5	27.6	34.3	303	51	41	6	2	0
80.2	26.8	33.4	390	65	26	7	2	0
79.1	26.6	33.7	186	76.6	19.7	3	0.5	0.2
77.7	26.8	34.5	237	74	16.5	9	0.3	0.2
84.7	28.6	33.8	302	73	20	7	0	0
75.4	25.9	34.4	226	57.6	35.7	6.1	0.3	0.3
80.1	26.9	33.5	370	66.3	26.9	6.7	0	0.1
100.9	33.3	33	398	42	40	8	7	0
74	25.6	34.6	324	54	36	8	1	0
77	25.9	33.6	238	66.1	22.4	8.6	2.6	0.3
73.7	25.3	34.3	253	83	9	6	0	0
80.2	28.3	35.2	330	25	59	13	1	1
93.7	33.5	35.7	398	54.4	39.2	4.5	1.7	0.2
80	27.2	34	206	30.4	54.5	14.5	0.3	0.3
95.7	32.2	33.7	323	46	38	15	0	0
79.8	27.8	34.8	179	59.3	27.8	12.2	0.2	0.5
81.6	26.5	32.4	192	20	72	5	0	1
78	26.2	33.5	268	59	29	11	1	0
76.4	26.3	34.5	211	71	25	2.4	1.4	0.2
78.4	26.5	33.8	175	68	23	7	0	0

82.6	27.6	33.4	183	1	79	13	1	0
81.7	27.2	33.3	304	46.8	44.3	7	1.7	0.2
100.8	34.7	34.4	337	48	42	8	0	0
79.4	27.7	34.9	319	67.3	21	9.5	2.1	0.1
73.9	24.4	33	282	67	15	10	0	0
83.3	28.4	34.1	292	82	10	6	0	0
81.2	27.9	34.4	256	75	18	6	1	0
82.4	27.2	33	213	32	60	5	0	1
84.8	29.5	34.8	231	32	32	15	0	0
94.4	31.4	33.2	433	54.2	37.8	6.7	1.1	0.2
80.5	26.9	33.4	376	50	40	8	2	0
88.4	29.4	33.3	134	42.5	34.7	11.2	11.2	0.4
80.7	28.1	34.9	315	85.7	12.1	2.1	0	0.1
77.4	27.1	35	240	38.9	49.2	9.4	1.8	0.7
82.5	27.5	33.3	325	62.8	29.2	6.1	1.5	0.4
72.2	24	33.2	156	35.5	49.1	14.2	1	0.2
73.5	24.3	33	334	56.3	35.3	7.4	0.8	0.2
82.8	28.2	34.1	153	73.7	20	6.1	0	0.2
101.6	34.9	34.4	321	39.7	38.6	15.4	6	0.3
81.7	27.6	33.8	164	56.5	36.3	6.4	0.2	0.6
76.9	27.9	36.2	239	72	23	5	0	0
78.6	26.7	34	241	81.8	12.9	5.1	0	0.2
82	27.5	33.5	158	72.9	19.9	6.6	0	0.6
76.7	25.4	33.1	196	29.5	58.3	10.6	0.1	1.5
79.9	26.3	33	234	73	12	11	1	0
76.9	26.1	33.9	265	66.5	27.8	5.2	0.3	0.2
81.5	27.8	34.1	348	73.6	21	5.2	0.1	0.1
76.8	25.3	32.9	385	72.5	18	8	1	0.5
94	32	34	330	12	76	7	5	0
83.5	27.6	33	268	64.9	23.5	11.1	0.4	0.1
76.3	26	34.1	212	63.4	19.3	9.5	7.3	0.5
74.6	26	34.9	406	74.9	18.9	5.7	0.3	0.2
101.4	35.1	34.6	416	22	67	11	0	0
76.7	25.7	33.5	421	69	24	6	0	0
94.1	32.5	34.6	286	49	37	14	0	0
72.6	24.1	33.1	247	75	20.2	4.5	0.1	0.2
75.1	25.6	34.1	217	16.9	71.5	11.2	0.2	0.2
79.4	25.8	32.4	289	46.2	39.8	10.3	3.3	0.4
76.8	25.2	32.8	327	49.2	38.5	9.6	2.4	0.3
84.4	29.4	34.9	261	43.8	44.3	8.2	3.4	0.3
79.6	26.4	33.1	302	83.5	4.5	5	0	0
95.1	32.3	34	546	30	60	7	3	0
78	26.1	33.4	229	2	65	31	1	0
73.7	26.1	35.5	195	42	55	1	2	0
77.3	26.5	34.2	309	55.8	32.3	9.5	1.7	0.7
79.8	27.1	33.9	224	74.3	21	4.4	0.1	0.2

101.4	35.3	34.8	442	24	60	12	4	0
78.1	26.5	34	360	49.1	37.7	11.8	1.2	0.2
99	34.1	34.5	568	29	45	22	2	0
75.6	25.3	33.5	287	53	37.9	8.8	0.1	0.2
69.3	22.1	31.8	309	60.6	29.6	9.4	0.1	0.3
98.7	33.8	34.2	337	16	69	11	4	0
88.6	31	35	153	50.5	39.9	9.2	0	0.4
83.6	28.6	34.3	239	25	68	6	1	0
81.5	26.6	32.7	196	33.9	51.8	13.4	0.7	0.2
83.8	28.4	33.9	201	68.7	25	6.1	0.2	0
80.9	28.1	34.7	468	46	46	6	2	0
79.4	26.7	33.6	270	39	51	5	0	0
77.5	25.7	33.1	287	54.4	33.6	10.5	1.3	0.2
74.2	25.8	34.7	171	18.6	72.2	8.2	0.7	0.3
81.1	26.8	33	228	51.5	36.2	11.7	0.3	0.3
82.3	27.8	33.7	217	58	28	14	0	0
69.6	23.3	33.5	40	61.5	27	7.5	2.5	0
74.4	25.8	34.7	314	67.2	30.1	2.5	0.1	0.1
73.6	25.1	34.1	230	57	34	8	0	1
82.5	27.9	33.8	285	69.2	24.1	6.2	0.2	0.3
84.8	28.3	33.4	248	55.4	31.9	12	0.2	0.5
106.1	34.7	32.7	376	30.4	59.9	8.4	1	0.3
95.2	32.7	34.3	569	53.3	38.3	7.2	0.8	0.4
84.1	29.5	35.1	350	48.4	45.1	5.9	0.3	0.3
78.1	27	34.6	348	35	49	16	0	0
77.3	25.9	33.5	286	38	54	6	1	0
90.2	31.3	34.7	275	15.5	73.6	9.6	0.4	0.9
78.9	27	34.2	233	16.8	73.5	8	1.2	0.5
80.3	27.1	33.7	365	39	51	8.1	1.7	0.2
81.2	26.7	32.9	428	59.1	32.8	6.1	1.7	0.3
77.4	26.7	34.5	261	39	50.9	9.2	0.6	0.3
83.8	27.2	32.5	294	87.4	6.8	4.9	0.6	0.3
100	34.2	34.2	265	44	39	14	2	0
102.9	34.1	33.1	431	22.2	62.7	9.1	4.2	1.8
79.5	26.3	33.2	132	34.6	55.6	9.2	0.6	0
85.1	28.9	34	340	79.3	16.9	3.5	0.1	0.2
82.2	27.1	32.9	353	69.7	19.2	10	0.9	0.2
79.6	28.4	35.6	305	65	28	7	0	0
80.2	26.5	33	530	44.6	40.2	11.7	3	0.5
78.6	26.8	34.1	113	81.6	11.1	5.2	1.9	0.2
84.3	27.8	33	200	86.5	7.3	6	0.1	0.1
81.9	27.8	33.9	262	52	37	10	0	0
78.7	25.2	32	535	61.4	29.3	6.9	2.2	0.2
81.1	27.3	33.6	528	65	29.5	5.5	0	0
77.4	27.5	35.5	438	58.5	32	6	1	0
82.3	28	34	215	82.5	11.3	6.1	0	0.1

75.5	26.6	35.2	179	50	40.1	5.2	4.5	0.2
79.7	27.1	34	227	57.6	33.1	7.3	1.9	0.1
97.7	34	34.8	386	28.4	57.2	5.4	8.4	0.6
75.6	26	34.4	233	50.4	36.4	6.3	6.7	0.2
80.7	26.6	33	246	77	14	9	0	0
104.7	34.6	33	543	50	27	19	4	0
79	26.2	33.1	216	55.6	31.1	12.2	0.5	0.6
79.5	26.8	33.7	283	50	34	13	0	0
74.8	25.4	33.9	429	51.5	34	12.5	1.5	0
76.2	25.5	33.4	188	34.1	50.9	12.7	2.1	0.2
81.2	27.4	33.8	205	57.3	32	10.3	0.3	0.1
83.4	27.6	33.2	257	75.3	15.9	8.1	0.6	0.1
81.3	28.3	34.8	322	81.6	12.9	5.3	0	0.2
76.5	25.7	33.5	338	57	33	8	2	0
77	25.7	33.4	298	65.6	25.1	9	0.2	0.1
83.8	28.8	34.4	426	43.3	45.4	4.4	6.6	0.3
79.7	26.9	33.7	266	51.4	40.4	7.7	0.1	0.4
78.6	26.8	34	377	64.9	29.7	4.8	0.3	0.3
76.1	26.8	35.2	251	38	52	7	3	0
76.5	25.7	33.6	444	20.7	71.6	6	1	0.7
81.6	29.3	35.9	278	34.5	58.8	4.4	1.9	0.4
82.4	29.5	35.7	303	81	14	3	0	0
77.6	26.1	33.6	195	50.5	42.4	6.2	0.6	0.3
80.5	28.1	34.9	288	74.6	16.9	7.2	1.1	0.2
71.8	24.4	34	326	54.9	36.7	7.1	1.1	0.2
98.7	31.3	31.7	327	41	49	10	0	0
76.2	24.7	32.4	155	63.9	28.9	7	0.1	0.1
80.4	28.2	35.1	319	67.1	21.7	8.2	2.7	0.3
81.3	27.1	33.3	315	59	34	7	0	0
79.8	27.6	34.6	136	72.2	14.9	10.5	2.1	0.3
78.9	27.3	34.6	198	50.7	36.3	10.4	2.3	0.3
85.4	28.8	33.7	407	55.4	34.8	8.7	0.5	0.6
77.8	26.8	34.4	266	83	8	8	1	0
77.9	26.1	33.5	263	83.9	10.7	5.2	0.1	0.1
79.2	26.4	33.3	360	50	40	10	0	0
94.5	31.8	33.6	331	29	56	6	0	0
77.1	26.4	34.3	224	41.7	47	9.1	1.7	0.5
72.9	25.1	34.4	168	56.2	35.4	8.1	0.1	0.2
79.2	26.7	33.7	265	54	34.9	9.1	0.6	1.4
80.3	27.3	34	192	74	12.9	13	0	0.1
78.7	26.2	33.3	227	62.7	29	7.8	0.3	0.2
83.3	27.4	32.9	238	71.1	21.4	7.4	0	0.1
82	27	33	167	69.5	20.7	7.2	2.5	0.1
82.1	28.8	35.1	326	77	17	5	0	0
76.5	26	34.1	319	47.1	47	4.8	0.6	0.5
81.5	28.2	34.6	188	63	29	8	0	0

80.5	27.3	33.9	391	65.8	21.8	9.1	3	0.3
79.8	26.1	32.7	225	72.9	18.6	7.8	0.2	0.5
81	27.3	33.7	212	69	22	8	1	0
86.1	29.8	34.6	308	85.1	10.2	4	0.5	0.2
74.6	25.2	33.8	246	58.7	32.7	8.3	0.2	0.1
82.3	27.6	33.6	315	46.1	38.8	9	4.6	1.5
84.3	28.6	34	209	41.7	48	9.3	0.7	0.3
88.2	29.3	33.2	313	52	31	17	0	0
83.9	28.1	33.5	251	76.9	17.1	5.6	0.1	0.3
91.2	30.4	33.3	251	51.3	33.3	14.2	0.8	0.4
93.8	32.2	34.3	589	11	66	16	0	0
85.3	28.2	33.1	565	60.8	30.5	6.9	1.6	0.2
96.2	32.7	34	419	19	75	6	0	0
73.7	25.4	34.5	211	74	19	7	0	0
88.3	29.5	33.4	485	73	18	9	0	0
77.2	26.4	34.2	291	75.5	20.5	4	0	0
76.7	26.9	35.1	210	53.3	32.7	12.6	0.1	1.3
76.1	25.8	33.9	482	51.8	32.9	12.9	2.1	0.3
82.5	27.3	33.1	442	33	58	9	0	0
81	27.2	33.5	381	47.1	43.9	5.6	2.2	1.2
79.2	27.2	34.4	248	69.3	21.1	9.4	0.1	0.1
77.9	27.4	35.1	320	49	32	15	1	0
80.3	27	33.6	233	66.9	25.1	7.3	0.4	0.3
86.1	29.4	34.1	218	47.8	40.4	11.1	0	0.7
77.2	26.1	33.8	205	69.7	21.5	6.5	2.2	0.1
82.7	28.8	34.8	474	18.5	74.3	3.9	3.1	0.2
76	25.9	34.1	392	72	18	10	0	0
76.5	26.9	35.2	337	80.4	15.2	4.1	0.2	0.1
77	25	32.5	119	52.9	39.3	6.1	1.1	0.6
81.9	28	34.2	263	45.1	31.5	11.7	11.4	0.3
70.3	23.1	32.9	460	12	78	4	5	0
74.3	25.1	33.7	214	30	60	10	0	0
73.2	25.1	34.2	173	60.4	34.7	4.9	0	0
79.9	27.8	34.8	288	59.7	27.6	12.5	0	0.2
80.2	27.5	34.3	245	87.5	7.7	4.7	0	0.1
81.3	27.6	34	281	24	60	14	2	0
85.2	28.6	33.6	304	65	26	8	0	1
80.1	26.2	32.7	275	34	57.6	8	0	0.4
78.8	28.9	36.7	233	78.8	13.5	7.2	0.2	0.3
88	30.2	34.3	601	70	22	7	0	0
85.5	29.3	34.2	250	83.2	11	5.5	0.2	0.1
79.5	26.8	33.7	339	84.8	11	3	1	0.2
80.7	26.6	33	363	34	59	4	2	0
70.6	25	35.5	334	69	24.8	5.7	0.3	0.2
80.9	29	35.8	262	63.5	33.3	3	0	0.2
75.8	25.6	33.7	258	45.3	39.6	9	5.7	0.4

80	27.2	34	378	73.5	17.8	7.1	1.3	0.3
87.1	29.5	33.9	217	75.3	14.7	9.3	0.5	0.2
89.6	31.2	34.8	282	40.2	47.6	8.1	3.7	0.4
70.1	23.8	34	325	43.3	45.2	8.6	2.7	0.2
74.6	24.5	32.9	332	43	40	15	2	0
78.2	26.4	33.7	289	64.1	29	6.6	0.1	0.2
72.7	23.5	32.3	728	50.4	37.1	10.6	1.3	0.6
81.5	27.1	33.2	209	64.8	25	10	0	0.2
79.5	27.9	35.1	341	35.9	59.1	4.8	0.1	0.1
78	26.4	33.9	213	79	15	6	0	0
78.5	26.1	33.2	234	51.4	40.4	7.5	0.4	0.3
85.4	28.3	33.1	235	49	47	2	0	0
77.3	24.9	32.2	454	59.3	33.8	6.3	0.3	0.3
78.3	26	33.2	225	74	18	8	0	0
77.7	26.4	34	481	41.5	42.4	10.3	5.2	0.6
86.7	29.4	34	544	6	84	10	0	0
88.5	30.3	34.3	365	50.5	33.5	13.6	2.1	0.3
75.9	25.3	33.3	189	52	40	7	1	0
84.7	29.7	35	434	41.8	55.7	2.1	0.2	0.2
78.2	27.3	34.9	258	45	39	15	1	0
84.1	28.8	34.3	219	79.7	9.1	10.6	0.5	0.1
91.6	30.3	33	378	69.2	21.1	9	0.5	0.2
76.8	25.4	33.1	461	66.2	27.1	5.9	0.8	0
81.5	27.2	33.4	213	46.8	41.1	11	0.1	1
76.1	26.9	35.4	226	36.7	57.2	5	0.9	0.2
79.7	27.1	34	273	81.5	8	10	0	0
77.8	26.6	34.2	208	62	25	7	0	0
77.6	27.2	35.1	413	65	21	13	1	0
74.8	25.6	34.3	202	50	39	11	0	0
78.1	27	34.6	194	68.4	24.7	6.6	0.1	0.2
79.7	28.7	36	342	47.1	43.2	8.6	1	0.1
79.6	27	33.9	150	61.3	21.3	14.6	2.3	0.5
77.1	25.9	33.5	501	19	61	20	0	0
75.6	26.7	35.3	202	7	69.5	16.8	6.4	0.3
76.7	26.3	34.3	161	46.5	45.3	7.3	0.7	0.2
81.3	26.6	32.8	291	31.6	56.9	10.6	0.7	0.2
77.4	26.6	34.4	182	50.3	42.6	6.7	0.1	0.3
77.2	26.3	34.1	274	88.5	7.7	3.7	0	0.1
81.2	27	33.2	532	59.4	30.1	9.8	0.5	0.2
82.2	28.1	34.3	161	69.1	23.1	7.3	0	0.5
78.3	26	33.1	152	49	36	14	1	0
83.2	28.3	34	117	36.6	54.2	8	0.9	0.3
79	25.8	32.7	153	71.6	18.8	8.8	0.6	0.2
83.9	28.1	33.5	406	52.3	38.3	6.1	3.2	0.1
75.4	25.5	33.8	183	68.3	24.1	6.9	0.5	0.2
80.1	27.3	34.1	240	54	40	6	0	0

80	27.1	33.8	405	71.5	20.5	7.5	0	0.5
84.9	28.8	34	179	81	12	5	0	0
80.5	29	36.1	322	92	4	3	0	0
78.8	25.3	32.2	358	68	22	9	1	0
71.6	23.1	32.3	597	30.6	59.1	4.5	4.9	0.9
93.3	30.2	32.4	335	54.7	36.5	7.8	0.6	0.4
80.2	27.6	34.4	133	34	52	10	0	0
86.3	29.1	33.7	233	78.1	14.5	7.2	0	0.2
75.8	25.2	33.2	307	50.8	39.9	7.8	1.2	0.3
86.4	30.4	35.2	140	68.7	21.1	10.2	0	0
84.4	29.1	34.4	197	73	18	8.8	0.1	0.1
79.1	26.9	33.9	374	77	15	7	1	0
81.3	28	34.5	237	75.2	18.9	4.7	0.9	0.3
78	25.8	33.1	311	48	43	8	0	0
80.5	26.9	33.4	259	61	34	3	1	0
84.1	28.2	33.5	340	89.2	8.1	2.5	0.1	0.1
75.9	25	32.9	375	61.8	29.6	8.1	0.1	0.4
81.4	28	34.4	325	75	15	10	0	0
81	27.6	34.1	339	78.3	17.1	4.2	0.3	0.1
76.7	25.9	33.7	399	65	16	18.5	0	0
83.4	27.6	33.1	368	27.3	62.5	9	0.9	0.3
80	27.6	34.6	225	53.9	37.5	8.2	0.2	0.2
80	27.5	34.4	172	64.9	22.6	12.3	0.2	0
77.2	25.7	33.2	212	57.4	29.6	12.6	0.1	0.3
78.5	27	34.5	250	53	31	14	1	1
68.5	21.6	31.5	281	23	58	19	0	0
80.2	26.6	33.2	193	68	27	5	0	0
79.8	26.7	33.4	263	75.5	17.5	7	0	0
79.6	27.1	34	168	57.7	32.3	6.8	1.6	1.6
77.3	25.5	33	266	66.8	25.4	7.5	0.1	0.2
83.9	28.4	33.9	502	65	24	10	0	0
79	27.1	34.3	263	59	34.1	5	1.7	0.2
83.2	28	33.6	311	82	15.5	2.5	0	0
82.3	29.1	35.3	296	83.2	12.3	4.2	0.2	0.1
82	28	34.2	408	76.8	16.7	6.1	0.2	0.2
79.1	27.2	34.4	141	43.6	48.1	5	2.9	0.4
82.8	26.7	32.2	415	69	20	11	0	0
83.1	26.9	32.3	333	31	62	5	1	1
81.1	28.2	34.7	356	83.5	10.5	6	0	0
75	24.8	33	398	42	49	8	1	0
79.8	26.4	33	202	53.1	37.1	9.6	0	0.2
84.4	26	30.9	110	0.5	24.5	0.5	0	0
79.1	26.6	33.6	246	29.8	60.2	9.1	0.5	0.4
79.4	27.3	34.4	197	57	33	8	1	0
82.6	28	33.9	290	80.8	13.3	5.6	0.2	0.1
77.2	25.6	33.1	225	63	19	15	1	0

80	26.8	33.5	206	70.2	24.2	4.9	0.6	0.1
74.8	25.6	34.2	649	48	21	20	0	1
83.5	28.1	33.6	171	29	57	9	0	1
84	28.6	34	377	22.4	69.8	5.8	1.6	0.4
80.5	27	33.5	305	58	33	6	1	0
85.5	28.4	33.2	325	80.9	14.5	4.2	0.2	0.2
76.2	26.1	34.3	242	90.5	6.7	2.4	0.2	0.2
74.9	25.7	34.3	226	69	22.9	6.3	1.6	0.2
85.9	29.9	34.8	347	54.6	35.2	9.5	0.6	0.1
75.8	24.7	32.7	260	35.2	59.8	3.8	0.6	0.6
78.5	25.4	32.3	295	73	16	9	0	0
79.1	27.1	34.3	311	54	39.3	4.6	1.7	0.4
77.1	26.2	33.9	249	55.9	35.5	7	1.4	0.2
84.5	28.8	34.1	201	91.7	5.4	2.8	0	0.1
79.6	26.6	33.4	354	60.2	34.5	4.9	0.2	0.2
78.3	27.2	34.8	329	41.3	53.6	3.8	1.1	0.2
100.9	34.2	33.9	191	41	39	5	4	0
77.6	25.5	32.9	487	28.3	59.6	7.7	3.9	0.5
97.8	35.3	36.1	362	18	71	6	4	0
89.6	29.9	33.3	390	28	61	6	4	1
79.5	26.6	33.4	148	72.1	19.8	6.8	1.3	0
88.7	29	32.7	374	32.5	63.5	3.1	0.7	0.2
80.4	26.9	33.4	409	48.8	44.8	4.4	1.7	0.3
81.8	27.6	33.8	286	53.1	34.9	11.2	0.3	0.5
82.8	27.7	33.4	250	80.8	15.4	3.6	0.1	0.1
74.6	24.9	33.4	160	59.7	33	6.2	0.8	0.3
75.8	25.9	34.2	283	58	32	10	0	0
76.9	26.4	34.3	376	40.6	48.9	9.2	0.9	0.4
80.2	26.2	32.6	194	34	56.1	8.6	0.9	0.4
78.8	26.8	34	167	67.8	21.9	9.8	0.4	0.1
71.9	24	33.3	295	69.3	22.3	8.2	0	0.2
81.1	27.6	34.1	338	65.5	30.3	1.9	2	0.3
81.3	26.6	32.8	168	50.5	36.8	12.3	0.1	0.3
86.4	28.8	33.3	200	86.3	9.5	4	0	0.2
78.7	25.9	32.9	407	53	36	10	1	0
78.7	26.2	33.3	94	53.2	37.3	8.9	0.4	0.2
102.8	35.7	34.7	202	51	20	17	2.5	0
76.4	25.2	33	349	49	34	14	3	0
76.4	26.5	34.7	273	62.7	26.7	8.2	2.3	0.1
82.2	27.4	33.3	208	81.3	14.8	3.3	0.4	0.2
82.4	26.4	32	289	65.9	25.8	8.1	0.1	0.1
83	27.7	33.3	215	78	17	4	1	0
80.5	26.6	33	182	55	33	11	1	0
86.1	28	32.5	162	76.6	16.5	6.7	0	0.2
77.1	26.3	34.1	282	73	20	6	0	0
86.4	29.4	34.1	521	40	50	7	3	0

81.2	27.2	33.5	234	55	40	5	0	0
84.3	28.3	33.5	201	5	91	3	1	0
81.5	26.9	33	126	32	62	4	1	0
77.1	26.6	34.6	164	33.7	52.4	12.3	1.4	0.2
81.9	27.5	33.6	196	82.4	7.2	10.2	0.1	0.1
78.8	26.3	33.3	315	69	26	4	0	0
79.3	26.9	33.9	123	21	69	8	1	0
65.7	21.5	32.7	180	52	21	27	0	0
91.9	32.3	35.2	446	31	51	10	6	0
91.6	31	33.8	416	24.9	61.3	8.3	5	0.5
92.3	30.8	33.3	58	27	63	9	1	0
84.2	28.2	33.6	249	45.7	37.9	14.9	1.3	0.2
84	27.8	33.1	294	64.4	27.5	7.7	0.3	0.1
85.4	29.3	34.3	409	41	47	8	1	0
78	27.8	35.7	200	80.2	13.7	5.7	0.1	0.3
77.3	26.3	34	237	54	37	7	1	0
79.3	27.4	34.5	361	80.5	13.5	6	0	0
78.6	27.2	34.6	386	66.6	28.9	4.3	0	0.2
76.3	25.7	33.7	198	50.3	40.8	7.8	0.7	0.4
74.2	24.7	33.2	399	37.5	49.4	8.1	4.8	0.2
83.3	28	33.6	377	71.3	17.9	10.3	0.2	0.3
81.2	27.6	33.9	298	85.5	8.2	5.1	1.1	0.1
76.7	27	35.2	311	90	5	5	0	0
77.8	27.1	34.8	321	41.4	52.2	6	0.3	0.1
76	26	34.2	240	60	32	4	1	0
82.1	28.8	35.1	256	35	42	18	0	0
80.4	28.3	35.2	341	54.9	35.7	7	2.2	0.2
74.9	26.3	35.2	243	86	10	4	0	0
77.8	26.9	34.6	213	39	51	8	2	0
82.2	28.4	34.6	176	82.3	6.3	10.2	1	0.2
75.9	25.6	33.7	212	78.4	13.7	7.8	0	0.1
93.9	32	34.1	425	53.1	36.3	9.7	0.5	0.4
77	26.9	34.9	219	66.3	21.4	12.1	0	0.2
77.3	26.7	34.5	272	48	36	6	0	0
72.9	24.9	34.2	227	23	50	20	3	0
85.7	30.1	35.1	411	52.6	35	11	1.1	0.3
68.7	23.6	34.4	285	17	61	22	0	0
80.1	27.1	33.8	249	65.3	27.2	7	0.3	0.2
72.4	25.2	34.8	162	86	6	7	0	0
91.2	31.9	35	269	37.8	52.6	5.5	4	0.1
78.4	26.8	34.2	158	27.4	67.6	4.8	0	0.2
79.8	27.4	34.4	175	78	9	11	0	0
77.3	26.8	34.7	301	68.4	21.9	8.6	0.6	0.5
75.3	25.8	34.2	406	46.2	42.9	9.2	1.2	0.5
100.9	33.4	33.1	401	30	59	7	4	0
66.2	21.1	31.8	218	61	29	8	1	0

83	29.5	35.5	552	73	22	5	0	0
84.9	30.1	35.4	123	60	21	19	0	0
82.3	26.8	32.5	378	64.9	23.5	10.6	0.9	0.1
82.1	27.5	33.5	299	56	33	7	0	0
83	28.5	34.3	193	65	21	13	0	0
76.1	26	34.2	245	67	22.5	9.8	0.5	0.2
80.8	28.1	34.8	243	72.9	13.3	13	0.5	0.3
78.4	26.9	34.3	151	88.3	8.3	2.6	0.5	0.3
78.6	26.4	33.6	251	65	30	5	0	0
77.7	26.2	33.7	301	50	35	15	0	0
83.2	27.3	32.8	212	55.8	33.9	7.7	2.5	0.1
81.5	28.9	35.5	252	81.1	11	7.7	0.1	0.1
85.5	28.8	33.7	229	60.2	29	7.8	2.6	0.4
78.3	26.8	34.2	184	25.4	67.3	6.4	0.5	0.4
82	27.1	33	394	42	45	13	0	0
80.7	27.8	34.4	242	54.8	34	9.9	0.8	0.5
84.4	28.1	33.3	727	62	27.5	10.5	0	0
76.7	25.4	33.1	355	60	22	11	5	0
83.1	28.4	34.1	143	60	13	16	0	0
84.1	27.9	33.1	394	32.7	55.1	8.8	2.7	0.7
75.2	25.3	33.6	203	59	37	4	0	0
77.9	26.4	33.9	182	40.8	50.1	8.3	0.4	0.4
83.4	28.3	33.9	40	49	43	8	0	0
91.1	32.4	35.5	524	81.2	15.2	3.2	0.3	0.1
79.5	26.7	33.6	320	73	19	7	0	0
77.9	26.7	34.3	186	54	34.3	11.5	0	0.2
84	28.8	34.3	317	49.4	42	6.6	1.8	0.2
80.4	27.7	34.4	330	76.5	16.5	7	0	0
76.7	25.4	33.1	425	37.4	50.7	8	2.6	1.3
81.3	27.7	34	172	30	60	10	0	0
80.3	28.5	35.4	444	18	74	6	2	0
80.7	27.1	33.6	208	71.2	24.4	3	0.5	0.9
70.6	23.7	33.5	142	25.4	68.3	5.3	0.4	0.6
78.6	26.5	33.7	274	74.3	19.7	5.6	0.1	0.3
78.6	26.4	33.6	189	80.1	10.6	7.9	1.1	0.3
74.1	25	33.7	378	61	28	11	0	0
78	26.5	34	233	67	19	11	3	0
74.7	25.7	34.5	295	47	39	11	3	0
77.8	25.9	33.3	149	18	41	31	0	0
82	27.5	33.6	371	57	24	18	1	0
97.7	34.1	34.9	443	23	60	13	1	3
79.5	26.1	32.9	136	57.9	32.6	8.9	0.3	0.3
77.2	25.9	33.6	239	81.8	11.4	4	2.8	0
78.4	26.7	34.1	187	67	21	8	2	0
79.3	27.3	34.4	308	38	39	14	1	0
80.2	27.2	33.9	390	43	44.3	10.2	2.4	0.1

80.3	27.6	34.4	190	46.9	41.9	11.2	0	0
80.1	27.5	34.3	100	7	8.5	2	0	0
78	26.2	33.6	285	69	21	9	0	0
77.1	25.9	33.6	296	45	44	10	1	0
79.1	27.7	34.9	312	85	10.7	4.1	0.1	0.1
82.3	27.6	33.5	288	65	30	5	0	0
80.8	27.6	34.2	245	80	16	4	0	0
84.5	28	33.2	269	55	32	12	1	0
82.8	27.2	32.8	302	18	72	7	3	0
78	27.4	35.1	204	41	40	9	0	1
77.9	26.3	33.8	347	28	55	11	0	1
82	28.4	34.6	202	69.7	17.6	11.6	0.9	0.2
71.3	23.6	33.1	270	55	35	8	0	0
81.8	27.6	33.8	274	73	19	7	1	0
77.2	26.1	33.8	351	68	26	5.1	0.8	0.1
78	26.5	34	301	49.8	37.8	10.8	1.2	0.4
85.5	28.3	33.1	191	26	62	7	2	0
83.3	28.3	34	209	69.5	21	9	0.5	0
76.8	27.1	35.3	268	87.2	6.7	5.9	0.1	0.1
80.5	27.8	34.5	219	68	24	6	0	0
79.4	28.5	35.9	144	73.9	21	4.3	0.6	0.2
70.3	22.9	32.6	330	53	37	10	0	0
81.8	26.7	32.7	198	67.5	26.9	5.5	0	0.1
82	28.8	35.1	240	55.2	34.4	7.8	2.3	0.3
88.7	30.3	34.2	430	62	31	4	1	0
76.9	26	33.8	395	51	32	16.5	0	0
78.5	25.9	32.9	237	59.3	32.8	7.2	0.5	0.2
79	26.5	33.5	149	73.4	17.8	8.5	0.2	0.1
78	27.1	34.7	180	42.5	45.9	8	3.3	0.3
82.5	26.6	32.3	158	43.9	46.6	8.6	0.2	0.7
77.4	26.6	34.4	139	16	68	14	0	0
77.8	26.7	34.3	191	53.5	35.1	10.8	0.3	0.3
88	28.7	32.6	197	92.2	5.7	1.9	0.1	0.1
78.2	27.3	34.9	247	69.1	26.4	4.3	0	0.2
79.3	27.9	35.2	272	83.7	12.3	3.8	0.1	0.1
79.7	26.8	33.7	253	83	13	4	0	0
79.1	27.7	34.9	306	38	49	8	3	1
80.5	27.3	33.9	269	62.2	27.3	9.9	0.4	0.2
79.1	26.4	33.3	237	71	22	3	0	0
80.9	26.3	32.5	132	54.6	34.9	10.4	0	0.1
80.3	27.4	34.1	350	75	15	6	1	0
79.5	26.6	33.4	225	42.9	39.7	10.3	6.2	0.9
77.3	26	33.6	180	58.7	30.3	10.5	0.5	0
75.1	24.9	33.1	257	61	27	10	0	0
80	27.8	34.8	176	61.4	28.4	9.4	0.2	0.6
78.7	27.2	34.5	396	86.5	8.8	4.5	0	0.2

79.1	26.7	33.8	311	78	16	4	1	1
85.1	28.1	33	203	80.8	13.8	5	0.1	0.3
77	26.2	34.1	377	59.3	39.5	0.8	0.3	0.1
80.9	27.5	34	229	65	25.8	8.8	0.1	0.3
82.9	28.3	34.1	243	60	24	16	0	0
80	27	33.7	232	77	15	8	0	0
82.4	28	34	201	43	39	14	0	0
80.8	26	32.2	357	53	20	19	1	0
74.4	24.6	33.1	325	38.7	52.9	6.1	2.1	0.2
80.7	27.1	33.6	250	53	35	10	1	1
102.4	35.9	35.1	269	76.4	11.5	7.9	3.7	0.5
77.4	26.7	34.5	248	48.4	47.6	3.6	0.2	0.2
80.5	26.2	32.5	308	38	50	12	0	0
84.1	28.8	34.2	306	61	34	4	1	0
81.5	27.6	33.8	336	85.6	6.5	7.2	0.5	0.2
79.7	27.1	34	110	70	20.3	9.3	0.2	0.2
79.4	26.4	33.2	180	5	60	33	1	0
77.2	26.5	34.3	302	81.3	12.4	6.1	0.1	0.1
77.9	27.6	35.4	237	69.3	19	9.5	2	0.2
78.5	25.4	32.3	259	61	29	10	0	0
79	26.8	34	340	60	32.7	6.4	0.8	0.1
76	26.2	34.5	226	86.9	8.9	3.7	0.4	0.1
82.5	27.3	33	165	49	42	4	5	0
77.2	25.2	32.7	149	44.7	50.5	4.6	0	0.2
75.1	25.3	33.7	250	54	38	7	0	0
75.7	26.5	34.9	269	54.2	33	12.3	0.2	0.3
74.7	25.5	34.1	601	73.5	22.5	4	0	0
78.1	27	34.6	321	58	36	6	0	0
99.2	32.3	32.5	575	32	53	13	0	1
83	29.2	35.2	335	68.6	22.8	8.4	0	0.2
78.6	26.4	33.6	160	39	37	22	0	0
83.5	27.8	33.3	252	41	48	7	2	0
78.2	26.9	34.3	294	68	25	7	0	0
79.4	26.9	33.8	230	34.7	54.6	7.8	1.9	1
76.1	26.2	34.4	233	68	23	3	0	0
74.6	24.6	33	215	86.2	7.8	5.3	0.6	0.1
75.5	26.6	35.3	235	78.3	16.7	4.5	0.4	0.1
79.3	26.9	34	218	41	52	7	0	0
79.4	27.1	34.1	200	43	43	7	0	0
82.7	27.8	33.6	147	32	60	8	0	0
79.7	27	33.8	169	75.9	12	9.6	2.4	0.1
81.4	27.6	33.9	269	67.2	21.8	10.5	0.3	0.2
82.1	27.4	33.3	171	27.8	63.3	8.5	0.2	0.2
74.4	25.1	33.7	323	48	38	12	1	1
81.7	29	35.4	179	32.9	56.5	10.2	0	0.4
73	25.1	34.3	377	44.5	41.5	8.9	5	0.1

76.5	27	35.3	190	48.9	40	10.6	0.3	0.2
75.3	26	34.5	245	49.1	41.9	8.2	0.4	0.4
73.5	25.2	34.3	229	72.3	19.2	7.9	0.3	0.3
76.9	27.3	35.4	298	62	29.1	8.7	0	0.2
71.5	23.2	32.5	186	69	15	14	0	0
78.4	26.7	34.1	402	61.8	28.2	5.8	3.7	0.5
92	31.8	34.5	322	18	61	18	3	0
80.1	26.5	33.1	280	61.5	27	10.5	0.5	0
79.1	27.2	34.4	123	62.8	27.5	7.5	2	0.2
75.6	25.5	33.7	252	81	11	3	0	0
79.9	27.4	34.2	257	77.6	16.4	5.8	0	0.2
84.9	28.6	33.7	243	68	27	5	0	0
81.5	27.3	33.5	200	83.6	10.3	5.9	0.1	0.1
78.8	27.1	34.4	197	70.7	20.6	7.3	0.7	0.7
83.9	27.9	33.2	237	64	26	9	1	0
81.2	28.4	35	252	65.2	29.6	4.1	0.6	0.5
82.3	27.5	33.4	280	66.9	25.5	6.9	0.3	0.4
77.4	26.5	34.2	179	74.1	15.2	9.5	1	0.2
76	24.9	32.7	152	58.8	33.2	6.9	0.9	0.2
79.9	27	33.7	304	61.6	32.3	4.6	1.4	0.1
77.8	26.2	33.7	306	66.3	28.3	4.5	0.8	0.1
80.2	27.7	34.6	285	85.1	10.5	4	0.1	0.3
78.5	27.7	35.3	184	49	30	21	0	0
76.3	27.1	35.5	348	68.2	20.9	9.4	1.3	0.2
89.8	30.9	34.4	427	17.2	64.6	14.4	3.5	0.3
78.2	26.7	34.1	369	72.8	19.3	7.5	0.2	0.2
65.6	20.3	31	219	28	60	9	0	0
74.6	26	34.9	304	56.4	33.5	8.7	1.2	0.2
79	27.5	34.8	168	77	9	13	0	0
80.6	26.9	33.3	322	77.2	17.5	5.1	0.1	0.1
76.2	26.6	34.9	244	53	34	11	2	0
70.9	23.5	33.2	345	71.6	20.1	7.4	0.6	0.3
80.2	27.3	34.1	234	66	25.4	5.8	2.6	0.2
76.1	26.8	35.3	301	24.3	62.7	5.9	6.6	0.5
77.9	27.3	35.1	219	92.1	3.4	4.4	0	0.1
89.2	29.7	33.3	407	44	47.9	7.2	0.7	0.2
74.1	24.2	32.7	207	64	20	14	1	1
80.4	26	32.4	510	66	27.7	6.1	0	0.2
74.9	26.3	35.1	224	48.8	38.5	12	0.5	0.2
84.6	27.6	32.7	395	56	31	13	0	0
95	33.5	35.3	246	34.5	56.4	6.8	2	0.3
75	26	34.7	293	56.3	38.9	3.8	0.8	0.2
82.5	27.8	33.7	171	79	15	5	0	0
79.6	26.5	33.3	160	72	22.6	4.6	0.6	0.2
79.2	26.2	33	240	50	31	16	2	1
80.4	27.4	34.1	161	48	41	11	0	0

80.5	27.8	34.5	319	65.2	24.3	10.3	0.1	0.1
80.3	27.2	33.9	209	58.3	31.7	8.8	0.6	0.6
79.4	28.3	35.7	369	24.4	69.4	4.9	1.2	0.1
112.4	39.4	35	247	50	28	16	2	0
77.5	27.2	35.2	218	35.2	53.6	9.6	0.8	0.8
76.9	26.3	34.2	87	37.7	52.4	9.9	0	0
77.3	25.6	33.1	309	78	15	6	0	0
93.9	31.4	33.4	156	70.2	23.5	5.9	0.2	0.2
81.3	27.8	34.2	255	40.6	51	7.6	0.2	0.6
80	27.1	33.9	263	57.2	37.2	5.2	0.3	0.1
80.5	28.4	35.3	237	64.5	22.6	10.4	1.9	0.6
77.7	26.7	34.3	212	56.8	28.7	7	7.4	0.1
77.6	26.7	34.5	429	77.3	15	7.5	0.1	0.1
80.8	27.5	34.1	269	43	42	10	0	0
80	27	33.8	246	87.1	7.9	3.7	1.2	0.1
77.9	27.1	34.7	188	64.1	26.5	8.9	0.3	0.2
84.7	28.9	34.1	178	81.9	10.7	7	0.2	0.2
71.5	24.1	33.8	245	77.7	12.6	9.2	0.3	0.2
84.2	28.9	34.3	235	72	23	5	0	0
82.9	28.3	34.2	222	79.3	15.1	5	0.5	0.1
77.1	26.2	34	249	62	23	14	0	1
80	27.8	34.8	235	63.5	24.9	11.3	0	0.3
80.4	27.6	34.4	140	37.5	53.3	7.7	1.2	0.3
80.4	25.6	31.9	187	58.9	33.4	7.2	0.2	0.3
79.5	26.4	33.2	159	34.5	60.7	3.6	1	0.2
82.3	27.6	33.5	160	43.3	50.1	6	0	0.6
83	27.9	33.6	383	63	32	5	0	0
81.8	27	33	229	57.3	37	5.2	0.1	0.4
85.3	29	34	198	57.3	31.4	10.3	0.5	0.5
65.4	20.7	31.7	149	78	9	5	0	0
81.3	28	34.4	95	44	54	2	0	0
82.2	27	32.9	320	53	35	9	1	0
79.7	28.1	35.3	176	89	7	4	0	0
75.8	26.3	34.6	179	62	32	6	0	0
87.1	29	33.3	192	66.9	26.1	6.5	0.3	0.2
80.6	27.9	34.6	185	42	49	8	0	1
77.5	26.2	33.8	259	35.9	60.5	3.2	0.1	0.3
76.1	26.2	34.5	147	71.1	21.7	6.7	0.2	0.3
78.6	27.5	34.9	265	90.3	7.6	2	0	0.1
80.6	27.2	33.7	199	71.8	18.7	9.2	0	0.3
84.7	28.8	34.1	331	82.6	10.7	6.6	0	0.1
83.9	28.8	34.3	470	13	80	5	1	0
80.9	27.1	33.5	264	63.1	28.2	8.2	0	0.5
81.3	27.6	33.9	203	70.6	18.5	10.5	0.3	0.1
81.8	27.6	33.8	299	51.8	33	14.8	0.2	0.2
71.5	24.3	34	234	85	6.5	5.5	0	0

74.2	25.4	34.3	285	71.9	18.3	9.6	0.1	0.1
81.1	27.7	34.2	232	60.3	33.2	5.1	0.8	0.6
79.1	27	34.1	159	74.9	20	4.6	0.4	0.1
77.1	27.7	36	308	86	6.5	7.5	0	0
78.2	26.1	33.3	165	73.8	16.9	8.2	0.9	0.2
79.6	27	33.9	344	61.5	32.5	6	0	0
80.7	27.7	34.3	166	21.1	70	8.2	0.2	0.5
97.4	34	34.9	343	20.2	66.3	5.4	7.4	0.7
75.7	26.2	34.6	329	49.5	41	7.7	1.5	0.3
83	27.7	33.4	386	60.8	28	9.9	0.8	0.5
86	29.9	34.8	243	32.3	51.4	14.9	0.5	0.9
75.6	26.2	34.6	295	69	15	7	0	0
89.1	29.6	33.2	274	55.9	27.3	14.2	2.1	0.5
78.8	25.6	32.5	495	61	31	6	1	0.5
72	23.5	32.7	138	68.2	24.2	7.4	0	0.2
76.6	25.1	32.7	167	66	19	10	0	0
79.7	26.9	33.8	273	33.9	52.2	10.6	2.8	0.5
76	26.7	35.2	200	44.4	47	8.1	0.2	0.3
75.3	26.1	34.6	309	61	19	13	0	0
82	27.5	33.5	211	55.5	34.1	6.6	3.1	0.7
82.4	28.1	34.1	131	64.8	26.2	8	0.8	0.2
83.5	28.1	33.6	219	81.3	8.2	10.4	0	0.1
78	26.7	34.2	160	75.5	16.6	7.7	0	0.2
82.7	28.3	34.2	121	55	29.6	14.2	0.6	0.6
83.6	28.2	33.7	135	37.5	57	5.3	0	0.2
79.1	26.4	33.3	259	65.2	25.6	8.7	0.3	0.2
96.3	32.6	33.9	421	59	30	10	1	0
86.2	29.3	34	354	51	38.1	7.1	3.4	0.4
84.3	27.6	32.7	129	48	43	8	0	1
82.4	26.9	32.7	237	74.9	19.5	5.2	0.3	0.1
77	25.3	32.8	363	58.7	32.6	7.1	1.3	0.3
95.8	33.3	34.8	658	28.4	53.7	9.1	8.3	0.5
81.2	27.4	33.8	285	63	23	10	1	0
77.1	25.8	33.4	304	56.4	37.6	5.7	0.1	0.2
81.9	27.4	33.4	156	51.4	40.4	7.8	0.2	0.2
79.6	27.3	34.3	246	57.8	31.4	10.5	0.1	0.2
73.1	25.9	35.4	362	67.5	26	6.5	0	0
79.4	27	34	294	49	36	8	0	1
78.3	27.6	35.2	164	69.4	19.9	7.1	3.3	0.3
83.6	28.2	33.8	340	75.6	16.2	7.5	0.6	0.1
70.2	21.8	31	446	43.2	51.1	3.6	2	0.1
83	27.7	33.3	131	79.6	14.7	5.3	0.1	0.3
92.5	31	33.5	459	21	67.3	6.7	4.4	0.6
83.5	29.1	34.8	386	54	37	6	2	0
78.6	26.8	34	258	50	29	19	2	0
83.2	27.5	33.1	193	72	9	11	8	0

78.6	26.8	34.1	287	84.6	7.3	7.8	0.2	0.1
89.1	31.4	35.2	307	64	29.9	4.7	1	0.4
76	26.3	34.7	257	65.1	22.7	11.5	0.4	0.3
78.8	26.6	33.7	168	49	37	13	0	0
78	26.3	33.8	277	68	28.3	2.8	0.8	0.1
83.4	28.9	34.7	202	48.1	39	12.8	0	0.1
79.4	26.6	33.4	257	50.5	42.1	5.8	1	0.6
79.2	26.2	33	191	59.8	33.2	7	0	0
75.1	25.3	33.7	205	43	48	3	6	0
96.2	31.3	32.6	459	31	34	23	2	0
86.5	29	33.5	267	30.1	57.3	7.7	4.4	0.5
76.5	27	35.3	192	49	35	12	1	1
78.4	27.2	34.7	168	72	19	9	0	0
65.3	21.7	33.2	190	39	53	8	0	0
75.1	27.3	36.3	261	91	3	6	0	0
99	33.7	34.1	582	59	27	11	1	0
76.6	24.8	32.4	355	66.3	24.7	8.4	0.2	0.4
79.6	26.6	33.4	206	67	31.4	1.3	0	0.3
79.2	27	34.1	320	63.8	28	6.8	1.2	0.2
77	26.9	34.9	181	55	33.8	9.7	1.1	0.4
74.9	24.7	33	316	71	13	12	0	0
73.8	24.1	32.7	186	56.3	23.4	12.7	7.1	0.5
78.3	27.3	34.9	274	75.7	15.6	8.1	0.4	0.2
74.5	24.5	32.9	400	37	50	11	2	0
82.4	27.7	33.6	234	70.8	20.9	8.1	0.1	0.1
67.7	22.8	33.7	188	54	21	17	0	1
83.1	27.3	32.9	262	57	29	8	3	0
103.3	34.6	33.5	203	55.1	35.2	6	2.9	0.8
79.4	26.3	33.2	208	58	29.7	11.2	0.8	0.3
80.9	27.3	33.7	196	59	31.9	7.8	1	0.3
75.5	26.8	35.5	250	30	59	7	3	0
77.9	25.5	32.7	274	49	39	10	0	0
80.1	27.4	34.2	247	75	16.3	8.2	0.4	0.1
77	24.9	32.4	302	39.2	52.3	7	1.3	0.2
87.6	29.2	33.3	166	64.2	29	6.8	0	0
82	27.6	33.6	343	48	40	6	1	0
77.3	27.5	35.6	214	79.2	14.9	5.8	0	0.1
81.5	27.7	34	235	79.4	11.5	8.4	0.3	0.4
76.9	27.1	35.2	87	55.8	34.7	8.9	0	0.6
88.4	29.1	32.9	197	42	46	8	1	0
72.3	25	34.6	245	76.2	14.5	8.2	1	0.1
77	25.7	33.4	214	65	30	4	0	1
76.9	26.1	34	200	57.2	34.9	6.9	0.6	0.4
78.3	28.3	36.1	402	50.2	40.6	8.1	1	0.1
80.8	27	33.4	294	72	18.7	8.8	0.4	0.1
83.7	29.3	35	197	53.7	37.7	6.5	1.8	0.3

78.3	27.3	34.8	378	75	19	6	0	0
73.4	24.4	33.2	379	74.3	20.6	3.3	1.6	0.2
78.9	26.5	33.6	159	46.7	38.3	14.8	0	0.2
77.8	27.3	35	154	45.2	48.8	5.4	0.3	0.3
81.2	27.1	33.3	211	70	12	18	0	0
73.5	24.6	33.5	326	63.8	24.7	10.7	0.7	0.1
80.6	27.1	33.6	162	47.4	42.6	9.9	0	0.1
82.2	27.9	33.9	286	82.6	10.3	6.8	0.1	0.2
78.6	27	34.3	322	50.6	44	4.9	0.1	0.4
73.3	25	34	256	90.6	6.3	3	0	0.1
78.2	26.4	33.7	161	32.8	60.9	6	0.1	0.2
78.7	27.1	34.4	199	39.8	47.5	10.6	1.4	0.7
89.4	30.7	34.4	207	47.9	43.4	8.3	0.2	0.2
79.7	26.4	33.1	364	28.5	60.7	6.4	3.5	0.9
79.9	26.6	33.3	249	86	10	2	0	0
77.9	26.9	34.5	172	64	26	8	2	0
83.4	29	34.8	278	63	29.7	6.9	0	0.4
79.5	27.5	34.7	295	39	48	9	0	0
81.7	26.5	32.5	229	67.3	24.9	6.6	1.1	0.1
78.6	26.6	33.8	139	44.9	45.4	8.1	1.1	0.5
75.8	26.9	35.5	302	66.9	23.8	8.3	0.5	0.5
73.7	24.8	33.6	243	74.3	15.4	9.3	0.9	0.1
82	28.4	34.6	335	53.9	32.4	11.1	2.4	0.2
78.9	26.5	33.6	7	1	5.5	0.5	0	0
81.3	27.4	33.8	383	64	22	6	3	0
78	25.2	32.3	286	41.6	44.5	12.2	0.9	0.8
82.9	27.4	33.1	344	48	43	6	3	0
76.5	25.5	33.3	292	41.6	51.5	6.5	0.2	0.2
80.2	29	36.1	68	58	34.5	7	0	0
82.2	27.6	33.5	221	59	31	8	0	0
83.1	28	33.7	195	30.9	58.4	10.1	0.4	0.2
78.6	26.4	33.7	319	50.9	42.8	4.3	1.8	0.2
78.3	26.5	33.9	148	54.3	33.7	8.9	2.1	1
83.5	28.3	34	106	78	11	7	0	0
89.1	31.3	35.1	501	50.6	40.5	7	1.6	0.3
95.6	33.3	34.9	324	48.3	41.6	5.9	3.7	0.5
82.5	27.4	33.2	334	81	10.5	6.5	0.5	0
74.3	25.3	34	238	55	35	10	0	0
82	26.6	32.4	235	73	22	4	1	0
75.9	26.2	34.5	261	53.3	33.4	11.9	1.2	0.2
77.3	24.6	31.8	522	59	33	8	0	0
66.9	20.6	30.8	156	41.3	46.9	11.3	0	0.5
89.8	29.7	33.1	374	45	32	21	1	0
75.8	25.8	34.1	207	68	21	9	0	0
77.2	26.1	33.8	287	89.5	4.5	5	0	0
75.3	25.3	33.6	287	63	23	12	0	0

73.5	25.2	34.2	346	52.5	34.1	12.1	1	0.3
75.4	26	34.5	292	48	46	4	1	0
76.3	25.9	33.9	106	63	21	12	0	0
74.5	25.3	33.9	98	38.4	52.7	8.5	0	0.4
87.2	29.3	33.7	219	27.6	60	11.9	0.2	0.3
77.6	27.1	34.8	273	82.4	13.1	4.3	0	0.2
82.5	27.6	33.4	198	62	27.1	10.5	0	0.4
82.4	27.3	33.2	210	68.4	21.4	9.5	0.4	0.3
82.4	27.7	33.6	348	56.9	37.1	5	0.7	0.3
77.2	27.1	35.1	138	49	33	16	1	1
76.1	24.7	32.5	184	44	39	14	1	0
85.2	29.1	34.2	532	72	17	9	2	0
81.7	28.5	34.9	272	61.5	24.9	5.3	8.1	0.2
72.7	25.6	35.2	133	30	41	3	0	0
84	28.2	33.5	195	35	50	11	3	1
87.8	29.4	33.5	23	32	46	22	0	0
79.7	27.3	34.2	574	54.8	34.4	9.6	0.9	0.3
76.7	26.9	35.1	243	46.6	45.1	6.5	1.6	0.2
78.8	26.3	33.3	171	53	41	5	1	0
79.1	26	32.8	215	52	35	9	0	0
84.9	28.9	34	237	30.9	63.1	4.8	0.8	0.4
76.3	25.7	33.7	272	50	36	11	1	0
79	26.7	33.8	189	64.4	29.6	5.6	0.2	0.2
77.8	26.8	34.5	295	46	35	18	1	0
80	28.3	35.4	233	38	33	21	0	0
82.8	28	33.8	292	72	22.1	2.1	3.7	0.1
81.3	27.6	33.9	229	60.9	26	12.4	0.5	0.2
73.4	24.2	33	316	70	23	7	0	0
81.7	27.5	33.7	274	74.2	18.6	6.4	0.5	0.3
73.7	24	32.6	271	46	46	6	2	0
83.9	28	33.3	176	71.5	21.4	7	0	0.1
82.2	28	34.1	187	69	19	10	1	0
74.4	25.2	33.9	269	75	19	6	0	0
82	27.6	33.7	360	71.2	18.5	9.6	0.6	0.1
81.2	27.7	34.1	422	43	40	17	0	0
78.6	27.5	35	151	64.8	28.2	4.8	2	0.2
78.9	26.5	33.5	131	31	50	11	0	0
78.3	27.3	34.8	260	24	61	13	1	0
78.8	26.6	33.7	98	15	78	3	1	1
78.4	27.1	34.5	236	67.2	22.7	9.5	0.3	0.3
80.2	27.5	34.2	377	53	38	9	0	0
76.9	26.4	34.4	299	82.8	13.9	2.2	0.6	0.5
76.8	26.6	34.6	213	74	17	5	1	0
84	28.1	33.5	188	13	77	5	0	0
82.3	27.4	33.3	155	83.9	9	6.6	0.2	0.3
77.7	26.3	33.9	294	66.9	27.9	4.7	0.3	0.2

79.4	26.4	33.2	295	57.5	31.5	9.5	1	0.5
77	26.6	34.5	504	62	30	7	1	0
80.5	27.7	34.4	275	75	18	7	0	0
80.1	27.8	34.7	437	48.5	43.5	7.5	0.5	0
90.5	31	34.3	321	56	26	16	2	0
80.9	27	33.3	527	62	29	4	2	0.5
95.9	32.3	33.7	373	33	55.1	9.1	2.5	0.3
78.7	25.9	32.9	291	64	24	10.5	1.5	0
83.7	28	33.4	172	36	58	6	0	0
89	30.1	33.8	220	43.3	41.6	12	2.8	0.3
80.4	27.6	34.3	220	55	36	9	0	0
81.7	27.4	33.5	322	84.9	13	1.3	0.5	0.3
76.8	26.7	34.7	312	69	21	10	0	0
78.5	26.2	33.4	447	65.6	25	8	1.1	0.3
83.8	28.4	33.9	224	56	35.6	8.2	0	0.2
91.8	30.8	33.6	390	56	35	7	2	0
79.6	27.1	34.1	211	66	14	11	1	0
73.5	25.2	34.3	499	56.1	30.7	12.7	0.3	0.2
77.4	27.5	35.5	371	81.6	9.1	8.5	0.7	0.1
79	27	34.2	168	74	19	6	0	0
83.3	27.9	33.4	165	55	30	5	4	0
76.9	26.1	33.9	174	46	41	11	1	1
83.1	28.6	34.5	165	70	23	6	1	0
69.4	24.1	34.7	469	64	24	12	0	0
78.6	26	33.1	397	54	36.9	7.7	1.1	0.3
76.3	25.9	33.9	189	18	80	1	1	0
76.3	27.4	35.9	447	81.5	11	7.5	0	0
81.3	26.6	32.8	237	36	49	9	5	1
74.5	24.9	33.4	264	60	28	10	1	0
80.8	27.2	33.7	180	40.2	50	8.9	0.6	0.3
83.8	28.5	34	259	60.1	23.7	15	0.8	0.4
78.6	27.4	34.8	430	54.9	33.8	8.9	2.1	0.3
79.1	27.7	35	115	60.8	25.9	13.1	0	0.2
77.3	26.1	33.8	194	81	13.5	5.5	0	0
78.1	26.7	34.2	272	59.6	33.5	5.7	1	0.2
80.4	27.1	33.7	158	57	26	6	0	0
75.3	24.9	33.1	361	69.5	18	12	0.5	0
82.2	26.8	32.6	315	67.1	22.6	10	0.1	0.2
80.2	26.5	33	178	54	34	11	0	1
77.5	25.3	32.7	436	67	27.9	5.1	0	0
77.5	25.9	33.4	199	67.7	24.1	7.6	0.2	0.4
72.9	24.4	33.5	251	62.2	32.3	4.2	1.2	0.1
77.3	26.1	33.8	236	84	8	7	0	0
70.9	23.8	33.6	149	77	11	6	0	0
76.9	26.5	34.4	360	41.8	43.2	12.9	1.7	0.4
76.6	26.2	34.3	207	26.7	65.7	6.8	0.5	0.3

75.9	26.4	34.8	301	59.4	32.8	6.6	0.9	0.3
89.5	30.3	33.9	728	44.6	47.8	4.1	3.3	0.2
81.4	26.9	33.1	146	50.1	38.7	10.6	0.3	0.3
83.1	27.9	33.5	444	78	12	10	0	0
77.8	26	33.4	165	24.7	68.8	4.7	0.7	1.1
81.8	28	34.2	346	81.7	11.3	5	0	0
74	24.8	33.4	722	51	33	14	0	0
68.4	23.4	34.3	278	58.6	30.3	9.9	1	0.2
96.7	34.6	35.8	324	54	32.5	11.5	0	0
87.4	29.2	33.4	497	47	40	11	0	1
76.7	24.7	32.2	324	32.8	59.8	7	0.2	0.2
80.1	27.1	33.8	215	69	25	6	0	0
91	32	35.1	271	60	29	10	0	0
84.1	27.3	32.4	536	46.6	46.4	6.6	0.1	0.3
79	26.3	33.3	120	42.6	43.8	12.8	0.3	0.5
84	28.2	33.5	303	68	20	11	1	0
85	27.7	32.6	592	78	14.5	7	0	0
73.1	24.9	34	249	48	39	10	0	0
80.5	27	33.5	200	52.2	37.5	9.6	0.5	0.2
79.9	27.9	34.9	201	66	24	5	3	0
78.6	27.7	35.2	382	79	11	8	1	0
84.8	27.9	32.9	243	34	51.4	13.1	0.7	0.8
85.7	29.3	34.1	364	31.5	55.9	10.7	1.2	0.7
89.5	30.8	34.4	441	40.8	44.8	11.2	2.9	0.3
80	26.2	32.8	152	37	57.3	4.3	0	1.4
79.1	27.6	34.9	241	61	35	2	2	0
90.1	30.4	33.8	404	44.1	39	11.9	4.4	0.6
78.5	26.8	34.1	193	79.2	14.9	5.2	0.6	0.1
80.9	27.1	33.5	244	62.5	28	9.3	0	0.2
78.2	27	34.6	273	78.2	13	8.6	0	0.2
84.4	28.4	33.6	177	76.1	13.5	10.2	0	0.2
75.1	25.7	34.3	183	75.9	16.8	6.3	0.8	0.2
73.8	24.3	32.9	408	47	45	7.5	0.5	0
75.1	25.2	33.6	399	87	10	3	0	0
72.2	25	34.6	149	40.8	46.9	11.1	0.6	0.6
84	28.1	33.4	416	73	11	15	0	0
77.8	26.2	33.7	231	65.5	27.2	7.1	0.1	0.1
95.4	32	33.6	540	14	78	4	3	0
70.8	23.2	32.8	281	55.9	31.5	12.1	0	0.5
98.5	32.9	33.4	345	52	39.8	5.1	2.6	0.5
79.4	27.4	34.5	139	43.8	50	4.6	1.4	0.2
78.4	26.7	34	192	62.2	30.7	6.7	0.2	0.2
94.8	32.1	33.8	390	45	37	17	0	0
74.2	25.5	34.4	316	53	29	17	0	1
74.4	25.4	34.2	230	59	15	10	0	0
67.4	21.5	31.9	359	49	43	6	0	0

81.9	27.4	33.4	307	53	33	12	0	0
80.8	27.3	33.8	270	69.1	23.7	6.3	0.6	0.3
77	26.3	34.1	295	52.7	32.9	10.1	4.3	0
76.4	26.2	34.3	264	87.9	5.5	6.5	0	0.1
82.6	29.3	35.4	268	82	13.5	4.3	0	0.2
79.9	26.2	32.7	241	64	28	5	0	0
84.4	28.3	33.6	487	46.3	40.1	10.4	2.9	0.3
81.1	26.7	32.9	336	64	20	13	0	0
78.3	26	33.2	531	70	16	11.5	0	1
79.1	25.1	31.8	299	13	82	5	0	0
81.4	28	34.4	254	33	49	13	0	0
83.2	27.5	33.1	260	46.6	43.7	9.3	0.2	0.2
80.6	27.8	34.5	362	48.6	32.9	13.4	4.7	0.4
80.5	27.9	34.6	234	56	28	16	0	0
78.9	26.7	33.8	214	58	27	13	1	0
79.8	27	33.8	359	4	94	2	0	0
85.5	28.7	33.5	390	25	58	16	1	0
79.7	26.9	33.7	299	39.7	55.9	3	1.1	0.3
75.6	26.2	34.6	202	8	57	19	0	0
75.8	26.6	35.1	191	67.7	21.4	9.3	1.1	0.5
79.7	28.1	35.2	216	69.5	17.3	12.3	0.5	0.4
77.4	25.3	32.7	312	63	19	17	0.5	0
77.4	27.4	35.4	172	66.6	22	11.2	0	0.2
85.3	29.2	34.2	353	59.5	32.4	6.6	1.3	0.2
76.9	26	33.8	385	48.5	42.6	6	2.6	0.3
76.7	26.1	34	221	57.9	33.9	7.8	0.2	0.2
76.6	24.9	32.5	343	46.9	40.6	10.6	1.3	0.6
76.8	25.6	33.3	145	51.7	38.9	8.2	0.8	0.4
87.6	29.4	33.6	227	84.1	9.2	6.6	0	0.1
89.2	31	34.7	473	26.7	64.5	6.4	2.1	0.3
76	25.3	33.3	295	72	10	8	1	0
81.6	27.5	33.7	242	72	17.5	8	0	0
77.1	26.4	34.2	309	68	27	4	1	0
74.4	25.4	34.1	261	62	28	9	1	0
82.3	27.1	33	171	49.8	39.1	10.7	0	0.4
97.4	33.6	34.5	290	47	35	11	2	0
74.8	26.2	35	496	47.2	35.6	13.4	3.1	0.7
77.9	26	33.4	324	33.4	54	12.1	0.3	0.2
79.8	28.1	35.2	277	60	23	15	1	0
84.9	28.8	33.9	261	74.9	14.5	6.5	3.8	0.3
72.3	23.3	32.2	314	49	35	14	2	0
92.4	31.3	33.8	317	54.5	28.5	11.5	2	0
74.6	23.2	31.2	334	52.1	39.2	6	2.5	0.2
73.3	25.8	35.2	312	39.4	51.5	6.8	2.1	0.2
77.1	27.6	35.7	78	13	67	19	0	0
79.4	26.2	33	271	54	32	13	0	0

82.9	27.9	33.7	158	54.3	35	10.3	0.2	0.2
71.3	22.5	31.6	179	28.3	66.4	5.2	0	0.1
82.4	27.9	33.8	142	66.2	25.5	7.3	0.8	0.2
79.6	28.2	35.4	349	69.6	20.3	8.8	1.1	0.2
77.7	26	33.5	81	39.4	45.4	14.9	0	0.3
92.4	31.6	34.3	427	34	48	13	3	0
86	30.5	35.4	405	53.9	36	8.3	1.6	0.2
84.6	28	33.1	157	31.7	55.8	10.5	1.4	0.6
84.7	28.1	33.1	210	64	26	10	0	0
84.8	28.8	33.9	346	31	60	9	0	0
73.1	25.4	34.8	302	72	24	4	0	0
74.5	25.5	34.2	159	70	24	5	0	0
76.9	25.9	33.6	207	68	26	6	0	0
85.9	29.8	34.7	741	53	27	18	0	0
81.7	27.3	33.4	262	54	34	9	2	0
80.3	27	33.6	129	37.7	56.3	5.6	0.1	0.3
75.9	26.3	34.7	346	77	15	5.5	0.5	0
77.3	25.8	33.4	257	66	22	10	1	0
83	28.1	33.8	291	44	45	9.6	1.2	0.2
80.9	28.2	34.8	241	54	31.1	13.3	1	0.6
81.8	28.5	34.9	166	52	35	13	0	0
104.5	35.9	34.3	368	29	61	8	1	0
79.5	27.5	34.6	258	72.6	18.2	2.7	6.4	0.1
83	28.9	34.8	228	57.5	30	11.1	0.8	0.6
78.6	27	34.4	402	70.9	14.9	13.1	0.9	0.2
82.8	28	33.8	309	68.5	21.9	9.3	0.1	0.2
72.1	25	34.7	209	42	45	10	1	0
79.7	28.1	35.3	203	71	19.4	7.5	1.9	0.2
75.2	24.8	33	380	68	26	6	0	0
69.7	23.5	33.7	176	48.7	41.3	8.2	0.8	1
75.4	25.4	33.7	280	38	51	10	1	0
81.7	29.2	35.7	195	69	17	11	0	0
75.9	25.7	33.8	326	38	45	13	1	0
70.7	22.7	32.1	293	65	23.5	11	0.5	0
90.9	31.3	34.5	402	37.9	53.3	7.9	0.6	0.3
80.2	27	33.6	187	35	41	23	0	1
80.6	27.4	34	300	35.6	49.2	14.1	0.7	0.4
90.6	31.8	35.1	338	17.8	63.8	14.7	3.3	0.4
81.4	27.5	33.8	115	52.2	40.2	6	1.3	0.3
101.3	35.7	35.2	351	51	35	12	0	0
71.1	24.1	33.8	246	45	45	9	0	0
80.1	26.6	33.2	132	55.3	32.7	7.5	4.2	0.3
86.1	29.3	34.1	369	55	39	6	0	0
79.4	26.7	33.7	231	70.4	17.6	11.4	0.4	0.2
85	28	32.9	265	37	48	11.8	1.8	1.4
81.6	27.4	33.6	190	72.6	21	5.9	0.3	0.2

92.3	29	31.4	499	60.5	32.9	6.4	0	0.2
71.2	23.1	32.4	298	59	39	2	0	0
80.9	26.8	33.1	363	68.5	20	6	2.5	0
73.8	25.6	34.6	261	79.8	14.8	4.1	1.2	0.1
81.8	29.2	35.7	621	43.2	46.1	9.3	1.2	0.2
78.3	26.6	34	414	82.1	11.4	5.6	0.8	0.1
77.5	26.2	33.8	132	56.5	34.6	8.6	0	0.3
77.6	26.5	34.2	272	42.1	45.3	11.6	0.6	0.4
78.1	26.4	33.8	350	72	21	3.5	0	0
75.2	25.7	34.2	276	47.2	42.5	9	1.2	0.1
78.2	26.8	34.3	254	83.2	6.8	9.8	0	0.2
77.4	26.6	34.4	117	44	45	10	0	0
92.7	32.2	34.7	72	58.2	32.4	8.9	0.1	0.4
78.2	26.9	34.4	352	82.5	12.5	5	0	0
78.3	26	33.2	492	37.4	58.9	2.6	0.8	0.3
73.5	25.1	34.1	341	72.5	16	7	1	0.5
85.5	28.2	32.9	324	27.4	63.2	6.3	2.7	0.4
74.5	26.1	35	167	82.9	7	9.9	0	0.2
106.7	36.8	34.5	288	50	26	16	4	0
75.8	25.2	33.2	358	50.1	39.1	8.7	1.6	0.5
81.1	27.8	34.3	355	45.7	41.9	6.2	5.9	0.3
82.3	27.8	33.7	283	51.6	38.2	8.8	0.9	0.5
81.3	27.9	34.3	198	67	28	5	0	0
81.9	26.9	32.8	257	50.6	36.2	12.9	0.1	0.2
78.3	27.4	35	532	54.5	26.5	17	1.5	0
75.1	25.8	34.4	187	36.3	49.7	13.6	0.2	0.2
81.5	26.8	32.9	224	70	24	5	0	0
78.2	26.5	33.9	199	51.1	39.8	7.9	0.2	1
77.8	25.9	33.3	164	52	46	2	0	0
83.3	27.6	33.1	157	69.4	23.1	6.8	0.5	0.2
78.1	26.3	33.6	349	57.5	31.5	10	0.5	0.5
76.6	25.3	33.1	138	29.9	62.8	6.5	0	0.8
77.8	27.4	35.2	292	49	40	8	2	0
80.8	27.7	34.3	145	78.3	13.3	8	0.2	0.2
76.2	25.2	33	145	84.3	10.1	4.6	0.9	0.1
79	26.6	33.7	264	61.2	28.4	10	0.2	0.2
87.2	29.4	33.7	335	17	68	7	6	2
73.8	25.3	34.3	255	39.5	53.9	5.1	1.4	0.1
80.6	27.9	34.6	189	57	32	10	0	1
77.2	25.6	33.1	411	64.9	24.7	10	0.2	0.2
75.7	24.5	32.4	274	45	41	13	0	0
72.2	24.6	34.1	575	76.5	20.9	1.6	0.9	0.1
86.9	31.8	36.6	410	23.2	69.5	5.1	1.9	0.3
76	26	34.2	213	53.4	37	7.9	1.5	0.2
79.7	28.6	35.9	515	62.7	32.6	4.1	0.4	0.2
78.2	25.7	32.8	215	51.5	41.2	6.4	0.7	0.2

80.9	26.9	33.2	197	85	10	5	0	0
90.4	32.5	36	283	55	40	3	2	0
90.3	30.9	34.2	282	54.3	39.7	2.7	2.9	0.4
72.9	25.4	34.9	379	64	29	7	0	0
73.5	24.5	33.3	168	23	67	7	0	0
77.3	27.1	35.1	494	71	17	9.5	2.5	0
79.2	26.7	33.7	284	68.7	18.3	11.4	0	0
77.3	25.8	33.3	183	26	58	14	0	2
82.6	28.2	34.1	333	55.2	31.3	12.8	0.5	0.2
79.4	27	34	119	64.4	25.6	10	0	0
97.9	33.3	34	374	51	27	15	7	0
84.6	29.1	34.4	450	55.1	37.8	5.7	1.2	0.2
78.4	27.1	34.5	614	69.5	21	6	1	1
77	25.4	33	293	74	18	6	0	0
75.8	25.7	33.9	264	33	44	19	2	0
80.5	27.6	34.3	212	73.7	15.4	9.1	1.2	0.6
82.8	28.2	34	195	32.8	52.9	13.3	0.5	0.5
79.3	26.8	33.8	241	16	77	5	2	0
75.4	25	33.1	402	47.6	43.5	5.1	3.2	0.6
73.5	25.7	34.9	236	35	50	14	1	0
95	32.3	33.9	336	45.7	40.7	8	5.2	0.4
80.3	27.7	34.5	256	45	41.5	8.1	4.5	0.9
75.8	24.5	32.3	272	39.4	45.5	13.9	0.2	1
87.1	29.8	34.2	134	39.6	55.7	2.9	1.8	0
78.1	27.1	34.7	355	71.8	21.6	5.4	1	0.2
84.7	29.3	34.6	313	47.9	41	7.6	3.3	0.2
80	26.7	33.4	155	50.8	41.9	6.7	0.4	0.2
87.6	29.4	33.6	486	43.8	44.7	9.2	2.1	0.2
75.4	25.7	34.1	240	54	34	10	1	0
77.2	27.5	35.7	346	39	47	12	2	0
77	26	33.8	225	67	26.8	5.9	0.2	0.1
90.8	30.6	33.7	396	23	64	9	2	0
96.5	32.2	33.3	320	42	52.2	4.6	1	0.2
75.2	26.3	34.9	177	56	32	12	0	0
78.1	26.4	33.8	302	76.5	14	7.5	1	0
84.8	28.1	33.1	210	61	31.4	7.3	0.1	0.2
77.8	26.5	34	517	51	41	4	4	0
71	23.6	33.2	279	76.5	13	7.5	0	0
75.9	25.6	33.8	257	26.3	60.8	10.2	2.3	0.4
88.9	30.5	34.3	220	30.3	56.7	10.7	1.4	0.9
75.3	25.6	34	180	55	25	19	0	0
94	32.1	34.1	509	40	33	19	1	0
81.4	25.8	31.7	145	49.6	38.7	9.2	2.3	0.2
78.1	26	33.3	141	25.7	68.2	5.2	0.3	0.6
75.7	24.3	32.2	248	24	61	11	4	0
91.5	29.8	32.6	227	54.5	34.5	7	0.5	0

76.1	25.5	33.4	188	73	24	3	0	0
91	31.3	34.4	426	38.5	42.5	19	0	0
81.8	28.7	35	258	79.9	16.5	3.5	0	0.1
77.3	25.8	33.3	225	23.5	64.7	9.2	2.3	0.3
91.8	30.1	32.8	326	58.1	29.5	11.8	0.3	0.3
71.5	23.9	33.4	128	66.2	20.6	12.8	0.2	0.2
76.4	26.5	34.7	287	77.5	10	9.5	0	0
77.1	25.8	33.4	349	62.9	30.7	5.4	0.8	0.2
79.6	26.4	33.2	295	73.9	13.3	9.6	3	0.2
78.7	27.1	34.4	321	72	20	8	0	0
85.1	29.5	34.7	384	62.8	22.7	11.8	2.4	0.3
81	28.9	35.7	275	85.1	12.1	2.4	0.3	0.1
80.4	27.2	33.8	199	41.3	47.1	10.8	0.3	0.5
74	24.6	33.2	246	48.6	43.5	7.4	0.3	0.2
79.6	27.9	35	224	46	43.8	7.3	2.5	0.4
79.8	27.9	34.9	188	77	11.5	8.5	0	0
76.5	26.2	34.2	351	47.9	40.7	8	3.2	0.2
79.7	26.8	33.6	300	59	34	7	0	0
79.3	26.3	33.1	245	65	19	12	0	0
82.7	27.8	33.7	306	41.3	43	12.2	3.1	0.4
81.2	28.4	35	419	51.9	38.5	4.2	5.1	0.3
74.6	25.6	34.4	146	79.1	16.1	4.4	0.3	0.1
79.1	26.7	33.7	152	57.4	32.1	8	2.3	0.2
66	21.1	32	177	56.7	33.4	9.4	0.4	0.1
72.9	23.1	31.7	226	52.4	30.8	15.3	0.5	1
82.3	27.8	33.8	123	23.8	66	5.5	4.1	0.6
80.4	27.7	34.4	581	80	11	9	0	0
84.4	28.5	33.8	245	88	4	7	0	0
86.7	28.6	33	131	49.4	43.5	6.7	0	0.4
80.2	27.1	33.8	140	61	29	10	0	0
79.2	26.6	33.7	193	5	66	22	0	1
80.4	26.5	32.9	173	57	31	10	1	0
84.4	29.3	34.7	432	41	42	16	1	0
75	24.5	32.6	151	47.2	41.7	10.2	0.2	0.7
81.4	27.5	33.8	215	50.8	38.3	9.5	1.2	0.2
78.5	29	37	306	88.2	8.7	2.8	0.2	0.1
80.4	27.5	34.2	125	61.7	28.3	9.7	0	0.3
74	25.7	34.7	394	66.8	22	10.6	0.3	0.3
82.8	27.7	33.4	302	78	13	7	0	0
74.7	25.3	33.9	230	55	26	17	1	0
70.7	23.3	33	192	61.7	27.1	10.8	0	0.4
81.7	27.4	33.5	161	65.2	23.3	11.1	0.1	0.3
74.4	24.9	33.5	143	71	14	10	1	0
69.8	21.7	31.1	404	37	52	11	0	0
81.9	27.3	33.3	180	60.1	27.7	11.7	0.3	0.2
76.1	25.8	33.9	169	44.2	39.9	14.2	0.7	1

72.7	24.1	33.1	204	61.6	33	4.6	0.4	0.4
79.8	26.8	33.5	222	55.3	30	13.8	0.3	0.6
80	26.9	33.6	490	69.5	17.4	12.4	0.3	0.4
84.9	28.7	33.8	227	52.5	34.5	7	0.5	0
69.1	22.6	32.7	414	86.6	7.8	4.9	0.4	0.3
81.4	27.4	33.6	286	68.2	24	7.5	0	0.3
78.5	27	34.4	352	61	35	4	0	0
77.8	26.9	34.5	260	72.4	15.9	11.4	0.1	0.2
80.7	27.3	33.8	243	70	15	9	0	0
85.3	28.6	33.6	214	91.8	4.5	3.7	0	0
105.1	38	36.1	216	45.9	39.9	9.6	4.3	0.3
75.9	25.4	33.4	268	70	18	12	0	0
77.6	25.7	33.1	536	38.5	43.5	15.5	0	0.5
74.1	24.2	32.7	409	57	32	9	1	0
81.7	27.3	33.4	254	71.3	17.7	10.9	0	0.1
81.3	28	34.4	377	59.9	29.9	9.2	0.8	0.2
81.1	27.9	34.4	379	49.3	38.6	11.1	0.9	0.1
83.8	28.1	33.5	63	28	62	6	1	0
74	25.1	33.9	370	75.5	15.5	8.5	0	0
81.9	26.8	32.8	396	57.2	39.3	1.6	1.6	0.3
93.4	31.6	33.9	346	42	39	16	3	0
79.6	27	33.9	312	91.5	4	3.9	0	0
77.8	25.9	33.2	614	66	25.5	7.5	1	0
85.5	30	35.1	611	61.3	22.6	15	0.8	0.3
80	27.8	34.8	155	57.9	32.6	9.4	0	0.1
86.8	30.1	34.7	229	58	28	13	1	0
76.7	26.9	35.1	259	60	26	12	2	0
86.7	29.7	34.3	454	45	47.6	6.2	1	0.2
78.6	26.5	33.8	458	74.4	16.3	8.6	0.3	0.4
91.8	32.3	35.2	264	43.4	43.9	10.8	1.1	0.8
83.7	29	34.7	246	13	80	7	0	0
74.9	23.2	31	284	60	33	7	0	0
79	27.1	34.2	227	73.5	18	5.5	1	0
76.9	25.9	33.6	213	54.1	32.8	12.1	0.5	0.5
83.1	28	33.7	124	38.5	50.1	10.7	0.2	0.5
79.7	27.1	34	180	50	35	14	0	0
101	35.2	34.8	285	42	38	16	3	0
97.5	34.3	35.1	270	72	16	8	3	0
81.9	27.2	33.2	255	84	7.5	6	0	0
77.3	27	35	322	81.5	9.5	8.5	0	0
81.3	28	34.5	449	79	9	7.5	0	0
88.7	29.6	33.3	558	38	44	16	2	0
75.9	25	32.9	281	30.9	59.2	8.3	1.3	0.3
73.1	24.1	33	269	55.9	32	11.4	0.6	0.1
80.5	26.5	32.9	216	52.4	36.7	7.5	2.9	0.5
76.9	26.3	34.2	161	25	61	7	0	0

78.3	27.7	35.3	258	37.2	52.4	5.7	4.5	0.2
80.1	27	33.8	140	34	47	13	2	0
74.8	24.8	33.1	196	2	69	13	1	0
79.7	28.1	35.2	509	60.8	32.6	5.9	0.2	0.5
79.6	27.9	35	579	66.7	25.4	7.1	0.6	0.2
82.3	27.3	33.2	302	55.5	28.5	14	1	0.5
97.9	34.4	35.2	337	24.8	59.9	9.7	5.2	0.4
73	25.8	35.3	381	56.1	36.4	3.8	3.4	0.3
72.2	24.7	34.3	235	19	34	40	0	0
90.5	31.2	34.5	466	22.3	66.5	7.4	2.8	1
98	33.1	33.8	152	17	73	9	1	0
80.4	27	33.5	482	70.1	22.3	7	0.4	0.2
94.3	33.2	35.3	505	51.5	25.5	18.5	2.5	0.5
79.4	26.7	33.6	245	57.7	33.3	8.5	0.3	0.2
74.9	25.6	34.2	281	80.5	10.5	8.5	0	0
83.3	27.2	32.6	366	35	56	7	2	0
81.3	28.3	34.9	264	59	31	10	0	0
78.8	26	32.9	142	77.8	14.6	7.4	0	0.2
75.8	24.8	32.8	197	60.6	30.3	8.5	0.2	0.4
75.4	26.7	35.4	400	64	22	11	1	0
75.6	26	34.3	178	70	14	16	0	0
78.4	27.4	35	308	53.9	33.7	9.6	2.2	0.6
82	28.2	34.4	323	86	10	4	0	0
81.9	28.6	34.9	498	53.2	40.7	5.2	0.6	0.3
75.7	26	34.4	125	23	61	13	0	1
80.9	28.1	34.8	198	78.3	11.2	10.5	0	0
78.7	25.9	32.9	347	45	37.6	11.3	5.8	0.3
74.2	24.6	33.1	200	76.6	15.8	7.3	0.1	0.2
80.8	27.2	33.6	346	81.3	12.6	5.4	0.6	0.1
87.1	28.6	32.8	260	48.4	42.7	6.6	1.3	1
70.5	23.9	33.9	341	67.2	21.6	8.5	2.3	0.4
85.6	28.1	32.9	441	32.8	54.8	7.2	4.9	0.3
79.9	27	33.7	195	71	11	15	0	0
82.4	28.8	35	268	68	22.7	6.7	2.5	0.1
78.6	26.4	33.6	207	57.3	27.3	13.4	1.5	0.5
78.4	26	33.1	104	68	25	5	0	0
102.5	35.2	34.4	255	39.4	45.1	11.3	3.8	0.4
75.3	25.8	34.3	189	60.2	34.3	5.3	0	0.2
82.1	28.2	34.4	281	70	18.8	10.3	0.7	0.2
82.3	27.4	33.3	243	47	43.4	9.2	0	0.4
79.8	27.4	34.3	191	58	26	15	0	0
75.6	25.9	34.2	283	72.7	20.1	5.8	1.3	0.1
81.1	27.7	34.2	277	87	9	4	0	0
87.4	29.4	33.6	317	15	70	12	2	1
83.1	28.3	34	256	53	31	15	1	0
82.9	27.9	33.6	593	54.1	30.4	13.8	1.5	0.2

81.9	27.4	33.4	164	70	20	7	0	0
71	23.5	33.1	297	60	29	9.5	0	0.5
94.2	32.1	34.1	381	55	36	8	1	0
93.7	31.1	33.2	355	56	28.9	11.2	3.6	0.3
79.5	27.4	34.5	231	51	35.5	12.5	0.6	0.4
80.2	27.8	34.7	501	60.5	29	10	0	0
81.6	27.8	34.1	215	33.2	55.5	9.9	1.1	0.3
82	27.6	33.7	364	56	32	11	0	0
72.8	24.5	33.7	131	51.7	37.7	7.3	3.1	0.2
78.8	27	34.3	228	32.1	53.6	13.6	0.5	0.2
81	28	34.6	255	33.7	54.7	9.6	1.5	0.5
76.9	26.7	34.7	200	46.5	44	8.3	1	0.2
83.8	30.1	35.9	273	41	52	6	1	0
74.3	26.2	35.3	291	43.9	42.7	7.3	5.9	0.2
79.7	27.9	35	343	68	18	13	1	0
82.3	29.3	35.6	374	27.3	61.5	7.4	3.4	0.4
80.4	27.3	33.9	147	38.2	54	7	0.3	0.5
82.7	28.8	34.8	375	53.3	37.9	6.2	2.5	0.1
87	29.5	33.9	290	52.7	32.6	12.3	2.2	0.2
75.8	27	35.7	153	74	13	10	2	0
78.8	26.4	33.5	438	45	38	12	5	0
81.3	27.2	33.4	257	76	14	5	1	0
90.8	30.9	34.1	867	40.5	39.5	4.5	0	0
76	26.6	35	219	53	45	2	0	0
73.4	25	34	427	46.5	39.5	12.5	0	0.5
84.1	27.8	33.1	285	31	58	10	0	0
78.7	26.8	34	337	49.8	44.5	2.2	3.3	0.2
78	26.4	33.8	239	63.1	26.8	8.9	0.9	0.3
76.2	26.1	34.3	294	19.3	65.4	14.7	0.4	0.2
79.7	27.4	34.4	263	60	31	7	1	0
75.2	25.7	34.2	270	46.5	39.5	13.7	0.1	0.2
79.7	27.1	34	189	45.9	40.6	8.6	4.1	0.8
75.5	26	34.4	271	62.5	26.6	10	0.2	0.7
78.1	26.8	34.3	202	84.7	8.9	6.1	0.2	0.1
81.9	27.4	33.5	274	86.8	9.7	3.3	0.1	0.1
79.5	26.7	33.6	275	86	9	4	1	0
87.5	29.4	33.6	150	77.3	14.1	8.2	0.2	0.2
75.1	24.7	32.9	296	68	25	5	2	0
77.9	26.7	34.3	159	41.9	47.8	9.9	0	0.4
80.5	27.2	33.8	222	76.4	17	6.3	0.2	0.1
74	25.1	33.9	230	68	24	7	0	0
70.8	23.2	32.8	152	66	24	9	0	0
84.6	28.5	33.7	332	31.6	64.4	2.9	0.9	0.2
74.3	23.7	32	257	21	73	6	0	0
93.2	30.6	32.9	473	62	22	16	0	0
94.4	32.9	34.8	196	24.7	59.6	11.7	3.8	0.2

77	26.7	34.7	227	32.7	59.7	3.8	3.6	0.2
83.1	28	33.7	149	22.4	66.5	8.9	1.7	0.5
79.6	27.2	34.2	187	73.2	19.9	6	0.6	0.3
76.9	25.7	33.4	159	68.3	24.7	7	0	0
77.1	25.9	33.5	157	72	15.6	11.8	0.3	0.3
70.6	23.3	33	229	47	37.7	14.2	0.3	0.8
80.9	27.9	34.5	310	56	35	9	0	0
92.7	31.8	34.3	514	15	71	10	4	0
87.4	29.9	34.2	309	34.3	55.1	9.4	1	0.2
76.8	26.8	34.9	193	65	20	11	1	0
85.4	28.8	33.7	436	7	86	2	5	0
85.8	27.1	31.6	279	49.5	30.5	12	0	1
83.1	28.5	34.2	174	64	19	7	0	0
78.1	25.4	32.6	186	38.5	49.6	11.6	0	0.3
80.6	28.7	35.6	208	28	49	6	0	0
81.6	27.5	33.7	203	47	43	9	0	1
75	24.3	32.5	130	34.8	54	10.5	0.3	0.4
84.8	27.4	32.3	303	23.6	73.3	1.3	1.8	0
67.9	22	32.4	244	72.2	14.7	12.7	0.2	0.2
79.9	26.8	33.5	364	50.2	41.1	7.7	0.7	0.3
86.4	28.8	33.3	335	74	19	4.5	1.5	0.5
76.3	26	34.1	390	56.2	34.1	8.1	1.5	0.1
79.5	24.9	31.4	214	42.8	46	8.5	2.3	0.4
83.3	27.9	33.5	260	57.5	36.6	4.3	1.3	0.3
78.3	27	34.4	168	28	20.5	43	0	0
97.3	33.1	34.1	468	40	52	6	2	0
94.4	31.9	33.8	254	53	29	15	1	0
74.8	25.4	34	249	31	44	20	2	1
74	23.8	32.1	281	51	34	13	2	0
96.5	33.5	34.7	310	63.5	25.5	8.5	2.3	0.2
81.3	27.7	34	317	85	10.7	4.2	0	0.1
77.3	26.1	33.7	101	61	30	9	0	0
71.1	22.7	31.9	259	48.2	37.2	11.6	2.8	0.2
83	28.1	33.8	119	41	43	13	1	1
81.1	27.7	34.1	244	6	87	6	0	1
78.9	27.4	34.7	195	42	46	11	0	1
79	27.3	34.5	307	35	59	4	2	0
91.8	32.4	35.2	496	43.4	46.9	8	1.7	0
76.6	25.9	33.8	387	51	41	7.5	0	0
85	28.4	33.4	244	55	35	10	0	0
83.7	28.2	33.7	416	60.5	30.5	7.5	1.5	0
68.8	22.3	32.5	183	27	62	11	0	0
73.3	26.2	35.8	323	47	42	8	3	0
81.1	27.6	34.1	417	80	12.5	5.5	1.5	0.5
92.6	31.5	34	297	65.3	27.4	7	0	0.3
93.9	31.2	33.2	448	40.2	52.9	3.3	3.6	0

74.1	24.9	33.6	225	72.9	19.1	5.8	1.7	0.5
81.8	26.6	32.5	157	60	37	1	2	0
95.9	33.2	34.6	274	24	59	11	6	0
80.7	27.9	34.5	334	42.5	43.8	7.9	5.4	0.4
85.8	28.7	33.4	297	55.6	38.1	5.1	1	0.2
78.7	25	31.8	190	28	61	6	3	0
98.5	32.8	33.3	327	53	33	9	4	0
82.6	27.8	33.7	607	28.5	60.9	7.1	3	0.5
81.5	27.4	33.6	137	50	32	16	0	1
85.8	27.8	32.5	539	62.9	26.9	8.8	1.2	0.2
76.4	26	34.1	233	57.3	32.3	9.5	0.7	0.2
77.3	27.1	35	205	17	74	7	2	0
79	26.7	33.7	211	44.2	40.7	14.1	0.5	0.5
83.6	27.6	33	526	45.9	43.9	8.7	1.3	0.2
82.4	28.6	34.8	258	32.5	60.1	6	1	0.4
84.8	28.8	34	351	55	36	9	0	0
79.8	27.7	34.7	194	81.1	13.5	5.1	0.1	0.2
76.7	25.9	33.8	243	47.9	39.3	9	3.3	0.5
78.1	26.3	33.7	236	26.3	66.7	5.8	0.7	0.5
78.9	26.2	33.2	249	27.5	62.8	8.3	1.1	0.3
86.5	30	34.6	261	72.8	14.4	11.3	1.4	0.1
84.2	27.7	32.9	514	64	28	7	0	0
80.7	27.4	34	335	60.5	31.7	7.4	0.2	0.2
80.2	26.7	33.2	167	52	38	9.6	0.3	0.1
78.9	26.7	33.8	150	21	64	15	0	0
84.6	28.3	33.4	168	76.3	22.1	1.3	0	0.3
77	26.1	33.9	156	45.7	43.1	11	0.1	0.1
66.1	21.6	32.7	385	63	34	3	0	0
73.7	24.4	33.1	296	42.1	42.1	9	6.6	0.2
80	27.4	34.2	227	81.5	14.8	3.4	0	0.3
77.4	25.9	33.5	479	60.5	31.5	8	0	0
82.4	27.9	33.8	450	65.1	23.3	10.5	0.8	0.3
78.6	27.9	35.5	308	58.4	37.2	3.7	0.4	0.3
74.5	25.3	33.9	341	54	43	3	0	0
75.6	26.5	35.1	155	54.1	32.5	12.9	0.1	0.4
75.2	25.6	34	116	58.8	32.5	7.7	0.7	0.3
79.7	26.6	33.3	382	67.5	22.6	9.3	0.3	0.3
82.1	27	32.9	338	68	22.5	8	0.5	0
79.5	26.6	33.4	131	60.1	32.6	6.5	0.2	0.6
81.8	27.1	33.2	214	62.1	26.1	10.8	0.9	0.1
81.3	26.9	33	237	30	64	6	0	0
84.1	28.7	34.2	197	84.4	8.3	7.2	0	0.1
76.8	26.2	34.1	151	60	30	10	0	0
76.9	26.3	34.2	139	38	48	9	0	1
79.4	26.8	33.8	383	65.9	26.5	6.9	0.3	0.4
77.5	25.6	33.1	164	43.7	48.4	7.3	0.3	0.3

80.9	27.9	34.5	223	51.5	33.2	13.3	1.9	0.1
83.8	27.5	32.8	220	21	66	10	1	0
77.4	26.3	34	152	26	64.8	8.3	0.3	0.6
79.7	27.4	34.4	203	62.4	27.6	8.3	1.1	0.6
86.5	29.5	34.1	537	49.8	38.1	10.3	1.5	0.3
69.2	22.9	33.1	399	57	37	6	0	0
82.4	27.5	33.3	129	53.6	30.4	14.3	1.5	0.2
77	26	33.8	243	37	54	7	2	0
81.8	28.1	34.3	306	56.7	32.2	10.5	0	0.6
79.4	27.1	34.1	211	29	56	13	1	0
72.2	23.7	32.9	408	40.5	51	8.5	0	0
80.1	27.9	34.8	264	74.5	19.5	5.7	0.2	0.1
78.1	26.1	33.4	294	39.7	49.7	8.1	2.4	0.1
74	24.5	33.1	231	29	60	10	0	0
77.2	25.8	33.4	232	38	36	15	0	0
78.1	26.6	34.1	213	36.6	50.2	11.6	1.2	0.4
76.7	25.8	33.6	198	60	27	13	0	0
81.7	27.7	33.9	265	62.2	30.9	5.4	1	0.5
79.4	27.2	34.2	278	55.1	36.5	8	0	0.4
79.2	27.2	34.3	289	30.4	62	7.3	0.2	0.1
76.7	26.1	34	210	65.3	26.2	8.2	0	0.3
83.5	28.8	34.5	423	67.8	20.9	10.7	0.3	0.3
73.7	24.7	33.4	217	42	29	12	2	0
80.4	25.2	31.4	328	30	55.7	9.9	4.1	0.3
76.8	26.6	34.6	253	56.9	33.3	9.4	0.2	0.2
82.9	28.3	34.1	245	50.5	38.4	9.2	1.6	0.3
75.5	25.2	33.4	259	45	46	8	0	1
84.6	28.5	33.6	365	65.9	26.6	6.7	0.5	0.3
78.3	26.2	33.5	355	39.4	50.1	9.1	0.6	0.8
80.5	26.9	33.4	243	84.5	3	11	0	0
84.2	27.8	33	387	17	74	6	3	0
82.4	29	35.2	200	40	45	13	1	0
76.1	26.2	34.4	187	56.7	36.9	4.9	1.4	0.1
82.5	27.3	33.1	107	52.7	40.2	6.3	0.2	0.6
81.2	26.9	33.1	182	37.5	54.2	7.6	0.3	0.4
77	25.7	33.4	279	79.7	13.8	6.2	0.1	0.2
77.6	25.3	32.6	295	64	23	12	0	0
97.8	33.8	34.5	258	60	33	7	0	0
80.8	27.8	34.3	254	62	20	16	1	0
78.9	27.4	34.7	183	29	58	12	1	0
72.5	24.6	33.9	376	75.5	12.5	12	0	0
76.5	25.5	33.3	165	42.5	41.6	14.5	1.1	0.3
79.4	26.3	33.1	293	62	26	10	2	0
81.9	27.1	33.1	152	51	34	8	0	1
99.3	33.5	33.7	261	60	25	11	3	0
76.4	26.6	34.9	191	63.1	27	9.1	0.7	0.1

81.4	27.6	33.9	142	51	32	16	0	0
79.4	27.2	34.3	459	25.6	62.7	10	1.3	0.4
80.6	27.2	33.7	225	47.5	45.6	6.1	0.7	0.1
76.1	26.3	34.6	233	51.9	37.2	9.8	0.7	0.4
82.2	27.3	33.2	230	32	59	8	0	1
91.3	30.7	33.6	360	26	56	16	2	0
78.8	26.6	33.8	79	19	73	6	0	0
74.8	25	33.4	238	53	29	17	0	0
81.1	27.2	33.5	204	64.1	26.6	9.1	0.1	0.1
79	26.5	33.6	189	89.9	4	5.9	0.1	0.1
77.6	28	36.1	232	64.2	23.1	11.6	0.6	0.5
78.5	26.7	34	307	52	35.6	11.4	0.6	0.4
78.4	26.8	34.2	231	71	24	5	0	0
78	25.9	33.2	135	49	37	14	0	0
82	28.1	34.3	258	33	35	26	3	1
75.6	26.7	35.3	282	32	47	19	0	0
84.3	28.8	34.1	398	58.9	29	9.7	2.1	0.3
74.9	25.5	34	154	26	64	8	0	0
89.2	30.1	33.7	376	41.8	44.3	10.9	2.7	0.3
82.6	29.5	35.7	280	73.4	11.2	14.4	0.9	0.1
83.6	28.2	33.8	298	27.1	63	8.1	1.3	0.5
80	27.2	34	197	68	18	13	0	0
73.6	26.2	35.5	473	65	27.4	4.8	2.7	0.1
71.8	23.8	33.1	153	34.3	55	10.1	0.2	0.4
73.8	25.1	33.9	211	10	82	5	3	0
78.3	26.4	33.8	439	64.1	21.6	13	0.9	0.4
75.9	25.8	34	404	47	39	13	0	1
79.6	26.3	33.1	271	52	44	3	0	0
81.8	27.7	33.9	471	29.6	63.1	6.6	0.4	0.3
83	27.6	33.3	118	40.9	52.3	6.7	0	0.1
73.6	25.2	34.2	244	10	77	9	2	0
82.1	28.5	34.7	255	35.6	49.8	12	2	0.6
79.8	26.6	33.3	120	38.6	51.3	8.7	1	0.4
70.2	23	32.8	485	72	16	7	1	0
73.5	25.3	34.5	204	64	23	10	1	0
77.4	26.4	34.1	275	38.5	51	9.5	0.5	0.5
92	31.3	34	278	43	25	27	5	0
89.2	30.6	34.3	308	27	43	23	4	1
81.7	28	34.3	267	52	39	9	0	0
77.8	27.4	35.2	271	41.2	43.7	12.4	2.4	0.3
79.6	26.2	32.9	100	45.8	44.8	8.8	0.2	0.4
75.5	24.9	33	314	48	43	9	0	0
71	23.6	33.2	53	25	55	15	2	1
97	32.7	33.7	503	34	59	5	1	0
77.5	26.1	33.6	482	55	30	14	1	0
78.8	26.1	33.1	273	57	37	6	0	0

90.4	31.1	34.4	331	30.2	60.7	5.9	2.8	0.4
75.2	27.7	36.9	173	76	13	7	2	0
86.1	27.2	31.6	291	23.6	68.8	6.3	0.7	0.6
83.6	27.9	33.3	219	51.5	31.8	11.4	5.3	0
77	26.8	34.8	160	64	20	11	1	0
79.7	26.9	33.8	488	71	13	12	3	1
79.6	26.7	33.5	362	24.6	62.3	12.5	0.3	0.3
81.1	28	34.5	178	33.2	59.1	6.6	0.7	0.4
74.8	24.1	32.2	113	29.9	59	10.4	0.2	0.5
80.6	27.2	33.8	186	43	39	8	1	0
72.9	25.1	34.4	455	41.6	40.5	14.4	3	0.5
75.9	26.4	34.7	224	39	47	9	2	0
79.5	28.4	35.7	496	51	40.5	7	1	0.5
80.6	28.4	35.2	524	66	20	13	0	0
79.7	27.9	35	283	72.2	17.8	9.7	0	0.3
80.8	27.1	33.5	225	63	27.9	8.3	0.4	0.4
75.1	25.2	33.5	234	40.9	47.1	10.8	0.8	0.4
77.4	26.2	33.8	186	68	14	16	0	0
84.9	28.3	33.3	240	39	26	31	0	0
80.1	27.3	34.1	233	54	32.6	11.1	2.1	0.2
79	26.2	33.1	148	26.6	58.6	13.1	1.5	0.2
80.8	27.9	34.5	203	50.1	37.2	6.5	5.9	0.3
69.4	23.5	33.9	245	22	71	6	1	0
76.9	26.5	34.4	333	67.6	27.6	4.2	0.1	0.5
87.5	31.1	35.6	53	63	19	12.5	0	0
81.2	27.3	33.6	262	80	16	4	0	0
80.7	27.5	34.1	240	64.8	25.5	7.9	1.4	0.4
74.4	24.8	33.3	302	64.4	16.7	11.2	7	0.7
70.7	24.9	35.2	247	63.7	23.6	12.3	0.1	0.3
79.2	27.4	34.6	304	52.2	35.8	10.7	1.1	0.2
76.5	26.6	34.8	294	40	53.3	6	0.4	0.3
79.5	26.7	33.5	340	42.7	44.9	10.1	2	0.3
82.3	27.5	33.4	171	73.9	15.7	10.2	0.1	0.1
85.3	29.6	34.7	401	24	69	5	2	0
92.5	30.8	33.3	219	54.7	37.4	6.7	0.9	0.3
84.3	28.2	33.5	219	60	31	8	1	0
95.4	31.5	33	289	28.1	61	9.4	0.8	0.7
80.7	27.2	33.7	128	48.4	40.1	11.3	0	0.2
78.2	25.6	32.8	386	63.9	27.1	6.3	2.5	0.2
84.7	27.9	33	489	46.5	45.3	6.3	1.6	0.3
71.5	23.9	33.4	153	41.5	51.4	6.5	0.2	0.4
77.7	26	33.4	222	83.4	11.1	4.5	0.9	0.1
80	27	33.8	189	31	58	4	6	1
73.2	23.4	32	314	48	39	9	0	1
75.8	25.9	34.2	184	36	49	13	2	0
81.4	26.5	32.6	416	55.3	37.4	6.9	0.2	0.2

78	26.2	33.5	236	16	72	9	2	0
79.3	26.9	33.9	239	47	45	8	0	0
79.4	26.7	33.6	199	27.1	64.3	6.4	1.6	0.6
83.2	28.7	34.5	251	57	25	17	1	0
87.5	29.7	34	759	48.1	42	8.4	1.3	0.2
77	26.2	34	178	43	45	10	1	1
79.5	27.2	34.2	165	45	43	11	1	0
75.7	26.2	34.6	194	46	32	18	0	0
73.4	24.3	33.2	156	46.8	44.5	6.1	2	0.6
79.7	26.5	33.2	511	58	39	3	0	0
96.2	32.4	33.7	430	30	48	14	0	0
78.2	26.6	34	191	30.8	58.7	10.1	0.2	0.2
80.7	27.2	33.7	118	30.2	61	8.3	0.1	0.4
67	22.4	33.4	231	42.5	54.4	2.6	0.3	0.2
77.9	25.7	33	233	66.6	25.4	7.9	0	0.1
80.3	26.6	33.1	242	17	72	8	0	0
100.7	33.7	33.4	342	33.3	58.9	6.4	1.2	0.2
78.4	25.8	32.9	182	64.8	27.3	7.5	0.2	0.2
78.4	26.4	33.6	220	58	34.2	7.3	0	0.5
82.9	28	33.8	253	50.1	36.7	8.6	4.3	0.3
81.7	27.2	33.3	320	10	84	6	0	0
75.4	24.6	32.6	253	38.2	54.5	5.8	0.9	0.6
90.9	31.6	34.8	513	80.6	11.7	6.9	0.6	0.2
95	31.3	32.9	434	55	30	15	0	0
78.9	27.6	35	291	70.2	20.7	7.9	0.9	0.3
78.7	26.9	34.2	141	44.3	40.6	14.5	0.2	0.4
84.7	28.3	33.4	485	58.2	30.8	10.3	0.2	0.5
75.9	26.8	35.4	422	47.3	39.6	12	0.3	0.8
85.5	28.7	33.5	293	67.2	23.4	8.7	0.5	0.2
79.1	26.8	33.9	345	65.9	22.3	10.8	0.8	0.2
76.3	25.8	33.8	149	50	43	5	0	0
75	25	33.4	179	54	34.2	10.5	1.1	0.2
80.8	26.5	32.9	274	52.3	33.1	6	8.4	0.2
81.3	27.9	34.3	250	47.6	35	13.6	3.5	0.3
79.8	26.1	32.8	185	25.2	68.6	5.8	0.1	0.3
78.4	26.9	34.3	298	60	30	8	2	0
76.6	25.5	33.2	222	53	29	14	0	0
75.6	26.5	35.1	157	61.8	26.8	11	0.1	0.3
76.2	25	32.8	225	53.2	34.9	11.3	0.2	0.4
78.7	26.2	33.2	159	28.1	62.9	7.2	1.2	0.6
79.7	27	33.9	444	76	16.7	6.3	0.6	0.4
81.8	27.3	33.4	222	19.4	66	14.2	0.2	0.2
79.8	26.8	33.6	207	22	44	10	0	0
83.4	28.6	34.3	509	47	48.5	4	0.5	0
75.7	24.6	32.5	339	60	33	5	0	0
100.3	34.5	34.4	394	45	45	10	0	0

87.1	28.8	33	317	74.8	15.2	9.8	0.1	0.1
78.4	25.8	32.9	311	74	19	7	0	0
72.2	23.7	32.8	181	43	41	14	0	0
71.4	23.6	33	253	65.3	25	8.8	0.7	0.2
78.8	27.2	34.6	266	48	46	6	0	0
92.8	30.4	32.7	424	38	59	3	0	0
87.1	28.9	33.2	177	53	38	9	0	0
79.8	26.7	33.4	136	72.8	13.6	12.7	0.6	0.3
80.5	27.2	33.8	174	48.3	38.8	12.5	0.2	0.2
82.8	27.4	33	171	38.4	47.3	13.6	0.5	0.2
89.3	30.5	34.2	408	16.4	68.4	8.4	6.1	0.7
79.4	27.6	34.8	163	62.9	31.2	5.6	0	0.3
77.5	26.7	34.5	140	26	69	5	0	0
77	25.8	33.5	250	32	56	10	1	0
78.7	27.2	34.6	154	47.1	44.2	7.3	0.7	0.7
77.5	26.6	34.4	205	72.2	21.2	6.4	0	0.2
79.7	26.9	33.8	289	35	57	7	0	0
75.6	25.7	34	238	70	23.2	6.4	0	0.4
77.8	26.7	34.4	148	38.9	50.5	9.5	0	1.1
94.3	33.2	35.2	416	43	44	10	2	0
81.6	28.3	34.6	541	55.4	34.3	7.9	2.2	0.2
74.5	25.1	33.6	273	8	73	13	0	0
78.8	25.8	32.7	219	33	49	15	2	1
80.5	27.8	34.5	418	35	54.8	9.4	0.4	0.4
96.6	32.4	33.5	370	68	21	8	1	0
85.7	28.8	33.6	292	35	56	9	0	0
85.9	28.2	32.8	304	45.8	35.5	14.3	4.1	0.3
82.3	28	34	268	58	23	16	1	0
85.2	28.9	34	247	36	55	9	0	0
76	26.6	35	203	42	44.2	11.3	2.2	0.3
79.7	27.3	34.2	278	69	22.2	4.5	4.1	0.2
78.5	26.3	33.5	179	68.3	22.7	8.8	0	0.2
78.1	26.8	34.3	137	66	26	7	0	0
89.8	31.6	35.2	351	54	33.7	9.5	2.6	0.2
85.6	27.9	32.6	374	52.6	41.2	5	1.1	0.1
77.5	26.8	34.6	485	19.1	66.8	9.5	3.6	1
80.7	27.6	34.2	219	87	8	5	0	0
80.7	27.3	33.9	148	36.8	55.7	7.2	0	0.3
83	27.9	33.6	482	66	19	14	0.5	0.5
74.2	26.1	35.1	231	36	43	20	1	0
96.2	33.7	35	284	54	35.5	9.5	0	0.5
86.9	29.6	34	265	50.9	40.2	7	1.6	0.3
78.2	26.9	34.4	321	79.9	15.1	4.7	0.1	0.2
73.5	24.5	33.3	265	57.4	31.4	9.3	1.6	0.3
91.7	30.9	33.7	549	37.8	52.7	8.4	0.8	0.3
75.6	25.2	33.3	276	85	11	4	0	0

80.2	26.9	33.5	336	39.7	46	12.9	1.2	0.2
78.4	25.2	32.2	489	45	46	8	1	0
80.6	27.7	34.4	190	58.3	35.3	6.4	0	0
81.4	27.9	34.3	197	62.2	28.2	6.1	3.3	0.2
79.5	27	33.9	152	41.6	42.7	14.6	0.2	0.9
78.8	26.9	34.1	222	71	16	12	1	0
72.9	25.8	35.4	282	4	74	16	2	0
97.5	34.7	35.6	310	40	42	11	5	0
87.4	29.4	33.6	442	75	19.3	5.4	0.2	0.1
82	28.1	34.3	271	76	17	5	1	0
78.7	26.5	33.7	289	30	59	11	0	0
81.3	27.3	33.5	266	56	33	11	0	0
79.5	28.1	35.4	177	44.6	44	6.9	3.9	0.6
100.2	34.1	34	403	43	43	10	4	0
90.8	31.9	35.1	381	22	72	5	1	0
80.5	26.5	32.9	232	34.5	59.4	5.7	0.2	0.2
79.2	26	32.8	500	79.5	14	4	1.5	0
74.1	25.1	33.8	237	56.1	34.4	6.5	2.5	0.5
79.5	26.3	33	205	44	43	11	1	0
81.4	28.3	34.7	184	53	34	13	0	0
75.8	25.6	33.7	387	81	11	7.5	0	0
79.1	27.2	34.4	292	65.4	25.3	8.5	0.6	0.2
84.3	28	33.2	255	41.6	49.4	7.1	1.4	0.5
78.6	26.8	34.1	181	30	53	15	0	0
79.4	26.1	32.8	195	50.6	40.5	8.1	0.2	0.6
75.7	25.6	33.8	265	69	24	6	0	0
78.4	26.5	33.8	157	62	25	7	0	1
74.8	26.2	35	307	45	49	6	0	0
77.8	26.1	33.6	456	27.5	57.4	11.1	3.9	0.1
76.8	25.9	33.8	270	50	34	16	0	0
84.9	28.9	34.1	195	74.3	16.3	9.1	0.1	0.2
79.2	26.8	33.9	163	55	35	7	0	0
76.7	26.3	34.2	183	33.3	54.2	12.1	0.1	0.3
77.1	25.8	33.4	176	68	26	5	0	0
65.2	20.8	31.9	224	72	12	14	1	1
81.5	26.8	32.9	283	44	42	12	1	0
77.6	27.1	34.9	174	76	15	8	1	0
76.7	25.7	33.5	233	41	53	6	0	0
87.2	30	34.4	604	23	67	6	4	0
81.7	29.1	35.6	215	56.7	29.9	10.7	2.3	0.4
77.8	26.7	34.3	177	57.2	34.5	7.9	0.2	0.2
81.2	27.7	34.1	225	31	56	11	1	0
77.3	26.6	34.4	367	77.7	19.3	1.6	1.2	0.2
76	26.3	34.6	278	22	74	4	0	0
74.4	24.5	32.9	321	49	42	7	2	0
78.2	25.3	32.4	212	32	58	7	0	0

77.5	27.2	35.1	249	19.5	66.3	9.6	4	0.6
77.1	26.1	33.9	181	53	35	9	3	0
68.4	21.3	31.2	445	57.7	33.2	8.1	0.8	0.2
80.2	26.1	32.6	188	79	13	4	0	0
106.2	36.2	34.1	247	50	36	14	0	0
78.1	25.8	33	245	27	56	14	0	0
79	27.5	34.8	309	86.5	8	4	1.5	0
80.4	28.9	35.9	298	48.5	45.1	5.3	0.5	0.6
70.2	23.2	33.1	310	54	34.9	10.4	0.4	0.3
79.4	27.4	34.4	450	56	27	15	1	0
75.4	25.2	33.4	360	20	65	12	1	0
78.5	26.8	34.1	243	65	20	15	0	0
74.7	23.9	32	310	51.4	35.5	11.7	1.2	0.2
76.8	26.3	34.2	298	64.1	26.7	4.9	4.1	0.2
72.9	24.3	33.3	208	22	67	10	0	1
80.1	27.8	34.7	157	32	58.2	7.6	1.8	0.4
95.2	32.3	33.9	320	47	34	9	10	0
77.2	25.8	33.4	175	45.1	41.5	11.4	1.4	0.6
81	28.1	34.7	329	53	35	12	0	0
79.1	27.4	34.7	487	53.6	37.7	7.7	0.9	0.1
71.8	23.5	32.8	458	39.4	47.1	8	5	0.5
79.8	28	35.1	113	31	45	20	1	0
75.7	23.8	31.5	383	42	54	3	1	0
77.3	25.2	32.6	265	37.3	52.9	8.7	0.3	0.8
81.4	27.1	33.3	125	47	43	9	0	1
75.4	24.8	32.9	203	25.6	59.3	14	0.6	0.5
74.2	24.7	33.3	310	45	44	7	4	0
80.2	27.4	34.2	247	80.4	8.6	10.3	0.3	0.4
74.9	25.1	33.5	570	57	31.5	9	2	0
88.3	31.3	35.5	325	38	50.8	9	1.9	0.3
77.2	25.1	32.6	198	24.8	65.2	9.1	0.6	0.3
75.6	26.3	34.8	362	61	27	7	5	0
89.6	31.7	35.3	283	17	70	6	7	0
75.8	25.7	33.9	122	51	34.3	12	1.1	1.6
68.4	22.7	33.2	183	56.4	30.1	13.1	0.2	0.2
79.9	27.3	34.2	232	49.8	41	8.6	0.3	0.3
80.7	27.5	34	266	47	41	10	2	0
76.9	25.3	32.9	156	55.9	34.2	8.2	1.2	0.5
73	24.5	33.5	201	50.5	34.6	12.9	1.4	0.6
75.1	24.9	33.2	193	74.9	15.9	9	0	0.2
79.1	26.1	33	431	34.7	54.3	9.9	0.3	0.8
76.3	26.1	34.2	222	65.2	26.7	7.3	0.6	0.2
76.3	26	34.1	174	30	60	8	0	0
79.7	27.3	34.3	326	75.5	14.4	9.3	0.1	0.7
79.3	26.8	33.8	226	70.8	21	7.8	0.1	0.3
102.2	33.9	33.2	416	26	60	7	6	1

84.6	28	33.2	247	58.4	30.1	11	0.3	0.2
80.2	26.7	33.3	161	29.1	64.7	5.7	0.1	0.4
78.4	25.3	32.3	236	59	28	13	0	0
77.1	25	32.4	123	33	56	9	1	1
76.7	26.3	34.2	240	30	68	2	0	0
69.3	23.5	33.9	263	69.2	20	8.6	1.8	0.4
72.5	23.8	32.8	260	79.6	11.8	8.4	0.1	0.1
77.8	25.6	32.9	190	67.5	24.5	7.6	0.1	0.3
78.6	26.1	33.1	124	25	66	5	2	0
82.3	27.8	33.8	380	61.4	26.5	10.8	1	0.3
77.1	25.6	33.2	278	56.3	29.8	12.8	0.8	0.3
106.6	35.1	32.9	455	34.8	49.9	11.9	2.5	0.9
78.3	26.6	34	204	46.4	45.4	7.5	0.1	0.6
95.7	32.1	33.5	248	79.5	14.9	4.8	0.6	0.2
95.9	32.4	33.8	347	76.2	18.5	3.2	1.8	0.3
79.5	26.3	33	262	69.9	20.1	9.2	0.7	0.1
78.5	26.8	34.1	259	34.2	55.8	9.2	0.5	0.3
74.3	25.7	34.6	422	58	31	9	1	1
72.8	24.7	33.9	226	64	27	8	0	0
81.6	28.1	34.5	359	46	42	10	1	0
93.2	30.7	33	348	25	52	18	5	0
89.9	30.6	34.1	492	26.8	60.3	7.2	5.5	0.2
76.6	26.1	34.1	388	46.4	41.1	10.2	1.8	0.5
80.9	27.4	33.9	187	49	43	7	0	0
81.8	27.1	33.1	133	56.4	33.7	9.5	0.2	0.2
81.5	27.5	33.7	262	32.1	56.5	5.9	5.2	0.3
81.6	26.9	33	286	73	19	7	1	0
77	25.9	33.7	147	44.5	42.3	12.6	0.3	0.3
98.1	32.8	33.4	317	53.3	35.5	10	0.8	0.4
81	26.9	33.2	351	58.5	33.5	6.5	1	0.5
74.8	25.5	34.1	259	51.9	39.5	7.9	0.3	0.4
80.8	26.9	33.3	153	40.8	49.6	9.4	0	0.2
79.8	28	35.1	310	53	30	17	0	0
81.8	27.4	33.5	505	31	60.4	6.2	2.2	0.2
75.5	25.5	33.8	255	60.9	29.9	8	0.6	0.6
74.8	25.6	34.3	149	20	69.4	8.7	0	1.9
77.8	26	33.4	175	62.8	29.4	6.3	1.1	0.4
84	27.3	32.6	258	43	46	10	0	0
79.4	26.2	32.9	280	44.7	43.8	10.6	0.5	0.4
83.7	27.4	32.7	509	33.5	52.5	12.2	1.3	0.5
78.1	25.2	32.3	353	53.2	41	4.9	0.6	0.3
77.4	26.4	34.2	313	57.7	38.7	2.9	0.7	0
80	27.2	34	441	54.8	38.1	6.5	0.5	0.1
79.3	27.2	34.3	140	19	74	5	1	1
74.4	25.2	33.8	237	41.2	46.6	11.4	0.4	0.4
81.6	26.5	32.5	161	57	33.8	8.2	0.6	0.4

77.5	26.3	34	105	44	46	10	0	0
78.6	26	33.1	260	74.1	18.2	6	1.5	0.2
82.7	28.5	34.5	245	53	39	6	0	0
92.7	31.9	34.4	496	50.7	39.2	5.7	4.1	0.3
73.3	24.4	33.3	239	29.3	56.7	9.8	4	0.2
83.5	27.7	33.1	322	68.8	20.8	10.1	0	0.3
80.9	27.7	34.2	301	49.7	41.3	5.2	3.6	0.2
76.2	24.5	32.2	416	62.7	21.5	14.7	0.8	0.3
88.2	30	34	318	53.7	41.9	3.6	0.7	0.1
79.6	26.4	33.2	378	76	20.8	3	0	0.2
79.5	27.5	34.6	464	39	53	6	1	1
73	24.2	33.1	280	59.5	31	8.2	0.9	0.4
79.7	27.6	34.7	148	43.8	43.1	10.4	2.3	0.4
74.9	25.6	34.2	215	58.1	39.5	2.1	0.2	0.1
81.6	28.7	35.2	112	21	67	8	3	0
80.8	27.8	34.4	144	54.6	35.5	9.3	0.2	0.4
79.6	27.1	34	110	35	48	9	2	0
73.1	24.6	33.6	215	50.8	40.3	8.1	0.4	0.4
74.5	24.8	33.3	268	37.3	55.5	6.3	0	0.9
79.9	26.9	33.6	284	39	52	7	1	0
80.2	27	33.7	653	26.4	62.8	8.3	1.9	0.6
96	31.5	32.8	278	39	43	13	2	1
79.5	27	33.9	505	63	28	9	0	0
105.6	35.7	33.9	196	56.9	29	9.6	4.2	0.3
76.8	25.9	33.7	197	20	59.2	14.4	5.6	0.8
83.3	28.3	34	327	44	41.4	13.8	0.6	0.2
79.3	26.1	32.9	210	54.4	38.8	6.5	0.1	0.2
79.5	27.2	34.2	199	64	22	12	0	0
90.8	31	34.1	423	31	53	12	3	0
75.7	25.2	33.3	292	48.3	33.4	12.7	5.4	0.2
81.5	27.3	33.4	224	78	12	6	0	1
81.1	26.7	33	192	28.2	64.7	6.2	0	0.9
78.8	27.5	35	182	62	26	12	0	0
82	28.2	34.4	232	85.4	7.9	6.3	0.3	0.1
81.6	26.1	32	712	51.5	32	14.5	2	0
79.1	26.9	34	291	46.3	42.4	10.6	0.2	0.5
76.5	25.6	33.5	320	61	35	2	2	0
88.8	29.6	33.3	274	30	46	22	1	0
76.8	25.9	33.7	315	58	25	16	1	0
74.8	25.6	34.2	152	58	33	9	0	0
80.4	27	33.6	242	51.4	37	10.7	0.2	0.7
72.3	24	33.2	262	76.3	18.8	4.8	0	0.1
92.6	30.1	32.5	563	37	46	15	0	0
71	23.8	33.5	214	44	40	16	0	0
75	25.2	33.6	101	35.3	54.9	7.5	2	0.3
84.6	27.7	32.8	441	55.5	38	4.5	2	0

74.3	25.5	34.4	185	3	89	7	1	0
83.6	27.9	33.3	301	57.3	31.2	7.4	3.9	0.2
73.1	23.4	32	321	72.5	22.5	5	0	0
77.3	25.8	33.3	350	10	82	7	1	0
76.7	25.9	33.8	158	48.8	35.9	14.2	0.8	0.3
79.1	25.9	32.7	214	21	71	7	1	0
80.5	27.4	34	196	56	26	15	1	1
77.8	27.8	35.8	304	76.8	18.2	4.6	0.3	0.1
76.4	25.9	33.9	412	59.2	31.9	8.1	0.7	0.1
82.6	27.9	33.7	269	57.1	35.9	5.9	0.3	0.8
74.6	24.5	32.9	325	78.8	12.6	7.9	0.4	0.3
80.1	27.3	34.1	207	57.8	33.2	8.5	0.2	0.3
78	26.1	33.4	210	39.5	47.4	10.9	1.1	1.1
83.1	28.2	33.9	485	39.9	49	9.4	1.5	0.2
85.9	28.5	33.2	592	32.1	50.9	13	3.6	0.4
83.3	27.8	33.3	357	70	17	13	0	0
77.2	26.7	34.6	389	34.8	57.9	5.2	1.7	0.4
75.6	25.6	33.9	289	53	25	20	1	0
86.8	29.8	34.3	272	61.9	29.5	6.1	2.2	0.3
80	27.1	33.8	396	53.1	43.3	2.9	0.5	0.2
71	23.7	33.3	147	23	72	3	0	0
77.9	25.9	33.2	356	53.5	40.3	5.9	0.2	0.1
75.4	24.4	32.4	167	48	40	8	0	0
77.8	26.2	33.6	162	29.4	58.3	12	0	0.3
81.6	27.2	33.3	225	44.9	44.3	10.1	0.2	0.5
92.9	31.1	33.4	330	41	47	11	1	0
79.2	26.9	34	203	64	20	15	1	0
77.6	25.4	32.7	221	65	27	8	0	0
85.3	28.5	33.4	223	63.6	27.6	5.9	2.7	0.2
84.3	28.4	33.7	204	57	31	8	2	1
77	26.8	34.8	324	73	22	2.5	2.5	0
77.9	26	33.4	417	58.8	31.4	8.6	1.1	0.1
100.5	31.7	31.5	334	76.5	15.5	3.5	3	0
81.3	26.2	32.2	438	62	23	10	0	0
104.3	36.8	35.3	477	42	35	19	3	0
80	27.5	34.3	271	43	39	16	1	0
79.4	26.3	33.2	163	38	51	10.8	0.1	0.1
77.9	26.1	33.5	261	65.8	20.8	12.5	0.7	0.2
74.7	25.1	33.5	361	83.7	13.9	1.7	0.6	0.1
80.8	26.8	33.1	171	46	48	5	1	0
84.9	29.3	34.5	244	22.9	68.7	4.8	3	0.6
79.6	26.5	33.2	211	60	32.9	6.9	0.1	0.1
80.7	27.3	33.8	188	60.4	29	9.5	1	0.1
79.3	26.8	33.8	179	50	36	13	0	0
76.4	26.4	34.5	208	66	24	10	0	0
79.3	26.7	33.7	249	50.6	43.3	5.7	0.1	0.3

77.6	25.9	33.4	337	60	26.5	10.5	0.5	0
84.4	28.7	34	147	70.1	18.6	10.1	0.9	0.3
82.4	27.8	33.8	211	21.9	68.3	7.5	1.7	0.6
80.9	27.5	34	230	79.2	14.6	5.8	0.2	0.2
76	26.6	34.9	275	68.5	24.1	6.1	1.2	0.1
79.8	26.8	33.6	216	37.6	56.4	4.8	0.4	0.8
79.8	26.4	33	242	79.4	11.6	8.9	0	0.1
76.1	25.4	33.4	347	11	71	15	2	1
80.5	27.1	33.6	134	27.2	63.1	8.6	0.7	0.4
79.9	27.4	34.3	261	25	51	3	0	1
71.5	22.3	31.1	144	40.9	48.3	9.2	1	0.6
78.1	27.7	35.5	354	40	48	9	1	0
74	24.2	32.7	175	66.8	24.5	8.3	0.2	0.2
82	26.6	32.5	179	43.5	48.1	7.5	0.6	0.3
80.1	27.6	34.4	207	71.9	19.8	8.1	0.1	0.1
72.5	23.1	31.9	101	16	70	9	0	0
84.6	28	33.1	160	56	30	13	0	0
79.6	26.4	33.1	443	55.3	34.4	10	0.1	0.2
82.3	27	32.8	202	45.9	42.5	10.5	0.7	0.4
77.9	27.9	35.8	252	36	51.5	6.9	5.3	0.3
82.8	28.1	33.9	184	74.8	15.3	9.6	0.1	0.2
79.8	27	33.8	236	80	8.3	9.1	2.3	0.3
85.1	28.5	33.5	263	37	53	7	0	0
84.6	29	34.3	381	81	9	2	0	1
79	25.8	32.6	297	47.4	43	8.3	1.1	0.2
78.6	25.8	32.8	112	49.1	45.1	5	0.5	0.3
83.4	27.2	32.6	193	29	67	4	0	0
84.6	28.7	33.9	192	57.1	34.3	8.4	0	0.2
81.4	27.4	33.7	269	82.3	9.4	4.1	3.8	0.4
76.7	26.1	34	203	70	18	11	0	0
77.6	26.6	34.2	270	50.7	36.3	12.5	0.2	0.3
88.8	29.1	32.8	162	68	13	15	1	0
79.4	26	32.8	113	27	60	10	2	0
74.8	27	36.1	258	85	9.5	4.1	1.3	0.1
78.7	24.6	31.2	204	56	39	4	1	0
84.6	29.9	35.3	204	76	18	5	0	0
66.8	22.1	33.1	414	40.6	42.5	11.2	5.5	0.2
81.8	27.1	33.1	125	38.9	47.3	11.7	0.8	1.3
80.3	27.7	34.5	291	58	31	7	0	0
78.7	26.4	33.5	211	55	37	8	0	0
67.5	21.1	31.3	215	64.4	21.5	12.6	1.1	0.4
82.3	28	34	191	35.2	57.1	6.9	0.4	0.4
72.9	23.4	32.1	296	61.7	26.9	9.2	1.9	0.3
78.6	26.1	33.2	287	31	60	9	0	0
78.5	26.8	34.1	258	42.9	48.8	7.7	0.1	0.5
85.7	28.1	32.8	491	68.3	24.7	5.8	1.1	0.1

77.6	25.9	33.4	283	56.9	29.8	12.9	0.1	0.3
88	29.4	33.4	214	61	25	8	0	0
76.7	26.2	34.2	371	20	72	8	0	0
76.4	26.4	34.5	246	50.5	41	7.1	1.1	0.3
75.1	25.2	33.5	346	68.6	18.4	11	1.6	0.4
80.3	27.7	34.5	205	45.8	44.6	9.3	0	0.3
81.5	29.3	35.9	182	27.5	65.6	6.5	0.1	0.3
79.3	26.7	33.7	225	31	61	8	0	0
91.2	29.8	32.7	523	66.2	19.1	13.7	0.8	0.2
79.3	26.4	33.2	122	61	29	5	0	0
79.2	27.3	34.5	223	49	37	13	0	0
81.7	27.4	33.5	260	38.7	53.5	6.6	1	0.2
83.8	28.5	34.1	448	60	28	10	1	0
72.5	23.6	32.6	158	30.3	57.3	11.8	0.4	0.2
74.8	24.6	32.9	292	56.2	32.9	8.3	2.3	0.3
76.9	26	33.8	220	22.2	70	6.9	0.5	0.4
79.1	27.2	34.3	228	70	23	7	0	0
92.1	31	33.7	161	16	76	7	1	0
80.6	27.2	33.7	294	71	23	6	0	0
88.5	29.8	33.7	179	3	85	11	1	0
71.8	23.1	32.2	233	49.8	41	8.6	0.4	0.2
76.3	26.1	34.2	171	40.7	47.7	11.3	0	0.3
95.2	32.5	34.2	649	12	45	17	0	0
81.4	28.3	34.8	337	80.4	13.3	6	0.2	0.1
77.5	26.5	34.2	180	48	42	10	0	0
79.9	26.3	32.9	315	53.2	29.9	12.2	4.3	0.4
82.3	27.5	33.4	297	72	12	15	0	0
88.4	30.8	34.9	280	70.7	22.3	6.6	0.1	0.3
77.7	26.6	34.3	210	42	44	14	0	0
82.7	28.2	34.1	272	85	8.2	5.7	1	0.1
85.9	29.1	33.8	437	65	20	13	1	0
91.5	31	33.9	532	30	50	11	8	1
80.5	27.7	34.5	395	74.6	18.9	5.2	1.1	0.2
73.7	25.5	34.5	266	47	42	10	0	1
79.2	26.8	33.8	215	61.5	27.6	10.6	0.1	0.2
70.6	23.4	33.1	131	35	49	14	1	1
73.6	23.9	32.5	282	73.3	16.9	9.2	0.2	0.4
80.8	27.4	34	190	47	36	16	0	0
79.5	26.7	33.6	222	61.9	30.3	7.6	0	0.2
78.2	25.3	32.4	324	42	44	13	1	0
80.4	26.5	33	238	41	47	9	3	0
77.8	26.8	34.5	178	64.8	22	12.8	0.2	0.2
80.3	28.2	35.1	350	70.8	23.3	5.7	0.1	0.1
81.1	27	33.2	228	42	40	17	0	0
77.3	24.9	32.3	301	10	84	5	1	0
77.9	25.5	32.8	370	74.5	21.9	3.3	0.1	0.2

76.5	26.8	35.1	299	44	48	7.3	0.5	0.2
80.9	27.2	33.6	254	71	15	14	0	0
78.1	26.3	33.7	394	66	24	10	0	0
89.2	29.7	33.3	513	62	29	6	0	0
76.3	25.6	33.5	161	52.6	35.9	10.8	0.5	0.2
81.5	28.2	34.6	413	51	42	6	0	1
80.2	27.9	34.8	304	38.5	48.1	11.5	1.7	0.2
79.9	26.4	33	448	66.5	23.5	9	0.5	0
79.8	27.2	34.1	441	76	16	7	0	0
75.1	25.8	34.4	320	54.9	36.9	5.6	2.4	0.2
71.9	22.6	31.5	857	22.9	63.6	6.3	6.9	0.3
85.8	29	33.8	243	39.2	47.4	9.7	3.4	0.3
77.4	25.9	33.5	207	58	31	10	1	0
75.9	25.4	33.4	258	70.5	20.5	8.6	0.2	0.2
77.8	25.9	33.3	534	19	74	6	0	1
78.4	26.6	33.9	173	46	44	10	0	0
77.6	26	33.4	341	56	24	18	1	0
80.9	26.7	33	494	60	36	4	0	0
77.6	25.9	33.3	262	22	57	18	2	0
82.5	27.9	33.8	260	56.2	35.7	6.3	1.5	0.3
79.3	26.1	32.9	485	71.5	25	3.5	0	0
89.8	30.2	33.6	316	54	30.9	8.1	6.7	0.3
99.5	33.4	33.6	213	41	37	17	5	0
80.6	26.9	33.4	244	57	33	9	0	0
79.6	26.6	33.4	196	43	50	4	0	3
75.7	26	34.3	216	75.9	19.3	4.5	0.2	0.1
77.4	26.1	33.7	164	55.8	33.3	9.9	0.5	0.5
98.7	33.1	33.5	383	42	45.5	7.8	4.3	0.4
80.2	27.7	34.5	238	72.5	20.4	6.9	0.1	0.1
82.6	27.5	33.3	152	41	39	16	2	1
82.1	27	32.9	412	39.8	50.1	7	2.7	0.4
81.6	28.4	34.8	203	83.2	11.8	4.9	0	0.1
76.7	26.4	34.4	124	39.3	53.3	6.6	0.2	0.6
77.7	25.6	32.9	252	36.2	48	7.4	8.1	0.3
80.2	27.1	33.8	139	10	76	7	3	1
101.6	34.1	33.6	317	52	40	7	1	0
82.5	27.9	33.9	321	46.9	42.5	9	1.3	0.3
79	27.2	34.4	438	56.5	38.5	5	0	0
78.9	26.3	33.3	147	59.7	31.9	8.2	0.2	0
80.1	26.4	32.9	505	28	59	11	0	0
80.5	26.6	33.1	267	70	23	6	0	0
79.1	26.7	33.7	232	60.7	33.6	5.3	0.3	0.1
86.6	29.6	34.2	371	39	42	16	2	1
79	28.1	35.6	249	31.6	57.9	9.6	0.6	0.3
74.1	25.5	34.5	213	35.1	54.1	10.1	0	0.7
80.4	27.6	34.3	179	54.2	37.2	7.4	1	0.2

101.5	35.2	34.7	229	70.5	19	7.5	1.5	0.5
83.1	28.1	33.8	213	67	21	11.9	0	0.1
79.1	26.4	33.3	207	55	30	15	0	0
87	29.1	33.4	380	37.3	55.4	4.1	2.2	1
83.6	27.8	33.2	158	71.7	15.5	12.6	0	0.2
79.9	26.7	33.4	203	50.9	37.9	9.4	1	0.8
78.4	26.4	33.7	273	58.6	33.1	6.7	1.2	0.4
80.5	26.5	32.9	222	69	23	8	0	0
75.5	25.2	33.3	307	58.6	30.8	9.4	0.8	0.4
80.6	26	32.3	295	61.4	29.6	6.3	2.3	0.4
83.6	26.9	32.2	236	84	10	5	1	0
95.2	32	33.7	262	25.1	58.1	13.6	3	0.2
102.3	34.7	33.9	272	41.2	44.2	8.7	5.2	0.7
79	26.3	33.3	234	29.4	63.1	7.2	0	0.3
88.8	27.6	31.1	358	30.3	61.8	7.1	0.4	0.4
78.2	25	32	174	40.6	48.6	10.2	0	0.6
85	28	33	383	63	32	5	0	0
76.6	26.4	34.4	343	37.1	50.6	8.6	3.2	0.5
77.5	25.5	32.9	257	51	47	2	0	0
87.7	28.7	32.7	395	45.9	40	13.4	0.5	0.2
84.2	28.1	33.3	405	64.8	27.6	6.6	0.8	0.2
76.2	26.1	34.2	189	29	49	16	5	1
85.3	28.6	33.5	132	21.2	68.4	9.9	0.2	0.3
74	23.9	32.3	152	43.4	49.1	6.9	0.1	0.5
80.7	27.4	33.9	278	54.8	36.2	8.9	0	0.1
77.3	26	33.7	172	40.4	48.6	7.9	2.7	0.4
69.8	22.9	32.8	257	54.8	35.7	8.8	0.3	0.4
76.8	26.1	34	334	61.5	20	14.5	0	0
80.7	26.5	32.8	196	54	38	7	1	0
81.7	26.3	32.2	252	45.9	45.6	7.6	0.7	0.2
94.2	32.1	34	301	18	68.8	8.7	3.5	1
80	26.8	33.5	141	14	82	1	2	1
85.9	28.8	33.6	179	38	38	22	2	0
75.7	24.9	32.8	162	31	56	11	1	0
75.4	24	31.9	159	64.8	24.4	10.8	0	0
79.8	27.1	34	243	72.1	22	5.8	0	0.1
79.4	26.2	33.1	198	39	41	16	1	0
78.9	25	31.7	87	66	26	8	0	0
79.5	28	35.2	140	11	84	5	0	0
79.6	27	34	278	59	37	4	0	0
79.1	26	32.8	402	45.2	42	11.8	0.6	0.4
73.5	24.6	33.4	429	7	91	1	1	0
79.2	26.4	33.3	244	68.4	22.7	5.9	2.9	0.1
82.2	27.9	34	473	65	29	5	1	0
77.8	26.9	34.5	181	39.9	48.1	11.7	0.1	0.2
86.1	29.4	34.2	430	53.1	33.7	10.4	2.6	0.2

76.1	26.8	35.2	406	47	32	15	3	0
90.6	30.8	33.9	318	47	35	17	1	0
89	30.6	34.3	450	49	36.9	10.5	3.4	0.2
74.1	24.1	32.5	14	4	93	0	0	0
76.3	25.9	33.9	194	35	53	7	1	1
77.7	26.6	34.2	192	60.6	29.7	7.9	1.6	0.2
77.2	26.1	33.9	337	70	10	11	0	0
78	26.4	33.8	329	61	18	16	3	1
79.7	27	33.8	263	49	36	15	0	0
77.8	26.1	33.5	184	52	37	11	0	0
78.6	27.4	34.8	200	73.7	12.3	13.2	0.3	0.5
75.5	24.7	32.7	584	50.5	45	3.5	0	0.5
75.9	23.9	31.5	350	86	10	4	0	0
73.6	24.5	33.3	151	49.1	32.2	11.9	6.2	0.6
83.4	28	33.6	195	28	63	8	0	0
75.2	26.1	34.7	190	40	54	5	1	0
80.7	28	34.7	247	41	45	11	2	0
81.3	26.5	32.6	214	7	85	7	1	0
78	26.1	33.5	100	31.9	61.7	5.2	0.2	1
94.2	32.2	34.1	285	44	34	18	3	0
79.5	26.8	33.7	163	66.1	25.7	7.7	0.2	0.3
76.7	26.5	34.6	196	44.8	45.6	9.2	0.2	0.2
76.7	27	35.2	250	22.9	62.3	14.4	0.1	0.3
75.5	24.9	33	268	82.2	12	5.3	0.3	0.2
97.9	33.3	34.1	418	51.5	40	6	1.5	0.5
86	27.9	32.4	615	43	51	4.5	1.5	0
80.6	26.2	32.5	241	56.6	28.6	13.2	1.1	0.5
81.2	27.4	33.8	197	35	41	12	1	0
82.4	27.8	33.7	325	43	37	18	2	0
76.7	26.4	34.4	256	24.5	69.1	5.2	0.7	0.5
78.8	26.5	33.6	292	57	29	10	3	1
83.4	28.6	34.3	190	69.6	21.5	8.7	0	0.2
81.2	26.7	32.9	257	73.8	19.3	6.3	0.3	0.3
79.7	27	33.9	205	61.7	25.2	12.4	0.2	0.5
75.7	24.7	32.7	205	49	42	8	0	0
82.9	28.1	33.9	297	39.5	47.9	11.3	1.1	0.2
75.5	25.8	34.2	392	64	22.5	11	1.5	0.5
78.7	26.7	33.9	203	16	74	8	0	0
80.7	26.5	32.8	212	39.6	51.9	7.9	0	0.6
72.3	23.6	32.6	172	63.2	27	9.2	0.5	0.1
76.9	27.1	35.3	156	63	28.9	7.7	0.2	0.2
84.1	28.3	33.6	229	35.2	52.1	11.9	0.5	0.3
80.2	27.2	33.9	278	58.5	27.5	13.4	0.3	0.3
70.6	22	31.1	622	33.8	55	7.2	3.6	0.4
74.4	24.8	33.3	292	70	22	7	1	0
86.5	28.7	33.1	364	43.9	41.3	11.5	2.7	0.6

78.6	25.9	33	228	16.2	66.4	9	7.8	0.6
75.4	24.8	32.9	308	76.5	15.4	8	0	0.1
82.5	27.8	33.6	315	32	63	4	1	0
78.5	26.7	34	428	46.6	44.1	8.8	0.3	0.2
81.6	28.1	34.4	269	58.6	28.3	8.4	4.2	0.5
76.7	26	33.9	303	59	24	17	0	0
71.5	22.9	32.1	185	32	52	14	0	0
88	30.4	34.5	358	47.5	36.8	14.7	0.8	0.2
75.2	24.3	32.3	275	39.8	49.7	8.7	1	0.8
81.2	26.7	32.9	112	16	69	9	1	0
78.6	25.7	32.6	318	45	45	6	0	1
84.9	28.2	33.2	213	49	42	9	0	0
79.2	26.6	33.7	228	21	76	3	0	0
78.9	27.2	34.4	276	84	6	5.5	0	0
81.6	27.2	33.3	219	55	33	7	0	0
79.3	26.9	33.9	271	76.4	13.1	8.4	1.9	0.2
79.6	27.6	34.6	289	60.2	28.4	10.8	0	0.6
76.6	26.1	34.1	289	42.6	47	9	0.7	0.7
76.2	26.6	34.9	221	61	31.3	7.5	0	0.2
83.1	28.5	34.3	247	63	30.1	6.6	0.1	0.2
74.9	24.9	33.2	182	72	22	6	0	0
83.2	27.6	33.2	142	55	35	8	0	1
80.9	26.7	33	223	9	78	8	5	0
80.4	27.8	34.6	224	49	37	10	0	0
78.2	26.1	33.3	319	61	26	10	0	0
84.4	27.9	33.1	224	48.5	39.8	6.4	5.1	0.2
78	25.3	32.5	185	15	78	3	0	0
77.6	26.4	34	321	52.5	38.4	7.9	1	0.2
77.6	25	32.2	275	70	17	11	0	0
78.3	25.7	32.8	244	22.7	68	6.6	2.2	0.5
87.1	29.9	34.3	224	32.1	52.2	13	2.1	0.6
82.4	27.7	33.6	190	53.6	37.9	5.5	2.8	0.2
84.1	27.3	32.4	223	45	19	20	0	0
74.3	24.7	33.2	304	40	53	5	0	1
76.6	25.3	33.1	230	49.7	37.6	12.3	0	0.4
90.6	31	34.2	261	40	34	22	3	1
82.7	28.3	34.2	206	56	29	12	1	0
90.1	30.5	33.9	496	52.7	37.4	8	1.7	0.2
80.6	27.9	34.6	275	80.5	10.4	5.8	3.2	0.1
67.5	21.7	32.1	145	54	27	19	0	0
77.7	25.6	33	204	64	24	6	0	0
79.9	27.4	34.3	253	37.9	52.2	8.9	0.5	0.5
87	29.8	34.3	552	65	23	6	5	1
84	27.4	32.6	320	44.7	47.5	5.2	1.8	0.8
78.7	26.5	33.7	202	39	50.1	9.8	0.7	0.4
80.7	26.8	33.2	304	71	22	5	1	0

79.6	25.6	32.1	419	19	52	21	1	0
84.4	27.7	32.8	344	30.4	61	7.9	0.4	0.3
74.4	25.6	34.4	350	24.1	69.4	3.8	2.4	0.3
81.6	27.4	33.5	170	19	74	5	0	0
77.8	25.9	33.3	322	39.2	49	8.8	2.7	0.3
83.8	29.9	35.7	269	64.1	28.3	7.4	0.1	0.1
78.2	26.3	33.7	415	33	57.3	5.8	3.5	0.4
70.4	22.9	32.5	329	60	22	16	1	0
77.4	26.8	34.6	166	87.7	6.2	5.9	0.1	0.1
79.4	27.3	34.3	208	75	14	5	1	1
78.7	26.5	33.7	240	57	33	9	1	0
76.3	25.9	34	215	37.8	49.7	11.9	0.3	0.3
76.4	25.1	32.9	390	48	40	11	1	0
81.9	27.2	33.2	258	39	52	9	0	0
82	27.8	33.8	240	76	21	2	1	0
78.2	26.5	33.9	231	49	39	11	0	0
83.2	27.5	33.1	287	26	63	7	0	0
80.2	27.3	34.1	295	58.5	33.2	7.6	0.2	0.5
79.8	26.8	33.6	123	37.1	42.1	14.3	6.1	0.4
80.4	26.2	32.5	203	47.8	42	9.7	0.4	0.1
80.3	27.2	33.9	222	66.5	16	15.5	0	0.5
81.1	27.9	34.4	133	3	93	4	0	0
84.7	28.6	33.8	261	55.3	35.3	7.8	1.2	0.4
76.7	24.4	31.8	165	65	24	11	0	0
79.5	27	33.9	315	69	27	2	1	0
77.1	25.7	33.3	236	81.1	10.7	5.2	2.9	0.1
75.5	25.2	33.3	292	53	40	5	0	0
84.5	28.4	33.6	152	19	72	5	0	0
79	27.2	34.4	129	41	45	11	0	0
83.3	27.2	32.7	230	32.8	53.9	10.7	2.1	0.5
77	25.7	33.4	166	32.6	55.2	10.4	0.5	1.3
70	21	29.9	377	73.5	19.8	4.9	1.4	0.4
79.9	26.2	32.8	266	49	42	5	1	1
81.3	27.8	34.2	201	41	35	19	0	0
76.6	24.9	32.6	217	29	61	9	0	0
82.4	27.2	33	251	67.5	24.5	7	0	0
80.9	27.7	34.3	265	52.7	37.6	8.6	1	0.1
80.8	27.1	33.5	228	26.3	61.9	11	0.5	0.3
105.4	36.2	34.3	310	41.8	41.2	13.9	2.6	0.5
78.9	27	34.2	166	39	47	8	1	1
98.7	33.7	34.1	337	29	50	17	3	0
79.7	26.4	33.1	151	34.9	51	9.5	4.1	0.5
79.1	26.9	34	234	74.7	16	9.1	0.1	0.1
81.3	27.6	33.9	290	38.1	51.3	10.1	0.3	0.2
78.6	26.5	33.7	158	19	67	13	0	1
80.9	27.7	34.3	177	38.2	50.4	10.6	0.4	0.4

81	28.4	35.1	387	84.5	11.5	4	0	0
78.8	26	32.9	166	74	20	4	0	0
77.5	26.4	34.1	253	68.2	23.4	7.8	0.4	0.2
75.3	26.4	35	349	55.1	33.4	11.3	0.1	0.1
81.8	26.9	32.8	257	43	41	16	0	0
76.8	25.4	33	243	67.3	21.2	11.4	0	0.1
77.5	27.3	35.3	222	36	17	45	0	0
78.3	26.8	34.2	300	71	22.3	6	0.6	0.1
81.8	28.2	34.5	198	74	18.7	6.6	0.5	0.2
81.6	28.6	35	231	57.2	31.8	9	1.5	0.5
78.3	26.8	34.2	457	67	23	9	0.5	0.5
84.8	28.2	33.2	116	43	34	14	0	0
78	25.6	32.8	318	84	12	4	0	0
81.1	27.5	33.9	278	61	31	6	0	0
81.3	26.4	32.4	259	82.6	8.3	8	0.6	0.5
74.7	25.7	34.5	549	21	70	4	2	0
82.9	27.8	33.5	178	33.5	56.8	8.7	0.4	0.6
82.7	27.1	32.8	270	57.4	36.1	5.1	0.9	0.5
86.3	29.2	33.8	462	12	82	2	4	0
65.9	19.9	30.1	194	64.2	28.2	7.4	0.2	0
77.7	26.7	34.3	266	68	21.5	8.5	0.5	0
88.2	30.1	34.1	378	19.3	67.1	9	4.4	0.2
79.5	26.1	32.9	244	76.3	11.4	11.7	0.1	0.5
79.8	26.1	32.7	145	25	70	4	0	0
96.1	32.6	34	414	51	29	13	6	0
84.7	29	34.3	353	61.4	27.9	9.4	1.1	0.2
86.1	28.7	33.3	326	33.2	54.5	11.6	0.4	0.3
87.5	28.3	32.4	396	54	38	8	0	0
78	26	33.3	300	49.1	38.7	11.7	0.3	0.2
86.1	28.4	33	208	42.8	47.2	9.4	0	0.6
76.4	25.5	33.3	346	4.5	72.5	15.5	0	0
104.3	35	33.6	198	46	34	18	0	0
105	33.8	32.2	235	60	29	9	2	0
102.8	35.6	34.6	215	66.6	15.4	14.8	2.6	0.6
83.8	28.1	33.6	146	47	41.8	10.6	0.3	0.3
71	23.3	32.9	155	50	35.6	12.3	1.7	0.4
78.3	26	33.2	342	56	29	10	2	0
99.7	34.7	34.8	382	48	31	19	2	0
81.7	28.4	34.7	259	67	20.5	11.5	1	0
79.2	26.3	33.2	374	45.5	42	9.5	1.5	0
79.4	27.3	34.4	167	49	30	17	0	0
82.3	27.6	33.5	177	48.3	38.7	11.8	0	1.2
72.8	23.7	32.5	309	36	47	13	0	0
78.9	26.2	33.2	134	20	55	19	0	1
82.5	27.3	33.1	243	44	40	11	0	1
78.2	26.2	33.5	131	25.7	65.6	8.1	0.2	0.4

80.8	26.9	33.3	227	65.4	24.8	8.6	0.9	0.3
88.2	29.5	33.5	411	28.5	58.3	10.7	2.2	0.3
81.6	27.4	33.5	220	59	35	6	0	0
91.6	29.7	32.4	351	42	38	17.5	2	0
84	28.1	33.5	262	54.5	30.9	14.1	0.3	0.2
82.5	26.5	32.2	144	24	65	7	0	1
89.7	30.3	33.8	359	50.6	39.3	9.1	0.7	0.3
85.4	28.3	33.2	421	20	64	7	9	0
79.7	26.2	32.9	297	38	43	16	1	0
82.7	27.8	33.6	223	48	35	13	1	0
76.3	25.9	34	254	73	15.4	11.3	0.1	0.2
78.4	27.5	35.1	258	54.5	30.9	14	0.2	0.4
78.9	26.8	34	256	67	21	7	0	0
89.8	30.5	34	621	67.5	22.5	7.5	0.5	0
75.9	24.1	31.7	316	53.2	33.8	10	2.7	0.3
78.2	27.2	34.7	263	61.9	30.5	6.8	0.6	0.2
79.5	25.6	32.2	301	60	27	9	4	0
75.8	26.1	34.5	316	41	44.5	12	0.5	0
93	31.3	33.7	380	37.6	44.1	10.6	7.4	0.3
73.9	24.4	33	218	40	45.4	13.8	0	0.8
81.2	27.6	34	191	61.5	26.6	11.2	0.5	0.2
83.5	28.1	33.6	303	42.1	48.1	9.3	0.2	0.3
83.1	27.3	32.8	107	9	87	3	0	0
79.7	26.5	33.2	164	45	42	11	1	0
79.9	27.6	34.5	289	53	37	10	0	0
86.2	28.7	33.3	228	52	30	14	0	0
75.8	25.5	33.6	359	44	44.4	7.3	4.1	0.2
81.6	28.3	34.7	212	59.9	34.4	5.2	0.3	0.2
87.3	27.2	31.2	249	46.1	42.4	8.9	2	0.6
79.4	26.5	33.3	256	72.5	20.5	7	0	0
81.1	26.4	32.5	206	41	33	24	2	0
74.1	25.1	33.8	362	26.1	57.8	13.1	2.3	0.7
80	26.5	33.2	334	42.2	47.4	9.4	0.7	0.3
73.4	24.5	33.4	285	30.2	58.7	11	0	0.1
77.4	25.9	33.4	140	50	42	5	0	0
65.9	20.9	31.7	364	42.5	56.8	0.5	0	0.2
79.1	27.6	34.9	253	49	31	16	0	0
81	27.2	33.5	154	29.3	60.8	8.7	0.5	0.7
81.2	26.6	32.8	202	41	47	12	0	0
80.5	27.6	34.3	200	62	24	2	0	0
86.1	27.1	31.4	141	41	45	1	3	1
81.7	27.4	33.5	120	36.5	51.7	11	0.5	0.3
74.3	24	32.3	262	28	61	7	1	0
83.3	27.6	33.2	181	57.4	31.7	10	0.4	0.5
83.5	28.5	34.1	294	59.7	31.6	8.3	0.1	0.3
80.6	25.7	31.8	186	47	40	9	1	0

78.3	25.3	32.2	155	27	62	6	0	0
80.1	26.9	33.6	219	30	42	13	1	1
81.5	27.6	33.9	221	19.8	67.3	10	1.8	1.1
79.1	26.5	33.5	343	80	11	7.5	0	0
71.4	21.8	30.6	231	46.4	45.9	6.9	0.3	0.5
80.6	27.3	33.8	106	18	69	10	2	1
71.6	23	32.1	123	27.7	66.4	4.9	0.7	0.3
78.3	26.2	33.5	158	85.7	9.2	3.8	1	0.3
85.9	28.2	32.8	174	49.1	41.3	5.7	3.2	0.7
78.5	27.2	34.6	157	22.8	65.3	10.6	1	0.3
91.9	30.7	33.5	84	47	31	17	0	0
82.4	26.5	32.2	181	46.6	46.6	6.5	0	0.3
87.4	28.5	32.6	443	61.9	32.3	5.3	0.3	0.2
76.2	25.1	32.9	364	52	39	7	0	0
81.2	27.1	33.3	208	73.3	20.6	5.8	0.2	0.1
80.1	27.2	34	243	28	62.6	8	1	0.4
75.3	24.9	33.1	189	35	43	0	0	0
91.4	32.2	35.2	314	50.4	35.8	8.4	5.1	0.3
81.4	27.7	34	212	63	25	8	0	1
80.6	26.5	32.9	275	65.5	21	11	0	0
83.7	28	33.4	227	72.6	20.5	5.9	0.8	0.2
80.5	25.7	31.9	224	54.5	31.5	13.6	0	0.4
80.8	27.2	33.7	127	32.2	61	5.6	0.1	1.1
77.9	25.4	32.7	275	67	20	9	0	0
83.3	27.1	32.6	103	42	36	19	0	0
80.7	27.3	33.8	127	43.9	49.7	5.8	0.3	0.3
73.7	24.1	32.7	269	71	20	7	0	0
81.1	26.9	33.1	167	61	21	10	0	1
79.6	26.8	33.6	106	60.1	30.5	8.5	0.6	0.3
78.5	25.9	33	202	55.1	37	7	0.7	0.2
73.8	22.8	30.9	471	41	55	1.6	2	0.4
80	26.8	33.4	145	19	70	7	0	1
77.9	25.3	32.5	236	46.2	40.8	12.7	0.1	0.2
80.9	26.6	32.9	148	55.3	38.7	5.4	0.3	0.3
85.8	28.2	32.9	310	54	36	6	1	0
78.9	26.9	34.1	196	39	46	13	0	0
80	26.6	33.2	380	60.6	31.6	7.4	0.2	0.2
82.2	27.3	33.2	209	43.6	45.5	9.8	0.6	0.5
76.7	25.4	33.1	343	85	9	2	1	0
81	26.7	33	288	69	23	8	0	0
79.8	26.7	33.4	292	60	25	10	0	1
84.2	27.6	32.7	177	55.2	32.5	11.2	0.8	0.3
80.4	27.3	33.9	353	42.3	47.8	9	0.6	0.3
80.3	27.6	34.4	294	77	14	9	0	0
95.7	32.3	33.8	410	38	44	11	4	1
83.9	27.2	32.4	226	30	53	13	0	0

71.3	22.9	32.1	116	49.8	38.7	9.2	1.8	0.5
79	26.5	33.5	234	28	59	5	0	0
76.8	24.8	32.3	245	18.2	67.5	11.6	1.6	1.1
70.4	22.9	32.6	207	33.2	56.2	9.2	0.9	0.5
92.7	31.1	33.5	167	30	57	9	2	0
78.2	27	34.6	321	21	59	15	2	1
81.4	28.2	34.6	148	21.7	68.3	9.4	0.3	0.3
82.2	26.7	32.4	370	13	73	11	2	1
83.2	27.6	33.2	267	46.4	39.6	13.3	0.4	0.3
84.7	28.9	34.1	389	47.7	44.6	5.5	1.9	0.3
81.1	27.1	33.4	238	70.1	23.5	5.3	0.8	0.3
82.2	26.5	32.3	229	63.8	23	11.6	1.5	0.1
78.6	27	34.4	178	19	65.3	11.6	3	1.1
80.7	27.6	34.2	160	59.4	33.5	6.1	0.8	0.2
77.6	24.9	32.2	178	22	74	4	0	0
91.7	28.9	31.6	443	59	33.5	6.5	0.5	0
70.2	22.3	31.7	134	55.1	32.8	11.6	0.2	0.3
74.2	25.1	33.8	226	29	67	3	0	0
75.1	25.6	34	135	26.4	61.2	12.2	0	0.2
87	28.5	32.7	191	34	54.5	11.2	0	0.3
90.5	31.1	34.4	505	20.4	66.4	9.1	3.5	0.6
77.4	26	33.6	199	45.7	44.7	9.4	0	0.2
83	27.8	33.5	186	61	34	3	0	0
78	26.6	34.1	197	39.4	49.7	10.2	0.6	0.1
72.5	24	33	208	50.6	46.4	2	0.4	0.6
82	27.7	33.8	235	61	25.1	5.2	8.5	0.2
76.9	26.6	34.6	263	29.5	64.5	4.3	1.3	0.4
80.5	27.8	34.5	196	38.4	53.2	7.7	0	0.7
81.6	28	34.3	214	45.1	42.9	9.6	2.2	0.2
76.5	25.6	33.4	212	38.3	49.7	10.8	0.4	0.8
75.7	25.7	34	456	48	27	19	1	1
72.7	24.3	33.4	360	78	14	7	1	0
80.6	28.1	34.8	166	80.1	12.3	7.4	0.1	0.1
76.8	26.2	34.1	278	81.8	10.5	7.6	0	0.1
74.4	24.5	33	349	70	20	7	0	0
66.3	21.5	32.4	419	52	39	5	1	0
78	25.5	32.6	324	61	27.6	7.4	3.8	0.2
81.5	28.3	34.7	268	72	23	4	1	0
76.8	26.1	34	325	57	26	13	1	0
76.1	26.5	34.8	262	72	18	6	2	0
74.4	25.2	33.9	219	88	9	3	0	0
79	25.8	32.7	250	60.9	32.8	4.9	1	0.4
80.4	27.9	34.7	166	41.3	48.4	9.2	0.9	0.2
78.6	26.6	33.8	184	53.9	30.1	11.5	4.1	0.4
80.9	27.2	33.6	138	66	19	15	0	0
65.8	20.7	31.4	280	30	46	22	0	0

82	28.1	34.3	236	66	12	20	1	1
82.8	28.2	34.1	145	80.8	12.7	6.3	0.2	0
80.5	26.5	32.9	234	65	17	16	0	0
77.9	24.2	31.1	238	60	30	5	0	0
84.8	28.9	34	91	47	35	16	0	0
75.8	26.3	34.7	189	81	4	5	0	0
87.4	29.6	33.8	181	76.8	14.7	7.7	0.6	0.2
78.1	26.9	34.4	315	80.5	13.1	6.2	0.1	0.1
78	26.8	34.3	204	78.6	11.4	9.8	0.1	0.1
80.5	27.7	34.4	164	73.6	15.7	10.3	0.1	0.3
79.2	27.3	34.5	313	84.8	10.4	4.5	0.2	0.1
81.9	27.3	33.3	139	27.3	63.7	6.6	2	0.4
82.3	27.9	33.9	235	73	18	9	0	0
79	27.5	34.7	308	53.1	39.7	6.4	0.4	0.4
78.4	26.6	33.9	137	71.6	20.6	6	1.1	0.7
82.4	27.3	33.1	214	63.5	31.6	4.2	0.3	0.4
81.7	27.4	33.6	172	41	41	13	4	1
80.2	26.7	33.3	181	69.2	25.4	4.1	0	1.3
81.2	27.4	33.8	224	74	19	6	0	0
80.4	27.6	34.4	263	78.4	15.1	5.9	0.3	0.3
77.7	25.7	33.1	375	78	15	6	0	0
82.8	28	33.9	171	78	12.7	9.2	0	0.1
79.1	25.6	32.3	367	69.3	18.9	8.9	2.7	0.2
81.5	26.6	32.6	275	64.9	28.2	6.5	0.1	0.3
77.7	26.9	34.6	456	80.1	12.1	6.4	1.3	0.1
83.4	28.4	34.1	364	78	12	9	1	0
78.1	26.6	34	271	63.8	24.6	11.4	0	0.2
82.6	27.9	33.8	194	78.2	12.8	8.7	0.1	0.2
80.2	28.1	35	255	71.5	21.2	4.8	2.3	0.2
80.9	28.2	34.9	306	74.8	17.2	7.5	0.4	0.1
79.1	27	34.1	179	66.8	20.5	12.4	0	0.3
78.8	27.9	35.4	335	73	21	6	0	0
78.4	27	34.4	277	70.3	24.7	4.6	0.2	0.2
81.9	28.3	34.6	318	72.3	21.8	5.7	0.1	0.1
77.4	25.5	32.9	277	50.3	38.7	8.1	2.2	0.7
75.9	26.5	35	217	77.8	16.7	5.3	0.1	0.1
78.2	26.9	34.4	327	88	9	3	0	0
79.3	27.4	34.6	422	32.4	63	3.8	0.4	0.4
85	27.3	32.2	182	66.2	26.8	6.8	0	0.2
78	27.2	34.9	279	70	24	5	0	0
78.2	26.4	33.7	192	60.8	29.4	5	4.5	0.3
81.7	28.2	34.5	449	71.2	21.1	7.2	0.3	0.2
80.9	27.7	34.2	157	62.5	31.7	5.4	0.1	0.3
79.2	27.3	34.5	203	80.5	11.4	7.3	0.6	0.2
79.6	26.3	33	184	69.2	16.3	14.2	0.1	0.2
77.8	26.4	33.9	257	74.9	20.3	4.6	0	0.2

80.5	29	36.1	353	87.5	6.5	5.5	0.5	0
78.8	27	34.2	372	60	30	7	2	1
82.6	27.6	33.4	161	83.3	10.4	4.5	1.7	0.1
83.6	28.3	33.9	219	84.2	9.3	6	0.3	0.2
80.7	28.3	35	315	66.4	27.5	5.5	0.5	0.1
79	27.2	34.5	218	65	28	7	0	0
85.6	28.5	33.3	275	91.3	3.8	4.8	0	0.1
85.7	29.3	34.2	257	68.1	22.9	8.5	0.1	0.4
84.6	29.5	34.9	215	84.7	5.7	9.3	0.1	0.2
85.6	28.5	33.3	287	55	31.8	12.2	0.2	0.8
79.2	26.5	33.4	300	77.2	19	3.7	0	0.1
81	27.5	33.9	264	88	6	6	0	0
79.4	26.7	33.6	213	62.8	30.4	6.7	0	0.1
78.8	26.3	33.4	131	59.3	34.4	5.9	0.2	0.2
81.5	27.5	33.7	314	89.1	6.8	3.5	0.5	0.1
81.2	28.4	34.9	216	71.2	21.7	6.9	0	0.2
80.2	27.3	34.1	221	73.8	20.6	5.3	0.2	0.1
80.3	27.1	33.7	279	75.9	13.2	10.3	0.3	0.3
80.8	28.2	34.9	132	49	41.8	8.4	0	0.8
86.7	29.6	34.2	365	83	10	4	0	0
81.6	27.7	34	249	47	33	13	4	0
77.9	26.6	34.1	326	42.6	45.8	9.3	0.6	1.7
78.7	26.9	34.1	178	62	24.8	12.8	0.2	0.2
86.7	28.6	33	483	66.2	26.7	5.8	0.7	0.6
84.7	28.3	33.4	210	52.7	35.6	7.3	4.1	0.3
79.1	25.5	32.2	222	44.9	42.8	7.8	4.5	0
81.2	28.5	35.2	232	77	12.9	9.8	0.1	0.2
79.4	27.2	34.2	347	62	25	13	0	0
76.3	24.9	32.6	205	62.4	32.7	3.8	0.8	0.3
81.3	28.3	34.9	331	81.6	14.9	3.2	0.2	0.1
82.6	27.7	33.5	290	84	11	5	0	0
74	24.9	33.7	296	66	25.4	7.5	0.9	0.2
81.9	27.9	34.1	252	66.8	21.2	5.1	6	0.9
85.3	27.9	32.8	192	73.7	20.3	5	0.7	0.3
79.9	26.9	33.7	423	84.5	6	9	0	0
72.3	24.3	33.6	287	84	7	9	0	0
76.7	26.2	34.1	290	74.2	17.6	6.2	1.8	0.2
79	26.5	33.5	368	69.5	22	8	0	0
81.1	26.9	33.2	352	87	8	4	0	0
80	26.7	33.3	459	67	20.5	12.5	0	0
84	29.3	34.9	143	89	7.8	3	0.1	0.1
80.4	27.3	33.9	278	73.4	21.9	4.6	0	0.1
70.5	23.2	32.9	116	69.1	22.5	7.6	0.7	0.1
79.5	26.7	33.5	205	68	22.8	8.8	0.3	0.1
82.5	28.4	34.4	248	74.7	20.3	4.8	0	0.2
77.9	26	33.4	251	50	31	14	0	0

75.9	24.9	32.8	217	48.6	41.1	7.1	2.6	0.6
80.4	26.7	33.3	327	78.5	15	5.5	0	0
78.5	28	35.7	234	74	17	9	0	0
83.9	29	34.6	248	42.4	37.8	3.7	15.8	0.3
80.8	26.9	33.3	191	64	19	8	0	0
76.1	25.4	33.4	523	77	14	6	1	0
78.2	27.2	34.8	194	73.8	18.4	4.5	3.2	0.1
82.9	28.8	34.8	231	86	6	7	0	0
82	27.3	33.2	331	73.3	18.5	7.2	0.6	0.4
78.8	26.9	34.1	286	74	13	13	0	0
77.9	25.4	32.6	189	50.6	43.1	5.6	0.2	0.5
82	26.7	32.6	261	78.2	12.7	8.8	0.2	0.1
80	27.3	34.2	220	57.7	31.1	11	0.1	0.1
79.2	26.6	33.5	141	46	37	7	0	0
77.8	27.5	35.4	244	65.9	21.9	10.8	0.9	0.5
86.4	30.2	34.9	208	78.2	14.5	7	0.1	0.2
85.6	29.2	34.1	230	49.9	42.5	7	0.1	0.5
75.3	26.6	35.3	239	60.9	27.4	10.2	0.8	0.7
77.8	26.4	33.9	296	75	17.7	6.4	0.6	0.3
79.6	28.4	35.7	159	50.9	38.1	9.5	1.1	0.4
83.4	27.6	33.1	208	72.5	21.6	4.8	0.7	0.4
79.1	28	35.4	211	48.9	41.1	8	1.7	0.3
86.7	28.7	33.2	297	53	40	6	1	0
79.9	25.9	32.4	376	83.4	11.8	4.6	0	0.2
75.6	26.9	35.6	211	62	21	13	0	1
83.3	27.9	33.5	271	60.4	30.7	7.1	1.6	0.2
75.8	26	34.3	168	73	15	6	0	0
81.8	27.9	34.1	167	71.2	19.8	8.6	0.3	0.1
80.3	27.5	34.3	296	64	27.3	7.3	1.2	0.2
80	26.8	33.5	279	83.7	12.5	3.6	0	0.2
84.2	28.7	34.1	167	47.5	43.4	6.4	2.5	0.2
76.6	26.8	35	220	56.9	30.3	12.1	0.4	0.3
82.3	27.6	33.5	426	64	28	6	1	0
78.9	28.4	35.9	355	61.9	22.7	15.1	0	0.3
77.7	25.5	32.8	224	57.1	29.6	12.1	0.9	0.3
80.6	26.6	33.1	264	63	18	13	0	0
80.2	26.7	33.3	255	57.3	34.2	8	0.4	0.1
79.2	27	34.1	229	64.6	24.7	5.7	4.1	0.9
80.4	27.6	34.4	336	80.9	12.8	6.1	0.1	0.1
80.4	27.7	34.4	311	87.2	6.7	5.4	0.6	0.1
78.9	26	33	253	83	15	2	0	0
78	27.2	34.9	262	47.1	38.1	14	0.3	0.5
68.7	22.3	32.4	101	44.1	46.4	5	4.3	0.2
75.2	25.9	34.5	146	43.7	39.5	16.2	0.3	0.3
77.9	26.9	34.5	289	89.2	6.4	3.3	1	0.1
79.3	26	32.8	274	77.5	12.2	10.2	0	0.1

89.2	31.6	35.4	370	94	4	1	0	0
77.7	26.9	34.7	230	67.5	22.7	8.9	0.5	0.4
76.7	26.2	34.2	260	70	21	6	2	0
84.2	27.7	32.9	216	74	15	6	1	0
80.6	27.9	34.6	227	72.8	17.9	9	0	0.3
81.5	28.1	34.5	269	74	16.9	2.9	5.8	0.4
80	27.5	34.4	155	60.5	30.7	8.4	0.2	0.2
79.1	27.1	34.3	227	62	28.8	6.6	2.3	0.3
78.6	27.2	34.6	291	75.3	19.2	4.5	0.6	0.4
81.1	27	33.3	327	74	10	12	0	0
84.7	28.3	33.4	407	66	31	3	0	0
81.9	27.3	33.3	161	76	17.1	6.6	0	0.3
72.4	23.5	32.4	291	35	55	9	0	0
79	27.4	34.7	326	69.5	20.3	8	2	0.2
83.5	28.5	34.2	375	66	25	8	0	0
81.4	28.9	35.4	244	22	56	19	1	0
76.5	27.4	35.9	61	29.8	61.4	7.5	0.5	0.8
74.6	24.6	33	354	63	28	9	0	0
83.4	29.7	35.6	385	37	40	20	3	0
77.5	26.7	34.5	368	71	17	10	2	0
76.9	26.7	34.7	156	40.9	49.5	8.1	0.9	0.6
82	27.1	33	239	74.6	15.6	9.3	0.3	0.2
82.5	27.6	33.5	131	78.2	15.3	6.1	0.3	0.1
77.3	26.2	33.8	228	50.6	38.7	8.9	1.4	0.4
78.1	26.4	33.8	259	61	30.4	7.7	0.7	0.2
71	23.5	33.1	181	67.6	22.1	10.2	0	0.1
66.9	23.6	35.3	345	44.8	46.8	7.1	0.1	1.2
79.6	25.6	32.1	142	71.9	17.1	10.8	0.1	0.1
86.9	29.3	33.7	326	83	10	5	0	2
80.8	27.9	34.6	164	78.1	14.4	6.5	0.7	0.3
82.7	28.7	34.7	268	67.1	19	13.6	0.1	0.2
84.7	28.5	33.7	201	75.9	17	6.7	0.2	0.2
79.4	28.2	35.5	363	93	1	6	0	0
79.6	26.2	33	178	64.7	25.2	9.4	0.4	0.3
77.1	26.2	34	327	57.2	34.2	8.3	0.1	0.2
80	27.7	34.6	184	51	20	17	1	0
81.7	27.7	33.9	246	68.2	21.7	9.2	0.8	0.1
78.7	26.9	34.2	194	58.2	35.2	5.9	0.4	0.3
80.7	27.8	34.5	241	73.4	20.6	5.8	0	0.2
77.6	26.4	34	273	58.8	32.6	8.1	0.2	0.3
82.1	27.9	34	184	68.4	20.5	10.9	0.1	0.1
79.6	26.9	33.8	237	57.1	33.6	9.1	0	0.2
77	25.3	32.9	323	74.5	18.8	6.1	0.4	0.2
81.8	27.5	33.6	250	38	46	9	6	1
79.6	27.2	34.2	139	53	29	18	0	0
77.2	26.5	34.3	240	1	77	19	0	0

81.8	28.6	35	312	71.6	21.9	6.1	0.2	0.2
77.8	26.6	34.2	149	74.1	21	4.5	0.3	0.1
78.5	26.5	33.7	246	56	37	7	0	0
74.1	25.5	34.5	230	35	47.9	16.2	0.7	0.2
76.3	26	34.1	329	79.4	15	5.3	0.2	0.1
73.6	25.6	34.7	253	78.4	15.8	5.4	0.2	0.2
74	25	33.8	204	66.4	26.4	7	0.1	0.1
80.8	28	34.6	236	60.6	29	9.5	0.6	0.3
75.3	26.6	35.4	386	50.8	36.3	11.8	0.6	0.5
80.9	27.9	34.5	151	36.6	51.3	10.5	0.9	0.7
81.6	28.5	34.9	275	28	59	12	1	0
82.1	28	34.1	184	77.2	12.5	10.1	0.1	0.1
84.4	27.6	32.7	214	64.1	25.7	9.4	0.5	0.3
75.6	25.7	34	383	61	25	14	0	0
84.5	28.4	33.6	193	81.5	10.7	7.6	0.1	0.1
82	27.2	33.1	215	44	51	5	0	0
80.7	27.5	34.1	302	72.4	22.7	4.2	0.5	0.2
74.2	26	35	211	83	9	7	1	0
76.3	25.6	33.6	329	44.1	49.7	4.8	0.2	1.2
80	27.6	34.5	198	63	27.6	7.9	0.2	1.3
84.9	29.2	34.4	283	74.3	17.8	5.9	1.8	0.2
81.3	28.2	34.7	216	58.1	34.3	7.1	0.3	0.2
76.2	25.4	33.3	363	93	5	2	0	0
78.5	26.5	33.8	259	53	34	12	0	0
75.1	25.8	34.4	195	57.4	34.1	8.1	0.2	0.2
81.7	26.8	32.8	225	58.2	31.5	9.6	0.4	0.3
77.9	26.4	33.8	207	79	11	8	0	1
76.6	27.1	35.4	471	75.9	17	6.3	0.6	0.2
81.4	28.2	34.6	320	75.6	18.5	5.3	0.5	0.1
73.8	24.5	33.2	210	70.1	20.8	8.6	0.3	0.2
74.5	24.9	33.4	409	73.5	18	7.5	0	0
79.1	27.8	35.1	312	32.9	53.8	10.6	2.1	0.6
78.8	27.4	34.8	494	82	12.5	5.1	0.1	0.3
76.6	26.3	34.3	248	72.6	18.6	8.5	0.1	0.2
79	27.5	34.8	287	54	34	11	0	0
74.9	25.5	34	310	72	14.7	13.1	0.1	0.1
80.9	26.7	33.1	209	67	27	5	1	0
78	25.9	33.1	253	68.3	19.3	12.2	0	0.2
78	26.5	33.9	172	67	23.5	9.3	0.1	0.1
78.7	27.6	35	193	73.7	11	15.1	0	0.2
82.6	29.1	35.2	271	56.6	33.6	5.6	3.4	0.8
76.1	26.1	34.3	222	75	18	7	0	0
80.7	28.4	35.2	278	63.1	27.2	9.3	0.2	0.2
82.5	26.8	32.5	403	62	26	12	0	0
79.7	26.8	33.6	303	66	19	11	2	0
84.7	28.4	33.5	349	62	30	8	0	0

76.9	27.3	35.5	346	56	27	15	1	1
81.9	27.3	33.3	245	71.2	19.2	7.6	1.8	0.2
81.1	27.3	33.6	280	68	20.9	10.1	0.1	0.9
73.5	25.3	34.3	146	51.2	38.9	9.1	0.6	0.2
84.8	28	33	203	80	14.8	5	0	0.2
78.3	25.8	33	307	70.1	25.1	4.2	0	0.6
80.5	26.6	33.1	368	81.2	13.3	5.4	0	0.1
78.2	26.7	34.1	179	70.6	22.8	6.3	0.1	0.2
81.6	28.2	34.5	147	42.7	46.2	10.5	0.2	0.4
84.5	29	34.3	202	49	26	14	0	0
79.6	26.5	33.3	212	84	12	4	0	0
77.2	26.8	34.7	268	54.5	39.9	4.6	0.6	0.4
81.7	28.4	34.7	174	67.3	25.2	7.3	0.1	0.1
80.5	27.1	33.7	175	38	53.3	6.7	1.9	0.1
78.3	25.9	33.1	361	68	25	7	0	0
78.3	26.5	33.9	198	51.8	38.9	8.8	0.1	0.4
80	28.6	35.8	229	54.8	35.4	9.6	0.1	0.1
75.5	27	35.7	243	46	46	5	3	0
78	26.5	34	449	81	14	3	0	0
81.2	27.8	34.3	185	46.4	46.7	6.3	0.2	0.4
76.3	26.6	34.8	151	74	22	2	0	0
78.5	26.8	34.1	287	67.9	18.8	8.3	4.5	0.5
90.4	30.1	33.2	384	61.4	32.8	4.6	1	0.2
80.8	27.3	33.8	163	88	7	5	0	0
79.1	26.5	33.5	349	71.7	17.8	9.5	0.8	0.2
82.3	27.4	33.2	172	82.5	10.5	5.5	1.3	0.2
93.2	31.5	33.9	432	33	51.4	11.3	3.7	0.6
79.5	28.7	36.1	261	44	45	8	3	0
74.7	24.8	33.2	214	31	48	8	0	0
84.1	28.3	33.6	170	40	40	16	0	0
78	26.8	34.4	210	64.5	22.6	11.9	0.8	0.2
79.6	26.7	33.6	254	27.7	58.4	13.1	0.4	0.4
81.8	27.6	33.7	323	42	47	9	1	1
72.5	24	33.1	296	82	11	6	0	0
89.5	29.8	33.3	84	42	43	15	0	0
75.1	25.2	33.5	209	66.2	24.5	8.8	0.3	0.2
80.2	27.8	34.7	179	72.2	19.8	7.8	0	0.2
80.2	28.5	35.5	346	70.5	18.2	11.2	0	0.1
81	26.3	32.5	186	64	27	9	0	0
80.9	25.7	31.8	363	57.3	30.8	9.4	2.3	0.2
84.6	28.4	33.5	266	78	15	5	0	0
76	26.2	34.4	175	56.4	35.8	7.3	0.1	0.4
75.6	25.5	33.7	226	63.2	28.1	8.2	0.2	0.3
66.4	22.2	33.4	302	84.2	11	4.3	0.2	0.3
77.5	26	33.5	133	24.5	62.2	12.2	0	1.1
82.7	27.4	33.1	283	75.4	16	7.8	0.7	0.1

79.6	26.8	33.6	236	74	18.3	7.1	0.3	0.3
83.1	28.2	33.9	172	78.2	15.4	5.8	0.1	0.5
81.6	27	33.1	216	48.9	41.8	8	0.9	0.4
78.8	26.8	34	273	53.1	38	7.3	1.3	0.3
76.8	26.7	34.7	227	53.8	35.9	8.8	0	1.5
80.7	28	34.6	243	68.3	20	7.8	3.4	0.5
78.5	27.6	35.1	248	56.8	28.5	13.3	1.2	0.2
74.5	24.9	33.4	451	80.8	12.5	6.4	0.1	0.2
81.9	28.1	34.3	611	78.5	14.5	4.5	0	0
82.7	26.6	32.1	248	74.5	16.5	6	0	0
79.5	25.5	32	269	38.7	49.5	9.1	2.6	0.1
80.2	27.3	34	199	40	41	7	0	0
80	27.6	34.4	278	76	17.8	4	2.1	0.1
76.7	26.8	34.9	280	74.7	15	8.2	2	0.1
75.8	25.7	33.9	171	69	29	2	0	0
80.5	27.7	34.4	239	53	40	7	0	0
78.6	27.9	35.5	559	65	28	7	0	0
82.3	26.8	32.6	215	77.2	16.2	6.5	0	0.1
84.1	29.8	35.4	163	27	60	13	0	0
83.6	27.9	33.3	201	79.1	16.8	3.9	0	0.2
73.3	24.9	34	192	77	11	10	0	0
79.4	27.7	34.9	331	68.7	23.4	6.7	1.1	0.1
81.8	27.7	33.9	295	85.1	8.3	6.4	0.1	0.1
75.5	25.8	34.1	274	62	33	5	0	0
72.9	24.8	34.1	329	47.5	41.5	8.1	2.6	0.3
81.1	28.5	35.2	188	28.7	63.2	7.7	0.2	0.2
78.3	27.6	35.2	268	70.1	19.4	10.2	0	0.3
81.6	27.4	33.5	174	38.2	46.7	11.8	0.9	2.4
78.2	27	34.5	230	74	18.4	7.5	0	0.1
72.7	24.4	33.5	144	62.6	26.2	10.9	0	0.3
81.9	29.1	35.5	172	54	32	14	0	0
77.2	26.8	34.8	216	50.1	37.2	11.6	0.1	1
82	27.7	33.8	300	63	31.1	4	1.5	0.4
78	26.3	33.8	162	37.3	54	6.8	0.2	1.7
70.6	23.3	33	278	53	30	16	0	0
73.7	24.1	32.7	295	70.1	17.9	11.5	0.3	0.2
84	27.4	32.6	449	57.3	35.8	5.6	1.1	0.2
69.1	23.6	34.1	302	80	11.4	8.2	0.2	0.2
77.4	26	33.6	339	38.2	47.8	12.3	0.7	1
80.3	26.7	33.2	264	60.5	29.4	9.8	0.1	0.2
83	27.7	33.3	241	47	44	7	0	0
80.2	26.6	33.2	293	46.5	41.7	10.6	0.8	0.4
81.3	27.3	33.6	119	50.7	40.3	7.6	0.4	1
82.5	28.7	34.7	191	71.1	15.4	13.3	0.1	0.1
83	27.9	33.6	247	88	9.5	2.3	0.1	0.1
82.7	27.4	33.1	198	70.7	15.5	11.8	1.8	0.2

78.3	25.2	32.2	336	7	63	22	1	0
77.7	26	33.4	399	64	33	3	0	0
71.8	24.8	34.5	365	62.1	25.9	11.7	0.1	0.2
76.8	26.4	34.4	138	19	49	30	0	0
87.6	29.8	34.1	308	86	5	6	0	0
79.5	26	32.7	282	84.5	7.5	7	0	0
77	25.5	33.1	654	72	14	12	2	0
83.4	28.9	34.7	233	66.8	23.6	8.5	0.8	0.3
76.5	26.3	34.4	323	52.7	35.2	11.5	0.3	0.3
81.2	27.2	33.5	391	62.9	24.7	12.1	0.1	0.2
74	25.4	34.3	324	38.3	50.9	10.2	0.3	0.3
80.8	26.4	32.7	250	50	38	9	2	0
81.9	28	34.2	171	23	70	7	0	0
80.3	27.5	34.3	208	58.6	32.8	8.4	0.1	0.1
80.2	28.1	35.1	178	39.2	51.6	8.7	0.2	0.3
70.6	24.3	34.4	262	55.6	35.2	6.8	1.5	0.9
83.7	28.8	34.5	320	44	51	3	0	0
76.7	26.1	34	125	7	82	10	0	1
81.8	28.3	34.6	282	34.4	54.8	9.8	0.8	0.2
82.2	27.3	33.2	165	77.9	11	10.9	0	0.2
82.8	28.3	34.1	141	23	47	26	0	0
82.7	28	33.9	205	53	33	14	0	0
75.8	27.1	35.7	233	46.2	39.7	13.6	0.3	0.2
81.7	27.7	34	189	58.3	32.9	8.3	0.2	0.3
80.3	27.3	34	274	81.7	14.1	3.9	0	0.3
80.2	26.8	33.4	223	77	12	10	0	0
73.4	24.7	33.7	400	62	21	17	0	0
79.2	26.5	33.5	180	56.2	35.9	6	1.7	0.2
74.5	25.9	34.8	301	51.7	38.5	7.7	2	0.1
81.6	27.8	34.1	237	48.5	42.1	8.8	0.3	0.3
80.4	26.8	33.3	176	63	24	12	0	0
80	27	33.7	177	53.2	34	12.2	0.3	0.3
83.1	27.6	33.2	105	58.9	26.9	13.5	0.4	0.3
76	25.3	33.2	274	41	49	9	0	1
89.6	29.8	33.2	383	49.1	32.4	10.1	8.1	0.3
77.5	26.2	33.8	242	35.5	50.4	13.2	0.1	0.8
79.6	27.4	34.4	395	68.7	26	5	0.1	0.2
83.1	26.4	31.7	420	77	14	8	0.5	0
80.1	27.7	34.6	231	33	50	14	3	0
68.5	22.5	32.8	291	82	12	6	0	0
76.5	25.7	33.6	366	73	17	8	2	0
74.5	26.6	35.7	210	45.2	45.3	9	0.1	0.4
77.3	25.7	33.2	289	43.7	42.2	9.9	3.5	0.7
79	25.6	32.4	298	40	53	3	0	0
70.7	24	33.9	372	61.4	32.2	6	0.2	0.2
76.1	26.8	35.2	571	52.9	36.1	11	0	0

81.1	27.2	33.6	206	91	5.8	3.1	0	0.1
89.9	30.2	33.6	189	83.5	10.5	6	0	0
75.2	25.3	33.6	272	46.7	40.2	12.3	0.4	0.4
81.2	27.1	33.4	187	43.1	46	10.8	0	0.1
81.7	27.8	34.1	285	72.4	21.2	6.2	0.1	0.1
76.7	25.3	33	295	64.1	23.5	12.3	0	0.1
96.2	32.9	34.3	381	50.8	38.2	10.1	0.7	0.2
76.9	27	35.1	232	78.1	17.6	4	0.1	0.2
80.5	28	34.8	343	61	30	6	3	0
78	26.1	33.4	388	72.8	18.8	6.7	1.6	0.1
79	26.8	33.9	226	33.7	50.7	14.6	0.4	0.6
81.1	27.3	33.6	389	64.6	25.2	9.1	0.6	0.5
82.5	27.8	33.7	437	62	28	9	1	0
84.9	28.5	33.6	232	69.9	18.3	11.5	0.1	0.2
82.3	27.9	33.9	148	37.6	50.8	10.2	0	1.4
77.4	25.8	33.3	264	74	18	8	0	0
73.2	25.7	35.1	202	52.3	30.5	12.4	4.3	0.5
75.6	26.4	34.9	283	73	22	4	1	0
79.8	27.3	34.2	253	72	14	14	0	0
77.8	26.3	33.9	265	60.5	24	13.5	0	0
74.9	25.6	34.2	261	80.8	14.4	4.7	0	0.1
82.6	27.7	33.5	347	91	6.4	2.5	0	0.1
79.1	25.8	32.6	449	65.5	25.5	7.5	0	0
82.8	28.1	34	290	83.6	8.7	7	0.5	0.2
78.9	27.8	35.2	403	69.5	20	10	0	0
75.4	25.9	34.4	242	35.2	48.7	15.4	0.1	0.6
81	28.3	34.9	314	53.6	36.1	8	2	0.3
84.6	29.6	35	376	52	41.5	5	1.5	0
74.1	25.1	33.9	314	37.3	53.9	5.6	2.9	0.3
78.4	26.4	33.7	370	56	39	4	1	0
81.4	28	34.4	197	64	30	5	1	0
82.5	27.6	33.4	272	69.7	20	8.5	1.5	0.3
73.7	24.5	33.2	573	52.6	35.3	10.6	1.2	0.3
80.8	27.1	33.6	204	62	26.8	9.2	1.6	0.4
83.1	27.9	33.5	367	63	29	8	0	0
76.3	26.3	34.5	155	37.5	47.7	14.4	0.2	0.2
74.3	24.9	33.5	205	36.1	48.6	14.2	0.9	0.2
82.7	27.8	33.6	200	51.9	31.8	15.3	0.5	0.5
74.6	24.9	33.4	475	43.5	40.7	5.4	10.2	0.2
82	26.9	32.7	392	75.5	12.9	6	5.5	0.1
80.1	26.3	32.8	264	38	57	5	0	0
82.5	27.6	33.4	343	65.9	27.9	5.2	0.7	0.3
78.4	26.6	33.9	261	46.5	47.3	5.8	0	0.4
72.2	25	34.6	324	64	26	10	0	0
78.1	25.5	32.7	314	35.3	56.6	5.9	1.6	0.6
85.7	30	35	394	79.7	16.3	3.9	0	0.1

82.4	27.4	33.2	521	80	14	6	0	0
73.5	24.1	32.8	349	36.6	53.5	8.9	0.4	0.6
80.2	27.6	34.3	296	52.5	36.3	9.8	0.7	0.7
79.1	27.2	34.4	196	68.7	25.8	4.9	0.4	0.2
79.5	27.5	34.6	483	51	43.5	4	1.2	0.3
76.9	25.8	33.5	275	78.6	12.3	8.4	0.5	0.2
73.1	24.2	33.1	258	58.2	29.7	11.9	0.1	0.1
79.6	27.4	34.4	313	93.8	4.7	1.3	0.1	0.1
73	24.1	33	270	28	59	12	1	0
74.1	25.3	34.1	200	25	54	12	1	2
70.6	23	32.6	188	29	41	3	0	0
77.4	27.1	35	349	43.3	52.7	3.6	0.2	0.2
76.5	25.8	33.8	317	71	19.9	8.7	0	0.4
78.6	26.1	33.2	383	44.7	47.1	6.1	1.8	0.3
98.1	33.8	34.4	490	28.3	61.3	6.4	3.9	0.1
66	21.3	32.3	287	54	39.5	5.5	0.5	0
81.2	28.2	34.8	244	57.7	32.2	9.2	0.7	0.2
77.1	26.7	34.6	386	81.8	9.7	3.8	4.6	0.1
82.6	28.4	34.3	266	78	13.9	8	0	0.1
80	26.7	33.3	241	56	35	6	1	0
78.8	26.6	33.7	183	14	79	4	2	0
72.2	24.8	34.4	243	65	10	24	0	0
77.2	26.4	34.1	211	71.9	19.3	8.6	0	0.2
78.8	27.1	34.4	246	51	38.5	9.7	0.3	0.5
81.2	28	34.4	343	57	24.7	13.7	3.7	0.9
84.5	28.3	33.5	163	24	55	19	2	0
84	29.4	35	126	73.7	15.6	7.5	2.9	0.3
79.4	26.9	33.8	254	79.1	12.5	8.1	0.1	0.2
78.6	27.1	34.5	100	64.9	30.2	4.7	0	0.2
75.7	24.7	32.7	160	39	43	13	1	1
81.5	27.3	33.5	121	46.4	41	11.6	0.5	0.5
77.9	26.8	34.4	287	82	13	1	0	0
77.7	27.6	35.5	298	84.8	10.2	2.5	2.3	0.2
79.1	27.2	34.3	264	48	43.4	7.6	0.8	0.2
80.2	26.8	33.4	310	71.1	19.6	9.2	0	0.1
78.4	27	34.4	188	53	26	17	0	0
77.3	26.1	33.7	268	73.4	15.9	9.9	0.5	0.3
81.4	27.8	34.1	165	47.2	43.2	8.8	0	0.8
77.7	26.5	34.1	122	37.4	53.1	8.5	0.6	0.4
78.7	27.6	35.1	273	41.1	46.6	11.9	0.1	0.3
80.1	27.9	34.8	369	49.2	34.1	13.6	1.6	1.5
74.7	25.6	34.3	60	39.8	47	11.6	1.1	0.5
80.5	27.1	33.7	211	51.8	37	10.6	0	0.6
76.2	26.6	34.9	159	45.1	43.7	10.2	0	1
79.7	27	33.9	239	59.2	24.2	14.9	1.5	0.2
74.8	25.5	34	364	38	55	6	1	0

85.4	29.5	34.5	379	66.8	23.8	8.8	0.4	0.2
77.6	26.3	33.9	264	64.9	26.9	6.5	1.6	0.1
79.6	26.2	32.9	278	44.4	49	5.7	0.4	0.5
80.6	27	33.5	171	72	10	16	0	0
74.8	25.2	33.6	311	35	54	7	3	1
66.7	21.9	32.8	393	84.5	10	4	0	0
66.5	21.3	32	214	83.7	12.9	3.3	0	0.1
89.2	30.5	34.2	381	71.5	19.6	7.8	0.9	0.2
73	25.4	34.7	361	67	27	5	1	0
79.8	27.6	34.6	383	18.5	65.4	15	0.6	0.5
84.7	29.1	34.4	198	51	29	18	1	1
78.5	26.7	34	175	57	26	16	1	0
74.9	26.6	35.5	157	41	44.6	12.3	0.2	1.9
83.8	27.9	33.3	478	61	27.5	11.5	0	0
80.7	26.8	33.2	327	47.8	41.1	6.2	4.3	0.6
72.1	24.7	34.3	348	46	44.9	8.4	0.3	0.4
77.6	26.5	34.2	233	50.8	36.5	11.7	0.6	0.4
79.4	27.7	34.8	187	59.1	32.5	7.7	0.2	0.5
78.1	26.8	34.3	215	70.7	18.1	8	2.7	0.5
83.4	27.2	32.6	271	37.7	51	10.3	0.7	0.3
89.3	29.5	33	529	55.7	24	17.9	2	0.4
76.8	25.9	33.8	379	60	27	11.5	1	0
73	24.5	33.5	349	52.6	36.5	10	0.4	0.5
75.9	26.1	34.4	260	73	16.7	9.9	0.1	0.3
77.3	26.3	34	177	42	44	11	2	1
82.2	27.6	33.6	217	66.5	23.9	7.5	1.7	0.4
81.2	28.5	35.1	383	81	8	11	0	0
73.7	25	33.9	169	62.5	30.9	5.6	0.7	0.3
76.8	26	33.9	285	29.7	58	11.4	0.6	0.3
81.8	28.1	34.3	387	67.5	21.3	10.9	0.1	0.2
70	23.6	33.7	286	34.7	53	5.5	6.6	0.2
82.1	28	34.1	198	77.5	11.5	10.8	0	0.2
85.5	28.7	33.5	253	60.5	30.7	8	0.6	0.2
80.4	26.1	32.5	199	16	78	3	0	0
85.3	28.7	33.7	183	53	40	7	0	0
80	25.9	32.4	322	73	22	5	0	0
81.3	27.4	33.8	218	65.5	25.1	7.4	1.6	0.4
75.8	25.8	34	261	50.9	39.6	8.8	0.4	0.3
80.8	27	33.4	272	79.5	14	5.2	1.1	0.2
82.3	27.9	34	213	73.5	17.4	9	0	0.1
83.3	29	34.8	334	73.5	18.7	7.5	0	0.3
80.5	28.1	34.9	406	70.6	21.9	6.8	0.1	0.6
81	28	34.5	97	62.3	31.2	6.4	0	0.1
82.6	28.3	34.3	164	42.6	49.5	7.2	0.5	0.2
78.2	26.2	33.5	324	47	20	4.5	0	0
80	25.7	32.2	303	52	40	8	0	0

76.2	25.3	33.2	201	46.5	46.7	5.8	0.7	0.3
79.8	27.5	34.5	181	52	42.1	5.5	0.2	0.2
78.4	26.1	33.3	254	31.7	60.3	7.5	0.3	0.2
74.4	25.2	33.8	256	63	26	11	0	0
81.8	28.6	35	171	70.6	16.6	12.1	0.4	0.3
80.9	26.8	33.2	164	70.3	22	7.1	0.5	0.1
72.4	22.8	31.5	453	37.2	54.5	6.8	1.2	0.3
79.9	26.5	33.2	267	81.8	10.5	7.1	0.1	0.5
76.4	25.7	33.6	236	48.7	44	6.5	0.4	0.4
85.8	29.4	34.3	178	58.7	30.1	11.1	0	0.1
80.7	26.9	33.3	443	71.2	22.3	6	0.3	0.2
74.8	23.9	31.9	218	32	48	17	0	0
78.3	26.5	33.8	322	81	13	1	0	0
81.4	28.6	35.1	295	56.2	36.1	5.5	1.1	1.1
83.6	29.6	35.5	162	49.2	37.3	11.2	1.9	0.4
78.9	27	34.3	241	78	18.2	3.6	0.1	0.1
74.5	23.4	31.4	376	53.7	36.4	8.4	1.3	0.2
71.5	23.4	32.7	262	65	11.5	16.5	1	0
83.1	28.3	34.1	399	53.5	38.4	7.4	0.5	0.2
73.8	24	32.5	398	51.6	40.1	7	0.8	0.5
81.6	26.5	32.5	241	71.4	19.5	8.9	0.1	0.1
76.4	25.8	33.8	198	76	17	7	0	0
78.2	25.3	32.3	289	71.7	22.3	5.6	0.1	0.3
74.9	25.7	34.3	170	68.8	20.3	10.7	0.1	0.1
81.5	27.9	34.2	393	22.2	72.2	3.1	2.1	0.4
80.3	26.3	32.8	214	42.8	49.7	5.2	2	0.3
80.1	26.9	33.5	96	32.1	61.3	4.8	1	0.8
73.4	24.3	33.1	299	65.2	27.5	7	0.1	0.2
76.7	27.4	35.7	323	50.4	38.5	9.6	1.3	0.2
82.1	28.5	34.7	207	52	29	16	1	0
71.5	23	32.2	239	61.5	32	5.3	1.1	0.1
85.7	30	35	378	62	28.9	8.4	0.5	0.2
76.8	25.4	33.1	228	72.8	22	5	0	0.2
75.7	25.4	33.5	349	52.4	39.1	6.8	1.4	0.3
74.9	25.3	33.7	237	70	13	8	3	0
80.2	27.1	33.7	246	68	27	5	0	0
92.9	30.1	32.4	704	35	45	18	2	0
76.6	25.5	33.2	198	38	45	16	1	0
82.5	27.8	33.6	181	54.2	30.9	9.5	4.6	0.8
86.9	29.8	34.3	439	63.2	24.1	11.1	1.4	0.2
75	24.8	33	366	47.3	38.5	13.6	0.1	0.5
81.3	27.9	34.3	311	87.2	8.2	4.2	0.2	0.2
84.2	28.9	34.3	503	49.8	36.2	11.4	2.4	0.2
72.1	23.9	33.1	223	51.2	29	18.4	1.1	0.3
83.6	28.7	34.3	263	31	45	16	2	1
76.4	24.3	31.8	280	34	60	6	0	0

79.7	27.3	34.3	262	58.8	33.2	7.8	0.1	0.1
83	28.2	34	300	81	8	9	0	0
78.2	25.9	33	327	56.3	36.4	6	0.9	0.4
81.2	27.7	34.1	175	52	34	12	0	0
78.7	27.3	34.7	333	85.5	11	3.5	0	0
79.3	27.6	34.8	303	55.4	38.3	6.2	0	0.1
74.1	24.5	33.1	430	55	37	7	0	1
73.4	24.3	33.1	147	59.6	34.1	6.1	0	0.2
79.6	27.1	34	173	76.4	17.8	5.3	0.4	0.1
76.7	25.9	33.7	192	65	25	10	0	0
80.5	27.8	34.5	166	27	66	7	0	0
77.8	27.1	34.8	286	72	15.8	11.4	0.7	0.1
82.8	28.6	34.5	217	79.8	11.5	8.5	0	0.2
71.9	24.5	34.1	300	74.5	19	4.5	0	0
76.1	26.3	34.6	217	69	17	11	2	0
74.7	25.3	33.9	261	23	70.8	2.7	3.4	0.1
77.2	26.6	34.5	301	30.3	56.1	4	9.4	0.2
80.8	27.5	34	177	64.5	20.3	14.8	0.2	0.2
76.8	27	35.2	267	73.5	18	7.7	0.6	0.2
74.5	25.9	34.8	390	62.2	27.2	7.8	2.5	0.3
77.8	26.4	34	132	71.4	22.7	5.8	0	0.1
79.7	27	33.9	198	41	46	13	0	0
77.2	27.6	35.7	221	76.5	12.5	10	0	0
87.1	29.4	33.7	175	31.3	53.1	15.3	0.3	0
78	25.7	33	277	72.2	15.7	11.7	0.3	0.1
82.1	27.7	33.7	321	74	13	7	4	0
79.1	25.8	32.6	263	84.7	5.9	8.9	0.4	0.1
80.6	26.4	32.7	281	77.1	16.8	5.8	0.1	0.2
85.9	29.3	34.2	260	79	12	9	0	0
76.5	27.3	35.6	154	47.9	37.8	13.5	0.6	0.2
74.1	24.6	33.1	290	43.3	43.3	13	0.3	0.1
80.3	26.3	32.7	341	85.5	4.5	8	0	0
74.1	24.2	32.7	260	73.4	19.6	6.7	0.1	0.2
78.2	27	34.5	330	57.4	32.2	8.1	2	0.3
79.9	27.7	34.6	267	78	8	14	0	0
74.2	24.9	33.5	280	42	37	4	0	0
83.8	28.7	34.3	189	79	14	7	0	0
81.2	26.9	33.2	358	64.1	24.3	5	5.9	0.7
79.6	27	33.9	322	43.9	43.8	10.3	1.8	0.2
73.8	24.6	33.3	193	64.2	27.4	7.9	0.3	0.2
77.8	26.2	33.6	213	32.2	60.4	7.2	0.1	0.1
79	27.8	35.2	241	78.7	15.4	4.7	0.9	0.3
77.6	26.7	34.3	241	68.9	18.8	12.1	0.1	0.1
79.8	26.1	32.7	166	40	44	7	0	0
79.5	26.6	33.4	184	61.4	32	6	0	0.6
80.5	27	33.6	242	68	23	8	1	0

77.6	26.4	34.1	142	49.2	38.3	12.3	0.1	0.1
74.4	26.8	36.1	123	62.9	31.8	4.9	0.2	0.2
78.9	26.6	33.7	200	81.9	11.8	6.1	0	0.2
86.5	29.9	34.5	300	66	26	8	0	0
81.1	27.2	33.5	198	65	25.6	8.6	0.4	0.4
83.3	29	34.8	177	53	39	5	0	0
79.4	27	34.1	309	78	13	9	0	0
77	26.4	34.3	175	75.2	19.5	5.3	0	0
80.1	26.8	33.4	266	66	27	6	0	0
73.1	24.8	34	203	80.9	10.8	7.6	0.5	0.2
75	25	33.3	472	61.9	23.4	10.2	4	0.5
80.4	27.6	34.3	390	57	31	11	0	0
74.5	24.6	33.1	229	47.2	39.7	9.5	1.9	1.7
78.3	26	33.2	209	59.8	29.5	10.3	0.1	0.3
76.3	27	35.3	193	54	38	7	1	0
87.6	29.5	33.6	177	71.4	20.6	4.2	3.6	0.2
81.4	28.3	34.7	76	44	47.1	7	1.5	0.4
83	29.1	35.1	292	78	15	5	0	0
83.9	28.3	33.7	174	84	8	7	1	0
77.6	27.3	35.2	322	80.5	9	8.5	0	0
83.3	28.8	34.6	167	67	27	6	0	0
81.8	27.7	33.9	223	32.3	53.9	6.9	5.8	1.1
82.5	27.9	33.8	265	70	21	7.8	0.8	0.4
78.2	26.6	34	308	61.9	27	7.8	3	0.3
83.4	27.7	33.2	238	49	41	7	1	0
74.6	25.4	34	278	82.5	5.5	7.5	0	0
76.8	26.2	34.1	559	57.7	35.5	5.8	0.8	0.2
76.9	27.4	35.7	290	73.8	16.4	9.4	0.1	0.3
80.2	25.5	31.8	294	30.9	66.2	2	0.7	0.2
80.4	26.7	33.2	312	67.3	26	6.4	0	0.3
77.2	27	35	315	77	9	10	0	0
80.1	26.5	33.1	182	69	23	8	0	0
78.4	27.5	35	237	57.4	32.9	5.7	3.6	0.4
82.1	28.7	35	267	78.3	16.5	4.6	0.4	0.2
84.4	28.2	33.4	176	39	52	5	0	0
78.6	26.2	33.3	180	78.9	15.4	5.6	0	0.1
79.5	26.8	33.7	253	85	7	3	0	0
81.3	27.2	33.5	120	69.5	17.9	12.2	0.2	0.2
82.8	27.7	33.4	144	66	20	9	1	0
80.9	26.4	32.6	252	63.5	14	22	0	0.5
91	29.4	32.3	515	66.4	24.2	6.2	3	0.2
81.3	26.4	32.5	324	74.5	19.3	5.7	0.1	0.4
80	27	33.7	194	47	32	20	0	0
78.9	26.2	33.2	146	60.6	33.4	5.1	0.8	0.1
78	25.8	33.1	159	45	27	10	0	0
80.2	27.6	34.5	275	52.2	35.9	7.7	3.6	0.6

80	27.4	34.2	339	40.3	47.5	5.6	6.2	0.4
86.6	28.4	32.8	480	68.5	27.1	4	0.2	0.2
80.4	28	34.9	138	76	12.7	10.9	0.2	0.2
72.7	23.6	32.4	283	79.1	15.2	5.1	0.5	0.1
81.1	28.1	34.6	241	75.4	9.5	13	1.8	0.3
76.9	25.6	33.3	398	64	28	6	2	0
74.1	24.2	32.7	281	57	29	11	3	0
81.4	28.5	35	257	80.8	13.7	4	0.9	0.6
79.5	27.6	34.8	273	75.8	15.1	6.7	2.3	0.1
79.1	26.8	33.9	276	63.3	24.3	11.8	0.2	0.4
70.2	23	32.7	350	59.3	28.6	9.7	2.1	0.3
80.2	25.9	32.4	317	68.1	25.6	5.7	0.5	0.1
83.3	27.1	32.6	359	68.1	23.8	7.8	0.1	0.2
83.3	28.1	33.7	249	51.7	39.2	8.5	0.4	0.2
76.6	25.3	33	254	61	23	15	1	0
83	28.6	34.5	150	46	42.7	9.7	0.3	1.3
83.5	27.7	33.1	268	66	27	7	0	0
78.9	25.7	32.5	156	37	52	6	0	1
81.5	28.2	34.7	214	65.4	25.8	7.9	0.5	0.4
75.4	26.3	34.9	337	54.8	29.3	13.6	2.2	0.1
72.9	25.1	34.4	399	63.6	29.6	6.4	0.2	0.2
80.5	27.7	34.4	137	63.4	27.8	8.6	0	0.2
80.6	28.7	35.6	143	62.2	29.5	6.8	1	0.5
78.9	26.5	33.6	353	67.4	24.7	7.2	0.4	0.3
78.2	26.7	34.1	256	59.8	33.9	6	0	0.3
78.4	26.7	34.1	171	54.3	29.1	16	0.3	0.3
81.2	27.6	33.9	258	81.6	15	3.1	0.1	0.2
78.3	26.5	33.9	196	73.9	17.3	8.2	0.4	0.2
81.6	26.9	33	209	13	79	5	1	0
80.8	26.8	33.2	187	36.5	52.7	7	3.6	0.2
78.5	26	33.2	266	87	7.5	5.5	0	0
77.2	26.2	33.9	489	48	40	10	0	2
82.3	28.6	34.8	206	54.2	39.9	5.5	0.2	0.2
83.3	28.3	33.9	139	62.1	30.5	7.1	0	0.3
91.7	31.6	34.4	758	45	47	5	3	0
80.2	28	34.9	132	50.8	37.8	9.2	1.6	0.6
80.8	27.3	33.8	131	48.5	43	5.9	2.2	0.4
82.7	27.9	33.7	337	36.7	55.6	6.6	0.6	0.5
78.9	25.6	32.4	175	69.5	22.2	5.6	2.4	0.3
77	25.5	33.1	350	62.2	27.4	9.1	1.1	0.2
73.4	24	32.7	299	42	38	15	2	0
80.8	26.6	32.9	235	59.2	33.4	6.3	0.8	0.3
76.5	25.7	33.6	193	79.7	12.5	7.6	0	0.2
81.4	27.6	34	223	80.4	13.5	5.8	0.1	0.2
73.8	24.5	33.2	291	49	23	15	0	0
81.2	27.6	34	187	22	68	7	1	0

75.3	25.5	33.9	428	68.5	20	11	0	0
82.8	28.4	34.3	172	63.3	28.4	7.7	0.4	0.2
77.3	26.4	34.1	317	64	22	12	1	0
78.8	26.5	33.7	242	45.3	43.2	10.9	0.1	0.5
75.1	25.6	34.1	205	75	15	9	0	0
83.8	27.8	33.2	175	43	45.4	10.5	0.9	0.2
77.7	27.3	35.2	230	47.1	37.7	13	1.1	1.1
77	26.1	33.9	228	56.4	37.6	5.1	0.7	0.2
80.5	27	33.6	167	48	18	28	1	0
80.2	28	34.9	230	66	29	4	1	0
85.8	28.2	32.9	468	55.2	28.2	13	3.4	0.2
83.4	24	28.7	273	72.4	18.8	5.5	3.1	0.2
79.4	27.2	34.3	194	63	25	12	0	0
81.4	27.3	33.5	290	13.1	71.9	13.6	1.2	0.2
83.5	28.1	33.7	274	51	38	6	1	0
72.1	25	34.6	375	70.5	20.8	6.9	1.3	0.5
79.7	26.6	33.4	355	73.8	18.6	5.6	1.9	0.1
82.2	25.8	31.3	494	43	51.5	5.3	0	0.2
70	23.3	33.2	179	73.6	20.6	5.5	0.1	0.2
77.6	24.8	32	131	5	84	6	1	2
77.9	25.9	33.2	207	33.6	49.5	14.3	1.6	1
83.8	27.7	33	261	60	28	9	2	0
74.2	24.3	32.8	397	61.2	32.8	4.4	1.3	0.3
83.1	27	32.5	275	48	42	9	1	0
77.8	26	33.4	189	74	20	6	0	0
76.3	26.5	34.8	183	69.7	21.6	8.1	0.2	0.4
72.3	24.2	33.4	343	63.9	28	6.5	1.4	0.2
75.3	25.1	33.3	150	43	40.7	13.4	2.1	0.8
82.8	27.5	33.2	223	33	53	11	1	2
75.2	25	33.2	422	66	28	5	0	0
81.6	25.7	31.5	189	23	72	4	0	0
78.6	27.7	35.2	146	71.5	21.1	6.7	0.3	0.4
80.5	26.6	33	191	81	13	6	0	0
79.6	28.2	35.4	379	69	26.2	4.6	0.1	0.1
81.5	27.1	33.2	181	58.9	27	13.3	0.4	0.4
74.4	24.3	32.6	655	44.4	45	9.4	1	0.2
77.2	26.1	33.8	545	65	27	5	0	0
69.6	21.3	30.5	616	47	41	12	0	0
80.2	27.1	33.8	274	81	17	2	0	0
83.2	28.3	34	132	63.3	27.7	8.7	0	0.3
71.5	24.1	33.7	78	29.3	55.7	12.1	1.1	1.8
73.1	25.6	35	221	77.7	13.6	8.1	0.4	0.2
88.1	27.7	31.5	273	70	21	9	0	0
81.8	27.6	33.7	149	36.4	51.5	11	0	1.1
81.2	27.9	34.3	249	81.3	9.7	8	0.8	0.2
82	27.6	33.6	330	64	21	7	1	0

71.8	23.5	32.7	330	74	17	8	1	0
82.3	29.1	35.4	274	91.2	6.3	2.2	0.1	0.2
83	28.4	34.2	193	22	60	14	0	0
74.8	26	34.8	246	76.7	16.6	6.2	0.3	0.2
86.4	28.7	33.2	326	77.2	18.5	3.9	0.2	0.2
79.1	28	35.4	187	30	45	10	0	2
77.4	26.9	34.7	196	53.4	35.5	9.5	1.3	0.3
82.1	27.4	33.4	195	62.7	26.9	10.1	0	0.3
79.7	27.7	34.7	192	62.1	27.4	10.1	0.2	0.2
82.3	27	32.8	373	47.4	45.3	6.9	0.3	0.1
76.6	26.7	34.8	232	36	44	11	1	0
78.6	27.3	34.8	276	48.8	41.8	8.5	0.8	0.1
77.7	26.1	33.6	141	44	49	5	0	1
76	25.6	33.7	398	65.2	29.3	5.2	0.2	0.1
86.1	28.2	32.8	149	31.3	62.5	5.2	0.6	0.4
69.2	22.6	32.7	210	61.8	28.1	9.9	0.1	0.1
78.3	26.5	33.9	274	60.3	28.3	9.9	1	0.5
82.5	26.7	32.4	213	33.6	56	9.5	0.3	0.6
75.2	24.2	32.2	222	60.1	34.6	5.1	0	0.2
79.9	27.2	34	310	77.8	13	8.7	0.1	0.4
72.6	25.8	35.5	268	38.5	54.3	4.5	2.2	0.5
76.1	24.8	32.6	247	74.6	14.2	11.1	0	0.1
85.6	28.9	33.8	201	86.7	7.1	6.1	0	0.1
78.5	26.8	34.1	213	39	34	12	0	0
83.9	27.5	32.7	409	31.3	56.7	8.5	2.9	0.6
78.9	26	32.9	340	45.4	42.7	10.6	0.9	0.4
82.2	29.4	35.8	205	52.7	36.7	9.6	0.5	0.5
80.1	26.5	33.1	145	65.7	24.3	9.2	0.6	0.2
78.4	27.5	35	248	38.9	50.1	9.1	1.4	0.5
78.8	27.3	34.6	210	56	28	15	0	0
79.7	27.1	34	185	43.6	46.8	9.3	0	0.3
81.9	27.5	33.6	171	68	23	8	1	0
73.4	24.3	33.1	541	66	26.5	7	0.5	0
81.9	29.9	36.6	368	78	16	4	0	0
79.5	26.5	33.3	203	76.5	12.7	10.7	0	0.1
80	27.6	34.5	149	60.7	26	13.1	0	0.2
79.4	26.6	33.5	198	75	12.8	11.9	0.1	0.2
77.4	26.1	33.7	299	52.5	32.1	14.6	0.5	0.3
79.4	27.3	34.4	247	31	50	18	0	1
77.8	26.1	33.5	412	61	30	8	1	0
72.9	25.3	34.7	262	57	30	11	2	0
82.2	27.4	33.3	220	58	25	6	0	0
78.9	26.8	34	409	50	42	6	2	0
77.9	26.1	33.5	223	43	45.5	9.9	0.6	1
84.7	28.6	33.8	192	76.5	16.8	6.5	0	0.2
76.5	25.2	33	292	66	21	10	2	1

77.6	25.9	33.3	216	77.5	13.6	8	0.6	0.3
81.6	28.5	35	111	49	33	17	1	0
80	26.4	33	413	39	42	12	3	1
80	27.8	34.8	291	72	15	10	1	0
79.3	28.2	35.6	325	87.2	9.6	3.1	0	0.1
76	26.4	34.8	232	72.2	17.1	9.6	0.7	0.4
80.5	27.9	34.6	276	68.5	22.8	8.3	0.2	0.2
76.1	24.9	32.8	99	14	70	16	0	0
79.2	27.2	34.3	182	57.1	36.3	5.9	0.5	0.2
85.6	28.7	33.5	250	83	8	9	0	0
76.9	24.9	32.4	100	56	29	11	3	0
87.1	29.6	34	201	46	39	10	0	0
82.9	27.5	33.2	168	88	6	6	0	0
73.4	24.5	33.3	158	73	12	6	0	0
90.8	30.1	33.2	229	29	56.7	13.8	0.1	0.4
82.5	27.8	33.6	189	62.4	29.8	7.1	0.6	0.1
77.9	26.7	34.3	190	62	24	12	1	0
78.5	27.2	34.7	266	64	25	11	0	0
80.1	27	33.7	201	77.7	14.2	7.9	0	0.2
75.2	25.1	33.3	349	52	44	4	0	0
82.6	27.9	33.8	179	70.8	20.5	8.2	0.1	0.4
79.4	26.6	33.5	179	71	20	6	1	0
84.3	29.4	34.8	283	79.9	14.4	5.4	0.1	0.2
85.1	29.6	34.7	177	59.8	24.7	12.4	2.5	0.6
80.1	26.5	33.1	461	75.5	17	6.5	0	0.5
81	27.5	33.9	268	56.2	33.1	10.2	0.2	0.3
80.3	26.7	33.2	273	31	51	12	3	0
78.5	26.5	33.8	273	36	54	8.5	0	0
80.7	27.5	34.1	265	74.5	18.7	6.5	0	0.3
82.7	29.5	35.7	168	73.5	22.5	3.6	0.2	0.2
79.6	27.3	34.3	331	54.4	32.1	9.3	3.8	0.4
80.2	27.3	34	380	76.5	16	6.6	0.1	0.8
72.7	25.2	34.7	233	70	24	5	1	0
84.6	27	31.9	330	43.1	46.2	6.6	3.9	0.2
80	27.4	34.2	373	52	34	13	1	0
83.8	29.1	34.8	295	79	18	3	0	0
86.6	29.1	33.6	300	81.1	12.8	5.8	0.2	0.1
82.5	28	34	136	37.3	50.2	11.1	1.2	0.2
78.7	27.3	34.6	211	83	6.3	10.4	0.1	0.2
77.6	26.9	34.7	326	62.2	27.6	7.3	2.4	0.5
80.9	27.3	33.8	305	40.3	47.3	7.3	4.6	0.5
73.6	24.1	32.8	281	59	31	8	2	0
80.1	27.2	33.9	296	67.5	25	6	1.4	0.1
78.7	27.2	34.6	402	71.8	21.5	6.3	0.3	0.1
80.9	28	34.6	214	59	30	11	0	0
82.3	27.4	33.3	321	86.7	10.2	2.6	0.4	0.1

76.2	25.5	33.4	166	74	15	5	4	0
80.6	27.3	33.9	207	76.7	16.8	5.8	0.5	0.2
80.3	26.2	32.7	188	80.1	10.3	8.1	1.2	0.3
80.9	27.4	33.9	272	67.7	20.9	10.9	0.1	0.4
81.3	28.5	35	164	52.8	39.8	6.5	0.8	0.1
82.3	28.4	34.5	170	65.8	18.4	14.6	0.9	0.3
78.4	27.1	34.6	397	79.7	10.7	7.3	0	0
78.6	27.9	35.5	341	90	7	3	0	0
77.1	25.6	33.2	340	49	42	6	0	1
80.6	26.9	33.3	201	61	31	8	0	0
89.1	29	32.6	214	62	31.3	6.4	0	0.3
84.7	28.4	33.5	182	81	6	13	0	0
82.9	28	33.8	301	86.2	9.3	3.8	0.5	0.2
77	26.1	33.8	349	42	53	5	0	0
78.5	27.5	35	392	45.1	45.4	7.7	1.6	0.2
78.2	25	31.9	248	68.5	24.6	5.8	1	0.1
79.5	26.2	32.9	241	51	36	12	0	1
74.3	25.1	33.8	255	43	46	10	0	1
81.1	28.4	35.1	286	55	37	8	0	0
85.3	28	32.8	576	68.3	21	9.6	1	0.1
83.3	28.8	34.6	69	28	53	16	0	1
81.1	27.4	33.8	303	50.1	35.1	7.9	5.8	1.1
79.3	27.4	34.6	226	31.4	51.5	8.1	7.9	1.1
85.1	29.6	34.8	164	69	21	9	0	0
77.8	27.2	35	346	54.5	35	8.2	1.8	0.5
82.9	27.4	33.1	292	37	54.3	5.7	2.3	0.7
83.2	28.1	33.8	403	71.3	21.1	7.3	0	0.3
79	27.4	34.7	224	50	35	13	1	1
77.1	26.4	34.2	130	42	43	12	0	0
77.6	26.5	34.1	197	64.9	27.6	6.8	0.5	0.2
79.6	27.5	34.5	374	72.5	18.5	7.5	0.5	0
80.3	27.2	33.9	181	58	28	11	0	0
83.1	28.2	34	349	47	48	4	1	0
90.5	30.2	33.3	595	24.9	63.4	7.7	3.1	0.9
80	27.4	34.3	277	92	6	2	0	0
76.7	25.4	33.1	211	51.8	38.9	8.6	0.4	0.3
80.7	27.9	34.5	190	54	36.8	8.7	0.3	0.2
73.8	25.7	34.8	408	60	33	4	3	0
84.9	28	32.9	258	68	19.2	6.8	5.8	0.2
77.6	25.8	33.2	194	64.8	26.1	8.5	0.5	0.1
78.1	25.9	33.1	306	61.3	29.8	7.1	1.5	0.3
77.7	25.2	32.5	395	30	60	5	5	0
77.6	26.1	33.6	259	81.5	10.8	5.7	1.9	0.1
86.4	30.1	34.8	303	52	32	15	1	0
76.6	25.1	32.8	352	49	32.5	13.2	4.9	0.4
76.5	24.9	32.6	305	65.5	28.5	6	0	0

79.6	26.3	33	136	56.6	34.5	7.9	0.8	0.2
80.7	26.5	32.8	618	72	20	8	0	0
75.6	24.6	32.6	482	64.9	27.4	5.2	1.5	1
77.1	25.2	32.6	132	21	70	6	3	0
75.3	26.2	34.8	511	81	13.5	5.5	0	0
83.3	28.9	34.7	177	73.5	15.4	10.8	0	0.3
73.1	24.6	33.7	313	53	41	5	1	0
75.3	25.3	33.6	299	60	27	9	3	0
79.4	27.2	34.3	257	55	30	9	1	1
80.7	27.6	34.1	232	44.1	45.5	9.1	0.7	0.6
81.9	27.8	33.9	158	52.9	35.9	10.8	0.3	0.1
79.4	28.1	35.4	244	66.4	27.6	5.3	0.5	0.2
87.1	29.4	33.8	211	61	23	12	0	0
81.3	26.3	32.4	170	59.5	29.7	10.2	0.3	0.3
82.7	27.8	33.6	202	49.1	40.6	9.9	0.2	0.2
84.2	27.9	33.1	189	69.5	25	4.9	0.5	0.1
81.6	27.8	34.1	183	66	27	7	0	0
77.9	25.9	33.2	172	77.6	17.4	4.5	0.4	0.1
79.3	26.5	33.5	395	46.6	43.9	8.8	0.4	0.3
78.3	28	35.8	194	45	29	14	1	1
76.6	25.9	33.8	458	83	10.5	5	0	0
79.8	27.2	34	264	63.4	26.2	10	0.2	0.2
77.8	26.2	33.7	342	67.4	24.6	6.8	1.1	0.1
81.9	27.8	33.9	183	49	46	5	0	0
79.6	26.6	33.4	143	70.7	20	8.9	0.2	0.2
74.8	24.3	32.5	169	47.6	43.3	8.3	0.5	0.3
80.7	28	34.7	209	52.4	39.7	7.4	0.3	0.2
82.2	27.6	33.6	208	63.1	25.6	10.3	0.6	0.4
79.9	27.3	34.2	209	47.4	40.8	11	0.4	0.4
82.3	27.9	34	167	49	45	6	0	0
80.8	27.3	33.8	229	64.4	27.3	8.1	0	0.2
82.4	28.8	35	219	72.5	22	4.9	0.4	0.2
80	26.9	33.7	181	54.6	37.3	6.5	1.3	0.3
78.3	27.3	34.9	306	78.5	13	6	0.5	0
84.7	28.4	33.5	208	78.1	9.5	12.1	0.1	0.2
78.7	27.4	34.8	191	61.7	30.9	6.7	0.6	0.1
76.9	26.7	34.7	200	58	29	13	0	0
77.8	26.3	33.8	300	47	43	8	0	1
73.8	25.8	34.9	191	75	16	8.5	0.5	0
80.5	27.2	33.8	166	66.5	24	9	0.3	0.2
76.1	25.9	34	255	91	6	2	0	0
80.1	27.7	34.6	246	40.6	47.1	10.6	1.2	0.5
78.8	26.7	33.9	244	53	32	11	4	0
67.3	22.1	32.8	467	75	9.5	14.5	0.5	0.5
75.8	26.3	34.7	210	75	20	5	0	0
76.8	26.4	34.3	49	26	61	10	0	0

68.6	23.3	34	226	77.5	12.5	8.5	0	0.5
83.6	27.6	33.1	274	83.5	8	8	0	0
86.1	28.5	33.1	184	68	19	13	0	0
80.4	26.7	33.2	269	54	38	8	0	0
84.3	28.6	33.9	211	44	42	13	1	0
87	28.8	33.1	247	67.1	22.7	10.1	0	0.1
77.6	26.5	34.2	248	69.7	20.1	7.9	2.1	0.2
83.6	28	33.5	121	41	50	8	0	0
84	27.8	33.1	212	59	21	7	0	0
82.8	28.6	34.6	138	59.9	29.2	10.8	0	0.1
84.7	28.3	33.4	221	44.4	45.6	7.4	2.2	0.4
74.8	25.8	34.4	371	70.1	26.4	3.1	0.2	0.2
81.9	28.4	34.7	201	37	60	3	0	0
80.6	28.3	35.1	169	29.2	62.5	6.5	1.4	0.4
78.9	27.1	34.3	197	47.1	43	8.9	0.4	0.6
82.2	27.7	33.7	540	60	25	6	9	0
81	27.5	34	204	73.6	18.6	7.4	0.2	0.2
70.3	23.6	33.6	286	60.5	33.7	4.9	0.8	0.1
79.9	27.3	34.2	263	79.3	17.5	2.8	0.3	0.1
72.4	24.7	34.1	214	65	19	16	0	0
78.6	27.2	34.6	308	68	27	5	0	0
82.5	27.8	33.7	150	21.4	63.7	14.6	0	0.3
77.2	26.9	34.8	289	61.2	32.4	5.5	0.7	0.2
79.8	27	33.8	217	79.8	11	8.9	0.1	0.2
75.6	25.5	33.7	251	65	31	2	0	0
80.3	26.8	33.3	253	79.8	13.7	6.3	0.1	0.1
70.7	23.1	32.7	393	42.8	47.9	7.6	1.4	0.3
80.4	27.1	33.7	251	39	50.1	9.7	0.4	0.8
95.1	32.7	34.4	200	57.2	23.2	17	2.3	0.3
85	28.7	33.7	284	59.9	30.8	9	0.1	0.2
76.8	26.7	34.8	360	59	29	12	0	0
81.9	27.9	34.1	281	78	13	8	0	1
84.4	28.1	33.3	249	59.4	30.3	9.9	0.2	0.2
73.9	24.1	32.6	255	75.2	16.9	7.5	0.1	0.3
75.5	25.2	33.4	128	55.6	32	11.8	0	0.6
79.6	26.5	33.2	188	55.9	35.1	8.5	0.2	0.3
83.7	28.1	33.6	281	67	11	13	0	0
75.9	26.2	34.6	294	52	39	7	0	0
76.3	26.6	34.9	185	21	62	15	0	1
78.1	27.4	35.1	101	69.6	18.5	11.7	0.1	0.1
81.4	27.4	33.7	290	80.7	13.9	3.9	1.3	0.2
81.9	28	34.2	316	90.5	7.7	1.6	0.1	0.1
76.8	24.8	32.3	210	64.8	28.6	6.2	0.2	0.2
79.3	27.5	34.7	164	62	24	11	0	0
77.1	27.4	35.5	311	52.8	35.5	11	0.4	0.3
82.1	26.9	32.7	181	44	45.8	9.5	0.5	0.2

78.8	26.4	33.5	162	44.6	46.7	8.2	0	0.5
78.4	26.2	33.4	139	66.4	22.1	11	0	0.5
76.4	24.9	32.7	219	53.7	34.2	11.1	0.7	0.3
79.9	27.3	34.2	261	73	18	9	0	0
82.8	27	32.6	238	53.8	35.5	6.1	4.5	0.1
84	29.1	34.7	179	66.2	26.8	6.8	0.1	0.1
73.7	25.1	34.1	217	52.1	31.9	14.9	0.8	0.3
80.6	27.6	34.3	246	69.1	24.2	5.3	1.2	0.2
80.2	27.9	34.8	272	84.1	11.7	2.7	1.4	0.1
82.3	28.6	34.8	232	77	15	6	0	0
78.1	27.3	34.9	198	44	38	15	0	1
78.4	27.6	35.2	180	52.8	36.3	10.5	0	0.4
79.5	27.3	34.3	141	79.6	14.1	6.1	0	0.2
86.1	28.5	33.1	140	62.8	28.6	8.4	0	0.2
81.1	27.6	34.1	224	60.8	33.7	4.8	0.1	0.6
77	25.5	33.1	213	81.4	13.6	4.9	0	0.1
77.6	26.7	34.4	132	49.1	41.5	9.1	0.3	0
78.6	26.8	34.1	355	80	8	6.4	0.3	0
72.3	23.6	32.6	235	65.3	23.7	10.7	0.2	0.1
78.8	27.4	34.7	269	67.8	21.3	9.9	0.8	0.2
85.4	28.2	33	206	70.9	24.4	4.4	0.1	0.2
83.3	28.9	34.7	249	48	44	7	0	1
77.2	26.6	34.4	211	83	8.8	7.9	0.1	0.2
78.1	26.7	34.2	208	75	16	8	1	0
84.7	28.1	33.2	167	51.8	42.3	5.1	0.6	0.2
82.8	28.6	34.5	55	70	18	1	0	1
78.9	26.8	34	121	37	44	2	0	0
85	29.5	34.8	252	84	12	4	0	0
82.2	27.9	33.9	273	84	14	2	0	0
79.2	27.4	34.6	304	53	37.7	8.7	0.3	0.3
75.6	26.1	34.6	362	82.9	10.9	6	0	0.2
84.1	28.2	33.5	217	24	50	16	7	0
76.2	25.8	33.9	218	52.9	37.1	8.6	0.7	0.7
75.8	25.5	33.7	214	30	48	17	1	0
74.4	25	33.6	279	47.4	47	4.8	0.2	0.6
84.8	27.9	32.9	310	82.9	10	6.9	0.1	0.1
81.3	27.1	33.3	300	77.9	17.3	3.6	1.1	0.1
82.8	27.6	33.3	158	70	20.1	7.5	2.2	0.2
78.1	26.9	34.4	291	56.4	34.4	7.5	1.5	0.2
81.7	28	34.2	172	71.3	17.6	10.7	0.3	0.1
77.1	25.8	33.5	192	60.7	32.8	6	0.2	0.3
76.1	26	34.2	208	38.2	47.4	13.3	0.8	0.3
83.5	27.8	33.3	453	68	26	3	0	1
75.1	24.7	32.9	130	36.1	56.9	4.9	1.6	0.5
78	26.9	34.5	311	57.5	32.5	6	3.5	0.5
77.2	25.2	32.7	82	61.3	28.8	9.9	0	0

77	26.1	33.9	150	32	56	9	1	0
68.1	22.7	33.3	192	86.5	6.4	4.1	2.8	0.2
79.5	26.7	33.5	244	41	44	15	0	0
79.9	27.5	34.4	344	46	43	7	4	0
75.1	25.6	34	132	51.1	36.9	11.3	0.3	0.4
78.7	26.7	34	149	67.6	22.3	8.3	1.6	0.2
81	25.5	31.4	391	71.2	15	11.2	2.3	0.3
71.7	24	33.4	259	46.8	39.4	13.1	0.1	0.6
79.7	27.3	34.2	274	58.3	27.6	13.6	0	0.5
80.1	26.8	33.4	400	72	22	6	0	0
83.9	28.6	34	194	39.7	53	7	0	0.3
84.8	27.7	32.6	100	32	47	21	0	0
77.4	26.3	33.9	138	23.7	66.6	9.2	0	0.5
82.8	28.4	34.3	370	92	3	4	0	0
77.7	27.3	35.1	225	72.2	23.1	4.3	0.2	0.2
77.6	25.9	33.4	193	25	66	8	0	1
77.3	26.8	34.7	234	49	30	14	0	0
78.6	26.5	33.7	223	66	29	2	1	1
79.5	27.7	34.8	236	55.1	35.5	8.5	0.4	0.5
76.1	26.3	34.5	158	46.1	41	11.6	1.2	0.1
77.9	25.1	32.2	241	6	71	4	0	0
82.1	27.1	33.1	272	54	35	6	1	1
82.7	29.1	35.2	116	28.3	59.6	11	0.4	0.7
78.5	26.7	34	169	78.4	12	9.5	0	0.1
79.4	26.4	33.2	167	72	20	8	0	0
76.3	26.2	34.3	270	3	83	14	0	0
77.4	27	34.9	261	64.7	20.9	9.7	4.3	0.4
84	29	34.5	365	71.5	21.5	5.5	1.5	0
79.5	27.1	34.1	224	59	27	12	1	0
89.2	29.5	33.1	244	30.1	61.9	7.4	0.3	0.3
81	27.7	34.2	460	48.8	37.5	8.5	4.8	0.4
78.5	26.3	33.5	252	68.9	21.6	9.1	0.2	0.2
79.5	26.5	33.3	358	67.4	24.1	7.3	1	0.2
81.6	25.9	31.8	422	51.6	41.3	5.6	1	0.5
74.9	24.8	33.1	410	57.4	34.5	7.7	0.3	0.1
78.4	27.9	35.6	209	59	26	14	1	0
79.3	27.5	34.7	435	59.5	35	5.5	0	0
85.7	29.2	34.1	120	46	35	16	1	1
75.9	26.2	34.5	162	32.7	57	9.9	0	0.4
80.4	26.9	33.4	165	50.3	40	9.3	0.4	0
78.5	26.5	33.7	181	65.7	27.5	2.8	3.6	0.4
78.1	26.6	34.1	245	45	46	6	0	0
78.5	26.7	34	215	85.1	10.4	4.1	0.3	0.1
76.6	24.2	31.6	11	0	100	0	0	0
84.7	28.3	33.4	206	85.3	7.8	6.4	0.2	0.3
82.6	27	32.7	196	36.4	54.7	8.3	0	0.6

77.6	26.5	34.1	267	45	45.7	8.5	0.3	0.5
81.6	27.6	33.8	239	25	63	8	2	0
88.1	29.4	33.3	295	37.7	49.8	9.2	2.6	0.7
75.5	26.2	34.8	173	39.1	47.1	4.6	8.9	0.3
77	25.6	33.2	215	69.9	20.8	8.7	0.4	0.2
79.1	27.8	35.1	214	62.5	26.9	10.1	0	0.5
78.8	27	34.3	341	48.7	43.1	7.4	0.4	0.4
78.8	26.7	33.9	220	79	12.6	8.1	0.1	0.2
74.1	24.6	33.2	117	26	53	9	0	0
81.5	28.6	35.2	245	62.3	31.4	5.5	0.6	0.2
83.4	27.7	33.2	276	83.8	11.8	4.2	0	0.2
87.1	29.4	33.7	577	43	37	14	4	0
76.2	27.2	35.7	317	54	41	2	1	0
73	25.1	34.3	313	39.1	49.1	9.1	1.9	0.8
79.5	27	34	156	59	24	9	0	0
82.5	29.7	36	277	48.9	39.6	8.7	2.5	0.3
72.5	24.6	33.9	357	56.3	29.7	12.7	1	0.3
69.3	23	33.2	334	58	25	4	0	0
77.5	25.8	33.3	147	49.5	40	8.2	1.8	0.5
80.4	26.8	33.3	221	58	34.5	7	0	0
75.5	25.5	33.7	166	51	38	9	0	0
80.8	26.7	33	243	26.4	59.8	12.9	0.6	0.3
83.3	28.4	34.2	315	44.6	47.5	6.1	1.6	0.2
73.1	26.1	35.8	206	43	42	10	3	1
78.2	26.6	34	178	80	13	6	0	0
81.7	27.5	33.6	157	35.6	52.9	10.2	0.3	1
78.4	26.7	34	291	66	21.1	9	3.4	0.5
79.3	25.9	32.6	290	35	46	17	0	0
79.1	27.1	34.2	318	54.9	35	9.7	0.2	0.2
81.8	27.3	33.3	192	79	10	11	0	0
79.4	26.8	33.8	174	50	26	22	1	0
77.6	25.6	33	277	55	35	7	0	0
83.7	28.1	33.5	180	65.9	25	8.7	0.2	0.2
75.8	25.8	34.1	368	65	25	5	0	0
82.2	28	34	214	79	12	9	0	0
80.3	26.8	33.3	205	65	13	11	0	0
74.7	24.6	33	250	54	37	6	2	0
91.7	30.7	33.4	323	60.9	29.7	7.8	1.2	0.4
78.6	25.6	32.5	340	60.7	30.9	7.2	1	0.2
77.3	26.4	34.2	169	66.3	23.3	9.4	0.5	0.5
82.5	26.6	32.3	100	78	14	8	0	0
75.4	25.2	33.4	336	63.3	22.9	11.2	2.1	0.5
82.7	28.4	34.4	265	50	40	9	1	0
79.2	26.3	33.1	247	43.2	44.5	10.9	1.1	0.3
76.6	26.3	34.3	287	25.1	66.5	6	2.1	0.3
81.9	27.6	33.7	282	74.7	20.5	4.5	0.1	0.2

78.5	26.3	33.5	289	68.9	27	3.8	0.2	0.1
84.5	28.6	33.8	208	75.9	17.7	6	0.2	0.2
65.8	20.2	30.7	237	56.7	29.7	13.2	0.2	0.2
82.1	28.4	34.6	241	68.5	22.9	7.2	1.1	0.3
76.1	26.2	34.4	261	70.2	20.5	8.9	0.3	0.1
78.2	26	33.2	182	69.6	19.8	10.3	0.2	0.1
82.1	28.5	34.7	170	34.7	53.6	10.5	0.3	0.9
79.1	26.9	34	224	80	12.1	7.5	0.3	0.1
83.5	28.1	33.7	432	85.5	6	4	2	0
76.5	25.7	33.6	160	61.5	26.3	12	0.1	0.1
78.7	27.7	35.2	238	57	38	5	0	0
75.3	24.9	33	290	82.5	11.7	5.5	0.1	0.2
81.7	27.3	33.4	249	65.4	23.8	9.1	1.2	0.5
79.8	27.3	34.2	188	84.8	7.7	7	0.4	0.1
77.3	26.9	34.8	191	12	81	4	1	0
76.8	26.5	34.5	182	65.9	25.4	7.8	0.4	0.5
79.6	26.5	33.3	221	72.3	21.9	4.8	0.8	0.2
75.7	26.3	34.7	255	43	42	14	1	0
72.6	24.9	34.3	312	63	27	10	0	0
76.2	26.6	34.9	364	66.8	22.1	6.9	3.8	0.4
80.7	26.7	33.1	226	64	21.7	13.4	0.5	0.4
79.3	27.3	34.4	226	60.2	30.2	9.3	0	0.3
83.8	27.8	33.2	221	88	9	2	1	0
79.6	27	33.9	186	70.1	24.7	4.6	0.4	0.2
83.7	28.9	34.6	156	76.2	20.3	2.7	0.8	0
76.9	26.3	34.2	205	66	24	8	0	0
81.2	27.4	33.7	367	54.8	34.8	9.7	0.3	0.4
76.6	25.8	33.7	341	93.5	3.5	2	0	0
79.5	27.1	34.1	224	83.6	10.8	5.5	0	0.1
77.5	26.2	33.8	248	58	27	15	0	0
79.7	27	34	255	67	19	12	0	0
87.3	29.2	33.4	193	36.6	51.7	11.2	0.2	0.3
85.9	28.4	33.1	177	52.3	34.7	12	0.6	0.4
81.7	27.5	33.6	182	47.1	38.5	13.8	0.2	0.4
78.4	26.9	34.3	267	69.5	21.3	8.3	0.4	0.5
80	26.7	33.4	289	58	32	8	0	0
78.5	27.5	35	267	58.6	32.1	8.7	0.4	0.2
75.5	25	33.1	305	77.3	13.8	8.3	0.4	0.2
80.3	27	33.6	166	46.6	44.2	8.2	0.6	0.4
79.2	27.2	34.3	166	71.8	22.9	4.9	0.2	0.2
81.8	27.9	34	250	39	46	14	1	0
80.9	26.7	33.1	240	78.5	15.7	5.6	0	0.2
75.1	25.8	34.4	237	75	13.3	11.2	0.2	0.3
83.6	26.5	31.7	164	60	32	8	0	0
77.6	26.5	34.2	231	41	42	17	0	0
78.9	26.2	33.2	222	39	51	9	1	0

80.9	27.3	33.7	205	50	31	13	1	0
77.8	25.4	32.7	364	61.7	32.8	4.8	0.6	0.1
80.2	26.9	33.6	218	44	45	8	2	0
78.9	27.3	34.6	276	38.6	52.3	7.6	1.3	0.2
76.5	26.7	34.9	216	66.2	25	8.6	0.2	0
80.1	27	33.8	252	8	73	17	1	1
72.7	23.1	31.8	203	38.3	53.8	7.3	0.3	0.3
82.2	27.8	33.9	261	67	20	12	0.5	0
81.4	28.1	34.4	254	38.4	50.2	9	1.7	0.7
80.8	26.7	33.1	116	21	63	10	4	0
83	28.3	34.1	214	73.4	21.4	5	0	0.2
76.4	26.1	34.2	170	58.6	28	12.4	0.9	0.1
80.8	26.9	33.2	288	51	41	6	0	0
83.4	27.8	33.3	265	54	35	11	0	0
77.9	26.7	34.3	399	43.7	42.7	9.3	3.4	0.9
81.1	27.6	34	164	49.8	39.1	10.4	0.7	0
76.9	25.6	33.2	221	34	58	6	2	0
78.5	26.6	33.9	145	18	70	11	1	0
75.6	25.5	33.7	188	31.7	56	11.8	0.2	0.3
78.8	26.6	33.8	190	41.4	46.6	11.1	0.6	0.3
76.4	25.2	33	390	71	22.5	5	0.5	0
72.7	25.8	35.5	211	13	67	9	1	0
82.6	27	32.7	243	41	47	7	2	0
82.8	27.8	33.5	198	51.8	34.8	12.2	1	0.2
78.7	26.1	33.1	100	43	44.2	11.2	0.8	0.8
74.4	25.1	33.7	161	40.8	50.2	8.3	0.5	0.2
84.4	28.4	33.6	352	56.7	37.3	5.1	0.5	0.4
80.7	26	32.3	162	71	20	6	1	0
74.8	24.7	33.1	370	62.9	27.8	8.9	0.1	0.3
77.4	26.4	34.1	273	58	35	7	0	0
80.5	27.7	34.3	266	39.9	48.8	8.5	1.7	1.1
79.8	26.8	33.5	694	58	33	6	3	0
74.1	24.4	33	247	3	46	51	0	0
80.9	26.8	33.1	532	69	23	6	2	0
83.7	29	34.6	353	79.6	13.2	6.6	0.5	0.1
72.3	23.9	33	525	75.5	17.5	6	0	1
80.2	26.8	33.4	240	57.5	33.6	7.9	0.6	0.4
83.3	27.8	33.3	350	65.5	20	12.5	1.5	0.5
78.3	25.9	33.1	238	39.3	50	6.2	4.2	0.3
87.5	28.5	32.6	233	72	18.5	9	0.5	0
83.5	28.1	33.6	262	27.3	66.2	5.8	0.6	0.1
78	26.3	33.8	280	66	23	7	4	0
81.5	27.5	33.7	360	52	32	15	0	1
79.4	27.5	34.6	280	17	67	7.9	7.8	0.3
85.7	28.5	33.2	398	76.5	15	8.5	0	0
78.6	26.6	33.8	284	49.2	37.7	12.1	0.9	0.1

79.4	26.9	33.9	310	92.4	3.9	3.5	0.1	0.1
92.6	30.9	33.3	510	50	28	17	1	0
80.2	26.8	33.4	296	44	45	10	0	1
82.4	28.7	34.8	200	65.3	19.6	14.7	0.2	0.2
79.5	25	31.4	494	60	28	12	0	0
78.6	27.6	35.1	304	53	43	4	0	0
78.5	27.1	34.5	529	54	37.7	6.8	1.1	0.4
74.7	24.5	32.8	174	22	68	8	0	1
78.4	26.3	33.5	206	52.4	36.6	10.5	0.1	0.4
79.9	26.5	33.1	263	46	44	10	0	0
75.3	26.2	34.8	156	42.3	44	13.3	0.2	0.2
84.1	28.2	33.5	184	31	60	4	0	1
77.7	26.6	34.3	308	70	17.5	11.5	0.5	0
85.4	29.5	34.6	448	74.9	15.8	8.7	0.4	0.2
75.2	24.8	33	191	72	25.3	2.3	0.1	0.3
83	28.2	34	218	59	32	7	1	0
77.8	26.4	34	160	61.8	26.6	11	0.2	0.4
88.2	30.8	34.9	404	39.3	53.3	5.2	1.8	0.4
78.4	27.2	34.7	291	60.9	30.7	5.7	2.5	0.2
80.5	26.9	33.4	214	42	49	8	0	0
78.3	25.8	33	145	56.9	32.2	10.5	0.2	0.2
81.7	27.8	34	272	70.5	15	12	0.5	0.5
80.5	28	34.7	222	76.3	13.9	9.5	0.1	0.2
79.6	26.6	33.4	281	61.7	27.3	10.5	0.1	0.4
78.6	26.8	34.1	267	43	46	9	2	0
72.3	24.7	34.1	197	15	68	16	1	0
79.1	25.5	32.2	278	52	33.9	13	0.3	0.8
81.5	26.7	32.8	352	35	58.5	3	3.5	0
80.1	27.2	33.9	221	47.5	42	9.5	0.5	0
83.1	27.3	32.9	125	11	69	19	0	1
76.2	25.9	33.9	186	58.7	29.5	11.5	0	0.3
79.4	26.6	33.5	292	73	18.5	4.5	0	0
75.1	26.1	34.7	189	40	49.9	8.9	0.7	0.5
97.9	31.8	32.5	171	44.4	46.6	7.8	0.7	0.5
78.6	26.7	34	268	61	33	4	0	0
85.8	28.6	33.3	771	66.5	26	7.5	0	0
77	25.4	33	332	66.8	20.5	11.6	0.9	0.2
83.8	29.5	35.2	268	83.6	12.9	3.3	0	0.2
83.9	27.5	32.8	416	50	34	10	0	0
82	27.3	33.3	208	38	51	7	3	0
78.5	26.2	33.3	306	75.2	14.8	9.8	0	0.2
72.8	23.8	32.7	351	66	24	8.5	1	0
84.6	27.6	32.6	346	49.8	42.5	7.4	0.2	0.1
82.7	29	35.1	415	81.1	11.2	7.4	0.1	0.2
82.2	28	34	213	43.4	47.7	8.3	0.3	0.3
80.8	27.3	33.8	175	61.8	25.5	12.3	0.2	0.2

75.4	25.9	34.4	247	78.8	15.9	5.2	0	0.1
81.1	27.5	33.9	220	65.4	27.3	6.3	0.8	0.2
75.9	25.6	33.7	153	60.2	30.5	9.1	0	0.2
74.5	24.3	32.6	158	40.9	48.7	9.5	0.6	0.3
80.9	27.1	33.5	281	32.7	58.3	4.3	4.4	0.3
82.9	27.8	33.5	162	48.1	42.9	8	0.6	0.4
79.1	26.3	33.2	230	43	43	11	0	0
70.2	23.1	32.9	442	46	45	5	4	0
81.6	27	33.1	153	39	48	10	0	0
79.5	26.2	33	464	75	20	4	1	0
93.5	31	33.1	377	15	69	9	7	0
77.5	26.3	34	323	76	15	9	0	0
82.7	28.3	34.3	124	61	24	13	2	0
76.3	25.7	33.7	177	69.6	17.8	12.3	0.1	0.2
83.7	27.5	32.8	175	42.2	46.4	9.4	1.1	0.9
78.7	27.3	34.6	430	43	35.5	21	0.5	0
77.6	26.4	34	385	62.5	23	13	0.5	0
78.3	25.6	32.7	168	60.7	24.3	14	0.3	0.7
76.7	25.8	33.6	275	50	28	16	0	2
84.3	28.5	33.8	305	76.7	11	5	7.1	0.2
78.8	25.8	32.7	188	42	47	8	2	0
78.6	26.9	34.2	340	44.1	50.7	4.1	0.8	0.3
91.8	31.3	34	424	40	51	8	1	0
81.4	28.5	35	406	62.9	25.8	10.1	0.9	0.3
75.9	25.7	33.9	172	44.6	43.7	10.8	0.4	0.5
73	25.2	34.5	319	53.8	38.4	6.8	0.8	0.2
74.6	25.6	34.3	345	51.1	39	9.1	0.7	0.1
79.7	27.4	34.4	198	79.6	12.1	7.9	0.2	0.2
80.9	27.7	34.2	167	62.4	31.4	6	0.1	0.1
80.6	26.9	33.4	264	77.2	13.2	9.4	0.1	0.1
80.1	27.4	34.3	171	77.9	15.7	4.7	1.6	0.1
83.8	27.8	33.1	261	67.5	26.5	5	0	0.5
78.7	26.1	33.2	310	52	38	10	0	0
72.7	24.6	33.8	277	56.5	30	11.5	1.5	0
79.9	27.3	34.2	244	53.5	36.4	9.5	0.4	0.2
72.7	24.5	33.8	194	33	59	4	4	0
80	27.8	34.8	509	73.2	16.1	9.6	0.7	0.4
79.3	26.6	33.5	173	66	20	8	1	0
97.5	33.5	34.4	246	15.7	64.3	13.7	5.9	0.4
75	25	33.3	227	31.1	57.4	10.3	0.9	0.3
80.3	27.7	34.4	333	55.1	27.6	14.4	2.7	0.2
72.5	24.2	33.3	233	70.2	18.7	10.1	0.7	0.3
75.1	24.9	33.2	234	55	34.9	8.7	0	1.4
80.9	27.6	34.1	177	76.9	15.9	6.9	0	0.3
77.6	26.8	34.5	209	87	11	2	0	0
79.9	25.9	32.4	201	57.2	30.6	11.9	0.1	0.2

83.7	27.3	32.6	170	4	81	15	0	0
77.5	25.7	33.1	172	34.1	52.5	11.7	1.2	0.5
84.6	27.5	32.5	104	59	34.3	6.1	0.3	0.3
77.4	26.8	34.6	112	23.8	69.3	5.9	0.5	0.5
81.8	28.5	34.8	489	54	31	15	0	0
75.1	25.1	33.4	409	51	40	6	3	0
80.3	27.4	34.1	247	39.3	49.4	7.3	3.7	0.3
79.3	26.5	33.4	218	52	36	10	1	0
83.1	28.8	34.6	291	45.3	42.1	10.4	1.9	0.3
77.5	25.5	33	261	48.8	45.2	5.5	0.4	0.1
74.8	26.8	35.8	293	59	33.9	6.9	0.1	0.1
77	25.8	33.5	135	32.8	55	11.2	0.8	0.2
75.4	25.4	33.6	323	53	33	11	1	0
93.5	31.8	34	302	22.9	59.1	12.9	4.7	0.4
80.4	26.6	33	130	26.1	65.3	6	2	0.6
78.5	25.9	33.1	334	81	9.5	9	0	0
81.7	27.8	34	193	54	42	4	0	0
73.7	24.7	33.5	220	54.3	33.7	10.7	0.9	0.4
72.6	24.1	33.1	192	44.7	51.1	3.3	0.7	0.2
73.9	25.8	34.9	206	81.3	10.4	7.9	0.1	0.3
72.7	24.7	34	329	78.2	10.4	11	0.2	0.2
82.1	27	32.8	179	60.9	23.3	13.2	1.8	0.8
70.6	24.8	35.2	376	58	32	10	0	0
81.6	27.7	33.9	257	50.2	38.4	10.6	0.2	0.6
79.6	26.5	33.3	296	73	20	5	0	0
76.1	26.3	34.6	145	43.7	46.7	8	1.2	0.4
76.5	25.8	33.7	296	59	30	10	0	1
89.3	29.8	33.3	421	32.1	51.3	11.1	5	0.5
78.6	27.3	34.8	344	19.1	65	14.4	1.3	0.2
84.9	27.9	32.9	291	59.8	29.3	9.8	0.3	0.8
73.7	24.4	33.1	436	34	52	11	0	0
77.3	26.6	34.4	124	45.9	47.4	5.9	0.4	0.4
76.9	26.1	33.9	145	40.6	47.8	11.3	0	0.3
77.1	26.9	35	517	36.2	56.1	4.3	2.8	0.6
81.2	27.4	33.8	229	58.3	25.1	14.7	1.4	0.5
87.5	30.8	35.2	193	57	28	14	0	0
76.1	25.8	33.9	229	82	14	4	0	0
104.8	35.9	34.2	239	48	28	20	2	1
79.2	24.7	31.2	144	27	61.3	7.7	2.4	1.6
69.7	22.4	32.1	117	51.8	37.3	10.5	0.2	0.2
80.7	27.3	33.8	162	59	26	15	0	0
79.6	26.5	33.3	230	72	19	7	1	0
78.7	26.3	33.4	241	63.3	23.1	12.2	0.9	0.5
82.8	28.2	34	149	46.6	46.2	6.6	0.3	0.3
72	23.3	32.3	291	42	46	11	0	1
68.2	21.2	31.1	386	65.8	26.9	6.2	0.9	0.2

67.9	21.9	32.3	218	53.1	38.3	8.3	0	0.3
74.6	25.1	33.7	205	50	36	3	3	1
79.9	28	35.1	225	81.1	13.5	5.2	0	0.2
79.3	27.3	34.4	371	75	19	5	0	0
78.6	25.8	32.9	281	42.1	51.2	6.5	0	0.2
78.8	26.4	33.5	352	67	18	10	0	0
81.6	28.7	35.2	275	56.6	29.5	7.5	6	0.4
81.2	27.1	33.4	171	43	45	12	0	0
83.4	27.4	32.8	182	31	55.7	13.1	0	0.2
75.2	25.4	33.8	211	55.3	37.2	6.9	0.4	0.2
75.6	25.9	34.3	221	29.1	62.2	8.5	0	0.2
76.4	25.3	33.2	257	49	39.5	5.5	1	0
76.1	24.9	32.7	212	28	54	13	1	1
82.9	27.5	33.1	427	75.9	17.6	6.2	0.1	0.2
77	24.6	32	190	59.9	32.2	6.9	0.7	0.3
81.8	28.1	34.3	222	63	28	3	6	0
80.7	25.9	32.1	221	49	39	8	2	0
73.3	24.5	33.4	219	68.8	26.1	4.5	0.3	0.3
72.9	24.4	33.4	210	36.4	49.1	11.5	2.6	0.4
80.2	27.4	34.2	143	69.3	20.3	9.3	0.8	0.3
81.8	28	34.3	114	32	57	5	0	0
81	27.1	33.4	145	50.5	40.9	7.6	0.7	0.3
73.3	25.1	34.3	199	54.8	30.9	13.6	0.5	0.2
81.6	27.7	34	185	16	74	9	0	1
81.3	26.9	33.1	171	45.4	44.1	9.1	0.5	0.9
76.7	25.1	32.7	389	32.5	56.5	9.7	1.1	0.2
78.7	26.2	33.2	117	10	78	8	1	1
65.6	20.9	31.9	464	52	32	12	2	1
78	27	34.6	115	66	28	5	0	0
81.1	26.8	33	99	45.9	43.8	8.5	0.9	0.9
81.2	28.1	34.6	131	64.5	25.5	10	0	0
77.6	26.3	33.9	409	48.2	47.7	2.5	1.5	0.1
82.1	27.6	33.6	274	79.5	11.5	7	0	0
70.3	23.4	33.3	169	53.1	34.3	10	1.9	0.7
80	25.3	31.6	362	66	19	11.5	0	0
77.2	26.1	33.8	337	48	39.6	6.8	5.5	0.1
74.1	24.8	33.4	181	52.6	37.7	8.8	0.6	0.3
79.4	26.5	33.3	127	78.4	12.8	7.9	0.8	0.1
79.8	26.1	32.7	165	17	73	6	3	0
99.1	31.6	31.8	615	53	28	11	0	1
82.9	26.9	32.5	177	34.5	54	10.4	0.4	0.7
95.1	32.5	34.1	437	31	55	9	5	0
81.5	27.9	34.3	188	58	32	8	0	0
83.4	28.3	33.9	164	48	40.1	10.7	1	0.2
87.6	28.9	33	230	61	23	14	0	0
75.7	25.2	33.2	169	34	58	7	0	0

79.2	26	32.8	144	66.9	23.9	9.2	0	0
87.3	27.2	31.2	241	46	40	12.5	0.9	0.6
84.2	29	34.4	484	73	23	4	0	0
78.6	27.3	34.7	339	43.4	40.4	12.3	3.5	0.4
75.2	25.1	33.3	345	28	64	6	1	0
74.4	24.5	33	184	81.1	15.9	2.8	0	0.2
79	26.3	33.3	277	75.5	16.4	7.8	0.1	0.2
79.5	27.6	34.7	245	59.9	26.4	12.7	0.5	0.5
78.9	25.4	32.2	482	52	36	12	0	0
82.1	27.5	33.4	155	47.8	46.3	5.5	0.1	0.3
75.3	25.7	34.1	308	37	55	8	0	0
72.1	23.6	32.8	430	59.2	36.1	3.7	1	0
67.9	21.8	32.1	252	61.5	30.5	7.6	0.3	0.1
76	25.7	33.9	236	43	43	12	1	1
80	26.4	33	196	40.2	47.1	12.2	0.1	0.4
76.5	25.8	33.7	296	60	32	3	3	0
67.9	22.6	33.2	212	34.2	54.5	10.9	0	0.4
76.6	25.1	32.8	149	20.6	70.1	5.2	1	0
74.5	24.9	33.4	135	34	53	11	0	0
81.3	27.2	33.4	292	65	28	6	0	0
82.6	27.4	33.1	362	57	30	9	1	0
84.5	27.8	32.9	134	34	46	11	0	0
77.1	26.7	34.6	88	38.8	48	12.7	0.4	0.1
76.7	25.9	33.8	214	51	36	7	1	1
80.6	26.4	32.8	183	57.7	31.5	9.7	0.9	0.2
77.6	25.8	33.2	255	35	55.6	7.9	1.3	0.2
78.1	25.6	32.8	220	55	29	4	0	0
82	26.8	32.7	248	48	29	19	3	0
80	26.7	33.4	195	77.1	18	4.8	0	0.1
78.7	26.7	34	225	70.2	19.9	8.1	1.6	0.2
77.8	27	34.6	283	67.2	22.1	10.3	0.2	0.2
80.1	28.3	35.3	229	69.4	25	4.1	1.3	0.2
83	27.6	33.2	242	44	55	1	0	0
74.9	25.9	34.6	336	44	43	13	0	0
80.7	27.8	34.5	225	57.2	33.5	8.8	0.2	0.3
78.5	26.5	33.7	156	6	92	2	0	0
76.7	27.5	35.8	152	34.3	53.6	11.3	0.4	0.4
77.8	25.9	33.3	328	56	33	7	2	0
82	27	32.9	445	17	78	3	2	0
89.5	30.2	33.7	437	20.4	62.2	11.9	4.9	0.6
79.4	25.9	32.7	370	55	35	9	0	1
78.9	25.9	32.8	280	34.8	58.2	4.6	2.1	0.3
88.2	29.6	33.5	367	23.1	62.5	8.9	4.6	0.9
77.1	25.6	33.1	200	31.8	52.4	14.2	1.1	0.5
73.4	23.8	32.5	135	50.6	40.7	6.9	0.6	1.2
83.6	28.8	34.4	61	8	82	10	0	0

70.6	23.3	33	109	27.8	59.9	8.1	4	0.2
78.2	26.8	34.3	256	74.2	20	5.2	0.4	0.2
79.3	27.1	34.2	413	48	46	4	2	0
82.3	27.2	33	393	66	25	9	0	0
79.4	26.8	33.8	238	58	31	10.8	0	0.2
83.1	28.9	34.8	182	8	70	10	0	1
77.2	26	33.6	280	56.4	29.4	12.2	1.4	0.6
76.2	24.3	31.9	288	31	53	14	0	0
78.8	26.9	34.2	322	87.2	8.8	3.7	0.1	0.2
85	29	34.1	260	58.3	34.1	7.1	0.1	0.4
81.4	26.5	32.5	301	42	50	8	0	0
82.5	28.1	34.1	514	44.2	50.1	5	0.3	0.4
81.1	26.6	32.9	236	59	34	6.4	0.3	0.3
82.2	27.7	33.7	176	72.4	19.9	7.5	0	0.2
79.2	26.6	33.5	183	45	34	15	4	0
81.6	27.2	33.3	131	57	32	11	0	0
79	26.8	33.9	276	50.5	37.2	10.1	2.1	0.1
80.9	27.2	33.7	146	38.8	50.6	8.3	1.7	0.6
82.5	28.2	34.1	322	45	44	11	0	0
79.2	26.4	33.3	320	78.8	13.5	7.1	0.4	0.2
80.2	27.9	34.7	171	47	44.6	8.2	0	0.2
87.1	29.8	34.2	115	44.4	47.5	6.9	0.6	0.6
90	30.4	33.8	284	10	70	17	0	0
82.5	27.5	33.3	209	49.9	41.7	7.9	0.2	0.3
82.6	27.6	33.4	209	35.1	51.5	12.9	0.2	0.3
85.5	28.3	33	186	32	59	8	0	0
83.6	28.6	34.2	263	59.6	35.5	4.5	0.3	0.1
81	26.7	32.9	183	65	23	7	1	0
78.1	26.3	33.6	167	20	67	9	0	0
75.5	25.5	33.8	222	35	55.5	8.9	0	0.6
76.7	24.8	32.3	267	71.4	18.2	10	0.2	0.2
80.5	27	33.5	236	22.5	61.8	11.4	3.6	0.7
81	27.5	33.9	407	54	30	12	1	1
85	27.4	32.3	220	25	64	10	1	0
79.2	26.8	33.8	226	28.2	55.2	11.3	4.1	1.2
78.6	26.5	33.7	193	48.5	38.3	12	1	0.2
81.7	27	33.1	154	19.2	65.7	10	4	1.1
79.5	26.7	33.6	213	73.3	21.1	5.3	0.1	0.2
79.4	27.1	34.1	290	62.6	22.9	12.8	0.9	0.8
79	26.9	34.1	306	63	22	11	0	0
80.1	26.7	33.3	168	51	31	14	4	0
78.3	25.6	32.7	253	45.6	43.4	10	0.6	0.4
78.6	27	34.4	235	68.5	23.9	3.5	3.9	0.2
78.1	27.1	34.7	438	62	29	6	1	0
77.5	27.1	34.9	176	29.9	58	9.6	2	0.5
80.3	26.4	32.9	341	35	41	20	2	0

73.2	23.4	32	237	63.1	29.7	6.6	0.3	0.3
81.2	27.7	34.1	245	68	23	8	0	0
81.4	26.9	33	149	40	45.9	13.5	0	0.6
94.2	32.3	34.3	43	3	83	11	0	0
74.6	24.5	32.8	229	57	22	11	2	0
79.8	26.5	33.2	193	14	56	22	4	0
78.9	26.1	33.1	243	40	31	9	0	0
84.5	28.8	34	234	50	34	15	1	0
77.2	26.5	34.4	124	25.1	60.6	12.5	0.3	1.5
80.2	26.5	33.1	246	54	39.2	6.4	0	0.4
81	27.2	33.5	333	17	78	2	1	0
76.2	25.2	33.1	262	43.4	45.7	9	1.5	0.4
81	27.3	33.8	225	22	69	5	3	0
83.8	27.2	32.5	215	73	13	10	0	0
81.6	27.7	34	290	70.7	17	11.5	0.4	0.4
85.8	28.7	33.4	224	43	45.3	11	0.2	0.5
79.2	26.9	34	298	46.1	37.3	14.6	1.5	0.5
76.1	25.9	34.1	437	56.1	31.8	7.9	4	0.2
78.6	25.3	32.1	339	67	24.5	8.5	0	0
75.1	23.9	31.8	297	57	33	8	0	0
79	27	34.2	208	54	31	15	0	0
83.9	27.5	32.7	253	58	29	10	1	0
79.5	26.4	33.2	228	65.5	19.4	11.2	3.5	0.4
78.3	25.8	33	203	44.3	43	11.9	0.2	0.6
77.8	26.3	33.8	186	22	63	9	1	0
81.4	28.1	34.5	254	43.3	48	7.9	0.4	0.4
81.1	26.8	33.1	316	24.1	67.6	6.3	1.4	0.6
77.5	25.8	33.2	217	58.8	31.6	7.6	1.8	0.2
75.8	25.8	34	222	36.8	51.2	10	1.7	0.3
92.5	31.2	33.8	294	49	29	16	4	0
75.8	24.4	32.2	136	58.1	31.7	9.2	0.9	0.1
76.6	25.9	33.8	237	34.2	51.8	12.3	1.3	0.4
76.1	25.4	33.4	171	43	43.7	12.6	0.2	0.5
79.7	26.1	32.8	168	49.4	41.1	8.5	0.6	0.4
77.6	25.9	33.4	346	33.9	59.8	3.4	2.8	0.1
81.8	27.5	33.6	202	68	21	8	1	0
77.8	25.1	32.3	446	57	31	11	0	0.5
79.9	26.9	33.7	287	47	20	27	5	1
81.7	28.7	35.1	419	30	68	2	0	0
89.5	28.3	31.6	166	42.6	46.4	9.9	0.2	0.9
81.1	28.7	35.4	183	74.4	13.2	11.8	0.4	0.2
109.6	36.2	33	453	55	31	14	0	0
72.5	23.6	32.6	344	8	72	15	2	0
78.3	26.8	34.2	161	55.3	32.9	11.2	0.3	0.3
77.6	26.5	34.2	150	39	49.4	10.2	0.8	0.6
70.2	22.4	31.9	283	31	56	12	0	0

79.2	28.3	35.8	155	79.6	10.2	9.3	0.6	0.3
79.1	26.1	33	340	70	24	6	0	0
81.4	27.1	33.2	223	55	37	8	0	0
79.2	26.3	33.3	244	77	17	2	0	0
76.7	26.1	34	262	68	22	10	0	0
78.3	25.9	33	295	72.3	14.5	12.8	0.1	0.3
79	26.7	33.7	346	58.4	30.5	9.4	1.4	0.3
80.2	27.2	33.9	192	60.7	28.2	10.5	0.3	0.3
72.6	23.9	32.9	347	70.4	21.9	7.5	0.1	0.1
80.8	26.9	33.3	242	55.8	34.5	7.8	1.6	0.3
76	26.5	34.9	372	52	23	18	3	1
79.9	27.3	34.2	425	61.6	31.4	4.8	2.1	0.1
78	26.1	33.4	351	42.1	41.1	9.9	6.8	0.1
81.7	26.1	31.9	618	66.7	25.3	5	2.8	0.2
81.9	26.6	32.5	523	37	44	9	8	1
77.9	26.4	33.8	325	91.9	3.6	2.3	2	0.2
81.9	28.1	34.4	391	62.2	29.2	4.5	4	0.1
83.5	28.8	34.5	287	88.7	6.6	3.7	0.8	0.8
79.6	26	32.6	716	40	47	11	1	1
78.1	26.8	34.3	315	71.6	24.2	3.9	0.1	0.2
77.4	26.7	34.6	514	48.6	41.3	5.8	3.8	0.5
86.4	28.7	33.2	383	37.8	51.5	7.7	2.6	0.4
83.7	28.1	33.5	405	37	51.5	6.3	5	0.2
85.2	28.6	33.6	321	72.9	20	5.3	1.6	0.2
75.6	25.3	33.4	235	41	42	15	2	0
79.9	26.5	33.2	292	74	12.5	3.5	2	0
84.1	28.1	33.4	318	72.5	20.6	4.4	2.4	0.1
82.7	28	33.8	174	76.8	15.4	3.6	3.6	0.6
78	26.5	34	291	59.5	31.4	5.2	4	0
77.9	26.2	33.6	395	7	74	12	1	0
82	27.3	33.3	275	65	21	7	2	0
79.5	25.4	31.9	534	72	25	2	0	0
75.4	25.5	33.9	116	58	33	9	0	0
74.8	24.4	32.6	380	75	21	3	0	0
80.1	27.3	34.1	493	88	7.5	4	0	0
79.2	27.3	34.5	217	53.1	26.4	15.5	4.5	0.5
79.1	27.5	34.7	128	73	11	16	0	0
80.3	27.7	34.5	321	65.4	22.3	10.1	0.9	0.3
82.6	27.5	33.3	479	82.3	12.3	2.6	2.6	0.2
75.4	24	31.8	555	59.1	34.9	5.3	0.5	0.2
89.9	29.6	32.9	541	68	18	11	1	0
79.5	26.5	33.3	207	54	28	7	3	0
75.1	24.4	32.4	426	74	9	4	1	0
63.6	19.7	30.9	325	59	30	6	3	1
79.9	26.7	33.4	447	41.8	47.5	4.7	5.8	0.2
82.1	27.7	33.8	373	69	23	4	4	0

74.9	25.7	34.4	299	74.6	17.6	4.1	3.6	0.1
81.2	26.6	32.8	263	42	38	15	0	0
78.1	26.6	34.1	129	79.4	9.8	1.2	9.3	0.3
87.1	28.9	33.2	362	46	44	8	2	0
73.9	25.5	34.5	264	45	47	5	1	0
78.9	25.7	32.6	442	65	25.7	6.2	2.8	0.3
77.9	26.4	33.9	378	52	29	10	5	0
68.2	22.9	33.6	326	34.7	50.1	9	5.8	0.4
78.7	25.7	32.7	338	56	35	3	6	0
76.1	25.5	33.5	227	76	20	3	0	0
85.3	28.3	33.2	371	63.1	30.6	5.6	0.3	0.4
78.1	26.5	33.9	466	48.4	39.5	10.5	1.4	0.2
77.9	26.2	33.6	239	78	8	9	3	2
74.9	25.9	34.6	960	69	17	4	4	0
81.5	27.3	33.5	208	35.3	50.9	4.6	8.8	0.4
77	25.6	33.2	687	42	39	17	1	1
75.6	26.4	34.9	290	71.9	20.8	5.9	1.1	0.3
81.4	27	33.1	206	50	35	13	0	0
76.1	26.7	35.1	154	76	13	2	0	0
75.9	25.1	33	375	50.8	39.7	7	2.4	0.1
79.3	26.4	33.2	463	49	45	4	2	0
81.9	27.2	33.2	318	57	31	10	2	0
78.2	26.4	33.8	425	59	35	2	4	0
77.5	26	33.6	221	74	19	7	0	0
71.7	23.7	33	412	69	25.5	4	0	0
78.9	26.8	33.9	290	75	13	4	7	0
80.2	26.3	32.8	572	71	16	8	1	0
80.9	27.9	34.4	330	71	13	5	2	0
78.2	26.2	33.5	299	62.7	29.6	4.3	3.1	0.3
83.7	27	32.3	447	67	24	2	5	0
55.6	18.8	33.7	292	74	20	4	1	0
74.5	25	33.5	859	46	47.5	6	0.5	0
77	25.8	33.5	580	54.9	35	6.4	3.4	0.3
80.7	26.8	33.2	135	74	15	3	7	0
70.8	24.4	34.5	431	75	18	3	1	0
73.1	24	32.8	400	63	33	3	1	0
78	26.2	33.5	282	59	30	9	0	0
80.7	27.2	33.7	268	44.3	49.1	3.9	2.3	0.4
76.4	24.9	32.6	327	39.3	43.5	6.2	10.7	0.3
78.5	26.7	34.1	156	78	13	2	1	0
75	25.5	34	244	71	18	4	2	1
82.7	28.5	34.4	326	69	18	5	6	0
83.3	28.3	34	387	75	21	4	0	0
82.1	26.5	32.3	291	76	9	7	1	0
82	28.2	34.3	225	68.2	20	7.7	3.9	0.2
80.3	27.9	34.7	429	46.4	40.5	9.4	3.5	0.2

77.6	25.4	32.8	779	47	39	14	0	0
75.7	24.8	32.7	455	29	66	4	0	0
59.4	19.9	33.5	499	66.8	25	3	5.1	0.1
83.7	27.6	33	355	56	31	7	6	0
84.4	28.8	34.1	318	63	28	8	1	0
80.5	26.6	33.1	394	66	25	6	3	0
80.3	26.2	32.7	301	43.5	46.2	5.2	4.8	0.3
86.9	29.6	34.1	169	70.1	22.6	6.8	0.4	0.1
81.9	26.6	32.5	400	65	22.6	7.8	4.3	0.3
80.3	27.7	34.5	217	76.1	15.9	7.8	0.1	0.1
73.4	24.1	32.9	482	65	22	8	5	0
83.4	27.2	32.6	246	70	20.1	9	0.6	0.3
76.6	25.3	33	191	88	9	1.5	0.5	0
80.2	27.4	34.2	447	60.1	29.3	4.9	5.5	0.2
73	24.3	33.3	418	49	41	7	3	0
57.4	18.5	32.3	290	32	45	12	8	0
75.1	25.2	33.6	315	50.3	41.6	6.4	1.5	0.2
79	26.9	34.1	199	21	76	3	0	0
78.8	25.9	32.9	383	53.3	31.8	6	8.6	0.3
82.7	27.9	33.8	221	79	14	5	0	0
77.7	26.4	34	256	58	23	5	7	0
74.2	24	32.3	398	48.1	40.1	3.8	7.7	0.3
84.1	28.5	33.9	270	68.7	23.2	6.1	1.8	0.2
77.4	26.4	34.2	353	78.7	12.3	4.2	2.6	0.2
75	24.5	32.7	580	36	56	2	6	0
72.5	23.2	32	466	58	32	5	4	0
76.5	25.8	33.7	267	52	34.8	4.9	8.1	0.2
82.9	28	33.8	374	64.1	23.5	6.5	5.7	0.2
77.3	25.5	33.1	359	65.7	22.6	6	5.4	0.3
78.1	26.3	33.6	323	55	39	5	1	0
76.1	26.3	34.6	675	39.7	52.7	4.2	3	0.4
77.9	26.8	34.4	282	67.4	26.1	3.5	2.8	0.2
75.3	24.9	33.1	151	17.9	74.7	5.3	0.7	1.4
77.3	25.2	32.6	513	56.6	34.4	4.1	4.8	0.1
81.3	26.9	33.2	501	85.5	7.5	3	0.5	0
79.6	27.5	34.5	492	50	39	10	0	0
57.2	18.5	32.4	228	49.1	38	7.2	5.5	0.2
84.7	26.5	31.3	452	53.7	35.2	9.9	1	0.2
83.3	27.3	32.7	499	71	17.5	6.5	4	0
81.3	27.8	34.2	400	57.4	38	3.2	1.1	0.3
77.7	26.1	33.5	468	59.3	31.7	7	1.8	0.2
79.1	27.3	34.5	248	77.5	17.1	3.6	1.6	0.2
78.1	26.5	34	255	44.6	45.3	7.5	2.2	0.4
79.6	25.7	32.2	439	58	25	9	4	0
73.7	24.7	33.6	270	67	25.5	4	1	0
77.5	25.6	33	257	61.9	28.7	5	4.3	0.1

84.4	27.9	33.1	337	49	43	7	1	0
79.2	26.7	33.7	280	66	23.9	8.2	1.6	0.3
79.3	25.6	32.3	268	48	23	5	19	0
80.8	27.1	33.6	252	66.4	24.9	4.9	3.4	0.4
75.9	26.2	34.5	288	66.6	27.5	4.4	1.2	0.3
84	27.1	32.3	337	52.2	40.4	4.5	2.7	0.2
79.1	27	34.1	319	59	32	5	4	0
81.3	27.5	33.8	229	30	46	12	12	0
77.5	25.8	33.2	412	62	28	8	2	0
77.7	25.6	32.9	186	54	32.9	11.1	1.5	0.5
83.6	28.2	33.7	182	83	11	0	4	0
78.2	25.5	32.6	245	50.6	33.8	10	5.3	0.3
80.4	26.6	33.1	188	57.1	36.1	4.1	2.5	0.2
72.1	23.8	33	245	63.3	27.4	4.6	4.5	0.2
69	22.2	32.2	266	42.6	44.5	5.8	6.9	0.2
73.3	25.2	34	325	39	26.5	10.5	4.5	0
85.3	28	32.9	162	46.7	40.9	9.1	2.2	1.1
79.6	25.4	31.9	259	48.6	35.9	13	2.2	0.3
75.5	25	33.2	257	39	51	3	3	0
79.6	25.7	32.2	379	60	28	5	3	0
60.5	19.2	31.7	165	77	13	7	1	1
82.1	27.4	33.3	271	47.3	45.8	5.2	1.5	0.2
72.8	23.6	32.5	539	60.6	30.6	3.6	4.5	0.7
82.2	27.3	33.2	239	66.3	25.4	7.8	0.4	0.1
78.2	27	34.5	300	53	24	20	1	0
58.9	18.3	31	319	62.5	28.8	4.4	4.1	0.2
81.4	26.1	32.1	404	61.5	33	4	1	0
80.6	27.6	34.3	227	81	14.1	4.2	0.5	0.2
75.5	24.2	32.1	284	69.5	23.3	4.3	2.5	0.4
78.4	26	33.2	357	69.6	23.6	4.9	1.5	0.4
78.3	26.2	33.5	253	62	20	3	1	0
79.1	26.3	33.2	327	55.5	33.3	2.9	8.1	0.2
82.4	28.7	34.8	310	71.2	23.7	3.2	1.8	0.1
56.5	18.5	32.7	317	73	20	6	0	0
84.1	28	33.3	469	56	33.8	4.8	5.3	0.1
79.5	27.1	34.1	337	55	27	13	0	0
84	27.8	33.1	417	55.8	33	8.2	2.6	0.4
78.6	26.8	34.1	242	47	40	8	3	0
77.6	25.2	32.5	430	54	30	7	8	0
77.5	25.7	33.1	294	48	42.4	7.7	1.5	0.4
77.3	26.1	33.8	397	63.7	27.9	5.2	2.8	0.4
79	26.6	33.6	266	40	48	6	6	0
78.7	26.6	33.8	469	74	23	2	1	0
78.7	25.2	32	579	38	45	8	3	0
79.6	25.8	32.4	742	1	20	4.4	2	1.3
78.7	26.6	33.8	383	56.1	34.4	4.2	5.1	0.2

73.7	24.6	33.4	264	47.4	45.2	3.6	3.7	0.1
79.8	27	33.9	411	53	32	12	0	0
78.8	25.6	12.5	266	39	41	8	10	0
78.3	26.5	33.8	405	56	30	8	2	0
79.8	26.7	33.5	180	70.5	24.5	4.5	0	0
78.4	26.1	33.3	353	66.3	26.3	3.2	4	0.2
78	26.6	34.1	422	66	23	2	4	0
83	28.1	33.8	273	28	58	8	2	0
79.9	25.4	31.8	311	72	18.5	7.5	0	0
79.1	26.6	33.6	632	48	38	11	0	0.5
78.4	25.5	32.5	265	70.5	18	6.5	1.5	0
81	27.5	33.9	265	73.9	18.7	3.1	4.2	0.1
78.2	26.9	34.4	366	67.7	23.6	7.9	0.7	0.1
85.7	29.4	34.3	571	55.5	25.5	5	1	0
79.5	26.1	32.9	296	57	32	6	1	0
82	27.1	33.1	213	76.7	15.7	4.8	2.3	0.5
82.7	27.3	33	370	38	53	7	2	0
81.7	27.3	33.4	332	72	21	6	1	0
93.3	27.6	33.1	333	58.4	33.7	5.6	2.1	0.2
78	25.7	32.9	335	42	39.1	6	12.4	0.5
79.8	27.5	34.4	333	38.3	51	6	4.3	0.4
80.8	27.6	34.1	330	61	29.8	8.4	0.8	0
77.8	25.9	33.3	213	65.2	23.1	10.1	1.3	0.3
72.3	24	33.1	421	41.6	48.5	6.1	3.3	0.5
83.7	28.6	34.2	220	74.6	19	6	0.2	0.2
80.3	27.5	34.3	248	58	24	9	8	0
78.1	26.3	33.7	457	37	42	11	7	0
83.9	28.2	33.6	157	73	19	7	0	0
80.7	25	32.1	285	49.3	38.3	8.3	3.7	0.4
78.2	26	33.2	413	72.9	19.8	6.4	0.7	0.2
81.7	27.8	34	629	53	37	6	3	0
78.6	25.7	32.6	373	38	45	6	7	1
81.7	29	35.5	292	77.1	17.4	4.4	1	0.1
78.5	27	34.3	456	65.7	24.2	6.8	3.2	0.1
75.9	25.7	33.8	423	72	18	3	3	0
73.5	24.2	32.9	183	30	47	5	16	0
79.6	27.8	35	478	37	47	7	7	0
82.4	28.9	35	341	46	46	7	1	0
73.9	24.3	32.9	282	66	26	6	1	0
73.1	23.9	32.7	345	40	45	5	9	1
80.3	26.9	33.6	801	57	30	5	5	0
85.1	28.6	33.6	234	61.4	24.3	11.5	2.5	0.3
75.9	26.5	35	314	36.9	44.7	11.4	6.6	0.4
79.8	27.4	34.3	215	47	40	5	6	0
79.4	26.3	33.1	293	70.9	17	8.7	3	0.4
78.9	26.8	33.9	338	67.6	24.7	4.2	3.4	0.1

74.2	25.4	34.2	440	35.5	51.5	11.5	1.2	0.3
77	26.7	34.7	513	52	40	6	0	1
80.7	26.7	33	370	42	41	4	8	1
74.4	24.2	32.6	355	60.6	23.6	6.2	9.3	0.3
79.1	26.5	33.4	169	10	84	5	0	0
80.7	26.4	32.7	375	80	12	3	0	0
81.3	27.5	33.8	459	62.5	26.7	4.2	6.2	0.4
78.9	27.4	34.7	265	43	40	13	4	0
58.1	18.9	32.6	358	69	23.5	5	1	0
79.3	27.8	35	549	60.1	36	1.6	1.6	0.7
82.1	28.6	34.8	188	76	19.5	4.5	0	0
78.4	27.3	34.8	277	60.5	31.7	5.1	2.5	0.2
80	28.4	35.5	319	43	43	9	0	0
77.3	25	32.3	277	50	41	3	6	0
83.3	28.2	33.9	309	56.6	34.4	5.6	3.1	0.3
80.1	26.3	32.9	369	63	23	8	4	2
66	21.5	32.5	439	68	28	2	2	0
85.1	28	33	282	73	23	3	1	0
83.3	28.4	34.1	428	81	12	4.5	0.5	0.5
73.3	25.2	34.4	249	44.1	41.5	6.1	8.1	0.2
59.1	18.5	31.3	579	40	32	9	17	0
75.5	25.5	33.8	469	37	46	12	5	0
78.1	26.5	34	301	31	62	5	2	0
81	27.1	33.4	251	71.6	18.5	6.6	3	0.3
62.3	19.7	31.6	417	49	35	10	5	1
81.1	26.2	32.3	580	72.5	20	5.5	1	0
83.6	28.2	33.8	450	52.1	26.8	6.1	13.9	1.1
75.6	25.1	33.1	219	47.6	49.3	0.5	2.3	0.3
78.5	26.5	33.8	441	55	35	5	3	0
79.7	26.2	32.8	366	31	41	6	18	0
82.8	27.8	33.5	340	61.8	31.6	3.7	2.8	0.1
58.8	19.1	32.5	328	63	21	11	3	1
82	26.7	32.5	249	59	32	4	0	0
76.3	24.3	31.9	286	64.7	23.7	7	3.3	0.3
76.9	24.8	32.2	974	62	29	7	1	1
73.9	23	31.1	1073	20	74	3	2	0
79.2	26.5	33.4	480	73.7	19.2	6	0.9	0.2
79.4	25.6	32.2	748	47.5	39.5	6.5	5	0
78	25.8	33	311	75.1	17.3	5.2	2.2	0.2
85.4	27.3	32	344	49.2	39.9	4.7	6	0.2
78.9	26.8	34	436	84	6	1.5	4.5	0
78.9	25.7	32.6	274	65	23	3	7	0
81.2	26.6	32.8	119	30	50	7	1	0
78.3	25.8	33	379	39.5	51.2	4.7	4.1	0.5
80.3	27.2	33.9	262	72.5	18.8	6.6	1.9	0.2
69	21.8	31.6	342	35.2	56.8	4.6	3.3	0.1

78.8	26.4	33.6	315	63	26	6	0	0
70.6	22.4	31.7	427	28.1	56.1	14.9	0.7	0.2
83	27.6	33.2	450	45	26	13	7	0
78.4	26.8	34.1	253	58.5	27.3	8.2	5.8	0.2
82.5	27.1	32.8	357	75	10	3	2	0
77.6	26	33.5	255	79.4	13.6	5.8	0.8	0.4
84.4	27	32	552	44	41	8	0	0
70.8	23.5	33.2	326	52	37.6	9.9	0.3	0.2
82.3	27.8	33.8	297	63.4	28.4	5.2	2.8	0.2
74.1	24	32.4	463	41.8	49.1	5.1	3.7	0.3
79.1	26.1	33	306	60.9	25.3	5.2	8.2	0.4
81.3	26.2	32.2	332	47	45	4	3	0
77.8	26.3	33.8	266	33.6	54.2	10.4	1.2	0.6
76.6	25.9	33.8	233	43	44	9	2	0
82.2	27.5	33.4	335	82	7	5	1	0
78.8	25.8	32.8	351	37	42	10	8	0
85.5	27.9	32.6	421	62	26	6	4	0
75.3	25.8	34.3	40	85	9	1	1	0
81.5	27.8	34.1	368	65	24	4	3	1
77.7	26.5	34.2	148	3	93	3	0	0
74.1	24.8	33.5	207	79	3	3	11	0
82	27.1	33	495	69.5	21	4	4	0
75.1	25.2	33.5	422	68	17	4.5	7	0
80.5	25.8	32	279	67.2	22.4	9.7	0.4	0.3
77.2	26.1	33.8	216	41	50	2	4	0
79.6	27.5	34.6	339	85	5	4	2	0
80.5	27.2	33.8	248	58	37	3	0	1
82.8	27.9	33.7	462	71	21	5.5	2	0
82.1	27.9	33.9	454	63	27	10	0	0
79.1	27	34.1	382	64	30	2	4	0
77.9	26	33.4	406	68	15	8	2	0
74.8	24.6	32.9	473	63	23	4	6	0
83.1	27.9	33.5	84	70.7	19	6.3	0.3	0.3
77.9	25.2	32.4	163	54	35	6	1	1
78.7	25.9	33	191	31	54	8	0	1
81.6	28	34.4	517	71	21	4	1	0
77.8	25.3	32.5	222	43	46	6	2	1
77.1	26.2	34	292	29.5	58.5	3.5	8.4	0.1
72.5	25.2	34.8	276	57	32	3	5	1
74.6	25.2	33.8	407	46.7	48.7	3.1	1.4	0.1
76.7	25.8	33.6	276	58	35	4	2	0
80	26.7	33.3	255	58	33	3	5	0
78.1	24.9	31.9	404	73	16	8	3	0
77.6	27.1	34.9	257	38	53	3	5	1
76.1	25.1	33	364	19	68.1	10.1	2.4	0.4
70.1	22.5	32.2	252	40	51	6	2	0

80.2	25.8	32.2	382	49	43	5	0	0
80	27.3	34.1	354	82	14	2	2	0
79.9	25.9	32.4	339	62.1	26.2	6.2	5.2	0.3
78.7	26.6	33.8	297	71.5	22.6	4.9	0.8	0.2
81.7	28.2	34.5	359	52.7	37.8	7.3	2.1	0.1
78.1	25.8	33	228	70.8	19.4	8.2	1.5	0.1
88.2	29.8	33.7	809	42.7	48.6	5	3.3	0.4
76.5	26.8	35.1	280	56.8	34.4	6.4	2.1	0.3
77.1	25.3	32.8	247	69.7	19.4	7.4	3.3	0.2
79.3	24.7	31.1	334	83	10	6	0	0
78.8	26.7	33.8	286	71	23	3	3	0
74.4	24.9	33.4	318	72	25	2	0	0
77.1	26.4	34.3	205	71	18	9	0	0
76.9	25.7	33.4	329	50	21	11	10	1
80.4	26.4	32.8	328	64	16.6	3.8	15.4	0.2
81.6	27.4	33.6	315	84	12.1	2.9	0.9	0.1
77	25.9	33.6	297	69.3	24.6	5.6	0.3	0.2
80.4	26.3	32.7	575	40.5	37.5	16	4.5	0
81.3	26.9	33.1	339	70	19	11	0	0
75.4	24.8	33	292	41.3	47	6.7	4.7	0.3
78.2	26.7	34.1	436	47.3	44.3	3.7	4.3	0.4
86	29.1	33.8	408	62.1	29.8	5	2.9	0.2
80.8	26.8	33.1	638	63	30	2	2	1
76.2	25.8	33.8	309	77	13	6	3	0
83.4	27.4	32.9	251	44.6	40.8	3.9	10.4	0.3
75.9	25.5	33.5	370	10	77	11	1	0
78.4	26.2	33.4	291	85	9	4	0	0
80.4	26.5	32.9	321	55	34	7	4	0
81.6	27.4	33.6	403	30	59	9	2	0
75.9	24.9	32.7	279	76.5	18	3.2	2.2	0.1
78.1	26.3	33.6	275	59	22	17	20	0
79.2	26.2	33.1	299	82.1	11.1	6.2	0.5	0.1
75.7	24.1	31.8	607	64.9	25.5	7.6	1.5	0.5
84.3	27.8	33	645	53	37	3	6.5	0
59	19.3	32.7	389	72.4	21.2	5.5	0.7	0.2
69.7	24.2	34.7	232	84	11	4.2	0.7	0.1
83.3	27.3	32.8	441	37	59	3	1	0
81	26.7	33	220	81.4	13.9	3.7	0.8	0.2
83.5	26.9	32.2	27.5	81.7	12.9	2.3	2.9	0.2
79.2	26.9	34	292	61.7	25.8	4.5	7.8	0.2
81.2	27.5	33.8	267	42.1	43.6	7.5	6	0.8
82.4	27.7	33.7	337	70.1	21.3	4.9	3.3	0.4
82.4	26.6	32.3	229	65.8	26.5	5.4	2.1	0.2
79.4	26.4	33.2	314	53	26	3	4	2
75.9	24.5	32.3	820	53.7	39.5	5.5	0.9	0.4
84.7	27.4	32.3	232	31.2	58.8	8.8	0.8	0.4

74.2	23.3	31.5	664	45.1	44.7	7.1	2.7	0.4
79.2	25.6	32.3	334	75.2	21.8	2.5	0.4	0.1
81.4	25.9	31.9	239	69.9	20.8	4.3	4.8	0.2
82	27.1	33	446	41.8	47.3	8.4	2.2	0.3
79.2	26.3	33.2	490	48	44.5	4.6	2.4	0.5
79.1	26.4	33.3	397	59.1	31.8	5.3	3.7	0.1
71.3	23.1	32.4	331	33.3	50.7	9.1	6.6	0.3
74.6	25.9	34.7	293	62.7	29.4	5.3	2.4	0.2
78.9	25.9	32.8	356	67.9	24.6	6.5	0.8	0.2
79.3	25.5	32.2	330	34.4	55.3	5.2	4.8	0.3
80	26	32.5	205	65.4	23.7	2.8	8	0.1
79.6	25.9	32.5	333	74.9	19.6	3.3	2	0.2
80.8	26.4	32.7	367	61.5	31.3	4.4	2.2	0.6
84.3	27.9	33.1	368	67.4	25.5	3.5	3.4	0.2
59.4	19.3	32.5	378	55.1	32.4	6.3	6	0.2
80.8	26	32.2	169	65	26	2.1	6.7	0.2
76	25.6	33.4	302	49.7	35.3	12.2	2.6	0.2
82	27.6	33.7	237	76.4	16.2	5.5	1.8	0.1
80	25.6	32	402	75.1	18.1	4.9	1.7	0.2
64.7	19.6	30.3	380	43	49	6	1	1
80.9	26.6	32.8	394	42.1	49.3	7.2	0.8	0.6
81.6	26.7	32.7	343	52	37	8.4	2.4	0.2
75.9	24.4	32.2	366	73.3	19.8	3.7	3.1	0.1
80.6	26.5	32.8	274	75.1	14.9	3.5	6.3	0.2
76.7	24.6	32.1	219	61.3	30.6	6.6	1.3	0.2
80	27	33.8	386	62.2	31.1	4.7	1.8	0.2
74.3	25.3	34.1	207	60.6	29.1	7.8	2.2	0.3
78	25.9	33.1	379	55.5	36.1	5.7	2.6	0.1
80	6.9	33.6	443	76.6	19.7	3	0.5	0.2
77.9	26.2	33.7	371	75.2	18.1	4.2	2.2	0.3
78.1	26.4	33.8	82	19	72.7	6.3	0.4	1.6
82.5	27.8	33.7	398	57.3	34.4	5.4	2.6	0.1
82.5	27.2	33	403	61.2	2.2	6.4	8	0.2
84.1	28.4	33.8	439	74.8	17.3	4.6	3.2	0.1
79.6	26.3	33	397	56.6	34.7	4.9	3.6	0.2
73	24.9	34.1	177	38.9	53.2	6.8	0.2	0.9
77.4	35	32.3	460	19	70	5	3	1
62.7	19.5	31.2	445	17.7	75	6.9	0.2	0.2
82.6	28.2	34.1	524	65.3	26	5.9	2.6	0.2
80.1	27	33.6	370	59.5	26.5	6.6	7.2	0.2
83	28.5	34.3	232	38.7	49.8	7.1	3.9	0.5
79	25.6	32.5	458	56	35.3	5.5	3	0.2
73.9	25.4	34.4	244	46.3	42	3.9	7.4	0.4
82.2	27.6	33.6	332	62.9	30.3	5.5	0.9	0.4
83.2	28.7	34.5	119	70	18	9	0	0
58	19	32.8	372	62	29	6	0	0

82	26.5	32.3	64	85	7	8	0	0
77	26.7	34.6	574	23	66	9	0	0
64.1	19.7	30.8	532	46.8	39.2	4.2	9.5	0.3
79.2	27.3	34.5	239	66	26	3	2	0
86.9	28.8	33.2	285	52.3	37.7	4.2	5.3	0.5
70.2	22.3	31.8	195	36	54	7	1	0
77.8	25.8	33.1	212	73.3	18	7.2	1.4	0.1
77.8	25.1	32.3	271	40.1	48	10.4	1.2	0.3
81.5	27.8	34.2	272	76.8	20.6	2.4	0.1	0.1
83	27.8	33.5	386	70	11	5	6	0
79.8	26.2	32.8	225	49.8	42.6	3.4	4	0.2
58.5	19.3	33.1	352	61	33	5	1	0
64.4	20	31	589	44	43	9	4	0
81.6	26.7	32.7	224	84	9	5.3	1.6	0.1
59.6	20.3	34	439	86	6	8	0	0
80.1	26.5	33.1	371	63	29	6	2	0
82.2	27.7	33.7	341	36.4	49.5	6.7	6.7	0.7
82.1	27.7	33.8	373	69	23	4	4	0
77.7	25.3	32.5	306	48.9	42.7	5.5	2.5	0.4
74.8	26.2	35.1	156	75	18	2	3	0
81.9	28.4	34.7	779	18.7	70.3	6.7	3.6	0.7
78	26.4	33.8	445	58	34	5	3	0
86.5	29.1	33.6	321	83.1	12.6	2.7	1	0
83.1	28	33.7	251	67	20	6	4	0
82.9	27.9	33.7	296	63.4	28.6	6	1.7	0.3
77.5	26.5	34.2	363	46	35	16	1	0
79.2	25.3	32	392	53	37	6	2	0
79.7	26.3	33.1	387	66	26	7	1	0
80	26.6	33.2	285	63.7	30.5	3.6	1.9	0.3
78.5	27.7	35.3	290	61	31	5	3	0
77.2	27.5	35.7	319	58.5	35.2	4.8	1.2	0.3
75.6	25.9	34.2	468	31.6	62.1	4.6	1.4	0.3
79.1	26.5	3.5	394	56	34	6	4	0
76.1	25.9	34	475	60	28	8	4	0
82.5	27.8	33.7	146	69.6	23	6.6	0.2	0.6
82	28.6	34.8	640	32	61	4	1	1
77.2	26.1	33.8	175	45	45	7	0	0
85.1	28.8	33.8	263	87.9	7.4	4.5	0.1	0.1
79.1	27	34.2	380	71	22	6	1	0
81.1	26.4	32.5	363	73.7	20.9	4.4	0.8	0.2
86.7	28.3	32.7	381	70.2	20.2	6.2	3.2	0.2
82.6	27.9	33.8	312	82	13	5	0	0
81.8	27.9	34.2	543	54	35.4	5.2	5	0.4
84.5	28.3	33.4	465	67	12	2.2	18.4	0.4
78.9	26.1	33	283	77	16.4	6.4	0.1	0.1
81.3	26.9	33.1	479	36.5	53.4	7.1	2.8	0.2

76.4	26.3	34.4	138	22	58	12	3	0
78.7	26.3	33.4	301	80	16	2	1	0
78.9	26.7	33.8	183	55.2	37	7.1	0.5	0.2
77.3	25.4	32.8	332	45	39	10	4	1
77.6	26.6	34.3	206	5	84	4	1	0
78.8	26.9	34.1	432	75.5	15.7	2.9	4.5	1.4
81.7	28.4	34.7	243	47	45	5	3	0
76.5	25	32.7	437	36.3	52.5	10	0.9	0.3
64.5	21.9	33.9	604	34	61	2.4	2.4	0.2
77	26.4	34.2	206	40.4	47.6	11.7	0.2	0.1
81.2	28.2	34.8	544	71.2	22.9	3.3	2.5	0.1
81	25.9	31.9	488	56.6	33	5.4	4.8	0.2
79.3	26	32.8	271	37.2	41.5	8.3	12.6	0.4
78.7	24.7	31.3	620	62.1	31.7	3.6	2.2	0.4
81.5	27.1	33.2	441	61.4	28.1	3	7.3	0.2
83.5	27.3	32.7	759	60	35.5	4	0	0
78.7	25.6	32.6	290	59.7	30.9	6.1	3.1	0.2
85.7	28.3	33	462	69.1	26.6	3.9	0.2	0.2
82.9	26.6	32	299	45	48	4	3	0
84	27.9	33.2	246	78.4	12.8	4.9	3.8	0.1
65	19.4	29.9	327	35	45	6	13	0
73.6	23.9	32.5	331	52.1	38.2	5.7	3.5	0.5
82.3	26	31.6	431	47.6	40.6	8.2	3.2	0.4
87.5	28.4	32.5	311	63.2	29.3	5.9	1.4	0.2
80.9	25.6	31.6	515	65.9	24.9	6.8	1.9	0.5
77.3	25.6	33.2	559	65.1	25.5	4	5.1	0.3
82.7	27.4	33.1	244	55	31	10	3	0
79.3	27.1	34.2	318	37	52	3	8	0
59.6	18.5	31.1	360	64.5	25.2	7.1	3	0.2
78.3	26.2	33.4	427	49.6	32	5.9	12.3	0.2
76.5	24.4	31.9	310	59	33	6	0	0
80.9	28	34.6	235	65.6	24.1	8.3	1.5	0.5
61	19.4	31.8	380	56	35	5	4	0
79.4	27.3	34.4	266	45	48	5	2	0
	27.7	34.5	126	43	46	7	1	0
	28.5	34.4	228	51	35	8	0	0
	26.3	33.1	329	60	37	3	0	0
	27	34.7	292	62	24	10	1	0
	19.6	32.5	141	43	40	6	1	0
	29.5	34.7	263	37	45	9	3	0
	26.1	32.8	325	24	51	14	3	0
	27.8	33.6	324	40	41	9	7	0
	28.3	33.8	406	87	9	3	1	0
	27.5	33.7	364	56	26	7	5	0
	26.4	34.4	388	68	27	4	0	0
	27.6	32.6	218	59	31	1	0	0

27.5	33.2	519	45	44	4	7	0
28.1	32.6	382	61	28	6	3	0
25.5	32.3	358	9	66	11	6	0
19.4	31.3	527	44	46	6	4	0
28	35.6	284	44	68	26	6	0
28.1	31.3	420	19	68	12	1	0
26.7	34.6	345	67	19	7	7	0
25.6	31.3	412	28	42	8	19	0
25.2	32.4	441	2	81	16	0	0
26.2	33.7	555	30	49	7	12	0
28.5	33.3	210	55	33	7	3	0
28.5	35	316	80	10	6	4	0
26.8	33.3	432	54	33	6	6	0
26.9	32.8	397	76	18	3	2	0
26.9	33.5	422	41	43	8	4	0
27.5	33.7	508	80	16	2	0	0
26.7	34.2	229	34	45	8	1	0
25.7	33.7	745	28	66	5	1	0
27.6	31.4	349	73	19	3	1	0
20.1	30.2	382	38	50	5	6	0
27	33.1	531	39	44	3	8	0
28.9	33.8	556	69	22	5	2	0
26.1	32.9	761	48	43	3	3	0
26.9	32.2	345	64	22	9	2	0
23.8	31.4	430	53	30	10	5	0
26.4	32.9	534	32	49	10	8	0
27.1	33.1	402	52	25	2	13	0
25.9	32	431	27	58	10	5	0
27.5	32.6	294	62	28	5	1	0
26	32.5	233	41	48	5	2	0
27.8	32.2	736	26	59	7	6	0
24.3	31.5	281	39	43	12	5	0
18.8	31	471	30	58	10	2	0
26.7	32	352	29	48	10	6	0
27.6	31	441	79	13	4	1	0
24	31.4	511	73	18	7	2	0
29.6	32.3	369	49	36	2	6	0
25.9	32.6	459	39	55	2	4	0
22.3	31.6	630	12	75	8	2	0
18.2	30.7	481	17	74	4	4	0
25.1	34	371	65	22	6	1	0
27.1	34.9	365	54	35	7	3	1
26.7	34.9	377	70	21	4	5	0
25.8	34.5	511	47	50	3	0	0
28.8	32.9	336	69	24	3	4	0
28.9	33.6	363	66	27	4	0	0

24.2	32.4	342	51	41	6	0	0
17	29.4	434	26	56	10	7	0
25.9	32.5	433	29	68	2	1	0
28.1	34.3	582	70	22	7	1	0
25.4	31.6	197	57	29	5	2	0
25	31.4	430	7	76	13	4	0
28.9	36.8	295	50	37	10	2	0
26.3	33.8	381	10	70	13	4	0
28.9	32.2	356	47	48	3	0	0
24.5	32.4	361	56	36	1	2	0
26.5	35	555	73	19	5	0	0
28.1	34.1	762	49	31	9	3	0
28.6	33.8	477	50	36	6	7	0
27.6	33.8	298	56	32	4	5	0
25	33.4	445	42	46	7	5	0
26.1	34	319	69	20	7	2	0
28.5	33.9	238	58	27	4	0	0
26.2	34	465	44	48	2	5	0
25.3	33.5	560	43	45	10	1	0
27.4	33.2	535	73	16	7	0	0
28.3	33	397	67	26	5	0	0
20.2	30.7	589	39	55	3	3	0
18.3	32.8	666	25	61	6	4	0
26.4	34.7	569	30.8	53.9	10.2	4.6	0.5
29.3	34.5	251	84	10	4	1	0
22.7	33.2	296	61	24	4	3	0
26.9	32.3	394	41	46	6	1	0
20.3	32.6	442	50	36	8	5	0
27	33	379	45	46	7	1	0
24.7	33.5	285	44	46	6	2	0
26.1	33.8	364	74	4	4	3	0
26.3	34.1	358	80	12	4	0	0
26.7	33.1	518	52	41	2	2	0
26.3	32.2	263	75.8	19.7	3.8	0.6	0.1
25.9	34	585	10	66	14	9	0
26	34.1	495	73	20.5	1	2.5	0
24.8	33.8	280	49	39	8	4	0
24.3	32.8	432	33	59	7	1	0
25.1	33	264	57	35	6	0	0
27.2	36	541	28	61	8	1	0
26.3	32	256	70.4	19.8	6.9	2.7	0.2
27.3	34.3	378	76	17	2	2	0
25.5	35.1	426	48	3	3	7	2
27.5	32.7	235	61.4	26.9	10.1	1.2	0.4
27	32.4	300	68.9	14	14.7	2.1	0.3
25	32	282		40.7	6.1	2.6	

29.5	33.4	333	25.5	12.6	1.1
25.9	32.6	200	29.4	5	0.4
26.6	32.9	281	16.5	10.2	1.1
17.3	31.3	397	16.3	15.7	0.1
27.1	32.7	351	50.9	9.2	0
17.9	31.2	416	13.4	7.1	1.3
25.8	31.4	498	27.9	8.6	3.6
21.8	31.8	483	19	3.4	3.9
26.2	31.7	448	6.7	5.2	0.2
26.7	32.6	169	8.3	9.5	0.1
25.2	32.1	321	35.9	5.8	8.1
27.4	31.7	435	27.3	9.4	0.5
28	33.7	392	12.6	2.1	0.6
23.8	30.9	378	10.6	6	4.3
26.5	33	500	26.1	6.8	3.1
26	32.2	526	34.3	6.1	5.7
26.8	33.1	236	44.7	14.3	3.9
25.9	32.3	206	39.3	11.8	0.2
27.7	32.9	398	25.5	7.7	7.7
28.2	33.9	426	37.8	9.1	1.8
27.3	33.6	211	52.7	11.9	0.2
18.4	30.2	523	20.8	10.8	6.6
26.1	32.7	301	30.6	19.9	1.3
20	31.6	353	31.4	1.9	1.6
26.9	33.1	485	50.9	9.9	8.3
24.1	32.7	376	34.8	11.6	2.3
25.9	33.6	445	15.1	5.6	3.7
21.1	31.3	871	37.2	7	0.2
27.6	35.4	369	35.3	5.3	3.1
25.6	32.5	413	12.3	5.3	8.7
26.4	33.5	448	14.8	6.7	3.1
26.3	32.4	282	34	5.6	1.3
26.1	33.4	323	29.2	7.4	3.5
26.9	33.4	306	24.4	3	0.1
27.9	34.1	350	11.9	3.7	4
26.7	32.6	323	30.1	8.4	3.5
26.2	32	469	14.2	7.5	1
26.1	32.1	466	6.6	5.9	1.9
20.1	31.4	278	6.7	7.6	0.1
26	33.2	500	25	4.6	2.2
19.7	31.3	525	25.6	7.5	3.5
27.5	33.5	395	11.6	3.9	1.3
26.5	33	333	9.8	3.6	1.3
24.3	31.8	338	38.6	12.2	3.5
27.5	33.5	269	16.3	5.1	0.3
23.8	29.9	509	58.5	9.3	3

27.4	31.3	306	29.6	9.9	0.5
27.2	34.9	327	21.8	12.3	4.4
25.7	31.3	181	61	16.1	0.1
27.3	32.9	200	28.5	10.3	2.3
26.8	32.4	449	48.6	11.3	3.7
25.7	32.5	258	19.2	5	3.9
20	30.8	521	30.3	6.5	2
25.6	34.2	173	15.6	6	2.3
28.1	32.6	216	23.3	6.9	7.7
25.3	31.5	364	20.6	8.9	2.3
26.5	33.5	279	50.3	10.7	0
27	33.6	188	20	6.7	3.2
24.6	30.8	479	38.4	7.9	3.2
25.7	31.7	434	58.4	10.5	0.1
23.2	32.1	183	24.5	5.5	1.4
26.3	31.5	269	32.7	8	1.2
24.1	30.7	608	34.3	4.8	1.7
26.3	32.1	388	26.6	5.6	1
22.3	30.3	442	51.6	13.6	0
26.1	33	180	62.6	8.7	1.8
26.1	33.9	231	12.6	5.1	1.1
26	32.1	240	14.6	11.7	17.9
26.1	32.6	173	84.6	3.9	0.8
24.3	31.3	201	42.5	11.2	3.3
26.3	33.2	359	16.5	5.1	2.1
27.7	33.6	683	39.2	10.3	0.3
26.8	31.9	332	41.9	10.3	9.8
26.3	32.7	215	28.9	14	0.1
24.6	32.6	156	64.5	11	2.6
25.8	32.6	187	41.1	9.8	0
24.1	31.3	260	34.5	18.5	0.5
26.3	32.2	284	31.8	12.6	4.6
27.3	33	435	57.4	9.7	4.5
23.1	31.1	335	33.6	7	1.7
20.1	29.5	328	50.7	8.6	2
23.5	30.2	449	18.7	2.5	3.1
24.2	31.8	354	29	6.2	0.2
26.9	33.7	354	42.3	11.2	2.2
24.9	32.6	659	41.3	4.8	2.4
27.1	33.6	416	13.4	6.5	0
26.1	32.2	340	3.3	1.9	0.1
29.3	33.6	376	18.8	3.9	1.4
23.9	32.2	377	12.8	11.9	0
24	32.1	442	23.9	9.3	6.3
25.8	31.6	258	26.9	5.4	4.9
25.4	31.8	319	24	4.9	0.6

26	33	538	44.8	10.6	0.5
25.1	32.1	377	22.5	6.5	5.3
26.7	33.3	280	13.6	6.3	1.7
26.5	32.2	235	15.1	9.4	11.6
26.4	32.9	360	22.1	6.8	5.2
24	31.3	481	27.7	3.3	0.6
22.1	31.3	451	31.5	8.7	7.1
27.4	31.7	308	32.7	9.6	5.3
24.7	31.5	333	14.4	7	2.2
26.8	35.1	318	75.2	11.5	0.4
26.7	31.2	212	66.4	8.2	0.8
27.2	33.2	318	12.8	6.4	1.2
22.2	31.1	534	61.3	17.1	2.7
26.4	32.2	192	17.1	14.7	0.1
26.9	34	307	19.6	7.9	0.1
25.5	31.4	161	39.8	14.9	0
27.9	33.6	539	35.4	9.6	5
23	30.3	346	30.8	10.7	0.9
25.8	31.8	244	29.8	7.1	1.4
27	32.1	479	17.8	3.4	0.4
26.8	32.1	438	22.3	7	0.5
20	31.7	620	32.2	7	4.2
29.9	33	263	18.4	2.4	3
26.1	31.7	247	17.1	6	0.2
26.4	33	322	29.8	4.3	5.3
27.1	33.7	262	7.4	8.8	0.1
25.8	33	546	31.1	7.8	5.6
23.2	30.8	275	17.5	10.2	0.4
24	31.9	558	26	7.7	5.7
26.6	31.7	445	24.7	3	4
23.5	30.4	192	9.3	2.3	2
27.6	33.4	228	9.9	8.6	1.1
26.5	31.9	465	33	8.9	5.1
27	32.4	351	41.4	5.5	2.4
24.4	31.5	488	14.2	4.3	0.1
27.5	32.8	356	30.4	4.8	5.8
29.4	33.8	157	4.6	4.4	0.2
25.4	31.7	283	26.9	10.2	0.5
21.8	29.8	204	38.6	9	2
25.4	30	846	24.6	12.7	0.7
26	33.1	217	22.1	10.2	0.8
25.1	30.5	285	74.4	8.6	3.5
27.6	32.8	534	30.5	9.5	1
27	30.6	260	31.8	4.1	2.3
29.7	36	153	3.3	2.2	0.7
24.1	32.4	458	15.1	2.6	0.2

25.4	30.9	244	38.9	10.5	0.2
18.8	31.2	511	12.5	13.3	1.4
24.8	32.7	229	53.8	5.5	0
25.2	32.9	391	23.8	17.5	1.3
25.9	31.4	339	17.3	12.7	2.3
25.6	33.5	341	19.2	5.5	0.3
22.4	29.7	269	38.2	5	0
23.6	31.9	646	41.7	12.1	6.2
25.8	32	419	23.8	5.2	2.4
27	32.9	188	25.8	6.5	3.6
25.6	32.9	385	45.2	7.2	1.8
27.1	31.5	376	20.8	6.9	0
26.4	32.7	257	43.6	5.7	0
26.2	32.3	703	18.8	6.9	1.9
24.5	31.7	146	25.6	14.5	2.3
26.5	32.8	475	16.8	5.1	0.1
26.9	33	357	20.2	5.7	0.3
24.7	32.3	477	36.7	4.2	2.1
16.4	29.3	544	26.4	2.6	1.9
25.8	32.7	338	43.7	5.3	2
28.2	34	366	11.7	6.8	0.9
27.3	32.9	737	46.7	5.5	2.3
27.5	32	200	27.4	5.8	7.4
24.2	31.9	551	22.1	11.5	0.6
26.4	32.6	479	21.9	2.9	0.6
24.6	31.2	418	27.9	14	7.5
18.5	32	203	16.4	6	0.6
25.9	31.8	476	21.4	9.8	1.7
22.7	31.6	348	51.6	18.3	0.1
27.4	33.7	446	12.8	4	0
27.9	33.5	380	43.8	15.6	1.8

CRP (mg/l	AST (U/L)	ALT (U/L)
45.1	276	150
9.8	41	38
98	39	21
0.4	40	14
6.8	118	102
21	30	12
34.2	28	13
23.2	32	14
3.8	31	13
10.3	44	17
136.8	28	28
184.3	38	20
53.6	66	71
26	29	12
114.7	59	50
149.6	33	21
14.1	33	22
1.1	32	17
0.9	37	17
15.1	36	15
77.6	37	16
50.3	40	16
10.6	31	19
3.4	40	9
140.1	26	11
4.4	49	16
115	28	12
2.7	35	11
51.2	32	17
44.3	47	38
19.4	32	17
35.7	36	20
76.6	32	17
6.4	32	19
123.8	26	18
7.2	36	14
9.3	50	23
198.9	34	21
10.5	49	23
40.2	32	15
10.2	34	15
77.9	36	17
4	36	13
216.8	37	22
66	28	18

18	37	11
13	36	14
111.1	30	13
8.7	39	19
15.8	24	10
50	31	15
9.9	37	16
297.6	32	17
3.4	37	40
4	29	14
46.6	32	17
33.7	37	50
14.9	46	17
7.8	42	17
30.4	25	15
260.1	70	33
69.1	28	17
71.3	15	9
4.9	34	16
0.7	45	22
14.5	48	16
0.2	258	388
32	25	15
61.5	32	17
20.6	29	13
12.7	77	54
24.4	29	14
49.1	32	15
16.6	34	15
2.2	32	17
41.4	54	40
164	28	15
1.1	52	19
20	30	14
29.5	70	44
5.2	22	15
122.8	35	17
11.8	48	17
149	40	19
11.5	32	17
1.1	40	16
7.5	37	18
28.5	32	17
5.3	34	13
30.7	40	10
0.3	36	23

1.3	31	11
11.4	36	22
139.5	38	15
57.8	32	17
27	30	14
7.8	41	16
68.3	33	14
3.6	55	25
234.1	20	7
4	63	17
22.9	47	22
0.6	28	12
5.2	80	46
25.1	41	22
18.6	35	42
2.6	33	16
72.4	43	17
195.9	28	14
2.3	47	21
40.4	32	17
11.9	29	16
12.7	36	14
3.1	39	18
103.8	34	17
121.3	40	17
73.6	35	18
132.5	36	15
0.7	32	17
1.3	47	23
75	36	17
133.4	32	17
44	32	17
85.6	52	15
74.8	63	34
2.3	45	18
1.9	35	18
11.5	29	14
15.1	32	17
53.6	30	15
0.19	36	17
93.6	28	13
0.8	43	14
5.1	166	204
176.6	26	17
1.4	32	17
39.7	32	17

34.4	34	15
13.4	53	29
26.7	32	16
2.7	39	16
23.6	32	14
2.9	137	123
3.1	58	21
3.1	36	21
79.4	38	20
9.4	32	17
102.6	38	17
6.9	54	29
43.6	35	14
14.2	31	16
9.5	32	17
18.2	36	21
225.3	23	19
44.2	44	26
104.6	41	25
55	17	15
6.3	42	35
4	63	20
41.9	39	16
6	32	17
12.5	32	17
52.9	31	14
0.19	40	18
0.9	31	18
35.6	65	52
60.2	26	13
50.5	32	17
117.3	65	31
96.4	30	22
0.19	62	17
7.7	32	17
1.6	45	27
121.3	45	70
145.7	27	15
9.4	32	19
1.5	36	14
0.5	35	16
57.8	32	17
157.4	32	17
62.4	34	20
121.9	56	27
17.5	34	23

11.4	44	13
108.7	36	17
6.6	45	24
16.4	28	9
0.2	32	17
26.7	40	19
110.5	33	20
42.4	35	17
48.1	34	15
57	33	13
59.4	30	14
4.5	38	17
97.5	35	17
21.5	52	18
68.6	29	9
0.2	62	49
5.2	37	20
1.6	32	17
126.9	52	43
5	42	22
1.3	43	23
9.5	41	18
0.2	38	20
23.5	34	17
8.5	36	23
19	30	24
2.8	47	24
39.6	66	30
59.6	39	19
315.4	47	34
6.6	36	14
139.9	32	14
64.4	36	17
38.4	45	24
21	32	17
10.2	37	22
5.1	45	20
50.7	23	12
73.7	36	20
0.2	32	17
8.1	38	15
3.8	43	43
15.4	30	10
0.6	32	17
0.5	44	15
0.4	25	18

2.4	34	16
0.6	74	49
0.2	27	19
17.2	43	22
1.8	32	17
0.9	41	24
107.8	31	13
8.4	24	21
2	26	13
22.5	45	40
2.7	38	22
0.3	85	40
141	53	29
280.8	46	31
4.7	58	30
39	32	16
1.2	42	18
4.3	33	17
142	42	20
114.2	44	11
0.2	61	38
60.1	36	40
5.7	32	17
0.19	35	14
32.1	37	26
1.8	40	37
62.8	53	19
8	36	22
1.3	62	17
1.6	67	10
0.19	47	20
126.3	34	14
61.4	35	17
58	48	21
8.2	35	21
30.2	38	17
51.1	32	17
0.19	31	19
45	30	21
11.3	28	12
14.6	32	17
0.19	34	16
7.6	32	17
66.8	39	15
12.2	54	36
0.9	41	25

16.1	32	17
5.1	34	28
8.8	27	8
1.8	44	14
0.19	37	22
3.7	32	17
4.5	39	20
23.7	32	17
21.7	34	16
106.5	32	17
13.8	28	19
0.7	50	42
20.1	31	17
20.1	32	17
41.3	37	10
16.8	25	11
3.1	39	27
1.2	32	17
2.6	45	37
0.19	34	28
122.8	43	20
63	39	20
56.4	32	17
1.3	36	22
12.4	31	20
3.5	36	18
162.3	30	11
0.6	50	44
26.5	50	19
151.7	26	12
54.5	42	26
7.7	20	10
2.9	55	31
60.1	33	18
10	32	21
102.5	67	15
127.3	42	23
28.6	30	15
15.1	29	12
79.9	32	17
8.3	322	172
57.2	37	18
1.7	50	17
34.6	28	13
25.3	25	19
112.8	35	12

67.8	32	17
0.19	28	22
26.9	31	41
6.6	32	17
6	27	10
18.6	32	17
7.6	32	17
2.6	32	17
21.9	28	12
3.2	39	23
30.7	42	22
143	32	17
6.7	38	27
13.5	33	14
1.7	42	37
0.2	48	24
11.7	48	26
272.8	32	24
63.3	32	17
49.7	43	17
0.6	42	18
36.5	17	12
36.6	32	15
21.6	45	18
9.3	35	18
77.9	43	21
4.4	39	25
17	28	14
51.3	34	14
0.19	32	17
0.8	39	18
0.19	35	17
70	32	15
5.9	35	30
74.4	39	21
35.5	32	17
19.4	32	17
79.3	24	13
2.9	36	18
44.1	35	12
37.8	35	22
131	33	16
93.4	20	15
6.7	32	17
0.2	32	9
17.1	25	15

27.8	32	13
7.2	43	18
270.4	38	14
157	37	20
27.5	32	17
27.9	39	15
9.4	30	17
41.2	33	19
13.1	34	14
0.19	33	18
46.8	29	12
102.1	32	17
3.8	35	18
0.19	34	16
3.1	28	19
178.1	29	13
45.3	62	36
10.1	27	26
0.2	29	19
4.9	26	13
76.4	55	34
50.8	43	25
0.9	32	21
11.5	45	17
19	37	15
6.1	47	34
10.8	32	17
153.8	32	17
0.3	31	19
3.3	32	16
0.5	46	22
1.1	61	21
8.2	42	16
11.9	34	19
12.1	44	21
28.7	41	26
15	38	18
23.7	28	23
0.19	39	21
50.4	32	17
10.6	30	18
12.1	30	13
109.5	30	19
110.6	86	103
153.7	30	13
4.4	46	20

17.4	32	17
0.5	40	26
15.1	33	21
53	67	36
189.2	30	13
0.2	74	58
4.6	53	72
64.4	32	17
11.9	34	18
7	141	62
166.1	37	18
3.8	52	35
32.8	32	17
60.6	74	43
31.9	32	13
28.1	35	14
13.4	39	21
6.3	36	14
37.1	24	13
16.5	36	18
105.1	40	27
22.8	1489	963
4.1	34	18
17.7	32	17
22.5	56	54
1.9	44	52
115.7	32	17
8.4	34	14
4	34	17
101.2	44	26
1.8	30	19
1.2	32	26
51.8	36	14
103.7	28	13
8.3	32	17
23.9	33	21
0.2	37	26
4	29	24
4.2	32	17
0.19	36	18
7.4	30	22
178.5	31	15
113.4	30	17
12.9	50	17
2.5	35	19
4	41	16

35.5	22	17
51.9	51	25
21	37	28
185.3	26	15
4.7	39	20
112.8	36	24
1.5	35	16
11.7	31	12
102.8	31	11
113	27	15
68.8	33	22
18.7	36	31
6.4	35	19
53	32	17
12.3	32	24
34.6	45	42
29.9	33	16
75.5	47	27
27.3	34	12
6.1	45	15
6.3	32	13
110	41	29
8.9	29	26
7.5	54	20
0.2	27	17
204.7	27	102
6.1	29	20
0.3	43	16
76.3	39	18
1.3	733	805
0.19	34	30
68.9	19	11
16	37	23
1.4	34	13
1.3	40	15
50.1	27	17
30.7	35	14
75.3	32	17
74.1	39	18
2.1	42	16
68.5	21	11
12.1	31	11
5.3	35	15
2.1	32	17
0.4	51	30
4.4	69	81

3.3	32	17
3.7	77	24
0.19	32	17
4.5	56	20
1.3	58	23
229.4	31	21
0.3	33	16
4.8	48	23
8.8	129	352
28.5	33	18
3.2	35	15
5.6	37	22
4.3	31	15
12.6	46	17
16.5	32	17
72.1	54	38
35	40	29
79.3	32	17
29.9	38	16
2.7	37	17
122.3	42	20
36.6	27	13
0.19	36	15
13	45	33
0.6	32	17
9.4	32	19
0.9	32	16
3.3	34	11
65	36	17
134.6	28	13
28.4	34	17
3	55	31
0.19	39	21
0.9	34	20
15.1	32	17
0.19	32	17
14.6	37	18
6.5	37	20
0.9	40	17
46.6	44	17
105.2	30	15
21.1	79	319
11	31	13
78.8	32	16
122.1	46	33
122.5	28	16

0.19	30	20
52.8	59	29
6.7	28	16
0.9	35	21
52.4	36	25
10	34	27
25.6	32	17
18.1	41	24
18.4	33	14
13.2	62	47
8.5	35	16
24.1	28	13
30.2	49	14
0.3	37	16
3.3	70	32
18.6	32	17
9.1	42	14
26.9	44	17
15.5	39	14
16.3	32	17
4.1	34	14
5.4	34	15
8.7	63	34
35.3	31	11
5.4	42	15
45	29	14
20.8	33	19
0.7	24	18
0.4	44	18
45.5	32	17
0.19	41	19
0.19	37	17
4.6	38	31
4.1	48	33
3.4	36	17
4.3	39	18
7.7	42	34
27.9	32	17
12.7	41	29
12.7	45	19
5.7	32	17
2.7	38	25
9.8	38	15
129.5	30	15
12.6	34	15
36.4	38	21

23.6	40	12
6.2	32	17
5.1	31	16
159.5	44	36
20.7	26	13
72.4	32	17
5	31	17
1.9	53	21
11.2	32	17
7.5	40	21
92.8	51	20
0.7	59	23
1.5	34	14
118	31	14
77.2	42	35
15.4	32	17
1.5	32	17
8.2	60	19
4.4	32	17
12.7	32	19
7.2	52	34
47	32	17
4.2	71	83
30.3	37	18
75.5	32	17
15.1	32	17
0.19	28	13
0.19	32	24
20.6	36	16
9.8	32	17
22.8	34	20
6	46	19
13.1	32	17
2.5	34	19
18.5	35	17
19.9	28	16
38.2	28	12
120.2	32	17
4.3	32	17
23.6	45	27
39.7	39	17
25.4	31	15
0.9	30	15
131.1	25	8
27.2	28	12
0.4	37	11

0.19	68	86
60.3	27	17
35.6	31	15
21.2	32	15
4.4	52	24
0.5	36	17
74.5	57	26
3.2	32	18
0.8	43	16
10.4	37	24
201.7	32	17
10	40	16
33	32	17
1	59	16
3.5	42	21
2.9	42	16
2.4	31	18
61.1	34	14
42.8	38	18
135	30	18
15.3	32	17
92.2	30	12
28.4	30	15
171.3	26	16
1	37	21
2	33	20
6	39	20
13.5	39	20
35.9	28	11
153	32	17
1.4	39	17
5.6	51	18
12.4	32	17
25.8	41	13
0.7	55	19
34.9	49	20
2.4	67	17
20.8	32	17
8.7	32	17
28.5	32	20
58.6	32	17
29.7	32	17
15.1	32	21
0.5	57	21
10.8	40	27
0.19	32	18

3.2	111	102
0.9	43	13
74.7	40	10
12	33	16
68.2	53	38
0.9	40	11
249.4	25	14
21.4	64	15
120.4	30	19
11.4	39	24
7.2	28	16
0.8	40	20
0.2	32	17
4.3	47	23
1.8	54	36
0.19	41	16
25.4	37	22
3.9	143	125
209.7	21	14
19	36	20
13.6	38	19
69	42	30
38.4	43	16
30.3	32	17
3.3	42	20
11.4	32	17
0.19	38	28
49.3	45	33
0.7	41	20
0.19	40	14
1.6	32	17
3.6	53	23
12.3	32	17
44.9	44	14
38.3	60	44
34.4	43	32
29.2	32	20
0.5	49	24
0.6	48	14
21.3	48	25
9	35	17
0.7	69	39
0.4	55	33
0.4	146	51
34.3	34	24
11	32	17

20.1	35	18
5.7	44	14
16	29	16
1.9	42	21
10.2	32	17
2.6	28	16
1.9	30	21
11.1	32	17
16	32	12
24.3	41	17
3	33	20
1.7	47	20
42.5	32	17
0.19	40	24
0.8	32	17
15.5	32	17
3.9	52	26
42.7	31	11
23.1	28	13
4.1	42	19
5.7	40	20
0.6	47	31
12.8	44	23
0.6	54	29
6.2	32	17
0.19	38	23
2.9	30	17
2	29	19
0.5	32	17
22	28	16
8.4	37	24
35.3	32	17
0.4	47	27
1.2	48	37
47.2	31	13
32.8	42	25
9.9	34	15
1.2	39	19
9.1	31	19
22.6	33	24
0.19	43	16
29	42	24
54.6	41	19
12.6	38	18
14.2	41	17
18.3	55	34

0.4	31	18
2.6	159	132
9	53	18
9.3	34	15
97.2	32	17
5.9	36	16
118.4	19	14
19.3	26	15
0.4	56	54
8.9	43	27
52.6	43	13
1	28	20
0.19	48	22
1.9	46	16
0.19	23	11
0.19	46	27
11.2	32	17
33.5	43	16
49.3	34	18
12.1	50	18
44.1	39	29
0.5	33	17
14.1	41	15
41.6	37	19
20.9	36	20
44.8	33	12
0.6	38	22
0.19	46	37
10.2	25	16
11.5	38	30
385	32	17
15.5	50	17
27.3	36	21
1.9	38	18
0.3	32	8
15.4	32	17
2.7	46	20
11.3	50	29
5.1	30	13
5.9	50	19
0.19	33	9
0.3	46	32
14.1	54	28
0.4	31	17
0.6	74	116
8.8	38	21

24.1	51	41
12.2	32	17
49.8	26	11
75.6	37	19
1.9	40	21
6.1	57	28
0.6	84	76
46.6	25	20
0.4	32	17
32.2	50	26
20.2	44	16
0.19	36	23
0.19	42	19
4.3	24	12
0.19	37	28
0.8	41	14
18	32	17
77.1	32	17
2.2	57	20
0.9	32	17
0.2	35	14
9	32	17
1.8	32	17
74.6	30	23
2.8	76	30
18.5	32	17
0.9	45	30
7.3	41	17
1.3	39	22
4.5	33	14
7.3	51	30
4	42	22
39.6	26	23
26.6	37	12
74.9	25	14
16	32	17
0.19	28	14
0.8	56	54
23.2	32	19
1	51	25
4.5	37	18
2.9	35	17
59.3	32	17
3.9	32	17
71.6	29	13
4.6	26	13

0.19	29	17
7.5	32	17
21	44	20
2.1	51	33
5.6	32	28
1	32	17
8.4	48	29
0.3	50	50
0.2	61	29
57.3	25	14
68.7	25	21
1.9	40	29
25.1	37	19
3.1	42	22
31.6	21	14
0.3	42	21
49.8	51	28
46.4	32	22
0.19	28	16
9	57	20
4.5	32	17
0.5	27	10
3.4	32	17
0.19	21	11
29.1	32	17
2.4	28	30
0.4	45	19
0.4	32	17
5.4	33	22
2.7	52	24
1.3	43	33
4.6	37	20
9.5	37	16
1.9	67	39
9.6	48	24
2	33	15
0.6	50	19
0.19	50	16
77.1	24	12
3.5	44	14
5.9	43	19
3.9	32	17
2.4	35	16
0.9	56	19
1.5	51	56
0.6	24	13

9.8	32	17
12.3	26	21
54.8	39	31
0.2	52	32
1.8	32	17
5.4	34	15
1.8	40	28
44.4	36	19
16.5	35	25
0.19	27	15
0.8	336	630
12.4	23	16
0.3	32	17
0.4	32	17
0.19	46	29
9.7	20	17
14.8	45	15
2	32	17
20.5	32	17
12	44	25
2.8	59	20
5.7	56	46
4.5	45	24
20.1	39	28
3	59	19
15.2	32	17
7.4	32	17
45.3	299	245
1.8	48	26
14.1	32	17
180.1	26	13
16.1	40	20
134.4	46	25
131.5	25	22
9.2	32	17
20.4	39	21
79.2	37	24
64.6	39	23
19.9	30	16
0.2	53	24
6.6	24	11
1.8	60	38
0.7	54	66
11.3	34	16
0.19	42	32
0.19	42	24

50.1	26	27
9.4	32	17
9	43	24
258.3	24	13
0.8	32	17
2.3	47	40
161.5	36	20
0.4	32	17
102	40	14
0.7	37	16
163.2	22	17
38.7	40	17
17.5	30	15
0.3	49	29
6.1	32	17
71.5	20	13
135.5	22	10
20	24	16
109.6	28	13
8.9	32	16
58.7	336	835
54.6	24	16
123.2	28	12
112.2	22	13
0.4	23	15
70.6	32	17
1.9	22	12
39.2	38	17
140.3	38	34
356.3	25	21
20.4	37	13
27.2	31	11
13.2	38	16
153.3	28	17
15.7	32	17
103	24	14
42.3	16	9
33.2	20	11
61.9	32	17
6.8	42	19
14.8	36	17
259.4	24	16
26.4	32	13
77	37	15
8.2	40	13
172.3	37	11

273.1	30	17
142.5	36	13
72.8	27	13
29.6	43	13
5.5	32	17
42.8	24	7
3.6	30	10
63	26	15
103.2	25	10
9.1	65	17
15.9	27	11
7.5	30	15
30.7	44	18
261.4	22	13
55.8	31	11
24.3	31	30
10.7	32	17
8.9	45	50
0.3	30	21
94.8	28	18
3.2	32	13
0.5	24	20
33.6	22	12
1.8	36	17
0.4	26	12
18.7	35	15
107.7	37	16
0.2	42	18
55.9	34	20
73.6	43	26
25.6	32	10
70.5	25	13
95	261	63
3.2	28	12
17.3	30	14
116	27	13
3.7	31	12
110.5	48	15
4.4	27	18
71	28	21
49.7	42	23
151.6	32	13
157.3	35	15
227.4	29	18
8.1	30	8
115.9	43	17

147.8	38	16
36.2	27	8
54.4	29	18
27.1	33	10
31.5	36	23
19.7	32	17
5.3	33	24
2.4	34	12
97.2	27	13
71.5	27	11
4.6	33	13
20.5	29	14
2.9	46	21
6.8	35	14
67.6	35	14
9.2	43	18
8	37	17
25	40	42
41.1	29	15
31.1	30	16
1.6	32	16
33.5	27	13
61.1	36	10
4.1	36	20
183.2	32	17
24.6	32	17
48.1	40	16
56.2	28	14
107.4	45	35
2.7	32	7
6	27	14
104.8	25	12
4.1	25	14
108.5	29	18
147.3	20	10
53.8	30	13
31.7	38	9
165.9	22	14
6.4	41	19
39.2	28	16
77.8	28	14
8.8	48	14
35.3	75	29
16.4	42	31
1.6	99	74
18.9	28	16

7.9	33	10
71.1	37	20
139.5	20	10
38	32	17
2	29	19
5.9	44	19
26	35	15
6.9	32	17
46.1	35	13
226.5	49	17
0.7	31	20
26	46	32
27.7	25	10
67.2	27	12
40.1	33	22
66.2	27	15
6.9	28	16
22.5	32	17
100.7	55	17
0.19	32	17
23.9	33	19
71.2	39	19
11.1	30	17
78	24	15
8.3	39	16
18.9	36	17
35.4	35	12
2.4	30	13
41.3	22	10
121.4	26	18
52.1	35	28
51.3	34	18
54.7	40	16
1.1	37	22
117.1	32	18
107.5	32	13
2.3	35	15
20.6	32	17
6.2	30	17
52.2	30	20
4.6	33	15
0.2	36	16
13.7	41	20
9.1	40	19
15.1	32	17
6.1	35	16

94.1	32	16
42.5	23	13
0.19	48	26
16.4	34	17
35.9	33	11
53	34	17
5.3	28	14
11.8	57	19
9.1	38	17
172.1	34	14
27.6	36	18
19.7	29	12
6.1	30	12
5.5	49	13
4.5	39	25
19	44	11
144.7	36	14
115.7	28	9
22	32	17
6.7	35	16
43.6	35	23
159.4	23	13
309	32	13
22.3	32	14
39.2	32	17
184.2	33	15
11.7	32	16
18.6	29	12
0.19	26	12
57.9	40	30
71.4	23	13
79.6	27	10
73.5	34	17
38.3	375	121
15.6	39	17
2.2	38	17
36.8	32	17
8.3	32	17
112.5	36	15
95.2	26	17
72.5	40	28
9.4	30	13
0.5	39	15
16.1	46	22
0.19	48	22
61.8	29	14

18.6	33	10
14.2	26	16
136.2	32	17
2.3	48	40
14.4	46	35
28.9	39	18
0.19	37	13
98.7	48	24
4.1	32	6
61.4	36	17
26.2	33	18
74	32	17
32.1	30	14
103.3	35	10
43.1	43	18
2.9	40	17
55.1	31	17
34.9	34	17
56.4	42	21
8.6	39	17
41.8	31	14
151.5	33	12
11.5	55	175
161.6	28	17
0.3	50	17
147.8	34	20
75	52	29
9.7	46	29
33.3	32	17
2.8	38	17
93.9	20	11
21.5	26	11
28.8	45	42
74.6	30	16
0.4	57	19
5.3	54	16
222.1	28	11
9.7	51	18
33.6	43	18
160.9	25	29
2.4	32	17
10.3	37	22
9.1	26	13
37	32	11
23.1	34	10
10.3	47	24

93	47	17
3.8	34	17
4.6	41	24
224.6	30	16
1.6	35	16
13.6	32	17
30.9	38	17
7.7	40	15
31.7	45	35
0.2	35	19
19.1	40	15
202.2	29	17
70.8	39	21
27.8	36	18
72.2	29	14
24.9	49	29
10.7	33	14
56	32	17
34	81	56
9.6	55	41
48.9	32	9
10.3	39	18
27.2	41	17
112.1	47	14
184.4	32	17
28.1	32	17
23.7	45	27
33.2	79	55
59.6	33	16
6.2	36	12
79.4	45	28
19.7	41	22
5.6	40	11
72.3	27	12
139.9	38	12
45.3	36	20
6.8	27	14
31.4	24	12
2.5	37	27
0.2	39	16
5.4	33	21
19.6	32	17
56.5	29	12
1.2	45	20
224.5	45	26
0.4	32	15

7.4	26	22
75.7	29	13
54.3	32	15
111.7	18	8
10.9	35	18
133.8	37	14
1.2	64	26
74.1	26	21
137.1	32	15
49.9	32	20
12.1	28	16
46.2	32	17
126.7	26	13
36.9	26	17
0.8	32	17
2.3	32	17
15.7	52	27
14	42	14
10.5	38	17
120.2	37	15
19.4	32	17
39.8	37	21
22.1	33	14
36.2	32	17
8.9	42	17
57.6	28	12
20.9	28	17
48.8	45	17
32.1	36	19
0.9	32	17
16.3	32	18
28.8	42	14
10.8	34	15
20.9	43	13
138.1	32	20
72.9	31	13
129.2	32	17
16.3	30	14
55.1	36	24
66.1	24	9
39.3	36	17
37.2	42	14
28.1	29	14
1.4	55	23
181.4	27	16
34.1	42	21

7.1	42	19
219.9	34	13
6.2	36	47
5	43	34
143.6	30	18
0.2	36	20
0.2	34	21
4.2	31	15
78.7	27	12
25.5	55	36
3.8	38	32
3.6	32	17
59.6	39	14
94.2	32	17
4.3	52	22
22.1	32	17
0.2	45	16
38.7	42	17
11.6	50	42
1.3	27	20
21	31	21
3.5	22	12
80	30	15
34.6	43	26
1	36	14
25.5	45	23
58.8	32	18
123.6	28	17
0.19	46	20
11.7	32	17
8.8	54	17
44	31	14
25.3	32	17
1.7	55	17
77.8	27	12
2.1	39	17
0.6	109	134
100.9	32	17
140.8	37	26
14.2	40	18
175.3	91	68
43.1	30	22
62.6	43	36
125.1	31	6
75.8	31	22
101.8	51	24

23.1	95	53
375.6	31	23
70.7	32	17
2.9	32	17
3.8	33	12
16.6	34	16
152.4	19	8
59	30	12
141.8	33	14
143.7	35	16
38.8	45	26
1.7	32	17
8.5	57	20
5.2	32	17
17.4	40	14
73.8	35	15
0.8	32	17
1.2	28	13
43.9	30	12
5.7	59	26
8.7	28	11
18	29	14
42	41	18
1.2	32	22
132.2	33	14
185.7	36	15
94.6	27	12
6.2	45	17
76.6	40	17
6.1	109	81
5.6	39	38
58.4	29	19
8.1	38	14
54	21	16
27.4	30	23
9.1	25	16
3.4	42	24
4	108	65
220.9	22	13
39.4	24	17
16.3	45	17
262.8	54	65
1.7	32	17
3.3	43	19
14.1	20	13
21	35	16

7.1	62	15
72.7	33	26
27.9	40	22
8.9	38	24
40.4	32	14
7	47	46
320.1	32	17
10	32	17
96.5	33	12
3.5	149	50
142.9	28	14
60.7	46	31
23	31	21
2.1	32	17
9.4	37	23
15.9	132	205
64.1	39	19
74.4	32	17
20.8	37	15
52.2	49	42
16.8	56	22
17	30	19
165.2	37	14
3	32	17
36.2	32	17
28.9	20	15
325.7	63	43
25.6	32	17
59.9	40	37
31.5	32	17
32.9	26	16
11.9	40	18
15.4	32	16
70	36	28
0.2	27	18
97	19	9
5.4	49	22
6.6	32	17
1.8	64	33
2.5	32	17
35	32	26
1.5	32	31
129.8	166	122
0.7	41	27
0.2	26	19
42.8	38	23

0.3	57	47
42	36	20
142.4	32	17
0.6	38	14
0.5	30	15
35.9	30	17
3.6	44	25
2	41	13
6.1	50	22
2.2	54	15
21	31	17
56	25	11
9	32	17
36.5	25	16
21.9	54	68
26.7	45	14
99	22	13
1.3	39	20
0.3	30	20
11.1	39	17
1.2	32	17
1.2	66	36
9.1	23	17
9.3	46	30
2.8	34	14
255.7	32	17
102.1	39	45
61	33	20
40.6	49	41
29.2	31	20
25.2	27	18
10.8	30	20
1.8	31	16
104.9	36	13
0.6	61	53
5.6	126	47
0.2	51	23
33.2	37	14
23.7	64	67
130.7	52	18
16.3	22	16
1.8	36	19
2.3	34	17
15.1	36	17
18.5	41	23
1.2	32	17

0.19	35	16
5.5	33	16
1.1	30	30
4.6	52	25
1	27	5
6.2	84	81
133.5	58	38
21.6	32	19
0.4	32	17
18.2	30	23
42.9	32	17
2.2	42	23
0.4	32	17
67.9	25	9
48.9	32	17
5.1	39	26
1.1	28	13
2.2	42	15
0.9	24	12
13.7	39	17
0.2	33	23
1.6	83	64
0.19	30	27
1.3	31	20
36.6	28	11
231.5	42	21
6.9	32	16
48.8	31	14
124.6	32	17
119.4	32	17
0.9	41	35
0.2	35	20
1.7	45	35
25.4	31	22
32.9	39	19
2.6	44	12
69.2	37	24
8.8	34	11
53.3	44	36
45.4	41	17
51.9	23	11
22.4	43	22
6	42	24
9	32	17
101.5	28	14
9.2	94	100

19.1	39	12
34.8	32	17
8	57	23
47.4	28	20
119	37	34
1.3	41	16
3.1	47	23
40.8	18	12
101.6	28	12
63.9	26	10
15.4	23	12
60	44	17
24.2	39	24
3.5	48	25
0.6	39	21
10.2	50	27
2.4	37	17
1.3	44	38
0.2	40	23
4	33	14
0.5	55	38
4.5	62	17
5.1	42	18
4.7	39	11
110.2	30	13
62	42	18
1.5	49	21
1	22	16
0.2	32	17
25.9	48	18
67.9	36	20
21.3	75	46
108.6	47	55
1.5	31	14
66.4	29	6
19.3	50	31
37.8	25	15
4.6	47	27
0.2	26	11
101.1	32	17
47.1	41	12
0.8	42	27
15.6	36	20
64.3	31	19
61.5	39	20
34.6	34	10

28.5	30	19
7.1	32	17
4.8	32	17
6.8	37	25
1.2	40	20
56.6	28	15
214.5	28	19
1.1	51	22
75.6	37	15
1	29	25
15.6	36	18
60.4	39	14
6.3	53	36
36.1	23	16
18.9	38	17
37.8	32	17
4.2	152	90
8.8	27	12
1.4	31	25
15.3	52	63
3.4	43	32
9.5	51	23
9.7	57	38
57.8	41	19
115.2	30	14
52.1	59	65
11.8	42	27
17.3	43	15
181	26	14
7.1	32	17
65.6	48	21
220.1	25	14
26.3	32	17
15.1	27	9
15.4	31	28
23	24	14
3.3	28	15
1.3	28	22
94.6	48	23
0.9	196	190
63.2	43	20
141.3	21	14
17.4	35	25
35	40	16
2.2	48	26
14.1	55	28

18.6	31	18
116.5	24	15
139.1	55	35
64.1	43	19
1.3	63	14
4.2	46	27
7.6	33	20
5	45	27
1.1	115	106
60.5	25	12
10.6	37	20
27.9	48	32
45.6	31	22
132.9	150	143
6.8	43	31
3.7	30	18
11.6	31	13
0.19	30	14
3.6	191	113
22.6	68	85
0.19	51	19
8.4	35	28
3.6	39	11
107.3	105	162
2.9	37	16
9.1	39	19
22.9	22	14
16.7	30	13
130.5	33	13
41	27	16
9.1	39	18
0.3	53	43
2.6	32	17
1.4	38	10
11.6	37	14
0.4	32	17
33.8	23	16
0.3	40	19
36.7	62	43
24.2	32	21
5.6	36	18
65.9	32	17
59.4	72	60
45.6	31	14
7.7	50	21
124.1	26	12

0.2	66	42
48	24	12
4.9	41	19
7.4	47	22
16.8	32	17
40	37	21
48.1	32	17
200.7	32	17
113.3	35	19
2.5	27	20
72.3	27	14
91.7	60	34
4	42	27
13	56	40
0.19	52	19
50	33	17
0.19	76	56
209.5	44	20
195.1	39	22
0.2	72	31
3.2	33	17
4.7	42	23
47.4	34	22
10.7	36	18
161.2	23	27
63.7	27	16
35.9	30	14
30.8	32	15
3.2	45	18
61.6	26	12
6.2	40	14
113.5	30	12
8.9	39	22
79.4	42	31
93.6	30	18
66.3	24	17
130.4	37	16
5.4	46	30
61.9	35	37
24	35	16
132.3	28	24
67.6	25	16
41.4	27	14
6.2	41	32
0.2	50	53
64.5	33	14

60.7	66	47
298.6	31	27
0.19	58	21
1	43	26
0.2	39	20
42	27	16
15.4	32	17
14.6	26	20
12.3	38	14
93.2	32	17
15.6	390	168
31.3	26	11
0.9	45	21
19.1	28	13
3.5	45	26
0.2	29	18
89	38	17
48.7	48	28
0.8	36	26
0.2	87	64
43.6	40	17
96.1	91	52
28.4	31	16
13.7	46	22
58.8	32	15
156.9	21	13
1.9	33	17
3	32	21
23.7	40	22
0.8	64	31
77.6	21	14
25	47	20
216.8	24	14
1.6	91	89
63	30	11
0.2	42	22
0.8	32	17
66.1	35	17
5.4	34	19
49.7	22	18
46.9	27	11
35.7	30	17
51.7	56	21
27.9	34	26
54.6	30	15
3.2	35	21

5.6	39	16
43.1	29	17
11.1	30	20
50.2	43	24
70.4	33	17
5.8	43	23
0.9	39	18
0.2	32	17
27.6	40	22
20.1	24	16
42.2	36	40
1.1	92	29
28.1	32	21
2.1	33	16
11	33	22
27	34	14
41.8	33	17
0.19	29	13
17.9	36	11
0.7	34	22
1	61	37
9.3	41	18
10.6	41	19
10.3	44	32
0.4	46	33
3.4	69	88
6.7	32	17
0.2	35	14
1.2	64	13
102.5	33	13
5.1	30	14
44.3	39	30
31.5	50	51
12.7	42	17
119.8	25	17
7.5	32	17
0.19	35	18
15.2	37	25
160.7	37	18
2.6	32	17
0.2	32	17
1.1	28	12
0.2	29	9
4.7	23	13
128.7	22	13
11.6	35	22

75.6	40	29
11	57	23
45.9	54	65
7.2	36	23
35.6	47	20
222.4	33	24
2.6	24	11
1.8	38	26
232.4	33	14
5.5	32	17
0.4	69	44
110.8	32	12
96.2	38	18
51	37	35
19.1	34	14
28.2	33	28
35.7	46	23
15.1	32	17
1.3	36	22
31.7	26	14
47.4	32	17
2.3	43	22
50.9	29	22
20.8	35	20
4.6	62	38
3	32	17
9.7	36	23
104.2	57	24
5	31	16
554.1	35	21
33.1	34	16
27.2	35	23
24.7	26	17
19.2	32	24
1.5	47	38
16.2	32	17
4.3	32	17
5.5	37	17
25.2	30	14
76.8	30	19
5.4	32	17
0.19	32	20
3.5	192	206
5	75	45
34.3	35	20
115.4	26	14

1.1	32	17
57.8	24	17
9	62	26
3.1	56	26
0.4	45	15
34.9	34	18
18	38	19
2.5	74	68
28.3	49	24
8.5	52	32
175.6	32	17
76.3	32	17
1.1	160	63
231.4	31	19
1.3	43	18
12	40	12
4.9	57	20
9	30	12
42.8	67	158
3.4	32	17
243.7	35	19
45.1	32	17
1	45	40
0.3	37	19
158.1	18	45
19.6	36	22
5.5	38	19
3.4	43	23
28.8	32	17
0.9	46	19
36.1	48	22
5.5	47	22
42.9	21	16
76.4	32	15
6.6	31	16
26.8	37	18
4.2	29	15
1.4	67	62
5	36	13
42	824	295
54.5	23	16
79.8	36	11
27.8	23	10
19.4	32	17
141.2	31	23
7.9	53	18

4.4	37	28
12.5	32	17
19.9	39	17
15.5	45	30
0.2	46	18
39.5	43	23
0.19	28	16
16.1	24	13
7.8	33	20
0.4	37	20
67.6	29	15
6.3	28	6
20.7	42	18
0.19	55	32
120.1	31	20
1.7	32	17
62.8	28	25
58.8	124	169
7.2	46	26
27.9	24	14
4.4	38	18
132.3	48	27
91.9	23	12
38.3	43	31
181	36	19
41.6	22	14
0.5	25	13
5.1	32	17
1.9	39	24
73.5	24	12
38.9	34	25
39.8	36	22
63.6	32	15
63.3	24	13
13.3	32	25
171.2	34	18
17.2	54	28
13.4	38	17
55.5	32	17
0.19	34	12
48.8	40	19
0.4	40	37
143.8	71	16
12.3	37	29
0.2	62	32
54.2	33	14

13	33	19
36.4	33	16
10	64	56
27.7	36	16
59.3	35	16
18.9	50	34
3.9	38	18
60.8	38	28
63.4	34	16
4	37	20
0.2	107	44
53.2	41	23
9	61	38
17.2	45	38
5.9	45	30
10.2	31	15
2.6	43	27
14	32	17
35.1	18	17
2.9	42	31
25.8	89	69
9	32	16
24.4	42	24
12.2	31	13
9.5	32	17
6.8	38	19
148.2	23	11
65.5	18	16
141.8	19	13
1.8	43	20
360.8	28	14
4.8	45	20
1.5	46	17
74.3	43	31
14.6	31	15
9.7	37	22
110.5	47	26
2.4	27	17
0.5	66	31
1.3	60	47
70.9	70	45
42	32	17
50.6	94	30
42	26	14
0.19	38	16
0.19	35	18

288.1	30	20
175	32	16
0.8	37	18
9.2	32	17
0.2	24	15
3	110	95
0.6	30	17
39.9	39	35
38.5	32	12
78.2	32	17
58.4	36	16
2.2	44	21
143.4	32	17
4.7	66	38
0.8	38	18
18.1	37	17
9.3	50	67
157.3	48	21
0.2	29	20
14.1	42	23
68.8	37	18
28.2	43	21
245	32	17
269.7	40	25
143.1	21	34
3.2	30	21
124.9	36	16
45.8	29	25
0.6	53	49
158.1	32	12
16.8	33	13
133.9	788	646
176.6	15	10
104.6	33	15
1.3	28	12
47.6	28	27
2	32	17
14.8	29	14
0.2	57	32
12.5	88	68
8.9	28	36
72.6	61	53
9.9	41	14
89.4	7	31
53.7	38	18
0.7	42	25

32.5	28	23
13	37	34
114.1	35	19
21.8	29	16
5.9	25	14
0.2	21	12
11	28	64
8.2	32	17
1.2	149	82
2.6	32	17
2.8	38	16
5	33	14
5.7	30	16
17	27	13
3.9	31	11
0.8	31	14
16	32	17
1.7	36	13
30.9	117	90
73.4	30	17
86.1	36	31
6	32	18
1	44	30
1	32	21
5.3	36	20
194.3	41	26
61.1	33	24
13.9	32	19
9.5	32	17
0.2	25	15
0.19	39	14
48.8	33	11
1.6	29	13
30	36	21
215.6	23	15
64.2	46	21
0.5	31	20
4.4	43	18
0.2	30	21
61.8	26	16
1.9	31	15
106.4	64	29
67.6	29	18
12.1	68	40
1.9	22	15
15.1	32	17

70.3	39	13
38.9	30	21
32.4	34	18
174.5	36	27
59.3	36	15
0.3	32	19
71.4	23	18
75.7	29	12
13.5	63	26
11	52	30
7.6	27	13
40.8	34	18
64.8	28	18
0.19	35	23
0.4	29	15
26.8	38	19
138.8	27	14
22.3	39	23
3.4	35	18
24.7	40	16
23.2	42	16
147.1	40	16
5.8	42	19
44.2	57	29
102.2	31	17
15.1	32	17
175.4	23	14
145.8	27	12
92.8	32	17
0.8	51	19
2.8	32	17
7.5	33	21
52.7	38	14
41	32	14
4.6	32	17
3.6	145	205
1.9	34	18
0.2	41	27
4.4	36	18
139.6	32	15
26.2	32	17
28.5	34	28
11.1	42	12
3.3	963	17
2.8	47	19
69.3	31	15

15.1	55	29
0.2	27	30
47.7	31	16
0.19	25	11
1.1	42	30
16.6	29	12
39.1	32	14
14.7	23	17
16.2	44	23
69.9	37	16
44.9	31	29
1.7	24	11
14	42	124
109.8	26	21
62.8	46	24
152.9	21	16
56	32	17
97.9	26	16
77.2	32	20
54.7	35	20
45.8	35	12
21.4	61	53
3.4	36	20
49.1	22	14
77.3	25	12
220	39	18
1.5	30	15
1.3	47	18
2.6	28	13
242.7	45	26
0.3	26	18
70.4	23	11
3.8	32	14
74.4	29	14
2.6	32	17
22.9	32	16
278.8	37	35
31.3	38	20
22.1	32	17
30.4	32	23
5	32	14
106.9	32	17
26.7	35	20
162.5	26	14
22.9	49	31
174.9	32	16

11.1	157	194
8.6	32	17
76.6	34	17
67.7	32	17
117.3	52	20
3.3	31	15
11.2	29	13
5.7	38	19
23.5	30	16
75.5	32	17
10	33	18
3	32	17
157.8	35	28
79.8	25	13
8	60	40
0.9	27	23
12	26	21
13.8	32	19
59.1	41	22
3.1	55	25
66	20	11
1.4	18	16
242.9	23	11
0.7	30	23
17.8	32	17
1.1	34	26
63.4	36	20
41.5	40	9
2.8	38	15
33.1	32	16
32.6	47	43
50.7	23	18
7.6	42	25
96.2	31	15
43.8	32	17
0.2	38	15
0.19	30	7
1.1	35	18
8.1	28	14
1.2	57	36
2.6	60	52
10.8	1173	698
2.9	61	30
36.8	53	46
22.7	44	16
49.3	27	14

31.2	29	15
52.4	37	27
0.5	33	15
1.1	56	43
0.4	52	51
14.8	47	30
4.3	42	25
19.9	26	17
78.1	46	39
1	47	31
279.4	34	28
8.1	29	14
0.8	31	17
9.1	32	17
5.1	51	24
33.4	35	16
13.9	44	18
36.3	54	27
127.8	34	14
11	44	12
0.2	32	16
9.2	56	27
3.5	37	20
28.7	30	12
4.2	52	26
5	42	25
92.6	29	15
176.7	38	37
2.2	32	17
2.4	39	21
19	30	19
44.3	32	17
30.5	37	20
60.1	32	17
51.7	32	18
5.1	33	17
103.8	23	15
2.8	32	17
39.1	26	17
184.9	23	15
16.4	22	10
32.7	40	24
10.4	32	17
75.6	23	11
9.7	26	23
79.7	28	14

154.7	45	30
13.6	39	21
8.6	32	22
0.2	23	9
137.9	39	13
0.9	34	13
0.8	26	22
0.19	29	20
0.4	30	23
136.3	32	15
319.7	28	17
25.2	40	44
64.8	32	17
1.3	33	13
0.2	48	31
5.7	32	17
5.6	32	17
16	32	10
53.3	27	14
15.6	31	25
1.2	41	43
129.2	30	13
5.4	50	19
57.7	32	17
24.7	32	17
46.3	38	13
0.2	30	24
22.2	42	23
14.9	44	24
6.4	54	39
0.2	24	14
54.1	72	75
0.19	32	17
10.3	35	13
24.7	34	24
10.3	40	16
16	49	18
11.1	41	22
7.6	32	17
1.4	40	20
1.5	44	22
37.4	30	20
4.6	40	25
9.2	35	19
101	26	11
144.2	25	21

0.2	32	17
114.3	32	17
47.4	38	17
194.6	34	19
40.3	33	18
1.5	46	21
15.5	31	21
57.1	33	17
57.7	37	21
99	37	16
18.2	46	23
12.1	28	14
0.4	32	32
0.4	81	61
2.3	82	52
27.3	95	79
54.7	38	19
126.9	39	19
41.8	28	13
93.9	24	22
51.4	21	9
214.3	32	17
0.3	32	17
0.2	34	32
12.1	35	15
15.5	34	21
2.6	63	48
107.7	19	12
1.7	32	17
8.6	36	13
63.2	40	19
41.7	32	19
8.8	41	27
52.3	24	10
16.9	34	20
13.9	33	19
43.7	40	21
5.3	21	20
6.7	57	37
3.7	47	19
32.7	32	18
100.5	36	29
14.8	34	16
0.19	33	14
54	43	35
77	32	17

213.9	20	13
3.6	29	17
2	32	17
48.5	26	16
0.3	32	17
12.4	38	16
29.5	32	17
11.8	38	31
72	32	17
31.7	36	21
16.1	37	31
3.4	25	11
0.9	32	17
30.4	28	13
58.3	32	17
10.2	27	16
225.7	22	10
59	37	26
1	30	17
27.8	37	17
173.8	32	17
173.9	42	15
94.1	20	14
148.3	32	17
2.4	35	17
49	32	19
1.5	50	24
0.4	30	15
171.7	40	10
149.2	40	20
13.3	21	9
61.7	93	43
28.9	49	40
67	22	20
1.8	59	44
0.7	31	18
171.2	29	12
174.8	43	28
11.8	44	26
3.8	51	27
3.7	42	28
29.1	58	24
15.5	34	19
4.2	44	21
0.2	34	24
2.5	38	14

10.1	38	13
10	35	21
22.2	34	16
0.4	27	14
3	34	17
9.9	36	27
12.1	66	21
3.5	36	17
50.7	26	20
13.1	37	19
1.6	27	14
4.6	39	33
57.1	32	17
4.3	42	14
9.5	27	10
33.2	27	14
1.1	48	39
1.5	40	20
9.1	42	16
6.2	35	11
0.6	33	16
0.3	39	20
52.2	23	12
39.1	34	18
0.19	30	20
34.3	36	22
0.7	32	17
130.7	30	20
0.19	34	16
28.8	35	16
6.5	28	13
10.4	32	12
192.6	25	15
0.2	52	31
7.2	26	19
4.3	32	17
325.5	33	14
0.9	50	41
4.6	32	31
0.19	54	24
0.5	30	13
80	47	28
0.2	56	59
180.5	30	16
9.2	32	15
145.6	37	22

13.4	37	20
27.9	22	12
3.6	23	17
62.3	31	18
173.3	27	16
3.8	44	32
1.1	32	17
1.4	32	17
61.8	39	12
24.6	23	18
44.8	32	17
4.7	21	17
99.5	31	14
2.9	32	16
32	25	16
57.1	37	26
106.9	32	17
3.9	68	34
1.6	30	19
6.7	32	15
9.8	41	14
3.1	32	17
5.6	45	26
2.7	187	151
251.4	34	15
2.8	36	17
2.1	40	17
17.7	25	18
0.19	28	14
12.7	31	17
0.8	37	15
64.3	32	17
0.19	24	10
149.1	50	21
2.3	22	13
61.6	28	15
2.7	32	17
45.6	31	31
41.6	37	18
0.3	40	17
426	18	10
0.19	30	12
78.9	35	22
2.7	43	24
5.2	40	21
15.1	32	17

0.2	18	10
109.2	37	21
0.19	19	11
88.3	26	16
58.7	33	14
0.19	28	7
8.7	67	45
2	32	17
16.4	35	19
2.9	35	14
0.6	41	35
90.9	43	19
208.1	29	14
0.7	56	32
60.9	32	17
128.6	27	18
190.2	47	42
19	26	11
103.1	43	15
13.9	40	16
23.2	43	24
0.3	19	14
20	37	29
0.19	32	17
27.4	51	21
3.7	34	18
0.19	64	52
1.5	32	17
17.5	32	24
22.7	41	27
15.4	45	18
1.3	39	16
1	24	9
0.5	18	13
0.8	32	17
1.2	44	20
47.9	28	24
42	42	22
10.9	26	17
0.7	35	16
24.2	32	15
1.4	47	21
3.5	99	63
115	32	17
8.1	34	13
9.6	27	12

2	47	24
0.19	32	17
0.19	38	29
0.9	32	17
57.4	32	17
1.6	22	9
0.5	39	27
16	32	17
1.5	32	17
0.5	42	31
5.1	32	17
46.6	30	14
33.5	32	14
98.2	33	24
23.8	40	17
3.4	51	44
3.2	32	17
21.9	32	17
11.1	32	17
0.19	31	13
0.19	37	34
7.5	43	15
13.5	64	43
2.1	38	15
40.3	28	18
0.3	23	12
47.5	37	14
9.3	34	18
1.7	32	17
8.9	39	15
120.6	32	17
21.5	32	17
28.5	35	16
9.3	32	17
98.9	22	12
4.3	52	15
17.4	47	16
110.7	37	18
33.7	35	12
11.9	37	13
5.2	51	19
50.6	43	26
64.4	46	21
12	31	14
11.8	27	7
9	44	26

35.1	27	16
37.2	36	20
54.5	37	19
0.2	33	23
50.2	45	16
0.19	30	16
1.1	23	10
61.8	24	12
14	33	14
8.1	33	17
23.2	30	12
7.2	30	21
0.19	38	34
105.2	32	11
102.3	42	47
0.19	37	19
21	29	23
18.1	27	14
20.3	33	11
14.2	27	17
77.4	32	17
134.2	19	14
1.7	37	19
2.4	36	17
2.5	39	17
0.8	62	42
31.1	35	21
0.5	38	20
1	45	13
2.1	75	60
16.8	27	22
3.2	44	25
66.8	36	15
8.3	97	34
43.6	27	14
0.2	38	38
2.5	32	20
1.5	63	37
0.8	37	15
62.3	46	42
109.3	25	13
14.4	28	10
92.1	35	16
62.1	20	16
0.19	44	26
43	28	13

0.2	44	43
107.4	68	19
3.4	35	35
23.8	48	28
30.9	34	17
42.6	30	13
2.6	31	26
38.1	36	13
27.5	32	17
169.9	45	19
49.4	26	16
4.4	31	25
123.1	32	12
10.6	39	23
16.9	29	17
0.4	51	30
7.7	35	31
4.9	49	25
2.9	38	35
3.2	34	20
0.4	32	19
1.4	41	28
0.7	41	32
1.4	39	15
144.7	23	18
26.2	28	14
143	27	14
217.4	22	16
39.4	35	14
19.8	39	17
61.4	68	69
62.4	41	18
246.5	34	16
9.1	45	30
27.6	32	17
6.6	41	17
8.3	53	18
0.5	36	22
12.4	31	21
51.1	28	11
133.2	32	17
0.19	32	17
1	47	17
2.2	33	21
10.7	39	15
13	36	21

179.4	20	9
304.5	41	24
6.2	47	16
36.9	32	21
1.6	67	65
5.7	37	17
22.1	32	17
147.5	31	14
50.3	36	16
4.4	32	17
7.5	26	11
71.5	32	17
2.8	32	17
29.7	45	16
107.7	39	15
0.3	31	18
41.8	30	16
32.6	32	17
31.1	47	38
125.4	47	88
0.7	35	23
1	45	14
14.4	34	13
64.6	37	20
3.6	35	13
2	38	20
22.8	27	14
6.5	52	16
39.2	39	19
78.6	37	18
69.5	27	20
19.6	32	17
97.9	32	15
0.5	35	22
0.19	36	21
0.8	36	16
143.8	38	22
3.8	53	115
2.8	29	18
13	28	16
21.8	32	17
288.9	47	17
0.19	38	15
5.3	44	23
44.1	31	12
26.7	30	18

51.6	34	20
231.1	68	47
0.6	50	73
18.5	30	25
9.6	22	12
59.8	31	15
3.6	32	17
37.6	30	12
15.2	34	30
27.4	74	76
36.9	28	15
0.19	37	17
18.4	30	15
49.3	102	68
0.3	32	17
0.19	47	50
45.7	21	12
0.19	22	13
0.2	43	28
2.3	36	19
2.8	31	14
89.8	17	14
26.9	36	33
0.3	49	21
22.4	33	13
5.7	32	28
43.2	32	17
30.9	32	17
32.3	37	30
10.1	32	17
27.7	48	22
61.6	22	12
58.2	44	16
14.2	93	65
40.7	31	20
13.6	32	17
0.8	86	13
45.6	67	45
8.5	32	17
11.6	41	11
71.5	32	17
1.6	37	14
21.8	32	17
19.9	25	13
77.1	30	15
4.9	41	29

8.8	31	15
0.19	38	13
0.7	38	13
3.7	57	35
2.7	32	17
6.4	32	17
0.6	36	13
18.6	34	17
0.4	58	16
0.19	25	19
1.6	32	17
3.7	32	17
9.3	27	13
10.5	37	14
2.1	35	14
135.8	43	29
0.19	58	38
21.1	32	17
1.6	32	17
17.1	32	17
3.9	45	6
22.8	44	16
0.19	33	17
26.6	33	21
47.3	42	16
120.9	38	22
62.5	29	20
230.3	47	18
3.5	44	20
10.2	26	13
7.2	30	18
52.3	29	22
16.3	36	22
212.8	50	28
126.6	19	11
0.7	60	52
77.8	19	17
9.7	32	17
31.2	37	23
5.1	20	11
0.8	32	17
39.6	28	12
9.6	27	14
41.1	34	24
0.19	24	13
95.5	25	15

41.3	32	17
20.8	31	21
206.3	18	11
15.7	32	17
41.9	50	22
5.8	38	19
2.8	26	16
7.2	32	17
3.4	46	18
107.4	40	17
48.3	36	14
67.8	33	20
48.2	32	17
10.2	34	18
39.9	30	31
37.9	42	19
54.5	29	22
54.9	29	20
135.9	53	27
0.19	31	23
7.9	43	14
3.3	28	14
6.5	138	119
197	46	36
40.9	32	17
17.1	55	46
3.3	24	17
45.3	32	17
97	112	124
50.8	28	21
1.1	45	33
7.5	37	18
0.3	48	19
7.2	35	22
4.3	32	13
7.2	32	17
61.6	31	13
44.1	46	30
45.5	32	17
6	30	15
0.19	26	14
6.7	44	25
21.1	32	17
3.9	30	15
369.5	39	27
29.1	32	17

31.3	43	18
19.9	35	18
25.4	34	19
33.9	32	10
0.19	31	14
35.3	31	14
1.5	32	15
59.7	40	16
0.19	35	24
40.4	68	51
5.5	32	17
4.5	37	19
18	53	22
27	32	17
1.3	39	16
0.5	60	45
0.3	32	17
55.7	42	17
42	32	13
134.3	36	20
7.3	36	19
37.6	32	16
1.6	55	12
10.4	39	17
84	28	23
35.5	32	17
5.4	30	13
21.7	24	9
0.19	41	30
53.7	31	15
115.8	32	17
3.4	32	17
35.6	32	14
4.3	35	18
0.19	28	15
0.2	33	10
0.19	40	21
41.8	36	20
138.9	36	12
13.3	28	13
38.9	35	18
1.4	56	24
0.9	46	17
17.8	37	19
4.6	57	39
0.19	32	17

2.7	24	10
12.6	34	15
80	24	17
1.7	35	20
66	52	31
139.2	32	17
67	38	21
359.4	33	24
33.5	32	17
25.6	32	17
11.3	32	17
41.6	32	17
63.4	30	15
1.7	37	15
0.7	31	14
0.19	35	19
79.1	33	12
45.4	32	17
10.4	30	17
29	32	17
51.8	32	17
2.4	48	24
3.8	38	22
66.8	32	17
105.3	28	15
11.8	34	14
14.8	39	23
35.6	34	19
0.19	43	27
9.6	27	13
136.2	28	10
5.7	38	19
51.5	34	15
2.1	48	18
0.8	36	15
26.3	26	15
0.9	37	14
0.5	31	12
1.4	51	26
8.7	64	23
3.3	33	16
40	27	18
6	38	21
9.7	37	17
19.7	34	28
15.8	30	20

34.3	34	20
16.4	36	18
0.5	47	30
28.3	37	19
38.7	32	17
1	32	17
0.3	25	14
71.9	46	21
2.3	40	16
75.5	33	51
4.5	37	16
1.6	32	17
0.9	33	15
0.19	44	17
32.2	33	16
0.4	32	17
71	32	17
111.6	30	14
11.8	47	20
107	28	15
0.19	35	15
0.19	36	12
3.3	29	10
3.9	31	11
0.3	37	26
3.1	32	13
7.6	45	17
0.19	34	14
3.1	37	18
160	30	13
139.5	32	17
23.7	29	13
33.5	31	14
0.2	39	22
1.8	57	43
2.9	32	17
20.8	32	17
2.1	32	17
1.4	32	17
117.2	40	28
0.19	27	13
12	35	15
188.5	32	16
24.5	46	17
14.8	22	11
10.5	36	22

3.2	32	17
0.5	34	15
0.19	44	40
0.19	31	16
1.3	26	11
2.8	55	18
27.3	45	31
0.6	30	26
1	43	27
11.6	32	17
0.5	44	17
54.7	27	14
61.5	32	17
65.6	66	42
4.7	39	21
5.8	35	18
7.4	37	16
48.1	39	22
34.3	52	20
53.8	32	17
103.3	29	21
7.8	33	16
9	33	15
4.1	32	17
32.4	30	13
4.3	37	17
2.3	50	28
9	32	20
2.2	47	17
200.8	32	17
16.5	45	14
12	31	15
0.19	39	16
7.7	42	12
4.8	49	19
0.7	47	20
0.6	56	21
29.9	32	17
4.3	47	20
20.9	41	15
21	46	32
0.19	33	23
5.7	32	16
61.1	40	21
3.8	41	17
40.8	29	12

101.8	32	17
1.8	41	21
6.7	38	14
179.1	32	17
45.9	29	13
8	46	17
0.2	40	16
0.19	18	12
2.7	46	21
23.1	42	38
3.5	139	123
168.4	25	13
0.19	83	36
116.4	28	20
8.2	42	17
77	24	12
0.3	46	25
2	67	47
8.9	28	18
2.7	45	20
28.8	40	18
73	42	21
27.4	38	25
9.4	45	18
0.8	48	20
185.1	32	17
68.3	32	17
5.8	32	17
1.7	94	33
0.3	39	20
44	38	14
0.19	29	21
61.1	33	14
42.4	48	21
0.6	33	16
2.9	28	15
5.7	30	16
115.3	43	21
22.5	44	17
6.1	38	22
18.6	31	17
28.4	32	13
0.19	34	23
4.6	44	34
101.9	39	11
4.6	37	19

33.3	35	17
4.6	32	17
3.9	68	25
38.4	35	23
2.2	39	20
14	49	19
4.1	34	17
14.3	52	23
0.19	35	19
96.9	33	25
1.9	32	17
11.5	36	18
59.2	50	16
6.3	53	28
28.5	41	16
75.3	26	14
9.9	199	283
3.6	36	17
0.19	38	21
4.2	45	21
181.1	25	24
5.5	37	24
17.1	32	17
37.2	36	20
14.3	37	25
57.8	32	17
54.8	32	17
2.3	37	11
1.8	61	16
6	32	17
0.9	32	17
0.6	36	18
37.6	32	20
13.7	33	13
150.7	32	17
4.4	41	15
34.5	42	22
5.9	28	12
2.1	56	24
7	47	19
12.2	35	14
30.5	26	17
2.9	32	17
2.8	42	24
3.8	30	20
7.4	32	17

0.19	32	19
0.4	32	22
0.2	37	16
0.19	69	17
30	29	21
32	27	16
3.6	36	17
15.4	27	14
8.1	58	50
8.6	39	25
12.9	36	12
13.5	32	17
0.19	42	15
0.19	63	36
29.2	38	19
33.4	88	21
36.2	36	17
108	51	57
7.1	37	16
7.1	41	14
1.3	43	26
116.4	33	17
36.8	32	17
141.5	25	11
107.5	25	21
7.1	27	18
56.8	37	21
45.8	47	32
67.6	197	98
29.9	34	18
0.19	39	23
22.8	32	17
0.4	32	17
157.3	38	14
8.7	35	21
0.19	23	10
128.3	32	12
26.5	32	17
2.8	39	16
19	40	19
22.9	42	57
42.5	54	22
2	35	28
2.9	32	17
2	37	15
161.2	20	17

20.2	32	17
0.8	38	16
141.3	39	26
13.4	37	15
8.9	47	29
4.1	34	20
35.3	38	16
23.6	28	26
139.2	21	17
61.7	32	22
58.4	41	16
4.3	42	23
1.4	30	22
242.1	38	10
0.19	32	17
24.2	151	284
1.3	35	13
20.5	45	37
45.2	40	18
56.4	28	13
0.19	42	26
55.5	31	15
3.8	40	16
3.4	32	17
20.5	36	24
12.1	34	11
0.4	49	28
41	26	13
24	29	16
53.9	34	20
24.8	35	14
13.2	27	12
71.9	36	15
27.3	29	12
1.9	49	31
24.9	32	17
12.2	51	30
2.7	52	70
2.2	50	28
31	31	16
4.3	36	28
4.3	41	50
33.9	57	17
0.7	62	25
53.2	32	17
10.3	47	22

60.6	33	19
22.8	32	17
26.7	37	18
7.5	45	37
42.9	31	24
44.1	21	14
0.2	32	16
138.9	21	14
108.6	44	20
21.7	39	47
50.5	32	18
18.9	35	24
67.9	32	17
14.2	33	16
41.2	35	13
17	26	17
126.6	52	28
16.7	31	16
2	34	13
258.2	63	32
140.5	44	19
2.1	47	15
108.7	33	15
78.2	28	14
7.7	35	26
0.4	45	23
0.19	37	25
2.7	44	12
6.7	35	19
17.3	38	11
41.5	30	16
3.4	47	22
49	32	11
255.5	42	22
5.2	34	18
180.7	63	10
107.8	32	17
3.7	46	16
3.7	43	17
2.2	26	14
42.9	27	11
11.9	32	15
6.1	38	26
251.8	30	18
11.3	40	30
0.7	62	23

22.4	32	16
14.7	28	19
21.7	31	13
0.7	57	35
9.9	32	17
4.5	32	19
22.1	42	18
4.3	51	20
97	17	8
22.8	37	28
134.4	34	18
25.1	50	29
37.8	32	17
51.7	33	19
1.7	47	21
1.4	62	41
70.6	72	32
6.6	32	20
20.6	38	16
27.4	28	14
14.6	35	13
5	32	17
10.2	32	17
102.9	26	16
4.1	32	17
123.9	34	22
31.7	30	16
0.9	35	14
18.7	41	18
239.8	24	13
6.8	30	13
69.4	26	16
36.8	32	17
118.9	35	16
1.7	35	15
132.8	23	10
38.8	37	18
0.2	31	19
1.6	62	48
11.9	46	20
2.4	32	17
4	32	17
20.3	32	26
41.4	32	18
262.2	83	24
66	37	27

161.1	33	14
0.3	32	10
0.4	38	23
53.9	31	16
54.7	44	21
3.1	31	17
36.7	29	23
131	31	21
128.7	32	18
0.19	52	20
22.3	32	17
0.3	37	15
5.4	28	19
75.1	34	18
52	39	28
0.19	34	19
8.8	36	19
0.9	32	13
156.9	26	17
7.8	49	27
0.5	32	17
78.2	32	14
101.4	43	36
17.5	32	17
3.7	50	36
0.2	46	21
78.1	26	16
21.3	56	19
45.6	46	39
0.19	24	14
0.6	31	20
71.2	35	14
3.6	62	53
62.7	33	17
5.6	41	15
29.4	277	15
1.8	29	24
26.2	35	30
60.1	53	44
7.8	30	14
2.5	39	16
0.19	25	9
114.1	47	138
24.4	32	27
71.1	32	15
37.9	23	17

10.1	32	17
5.3	40	13
10.9	32	17
2.7	29	15
94.6	48	19
0.4	30	22
1.1	119	70
1.6	32	17
12.6	45	23
15.1	32	17
2.7	32	22
156.4	33	16
65.4	55	23
79.5	25	21
50.7	28	15
5	74	33
7.8	24	14
36.1	36	16
35.3	28	14
10.7	46	24
56.3	40	16
0.19	45	17
12	34	19
0.2	59	76
10.8	45	22
18.6	24	11
8	27	26
24	30	13
5.2	45	20
1	48	49
18.5	27	11
11.3	33	16
16.7	36	24
114.2	20	15
3.2	35	19
1.6	35	24
27.7	67	41
0.19	38	20
6.9	41	18
0.2	74	10
98.9	46	29
18.9	39	15
10.3	122	119
9.4	36	24
9.7	47	31
33	40	20

0.19	67	65
40.4	61	18
11.8	28	13
18.7	27	9
26.5	35	37
37.3	32	20
4.2	35	15
4.6	56	44
165.5	39	13
5.7	36	21
1.4	32	22
11.7	43	21
100.6	25	14
0.19	34	19
0.19	53	53
45.2	25	12
0.6	35	20
50.5	32	14
7.4	42	12
2.4	47	50
15.1	36	15
19.4	38	25
26.7	48	17
14.6	28	19
30.6	32	19
4.2	47	29
8.2	32	17
11.9	40	22
71.4	32	17
6.8	32	17
26.9	32	12
0.8	32	17
0.19	40	19
47.9	31	16
17.8	77	57
25.6	35	16
0.19	38	26
1.5	34	18
14.9	39	18
9.2	32	27
1.7	42	22
47.3	35	21
0.19	35	22
29.1	35	28
44.1	33	25
5.1	37	25

302.5	68	26
9.5	34	18
10.6	25	27
73.2	31	16
0.9	44	14
143.2	25	16
121.3	29	16
1.2	39	18
1.1	44	20
5.7	32	17
9	27	16
59.4	40	25
0.5	28	20
44.2	33	13
9.4	47	28
0.4	36	16
14.3	32	17
1.1	39	24
3.5	37	19
0.2	42	29
3.3	28	11
5.3	30	15
4.6	32	17
2.1	42	75
0.4	36	15
4.8	31	23
14.7	31	15
12.6	29	20
237	29	13
13.8	51	24
6.2	44	36
0.3	47	38
0.19	50	39
54.4	49	20
152.6	24	8
116.4	32	17
94.2	29	28
8	30	16
1.2	39	24
0.19	35	21
200.4	40	17
65.2	28	17
17.6	30	11
4.1	48	27
1.5	40	22
59.2	239	102

114.7	36	19
39.2	34	20
2.7	36	20
5.1	51	27
153.9	31	18
3.6	29	14
291.4	23	10
23.8	42	35
25.3	32	17
16.2	43	15
58.4	29	17
1.9	48	17
0.19	53	20
0.9	43	31
4.3	46	20
213.5	30	19
6.3	32	17
1.7	32	15
74.9	32	17
0.5	34	22
14.9	27	18
0.19	34	19
2.7	31	13
11.7	29	15
44.6	44	18
3	54	20
1	67	39
63.7	30	13
20.7	36	18
39.7	39	18
65.7	506	392
3.5	32	17
0.7	32	17
39.1	47	14
5	29	16
10.7	57	23
6.4	51	20
0.7	35	19
12.4	27	12
155.4	32	17
2.3	46	16
35.8	33	13
35	46	15
59	38	17
2	31	17
16.4	41	18

23.4	28	13
8.4	38	17
96.9	32	16
6	42	15
8.4	23	8
0.9	46	18
11.7	89	192
37.4	32	17
72.7	38	18
0.6	736	327
1.3	62	18
130.1	35	19
254.4	68	44
67.7	32	17
57.6	47	22
4.1	37	26
26.7	35	22
0.19	108	52
76.9	32	22
1.5	27	19
37.1	32	17
278.2	20	10
112.4	22	9
31.8	31	21
11.6	51	24
1.1	50	39
3.9	33	18
24.6	46	48
0.3	38	26
2.5	30	17
0.19	50	28
26.8	23	10
8.9	36	19
0.8	34	20
29.1	33	15
4.5	35	13
0.4	27	12
2.4	33	13
253.8	21	13
166.9	35	16
74.2	45	18
6.2	30	29
0.19	42	24
4.6	37	20
3.4	34	22
11.5	54	29

2.1	35	28
130.5	42	22
46	32	17
39.5	36	30
42.4	39	18
53.6	34	12
0.19	42	20
41.6	26	17
40.2	30	13
0.4	27	17
2.2	24	23
34.9	36	24
37.7	22	11
8	32	17
163	31	13
1.9	51	76
240.3	39	22
21.4	39	16
4	32	17
18.3	28	11
1.5	40	14
11.9	32	17
168.4	31	21
11.1	47	36
6.9	52	22
3.5	44	30
24.8	19	12
126.7	56	19
3	35	11
4.7	52	44
49.8	40	38
18.5	22	15
224.6	26	12
14.8	41	19
15.6	38	18
9.3	40	14
0.4	84	10
16.5	33	17
2.6	32	17
0.19	44	22
182.9	60	31
24.5	24	13
0.8	53	20
0.19	42	16
29.5	40	14
5.5	44	28

60.8	36	15
79.2	47	31
2.7	30	17
1.1	26	19
36.4	35	17
62.3	31	16
1.5	43	14
5.8	35	33
54.2	32	17
24.9	37	19
0.6	39	18
1.4	33	19
0.9	32	17
0.2	32	28
0.19	43	21
0.19	32	17
0.5	49	32
29.4	24	15
0.4	57	42
7.7	28	15
9.7	32	17
293.5	32	17
284.1	27	15
120.4	32	17
50.8	32	17
6.4	41	26
8.4	247	289
18.4	37	18
0.19	42	25
31.6	43	13
4.2	41	33
2.8	47	24
4.7	32	16
0.3	26	12
0.19	38	24
3.7	36	18
50.7	25	11
68.9	31	16
3.7	53	30
2.2	35	16
56.4	32	15
28.2	54	31
68.6	36	21
0.9	32	17
61.4	32	17
0.5	49	18

0.19	32	17
0.3	48	15
16.5	45	21
2.1	37	18
0.5	65	30
3.6	49	27
226.2	54	44
0.4	38	27
18.7	29	18
1.3	57	24
0.19	65	47
45.1	44	17
3.7	40	15
7.2	48	20
26	32	17
44	37	18
20.1	45	18
51.7	21	12
5	35	18
51.5	30	13
8.5	33	15
10.1	39	31
5.2	32	17
0.19	32	17
120.4	42	20
0.19	26	12
46.8	18	12
0.19	44	20
10.2	32	18
1.9	34	13
13.5	34	15
168.9	32	17
12.8	38	13
0.4	52	17
1.3	102	51
3.4	58	20
30.1	29	18
0.4	32	17
42.3	31	20
9.2	42	18
26.9	39	26
9.5	35	14
8.7	32	17
2.3	34	14
18.7	31	15
3.1	33	15

22.8	27	20
181.6	121	174
0.19	30	8
33.4	56	61
15.1	32	17
6	32	17
8.1	32	27
0.4	32	17
3.8	32	17
159.5	24	18
128.8	40	26
0.19	41	17
0.6	40	21
35.6	27	18
14	47	28
1.6	38	36
0.6	37	28
2.1	100	94
2.4	37	22
2	32	17
0.9	40	41
0.3	50	20
46.3	40	13
6.2	32	17
4.9	38	17
47.2	32	17
7.1	51	20
3.1	32	17
94.9	28	39
1.4	50	20
21	34	27
30.8	39	16
0.19	43	20
69.1	32	17
71.5	437	273
1.7	39	19
15.9	36	16
34.9	33	14
7.1	32	17
120.2	32	20
79.7	32	17
40.4	32	17
3.3	32	27
11	32	17
41.1	32	17
42.7	44	21

2.3	41	25
0.19	59	17
21.5	44	26
0.8	31	18
28.4	38	24
42.2	37	37
61.1	30	19
1.2	39	15
8.8	32	17
4.6	56	29
35.8	32	17
0.6	30	15
5.5	32	17
3.6	43	24
182.7	30	23
17.5	40	22
13.7	46	19
4.9	34	10
2.2	35	23
5.7	56	36
113.1	30	12
45.5	38	28
146	101	47
0.19	27	14
6.6	32	17
5.7	36	22
0.4	38	16
50.1	35	20
34.3	29	10
60.4	29	10
0.19	40	27
14.4	39	18
48.4	51	43
7.8	87	61
3.3	51	17
116.7	37	17
41.1	37	16
2	39	13
187.2	44	21
12	50	17
1.4	32	17
1.6	44	19
21.6	31	18
53.9	50	24
3.8	30	18
47.2	25	21

5.7	41	22
0.5	27	16
0.9	45	22
14	45	25
2.6	50	18
0.9	22	18
2.6	84	45
156.9	41	33
20.3	30	14
9.2	33	14
1.8	44	25
25.2	46	27
73.5	36	29
19	33	15
0.3	96	65
24.6	55	40
2.3	88	72
25.6	105	60
0.3	33	25
0.19	44	17
2.2	50	25
64.2	29	17
5.3	34	34
2.1	45	21
5.6	66	46
68.2	23	15
40.4	42	39
1.7	47	20
8.4	38	21
8.3	55	23
0.19	36	17
12.6	44	22
23.3	32	17
2	41	32
24.5	36	20
0.19	43	25
0.8	23	16
25	28	14
5.4	47	38
0.2	37	15
8.9	57	29
7.6	45	20
76.8	31	29
0.3	66	37
37.2	22	16
2	40	18

0.19	22	17
2.1	34	21
0.9	43	30
17.4	23	14
10.4	37	17
1.1	35	28
0.3	34	15
35.2	36	13
0.8	67	27
14.9	40	35
1.8	40	23
17.4	87	165
4.6	32	36
38.4	34	18
14.6	44	16
2.7	48	22
0.19	44	24
21.8	48	22
127	17	16
0.4	33	16
1	48	28
2.7	33	13
0.19	49	20
6.2	37	22
60.3	150	70
0.7	35	24
0.9	58	47
9.2	32	17
12.9	43	25
3.2	32	17
14	32	17
2.6	98	45
7.2	26	11
0.19	44	34
9.8	25	12
23.1	49	22
1.4	36	26
5.8	45	19
115.9	29	33
50.7	31	16
4.8	73	22
29	29	12
1.9	32	17
69.7	38	20
1	51	24
21	41	23

1.9	48	24
11.5	41	22
0.2	42	25
10.7	43	29
31.3	23	11
65.6	32	17
8.1	44	19
153.8	40	31
8.5	41	13
2.4	62	38
116.3	32	17
25.1	37	15
0.7	43	22
7.9	39	19
9.1	52	29
5.9	57	20
0.19	39	17
14.3	35	12
0.5	49	26
56.7	30	10
0.19	32	17
5	46	17
7.6	47	25
287.5	32	17
2.4	30	15
0.6	50	36
35.6	25	21
0.9	32	22
0.9	34	24
20.1	49	38
32	42	32
6	32	17
10.7	36	14
3.7	32	17
25.4	54	21
33.6	31	19
25.8	28	14
15.3	31	17
10.5	36	17
0.5	95	55
6	40	17
0.19	32	17
254	32	17
66.5	40	43
115	32	17
0.19	87	74

20.1	29	16
3.4	45	20
8.8	50	17
7.3	43	29
9.9	48	19
2.3	29	22
64.3	41	17
1.6	30	14
0.5	32	17
19.2	41	22
0.19	36	21
24.8	32	17
0.19	47	19
0.2	43	18
1.4	34	15
9.4	32	17
2.9	41	19
2	42	11
1.4	48	17
9.3	19	9
13.8	39	43
18.3	36	21
20.2	43	23
2.1	72	23
248.1	18	12
1.6	36	16
2.2	65	30
13.5	35	13
0.19	57	23
2.6	42	22
4.6	40	25
5.3	42	18
20.2	46	14
14.8	27	19
18.3	25	15
0.19	39	41
38.9	34	20
6.4	51	22
34.7	32	20
9.9	32	17
0.6	27	17
47	27	15
0.19	41	16
14	29	19
8.1	51	63
314.2	25	13

44	36	39
22.6	46	37
76.3	16	15
6.7	28	12
18.6	41	15
6.6	32	17
68.6	21	18
0.19	24	7
11.2	43	31
28.2	38	23
10.6	39	32
7.9	45	18
2.8	32	19
0.5	50	35
0.19	45	27
19.4	46	24
6.5	35	16
5.1	33	24
5.7	47	14
12.7	64	29
36.4	31	18
0.19	37	28
1.7	26	16
33.9	46	32
3.5	40	15
37	32	19
19	42	21
1	38	24
49.5	25	20
4.2	34	18
30.1	33	13
12.4	38	17
3.1	39	20
59	41	15
5.1	50	29
11.7	40	25
115.6	50	34
2.1	31	16
0.19	75	40
1.1	38	41
4.5	61	50
8.4	35	15
99.9	21	13
3.8	37	22
60.7	32	17
3.4	43	13

1.7	29	14
15.6	33	16
28.5	23	14
166.4	32	17
4.2	22	16
0.8	43	23
52.7	26	15
0.19	24	29
8	37	18
0.19	44	20
24.1	34	19
14.2	42	11
27.6	28	19
31.3	37	17
0.19	30	16
0.19	40	18
3.5	34	18
23.8	57	27
33.3	36	28
0.9	48	37
55.3	45	26
18.5	40	20
2.9	41	15
4.3	62	23
2.7	49	20
7.2	56	26
21.4	36	12
3.6	38	22
94.6	26	10
5.4	22	17
1.7	59	18
12.7	29	22
0.19	38	28
1.9	75	35
5.1	57	21
50.5	32	17
16.6	99	46
2.8	48	8
103.6	32	17
64.7	65	33
19.1	32	17
2.3	46	18
0.3	32	18
0.5	43	21
7.1	38	17
0.19	24	15

3.6	35	14
32.4	51	17
59.1	32	14
4.1	68	38
2.1	41	15
1.1	48	35
9.3	25	14
52.6	28	13
5.7	47	20
28.9	25	18
11.8	36	21
0.19	25	9
0.7	53	27
3	29	17
4.7	27	13
16.2	35	25
2.8	36	30
51.7	73	18
1.8	32	17
6.7	32	17
1.3	32	21
0.19	37	31
0.7	26	19
20.6	32	17
22.3	41	15
1	42	18
1	32	17
14.7	52	18
63.8	22	15
1.8	45	19
7.7	39	19
2.4	49	21
47.2	31	20
34.3	31	74
10.1	38	23
3.8	109	97
12.5	33	9
14.7	37	18
5.7	49	25
5.3	27	19
12.1	32	17
3.4	39	33
1.6	36	22
0.7	46	25
28.6	40	24
8.8	55	33

18.2	44	15
10.2	38	17
38.7	41	50
2.4	24	20
6.3	32	17
16.9	44	19
46.2	39	41
3.9	42	21
138.2	27	20
2.5	42	22
0.7	32	17
1.5	34	31
6.4	32	17
23.3	32	20
1.7	49	24
10.6	46	22
110.9	59	22
2.3	65	34
1.4	49	18
34.6	32	16
0.19	48	52
1.8	26	19
26.7	49	29
3.7	34	11
1.2	36	22
26.3	32	22
16.6	33	17
8.3	36	19
20.6	30	23
10.5	31	19
20.1	36	16
0.7	51	22
7.2	37	35
11.2	41	18
10.3	32	17
29.3	34	23
116.9	39	32
0.4	70	55
5.3	31	18
25.9	32	17
8	42	11
51.4	26	12
74.3	49	40
2.4	53	33
3.1	45	17
152.7	32	17

10.6	46	25
8.5	112	148
202.6	43	23
0.2	50	25
3	35	23
7.9	41	17
33.5	32	17
3	31	14
4	35	25
23.2	76	93
7	30	11
44.5	32	17
6.1	52	18
4.7	39	35
4.5	74	58
22.5	27	14
1.6	39	29
3.5	25	24
0.5	49	33
0.19	24	9
0.5	89	42
3.8	28	22
14.3	54	23
11.1	51	17
2.6	43	21
102.5	20	18
30.1	82	64
37.3	28	11
37.5	34	16
9.5	47	18
0.3	37	17
16.1	36	27
30.1	72	9
0.3	31	11
4.5	34	23
3.2	33	22
7	51	21
17	39	22
2.4	34	12
9	31	16
0.19	32	17
64.4	46	43
49.6	26	17
100.8	32	17
98	32	17
1.9	44	22

30.3	30	13
4.9	164	187
0.7	44	19
5.3	28	13
0.5	32	15
1.9	56	19
38.4	32	17
0.19	50	48
1.4	37	12
118.5	46	25
2.9	52	29
0.4	42	22
23.6	39	18
3	40	18
117.1	36	16
1.8	83	33
0.6	29	15
239.5	26	12
5	32	17
0.8	38	18
47.1	32	17
0.3	51	25
16.4	49	24
15.4	26	15
16.7	45	27
0.19	32	17
1.4	51	27
16.5	31	16
10.4	1161	456
5.1	40	19
104.5	35	20
0.5	42	20
3.3	71	62
38.9	42	17
7	42	16
39	47	23
5.1	31	20
0.9	32	26
17.3	33	19
5.1	43	26
7.5	48	17
1.7	37	19
218.9	27	10
11.9	46	28
20.1	32	17
35.2	41	22

7.7	44	31
60.6	31	16
3.5	39	26
29.4	31	11
21.6	34	15
0.5	43	15
1.7	45	18
45.7	33	15
16.2	37	31
0.7	64	37
171.4	36	54
23.1	48	33
17.8	36	21
1.9	56	32
5.6	33	26
0.3	52	31
29.1	30	16
7.2	71	33
22.5	32	16
0.5	54	28
8.6	39	16
4.3	46	21
113.4	25	17
4.8	62	52
48.1	30	16
35.3	31	18
29.5	35	11
14.7	55	41
0.19	32	17
0.7	34	17
7.7	23	14
0.5	49	48
0.19	32	16
25.8	41	18
29.2	35	15
42	56	26
0.19	38	17
16	42	22
5	41	16
23.3	32	17
3	55	35
13.4	42	15
62.4	36	19
14.8	57	28
1.4	48	17
0.19	44	22

117.5	32	13
55.9	31	16
20.2	43	23
143.9	18	18
10.1	54	19
20.7	32	21
1.5	36	34
61.5	32	17
65.6	33	24
4.4	30	29
0.19	25	13
0.7	116	54
67.3	26	15
3.6	42	31
0.4	32	17
5.2	47	21
2.6	37	39
4.7	32	24
2.8	43	17
3.6	32	15
49.7	37	38
3.3	51	27
0.8	47	32
0.8	29	13
10.8	121	115
2.9	38	25
35.6	34	22
6.9	43	16
20.1	29	17
3.8	66	37
13.7	27	21
1.5	42	18
18.9	50	28
53.2	32	17
0.9	58	32
20.5	22	22
2.7	34	11
3.2	59	37
6.6	43	19
2.8	40	22
0.19	55	19
0.19	33	15
3.2	24	20
0.4	42	35
10.6	47	24
0.6	48	17

2.1	34	32
24.1	34	15
38.9	32	17
0.3	29	31
0.6	36	15
7.8	53	41
25.5	32	17
47.4	32	17
20.8	46	12
11.2	33	14
71.5	34	19
0.7	37	35
0.4	33	16
2.1	57	20
6.1	76	43
7	32	17
67.5	33	20
44	41	20
13.8	40	16
6.8	30	20
6.2	32	17
1.3	45	30
0.7	33	12
4.9	44	22
3.4	38	17
6.1	32	17
14.8	32	17
25.7	29	15
8	55	31
33.7	37	17
0.19	27	19
0.19	35	19
156.1	32	19
1.8	39	18
13.5	34	8
47.8	32	17
281.2	38	17
26.3	60	36
20.5	44	22
1.6	32	17
46.6	34	38
117.8	53	60
72.5	304	216
30.7	39	29
0.7	76	53
54.8	51	45

13	43	30
117.2	23	18
25.8	32	22
365.2	93	23
12.8	32	17
4.3	47	19
150.5	24	13
1.1	40	27
36.5	28	23
57.5	33	15
12.6	38	24
66.1	42	15
130.5	27	20
13.7	55	31
1.2	41	18
4.7	40	35
7.9	34	20
1.8	72	34
4.9	71	37
0.4	35	18
61.2	40	14
0.5	38	20
0.19	38	18
15.9	56	27
2.1	61	47
13.8	38	28
0.3	60	38
33.3	36	16
2.4	43	24
0.19	38	40
137.7	30	12
6.4	32	17
28.2	25	14
2.9	30	12
12.6	46	22
44.4	32	17
22.1	27	15
0.4	36	20
0.2	48	15
14.1	31	19
41	31	14
0.3	48	25
7.8	38	16
45	38	20
57.4	30	14
6.3	46	29

0.3	39	17
120.1	32	17
0.3	32	17
2.1	35	31
4.4	40	17
6.1	38	18
0.19	38	11
221.1	36	25
1.4	57	24
4.5	32	17
4.9	32	17
0.19	35	29
2.3	48	25
2.5	34	13
69.9	45	23
16.4	36	18
24.4	43	14
2.2	34	14
20.1	39	16
9.8	38	13
65.4	32	17
0.9	32	17
0.5	36	18
6.4	61	19
3.5	55	24
0.19	41	19
1.5	32	17
15.9	32	17
44.5	32	17
0.4	47	18
1.1	40	35
9.2	31	14
79.2	56	21
13.9	44	19
20.4	59	20
0.9	47	26
3.9	36	15
19.4	28	26
42.1	130	57
27.3	50	18
17.2	38	15
0.5	38	20
32.1	27	15
0.19	32	18
2.4	47	24
107.1	181	211

130.3	32	17
29.1	36	17
0.19	30	22
0.5	62	30
22.8	39	21
4.9	45	21
0.6	20	12
10	43	23
26.3	32	13
10.9	32	17
30.3	32	17
26.3	43	21
9.5	35	16
21.1	45	16
4.9	42	22
0.8	65	32
1.1	32	17
9.9	34	14
1.3	40	20
2.4	35	23
5.9	30	12
1.6	35	14
23.4	40	31
11.3	56	18
4.2	31	21
34.3	32	17
19.4	36	16
0.5	32	17
30.2	77	70
0.5	43	14
4.2	32	17
6.4	30	12
18	48	35
1.8	45	26
60	32	17
19	43	20
161.2	28	20
1.8	45	16
1	32	17
3.9	50	36
1.4	35	19
1.1	44	14
28.4	36	22
3.1	34	22
9.2	47	14
1.3	32	17

0.3	29	14
42.8	47	21
2.1	32	17
1.3	45	42
8.1	92	82
27	34	20
229.1	17	12
2.6	32	15
36.7	28	10
4.3	32	13
50.9	34	16
19.6	81	44
34.5	33	14
15.8	57	93
3.5	41	17
0.19	42	34
15.7	95	108
0.2	28	15
0.5	22	27
1.1	32	17
46	42	26
0.19	35	30
0.19	31	16
1.9	55	24
24.4	22	12
53.5	25	18
23.1	34	16
38.4	31	19
32.1	37	18
0.19	32	17
124.6	19	20
2.6	39	16
20.6	46	17
11.9	28	11
0.19	57	55
20.4	32	17
4.5	45	18
3.8	35	19
17.1	47	17
123.7	31	16
0.3	48	26
1.5	58	29
6.2	28	15
1	32	17
15.3	43	20
4.3	41	19

40.4	32	18
4.1	59	42
19.8	39	17
65.5	21	16
109.9	16	16
3	32	17
22.6	30	24
0.19	32	17
3.3	41	23
42.5	32	17
92.1	54	33
27.8	45	26
21.5	32	17
40.3	42	51
39.5	29	19
3	60	58
63.9	42	30
11.1	33	33
0.6	30	23
14.2	52	20
150.6	35	14
21.7	41	27
0.7	54	26
6.4	33	19
8.6	45	20
5.1	51	28
18.5	39	20
31.3	40	19
8.4	408	259
50.6	32	17
0.8	40	22
18.7	35	20
6	32	20
21.8	42	47
0.3	36	26
37.7	27	12
26.5	42	32
1.4	42	16
40.5	32	17
56.7	49	39
5.6	42	17
7.1	48	23
34.6	50	27
38.6	32	17
3.2	49	19
78.4	36	14

3.4	60	25
127.9	55	32
2.9	32	17
72.5	26	12
13.1	53	16
6.2	45	15
4.9	57	26
0.19	40	12
16.9	24	10
1.9	44	19
158.6	19	15
14.4	32	17
58.1	29	24
1.7	50	22
56.8	39	16
0.4	32	17
15.1	32	17
3.4	33	22
109.9	32	14
9.4	46	19
11.2	55	48
22.1	32	17
2.7	32	17
42.8	32	17
8.5	56	27
39.7	47	21
2.7	60	29
0.7	43	17
1.4	47	19
143	32	13
18.4	25	17
4.9	42	15
6.3	50	18
10	32	17
78.5	134	666
2.1	32	17
10.3	52	37
6.2	36	16
122.8	31	17
75.1	39	17
43.5	32	17
79.8	29	15
12.4	47	18
22.3	32	16
1.1	40	28
2.5	45	24

2.9	32	17
135.9	37	16
0.2	43	25
1.5	41	19
57.5	36	14
3.8	52	30
0.4	51	32
2.9	34	17
29.3	32	17
18.8	32	17
13.6	38	14
134.4	35	17
2.2	50	20
22.8	45	22
2.4	47	17
15.6	36	28
57.7	48	20
38.5	34	16
2.1	54	16
8	36	23
1.6	31	14
4.1	32	17
7.8	41	15
3.9	34	17
0.4	32	17
2.9	31	14
0.19	31	15
0.5	55	28
0.6	29	14
3.8	44	13
162.6	27	17
6.5	34	19
9.8	34	17
132.7	31	11
6.4	42	14
22	34	15
2.4	24	15
95.4	32	17
38.7	42	21
5.6	322	139
103.7	27	16
21.2	40	26
4.8	41	15
3.7	28	16
120.7	38	17
240.9	43	23

4.4	36	20
9.1	44	34
10.8	47	35
28.3	34	16
53.5	66	33
341.5	20	13
63	27	14
142.3	24	10
65.5	29	11
37.7	31	15
8.7	38	15
0.19	72	43
78.7	30	9
1	35	15
16.4	50	16
11.6	34	15
12.8	39	17
38.8	55	19
106.8	38	19
73.4	41	13
4.9	23	9
111.4	30	11
17.1	32	14
142.4	37	17
192.3	30	9
3.5	32	14
116.5	36	17
108.9	45	29
79.8	26	12
70.3	23	13
8.4	48	19
45.9	32	10
52.9	31	13
0.7	43	20
14.2	23	16
0.7	39	20
48.8	26	15
0.3	31	16
99.3	26	10
33.1	34	13
18.1	33	19
32.4	27	9
117.6	45	18
54.9	25	11
58	32	17
36.1	25	13

22.5	36	15
10.1	32	20
94.6	33	19
50.6	29	12
17.2	32	17
55.2	37	13
138.5	51	20
28.4	32	18
47.8	32	17
3.2	33	22
33	42	29
19	29	19
12.3	44	15
3.8	45	17
67.7	28	9
25.4	36	17
56.1	36	17
150.8	32	14
2.4	50	20
79.5	41	21
2	42	18
22.2	30	14
117.5	41	26
0.7	29	13
1.6	32	17
3.8	32	17
59.5	26	13
6.3	32	17
7.1	32	17
9.8	28	23
110.3	26	10
42.1	26	15
50.6	59	29
65.9	32	13
56.9	32	17
43.4	33	14
13	39	13
37.2	26	10
118.1	22	9
31.7	32	12
185.7	34	20
45.6	54	29
97.8	29	14
37.8	38	14
12.1	38	14
53.8	27	16

19.1	35	20
0.3	33	18
7.2	35	19
3.7	35	42
171	56	42
6	37	20
9.7	32	17
99.8	33	20
39	43	7
115.1	35	17
135.1	38	19
132.7	30	19
53	35	13
22	61	19
162.4	32	17
124.3	37	12
49.6	29	14
46.9	29	14
0.4	32	17
16.1	36	14
14.5	35	21
7.9	28	15
26.4	24	12
30.7	29	13
20.9	39	18
6.3	35	15
42.2	41	18
16	32	19
4.8	32	17
4.6	52	30
15.6	50	13
62.2	32	17
37.2	29	9
20.2	33	13
207.2	27	14
28.8	33	14
6.4	29	12
0.19	32	11
48.8	32	17
4.6	28	16
44.5	59	20
51.1	33	20
1.7	43	22
61.9	29	13
26.4	31	15
74.3	27	15

262.6	23	16
4.9	37	20
3.8	29	8
61.4	31	13
60.1	31	14
5.7	32	14
44.1	44	23
14.5	32	17
10.1	28	12
178.6	38	17
13	31	12
3.4	84	19
3.8	37	18
3.4	30	42
42.2	39	23
1.6	113	35
9.5	51	25
60.6	37	18
0.5	32	29
20.2	35	16
10.9	33	17
71.9	27	13
193.2	31	11
58.2	32	17
91	32	17
46.1	34	14
94.4	52	39
35.4	35	18
59.6	40	17
41.3	32	17
88.5	32	17
111.4	39	14
4.7	41	14
3.2	32	17
20.7	45	17
54.1	34	9
6.5	31	12
75.3	38	19
14.9	50	18
13.8	30	14
103.4	28	20
10.6	50	19
68.3	46	19
56.3	34	18
75.2	47	23
4.4	34	15

6	32	17
103.5	33	16
99.3	35	18
0.4	32	17
2.6	32	17
64.2	47	23
37.6	37	16
131.2	27	16
4.6	38	21
2.3	30	12
1.2	45	29
62.5	40	17
61.1	40	20
40.5	31	21
101	32	17
8	43	12
1.7	32	17
91.2	35	20
54.4	35	28
7.4	32	17
46.2	27	13
4.8	100	186
4.1	63	24
127.6	32	17
36.4	33	15
47.4	51	42
207.6	36	14
5	32	17
13.4	32	17
101.5	37	15
69.6	21	9
6.5	218	258
27.8	26	9
3.4	32	17
100.7	25	13
2.4	42	19
18.9	33	14
18.2	36	15
226	37	18
51.5	57	26
54.8	32	17
74.9	31	14
150.3	31	16
145.7	32	17
70.4	35	14
4.8	28	13

44.7	32	17
19.7	34	13
26	41	19
2.6	33	16
37.2	45	21
59.8	44	24
54.7	63	30
46.1	38	18
7.2	81	63
183.5	61	41
229.5	27	10
42.4	32	32
109	35	16
7.5	52	12
10.7	37	17
69.4	33	11
3.8	43	23
1.3	34	18
0.4	45	17
9.8	43	20
239.8	31	19
144.7	32	19
1.4	37	10
110.1	27	11
99.4	35	22
78.8	34	15
0.19	25	22
0.19	39	19
223.8	60	33
0.2	160	171
62.5	26	13
0.19	33	18
4.1	44	18
27.8	126	50
14.7	149	129
8.3	32	27
25.3	43	19
48.3	32	17
20.1	31	12
56.2	32	15
6.7	30	5
47.6	40	16
19.5	29	15
78.7	26	12
1.7	52	12
112.5	34	11

30.7	45	17
114.1	25	14
32.7	32	19
38.6	32	18
25	45	19
27.2	84	51
72.7	31	13
27.7	48	32
59.8	26	11
66.2	43	15
67.7	38	22
158.4	51	16
18.3	275	136
21.8	24	10
75.3	66	20
12.8	30	20
31.1	34	20
68.4	34	15
2.8	42	44
44.7	40	24
206.3	38	23
3.4	32	17
47.6	38	20
190.9	28	15
39.8	21	16
1.1	56	20
34.2	46	31
21.2	35	21
146.4	33	14
55.2	35	15
33.6	33	14
59	41	28
0.5	44	19
13.2	40	18
11.6	32	17
7.3	34	12
12.6	41	26
52.8	32	13
13.2	40	19
0.9	45	17
2.3	43	18
15.1	32	17
16.5	46	15
6.6	35	14
5	37	14
120.3	24	12

13.7	61	26
4.8	56	39
30.8	22	19
310.4	25	13
33.3	29	17
11.6	32	14
0.6	32	17
5.1	25	12
53.6	29	15
18.6	31	16
45	43	21
13.9	32	17
0.2	46	27
11.6	32	16
3.2	75	27
17.8	32	17
49.6	46	31
1.4	44	14
0.7	43	25
3.3	43	17
2.6	81	80
195.2	63	17
1.2	30	18
8.6	32	17
25.7	25	17
36.8	38	13
56.6	35	16
2.9	41	16
5	30	21
16.6	33	15
90.9	31	13
0.6	32	17
0.19	28	17
2.4	60	36
2.3	56	36
32.8	39	15
28.9	32	17
63.8	28	21
7.6	29	13
9.5	36	15
30.7	28	15
14.9	45	17
0.4	41	17
35.1	55	50
15.1	31	28
6	33	16

28.8	38	14
65.7	27	16
2.4	32	17
4.4	51	20
6.4	38	17
67.1	34	23
173.2	21	12
37	71	63
9.5	32	18
1	32	17
8.1	36	18
13	43	19
36.2	36	19
45.6	23	15
9.1	71	17
9.1	34	24
5.9	82	78
10.8	42	27
145.3	26	18
135.1	32	17
125.8	32	17
0.19	26	14
36.4	32	17
211.3	36	39
52.5	36	14
194.2	52	17
28.6	32	15
9.3	57	53
0.3	42	36
2.6	45	20
3.3	40	23
21.4	36	16
27.7	30	16
10.4	32	18
5.5	31	16
7.5	93	58
11.3	38	18
2.7	37	15
167.7	23	30
180	35	23
56.9	30	15
6.1	47	20
2.4	49	21
22.1	31	20
1.6	40	17
0.19	35	19

134.8	32	17
4.1	49	58
11.9	36	17
99	33	17
12.6	48	25
46.7	32	17
51.9	29	13
4.6	42	33
4.5	41	29
1.8	38	27
213.2	50	21
0.4	54	36
16.2	33	11
2	32	16
1.1	27	17
41.8	31	11
35.7	50	36
27	51	38
10.1	57	44
2.1	31	15
0.2	32	17
226.5	27	18
135.6	27	11
7.6	32	12
0.3	32	15
1.4	34	22
44.8	23	12
21.1	49	21
104.1	61	56
89.6	35	7
65.8	42	19
291.7	31	15
0.9	32	17
10.7	32	17
49.6	32	17
64.8	35	29
43.9	27	13
1.4	50	25
5.5	33	24
0.9	52	22
26.1	32	28
150.4	53	33
2.7	31	19
10.3	32	17
62.6	30	16
5.2	27	18

40	33	23
12.1	37	19
9.8	68	29
138.3	36	23
6.5	32	18
57.5	30	14
51.5	37	17
54.8	32	17
11.9	26	14
39.5	28	22
8.1	46	19
5.6	32	17
6.1	76	58
49	32	17
27.4	34	15
5.5	42	19
6.2	43	38
4.8	39	20
8	33	24
60.6	24	19
1	28	15
21	40	22
75.7	23	14
69.4	24	7
5.1	41	18
2.6	42	17
10.2	30	14
30.8	38	16
0.3	46	26
121.8	39	22
6.5	32	17
45.2	32	17
11.7	28	12
5.7	32	17
15.4	52	18
146.7	22	19
4.3	61	28
8.9	33	17
0.4	29	13
70	34	15
111.9	38	10
115.2	31	18
3	56	18
15.1	41	17
111.4	70	58
41.6	32	13

191.9	41	21
0.4	39	19
16.6	32	17
71.7	34	15
10.6	42	17
12.5	42	19
28.2	57	53
44.7	46	18
47.4	60	29
56.6	27	13
18.1	31	18
30.7	67	30
145.9	32	12
7.1	32	15
72.5	33	19
47.6	32	17
69.9	30	17
146.8	49	44
20.1	47	35
16	63	68
24.6	27	9
129.6	39	22
42.6	59	20
33.7	24	11
0.2	42	24
1.9	67	41
10.3	102	41
52.9	30	15
49.5	37	20
188.1	32	17
4.1	30	14
18	38	24
8.2	41	24
1.7	34	19
26.9	32	17
34.2	31	14
53.4	22	14
5.5	36	14
12.7	50	53
34.8	25	18
108.6	59	42
2.2	28	10
19.9	62	80
37.5	31	17
0.6	33	17
11.5	54	34

2	133	134
131.9	25	11
8.2	31	12
14.6	36	12
167.3	32	17
1.7	32	16
42.6	30	14
56.3	43	19
5.2	27	13
36.1	25	12
9.1	32	17
26.9	37	21
35.5	30	16
93.8	32	17
6.2	30	14
5.4	30	22
0.3	42	19
3.2	40	19
2.4	31	16
19.2	37	15
52.2	48	22
1.4	56	17
108.4	23	12
1.2	37	20
2.2	31	15
0.2	33	19
15.1	32	17
131.4	27	19
3.4	44	21
1.6	44	16
12.5	40	19
205.9	25	14
40.5	32	16
6.7	30	14
64.8	24	16
107.2	48	28
15.2	50	25
0.7	32	17
0.2	37	20
77.8	32	17
0.3	32	17
0.7	32	22
92.9	25	14
118.3	43	19
2.4	43	18
110	29	10

77.2	41	17
7	34	15
26.2	35	16
9.7	42	19
10.1	37	11
0.19	46	20
28	42	13
20.1	38	11
95.6	42	17
6.2	28	14
0.4	34	15
28.7	29	15
2.2	39	22
55	32	14
34.1	29	13
11.8	46	15
4.7	42	19
104.9	34	15
72.7	32	17
124	31	15
17.5	51	18
16.9	38	20
12	38	23
168.9	29	24
20.2	33	15
191.5	23	13
28.5	42	33
27.6	35	16
4.4	47	20
53.8	32	17
53.2	34	20
78.4	29	11
18.8	32	12
0.19	35	26
75.5	32	23
73.6	32	17
298.8	42	17
3.4	33	22
22.4	42	23
125.7	17	11
178.3	41	37
23.1	31	18
77.5	30	16
4.3	33	19
102.6	67	46
0.19	30	19

0.19	34	24
8.1	190	41
69.9	30	12
4.5	46	27
2.4	32	17
29	31	17
56.4	34	14
3.4	33	13
13	42	21
3.5	32	16
250.3	205	93
1.4	27	20
5.1	36	11
11.1	39	16
123.2	39	14
4.2	52	23
1.6	39	6
13.4	33	11
3.7	32	13
1.2	48	61
9.7	31	12
15.4	38	17
3.5	32	17
41.4	32	17
54	70	42
0.2	49	23
0.2	37	16
36.9	34	18
0.8	55	63
1.3	32	17
22.4	39	17
187.9	30	18
5.9	29	12
5.1	42	21
0.2	31	37
0.19	37	15
0.6	43	20
9.8	40	17
8.9	32	17
67.7	31	18
39.4	29	18
2.8	35	12
135.9	64	44
1.3	38	29
60.4	26	13
4.2	45	17

36.7	32	17
76.8	30	14
3	37	21
28.8	32	17
51.1	38	18
3.9	38	18
7.1	50	20
12.4	32	17
253.9	29	22
3.9	27	20
59.3	36	22
71.1	21	13
87	41	21
2.3	36	28
3.5	32	17
0.7	51	43
34.7	42	17
0.19	70	43
5.2	32	17
0.4	64	30
0.19	64	19
173.2	42	18
29	40	20
5.9	31	16
102.3	32	14
0.6	30	13
66.1	29	16
1.4	47	32
0.8	34	14
9.9	39	63
22.6	67	27
197.9	32	17
29.1	36	20
11.5	36	21
43.9	40	19
18.7	32	17
103.7	32	17
5.5	31	18
39.6	33	20
2.3	89	40
48.5	147	94
4.3	32	17
54	32	17
7.2	55	22
8.4	25	12
62.5	23	13

107.2	32	13
0.5	35	20
49.7	119	177
53.3	30	17
0.19	37	18
104.3	47	25
30.9	37	16
3.1	42	20
26.4	48	24
52.1	115	113
124.7	25	19
10.7	41	30
0.4	51	18
33.1	27	17
2.1	49	18
27.9	54	18
2.8	38	20
0.19	52	68
11.1	39	17
13.7	38	12
0.19	32	17
24.8	41	17
36.6	34	14
14.5	28	14
1	22	19
11	37	21
1.3	38	7
4.9	35	18
6.6	37	31
0.4	33	13
7.3	36	16
4.6	32	9
13.2	72	48
0.19	19	12
66	43	16
2	34	18
172.2	32	17
68.1	35	18
68.2	32	17
46.4	25	20
41.8	32	17
38.3	32	17
1.2	32	17
1.5	43	14
115.1	49	19
0.9	120	119

4.9	31	13
3.5	34	14
4.2	35	23
193.1	32	17
0.19	35	17
129	23	13
4.2	37	28
0.7	47	21
47.2	43	10
64.1	31	14
62.9	38	15
5.2	47	16
74.3	34	14
245.4	93	61
68.5	38	23
5.6	33	14
47.8	34	19
54.5	32	17
52.3	22	9
10	30	12
35.4	32	15
36.5	32	17
35.4	35	20
25.7	36	15
112.7	35	15
11	39	15
12.1	25	14
18.5	76	71
124.3	47	33
7.2	42	18
71.5	33	18
0.3	42	29
8.2	30	22
70.4	59	58
0.7	29	15
13	30	15
2.2	27	19
113.2	28	16
41.1	26	19
0.9	33	17
0.7	35	17
1.4	31	17
0.19	27	15
20.4	26	13
37.2	32	17
29.3	35	21

6.1	38	16
134.4	38	19
19.8	41	21
7.3	32	17
6.2	32	17
0.5	41	17
151.2	74	31
0.19	27	14
98.6	32	25
25.5	32	17
2.2	35	13
121.8	32	17
0.5	32	22
60.9	28	19
17.4	41	19
16.7	47	39
13	43	20
42.6	46	25
6	29	15
38.6	34	18
0.19	146	119
13.8	34	12
0.3	26	12
67	32	17
1.2	39	17
0.19	31	11
14	38	22
5.1	29	12
136.9	44	18
35	32	17
19.7	35	16
3.4	57	21
0.6	29	16
2.7	47	27
34.2	35	16
7.4	32	17
0.9	50	19
68.7	37	14
5.3	28	16
35.4	31	14
9.1	41	27
0.7	54	23
17.2	31	11
1.4	50	26
13.8	29	16
181	46	44

23.2	34	14
114.6	32	16
24.6	32	17
2.4	38	33
146.7	50	20
1.2	41	17
11.7	33	14
95.5	25	16
48.6	32	17
7.8	34	19
10.7	36	15
1.9	32	17
137.1	32	17
16.6	38	17
10	32	13
174.3	32	17
6	34	15
16	32	19
117.1	28	21
135.7	34	14
71.3	23	11
0.8	52	24
0.4	34	17
20.9	40	18
13.4	28	14
30.8	28	11
9.4	32	17
4.8	32	17
22.1	35	22
10.1	38	19
5.9	62	29
143.5	33	22
14.9	32	16
9.8	31	16
49.5	32	21
13.1	39	12
5.4	32	17
26.4	65	45
132.9	41	21
2.7	38	17
4.3	31	19
0.9	37	20
0.6	35	14
115.3	37	15
57.2	34	15
19.3	655	398

16.4	39	22
21.7	32	17
71.2	45	14
18.5	100	117
47.7	26	16
23.4	38	20
22.1	46	14
12.4	43	18
165.5	55	28
7.5	44	19
0.9	32	12
1.8	32	17
4.7	36	7
1	51	18
5.7	45	32
98.6	27	18
25	32	17
55.6	44	21
0.3	42	24
4.4	30	14
3.7	42	15
0.19	53	16
47.2	29	13
35.5	26	11
17.2	49	17
45	28	14
32	42	32
23.7	30	6
2.3	40	32
23.3	29	14
11.2	22	10
136.7	43	20
21.6	27	13
5.4	32	17
43.4	43	8
30.9	38	13
281	33	17
17	48	33
0.3	32	17
19	47	22
6.1	32	16
12.1	33	17
16.5	32	17
67.7	34	12
2.6	37	19
15.6	33	13

9.5	32	17
20.5	34	11
12	32	16
18.7	30	11
3.5	35	16
35.9	50	17
3.5	39	17
0.5	34	19
21.3	36	17
96	24	14
10.2	42	17
2.7	35	16
14.4	60	25
12	32	13
1	49	28
20	37	20
3.6	32	17
170.7	18	11
74.5	30	18
4.7	37	17
30.9	35	19
29.6	42	21
58	35	17
9.7	29	12
2.6	46	16
1.5	107	33
15	47	30
0.19	35	18
0.6	54	32
15.1	26	13
77	44	20
0.5	34	16
20.6	31	13
3.8	38	15
6.9	32	17
4.5	34	13
4.3	35	17
11.3	32	17
0.4	40	20
2.6	30	13
42	38	9
3.7	38	18
45.5	53	31
2.9	48	17
11.4	44	16
19.7	51	16

2.3	54	22
1.4	35	14
7.2	32	17
19.7	51	26
10.4	32	17
2.2	41	19
6.8	42	22
14.3	47	16
39.1	32	17
31.6	32	17
7.5	32	17
56.4	35	32
4.4	43	19
2.2	42	23
32.1	66	43
0.19	26	18
44.3	35	18
0.6	31	13
15.2	32	17
20.7	31	14
116.8	39	18
43.1	44	19
1	51	18
41.1	46	23
18.6	42	25
5.7	41	17
2.3	33	17
31.8	32	17
20.6	23	14
44.5	47	18
72.8	29	12
42.9	38	20
62.1	26	14
2.7	46	26
36.1	38	47
145.4	29	15
2.7	73	14
8.6	40	22
0.8	64	26
0.5	32	17
0.4	33	10
11.8	38	18
9.2	39	29
49.2	29	25
38.3	34	14
4.1	27	12

4.6	31	16
1.9	42	21
2.1	76	49
1.4	31	15
1.6	35	18
14.9	85	68
38.6	38	23
53.8	36	17
3.1	128	99
0.2	58	41
20.4	38	14
19.2	44	36
0.19	45	20
4.4	32	17
271.4	33	18
13.1	32	17
0.3	40	16
202.1	44	16
12.4	48	18
119.6	30	14
65.6	36	23
4.6	32	17
43.6	41	20
4.2	32	17
15.5	36	17
2.6	45	20
8.7	40	18
3	48	21
7.2	36	34
38	34	17
11.1	38	15
32.8	26	10
4.2	42	17
59.2	49	16
15.1	35	15
59	65	43
24.8	30	15
42.9	36	17
71.6	28	10
1.6	39	17
25.1	63	41
2.5	28	14
5.5	32	16
10.8	34	19
0.19	39	29
0.6	37	22

0.4	50	25
57.3	24	13
2.5	32	17
23.9	36	17
16.3	31	26
22.3	35	20
0.4	40	15
3.9	43	18
103.8	177	431
18.7	34	19
54.2	37	20
1.4	51	19
2.1	35	15
0.19	34	15
13.4	79	52
17.7	38	17
0.19	48	17
36.5	32	17
17.4	30	16
19.4	33	15
23.1	31	19
4.4	41	19
67.5	32	17
6	34	19
45.7	42	20
46.5	32	14
5.4	39	23
2	32	17
35.2	35	17
5.9	32	17
397.2	22	11
6.5	32	17
1.2	38	17
3.3	38	16
37.6	23	12
3.7	32	17
1.1	37	19
25.4	34	19
3.6	32	17
56.7	38	19
39.1	32	21
16.1	32	15
1.3	37	17
20.7	40	17
33.3	48	27
2	32	17

12.4	34	23
0.19	50	25
5.4	42	16
0.19	49	33
4	38	22
2.6	39	15
22.7	32	17
66.6	38	17
3.2	32	17
0.6	54	22
9.9	48	30
9.3	33	17
0.19	37	13
21.3	73	81
0.6	27	15
1.2	35	12
58.7	36	15
5.2	35	20
8.9	39	26
0.2	36	14
57.3	20	11
3.3	55	41
18.3	32	17
39.2	32	14
10.3	39	15
48.1	29	20
51.2	25	20
9.4	32	17
6.5	32	17
27.6	32	17
1.1	31	20
46.1	31	54
238.1	24	10
14.3	35	28
7.1	47	22
56.3	29	8
18	28	15
47.2	31	18
2	43	18
9.5	57	21
0.7	37	22
16.3	28	10
53.7	22	13
0.7	31	19
116.9	31	12
8.8	44	26

1.2	44	19
112.3	32	24
9.5	38	20
13.2	49	21
58.1	52	49
2.4	46	26
3.6	30	16
0.8	67	21
50.1	28	16
1.5	37	23
1.3	43	18
1.6	69	27
38.5	32	17
5.6	35	23
4.3	32	13
4.6	56	20
3.3	51	20
11.8	54	22
32.5	34	14
39.5	32	17
7.8	43	20
65.7	25	13
20.3	42	18
27.7	37	18
35.9	32	17
0.2	32	17
3.9	38	14
0.4	53	29
224.1	32	17
4	62	38
26.6	45	12
233.1	34	22
1.6	54	25
12.7	119	121
0.8	44	15
8.2	40	34
76	51	24
118.9	32	17
60.2	43	49
12.5	43	18
34.8	42	25
188.5	29	19
62.4	32	17
5.4	37	17
0.5	32	17
21.7	46	27

109.5	45	18
0.7	124	156
3.3	57	37
22.1	38	18
0.19	50	23
1.2	48	17
7.6	55	26
22.2	28	12
4.5	46	17
31.2	27	15
0.19	39	27
124.7	30	14
2.5	50	19
39.2	33	16
6.6	65	31
173.4	34	25
30.1	30	17
7.5	46	22
12.2	32	15
41.4	40	14
15.1	32	17
0.19	33	18
2.6	34	25
5.9	25	11
3.5	46	39
29.8	35	17
101.4	40	24
20.9	36	23
1.2	54	28
6.3	31	10
5.7	38	13
59.1	35	23
62.9	31	15
56.1	40	32
9.5	37	24
0.2	45	25
0.19	33	31
68.2	38	17
0.3	45	21
23.3	34	16
71.4	26	15
15.1	32	17
2.7	39	19
3.1	36	16
9.4	41	19
41.8	32	17

24.5	32	17
4.9	47	20
19.2	45	19
0.3	46	21
39.7	37	29
10.9	32	17
37.2	32	17
13.4	35	17
1.4	45	28
5.6	41	15
13.4	34	20
14.9	26	19
38.4	40	16
0.3	22	16
1.1	52	22
15.1	60	20
5.5	42	30
2.3	36	22
11.8	37	11
58.4	24	19
12.2	39	15
68.1	32	17
107.9	29	16
14.6	43	21
45	38	23
5.7	43	19
14.6	106	67
0.8	32	17
0.19	33	23
6.7	37	19
21.9	32	20
3	52	21
35.3	41	16
0.19	37	19
0.6	31	15
12	36	17
35.2	37	21
3.4	38	15
3	37	18
7.7	40	14
4.2	83	70
6.4	41	17
28.8	29	13
1.4	49	18
3.3	49	30
18	32	17

2.2	44	15
252.7	31	19
17.5	54	29
195.7	24	17
39.4	32	17
113.4	31	16
14.2	33	21
33.5	41	21
0.19	87	57
74.5	28	12
2.2	49	20
6.7	51	60
102.1	31	13
16.2	33	22
2	54	21
2	31	15
245.5	21	4.9
13.6	38	21
0.2	32	17
10	47	19
15.3	59	31
17.8	46	20
11.7	44	22
0.19	52	24
1.8	32	17
4	80	84
1.2	50	26
72.2	28	15
35.6	32	17
1.8	40	11
20.2	35	13
19	32	17
79.6	25	11
32.1	32	17
108	40	21
27.5	29	23
2.4	64	45
62.1	103	57
16.6	138	100
100.9	32	53
6	49	18
0.19	33	19
161.1	30	13
0.3	35	16
30.1	35	16
3.1	38	20

8.8	47	21
2	69	68
2.1	32	17
7.9	31	21
2.4	32	17
15.7	48	24
29	39	15
8.1	45	26
12	32	17
5	50	20
41.6	29	13
24.7	23	6
4.5	32	17
14.9	53	38
6.2	38	19
56.7	41	28
2.8	39	14
1.5	45	19
35.7	37	13
102.7	31	14
69.1	25	16
14.1	44	19
2.7	77	98
6.4	101	121
6.3	47	16
4.1	34	15
60.3	29	17
0.6	29	22
26.5	29	18
52.6	32	13
26.8	38	23
0.19	33	16
32.9	26	17
0.9	62	51
26.7	36	23
1.4	54	26
0.19	49	26
56.2	30	24
0.19	30	20
0.3	32	17
28.9	65	52
0.19	42	25
0.19	39	27
26.5	53	35
0.6	63	23
20.8	48	19

7.4	28	15
35.4	28	10
0.19	43	19
22.6	32	17
18.3	40	20
10.7	32	17
1.1	41	27
27.5	32	17
0.19	35	14
39.5	34	21
13.8	36	19
16.8	37	24
14.8	32	17
3.9	32	17
0.9	45	12
4.2	53	18
5.8	41	17
2.4	43	18
4.9	32	17
5.1	46	34
3.5	53	22
2.7	47	20
137.5	28	26
58.7	42	16
9.6	46	17
8.7	37	44
3.4	46	12
8	34	13
8.3	37	14
1.2	32	17
8	40	13
3.7	47	44
4.6	42	23
0.9	48	17
1.3	42	38
20.6	38	14
0.19	32	17
164.7	48	24
4.4	59	41
145.1	32	17
45.7	33	15
3.1	43	17
2	25	16
4.8	32	17
11.7	44	22
10.5	45	26

1.2	51	11
55.1	32	18
2.1	60	21
6.1	37	41
66.3	41	24
16	38	13
100.4	47	25
15.1	43	22
2.8	52	28
17.9	41	19
1.5	38	22
9.8	46	18
0.19	32	15
27.8	32	17
4	34	19
2.5	32	17
4.1	45	21
21.7	66	65
179.7	24	14
38.1	40	17
15.1	32	24
70.8	34	26
5.5	32	17
41.5	32	17
22.3	56	44
2.5	40	15
0.3	49	23
29.9	32	19
1	44	30
4.2	33	19
1.8	30	12
1.7	39	20
13.9	40	20
1.8	32	17
0.19	49	35
69	38	22
52.7	35	17
1.2	191	136
0.19	38	21
4.9	60	22
9.5	27	11
8.9	28	19
2.6	38	11
2.2	32	24
17.1	52	26
0.2	60	26

5.8	29	12
127	32	17
9.1	45	27
65.2	40	20
159	29	21
18.6	32	17
34.8	29	15
41.9	38	16
139.9	39	20
93.7	42	29
0.2	35	15
104.8	40	72
55.9	36	32
79	30	91
10.3	29	39
193.3	60	135
3.2	29	16
105.2	374	606
53	59	34
114.2	35	24
45.4	29	16
8.3	32	20
9.9	40	147
45.7	18	18
172.2	34	42
369.8	25	52
227.1	122	248
41.5	26	13
25.3	32	15
35.7	33	14
187	24	84
42.9	53	20
164.8	128	97
69.8	39	15
13.2	22	54
11.1	47	21
131.4	25	15
51.1	27	22
48.2	377	339
70.5	23	12
138	32	27
167.1	150	86
265.1	48	49
58.4	33	16
7.4	27	28
130.8	29	50

221.7	303	195
9.4	39	15
160.4	25	16
22.4	23	14
4.2	52	22
52.3	29	20
151.2	27	52
26.7	26	20
52.8	26	14
78.1	26	14
74.8	269	582
29.3	26	16
0	240.5	28
169.1	26	22
55.9	68	219
11.6	34	26
39.5	25	16
149	20	9
128.8	125	36
76.9	43	39
107.2	37	57
163	23	22
66.3	19	22
168.5	42	32
73.6	21	14
331.2	24	74
52.8	44	24
267	43	86
42	23	29
140.2	21	26
143.5	212	120
4.8	34	13
44.4	25	18
284.9	24	31
59.9	63	198
8.8	38	23
18.4	34	16
42.8	250	337
51.9	28	14
253.7	84	172
146.8	34	120
50.1	170	96
176.5	31	22
155.3	76	55
76.3	430	214
53.8	20	14

31.2	36	59
125.9	63	140
126.7	36	17
180.9	38	120
20.7	85	149
101.9	21	19
11	30	61
42.3	34	15
170.1	43	63
60.5	440	195
67.2	403	147
109.7	79	83
316.7	52	97
27.5	33	17
178	26	43
28.4	33	14
116.7	27	29
0.5	165	89
128.9	22	19
235.6	27	103
242.2	24	40
66.7	23	18
47.9	2	58
212.5	24	68
25.7	23	18
103.9	41	80
39	37	27
112.1	23	45
203.1	24	15
46.9	48	39
0.5	45	40
77.6	101	217
1	94	44
99.3	29	17
242.7	23	13
1.4	48	31
66	17	30
15.7	18	7
104.4	119	51
114.8	39	24
160.3	380	216
131.1	63	125
14.1	42	17
110.7	88	263
188.6	23	10
144.9	55	80

24.5	14	75
64.9	30	79
170.9	25	35
103.6	127	255
65.6	50	72
74.4	25	73
239.5	23	22
8.8	25	26
50	30	43
1.2	31	13
162.1	45	26
71.5	53	189
55.7	72	163
184.7	22	71
13.6	41	21
59.2	223	192
0.9	117	75
208.1	35	58
94.9	176	179
75.3	34	59
68	281	732
23.1	31	17
7.5	59	53
105.7	476	206
43.3	28	45
51.8	173	165
133.7	23	11
94.6	179	231
53.6	26	16
55.2	352	248
149.9	54	58
59	26	15
77.6	46	39
94.5	42	122
122.2	38	31
243.4	72	195
69.7	22	50
53.6	29	77
128.2	24	17
34.3	23	13
44	27	29
16.4	30	21
7.8	22	17
25.4	84	110
33.2	61	47
44.6	128	361

63.2	27	16
72.2	46	35
68.1	16	12
49.4	28	39
164.9	40	61
99.6	32	30
145.9	79	332
43.4	34	13
237	24	67
27.6	29	40
34.4	66	142
45.1	37	529
42.5	25	12
99	32	27
69	38	19
122.2	66	166
68.4	25	43
60.1	23	47
78.5	21	21
49.1	28	28
35.4	30	24
56.9	128	127
92.7	225	20
3.2	26	13
41	41	21
21.3	127	277
24.91	26	31
34.2	42	24
100.7	34	15
118.21	21	15
26.1	51	37
32.8	36	103
291.8	30	118
111.3	20	19
69.3	19	38
96	35	18
9.1	39	37
60	28	23
126.5	71	46
38.7	35	21
68.7	30	30
40.5	48	42
40	23	18
101.2	66	35
79.1	31	74
42.4	42	33

24.9	27	19
22.9	43	36
43.1	29	16
133.5	31	36
0.8	187	179
67.8	75	252
64.9	23	16
67.6	251	103
205.3	21	143
2.9	32	10
248.6	70	8
48.1	28	11
111.9	29	56
1.8	28	80
195.8	27	57
32.7	28	20
220.7	24	20
242.8	50	73
9.6	21	17
25.3	34	24
32.5	138	149
3.1	29	14
16.3	37	22
37.4	27	13
13	28	11
218.5	29	48
1	38	43
76.2	29	15
99.8	35	18
5.9	19	22
19.6	31	48
135.1	39	36
49.8	162	306
71.4	30	36
63	75	32
17.7	27	10
104.7	31	15
54.6	38	32
74	123	350
108.4	23	66
172.6	43	82
273.2	27	31
8.6	96	102
48.8	28	18
45	199	246
0.2	45	19

50.9	127	62
37.2	34	19
59.2	26	41
55.9	41	22
248.1	48	71
39.3	202	62
112.3	27	12
54.4	108	47
19.62	70	164
14.7	25	11
74.7	22	5
63.3	28	17
1.2	35	16
32.1	35	19
194.2	306	90
21.53	28	12
136.3	27	17
225.4	42	26
79.4	20	11
12.5	45	27
161.56	78	103
96.9	20	43
78.43	29	19
179	88	82
16.7	32	26
177.2	28	16
107.5	111	263
135.4	39	22
31.7	21	11
54.7	23	39
57.4	31	169
70.8	72	121
354	22	30
26.8	68	106
4.9	323	226
5	28	48
60.1	55	213
78.11	40	27
178.5	76	117
122	134	95
12.3	38	14
43.7	33	37
6.66	25	7
116.9	20	17
0.7	32	27
71.5	56	35

51.8	25	10
184.8	126	169
6.5	41	89
188.6	78	149
114.1	842	387
29.7	102	52
13.4	35	19
43.4	30	42
73.9	155	336
153.8	60	59
133.2	58	169
28.6	68	166
106.2	60	34
52.1	26	34
35.4	27	21
59.1	22	14
52.4	34	14
37.7	26	14
50.6	133	64
13.2	32	35
1.69	25	18
9.3	30	16
3.3	31	18
196.4	30	108
25.7	34	50
5.4	35	25
114.2	220	157
117.3	130	285
1.19	28	9
30.3	97	109
28.6	35	49
38.1	24	13
14	34	18
23.3	40	21
142	502	762
118.59	643	576
3.59	26	7
92.9	38	92
181.9	138	106
108.4	121	153
10.3	31	16
17.8	73	41
104.5	189	332
39.7	105	261
5.5	36	21
17.9	42	41

30.9	33	16
135.9	32	22
81.6	59	46
31.2	25	15
0.3	27	15
112	27	16
58.3	28	18
33.5	360	237
172.5	28	123
37.2	28	12
86.5	51	71
104.7	138	339
98.9	67	55
71.1	26	12
68.1	35	133
131.3	45	33
58.3	32	23
79.6	92	139
75.5	29	12
44.8	36	34
51	33	16
35	23	11
111.7	187	182
84.8	42	92
104.5	40	21
70.1	125	242
108.7	37	22
22	56	35
69	35	12
59.3	122	102
0.3	75	15
34.5	30	294
33.6	23	30
36.3	98	248
134.7	50	102
13.8	60	20
61.8	31	23
3.8	48	23
59.8	20	28
79.2	44	21
28.1	32	34
237.8	31	44
62.4	58	78
103	23	10
8.1	39	13
62.5	24	17

55	58	43
118.8	22	16
9.7	27	19
118.6	86	117
42.7	27	29
24.5	127	92
103.3	45	47
126.8	28	14
33	167	504
71.8	46	34
78.8	91	77
24	238	279
38.6	28	15
291.1	41	16
123.3	18	22
51	97	279
24.3	206	286
130.8	29	50
101.1	140	262
196.1	39	70
40.8	35	20
28.9	43	21
84.1	19	22
59.8	119	77
98.2	71	50
69.8	271	573
60.1	24	117
99.1	47	26
73.6	415	497
28.7	25	14
36.8	225	197
1.4	27	16
14.9	31	91
58.4	38	14
3.1	36	13
4.2	57	24
8	52	20
136.7	32	24
72.4	124	322
223.7	113	96
79.8	20	13
127.3	20	48
137	21	18
13.6	25	55
58.5	75	52
23.2	34	16

1.24	30	15
143.47	32	48
119.7	47	145
2.7	25	13
0.2	75	34
0.3	25	18
51.9	39	62
2.54	51	33
0.2	43	25
2.4	41	24
37.1	25	11
38	72	68
4.8	28	15
77.05	31	26
31.3	27	18
0.8	37	17
65	32	18
59.05	42	108
12.1	40	19
57.8	40	48
108.1	27	26
56	26	15
5.4	36	9
22	49	57
62.49	36	22
12.21	22	11
197.66	15	49
26.3	38	23
16.4	34	16
118.2	26	62
50	42	54
77.2	31	14
26.5	25	12
34.8	35	25
37	33	19
6.7	103	60
84.2	40	54
162.4	42	74
11	122	63
103	28	120
104.2	21	15
10.8	34	28
131	22	26
211.4	94	66
83	51	210
18.5	36	13

59.4	31	18
42.1	46	30
71.4	43	126
66	30	24
178	41	49
28	25	86
70	43	19
202.3	24	27
176.4	33	22
20.1	42	53
38.1	16	48
23.9	37	72
105.8	33	60
187	22	35
39.7	34	35
185	21	87
7.62	64	69
11.4	197	159
64.9	22	13
8.8	30	12
8.7	41	29
9.7	32	44
11.3	41	51
186	31	95
39.7	26	36
20.1		
69.1	18	106
28.9	32	26
17.6	21	10
25.8	29	31
18.2	29	53
131	35	18
103	30	8
29.8	21	14
111	30	105
75.2	20	85
87.6	36	44
33.2	33	48
37.8	0	8
57	35	74
168.9	51	40
41.4	26	23
52.9	30	21
64.3	29	28
129	45	26
128	28	108

31.6	40	33
14.5	42	47
72.7	44	64
10.7		23
100	35	42
137	51	76
4.6	32	11
106	24	20
25.6	115	319
66.8	36	19
52	48	114
11.7	28	22
29.2	58	243
19.6	31	25
17.4	27	10
13.7	305	463
72.7	1024	146
41.4	31	22
108	35	12
125.4	23	18
49.9	25	139
45.5	33	16
12.5	35	22
15.2	38	72
71.3	22	78
48.2	470	238
38.9		38
10.5	33	58
21.6	30	111
248	19	63
249.5	16	13
51.3	29	103
26.2	44	103
101.2	27	110
78.2	31	53
149.3	36	187
21.6	69	22
25.2		16
58.2	59	32
13.4	60	75
22.4	37	49
57.6	29	18
149.8	19	16
115.7		403
131.1		77
	33	22.4

413	184
62	37.8
177	77.1
48	30.1
41	17.2
93	42.3
24	4.5
28	39.6
39	158.5
25	9.6
27.83	21.03
39	12
50	62.1
37	44.5
26	10.5
121	25.7
29	11.9
36	13.5
45	20.1
27	16.1
67	22.1
33	19.7
50	19.2
15	7.3
30	17.7
40	10.9
19	63.3
39	16.2
24	74.8
30	35.7
25	11.1
31	9.9
34	12.6
32	15.8
30	31.8
30	116.6
27	10.8
38	68.5
81	95.2
28	12.6
36	14.7
28	72
422	202.1
31	20
55	101.2
37	14.9

400	289
22	15.2
65	25.3
37	48.6
30	22.6
28	12.4
28	34.3
61	116.1
30	9.8
90	51.8
44	12.2
25	12.1
35	30.2
49	15
48	50.8
115	35.7
38	18.3
36	32.8
103	62.3
34	10.6
42	26.4
93	58.7
66	41.4
33	59.2
132	235.1
43	14.8
34	13.6
44	8.8
32	39.8
52	22.4
39	20.7
30	11.7
98	96.8
32	10.6
32	10.6
30	15.6
31	11.1
40	17.1
41	12.8
128	267.9
223	67.2
22	8.3
32	39.3
34	21.6
56	48.3
39	31.8

29	11.5
30	13.7
38	26.9
25	23.8
25	9.4
31	17.1
38	34.4
29	15.2
48	24.1
29	12.2
53	17.9
345	511.8
37	35
33	13.3
24	43.3
125	100
35.99	14.3
32	14.9
38	23.3
34	13.2
38	111.9
26	20.9
160	54.9
40	35
39	37
32	114.6
52	29.8
30	17.6
27	26.8
27	9.6
62	37
32	13.3
26	16
29	15.5
303	354.4
10	6.9
22	16.2
30	11.5
17	9.1
35	41.2
69	32.6
37	15.4
67	64.6
38	127.3
68	11.5
52	20.3

39	15.5
78	71.3
72	18.8
34	13.5
624	413.7
1062	325.1
60	38.8
27	13
41	179
47	28.6
33	16.7
45	14.4
52	21.9
27	14.1
34	19.2
27.95	10.59
24	14.5
32	17
121	52.3
30	12.7
26	8.6
46	27.6
26.18	17.01
58	23.9
412	499.3
28	31
27	15.8
27	45.1
26	12
19	10.6
33.92	10.26
