# Supplementary material for: A novel score system of blood tests for differentiating Kawasaki disease from febrile children
Source: PLoS One. 2021 Jan 22;16(1):e0244721. doi: 10.1371/journal.pone.0244721 (PMC7822339; doi:10.1371/journal.pone.0244721)
Supplement: Attachment — Submitted filename: A novel score system of blood tests in prediction Kawasaki disease from febrile children.docx [file pone.0244721.s002.docx]

**A novel score system of blood tests in prediction Kawasaki disease from febrile children**

Commentaries.

The objective of the study is to establish which routine laboratory results taken in the emergency room in children with fever could be helpful in differentiating Kawasaki disease from other febrile illness.

Is well established that diagnosis of Kawasaki disease is based on clinical signs and symptoms and the laboratory results are more helpful when there are incomplete or atypical presentations of the disease. I think that it is very bold to try to make a diagnosis of Kawasaki disease without the use of clinical picture, although it will be very useful to add a tool to guide doctors in the emergency department think of Kawasaki disease.

*Title*

We all know that diagnosis of Kawasaki disease is based on clinical signs and symptoms, and the use of a construct based on routine laboratory results will be very helpful to diagnose the disease. The objective of the score is to select which patients are more likely to have Kawasaki disease based only on blood exams, so I think it is better to change “prediction” to “for differentiating” Kawasaki disease from febrile children.

*Abstract*

As per journal guidelines, abbreviations are not recommended to be included in the abstract.

*Introduction*

It is important to establish the difference between complete and incomplete Kawasaki disease. I suggest that you correct the definition of classical or complete Kawasaki disease. (Lines 77 to 80) and make the difference of incomplete Kawasaki disease (line 80 “or fever lasting more than ….)

*Statistical Analysis*

Because the authors already have made all the analysis, they have to know which statistical tests were used and why they used it.

*Analysis process*

I think this is the most important part of the paper, and according to the proposed methods it is accurate for evaluating the objectives of the study.

*Results*

The number of patients included in the study is enough to make a good statistical analysis.

It is of note that in the univariate analysis (which one they finally used?) all except but one of the laboratory results used to compare Kawasaki disease patients with febrile patients had statistically significant differences.

Also of note, is that some of laboratory parameters that they use to try to differentiate KD from other febrile illness (eosinophil percentage. MCH, MCHC and monocyte percentage) have not been fully recognized as important in Kawasaki disease. And more important that after the multivariate analysis, that a higher eosinophil percentage is useful in recognizing KD. A z-score corrected elevated eosinophil percentage has been reported in the acute phase of Kawasaki disease has been recently reported by Kwak JH, Lee JH, Ha KS. Significance of differential characteristics in infantile Kawasaki disease. Korean Circ J 2019;49:755-765. And also in the convalescent phase after IVIG treatment. (Tremoulet AH, Jain S, Chanraseakar D, Sun X, Sato Y, Burns JC. Evolution of Laboratory values in patients with Kawasaki disease. Pediatr Infect Dis J 2011;30:1022-1026).

*Discussion*

I think it is appropriate and discuss all the important findings of the proposed model.

Although the study has the limitation that it is performed only in children of Asian race, and it needs to be evaluated in children from other countries and races.

*References*

The reference number 2 is incomplete

I suggest the author review the 2 references mentioned in the results review (Kwak and Tremoulet)

This paper adds a new score to evaluate Kawasaki disease in the emergency room by doctors not used to diagnose the disease. It is worth publishing it with small corrections
